# Microbial Cross-Talk: Unlocking the Cytochalasin Diversity from a Termite-Associated *Xylaria*

**DOI:** 10.1021/jacsau.5c01093

**Published:** 2025-12-22

**Authors:** Marie Dayras, Yaming Liu, Rebecca Kochems, Martinus de Kruijff, Sven Balluff, Sari Rasheed, Andreas M. Kany, Jennifer Herrmann, Sebastian Götze, Bernd Morgenstern, N’Golo A. Koné, Michael Poulsen, Rolf Müller, Christine Beemelmanns

**Affiliations:** † Helmholtz Institute for Pharmaceutical Research Saarland (HIPS), 28336 Helmholtz Centre for Infection Research (HZI), Campus E8.1, 66123 Saarbrücken, Germany; ‡ Deutsches Zentrum für Infektionsforschung (DZIF) e.V., Braunschweig 38124, Germany; § Inorganic Solid-State Chemistry, Saarland University, Campus Building C4 1, 66123 Saarbrücken, Germany; ∥ Unité de Formation et de Recherche en Sciences de la Nature (UFR-SN), 195203 Université Nangui Abrogoua, Station de Recherche en Ecologie du Parc National de la Comoé, 27 BP 847 Abidjan 27, Côte d’Ivoire; ⊥ Section for Ecology and Evolution, Department of Biology, University of Copenhagen, 2100 Copenhagen East, Denmark; # Pharma Science Hub (PSH), Saarland University, 66123 Saarbrücken, Germany

**Keywords:** polyketides, cytochalasan, fungal natural products, biosynthesis, metabolomics

## Abstract

Integrating organismal interaction studies with advanced genomic and metabolomic approaches offer great promise for discovering novel natural products and their derivatives, yet this strategy remains relatively unexplored. Here, we illustrate its potential by investigating a newly isolated *Xylaria* strain from a termite colony environment through combined genome and metabolome analyses, complemented by fungal–bacterial coculture experiments. Genome sequencing of the fungal strain allowed us to pinpoint a cytochalasin-related biosynthetic gene cluster responsible for the production of a portfolio of different bioactive epoxy-cytochalasins. Guided by the hypothesis of biosynthetic promiscuity of the underlying nonribosomal peptide synthetase (NRPS), we demonstrated for the first time that the NRPS can accept unnatural ortho- and meta-halogenated phenylalanine derivatives, leading to the isolation of multiple new chlorinated and brominated cytochalasin analogs. Second, based on the hypothesis that structural diversification can arise from interactions with commensal organisms, cocultivation with a termite-associated *Streptomyces* strain led to the discovery of a previously undescribed aspartic acid-containing cytochalasan derivative, designated xylachalasin A. Isotope labeling experiments revealed that bacterial catabolic activity is responsible for the modification of the fungal-derived cytochalasin. Isolated cytochalasins were also amiable for semisynthesis modifications, which was exemplified by the synthesis of bifunctional probes. Bioassays of a total of 26 isolated and semisynthesized derivatives demonstrated structure-dependent cytotoxicity in some cases with up to 3-fold log differences in potency and generally good plasma stability. Overall, our integrated approach underscores the vast potential of investigating fungal strains from underexplored ecological niches and their organismal interactions, offering new opportunities to discover novel natural products of potential therapeutic relevance and previously unrecognized biochemical processes.

## Graphical abstract

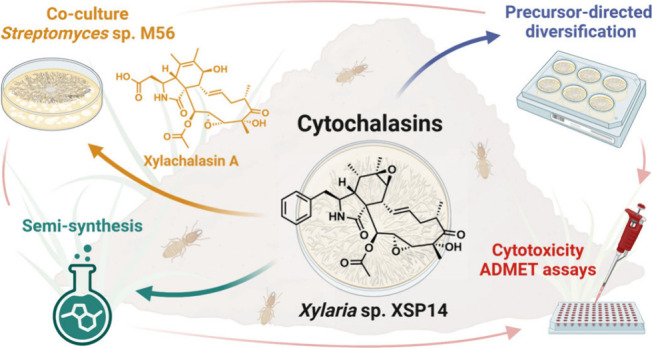

## Introduction

The farming symbiosis between termites and cultivated fungi has long fascinated researchers, not only for its ecological significance as ecosystem engineers, but also for its rich tapestry of chemical interactions.^1–3^ Termites in the subfamily Macrotermitinae (Termitidae: Blattodea) cultivate mutualistic basidiomycete fungi of the genus *Termitomyces* as their main food in the presence of a protective and catabolically active bacterial microbiome within subterranean fungal gardens thereby converting recalcitrant lignocellulosic plant material to an enriched fungal food.^4–7^ Nutrient-rich symbiotic environments inevitably attract parasitic or opportunistic species. In termite colonies, *Xylaria* species are frequently associated with decaying combs where a sit-and-wait strategy allows these coevolved fungi to monopolize combs and outcompete the fungal cultivar and protective bacteria once opportunities arise.^8,9^ Free-living *Xylaria* species, much like coevolved symbionts, frequently interact and compete with soil- and wood-associated microbiota such as Actinomycetota. To thrive in these environments, *Xylaria* and other members of the Xylariaceae biosynthesize a rich diversity of natural products, in particular antimicrobial peptides and cytotoxic cytochalasins, which are thought to improve the fitness within the competitive environment.^10^ As current knowledge on insect-associated isolates and the ecological modulation of secondary metabolism remains scarce, we investigated a newly obtained *Xylaria* isolate from a *Macrotermes bellicosus* termite colony under diverse stress-inducing cultivation conditions with focus on the production of presumably benefit-conferring cytochalasins (Table S1). De novo genome sequencing of *Xylaria* sp. XSP14 allowed us to identify a biosynthetic gene cluster (*ecy* BGC) presumably responsible for the production of epoxy-cytochalasins, and which prompted us to isolate and characterize nine natural derivatives. Building on our bioinformatic analysis, we hypothesized that the *ecy* BGC might exhibit broader substrate promiscuity than generally anticipated. By employing precursor-directed cultivation strategies, we uncovered an unusual tolerance toward 2’- and 3′-substituted phenylalanine precursors and successfully generated a broad library of unnatural and functionalized derivatives. Furthermore, we explored whether ecomimetic bacterial–fungal cocultivation^11,12^ could induce additional modifications within the cytochalasin scaffold. Here, we were able to demonstrate that a single *Streptomyces* strain (M56) can enzymatically transform fungal cytochalasins, yielding a novel and structurally distinct derivative with altered bioactivity. Lastly, we demonstrate that semisynthetic modification of the natural product scaffold enables the generation of chemical probes and structurally diverse unnatural derivatives. Comprehensive bioassays further revealed that cytochalasins exhibit structure-dependent cytotoxicity, underscoring their potential as promising leads for the development of cytotoxic therapeutic agents.

## Results and Discussion

For this study, we selected a morphologically distinct, previously unexplored *Xylaria* isolate (XSP14) (Figure 1A) and sequenced its genome using a combination of paired-end shotgun sequencing (DNBSEQ-G400) and long-read Nanopore sequencing (Oxford Nanopore Technologies, Oxford, UK) (Figures S1–S2, Table S1).^9^ Using publicly available genomes as references, we assembled a hybrid draft genome with a total haploid assembly length of 50.2 Mb and a GC content of 45.3%, comparable to that of other *Xylaria* genomes. The draft genome had high BUSCO completeness (98.6%) and 13,141 predicted proteins. An initial survey of the encoded biosynthetic gene clusters (BGCs) using Fungi-SMASH 6.0 ^13^ revealed a diverse array of polyketide synthase (PKS), nonribosomal peptide synthetase (NRPS), PKS–NRPS hybrid, and terpene biosynthetic gene clusters. Although precise BGC boundaries were difficult to define, the analysis highlighted the strain’s versatile potential for natural product biosynthesis. Phylogenomic analysis indicated that *Xylaria* sp. XSP14 grouped most closely with *Xylaria curta* Babe10 and not with termite-associated members of South African *Xylariales* (Figure S2, Table S1). Here, we acknowledge that *Xylaria* species, particularly those not associated with *Macrotermes natalensis*, have been shown to colonize fungus gardens of various termite genera.^9^ Moreover, individual termite colonies often harbor multiple *Xylaria* species, as strict host specificity appears to be lacking. Given the phylogenetic placement of *Xylaria* sp. XSP14, it is therefore plausible that this strain belongs likely not to one of the known clades of termite-adapted specialists.^9^ Given the relatively limited sampling of *Xylaria* in the Global South, it is expected that substantial, yet-undiscovered diversity remains.

**Figure 1:**
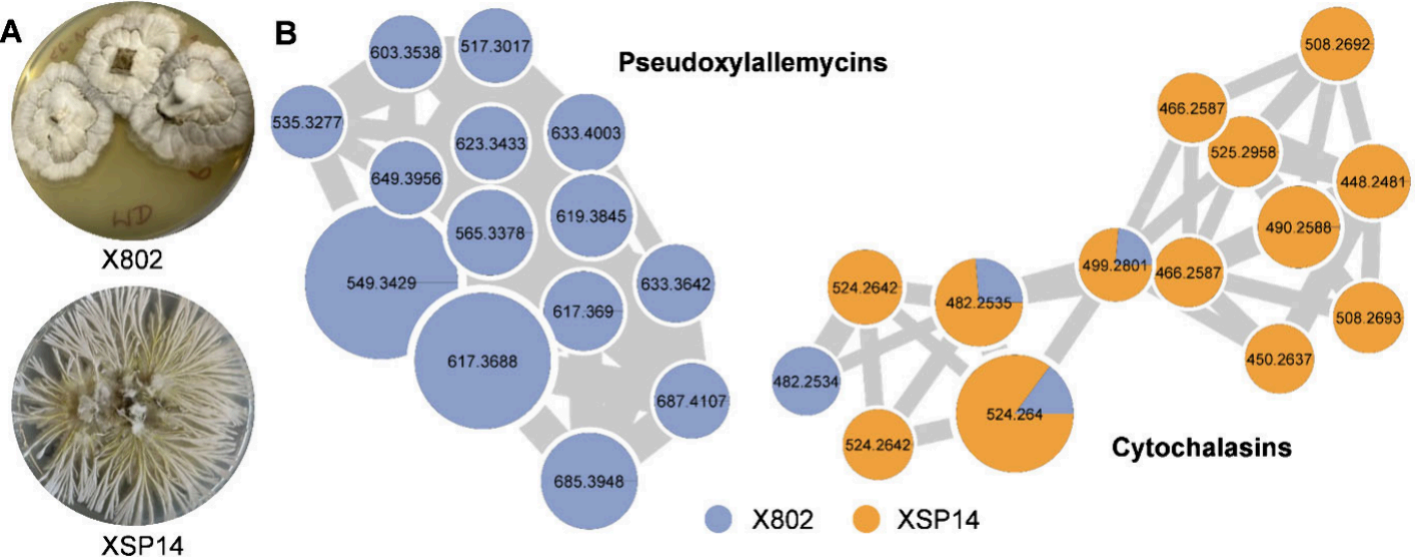
A) Pure cultures of *Xylaria* sp. X802, and XSP14 on PDA. B) Expanded GNPS-feature-based molecular network clusters of cytochalasins and pseudoxylallemycins from *Xylaria* sp. XSP14, and X802 culture extracts.

## Metabolomic Survey of *Xylaria* sp. XSP14 Uncovers a Broad Repertoire of Cytochalasins

For a comparative metabolomic analysis, *Xylaria* sp. XSP14 and the cytochalasin-producing reference strain *Xylaria* sp. X802 were grown in different growth media (Table S2).^8,14–16^ Biomass and supernatants were collected at various time points using solid-phase extraction methods. The enriched metabolic extracts were analyzed using ultrahigh performance liquid chromatography-tandem mass spectrometry (UHPLC-MS^2^). Obtained MS^2^-data was dereplicated using MzMine^17,18^ and the Global Natural Product Social Molecular Networking Web platform (GNPS) (Figure 1).^19^ Under axenic conditions *Xylaria* sp. X802 produced two major compound families pseudoxylallemycins (*m*/*z* 549.343, 617.369, and 685.395), and a cluster assigned to cytochalasin derivatives (*m*/*z* 482.254 and 524.264) (Figure 1B).^8,14^ Direct comparison uncovered that isolate XSP14 nearly exclusively produced cytochalasins, including ones with partially unknown molecular ion features and compositions (Figure 1B).

To elucidate the potentially unreported derivatives, *Xylaria* sp. XSP14 was cultivated in monoculture in 4 L potato dextrose broth (PDB) for 4 weeks. After separation of the supernatant from the mycelium, the former was extracted using Diaion HP-20 resin, while the mycelium was freeze-dried and extracted with methanol. Both extracts were purified using multiple rounds of MS-guided C_18_-column chromatography to yield the previously unreported deacetyl-18-desoxy-19,20-epoxycytochalasin Q (**1**) (*m*/*z* 466.2583 [M + H]^+^) as well as other known cytochalasin features (Figure 2). The planar structure of **1** was elucidated via 2D nuclear magnetic resonance (NMR) experiments (Table S3). ^1^H–^1^H correlation spectroscopy (COSY) analysis revealed six spin-systems, which could be connected using information derived from ^1^H–^13^C heteronuclear multiple bond correlation (HMBC) spectra (Figure 2). ^1^H–^1^H COSY correlation between H-18 and H-23 confirmed the lack of a hydroxyl group at C-18. The relative configuration of the 19,20-epoxy group of **1** was determined to be 19­(βH),20­(αH)-epoxy (*trans*) judging from the nuclear Overhauser effect (NOE) observed between H-20/H-21 and H-19/H-23. Key ^1^H–^1^H 2D NOE spectroscopy (NOESY) correlations led us determine the relative configuration of **1** as 3S*, 4R*, 5S*, 6R*, 7S*, 8R*, 9R*, 16S*, 18S*, 19S*, 20S*, 21S*.

**Figure 2:**
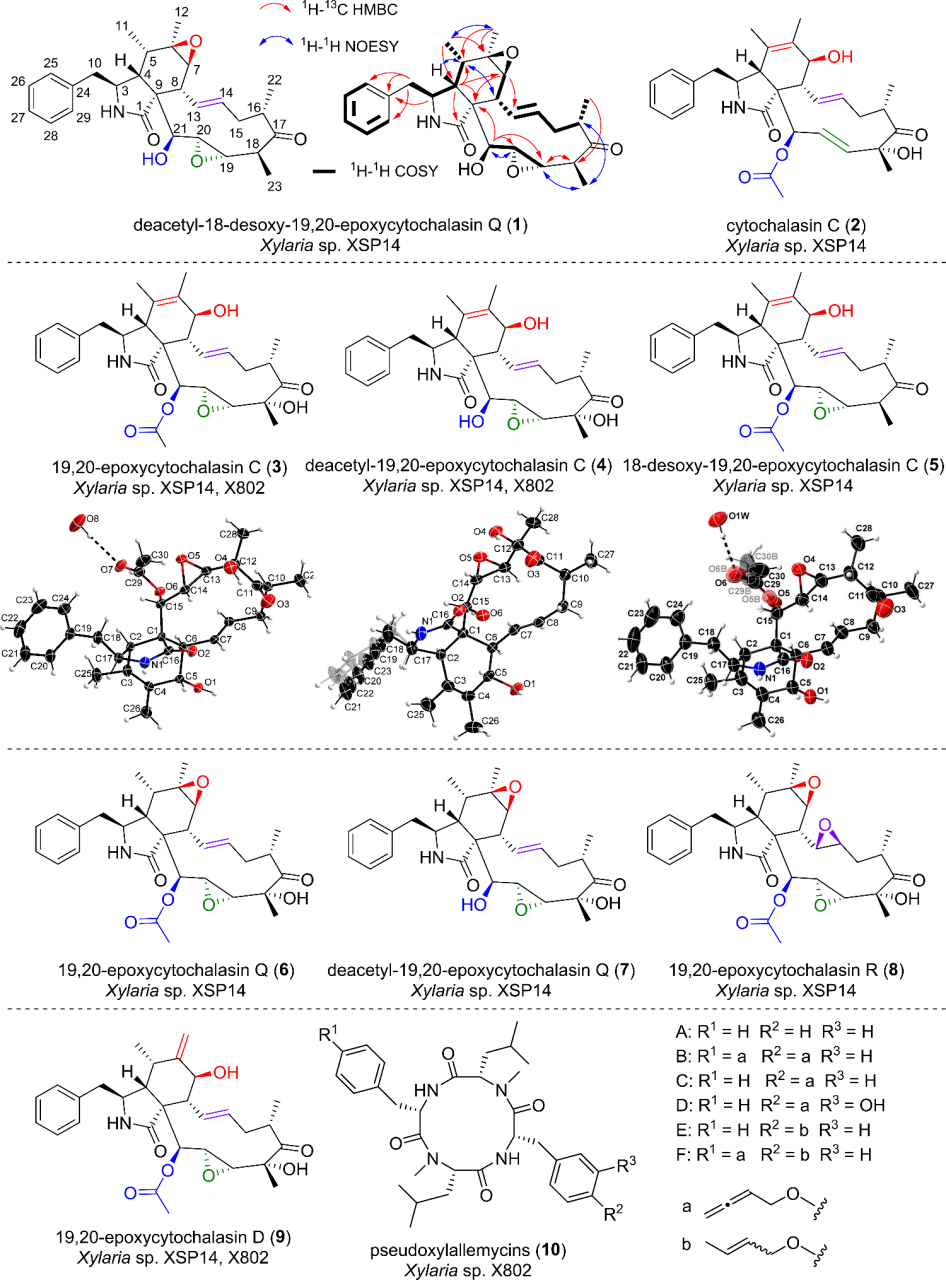
Structures of isolated cytochalasins **1**–**9** and pseudoxylallemycins (**10**) from *Xylaria* sp. XSP14 and X802, including key 2D NMR correlations detected for the newly described derivative deacetyl-18-desoxy-19,20-epoxycytochalasin Q (**1**), and crystal structures (ORTEP plot with 50% probability level) of compounds 19,20-epoxycytochalasin C (**3**), deacetyl-19,20-epoxycytochalasin C (**4**), and 18-desoxy-19,20-epoxycytochalasin C (**5**).

Purification of the supernatant yielded seven additional known congeners of this natural product class: cytochalasin C (**2**),^19^ 19,20-epoxycytochalasin C (**3**),^20^ deacetyl-19,20-epoxycytochalasin C (**4**),^20^ 18-desoxy-19,20-epoxycytochalasin C (**5**),^21^ 19,20-epoxycytochalasin Q (**6**),^22^ deacetyl-19,20-epoxycytochalasin Q (**7**),^20^ and 19,20-epoxycytochalasin R (**8**).^23^ In addition, 19,20-epoxycytochalasin D (**9**)^24^ was exclusively isolated from methanolic extracts of the mycelium (Figure 2). Fortunately, we were able to obtain single crystals of 19,20-epoxycytochalasin C (**3**),^20^ deacetyl-19,20-epoxycytochalasin C (**4**),^20^ 18-desoxy-19,20-epoxycytochalasin C (**5**),^21^ suitable for X-ray crystallography, which allowed to verify the 3D structure of this compound family (Figure 2). Here, it is worth noting that during the isolation and purification process, 19,20-epoxycytochalasin Q (**6**) and 19,20-epoxycytochalasin C (**3**) were always obtained as a mixture, and that the product ratio was dependent on the use of formic acid during the purification process. This led us to hypothesize that 19,20-epoxycytochalasin C (**3**) and other cytochalasin derivatives carrying an allylic alcohol moiety are likely artifacts of the purification process and not enzymatically transformed via a yet undetermined hydrolase.^22,23^ To test the influence of acids on the core scaffold of 19,20-epoxycytochalasin Q (**6**), we added formic acid in dichloromethane (DCM) and stirred the reaction mixture at room temperature (r.t.) (Figure S3). Indeed, 19,20-epoxycytochalasin Q (**6**) (RT = 6.69 min) was cleanly converted into 19,20-epoxycytochalasin C (**3**) (RT = 6.49 min), strongly suggesting that 19,20-epoxycytochalasin Q type can easily rearrange under mild acidic conditions present e.g. during purification (Figure S3). Since acidic conditions can lead to artifacts during isolation, purification strategies should account for this possibility.

## Comparative Analysis of the Cytochalasin Biosynthetic Pathway

The remarkable structural diversity of the cytochalasan superfamily (Figure 3) arises from three principal biosynthetic variations: (1) the substrate specificity of the NRPS adenylation (A) domain, which defines the amino acid backbone of the isoindole scaffold; (2) the iterative activity of the PKS module, which shapes the macrocyclic ring; and (3) the action of cytochrome P450 enzymes and associated oxidoreductases or monooxygenases, which introduce oxidative decorations to the scaffold.^22^

**Figure 3:**
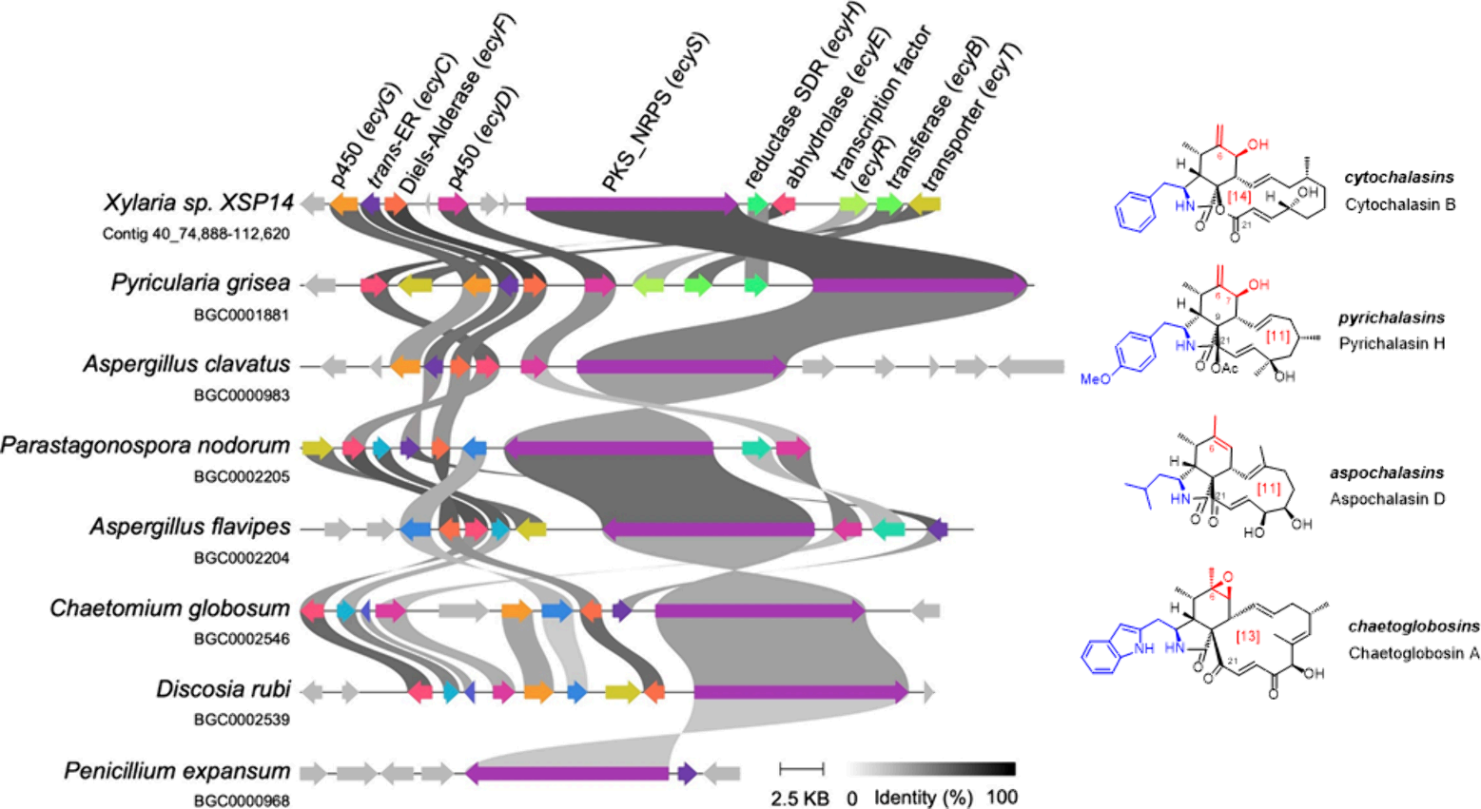
**Comparative synteny analysis of cytochalasan-related biosynthetic gene clusters from the MIBiG database.** Each BGC is labeled with the source species and its corresponding contig location or MIBiG ID. Genes within the clusters are compared, and homologous gene pairs sharing more than 30% sequence identity are connected. The analysis and visualization were performed using *clinker*. Chemical structures of known representatives of the major cytochalasan subfamilies (cytochalasins, pyrichalasins, aspochalasins, and chaetoglobosins) are depcited next to the encoding BGC. Abbreviations: BGC, biosynthetic gene cluster; MIBiG, Minimum Information about a Biosynthetic Gene cluster; SDR, short-chain dehydrogenase/reductase; P450, cytochrome P450 monooxygenase; PKS-NRPS, polyketide synthase/nonribosomal peptide synthetase.

To obtain deeper insights into the underlying structural scaffold variability, we compared first the architecture of BGCs across the cytochalasan-encoding gene cluster family (GCF), thereby providing a framework to rationalize our molecular findings. To this end, we conducted manual BLAST analyses to identify orthologous BGCs, using the characterized NRPS–PKS hybrid cluster *ccs* from *Aspergillus clavatus* and the orthologous cluster *cytA* from *Xylaria* sp. X802 (Figure 3). The BGC identified in *Xylaria* sp. XSP14 (*ecy* BGC) was subsequently compared with experimentally validated homologous BGCs available in the MIBiG database.^22,23^

The closest homologue of the *ecy* cluster outside the Xylariaceae was the *pyi* cluster from *Magnaporthe grisea* (anamorph: *Pyricularia grisea*),^24,25^ with gene pair identities ranging from 41% to 78%. The core biosynthetic genes, including PKS-NRPS,^26^ *trans*-acting enoylreductase (*trans*-ER), α,β-hydrolase, and Diels–Alderase,^27,28^ remain mostly conserved across these biosynthetic gene clusters with some exceptions. While the BGC encoded in *Discosia rubi* lacks the *trans*-ER, the BCG encoded in *Penicillium expansum* is missing both the α,β-hydrolase and Diels–Alderase. Tailoring enzymes, such as cytochrome P450 enzymes, Short-chain dehydrogenases/reductases (SDRs), and transferases, along with regulatory proteins like transcription factors and transporters, exhibited greater variability across species in both protein identity and gene content.

We subsequently compared the *ecy* BGC with cytochalasin biosynthetic gene clusters from the same genus, *Xylaria*, as well as from the closely related genus *Hypoxylon* (Figure 4), both of which are recognized producers of cytochalasins. The cytochalasin BGCs from *Xylaria* species displayed high similarity in terms of both protein identity and gene organization.^29–34^ The BGCs from *Xylaria* sp. XSP14 and *Xylaria* sp. X802 also showed strong similarity to the experimentally characterized *Hypoxylon fragiforme* BGC,^35^ with their PKS-NRPSs exhibiting 79% identity to the PKS-NRPS from *H. fragiforme*. The tailoring enzymes in the *Xylaria* gene clusters share 40% to 74% identity with their *H. fragiforme* orthologs. Notably, in the XSP14 BGC, the P450 *ecyG* and *trans*-ER *ecyC* genes are separate, a pattern observed in all other BGCs analyzed except for the X802 BGC, where these genes have been annotated as a single entity.

**Figure 4:**
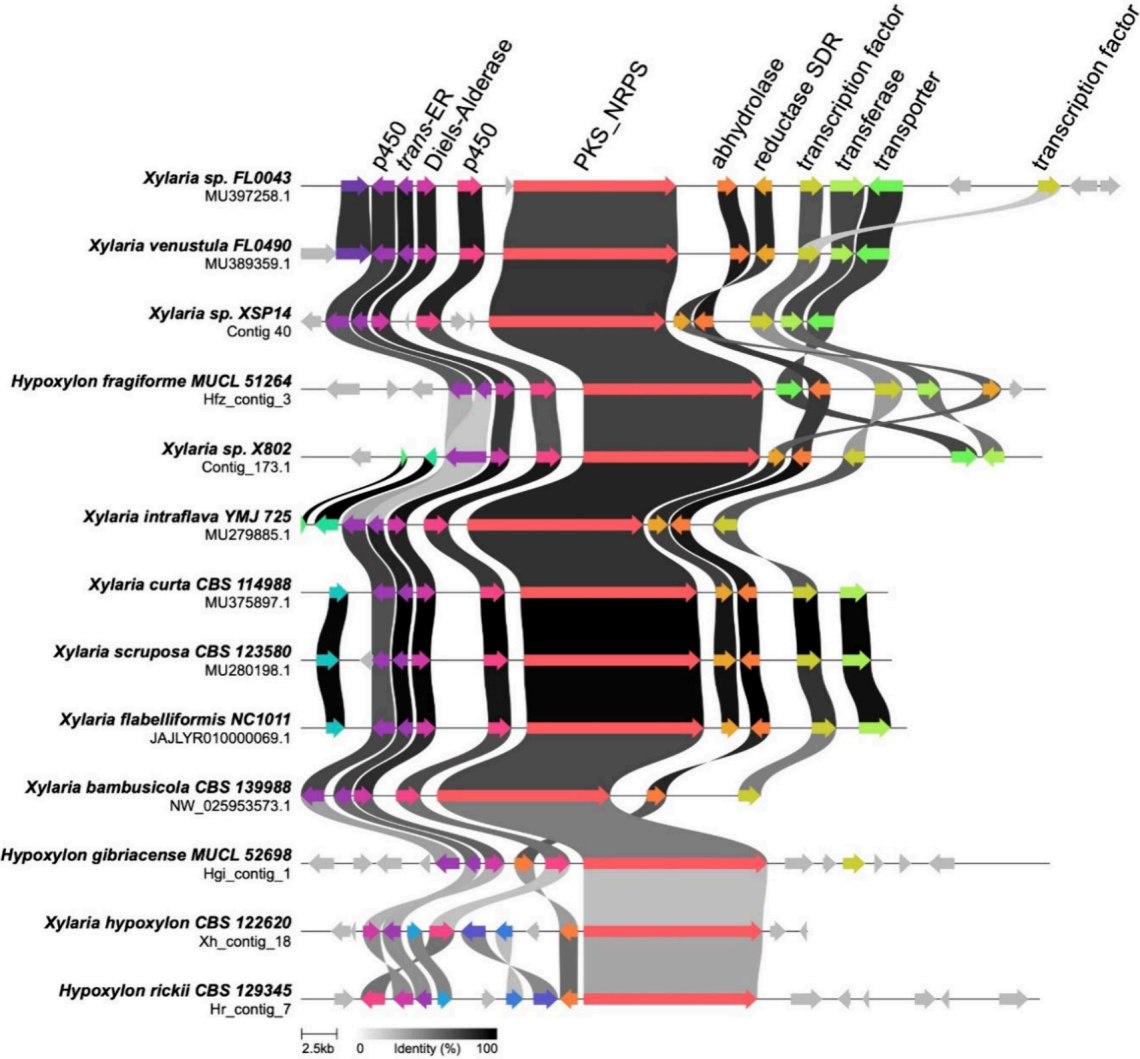
**Comparative synteny analysis of the** *ecy* **BGC and other cytochalasin-encoding BGCs within the genera** *Xylaria* **and** *Hypoxylon* **.** Each BGC is labeled with the source species and its contig location. Genes within the clusters are compared, with gene pairs sharing more than 30% sequence identity shown as linked. Analysis and visualization were performed using *clinker*. Abbreviations: SDR, short-chain dehydrogenase/reductase; P450, cytochrome P450 monooxygenase; PKS_NRPS, polyketide synthase/nonribosomal peptide synthetase.

## Harnessing the Intrinsic Promiscuity of the *ecy* Pathway

While wild-type strain *Xylaria* sp. XSP14 exclusively produced 19,20-epoxy-cytochalasin variants, we speculated that the adenylation domain of the NRPS might possess inherent substrate promiscuity that could be exploited alongside the native producer’s biosynthetic machinery. To investigate this, we analyzed the 10–amino acid Stachelhaus code of the adenylation (A) domain from *Xylaria* sp. XSP14 (Figure S4), a well-established predictive motif for substrate specificity of A domains.^49^ Although originally established in bacterial systems,^50^ analogous motifs can also be identified in fungal A domains. However, their predictive reliability is often lower, reflecting the divergent evolutionary trajectories of fungal megasynthetases and the limited structural and biochemical characterization of fungal A-domain specificities.^51^ In case of the cytochalasin NRPS, analysis of the 10-residue signature sequences across gene clusters showed some agreement between predicted A-domain substrate specificities and the amino acids incorporated into the scaffold, while ambiguities remained (Figure S4). Owing to the scarce experimental data on fungal A-domain specificity, and the potential for discovering new cytochalasin frameworks through enzymatic promiscuity, we employed a precursor-directed diversification strategy of the cytochalasin-producing *Xylaria* sp. XSP14, along with the known producer of tryptophan-containing chaetoglobosins *Chaetomium globosum* CBS 148.51 ^36^ to probe A-domain flexibility and the adaptability of the native NRPS–PKS pathway (Tables S4–S6, Figures S5–S7).^37^

Recognizing that wild-type titers are constrained by amino acid tolerance and metabolic flux variability, both strains were cultured on media supplemented with different concentrations of functionalized phenylalanine or tryptophan derivatives. Cytochalasan production and unnatural amino acid incorporation efficiency were then analyzed by HRMS^2^ and GNPS-based molecular networking, leveraging the similarity of *m*/*z* fragmentation patterns of the cytochalasan scaffold.

Contrary to the expected, screening over 30 amino acids revealed that *C. globosum* CBS 148.51 allows only minor incorporation of unnatural amino acids and changes in the chaetoglobosin scaffold (Table S5). However, *Xylaria* sp. XSP14 exhibited a remarkable capacity to accept and incorporate 2′- and 3′-substituted phenylalanine derivatives into the cytochalasan backbone (Table S6). Notably, *meta*-substituted analogs such as 3-chloro- and 3-bromo-l-phenylalanine were preferentially incorporated over the native phenylalanine. In addition, supplementation of *ortho*-substituted phenylalanine led to the production of new cytochalasin-related features that were likely derived from a different oxidation pattern of the core scaffold as deduced from the *m*/*z* signature (Figures S5–S7).

Guided by our analytical results, we substantiated the MS-based predictions through scale-up fermentations of *Xylaria* sp. XSP14 enriched with 3-chloro-, 3-bromo-, or 2-bromo-l-phenylalanine, which afforded milligram-scale yields of 2′- and 3′-substituted phenylalanine cytochalasin analogues, structural variants not previously accessible via heterologous systems or mutational or synthetic modifications (Table S8–S11).^38^

In total, we were able to isolate and fully characterize 11 new halogenated cytochalasins: *m*-chloro-19,20-epoxycytochalasin C (**11**), *m*-chloro-deacetyl-19,20-epoxycytochalasin C (**12**), *m*-chloro-cytochalasin Q (**13**), *m*-chloro-19,20-epoxycytochalasin Q (**14**), *m*-chloro-deacetyl-19,20-epoxycytochalasin Q (**15**), *m*-chloro-18-desoxy-19,20-epoxycytochalasin Q (**16**), *m*-bromo-deacetyl-19,20-epoxycytochalasin C (**17**), *m*-bromo-19,20-epoxycytochalasin Q (**18**), *m*-bromo-deacetyl-19,20-epoxycytochalasin Q (**19**), *o*-bromo-19-hydroxy-21-oxocytochalasin Q (**21**), and *o*-bromo-19,20-epoxy-21-oxocytochalasin Q (**20**) (Figure 5). The structures of compounds **11–19** were readily established by comparing their ^1^H and ^13^C NMR chemical shifts to those of their nonhalogenated counterparts or closely related cytochalasin congeners previously isolated (compounds **1**–**9**), and further supported by the X-ray crystal structure of *m*-chloro-19,20-epoxycytochalasin C (**11**). Additionally, we observed that *Xylaria* sp. XSP14 enzymatically modified the supplemented unnatural amino acids, which included reduction and glycosylation (Figure S8, Tables S11–S13).

**Figure 5:**
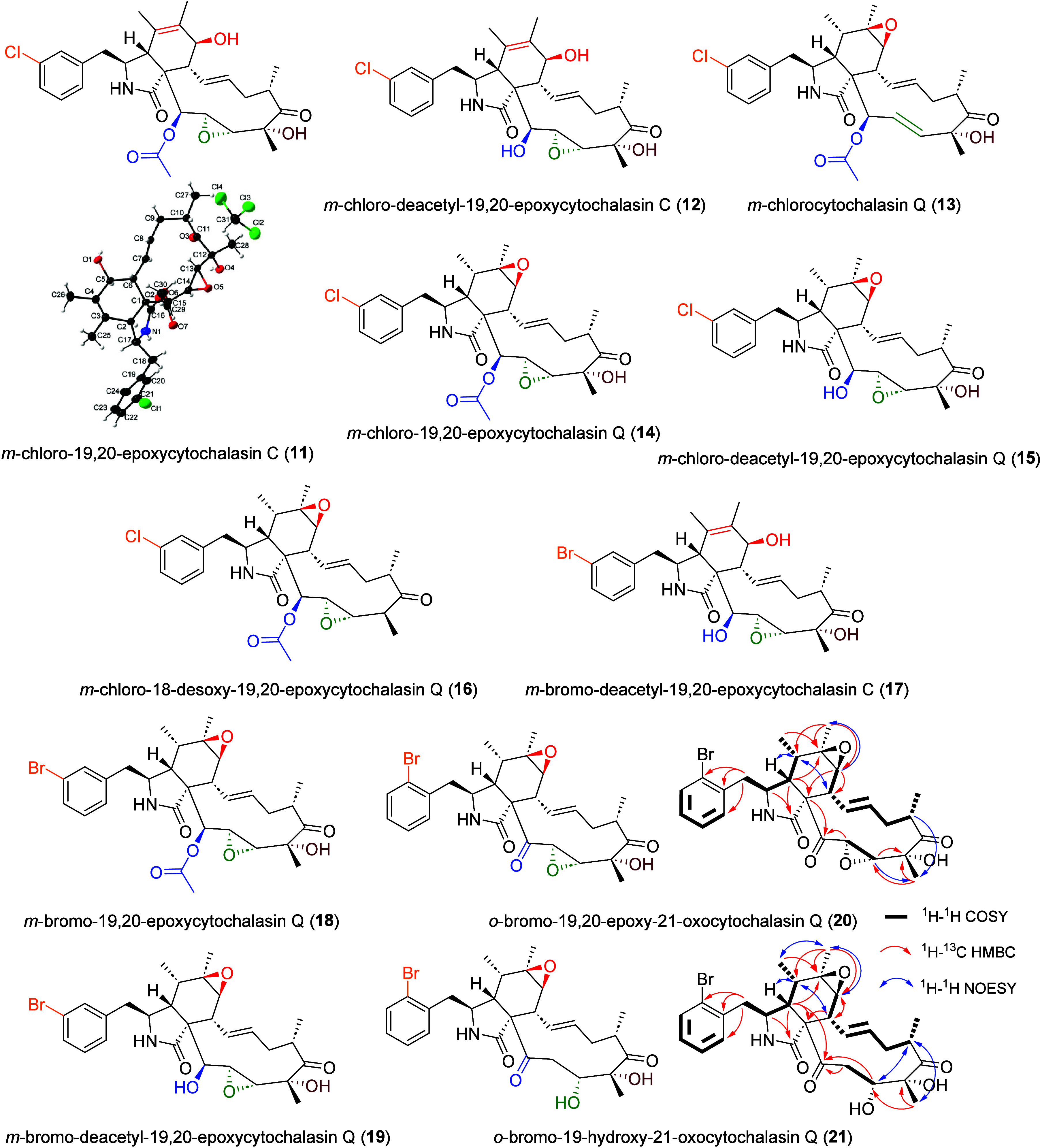
Structures of isolated cytochalasins from *Xylaria* sp. XSP14 cultures supplemented with 3-chloro, 3-bromo, and 2-bromo-l-phenylalanine.

Here, it is worth highlighting that compounds **20** and **21** showed an altered yet undescribed cytochalasin core structure. The molecular formula of compound **20**, C_28_H_32_BrNO_6_, was deduced from ESI­(+)­HRMS data (*m*/*z* 588.1481 [M + H]^+^, calcd. for C_28_H_33_BrNO_6_, 558.1486). Characteristic isotope pattern in the HRMS spectrum of **20** confirmed the presence of a bromine in the molecule. The ^1^H NMR and ^13^C NMR spectra of **20** were close to the reported and isolated compound deacetyl-19,20-epoxycytochalasin Q (**7**), differing by the replacement of the hydroxyl group at C-21 by a ketone and the presence of a bromine in *ortho* position of the aromatic ring, characterized by the well-known *ortho*-substitution ^1^H NMR aromatic region pattern. By 2D NMR experiments, four spin–spin coupling systems were confirmed by ^1^H–^1^H COSY and the planar structure was established by HMBC correlations (Figure 5), with in particular the key HMBC correlations H-20/C-21 and H-4/C21 linking the 19,20-epoxy and the isoindole ring to the ketone, closing the macrocycle. Key ^1^H–^1^H NOESY correlations led us determine the relative configuration of **20** as 3S*, 4R*, 5S*, 6R*, 7S*, 8R*, 9R*, 16S*, 18R*, 19R*, 20R*. The molecular formula of compound **21**, C_28_H_34_BrNO_6_, was deduced from ESI­(+)­HRMS data (*m*/*z* 560.1638 [M + H]^+^, calcd. for C_28_H_35_BrNO_6_, 560.1642). Characteristic isotopic pattern of a bromine in the HRMS spectrum of molecule **21** was again observed and the ^1^H NMR and ^13^C NMR spectra of **21** were really close to **20**, differing by the presence of an hydroxyl group at C-19 resulting from the opening of the 19,20-epoxy moiety. 2D NMR experiments and comparison of ^1^H NMR and ^13^C NMR chemical shifts with **20** confirmed the planar structure of **21**. Key ^1^H–^1^H NOESY correlations, in particular H-16/H-19, led us determine the relative configuration of **20** as 3S*, 4R*, 5S*, 6R*, 7S*, 8R*, 9R*, 16S*, 18R*, 19R*.

## Coculture Based Diversification and Isolation of Xylachalasin A

Inspired by the inherent promiscuity of NRPS-based biosynthetic machinery and previous studies on *Xylaria* that revealed altered BGC transcription profiles in response to external stressors, we hypothesized that the presence of competing organisms might impact the secondary metabolite profile and with this the cytochalasin profile. To test this, we selected the termite-associated *Streptomyces* sp. M56 strain, known for its antifungal activity and anticipated to serve as a representative substrate competitor (Figure 6A).^39^ At defined incubation time points, extracts of sections from the bacterial and fungal colonies, as well as from their interaction zone, were analyzed by LC-HRMS^2^. Molecular networking analysis clearly revealed that cocultivation induced distinct changes in the molecular ion cluster composition associated with the cytochalasin compound family with detectable new molecular ion features forming a distinct subcluster within the molecular network (e.g., *m*/*z* 492.2224, RT = 5.15 min, Figure 6B).^40,41^ For characterization of the newly formed cytochalasin variant (RT = 5.15 min, *m*/*z* 492.2224), *Xylaria* sp. XSP14 and *Streptomyces* sp. M56 were further cocultivated on solid medium (5 L PDA agar, approximately 100 plates). Metabolites secreted in the interaction zone were purified using a strategy similar to that described above. The ^1^H and ^13^C NMR spectra of the new compound closely resembled those of the known 19,20-epoxycytochalasin C (**3**), differing only by the absence of the characteristic phenylalanine moiety and the presence of aspartic acid attached to C-10, as confirmed by HMBC correlations (Figure 7A). Due to its characteristic new structural features, we named the new compound xylachalasin A (**22**). Given the unprecedented presence of an aspartic acid within the xylachalasin A backbone, we hypothesized that its origin might result from an oxidative degradation and potentially detoxification process of a precursor cytochalasin, analogous to a process previously described in a coculture study of *Aspergillus flavipes* and*Chaetomium globosum* *.* ^42^

**Figure 6:**
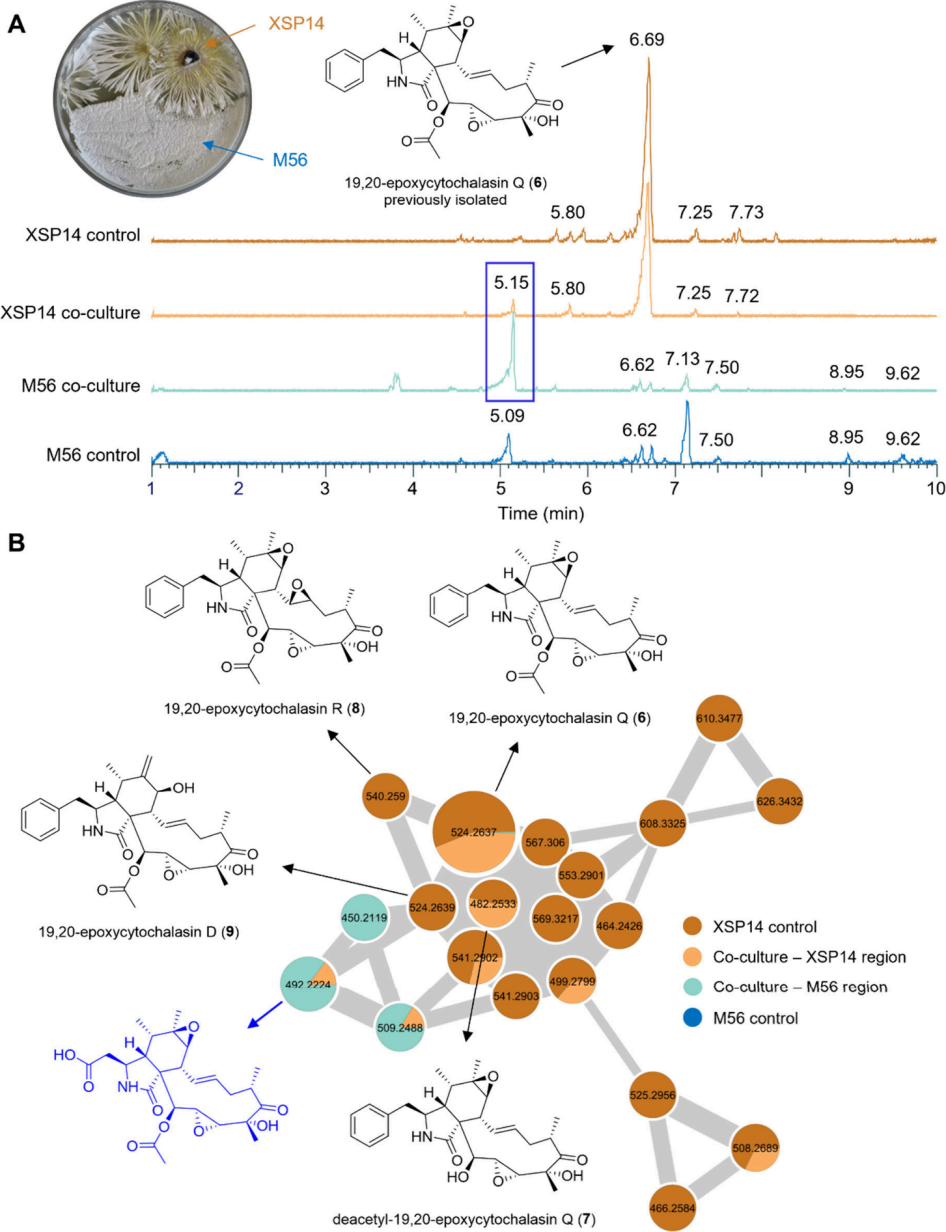
A) Photograph of a coculture plate showing *Xylaria* sp. XSP14 and *Streptomyces* sp. M56; comparison of UHPLC-HRMS chromatograms of *Xylaria* sp. XSP14 culture extract, coculture extracts of *Xylaria* sp. XSP14 and *Streptomyces* sp. M56, and *Streptomyces* sp. M56 culture extract. B) Expanded GNPS-feature-based molecular network cluster of cytochalasins from *Xylaria* sp. XSP14 culture extract, coculture extracts of *Xylaria* sp. XSP14 and *Streptomyces* sp. M56, and *Streptomyces* sp. M56 culture extract.

**Figure 7:**
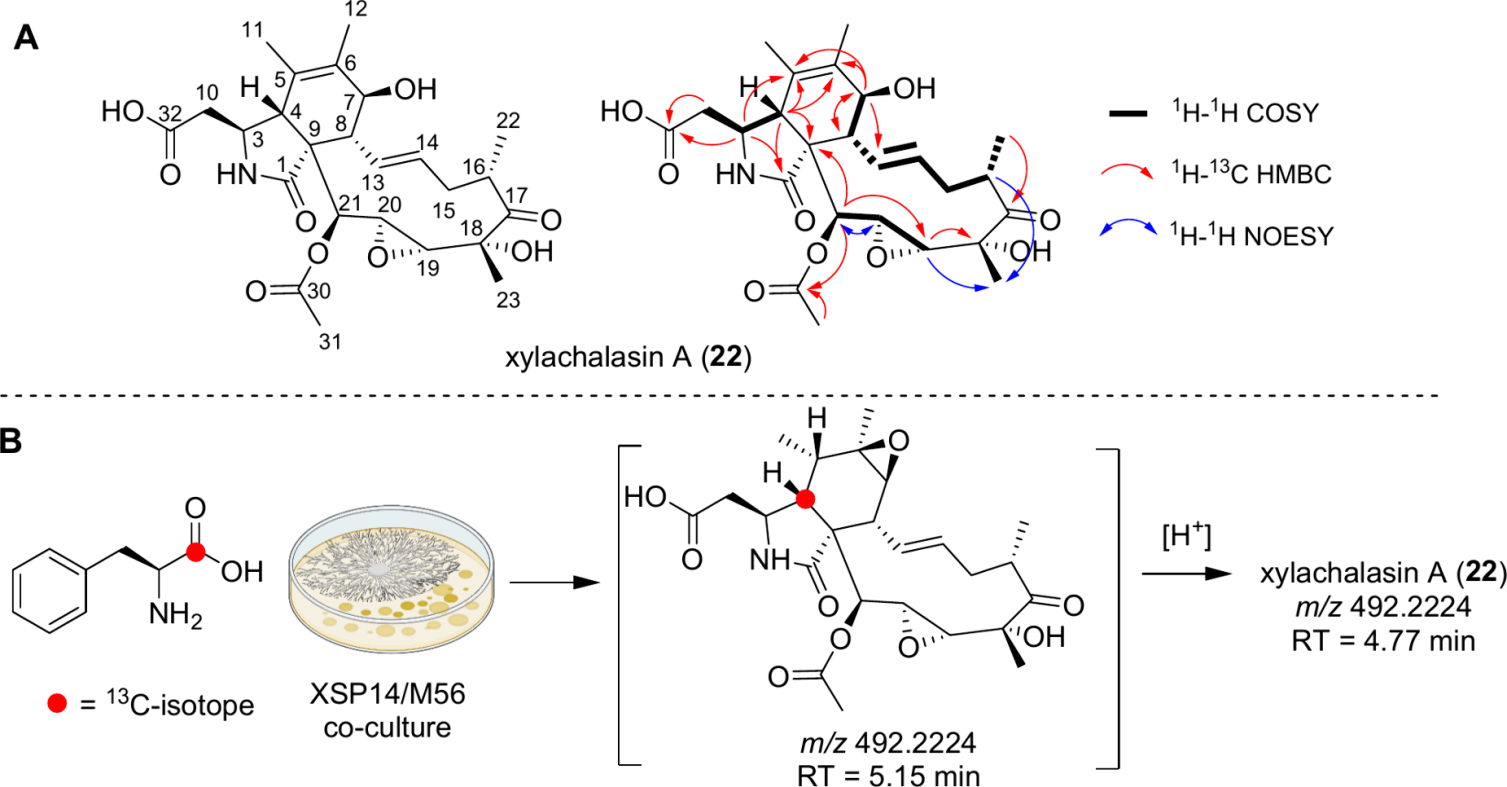
A) Structure of xylachalasin A (**22**), which was isolated from the coculture of *Xylaria* sp. XSP14 and *Streptomyces* strain M56, including key 2D NMR correlations used for structure elucidation. B) Stable isotope feeding experiment using l-phenylalanine, which is incorporated into a proposed epoxy-xylachalasin derivative (RT = 5.15 min), which rearranges under acidic conditions into the more stable xylachalasin A (**22**, RT = 4.77 min).

To test this hypothesis, we performed two complementary experiments. First, we cocultured *Xylaria* sp. XSP14 and *Streptomyces* strain M56 on medium supplemented with 1-^13^C-l-phenylalanine, followed by targeted LC-MS^2^MS analysis of cytochalasin-enriched culture extract. Notably, the molecular ion features corresponding to the epoxy-xylachalasin A (**RT = 5.15 min**) were clearly detected, but with an increased molecular weight of 1 Da (RT = 5.15 min; *m*/*z* 493.226), indicating the incorporation of a single ^13^C isotope into the core structure (Figure 7, Figure S9).

This observation suggests, first, that 1-^13^C-l-phenylalanine is incorporated into the cytochalasin backbone, and second, that *Streptomyces* sp. M56 actively degrades cytochalasins produced by *Xylaria* sp. XSP14. To support these assumptions, *Streptomyces* strain M56 was then cultivated on PDA agar containing 0.5 mg/mL of a cytochalasin-enriched fraction, and additionally on PDA agar containing 1 mM 19,20-epoxycytochalasin Q (**6**). After several days of incubation, MS^2^-based analysis of culture extracts (Figures S10–S12) revealed that strain M56 catalyzed the time-dependent biotransformation of 19,20-epoxycytochalasin Q (**6**) into xylachalasin derivatives.

Upon close inspection of our analytical data, we inferred that 19,20-epoxycytochalasin Q (**6**) is likely degraded first to an elusive epoxy-xylachalasin derivative (RT = 5.15 min), which rearranges to xylachalasin A (**22**) (RT = 4.77 min) over time (Figure 7, Figure S11 and S12). As xylachalasin A is an acidic compound, it is plausible that an autocatalytic process facilitates epoxide ring opening, which could explain the difficulty in isolating the epoxy-xylachalasin derivative (RT = 5.15 min), even under acid-free purification conditions.

The enzymatic modification of cytochalasins by *Streptomyces* sp. M56 revealed that the phenylalanine-derived isoindole moiety of cytochalasins constitutes a chemically sensitive site within the scaffold. The formation of xylachalasin A appears to be enzyme-mediated and likely parallels oxidative degradation processes known from bacterial phenylalanine catabolism.^43–46^ Genomic analysis of *Streptomyces* sp. M56 further supported this hypothesis, revealing several putative dioxygenase, dehydrogenase, and aminotransferase genes consistent with the proposed biotransformation pathway,^39^ along with additional candidate enzymes that may participate in cytochalasin modification and are currently under investigation.

## The Cytochalasin Scaffold as a Precursor of Chemical Derivatization

In order to enrich our cytochalasin collection even further for subsequent biotesting, the mixtures of 19,20-epoxycytochalasin C (**3**) and 19,20-epoxycytochalasin Q (**6**) obtained through purifications were engaged in chemical reactions to semisynthesize new cytochalasins (Figure 8). The new 7-acetyl-19,20-epoxycytochalasin C (**23**) and *N*-acetyl-19,20-epoxycytochalasin Q (**24**) were obtained by acetylation and fully characterized by HRMS and 1D and 2D NMR.

**Figure 8:**
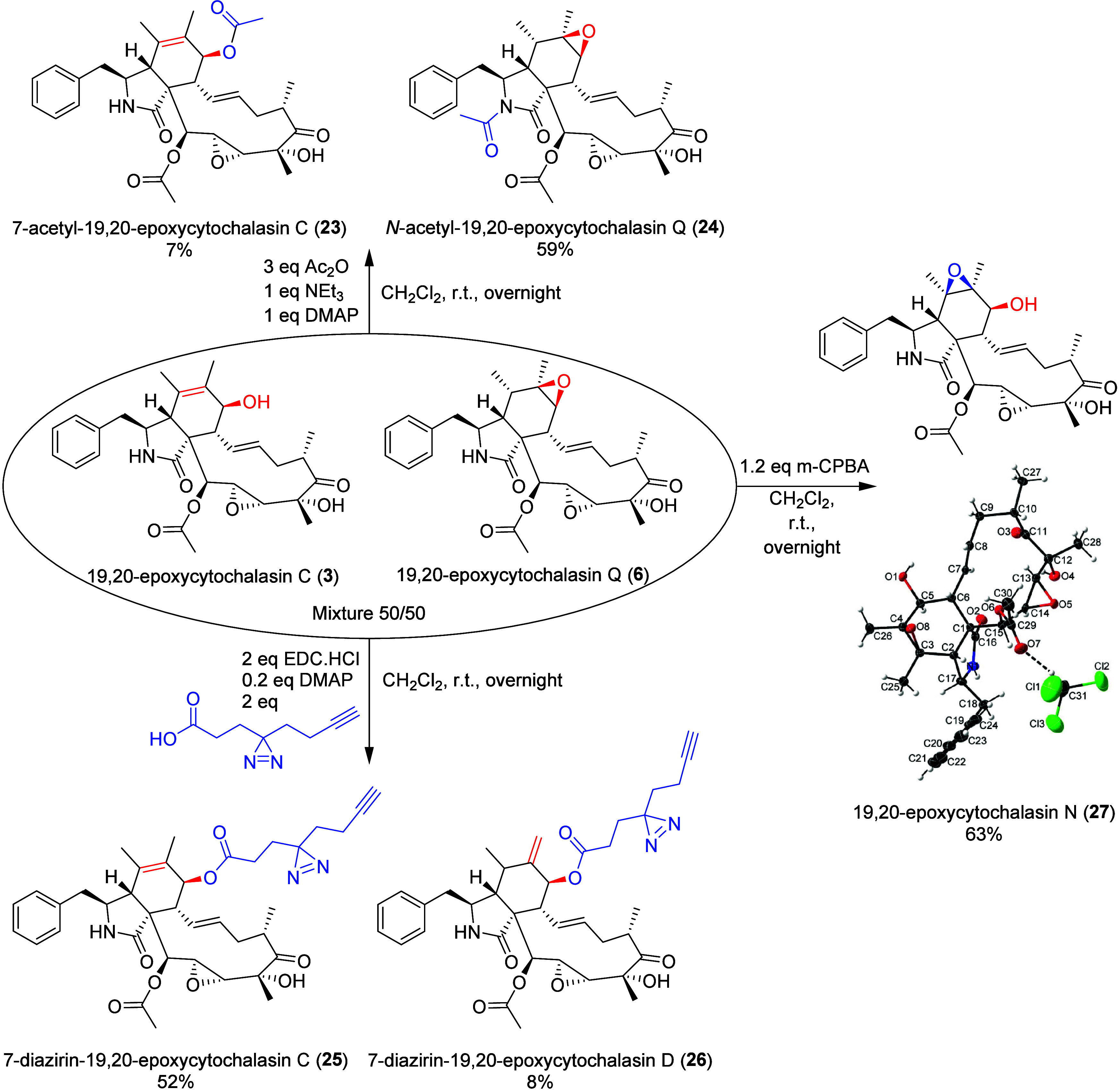
Semisynthesis scheme of modified derivatives using an enriched mixture of 19,20-epoxycytochalasin C (**3**) and 19,20-epoxycytochalasin C (**6**).

We next examined whether 3-(3-(but-3-yn-1-yl)-3H-diazirin-3-yl)­propanoic acid could be coupled to generate a chemical probe for labeling potential target proteins.^47^ Indeed, application of standard peptide coupling reagents successfully yielded two chemical probes, 7-diazirin-19,20-epoxycytochalasin C (**24**) and the rearranged congener **25**, which likely resulted from unintentional epoxide opening of compound **6**. For 19,20-epoxycytochalasin Q (**6**), the 6,7-epoxy ring opened during the coupling reaction via a proton shift yielding the allylic alcohol (C-7) in 7-diazirin-19,20-epoxycytochalasin D (**25**). Epoxidation of the mixture of 19,20-epoxycytochalasin C (**3**) and 19,20-epoxycytochalasin Q (**6**) using *m*-CPBA led only to the known 19,20-epoxycytochalasin N (**26**) for which X-ray structure was obtained to deduce the absolute configuration (Figure 8).

## Biological Evaluation

Actin is the primary and most extensively studied target of cytochalasans,^48^ with some derivatives suspected to bind covalently or noncovalently to the barbed (plus) ends of actin filaments (F-actin), thereby preventing further monomer addition.^23,33^ Because actin is essential for numerous eukaryotic processes, cytochalasans can disrupt cellular functions at multiple levels suggesting a broader, yet understudied, spectrum of activities. Even after decades of research, the availability of comprehensive biological datasets across diverse structural variants remain scarce and often inconsistent, largely due to the use of different cell line models and their varying sensitivities.^48^ To address these limitations, we aimed to provide a comprehensive assessment of the bioactivity and stability of the 26 derivatives of this study (Supporting Table S22). Given that bacteria possess distantly related actin homologues, we tested the natural isolates for antibacterial activity against *Staphylococcus aureus* Newman and the sefflux-compromised *E. coli* ΔacrB strain. However, no antibacterial effects were observed for any of the tested compounds. We then subjected all compounds to a standardized cell toxicity study using CHO-K1 (Chinese hamster ovary) and A549 (human lung adenocarcinoma) cell lines (Supporting Table S22). Overall, strong structure-dependent cytotoxicity was observed, showing often a log-fold lower IC_50_ value for the lung adenocarcinoma cell line A549 with exception of compound **23** and xylachalasin A (**22**). While most unmodified epoxy-cytochalasins (e.g., **3** and **5**) exhibited activity levels that were comparable to the positive control (doxorubicin), O-acylated *meta*- and *ortho*- halogen-substituted congeners (**11**, **15**, **17**) exhibited lower nanomolar activities against A549 (Table 1 and Supporting Table S22). Moreover, the potent activities of the halogenated derivatives are comparable to, or even exceed, those reported for cytochalasin B and related congeners,^20–24,41,48^ suggesting that meta-halogenation at the phenylalanine moiety does not diminish, and may even enhance, cytotoxic potency.^20–24,41,48^ Remarkably, the bacterial-modified derivative **10**, along with the chemically modified analogs **23** and **25**, showed a pronounced reduced activity against A549 cell line compared to the meta-substituted congeners **17** and **18**. The attenuated cytotoxicity of xylachalasin A (**22**) underscores the critical role of the aromatic ring for cytotoxic function.^41,48^ However, establishing direct correlations between specific structural features remained challenging due to the high number of stereocenters and structural variations. We then evaluated the half-lives of each compound using mouse liver microsome assays to assess their metabolic stability and rate of enzymatic degradation. Several derivatives showed prolonged half-lives (t_1_/_2_), consistent with enhanced resistance to hepatic metabolism. The shorter t_1_/_2_ of **23** compared with **3** indicates that the 7-acetyl moiety may confer metabolic instability. A similar effect of the 21-acetyl group was observed for **6** versus **7**, though this trend was absent in the halogenated pairs **18/19** and **14/15** (Supporting Table S22). Notably, the C-18- desoxy-derivative **5** exhibited reduced stability relative to its oxidized analogue **3**. The calculated the intrinsic clearance (CL_int_, μL/min·mg) from the microsomal metabolism data, representing the efficiency of enzymes in metabolizing the compound per milligram of microsomal protein per minute. While again, acetylation patterns significantly affected both potency and metabolic stability, the favorable plasma stability profiles observed for most of our derivatives represent a significant advantage, as metabolic stability remains a key challenge in developing therapeutics.^23^ A similar effect was observed for mouse plasma *t*_1/2_ (min), which reflects how long the compound persists in the bloodstream by integrating the effects of metabolism, distribution, and other clearance mechanisms.

**Table 1:**
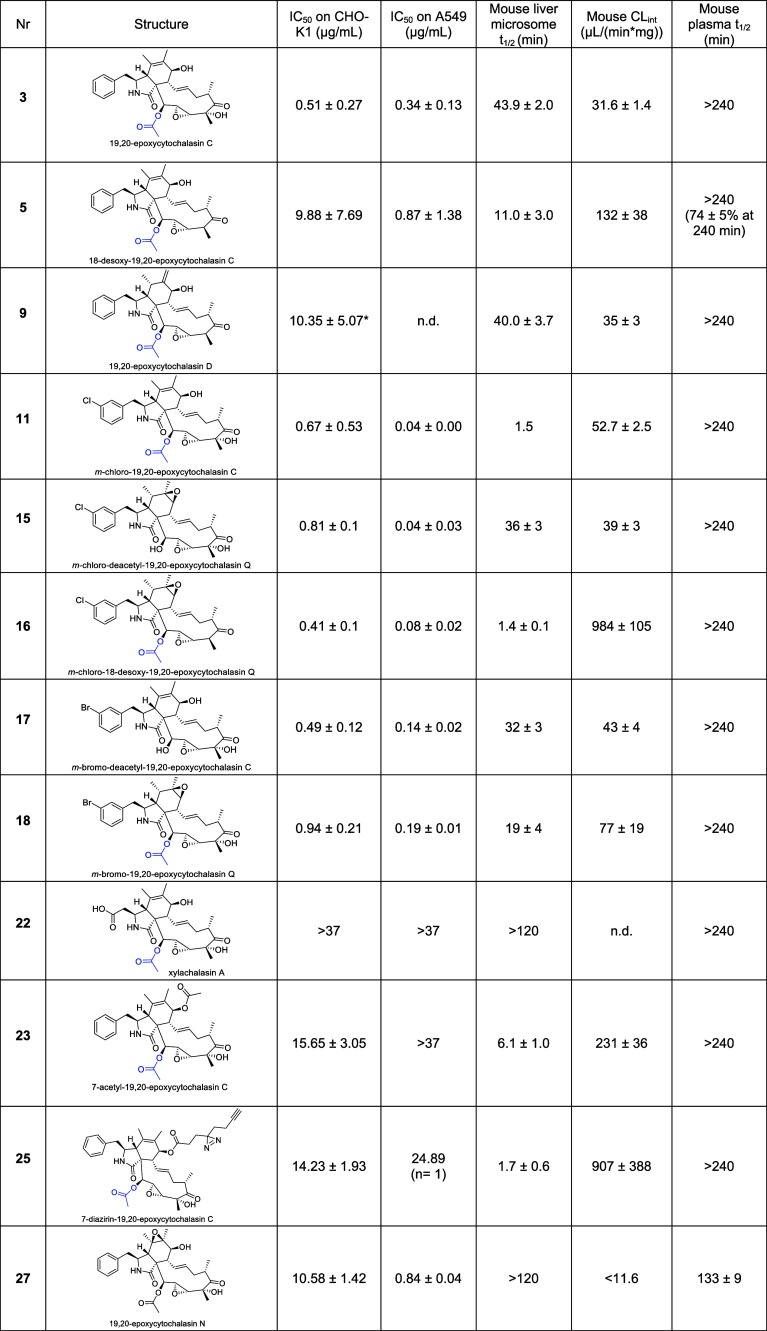
Selected Examples of Cellular Toxicity Studies and Relevant Stability Assays in Mouse Liver Microsomes^a^

a Experiments were performed in at least two independent measurements (data for an extensive set of examples can be found in Supporting Table S22); doxorubicin: 0.30 ± 0.14 μg/mL (positive control for cell line A548). Mouse liver microsome *t*_1/2_ and CL_int_ represent the in vitro metabolic stability of the compounds, reflecting the half-life and intrinsic clearance by liver enzymes, respectively. Mouse plasma *t*_1/2_ indicates the persistence of the compounds in plasma over time.

## Conclusion

Overall, our findings underscore the profound influence of interspecies interactions on secondary metabolite biosynthesis and highlight the value of coculture strategies for discovering novel bioactive compounds. Through genome sequencing and biosynthetic analysis of the fungal isolate, we identified the BGC variant *ecy* responsible for cytochalasin production in *Xylaria* sp. XSP14 with high overall similarity to BGC clusters spread across the *Xylaria* genus. We explored the intrinsic promiscuity of the underlying NRPS by precursor-directed diversification strategy and uncovered a surprising tolerance toward accepting unnatural *ortho*- and *meta*- substituted phenylalanine derivatives, which ultimately led to previously unreported chlorinated and brominated variants. Our results provide a foundation for future targeted investigations into the unexplored substrate promiscuity of the A domain encoded within cytochalasin BGCs, and potential across the diverse cytochalasan family. The work will thereby catalyze research into the rational bioengineering of these complex hybrid pathways. Our fungal–bacterial cocultivation experiments further demonstrated that *Streptomyces* sp. M56 can oxidatively modify fungal cytochalasins leading to the generation of highly altered structural scaffolds. Structural modifications were also introduced through semisynthetic approaches, including the synthesis of chemical probes **25** and **26**, which represent promising scaffolds for future investigations into the precise and still underexplored binding mode and site, as well as potential nonactin targets. Bioassays of 26 cytochalasan derivatives demonstrated structure-dependent potent cytotoxicity along with favorable plasma stability profiles. Intriguingly, the bacterial-modified variant xylachalasin A exhibited significantly reduced cytotoxicity indicating that bacteria are capable of altering the profile of cytotoxic natural products. Our study encourages future research into the ecological relevance of such catabolic-like transformations within natural systems. Our findings suggest that bacteria can attenuate the effects of fungal bioactive compounds, thereby likely improving their capacity to coexist and even compete within complex, densely populated microbial communities.

## Supplementary Material







## Data Availability

LC-HRMS2 data of the molecular network analyses has been submitted to MassIVE server: MSV000098573-MSV000098577, and MSV000098580. NMR data and supplementary data has also been deposited at Zenodo (10.5281/zenodo.16784814). Compounds of this manuscript have been tested and deposited with the following HIPS IDs C1945–C1976. Raw sequencing data have been submitted to the NCBI database under the BioProject accession number PRJNA1210561. The assembled genome of *Xylaria* sp. XSP14 has been deposited at DDBJ/ENA/GenBank under the accession JBPOEM000000000. The version described in this paper is version JBPOEM010000000.

## Notes

Supplementary spectral data (PDF)
Supplementary Information with additional details to experimental and analytical data; Figures S1–S12, Tables S1–S21 (PDF)
Table S22: Additional data (XLSX)
