# Supplementary material for: Microbial Cross-Talk: Unlocking the Cytochalasin Diversity from a Termite-Associated Xylaria
Source: JACS Au. 2025 Dec 22;6(1):179–92. doi: 10.1021/jacsau.5c01093 (PMC12848680; doi:10.1021/jacsau.5c01093)
Supplement: Supplementary file 1 [file au5c01093_si_001.pdf]

Supplementary Spectral Data

Microbial Cross-Talk: Unlocking the Cytochalasin Diversity from a Termite-Associated *Xylaria*

Marie Dayras,[1] Yaming Liu,[1] Rebecca Kochems,[1] Martinus de Kruijff,[1] Sven Balluff,[1] Sari Rasheed,[1] Andreas M. Kany,[1,2] Jennifer Herrmann,[1] Sebastian Götze,[1] Bernd Morgenstern,[3] N'Golo A. Koné,[4] Michael Poulsen,[5] Rolf Müller,[1,2,6] Christine Beemelmanns[1,6*]

Affiliation

1) Helmholtz Institute for Pharmaceutical Research Saarland (HIPS), Helmholtz Centre for Infection Research (HZI), Campus E8.1, 66123 Saarbrücken, Germany

2) Deutsches Zentrum für Infektionsforschung (DZIF) e.V., Braunschweig 38124, Germany

3) Inorganic Solid-State Chemistry, Saarland University, Campus Building C4 1, 66123 Saarbrücken, Germany

4) Unité de Formation et de Recherche en Sciences de la Nature (UFR-SN), Université Nangui Abrogoua, Station de Recherche en Ecologie du Parc National de la Comoé, 27 BP 847 Abidjan 27, Côte d'Ivoire

5) Section for Ecology and Evolution, Department of Biology, University of Copenhagen, 2100 Copenhagen East, Denmark

6) Pharma Science Hub (PSH), Saarland University, 66123 Saarbrücken, Germany.

*corresponding authors:

christine.beemelmanns@helmholtz-hips.de

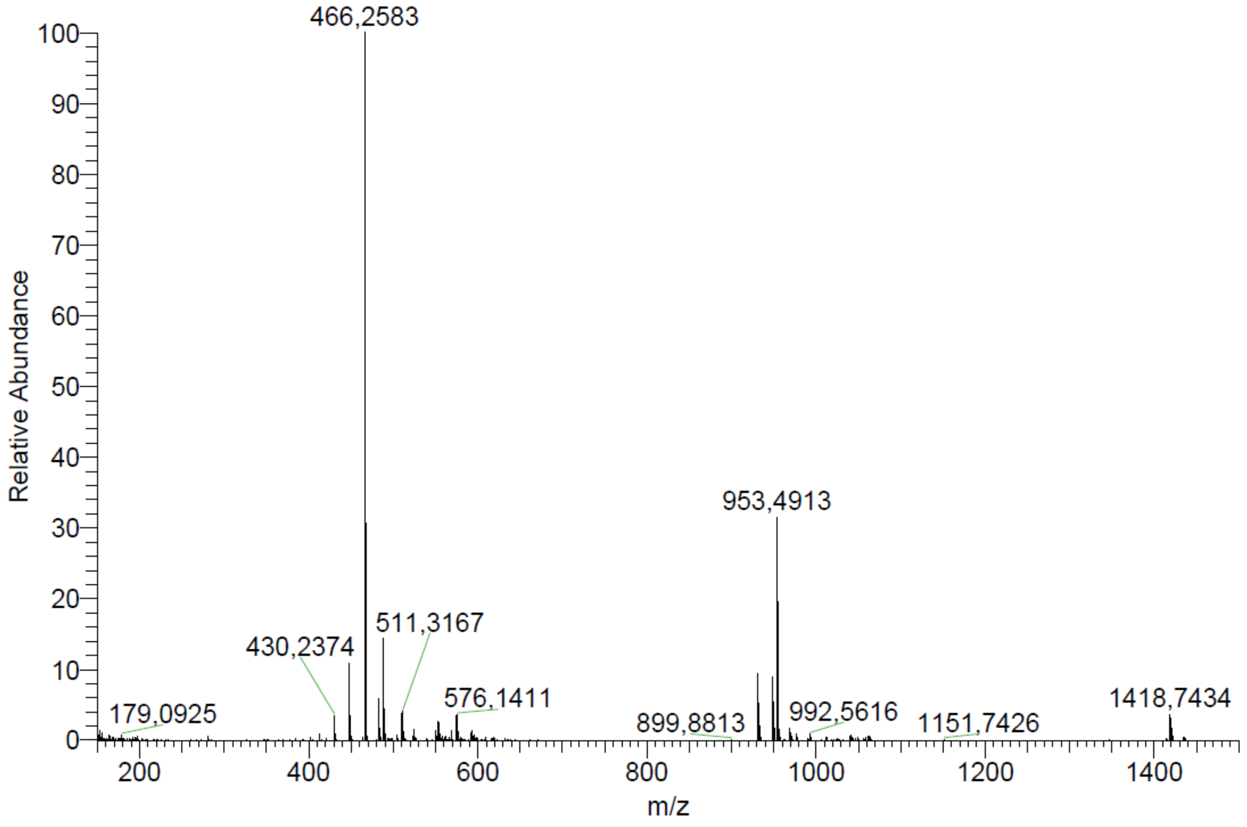

Figure SD1. ESI-HRMS spectrum of deacetyl-18-desoxy-19,20-epoxycytochalasin Q (**1**)

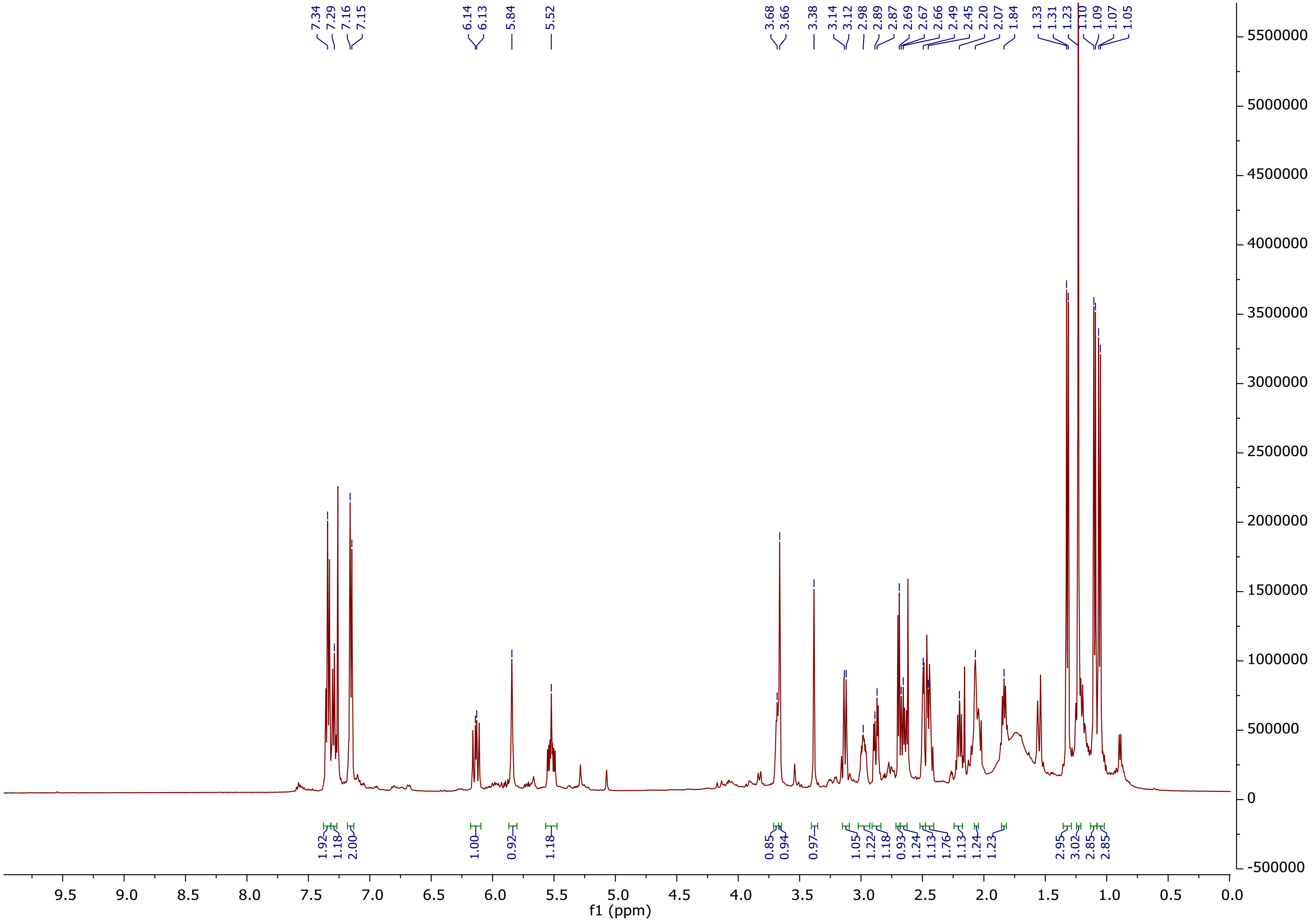

Figure SD2. ^{1}H NMR spectrum of deacetyl-18-desoxy-19,20-epoxycytochalasin Q (**1**) (500 MHz, $CDCl_3$)

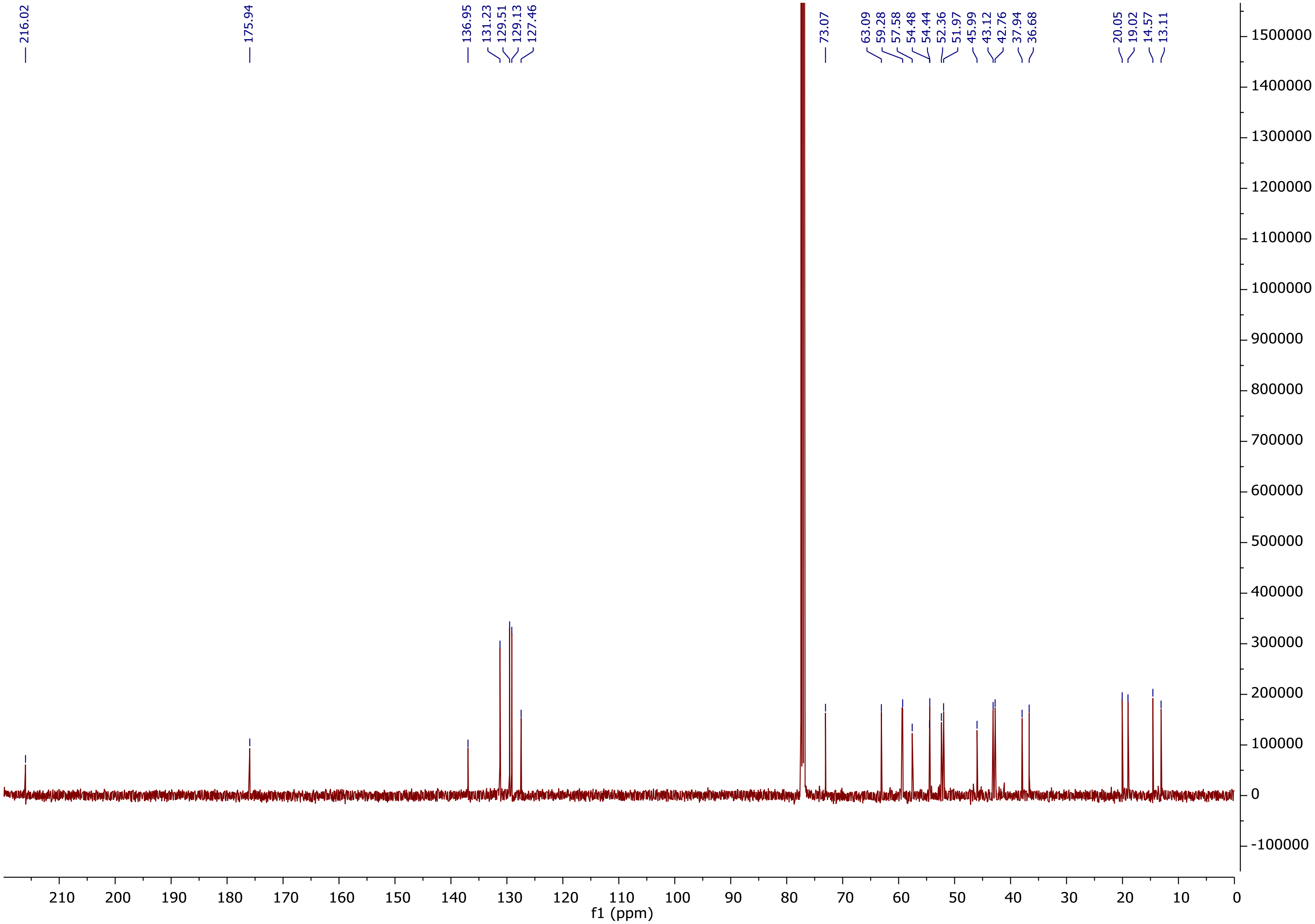

Figure SD3. ^{13}C NMR spectrum of deacetyl-18-desoxy-19,20-epoxycytochalasin Q (**1**) (125 MHz, $CDCl_3$)

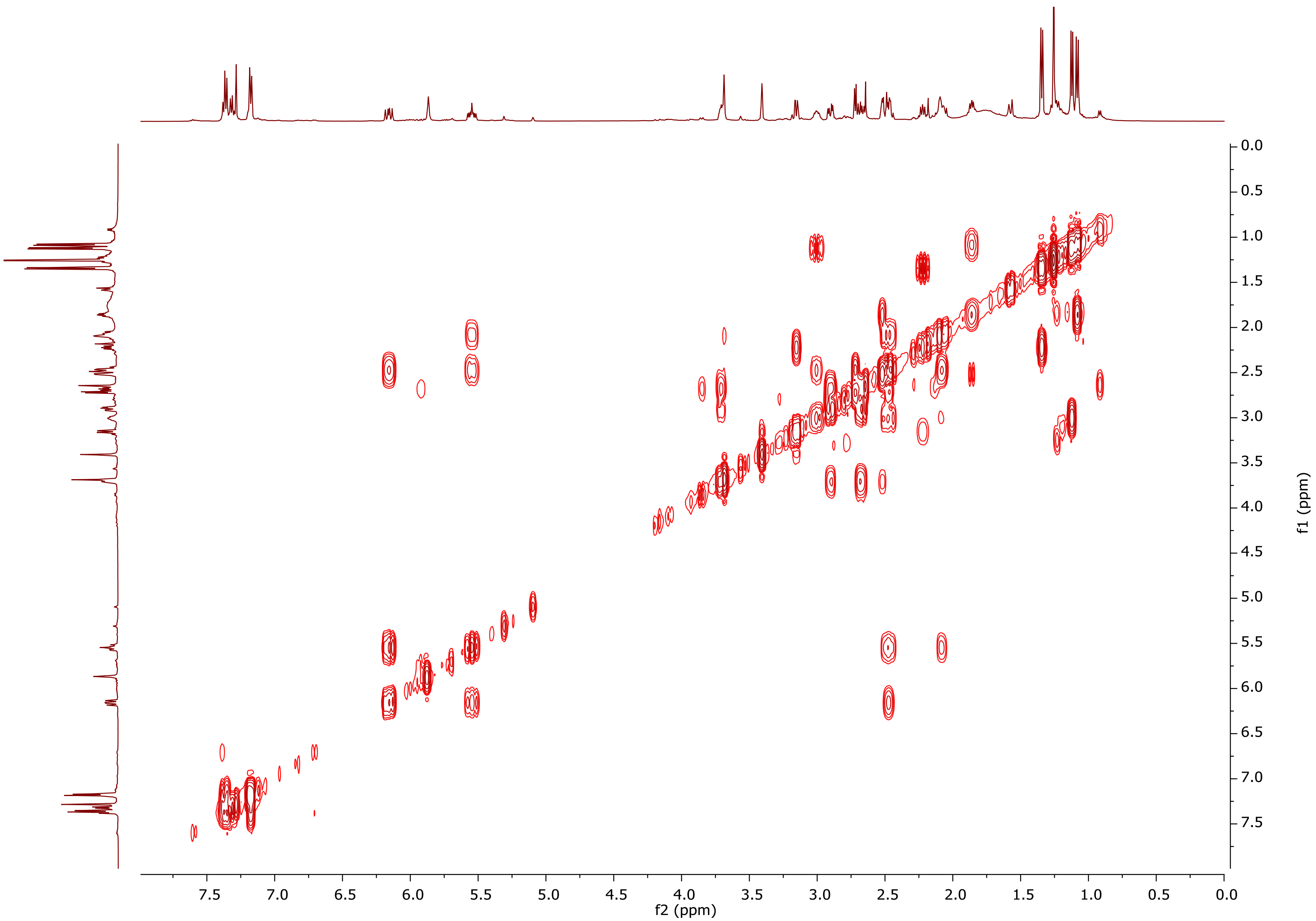

Figure SD4. ^{1}H-^{1}H COSY NMR spectrum of deacetyl-18-desoxy-19,20-epoxycytochalasin Q (**1**) (500/500 MHz, $CDCl_3$)

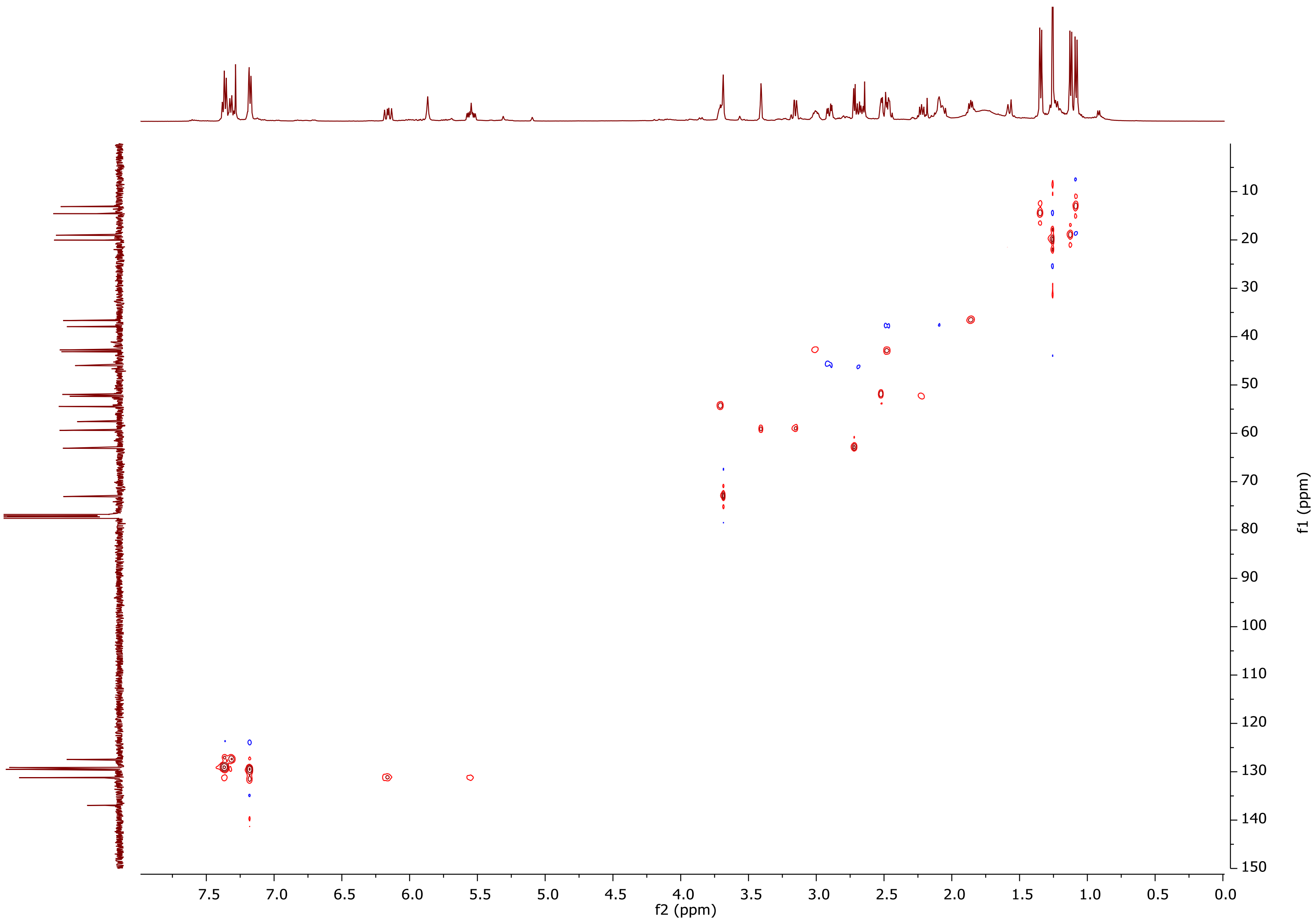

Figure SD5. ^{1}H-^{13}C HSQC NMR spectrum of deacetyl-18-desoxy-19,20-epoxycytochalasin Q (**1**) (500/125 MHz, $CDCl_3$)

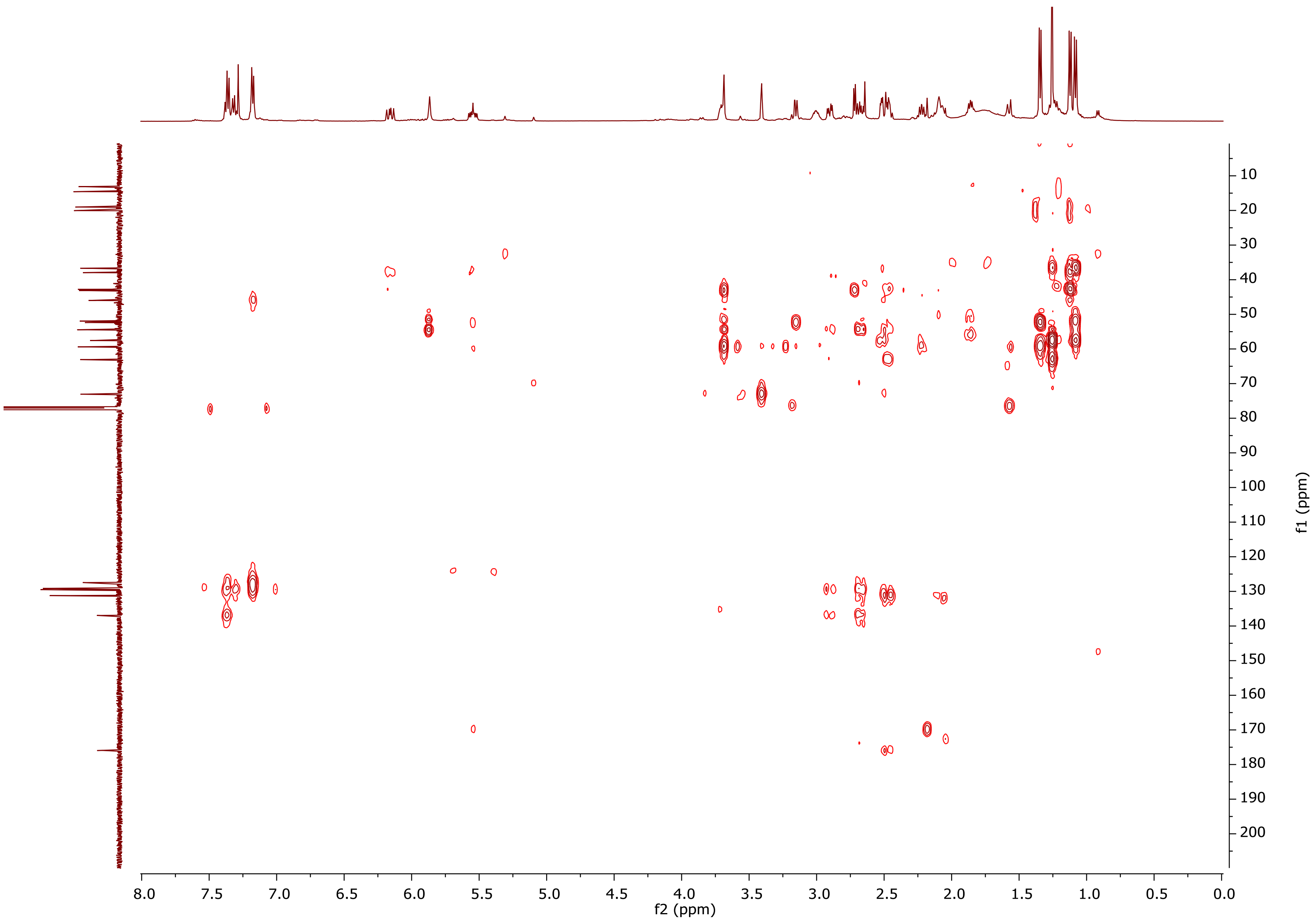

Figure SD6. ^{1}H-^{13}C HMBC NMR spectrum of deacetyl-18-desoxy-19,20-epoxycytochalasin Q (**1**) (500/125 MHz, $CDCl_3$)

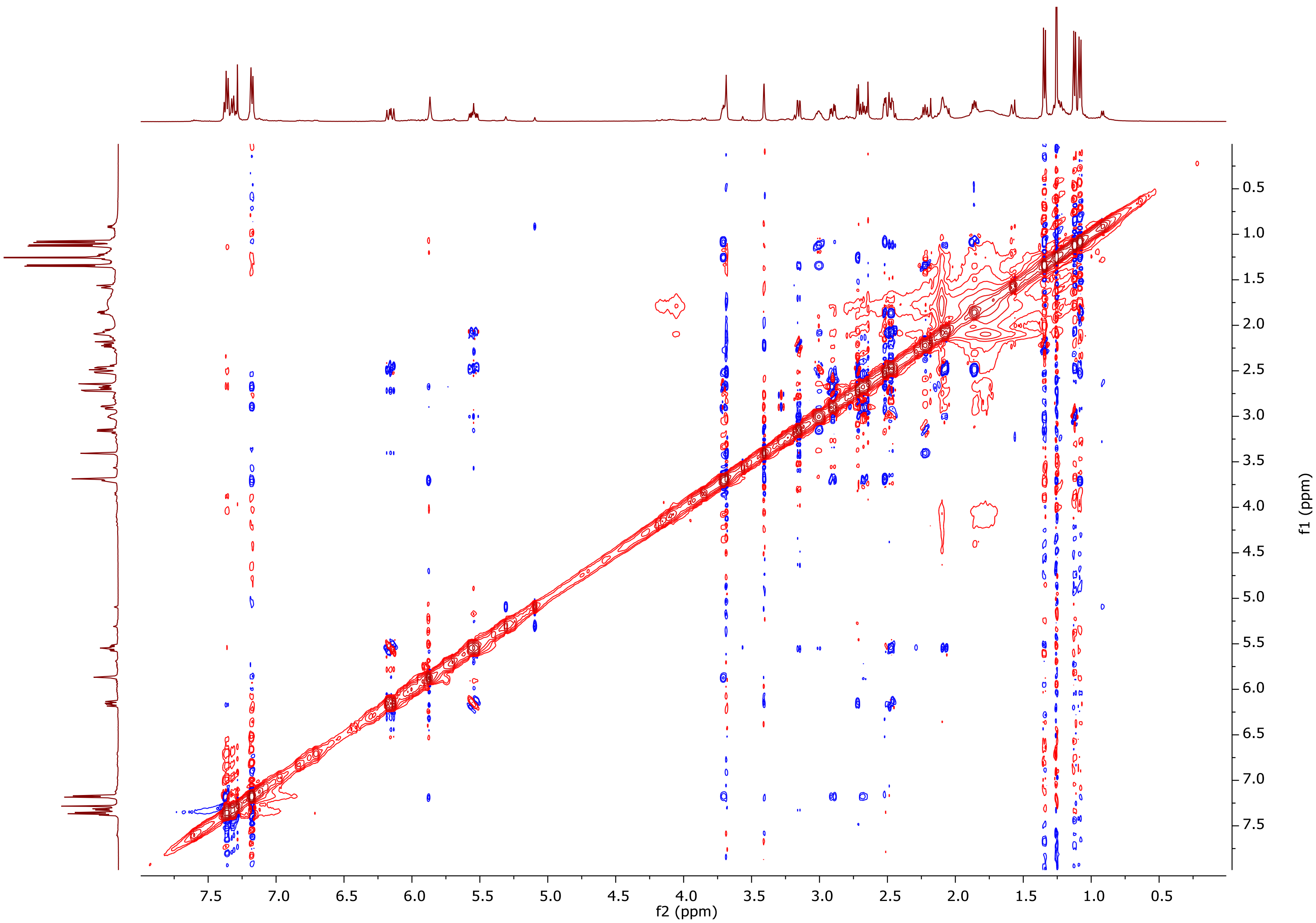

Figure SD7. ^{1}H-^{1}H NOESY NMR spectrum of deacetyl-18-desoxy-19,20-epoxycytochalasin Q (**1**) (500/500 MHz, $CDCl_3$)

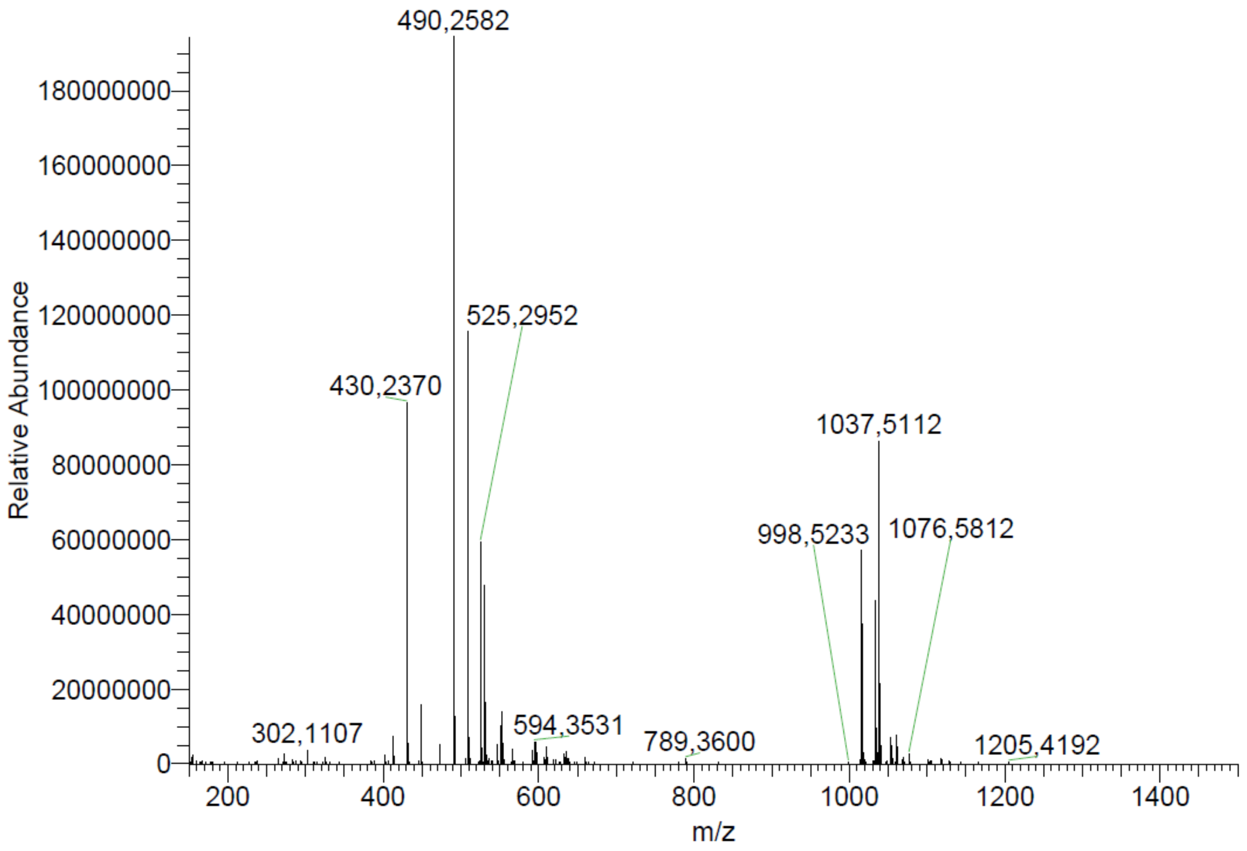

Figure SD8. ESI-HRMS spectrum of cytochalasin C (**2**)

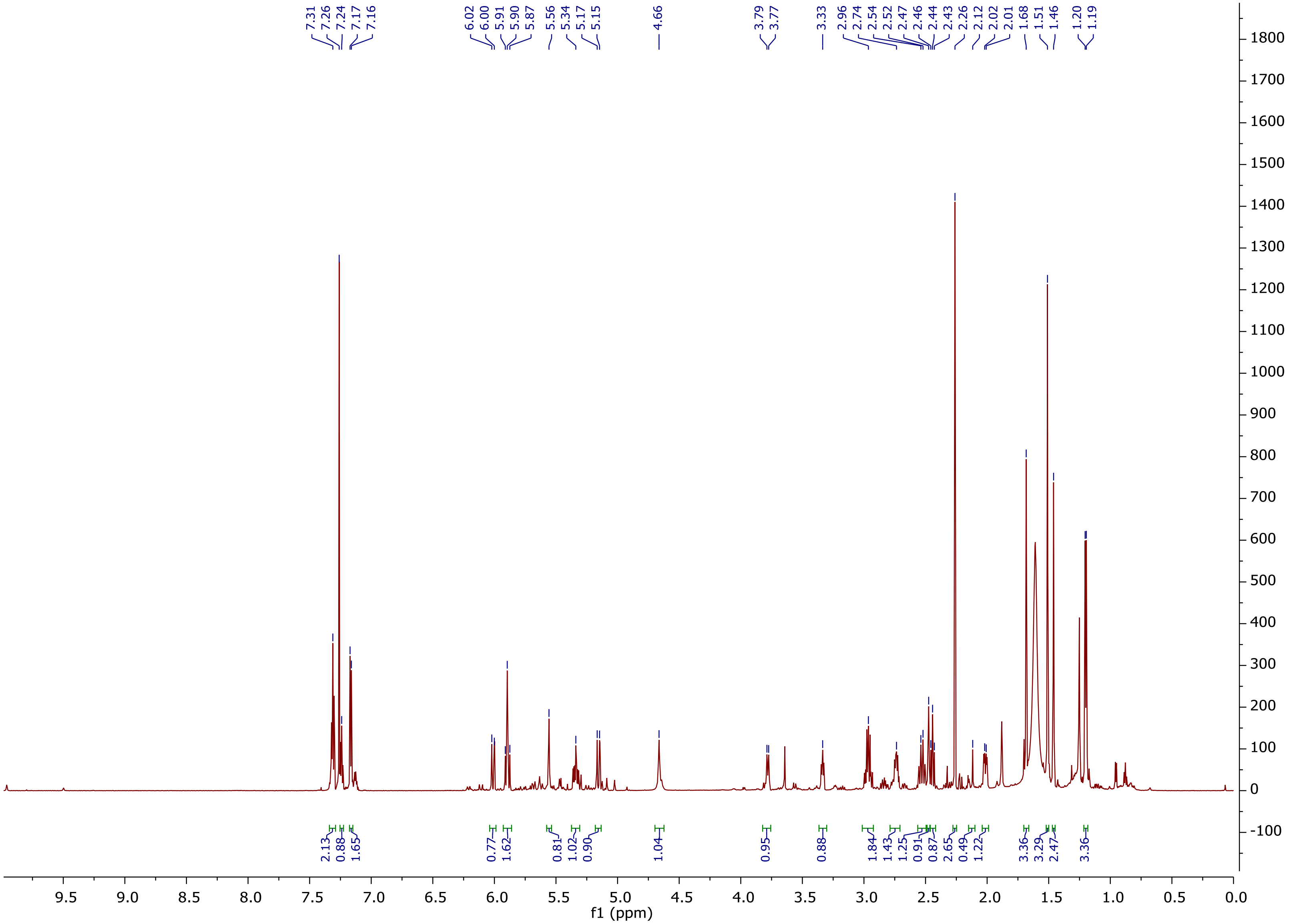

Figure SD9. ^{1}H NMR spectrum of cytochalasin C (**2**) (700 MHz, $CDCl_3$)

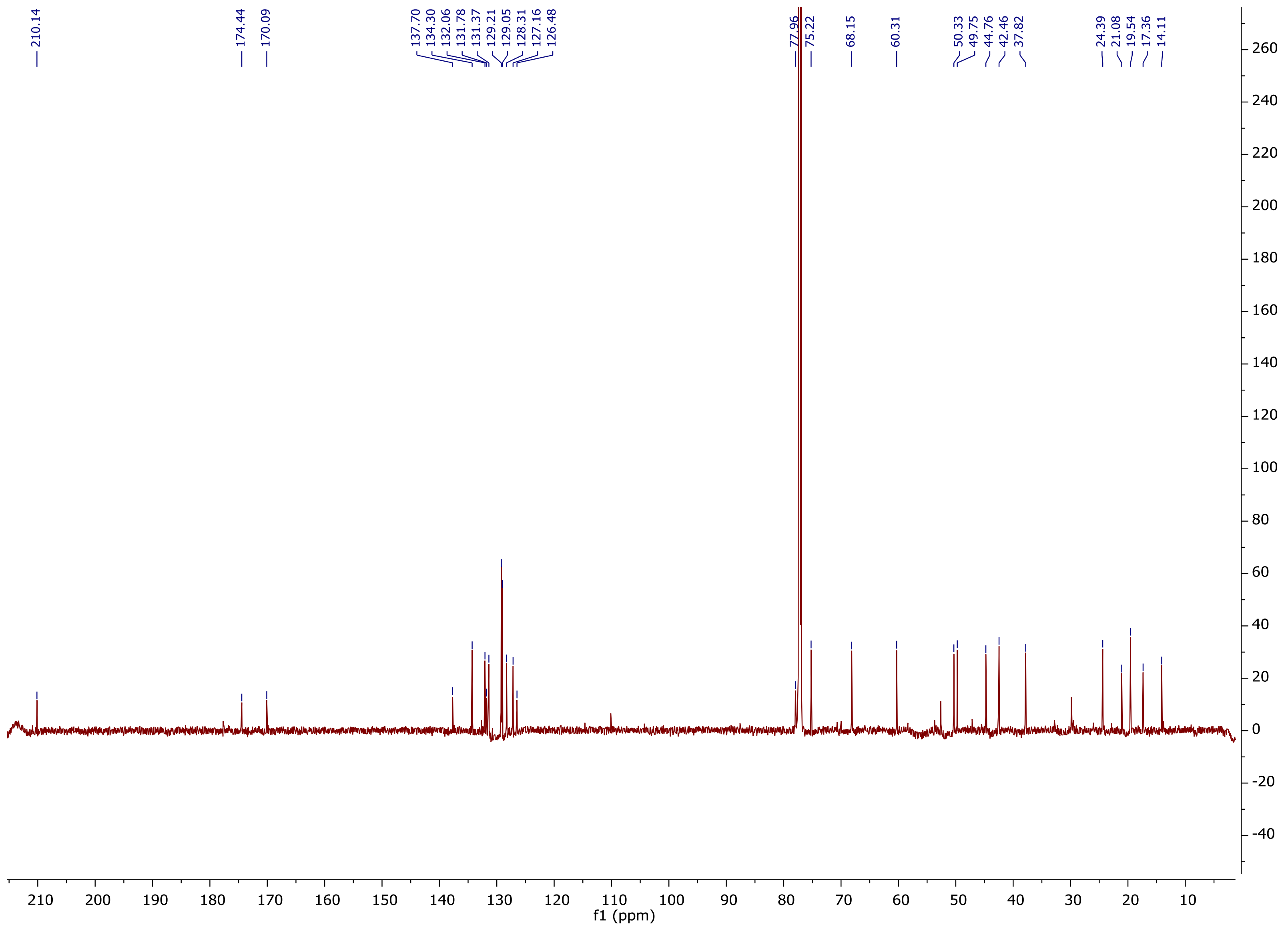

Figure SD10. ^{13}C NMR spectrum of cytochalasin C (**2**) (175 MHz, $CDCl_3$)

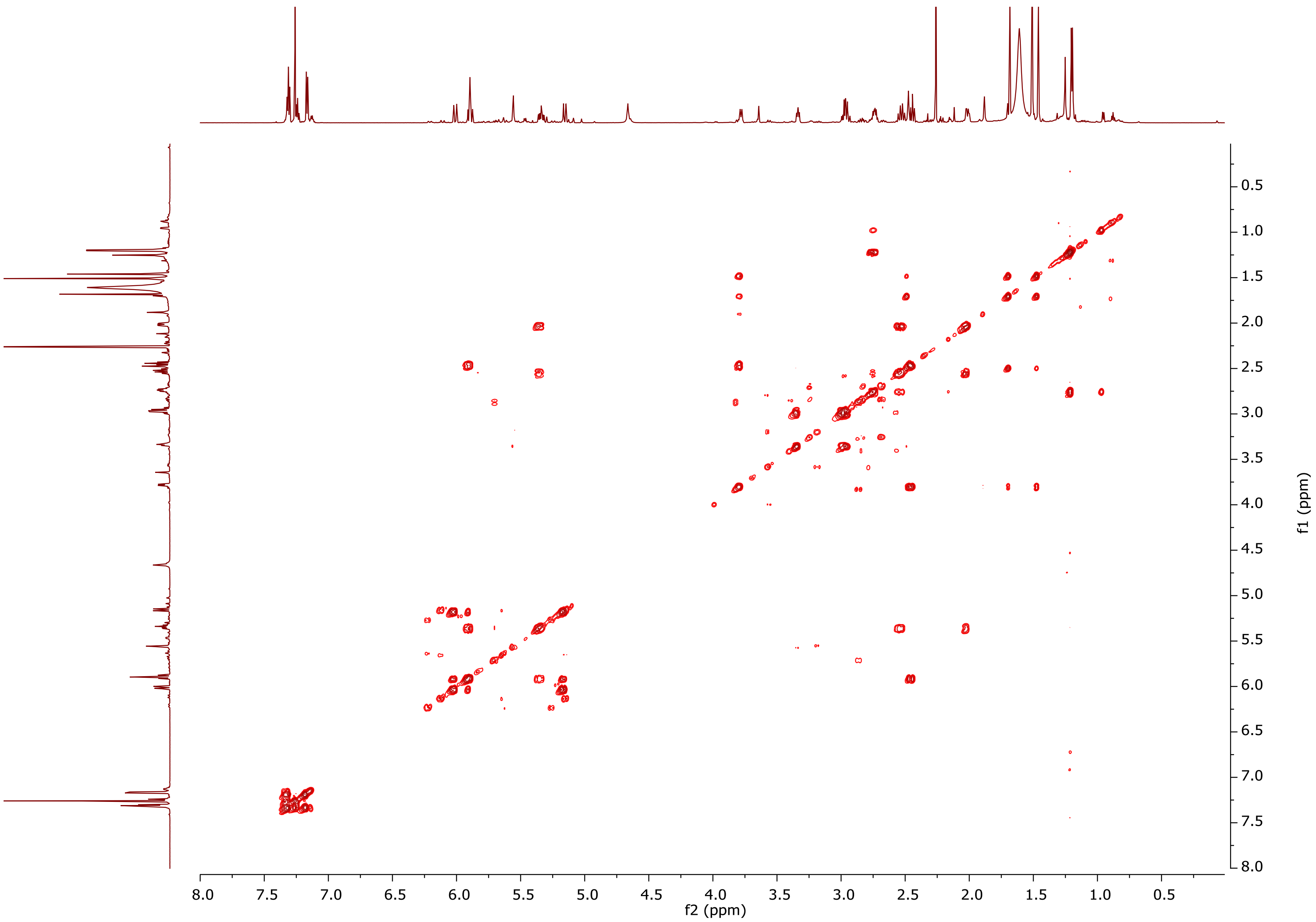

Figure SD11. ^1H-^1H COSY NMR spectrum of cytochalasin C (**2**) (700/700 MHz, $CDCl_3$)

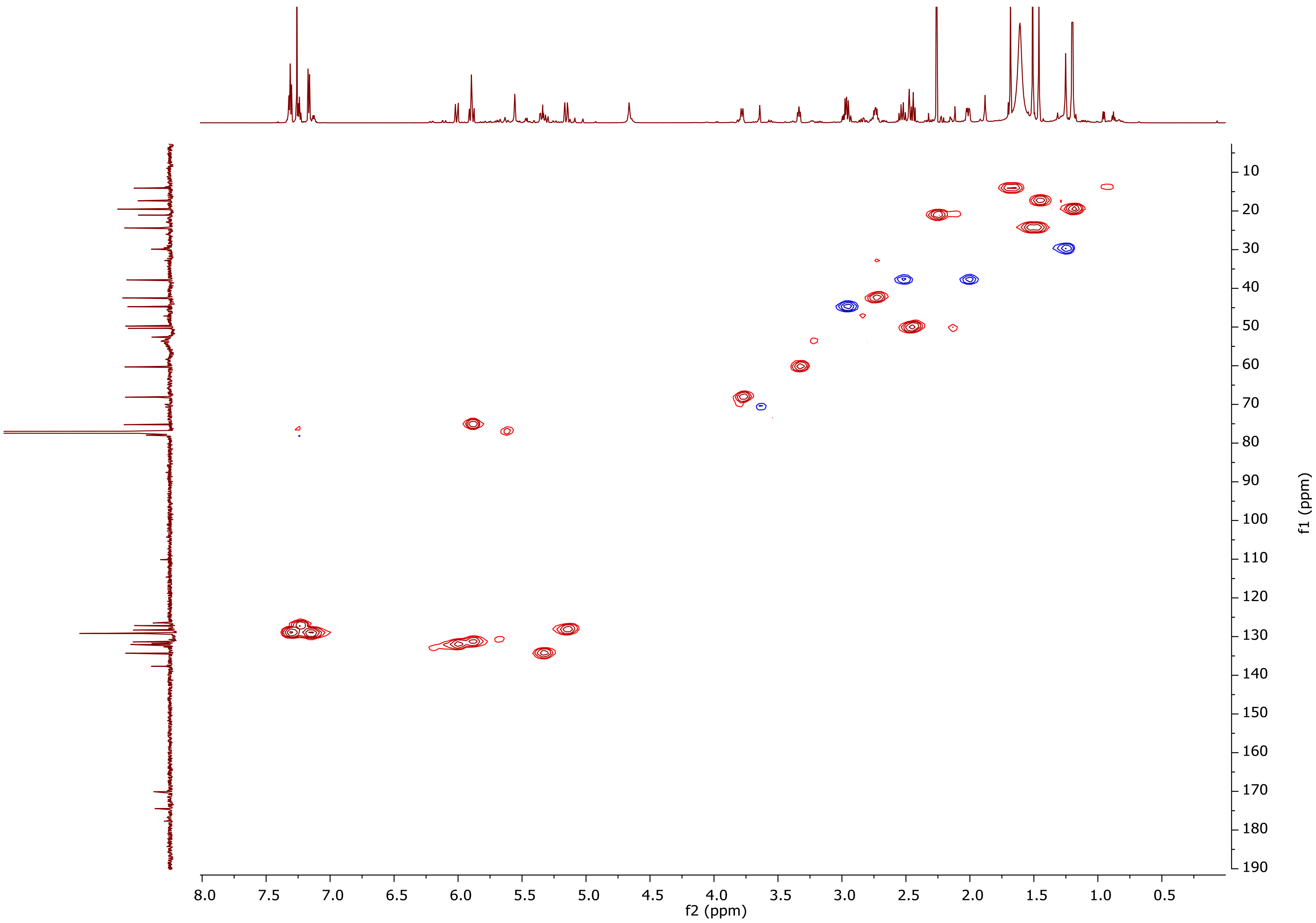

Figure SD12. ^{1}H-^{13}C HSQC NMR spectrum of cytochalasin C (**2**) (700/175 MHz, $CDCl_3$)

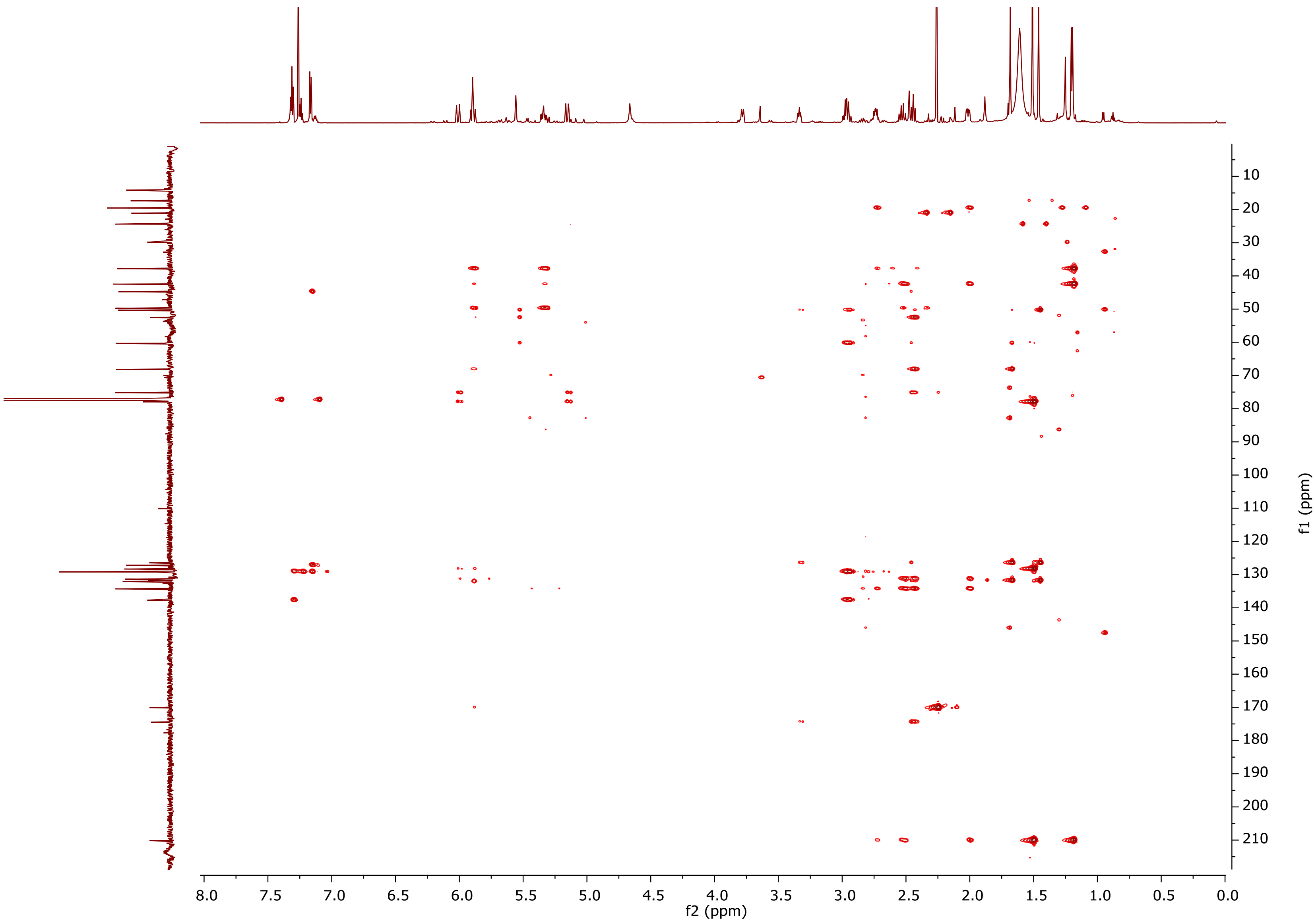

Figure SD13. ^{1}H-^{13}C HMBC NMR spectrum of cytochalasin C (**2**) (700/175 MHz, $CDCl_3$)

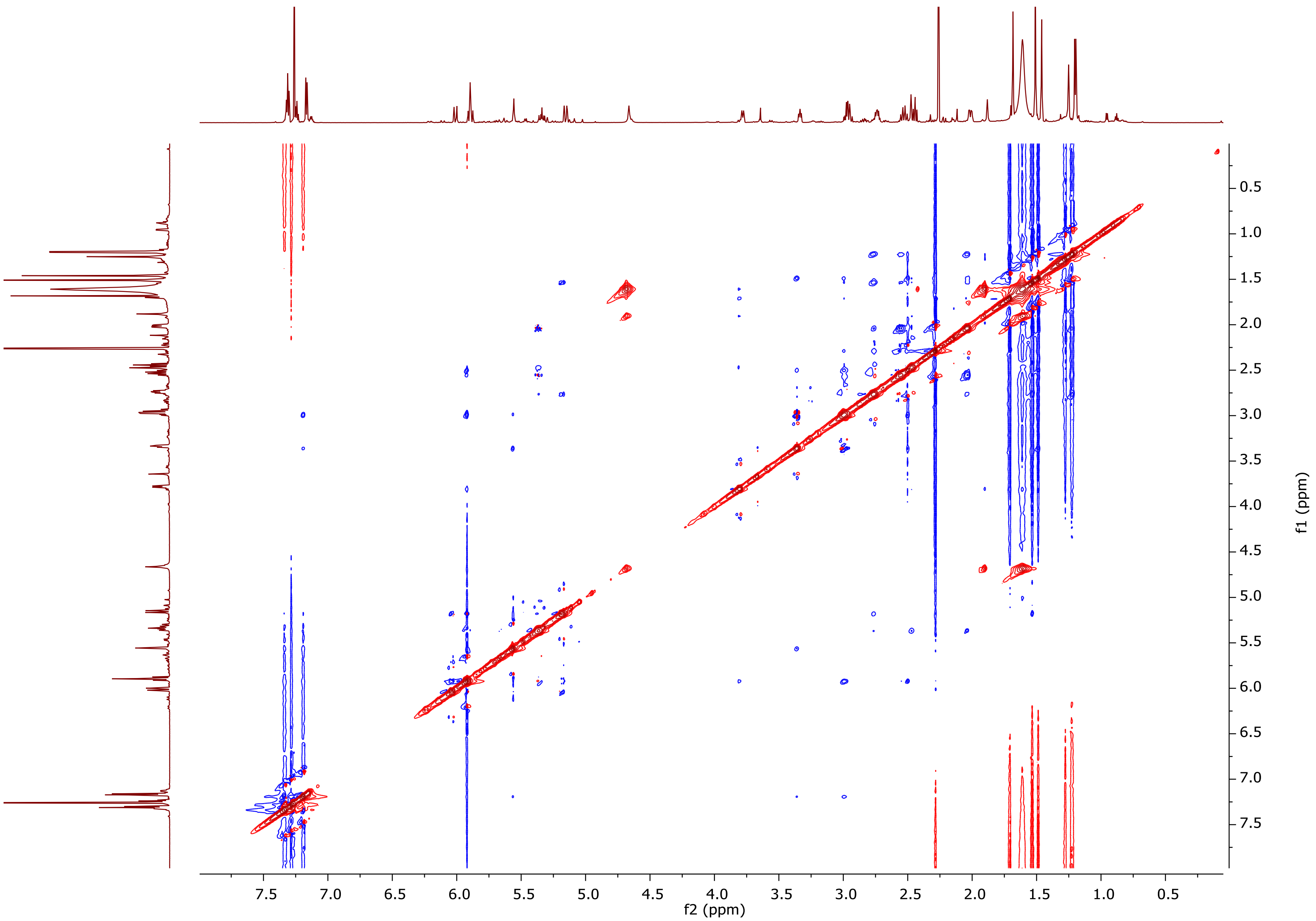

Figure SD14. ^1H-^1H NOESY NMR spectrum of cytochalasin C (**2**) (700/700 MHz, $CDCl_3$)

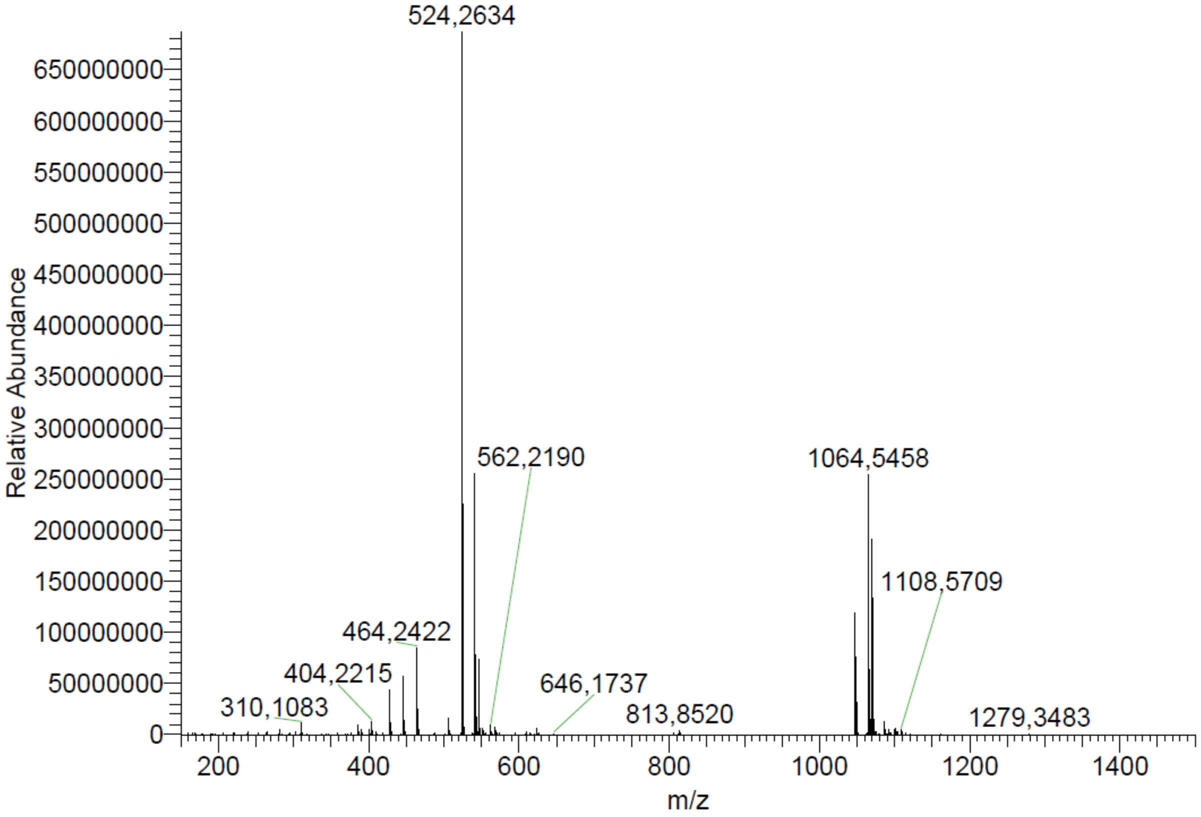

Figure SD15. ESI-HRMS spectrum of 19,20-epoxycytochalasin C (**3**)

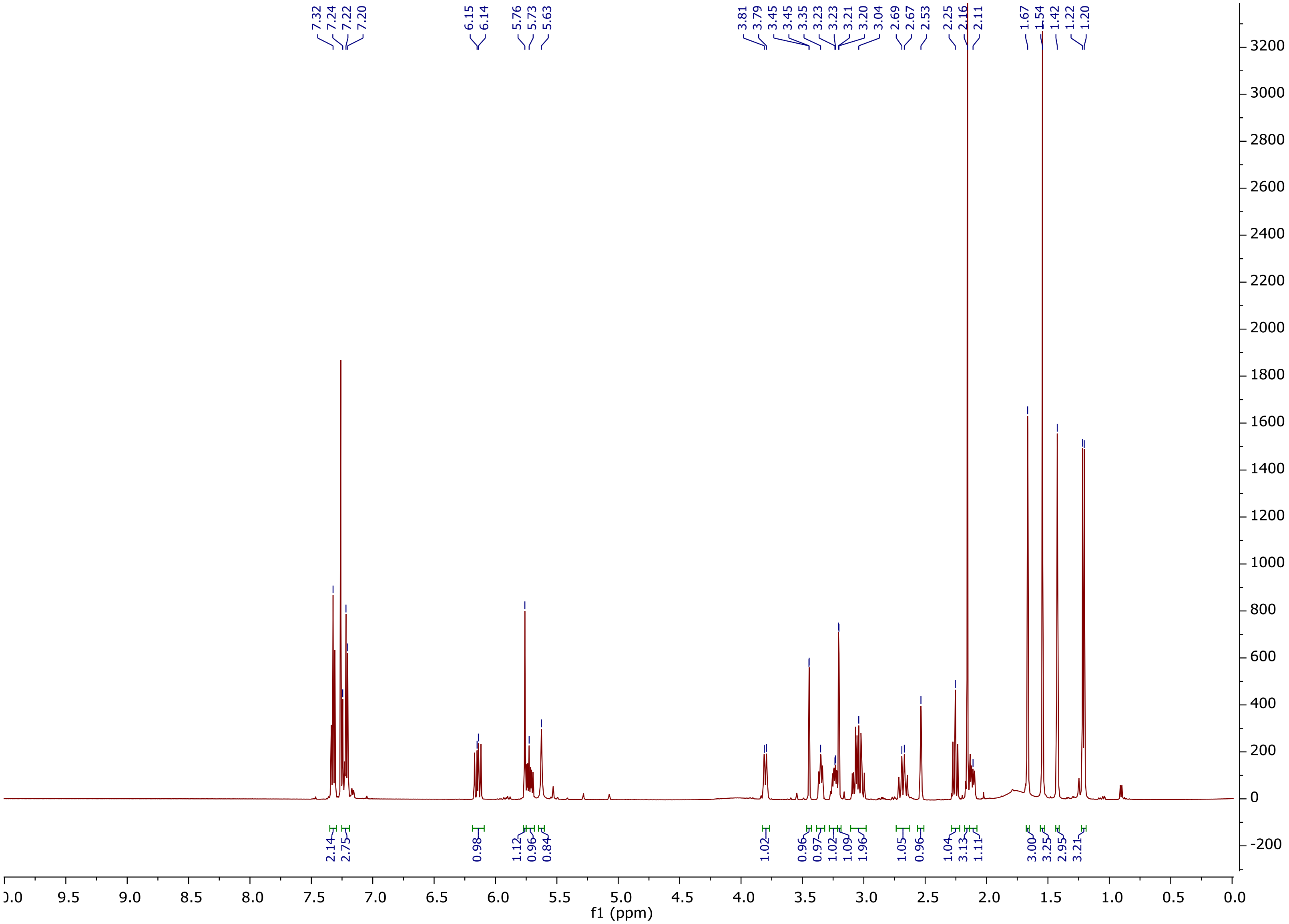

Figure SD16. ^{1}H NMR spectrum of 19,20-epoxycytochalasin C (**3**) (500 MHz, $CDCl_3$)

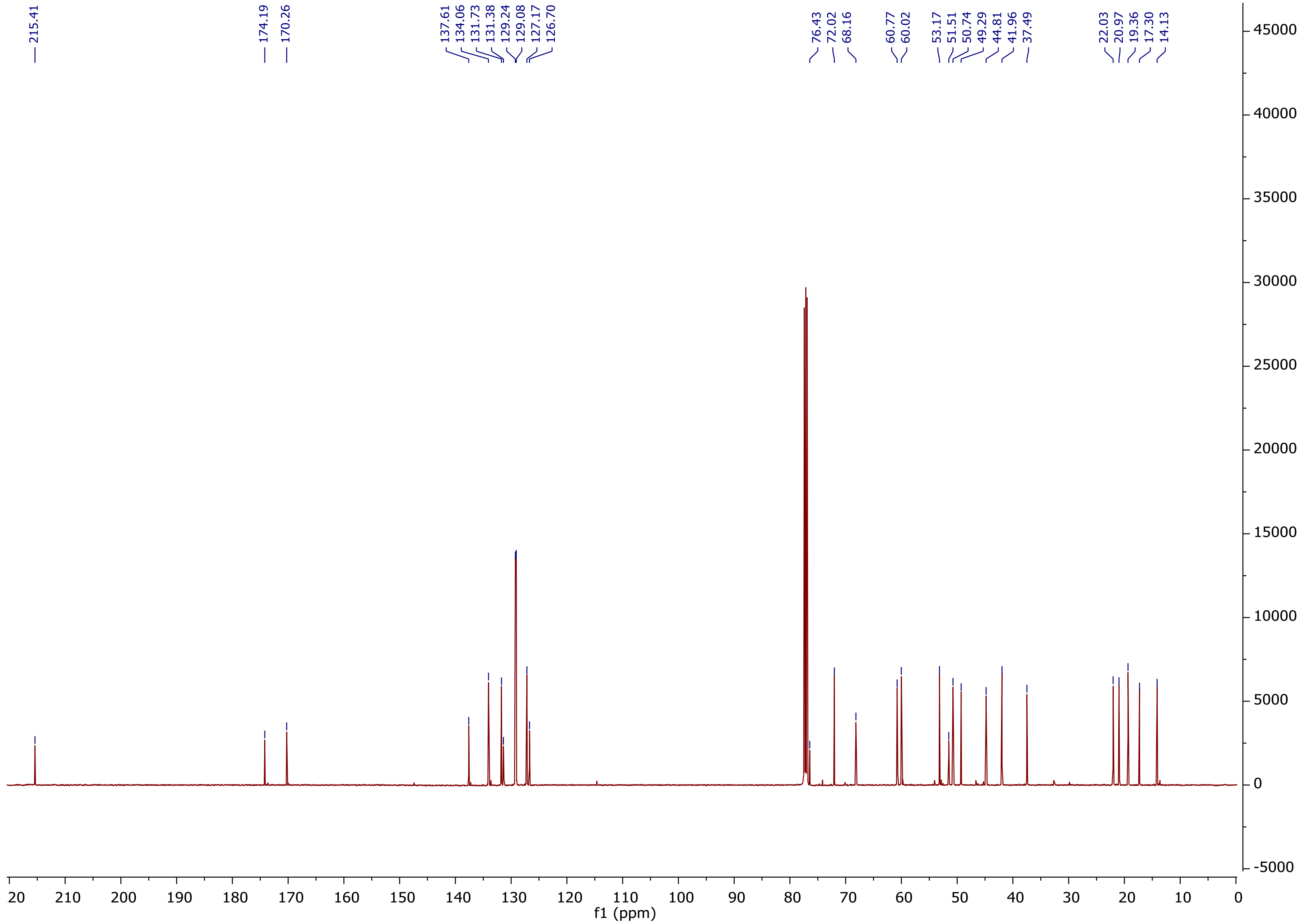

Figure SD17. ^{13}C NMR spectrum of 19,20-epoxycytochalasin C (**3**) (125 MHz, $CDCl_3$)

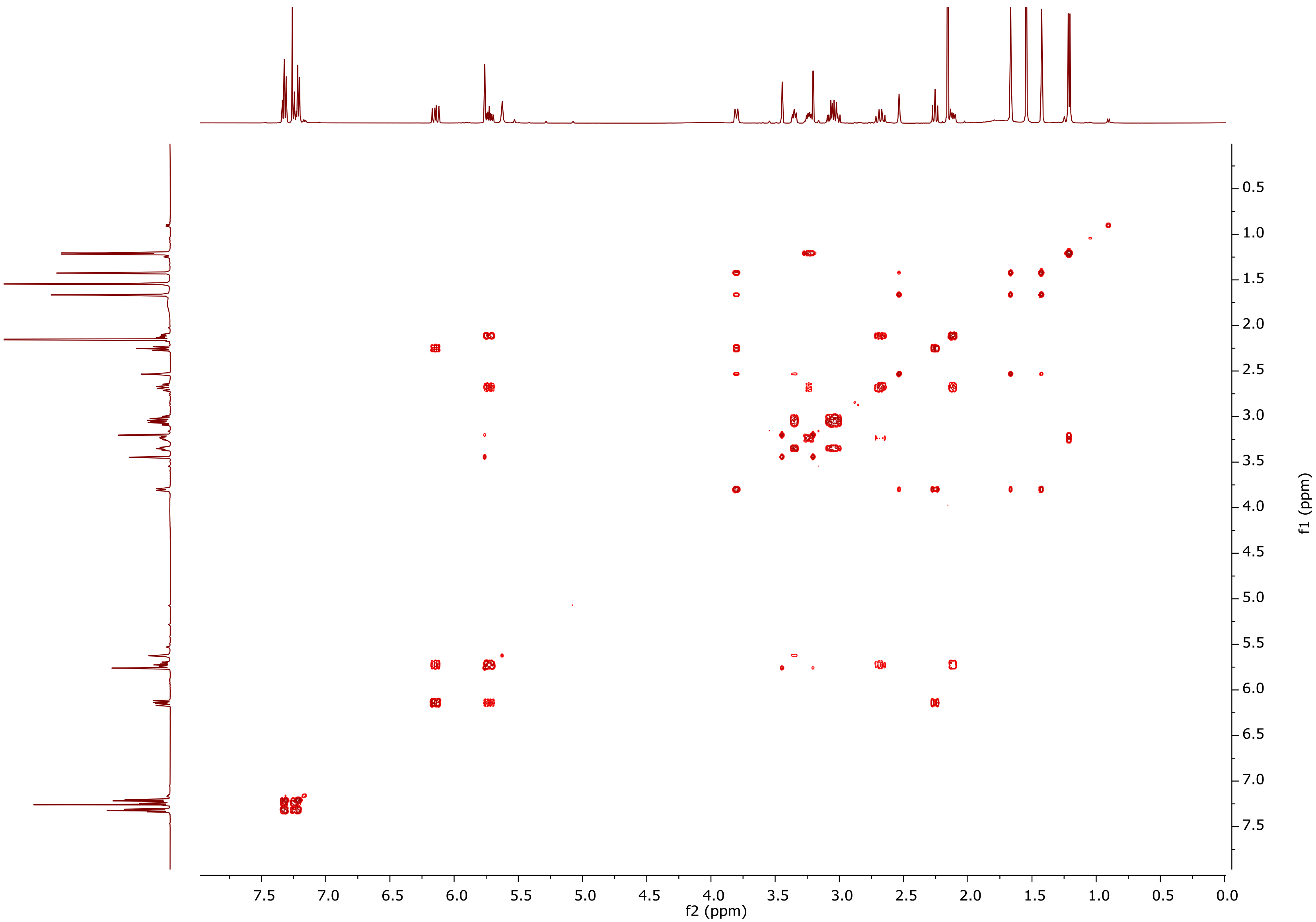

Figure SD18. ^{1}H-^{1}H COSY NMR spectrum of 19,20-epoxycytochalasin C (**3**) (500/500 MHz, $CDCl_3$)

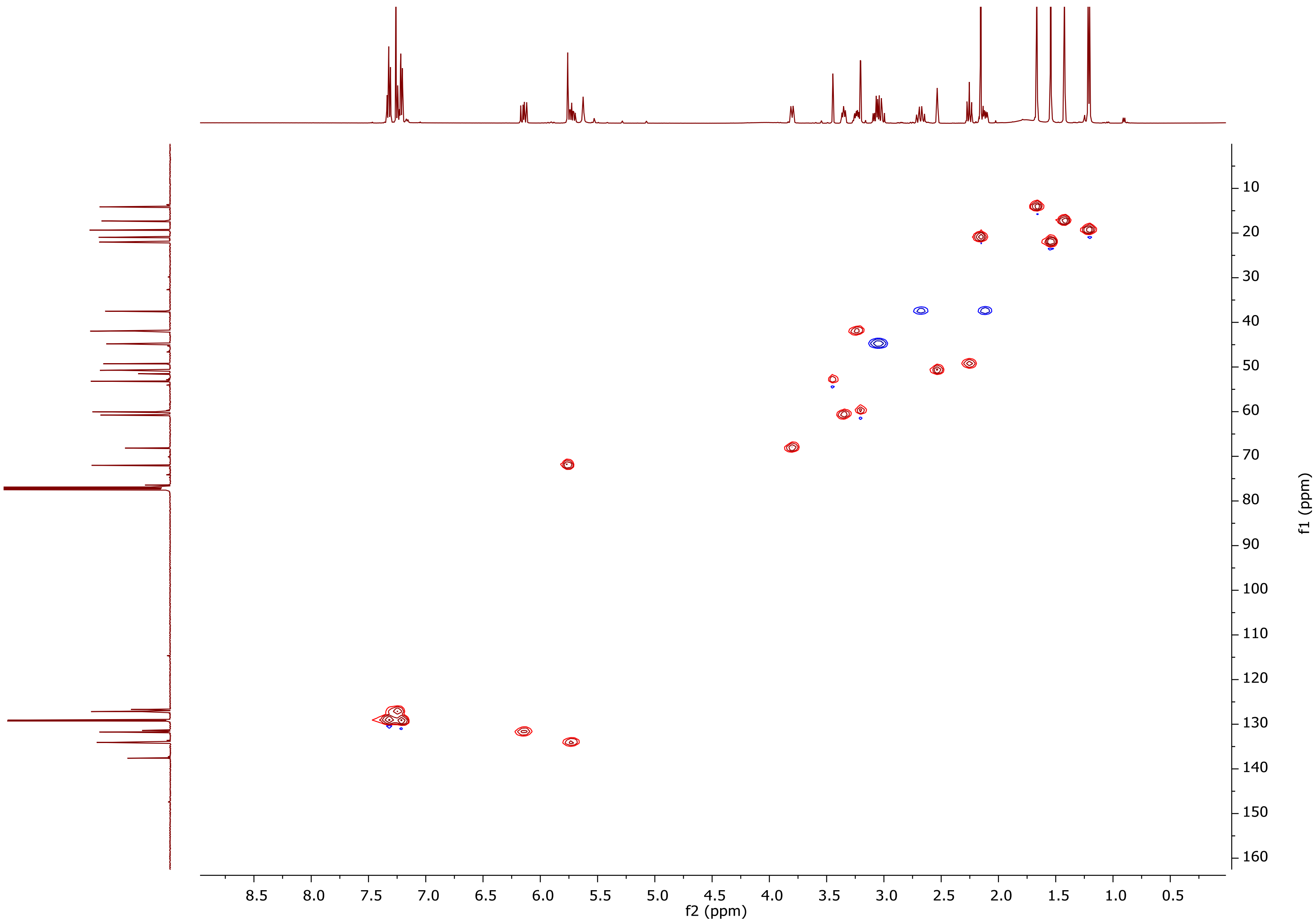

Figure SD19. ^{1}H-^{13}C HSQC NMR spectrum of 19,20-epoxycytochalasin C (**3**) (500/125 MHz, $CDCl_3$)

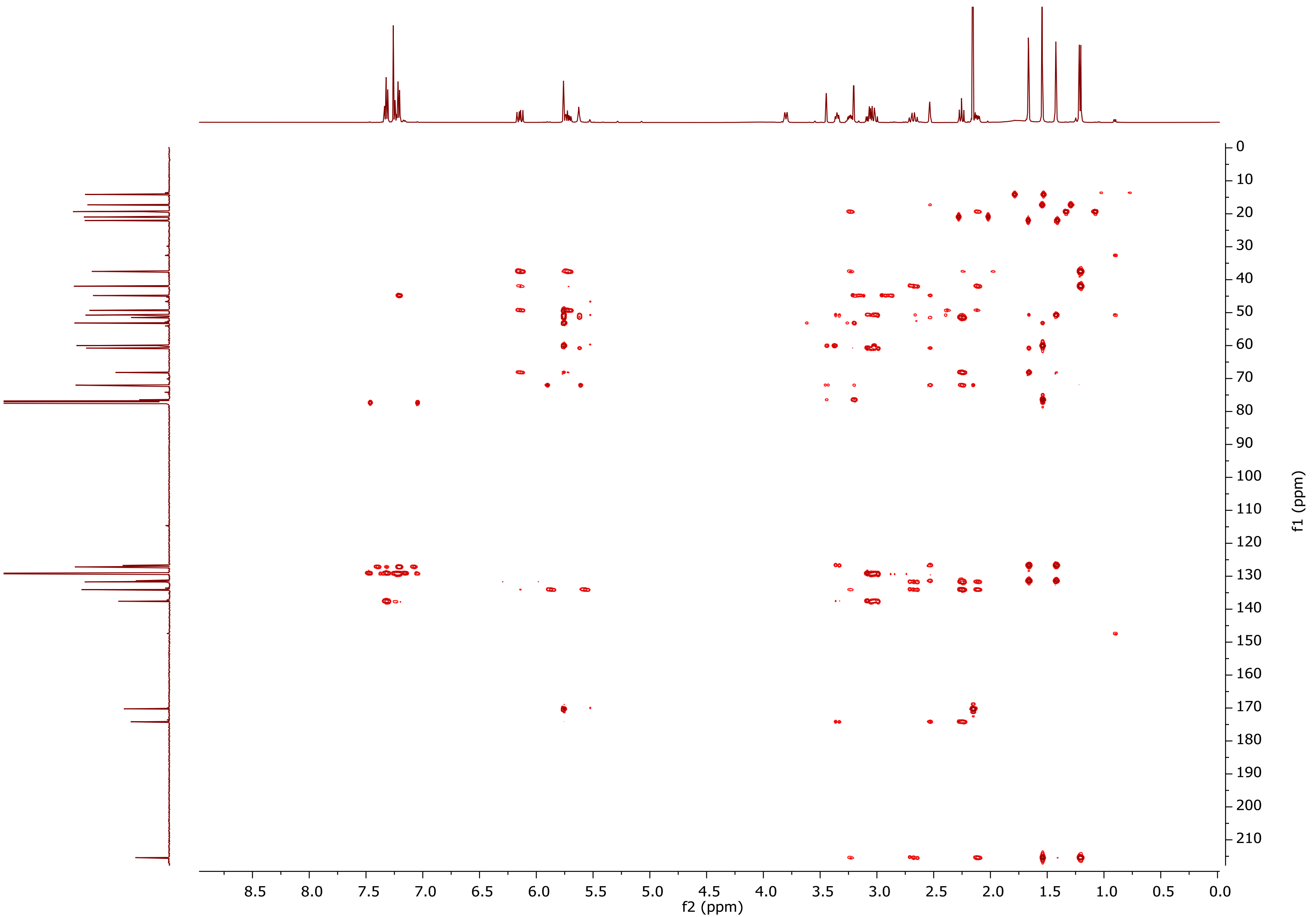

Figure SD20. ^{1}H-^{13}C HMBC NMR spectrum of 19,20-epoxycytochalasin C (**3**) (500/125 MHz, $CDCl_3$)

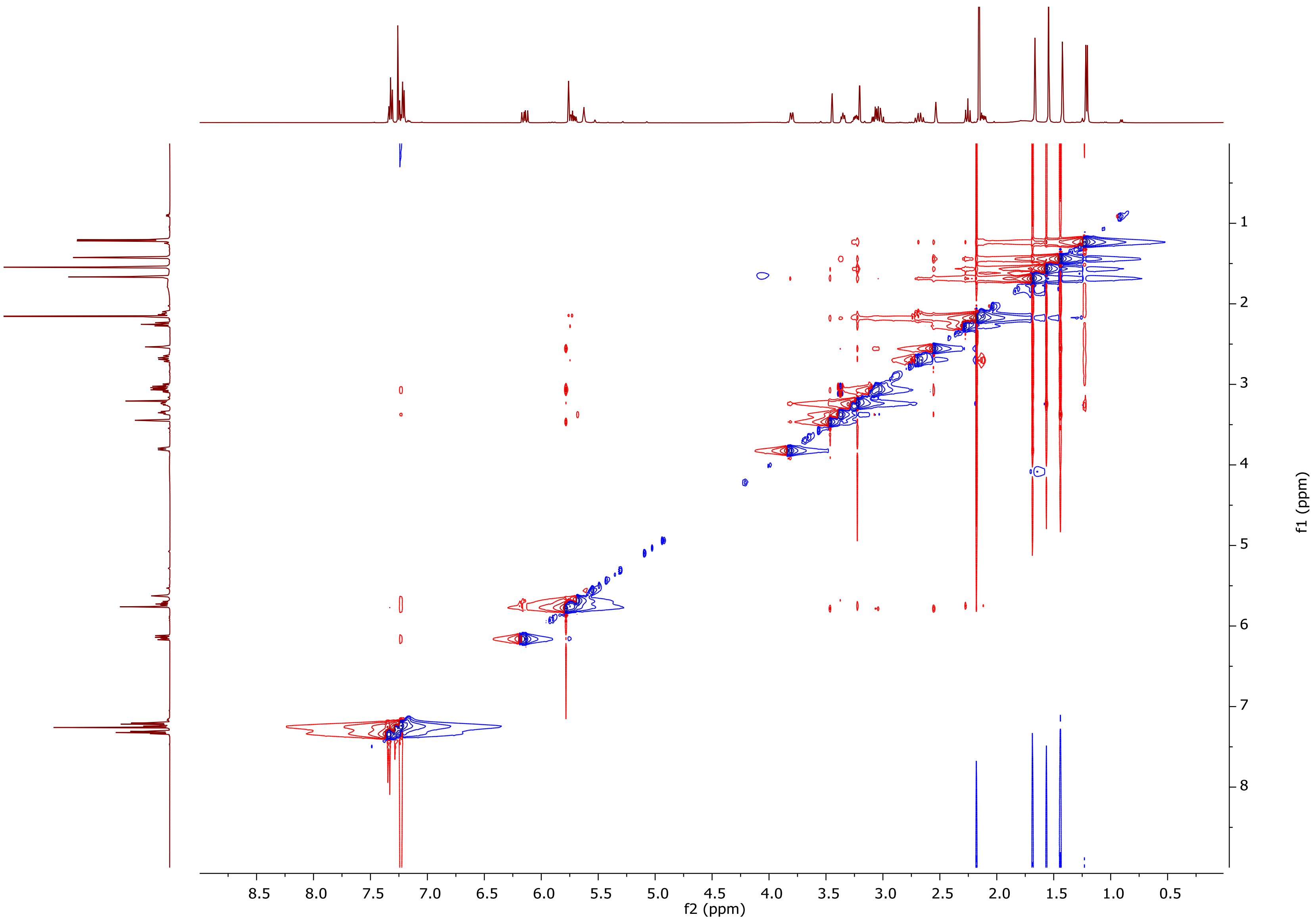

Figure SD21. ^1H-^1H NOESY NMR spectrum of 19,20-epoxycytochalasin C (**3**) (500/500 MHz, $CDCl_3$)

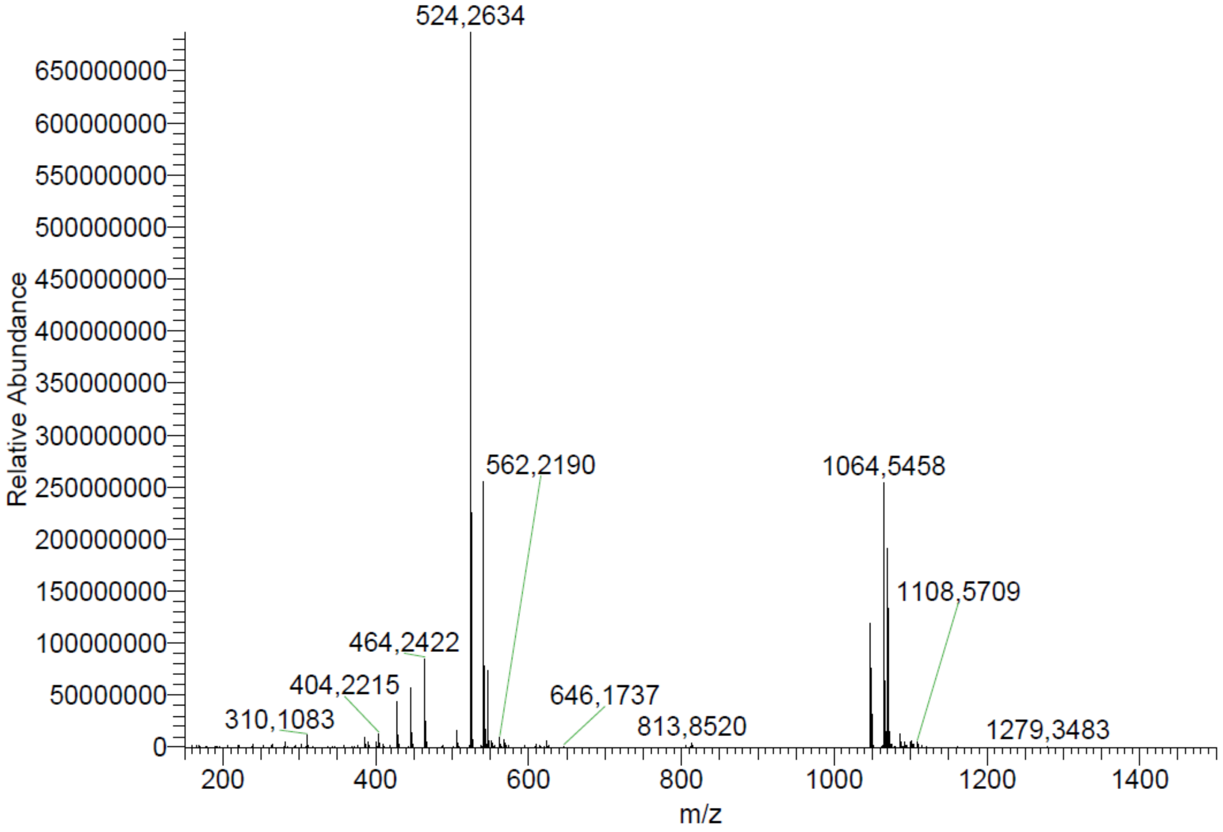

Figure SD22. ESI-HRMS spectrum of 19,20-epoxycytochalasin C (**3**)

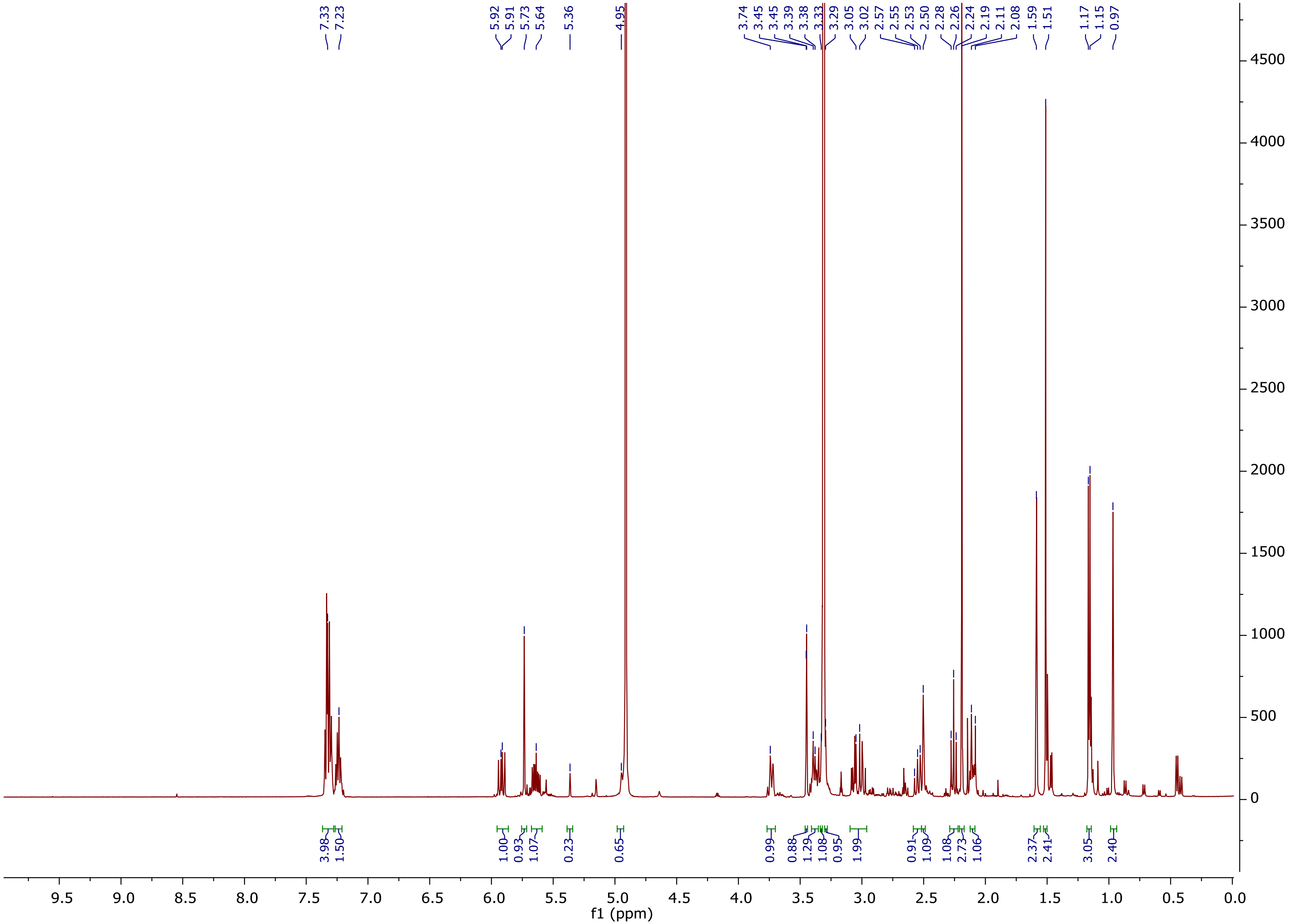

Figure SD23. ^{1}H NMR spectrum of 19,20-epoxycytochalasin C (**3**) (500 MHz, MeOH-d_4)

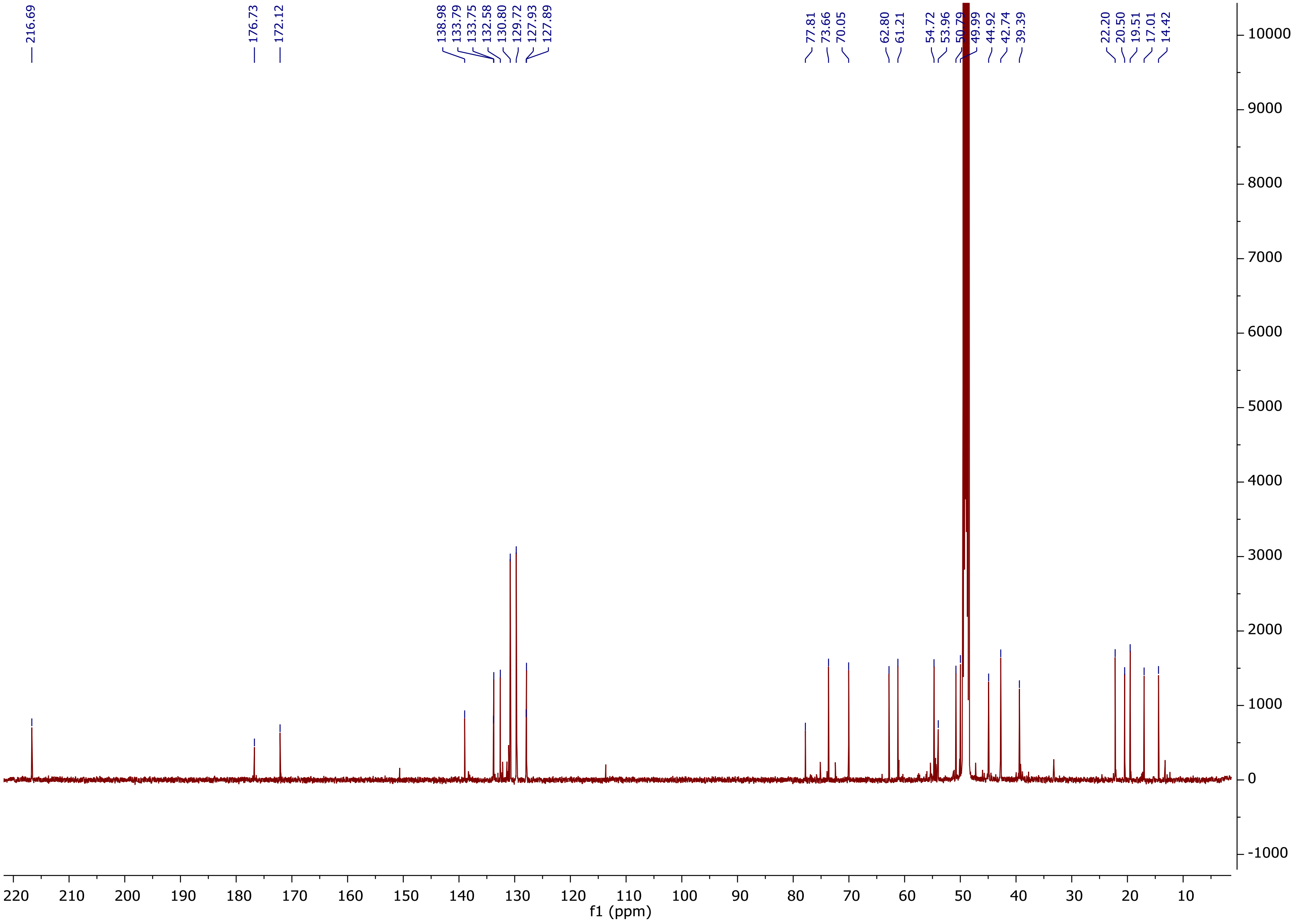

Figure SD24. ^{13}C NMR spectrum of 19,20-epoxycytochalasin C (**3**) (125 MHz, MeOH-d_4)

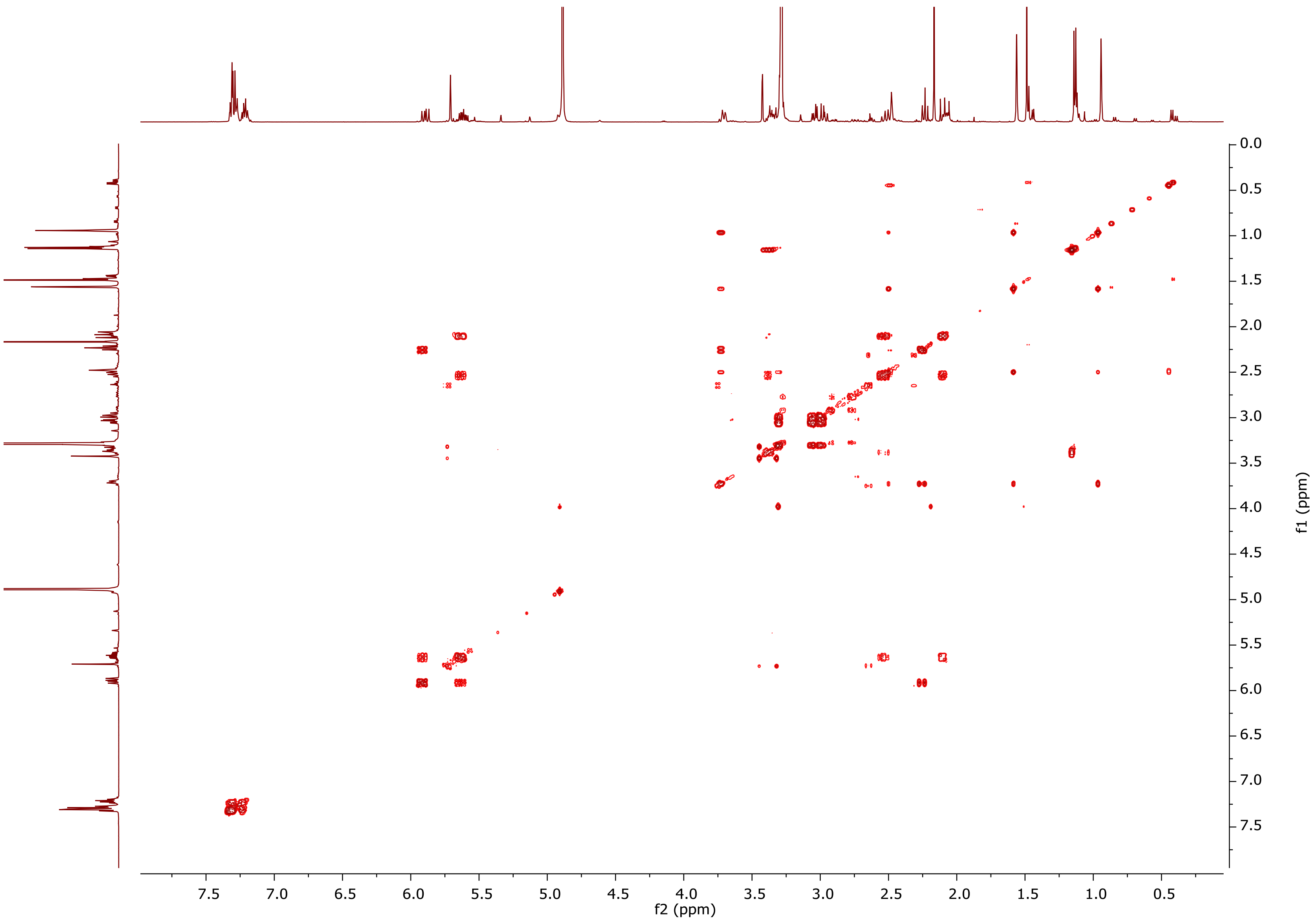

Figure SD25. ^{1}H-^{1}H COSY NMR spectrum of 19,20-epoxycytochalasin C (**3**) (500/500 MHz, MeOH-d_4)

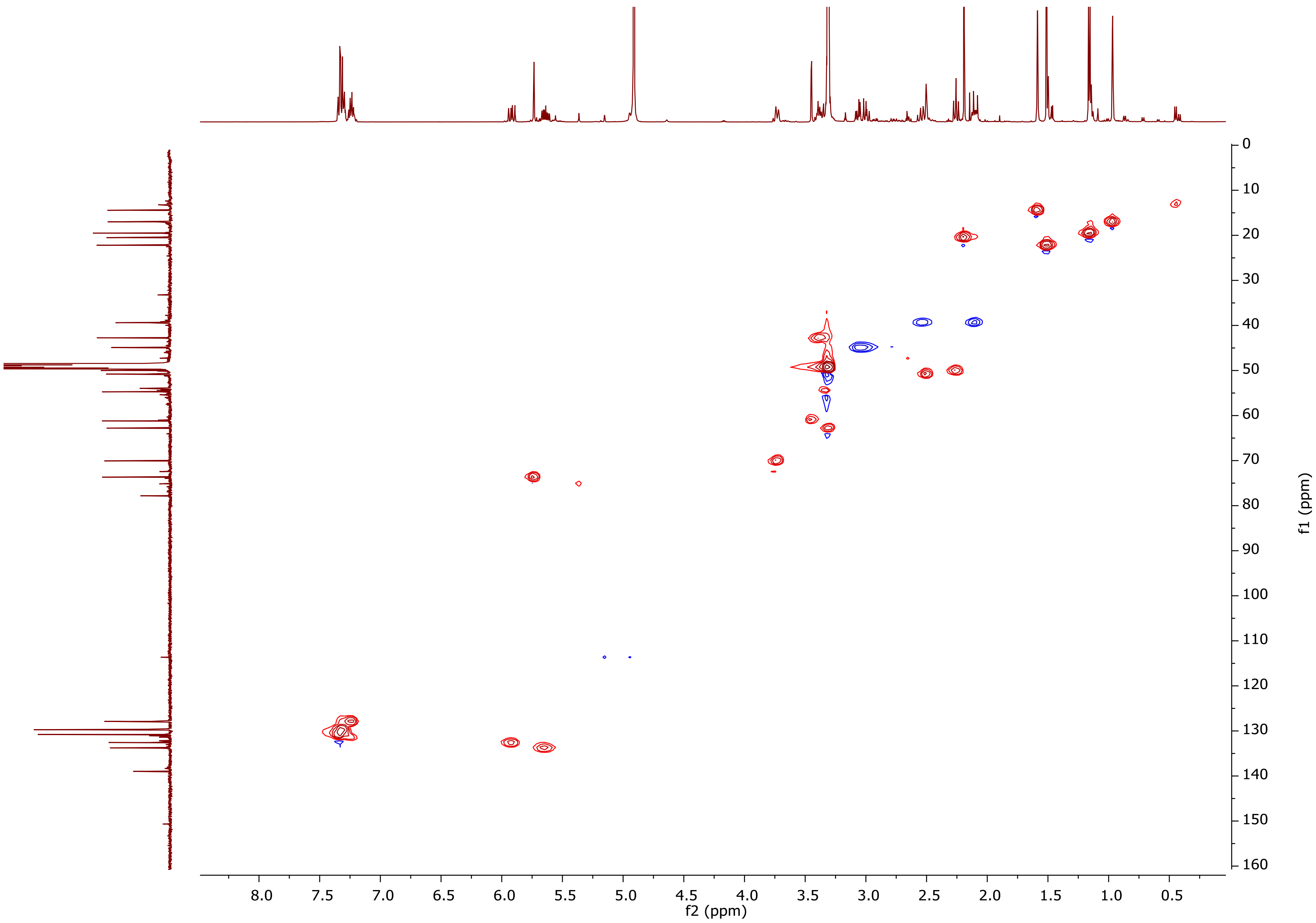

Figure SD26. ^{1}H-^{13}C HSQC NMR spectrum of 19,20-epoxycytochalasin C (**3**) (500/125 MHz, MeOH-d_4)

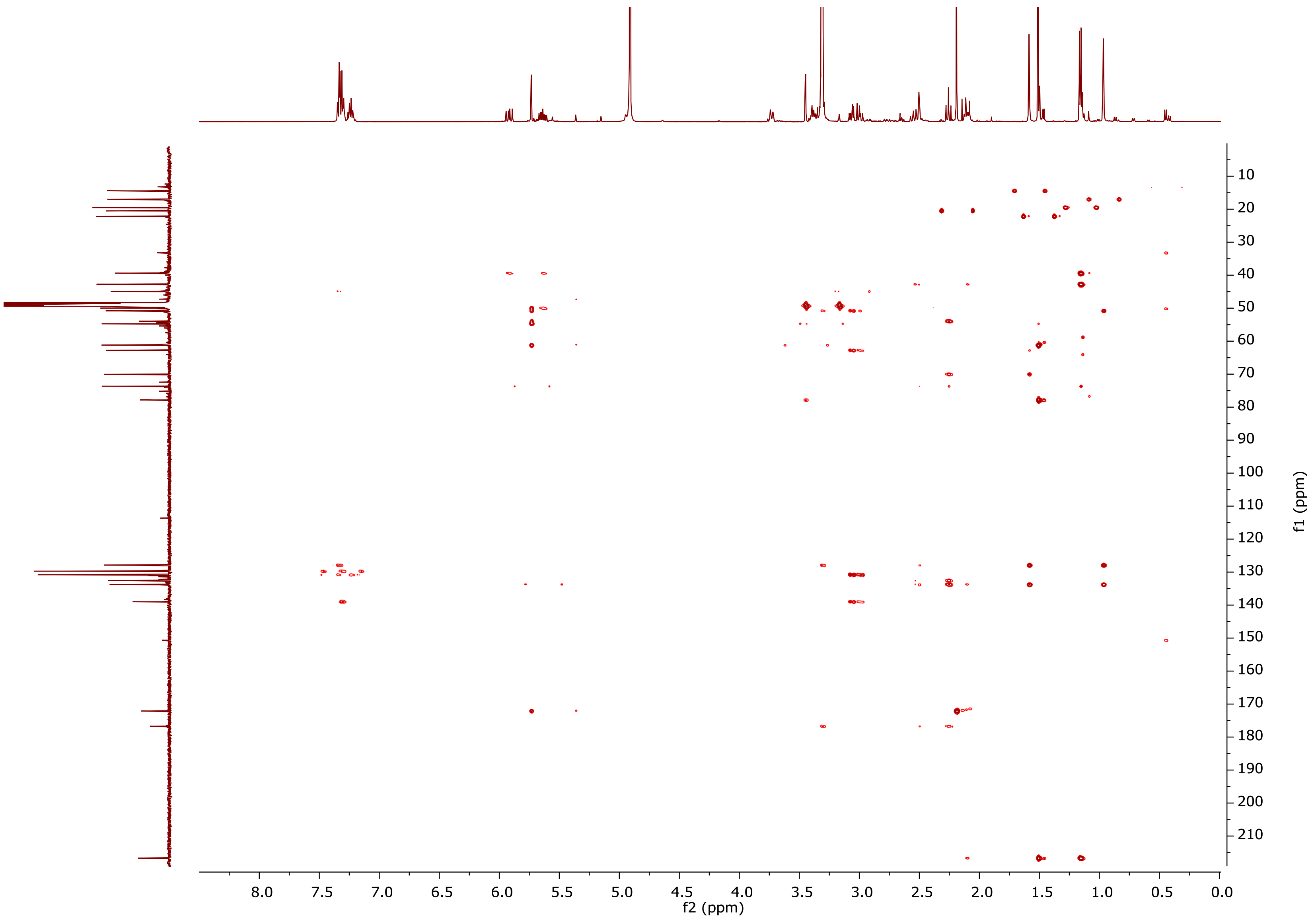

Figure SD27. ^{1}H-^{13}C HMBC NMR spectrum of 19,20-epoxycytochalasin C (**3**) (500/125 MHz, MeOH-d_4)

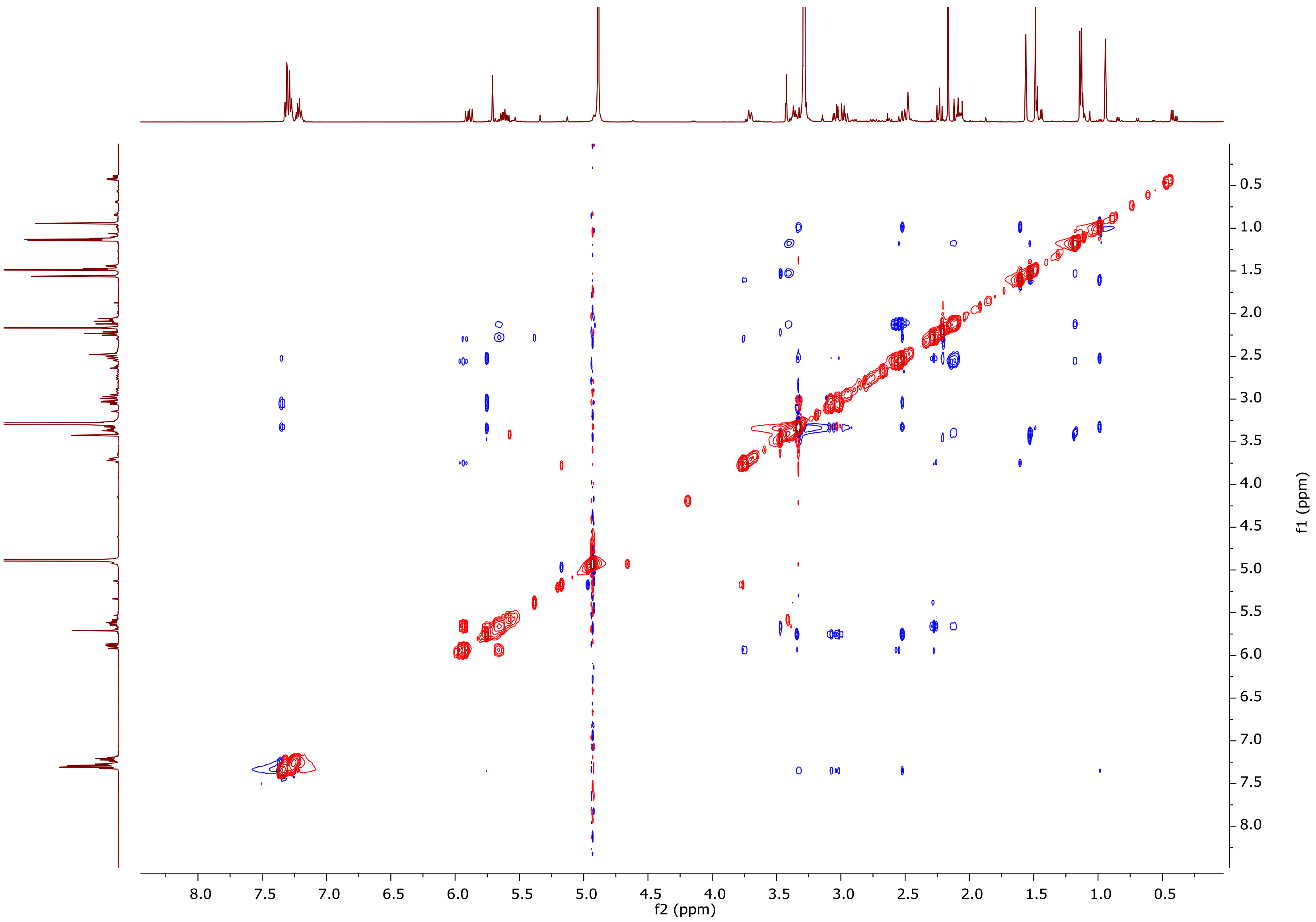

Figure SD28. ^{1}H-^{1}H NOESY NMR spectrum of 19,20-epoxycytochalasin C (**3**) (500/500 MHz, MeOH-*d*$_4$)

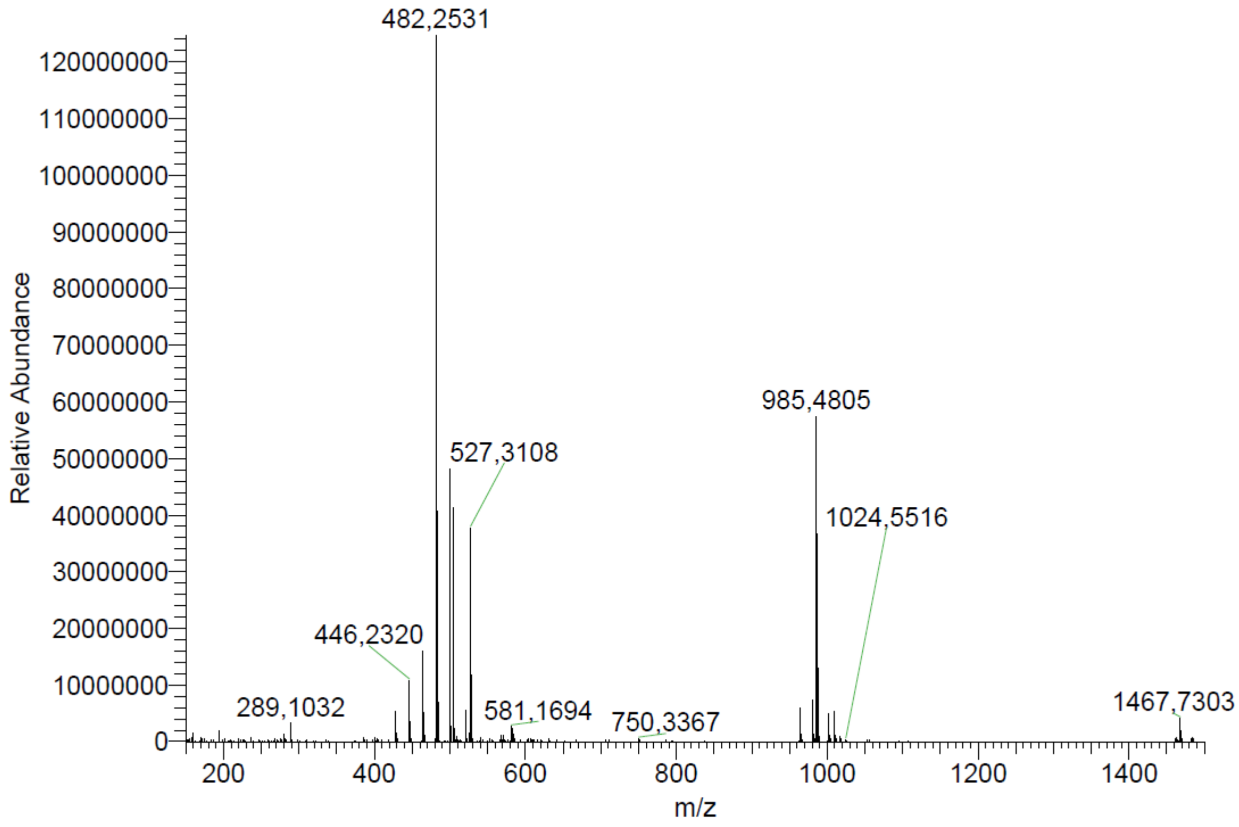

Figure SD29. ESI-HRMS spectrum of deacetyl-19,20-epoxycytochalasin C (**4**)

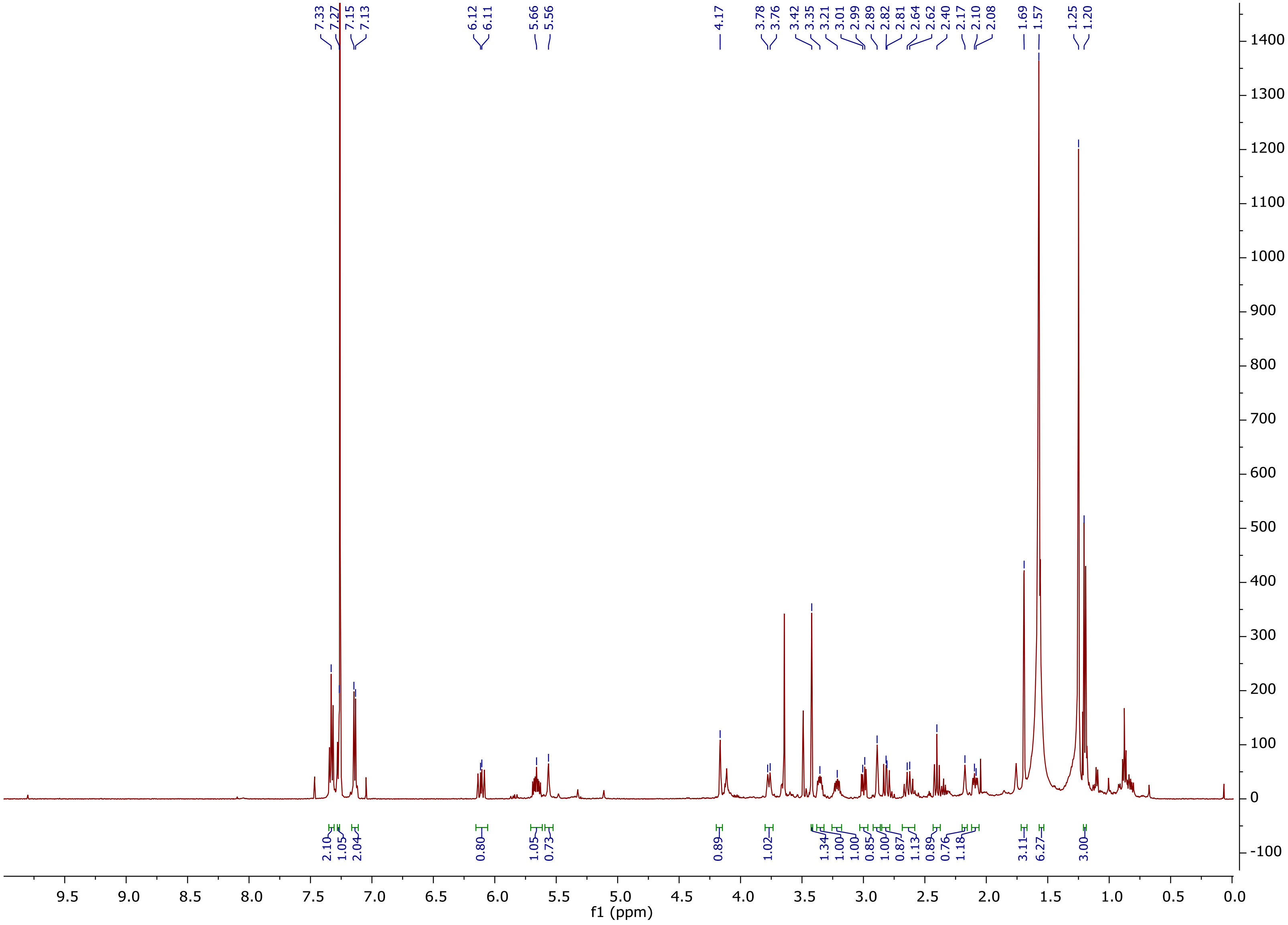

Figure SD30. ^{1}H NMR spectrum of deacetyl-19,20-epoxycytochalasin C (**4**) (500 MHz, $CDCl_3$)

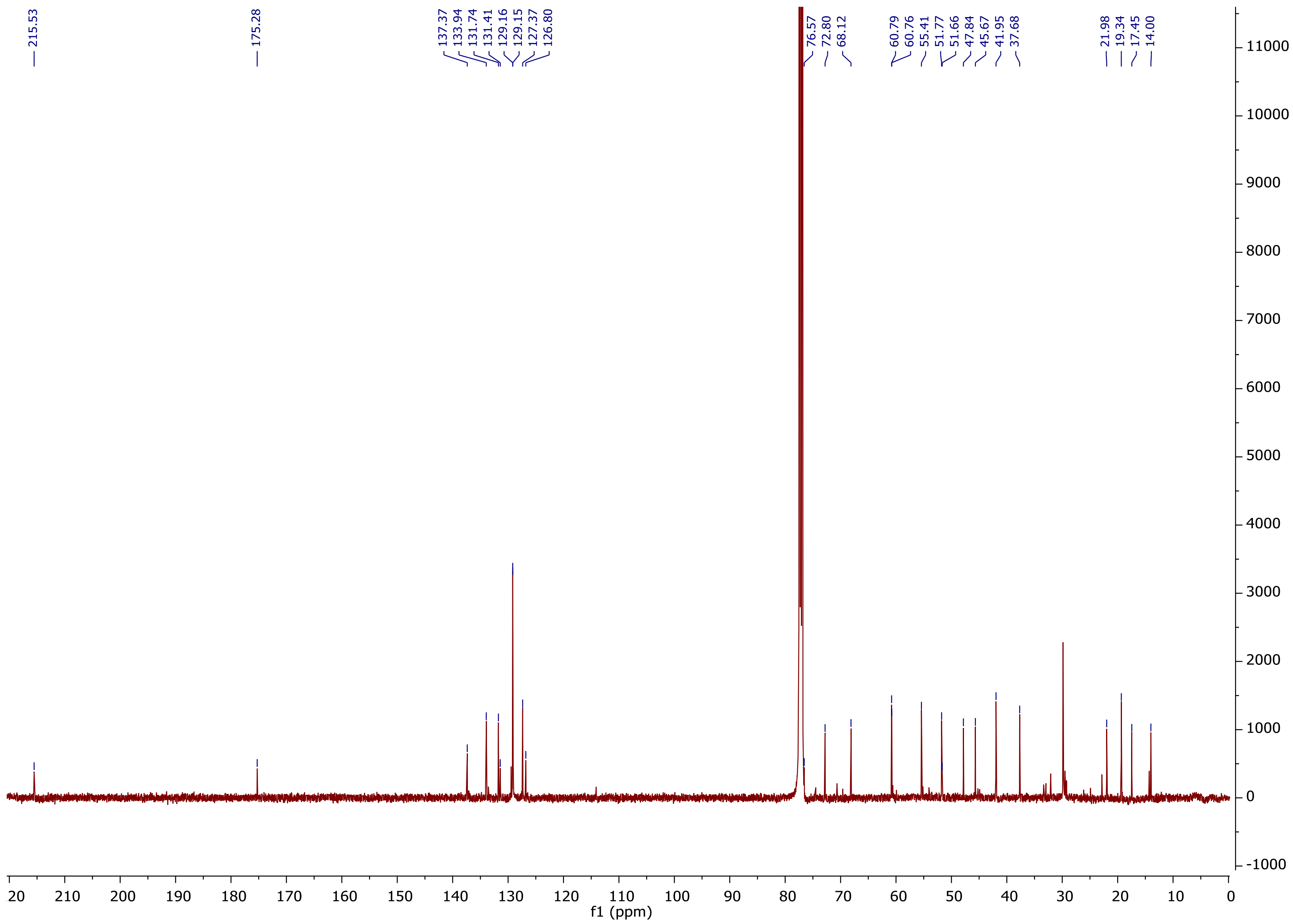

Figure SD31. ^{13}C NMR spectrum of deacetyl-19,20-epoxycytochalasin C (**4**) (125 MHz, $CDCl_3$)

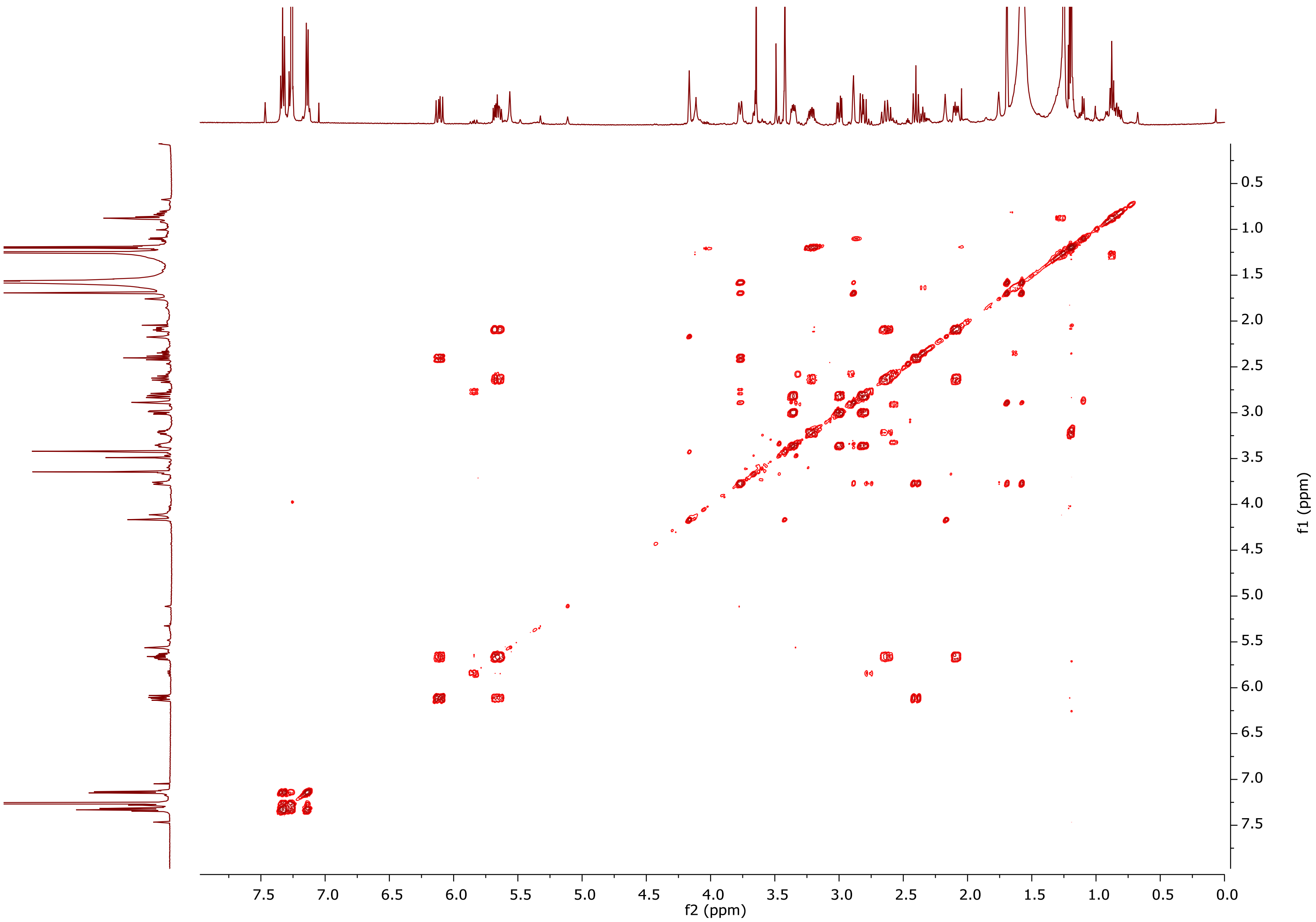

Figure SD32. ^{1}H-^{1}H COSY NMR spectrum of deacetyl-19,20-epoxycytochalasin C (**4**) (500/500 MHz, $CDCl_3$)

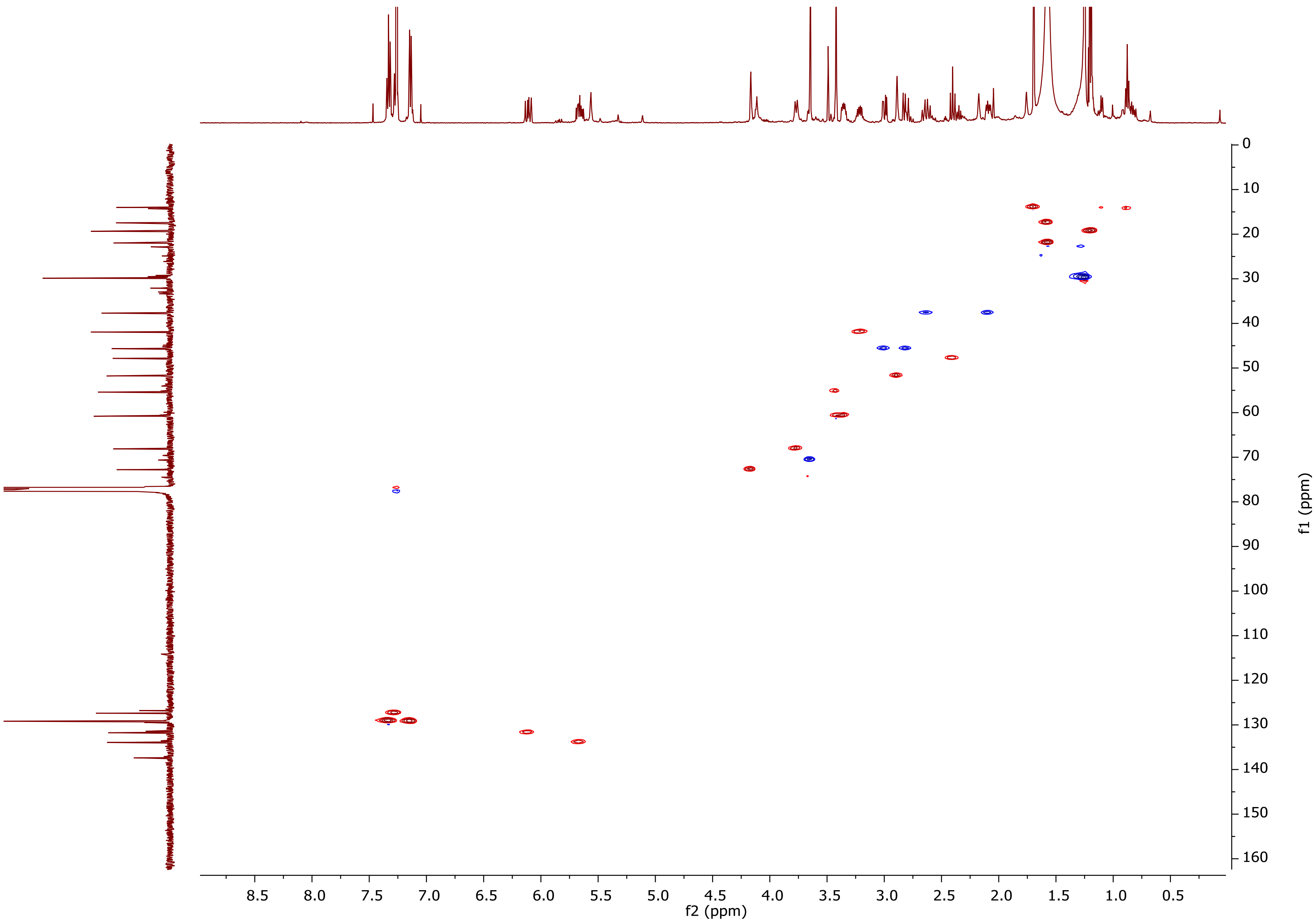

Figure SD33. ^{1}H-^{13}C HSQC NMR spectrum of deacetyl-19,20-epoxycytochalasin C (**4**) (500/125 MHz, $CDCl_3$)

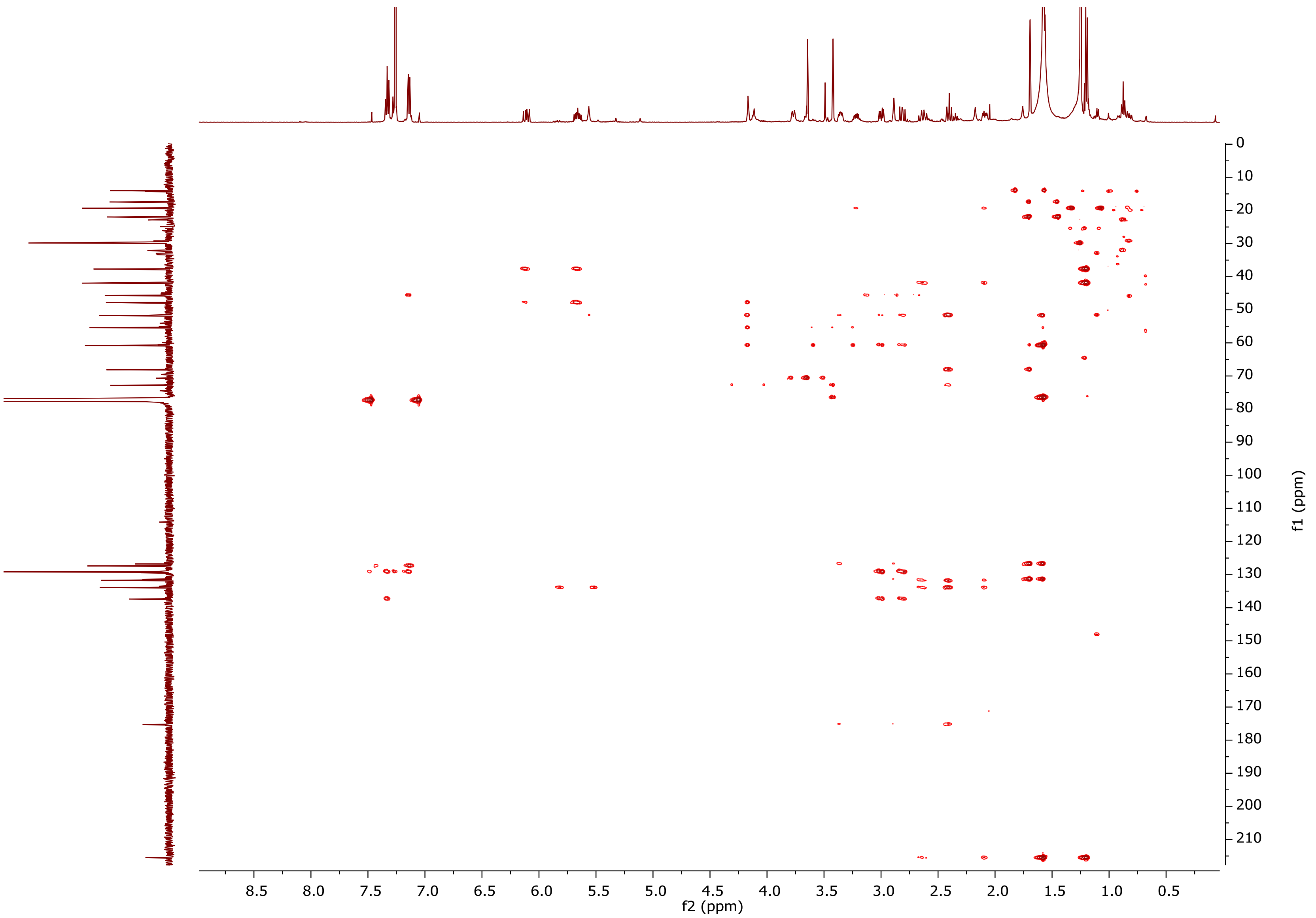

Figure SD34. ^{1}H-^{13}C HMBC NMR spectrum of deacetyl-19,20-epoxycytochalasin C (**4**) (500/125 MHz, $CDCl_3$)

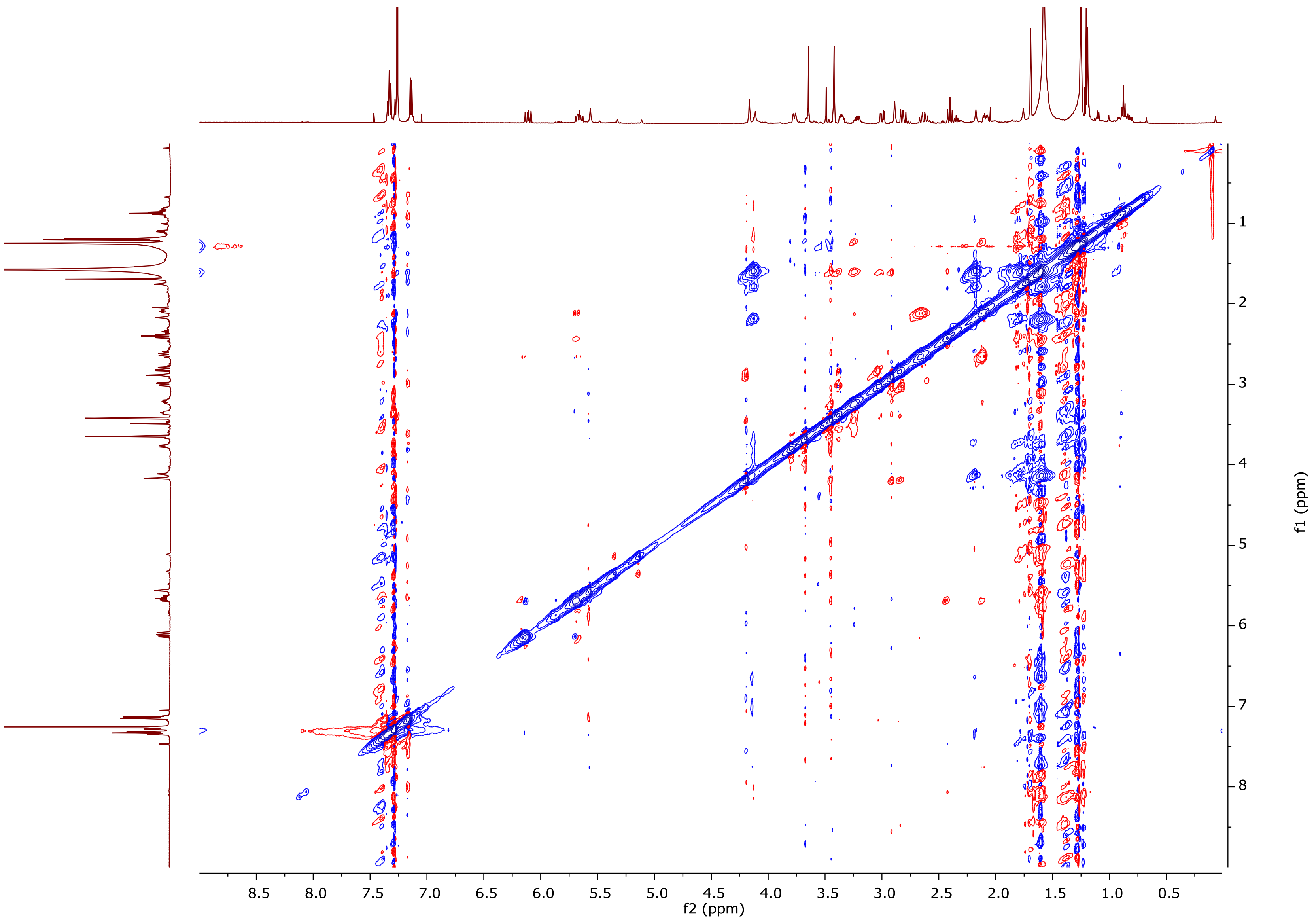

Figure SD35. ^{1}H-^{1}H NOESY NMR spectrum of deacetyl-19,20-epoxycytochalasin C (**4**) (500/500 MHz, $CDCl_3$)

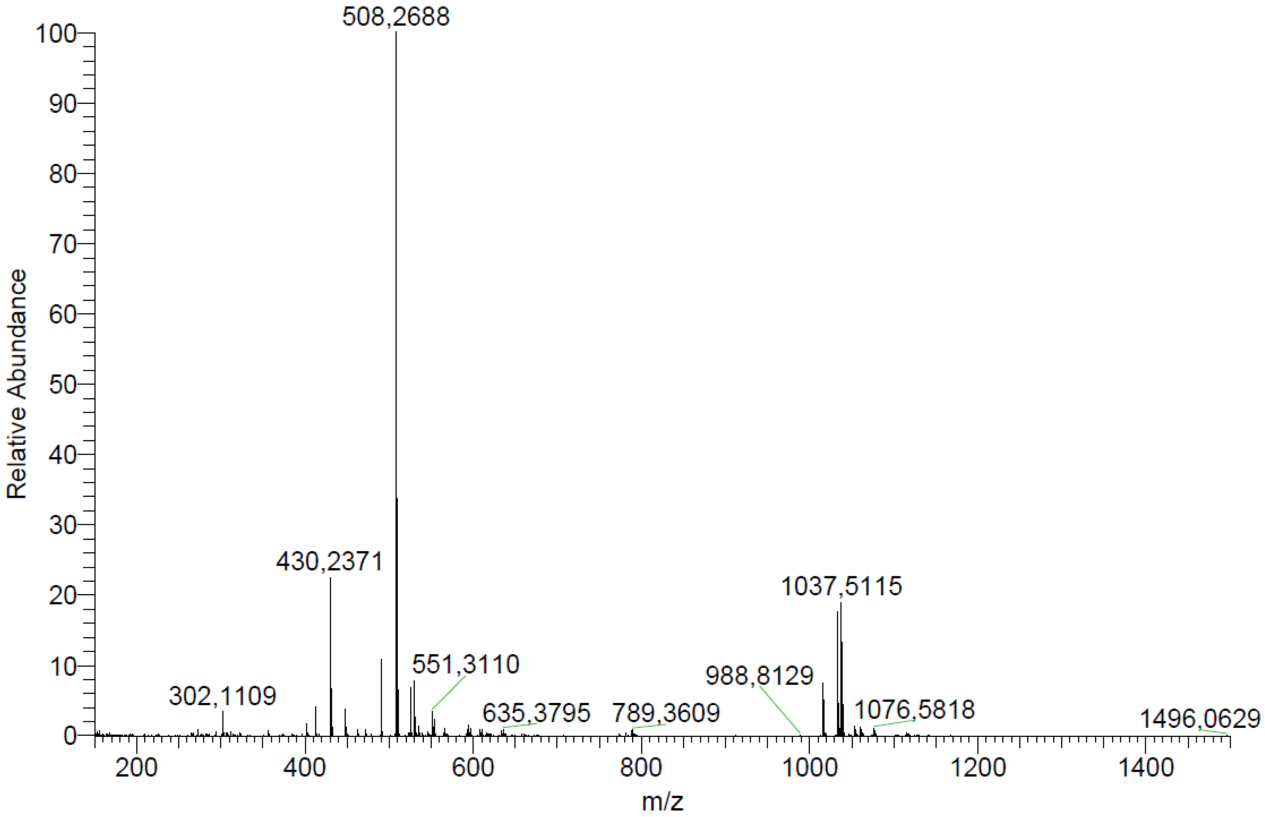

Figure SD36. ESI-HRMS spectrum of 18-desoxy-19,20-epoxycytochalasin C (**5**)

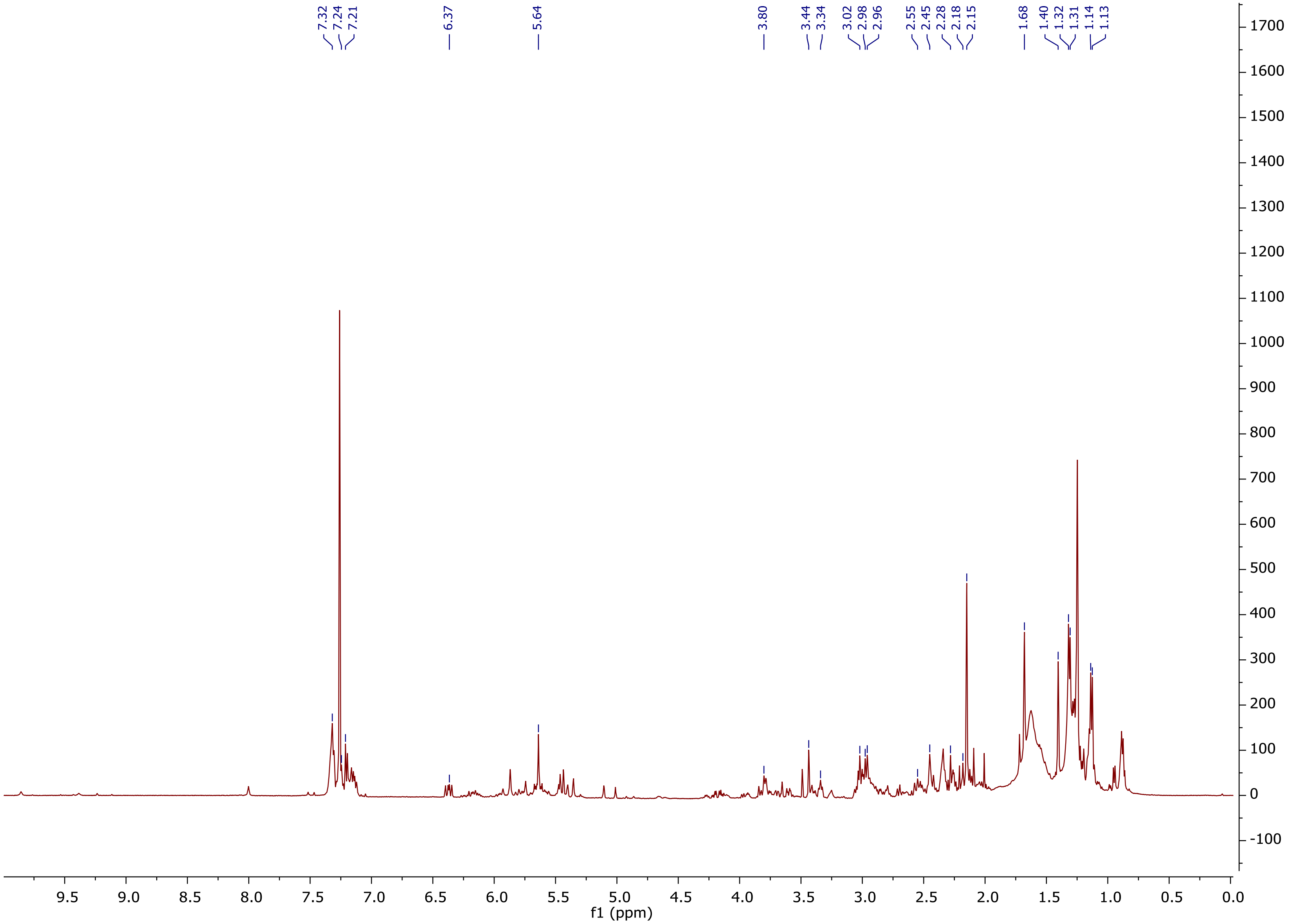

Figure SD37. ^{1}H NMR spectrum of 18-desoxy-19,20-epoxycytochalasin C (**5**) (500 MHz, $CDCl_3$)

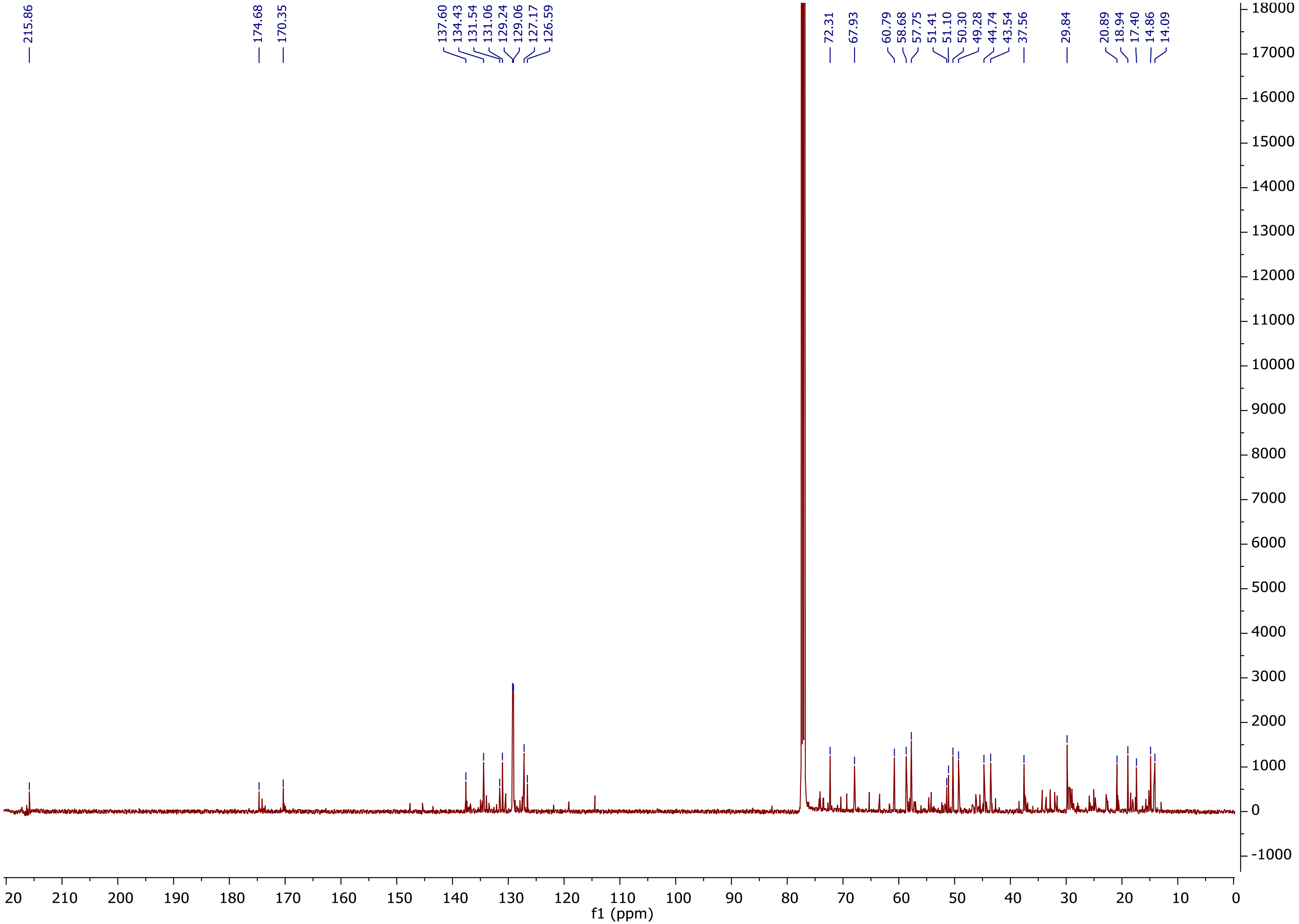

Figure SD38. ^{13}C NMR spectrum of 18-desoxy-19,20-epoxycytochalasin C (**5**) (125 MHz, $CDCl_3$)

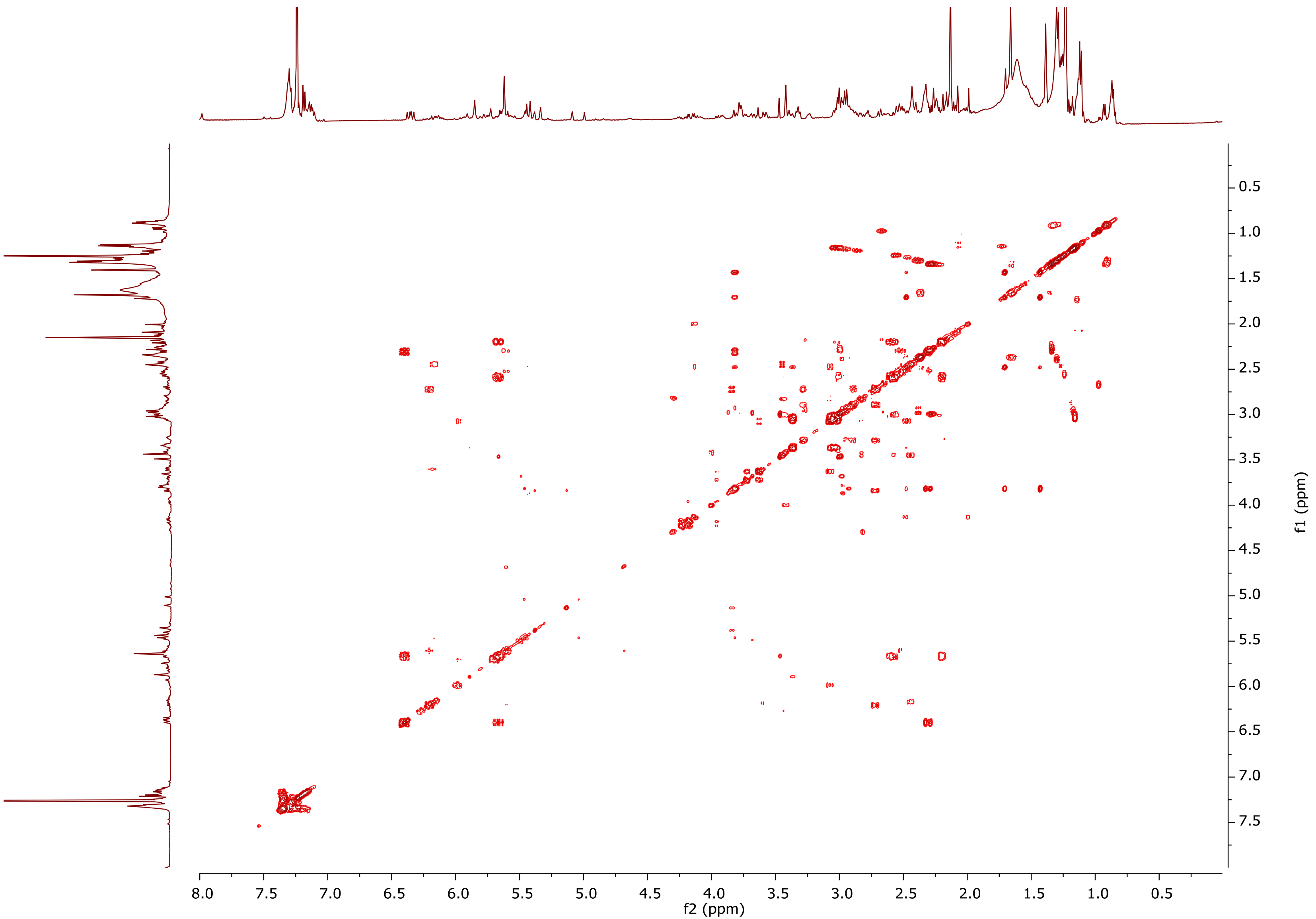

Figure SD39. ^{1}H-^{1}H COSY NMR spectrum of 18-desoxy-19,20-epoxycytochalasin C (**5**) (500/500 MHz, $CDCl_3$)

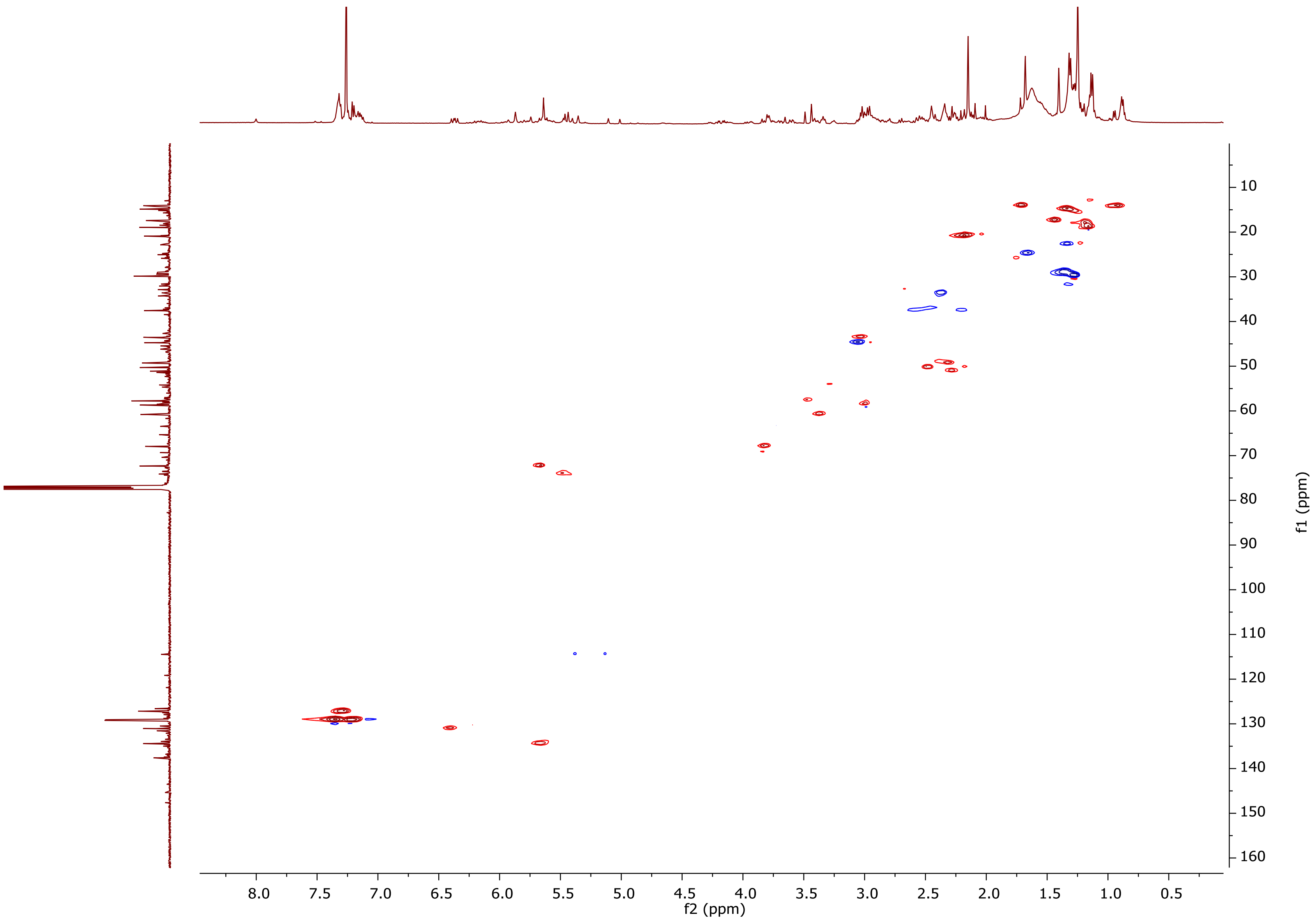

Figure SD40. ^{1}H-^{13}C HSQC NMR spectrum of 18-desoxy-19,20-epoxycytochalasin C (**5**) (500/125 MHz, $CDCl_3$)

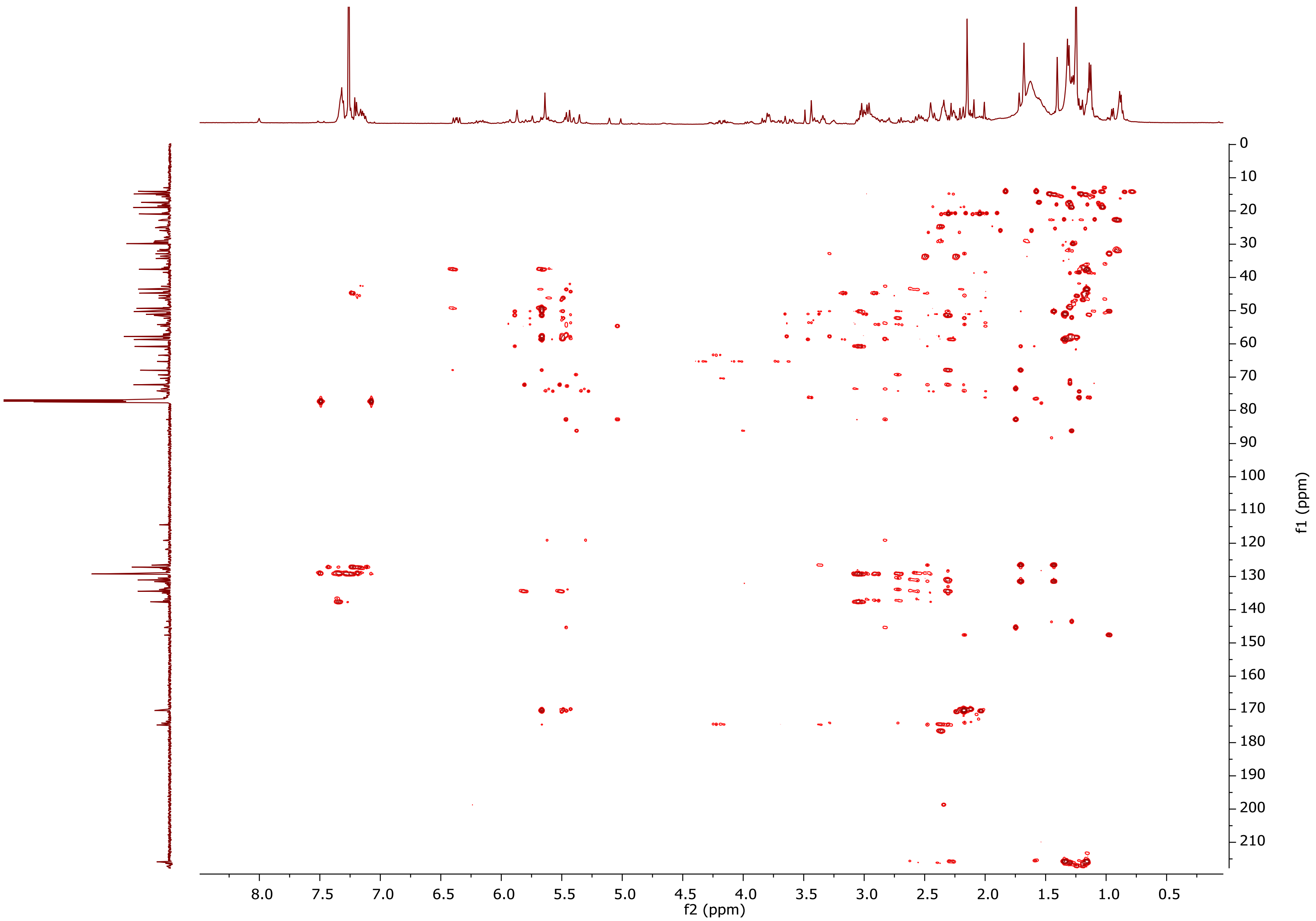

Figure SD41. ^{1}H-^{13}C HMBC NMR spectrum of 18-desoxy-19,20-epoxycytochalasin C (**5**) (500/125 MHz, $CDCl_3$)

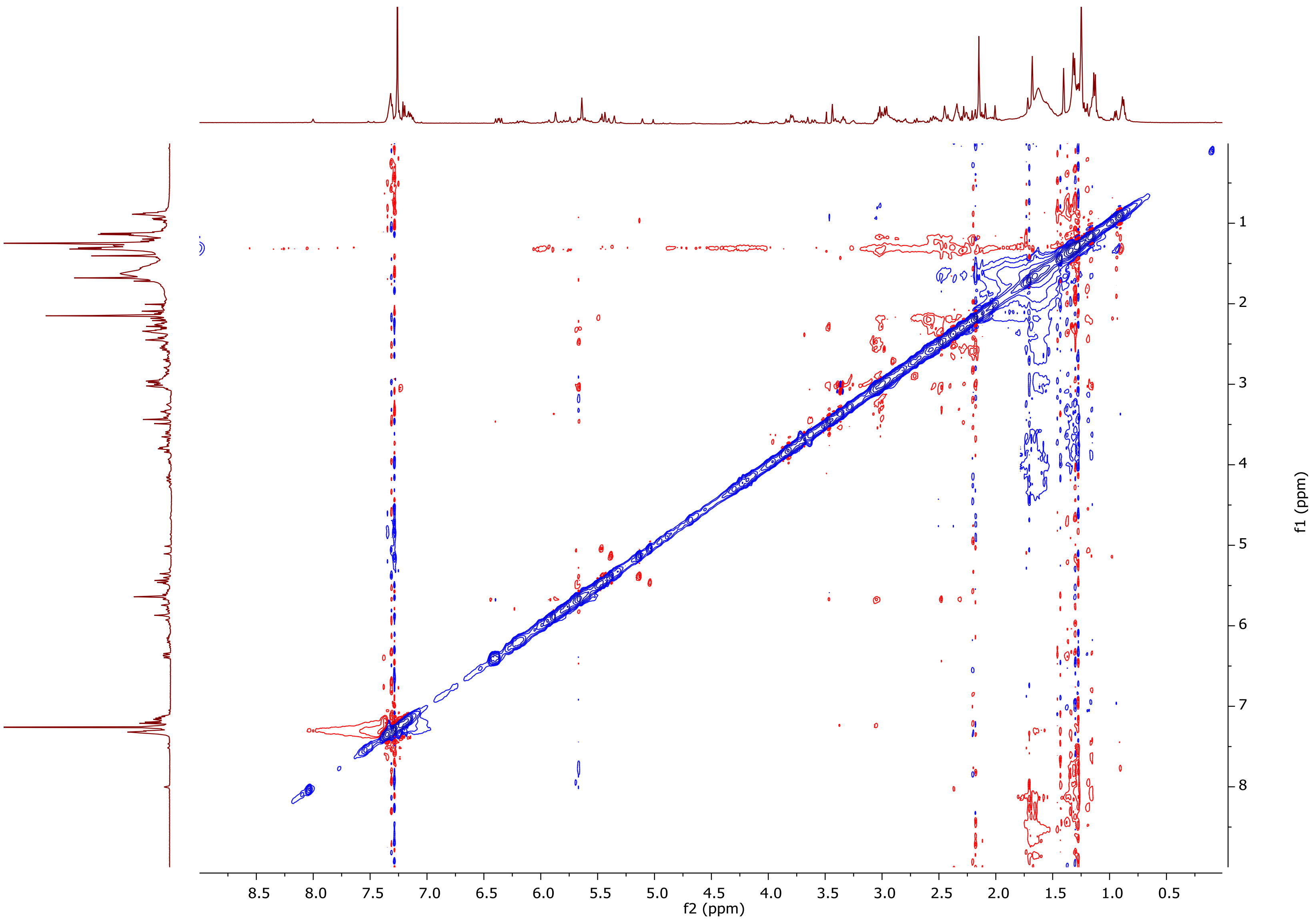

Figure SD42. ^{1}H-^{1}H NOESY NMR spectrum of 18-desoxy-19,20-epoxycytochalasin C (**5**) (500/500 MHz, $CDCl_3$)

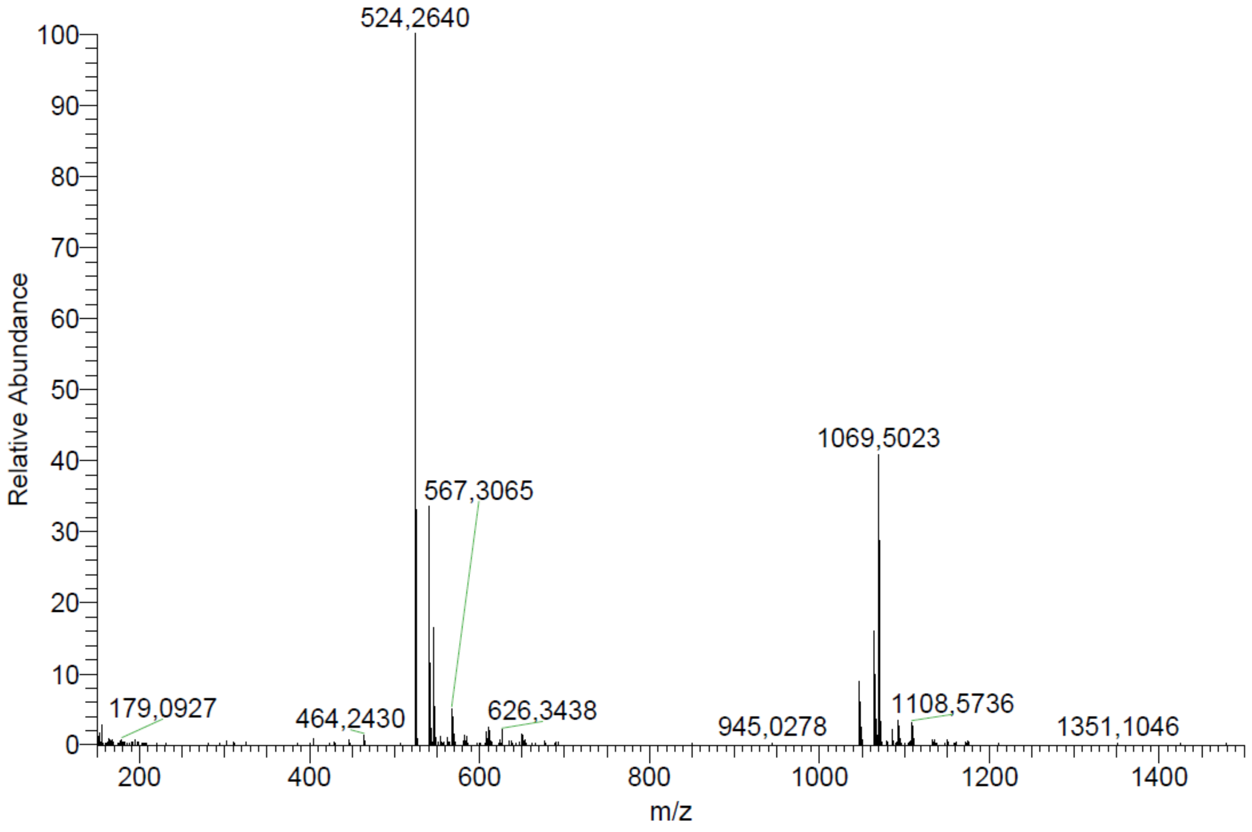

Figure SD43. ESI-HRMS spectrum of 19,20-epoxycytochalasin Q (**6**)

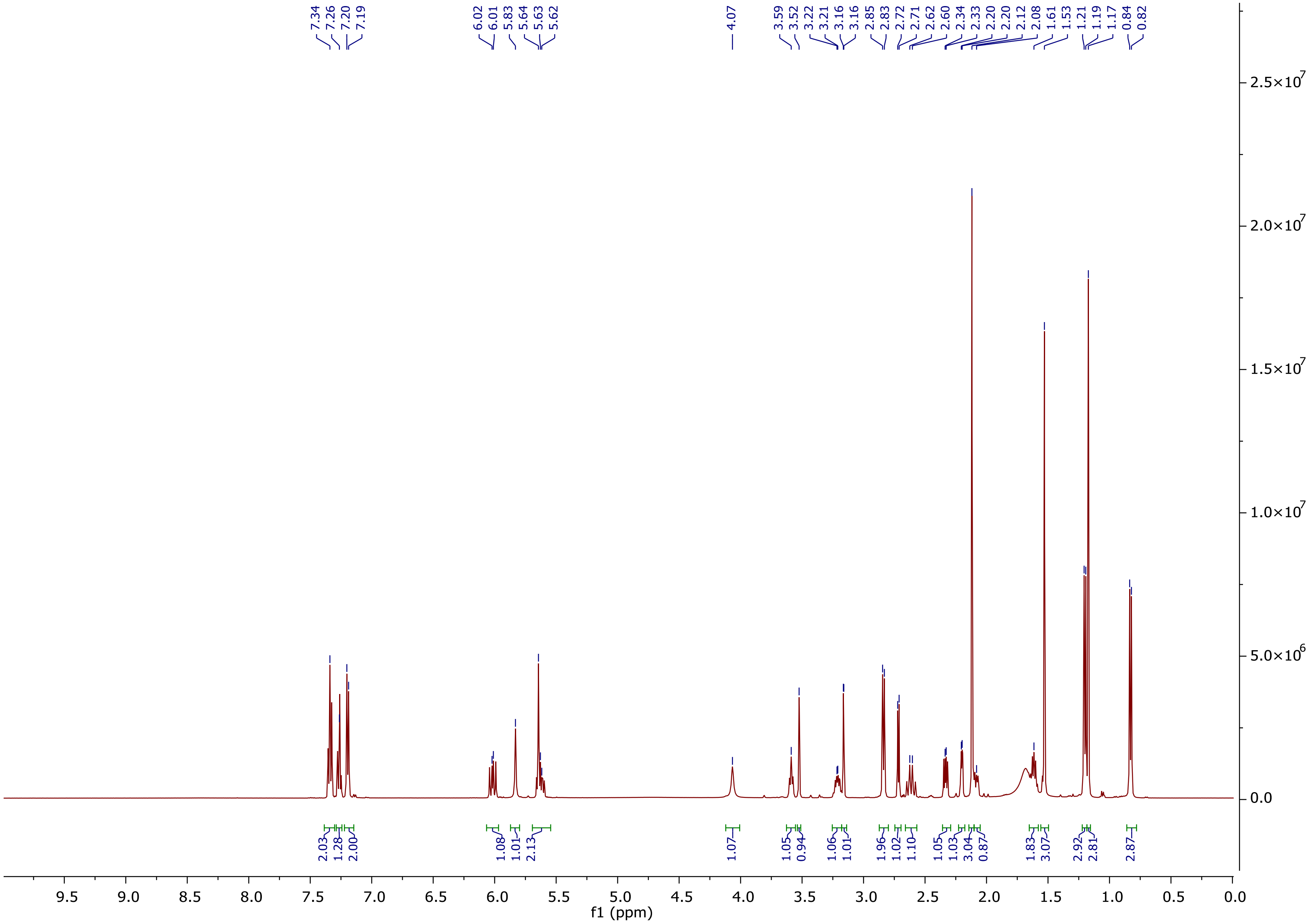

Figure SD44. ^{1}H NMR spectrum of 19,20-epoxycytochalasin Q (**6**) (500 MHz, $CDCl_3$)

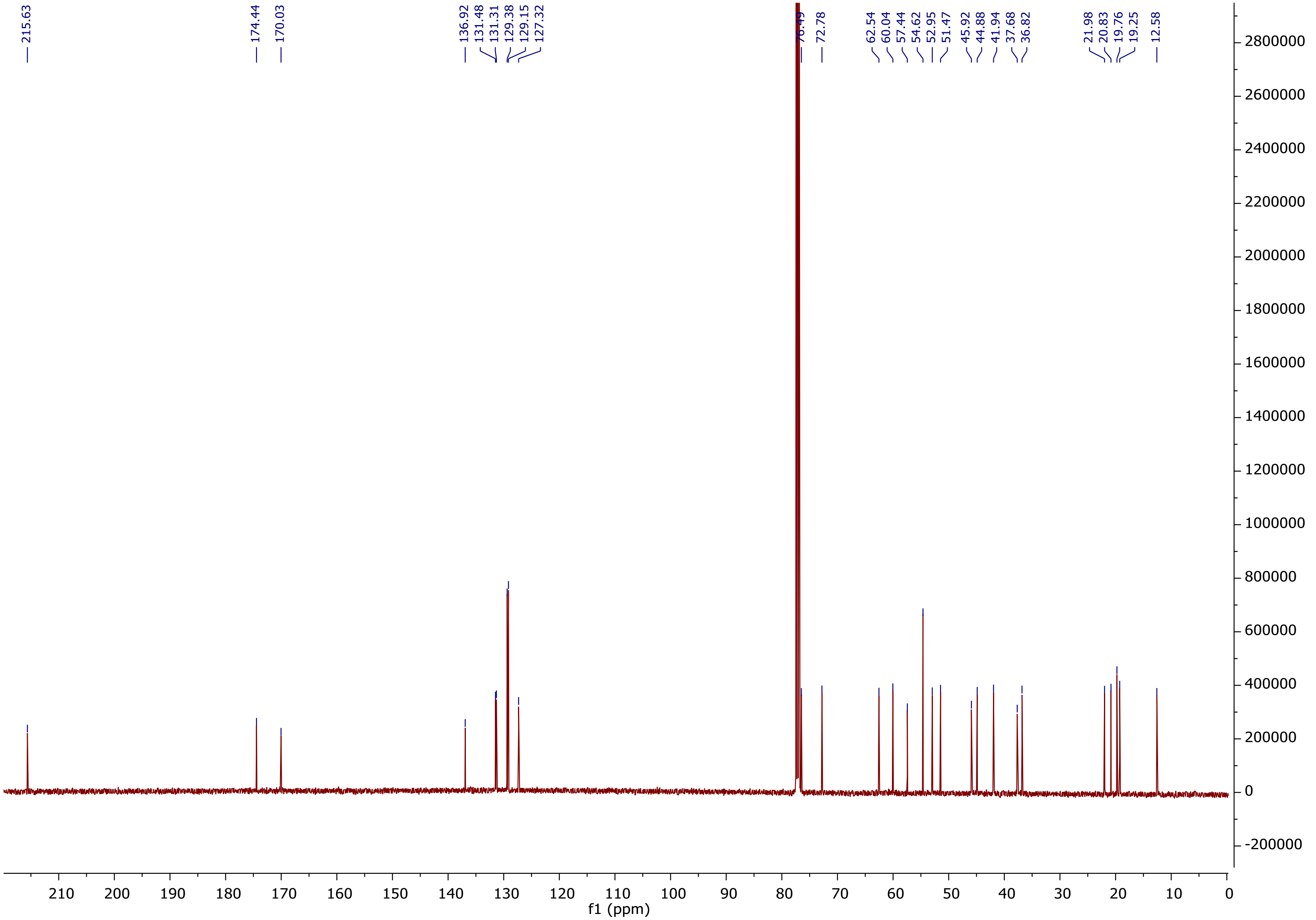

Figure SD45. ^{13}C NMR spectrum of 19,20-epoxycytochalasin Q (**6**) (125 MHz, $CDCl_3$)

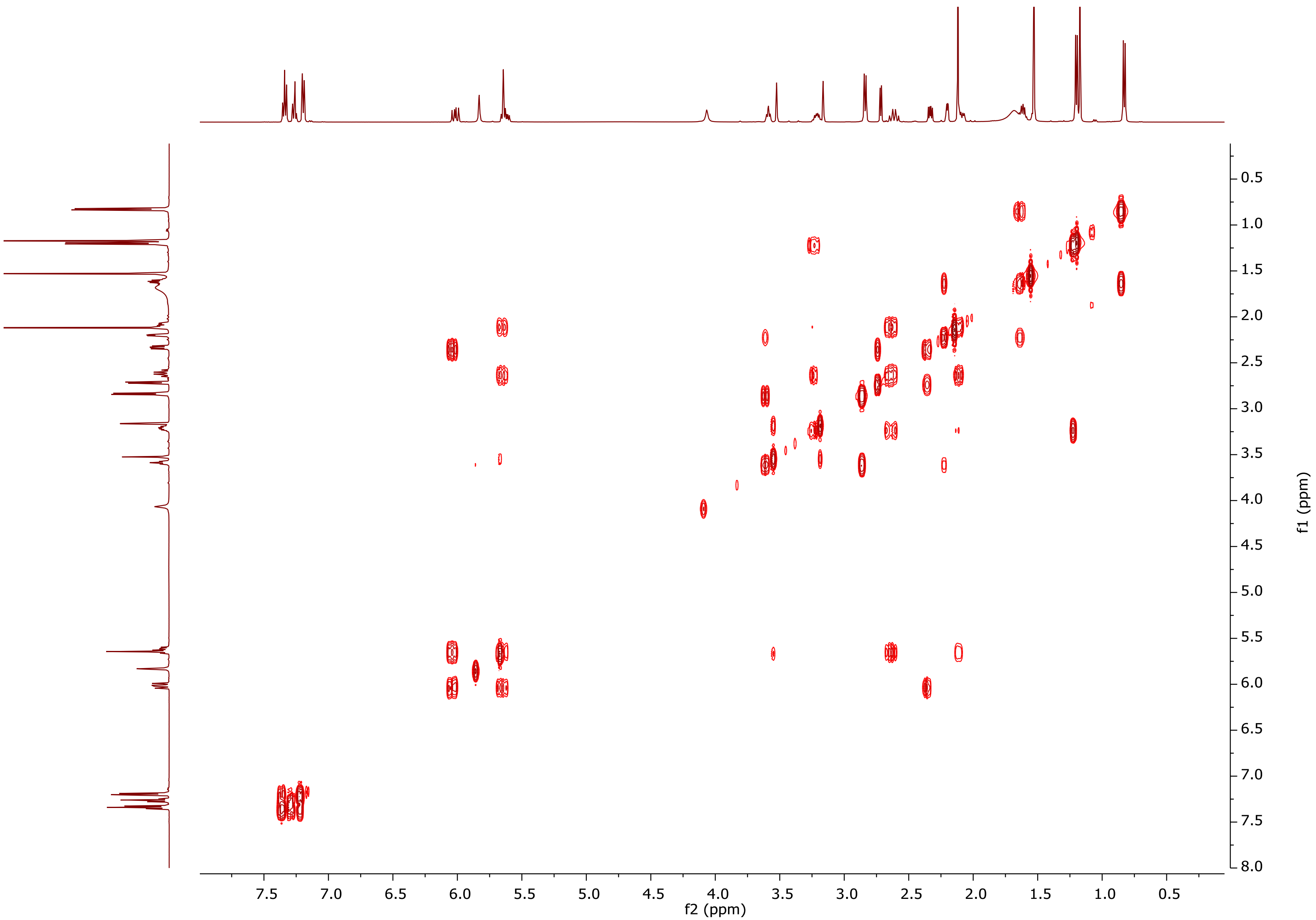

Figure SD46. ^1H-^1H COSY NMR spectrum of 19,20-epoxycytochalasin Q (**6**) (500/500 MHz, $CDCl_3$)

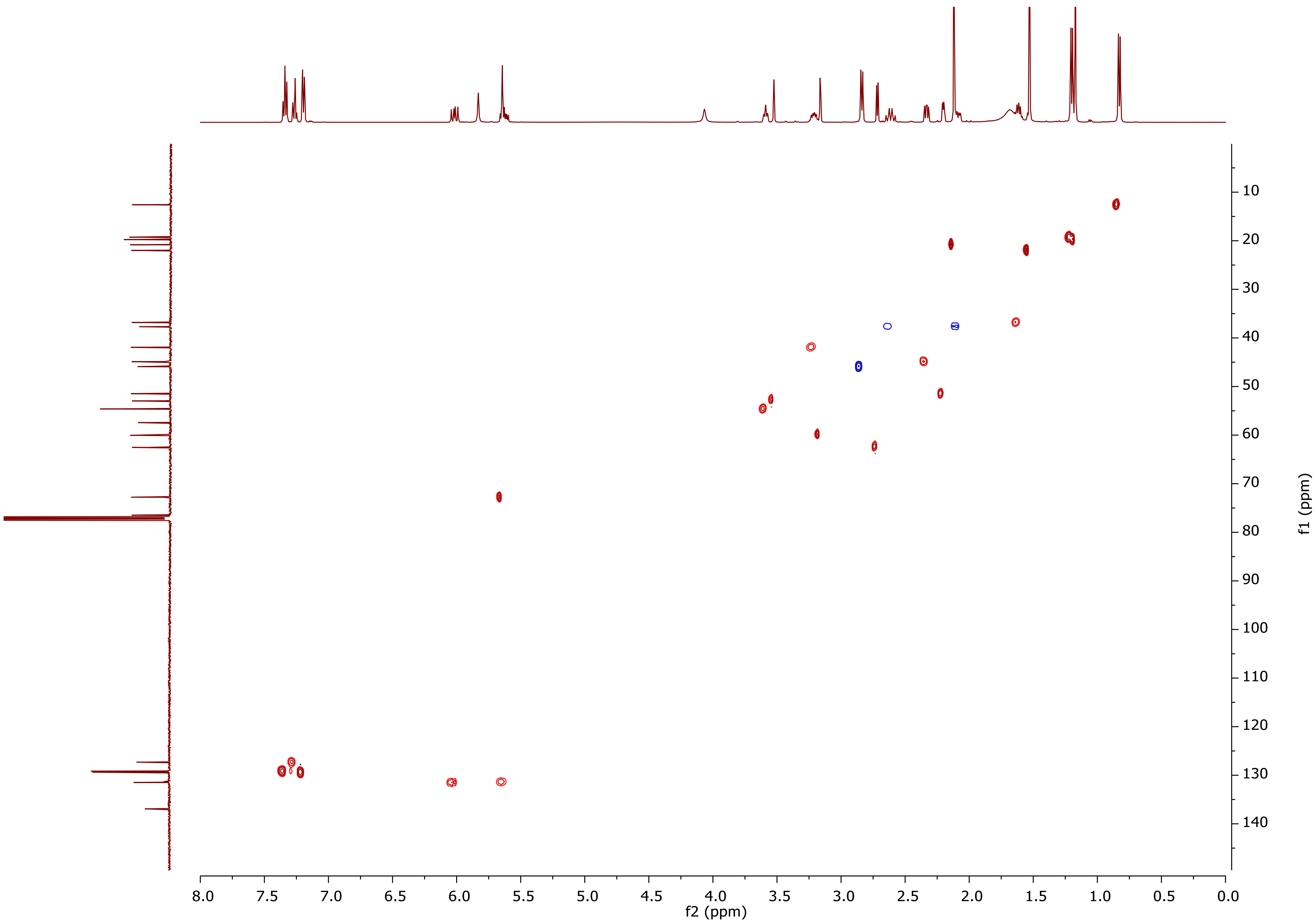

Figure SD47. ^{1}H-^{13}C HSQC NMR spectrum of 19,20-epoxycytochalasin Q (**6**) (500/125 MHz, $CDCl_3$)

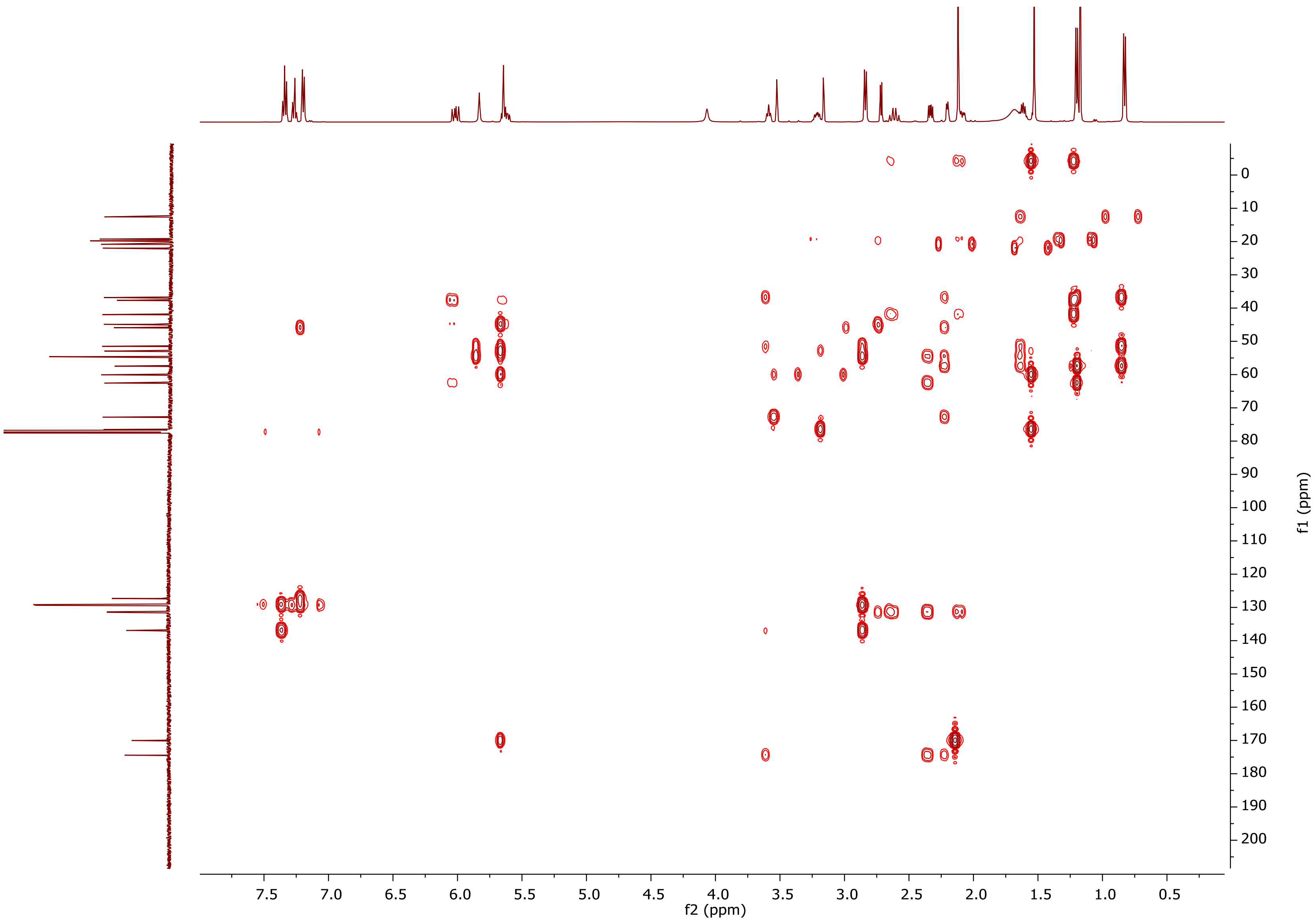

Figure SD48. ^{1}H-^{13}C HMBC NMR spectrum of 19,20-epoxycytochalasin Q (**6**) (500/125 MHz, $CDCl_3$)

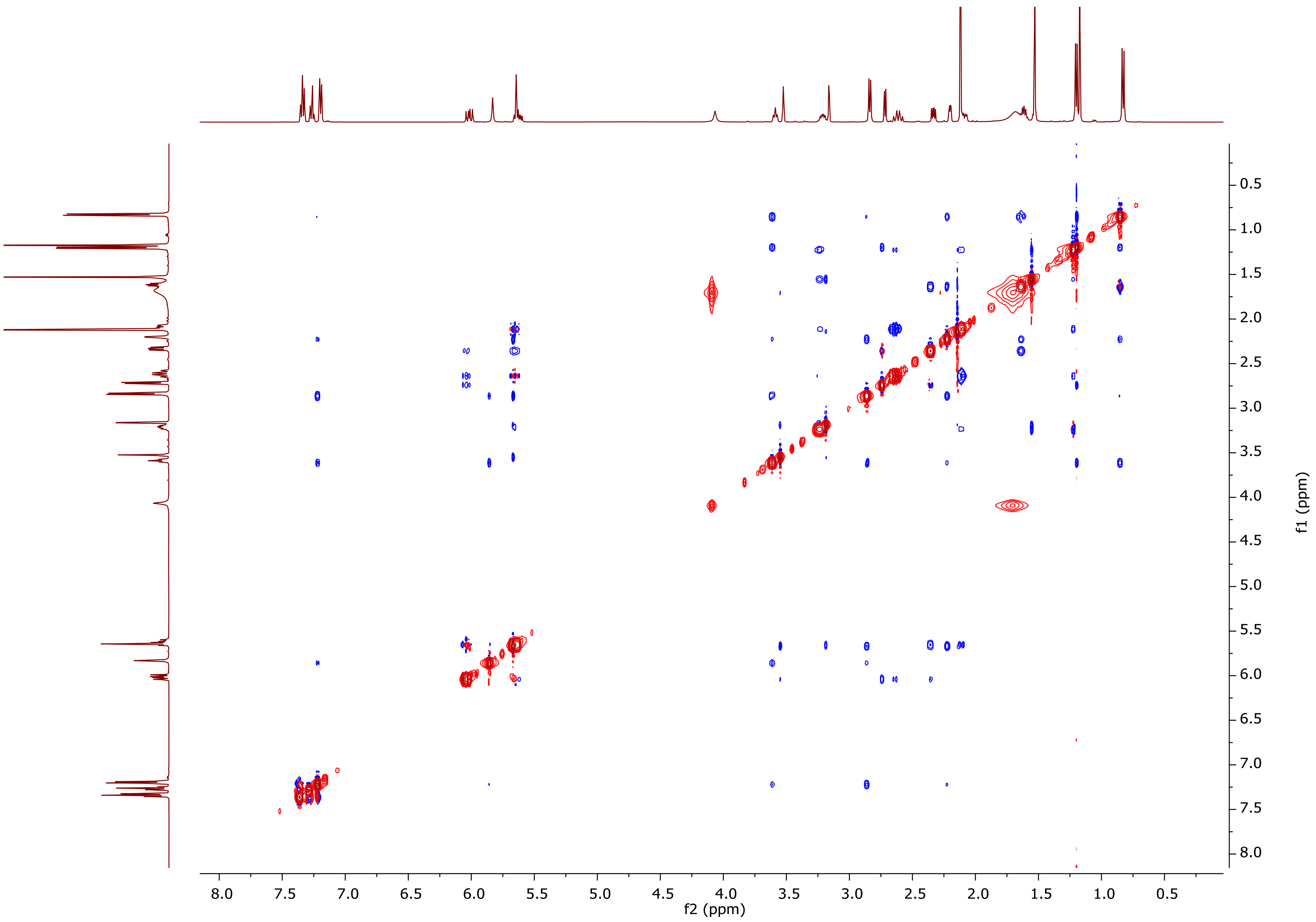

Figure SD49. ^{1}H-^{1}H NOESY NMR spectrum of 19,20-epoxycytochalasin Q (**6**) (500/500 MHz, $CDCl_3$)

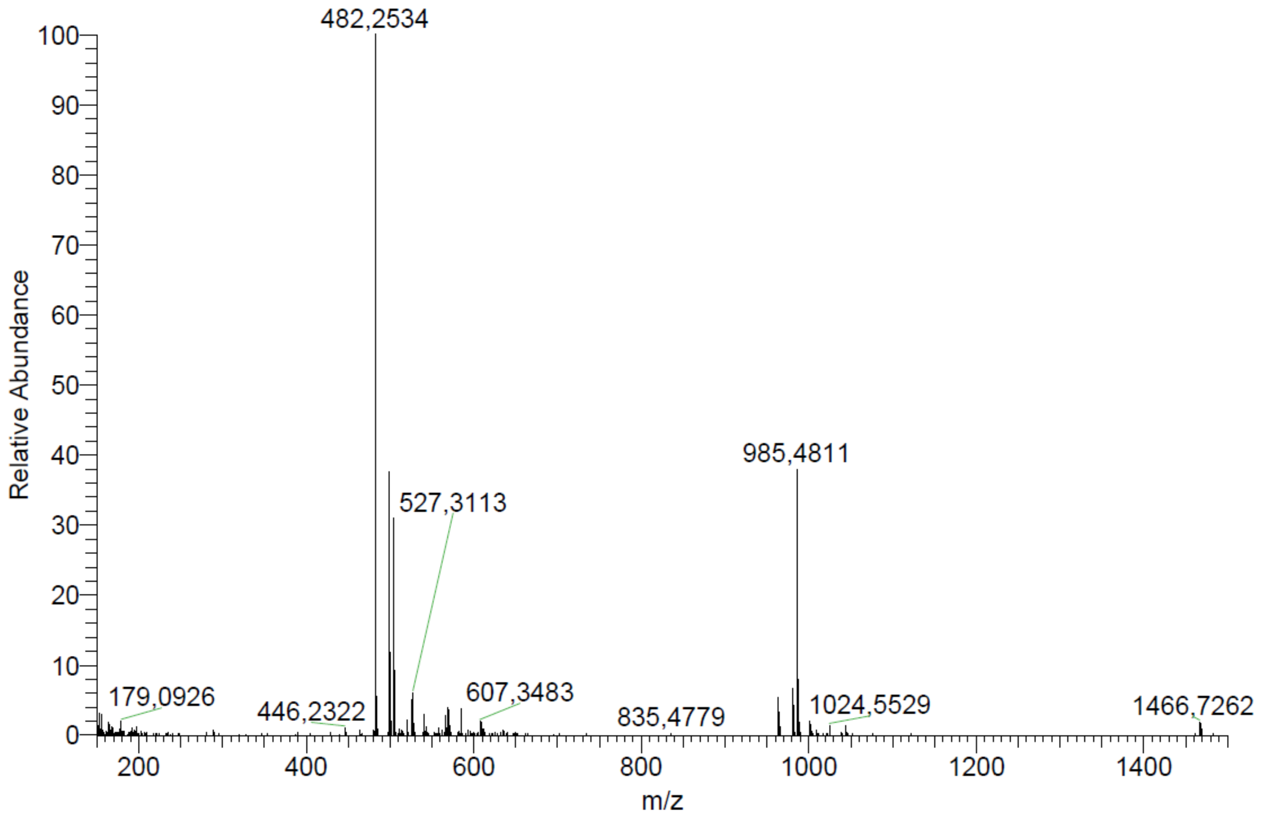

Figure SD50. ESI-HRMS spectrum of deacetyl-19,20-epoxycytochalasin Q (**7**)

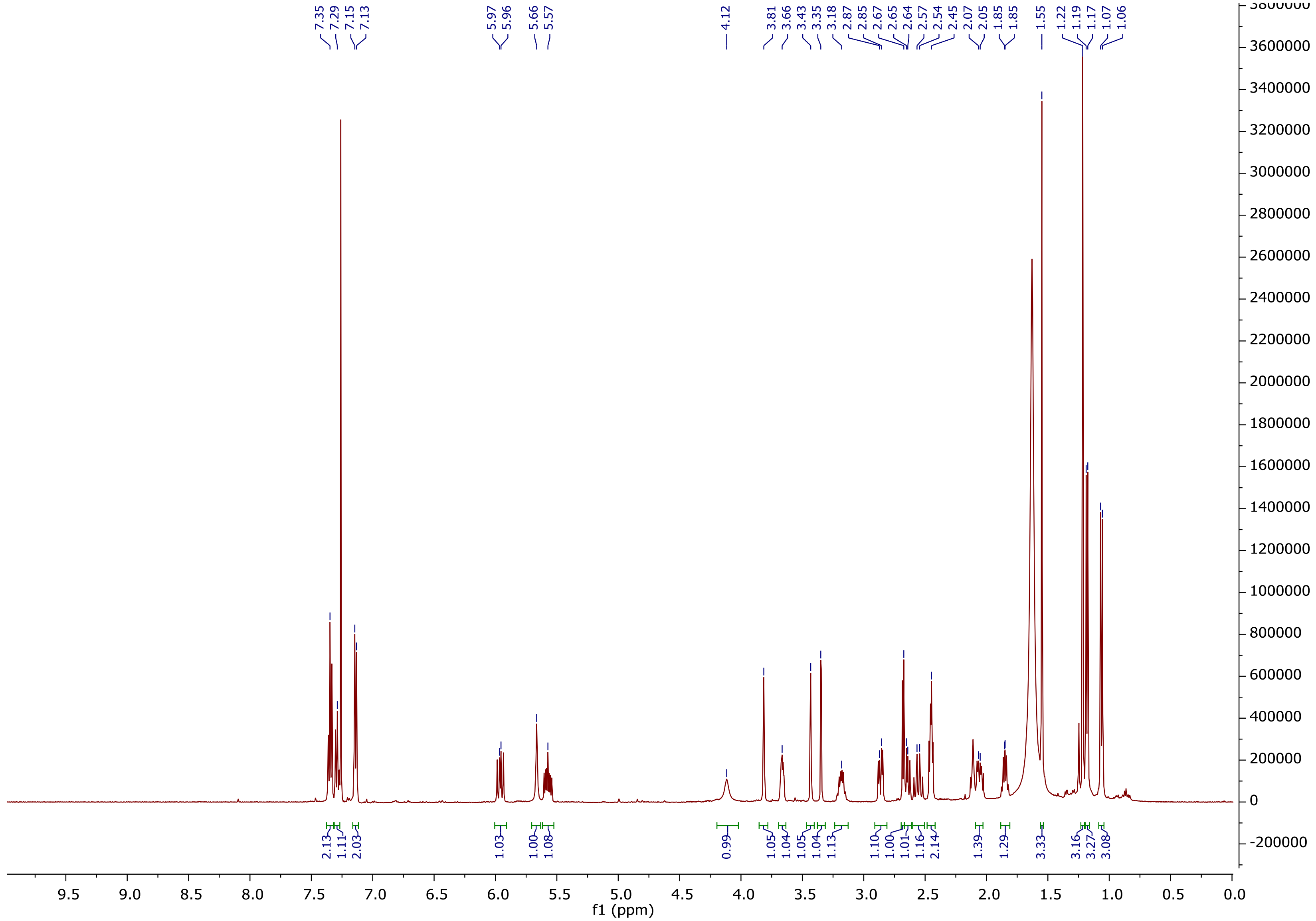

Figure SD51. ^{1}H NMR spectrum of deacetyl-19,20-epoxycytochalasin Q (**7**) (500 MHz, $CDCl_3$)

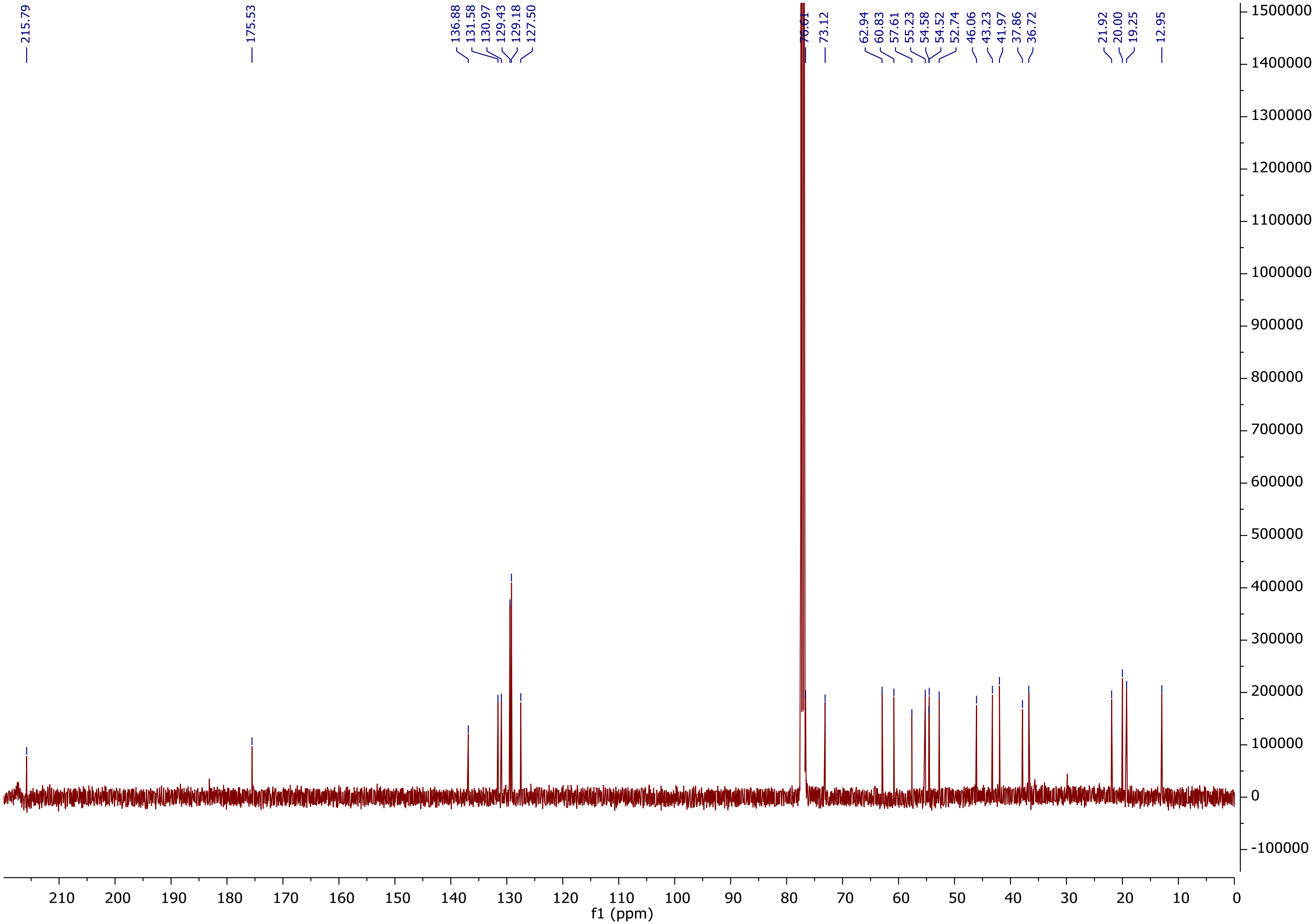

Figure SD52. ^{13}C NMR spectrum of deacetyl-19,20-epoxycytochalasin Q (**7**) (125 MHz, $CDCl_3$)

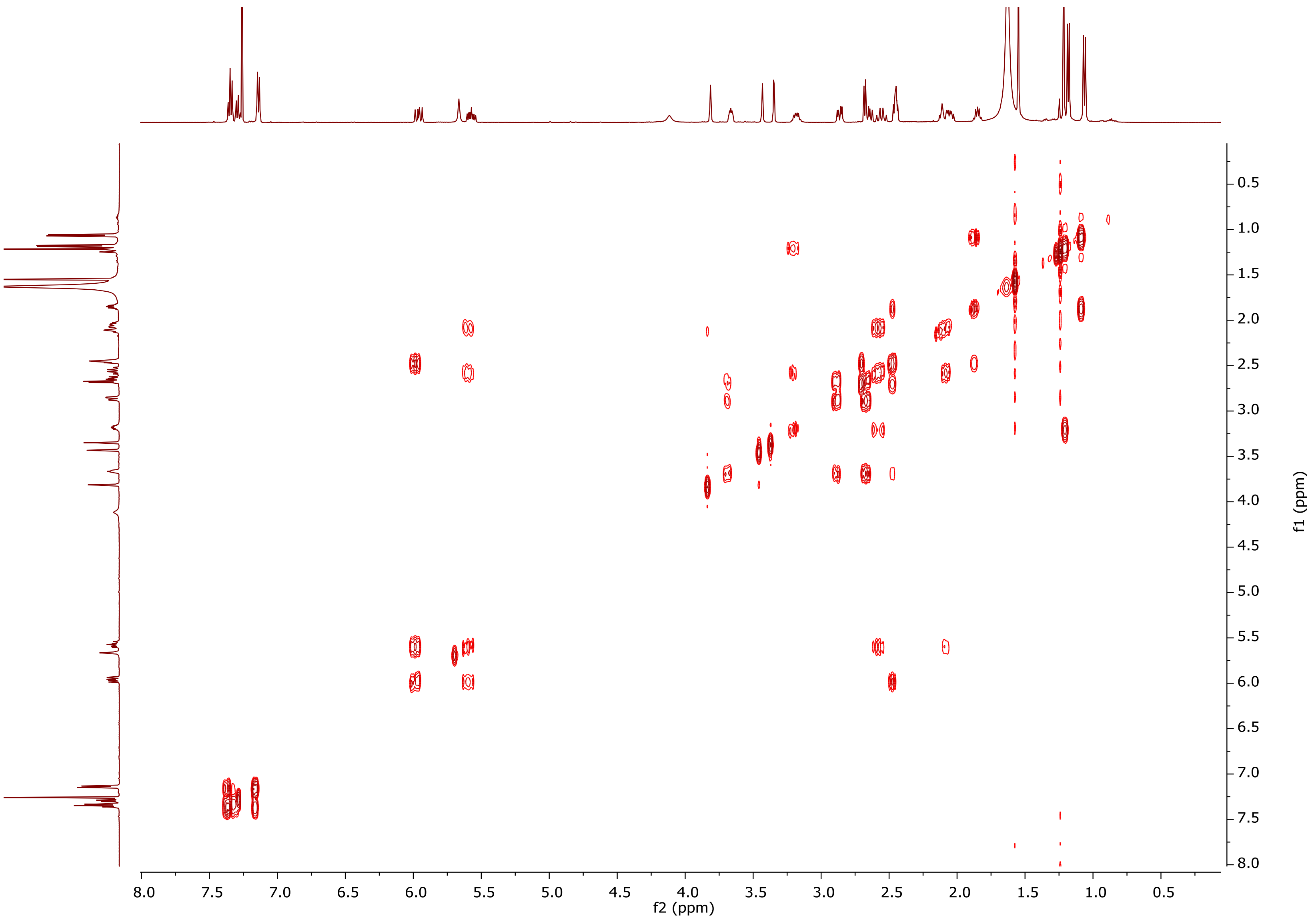

Figure SD53. ^{1}H-^{1}H COSY NMR spectrum of deacetyl-19,20-epoxycytochalasin Q (**7**) (500/500 MHz, $CDCl_3$)

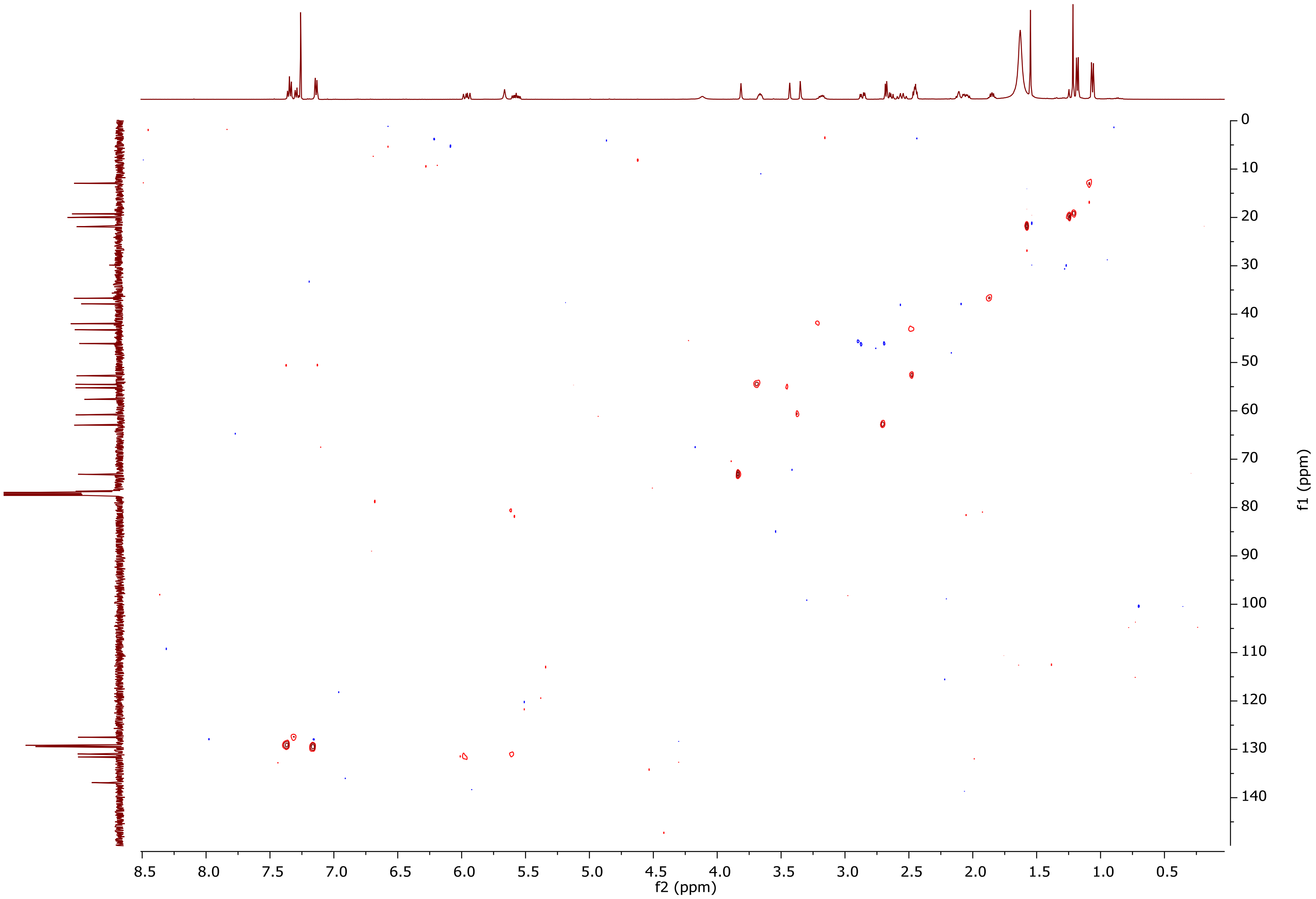

Figure SD54. ^{1}H-^{13}C HSQC NMR spectrum of deacetyl-19,20-epoxycytochalasin Q (**7**) (500/125 MHz, $CDCl_3$)

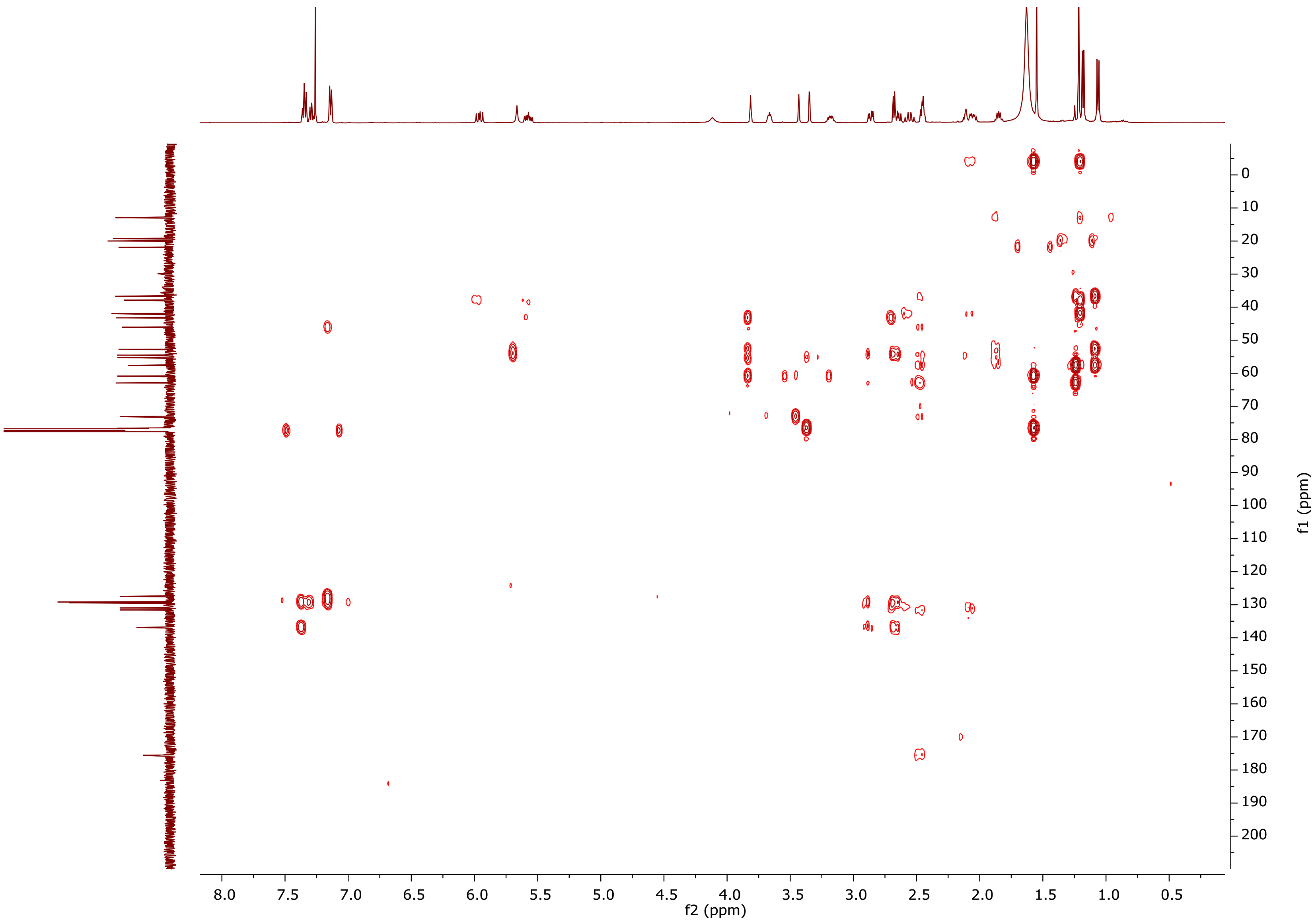

Figure SD55. ^{1}H-^{13}C HMBC NMR spectrum of deacetyl-19,20-epoxycytochalasin Q (**7**) (500/125 MHz, $CDCl_3$)

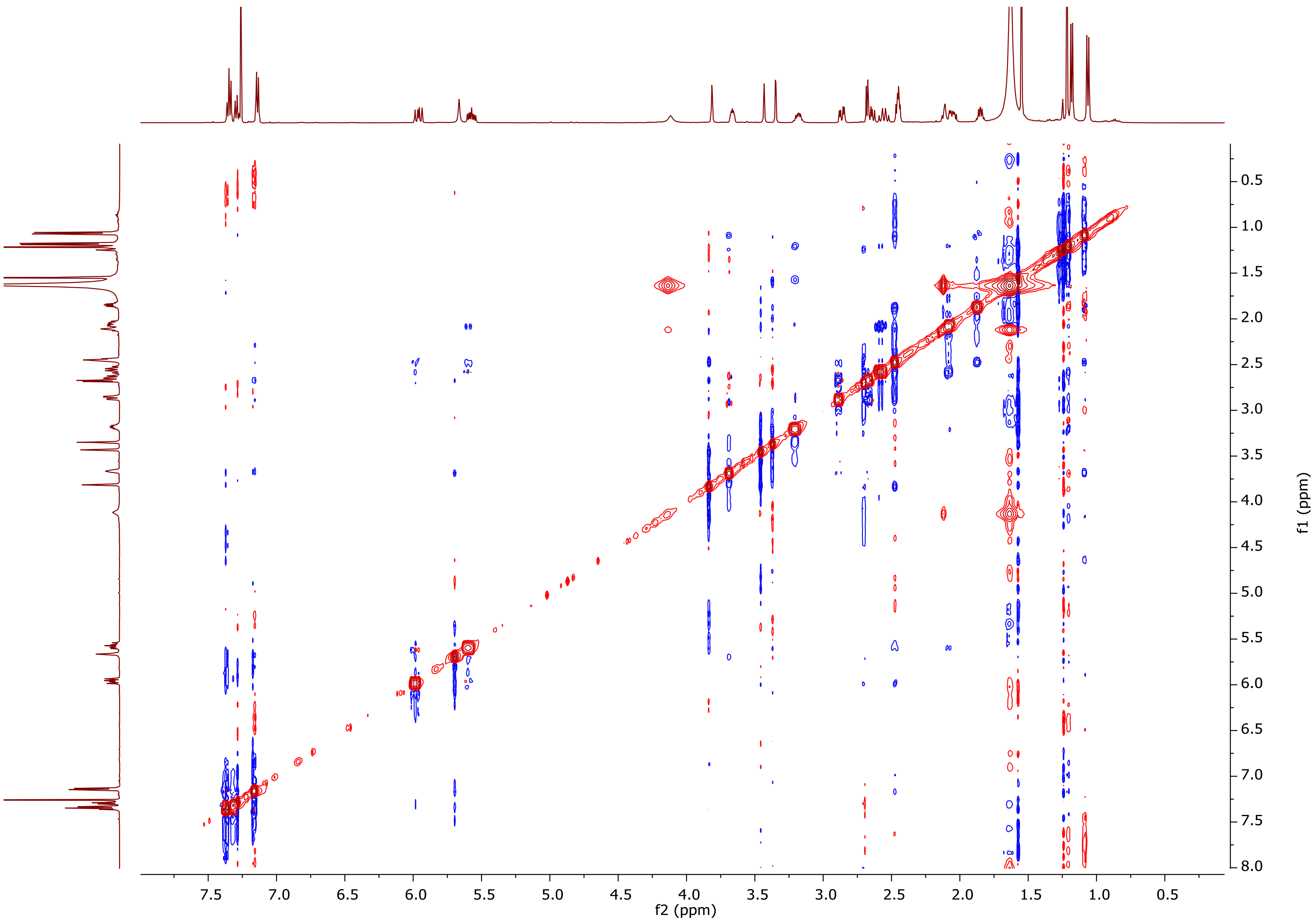

Figure SD56. ^1H-^1H NOESY NMR spectrum of deacetyl-19,20-epoxycytochalasin Q (**7**) (500/500 MHz, $CDCl_3$)

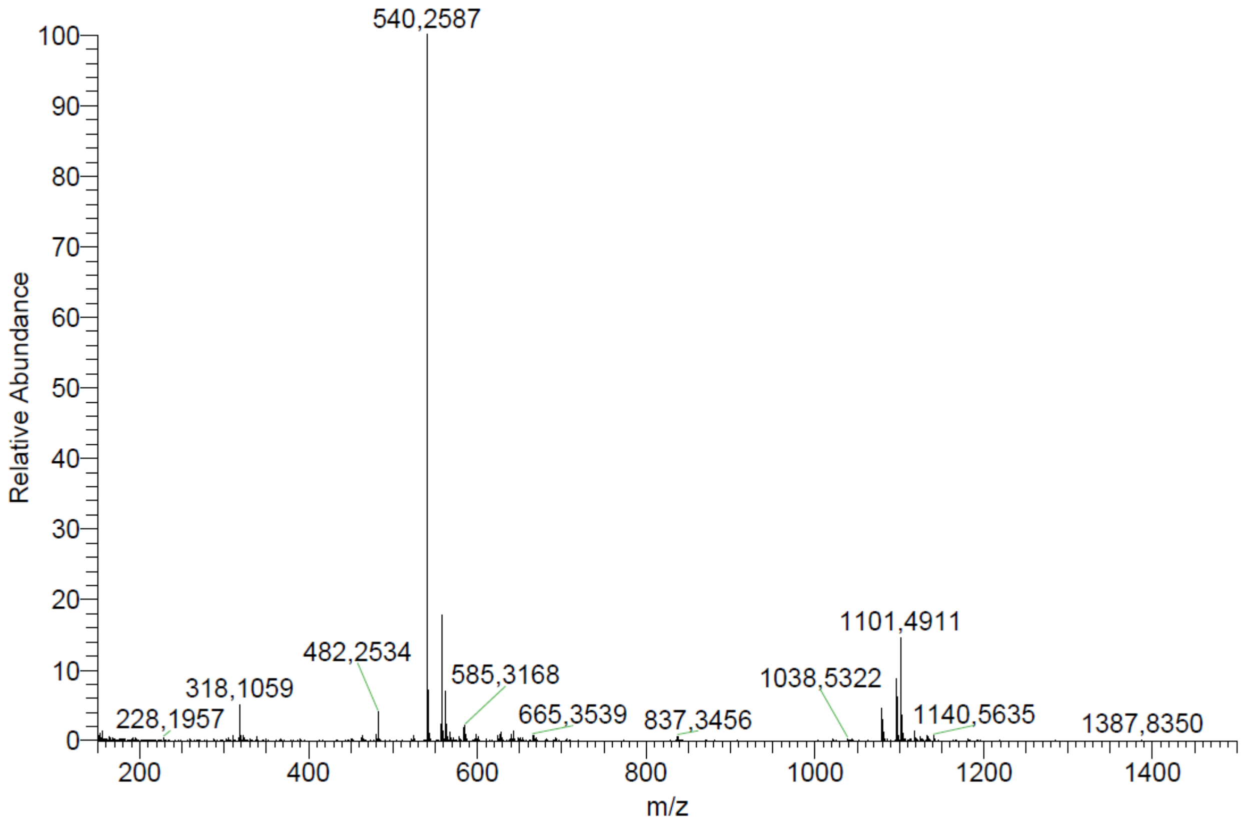

Figure SD57. ESI-HRMS spectrum of 19,20-epoxycytochalasin R (**8**)

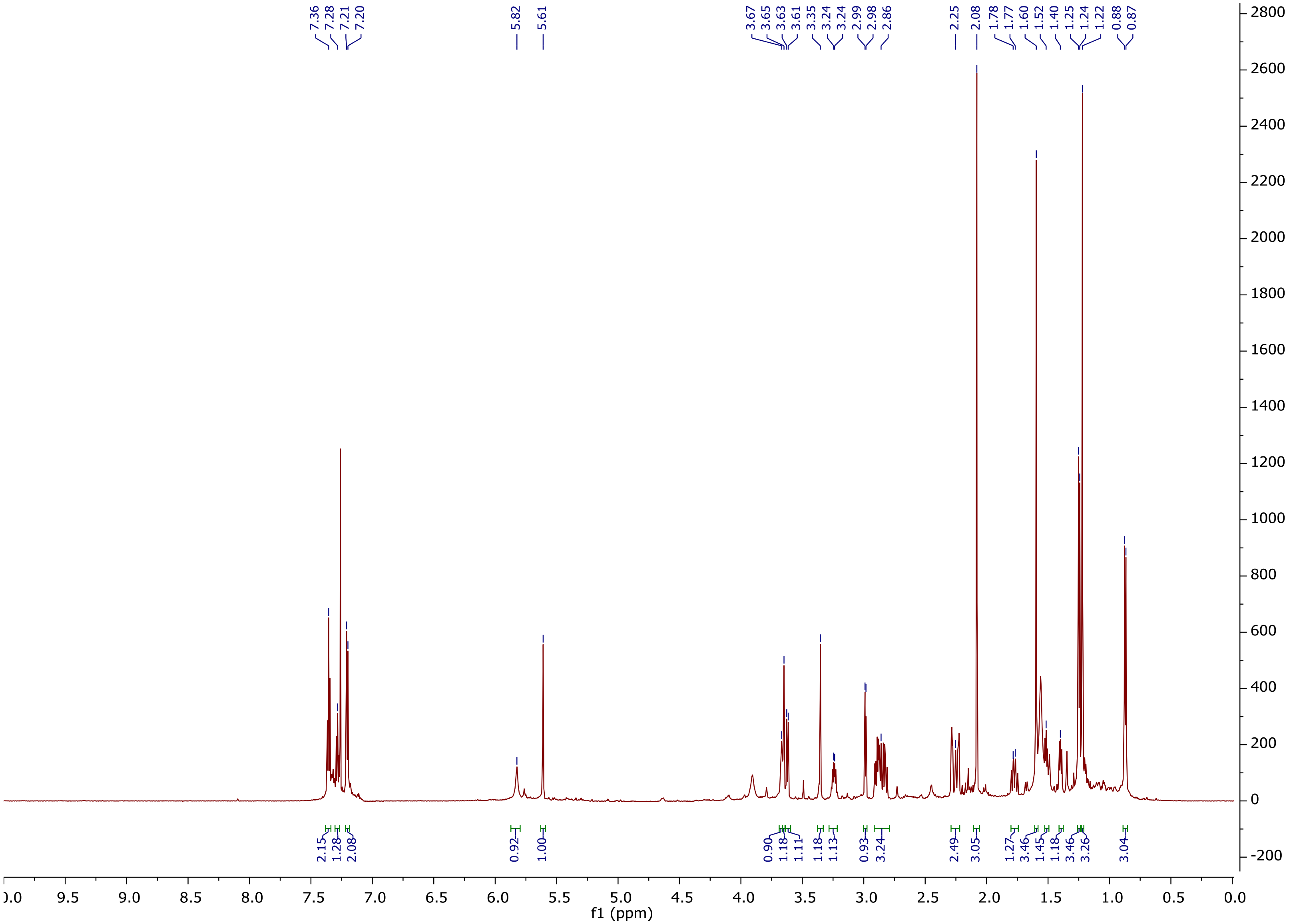

Figure SD58. ^{1}H NMR spectrum of 19,20-epoxycytochalasin R (**8**) (700 MHz, $CDCl_3$)

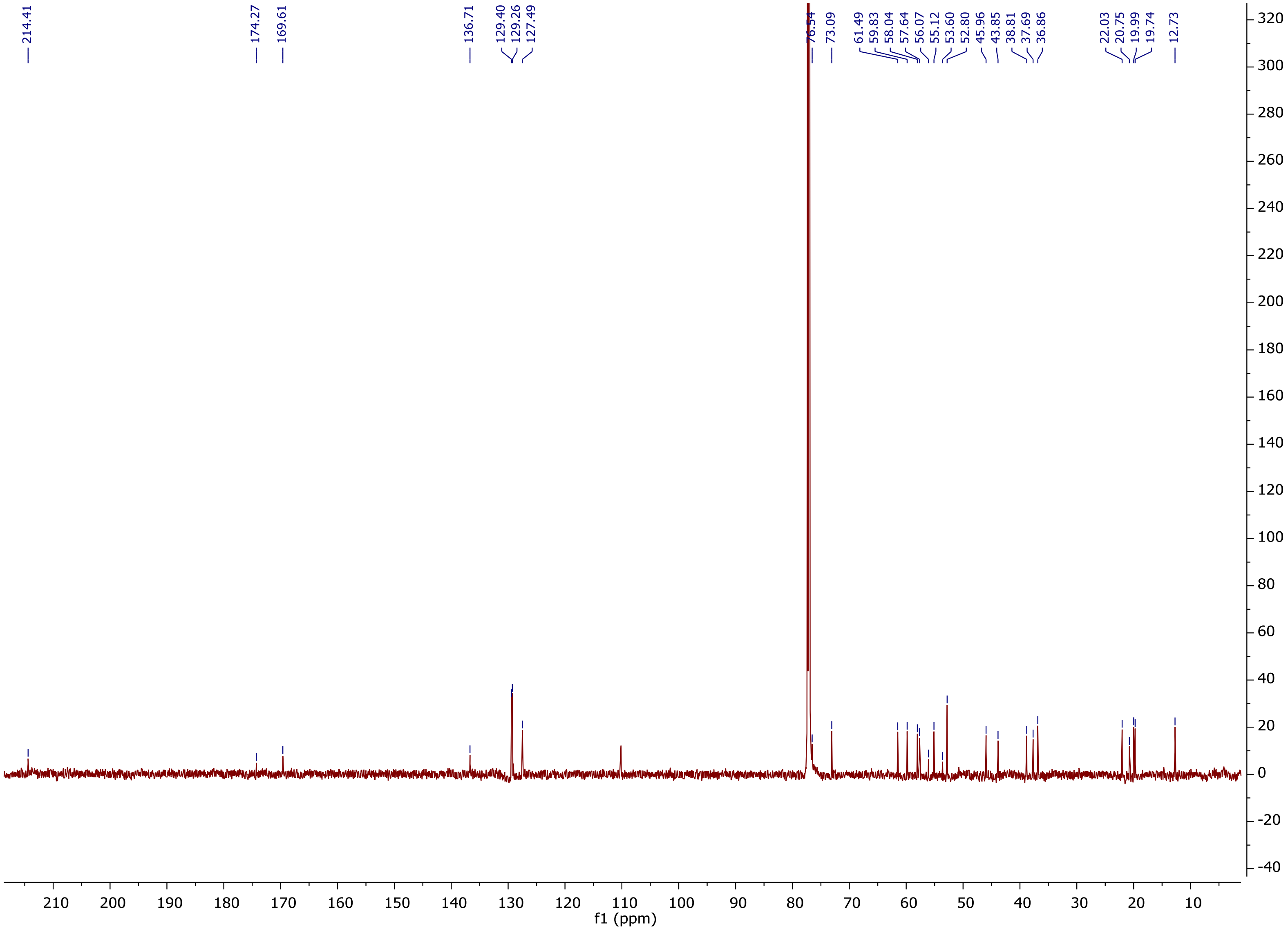

Figure SD59. ^{13}C NMR spectrum of 19,20-epoxycytochalasin R (**8**) (175 MHz, $CDCl_3$)

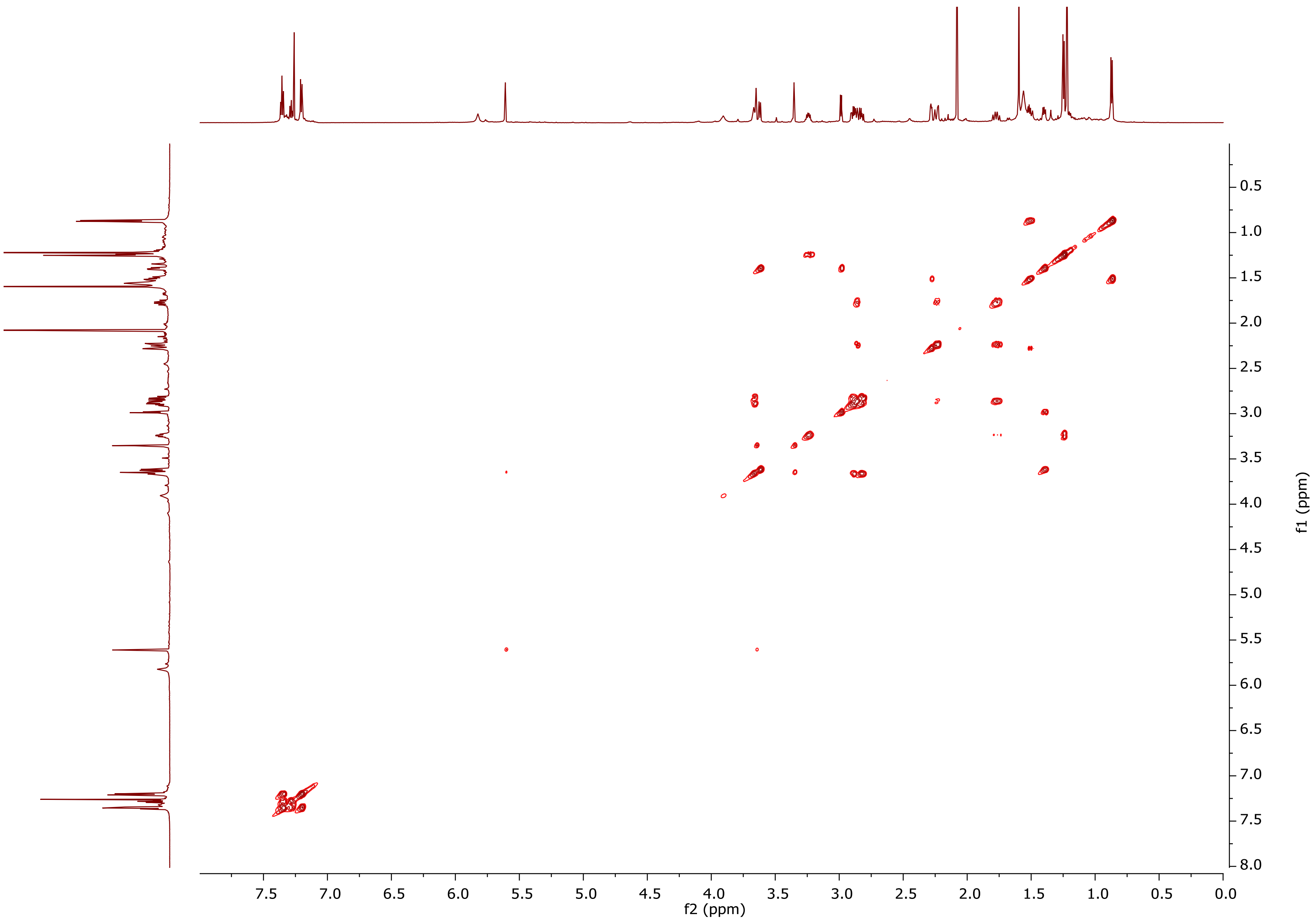

Figure SD60. ^{1}H-^{1}H COSY NMR spectrum of 19,20-epoxycytochalasin R (**8**) (700/700 MHz, $CDCl_3$)

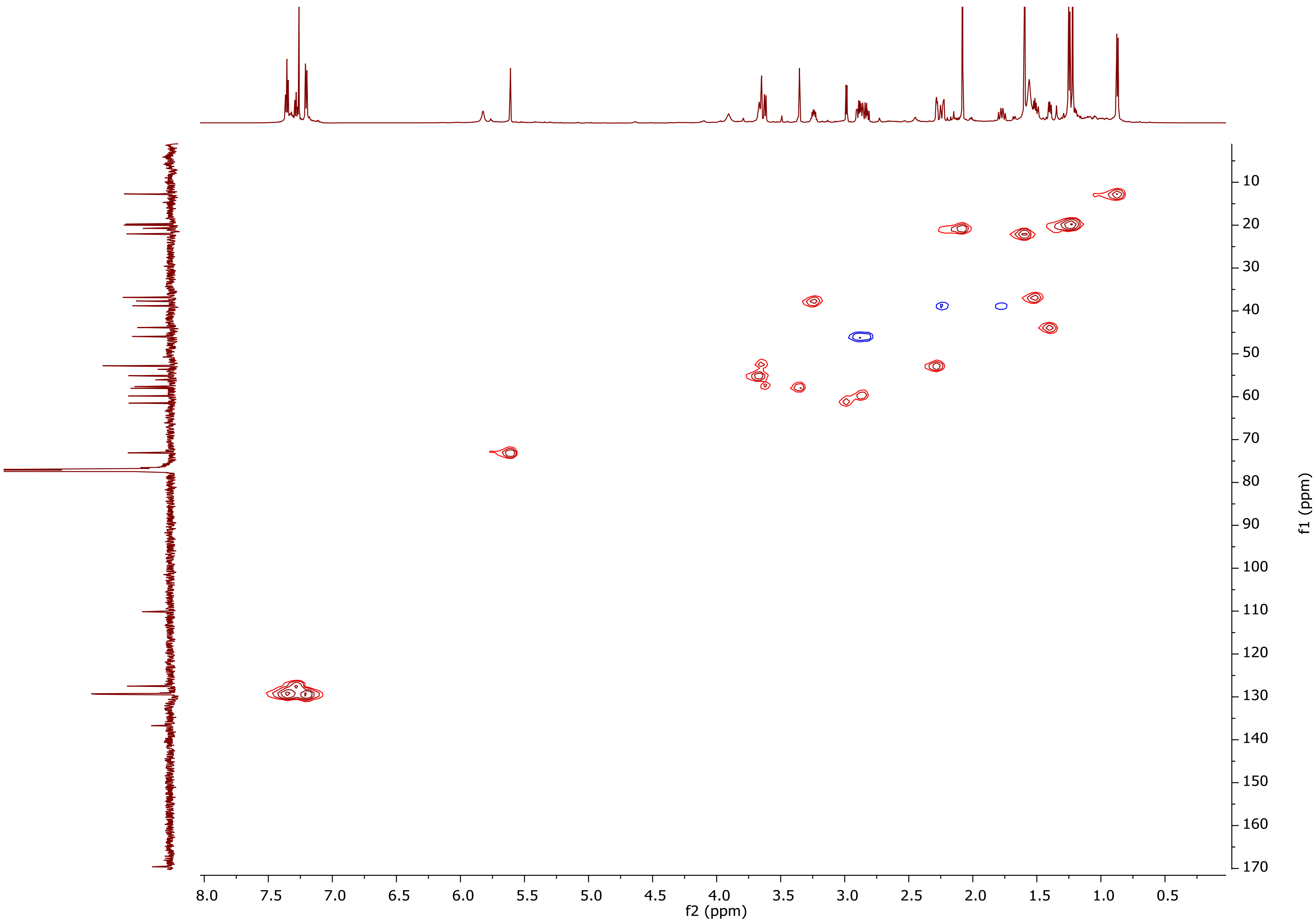

Figure SD61. ^{1}H-^{13}C HSQC NMR spectrum of 19,20-epoxycytochalasin R (**8**) (700/175 MHz, $CDCl_3$)

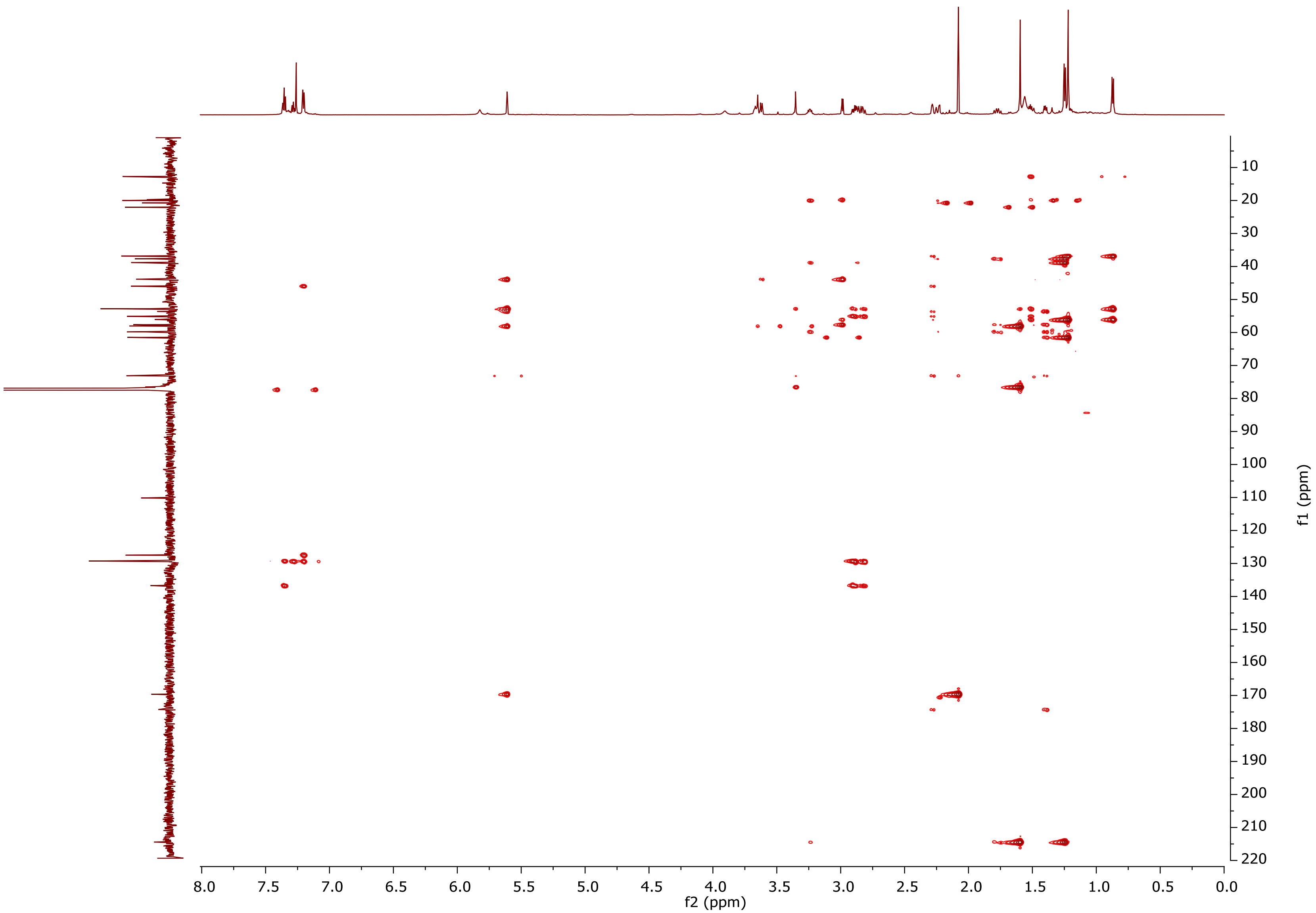

Figure SD62. ^{1}H-^{13}C HMBC NMR spectrum of 19,20-epoxycytochalasin R (**8**) (700/175 MHz, $CDCl_3$)

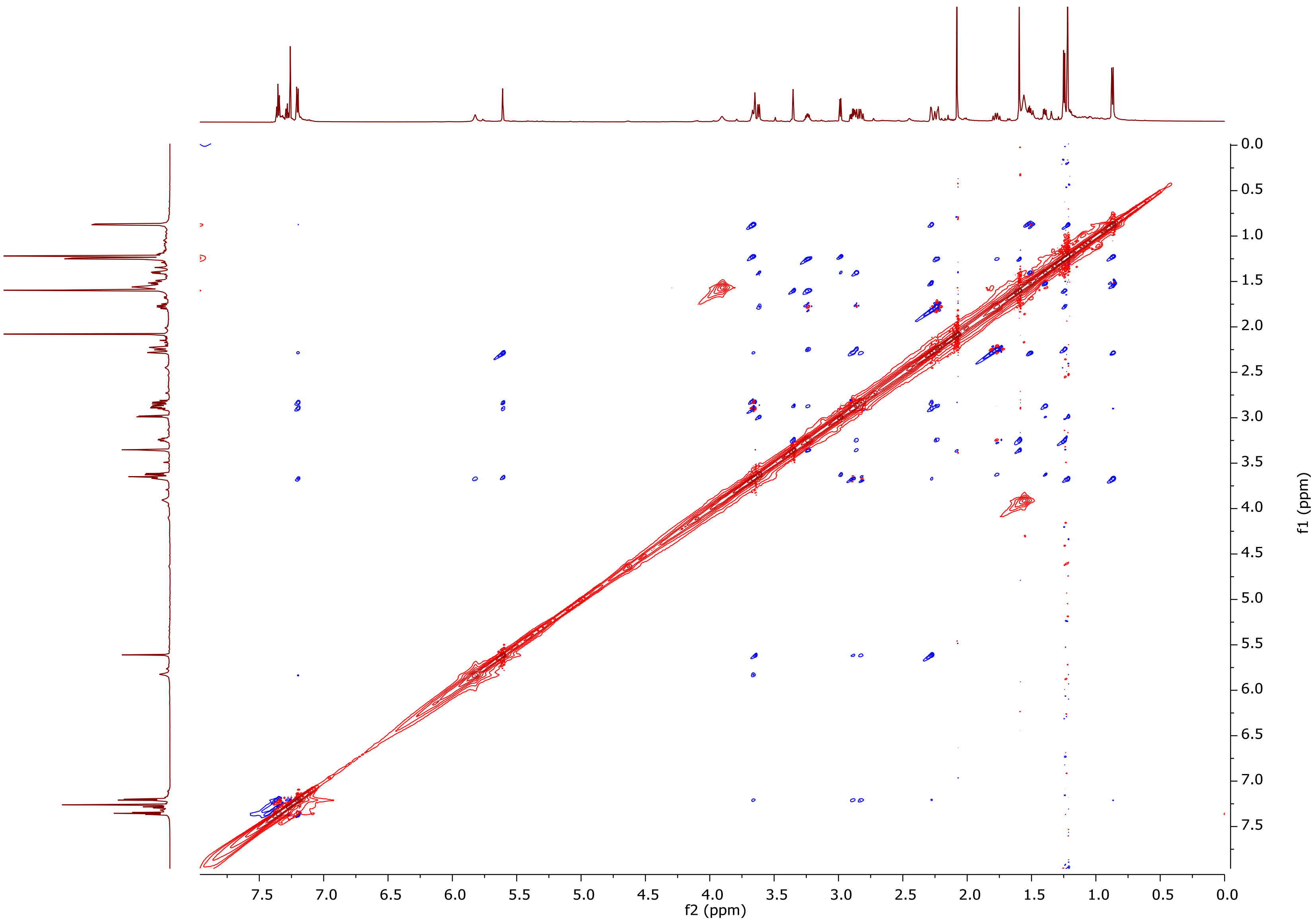

Figure SD63. ^{1}H-^{1}H NOESY NMR spectrum of 19,20-epoxycytochalasin R (**8**) (700/700 MHz, $CDCl_3$)

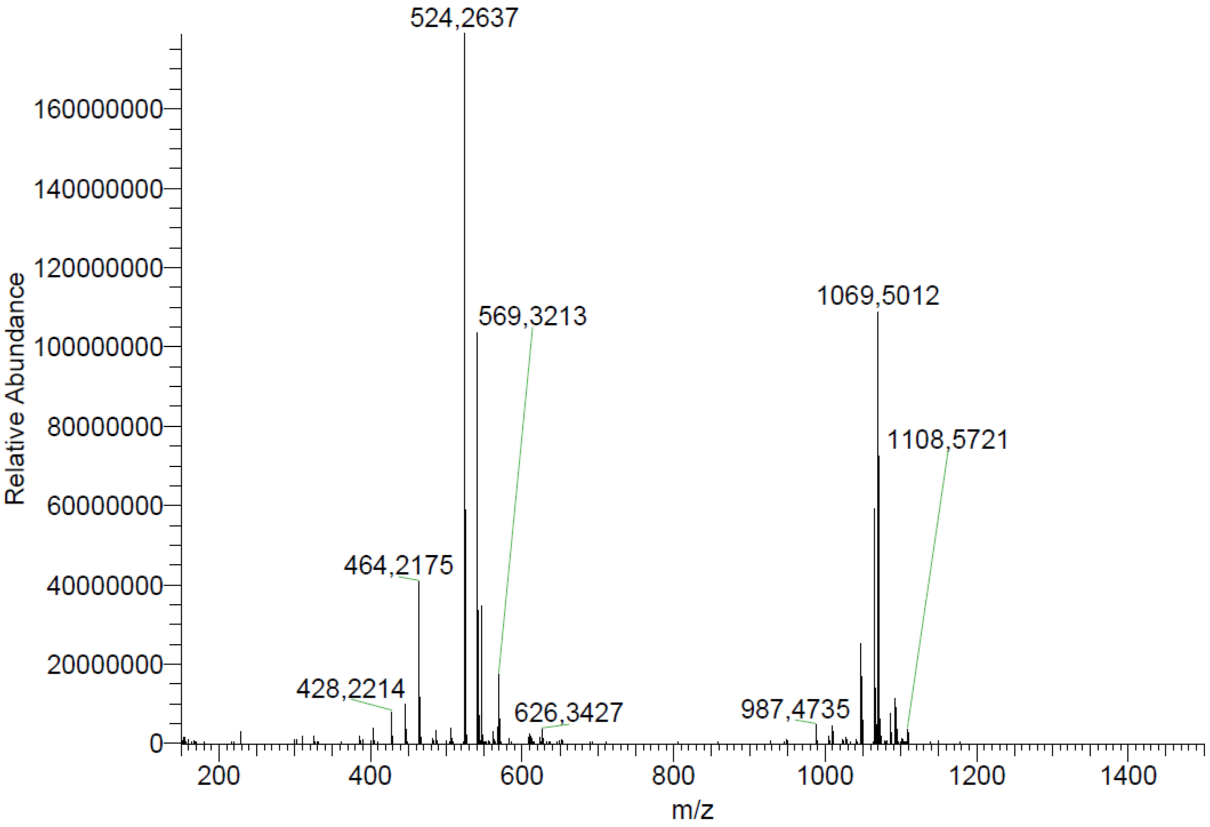

Figure SD64. ESI-HRMS spectrum of 19,20-epoxycytochalasin D (**9**)

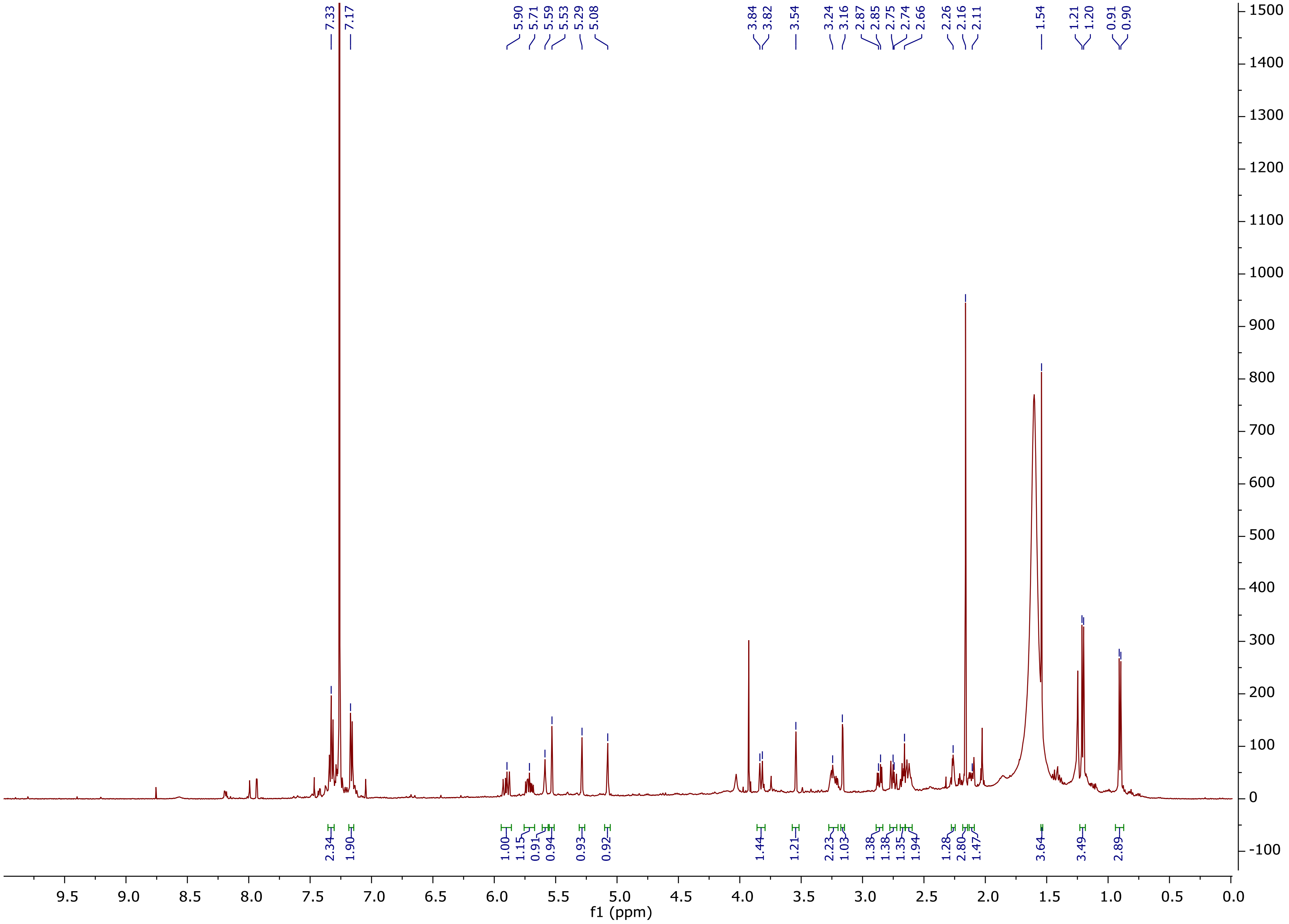

Figure SD65. ^{1}H NMR spectrum of 19,20-epoxycytochalasin D (**9**) (500 MHz, $CDCl_3$)

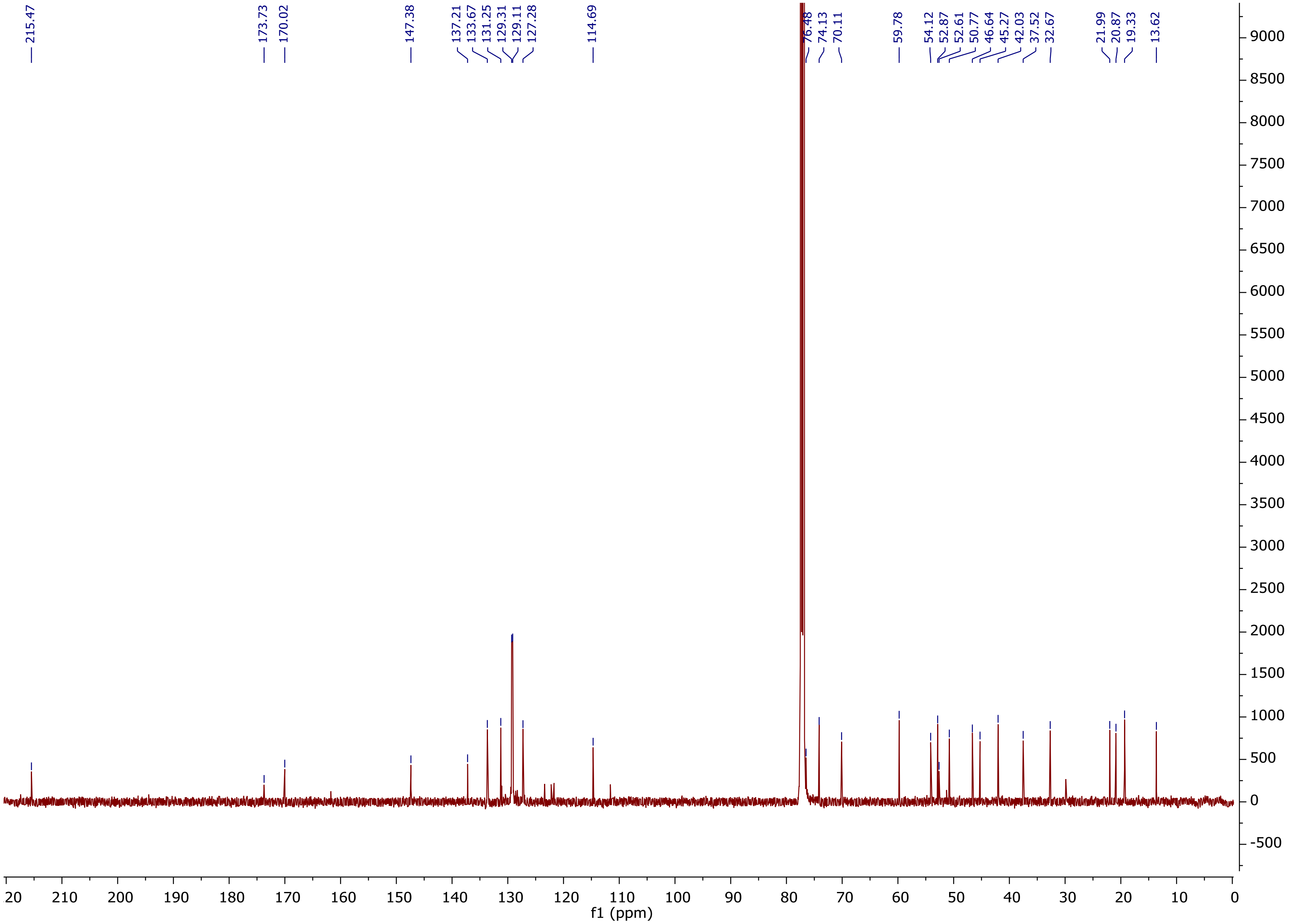

Figure SD66. ^{13}C NMR spectrum of 19,20-epoxycytochalasin D (**9**) (125 MHz, $CDCl_3$)

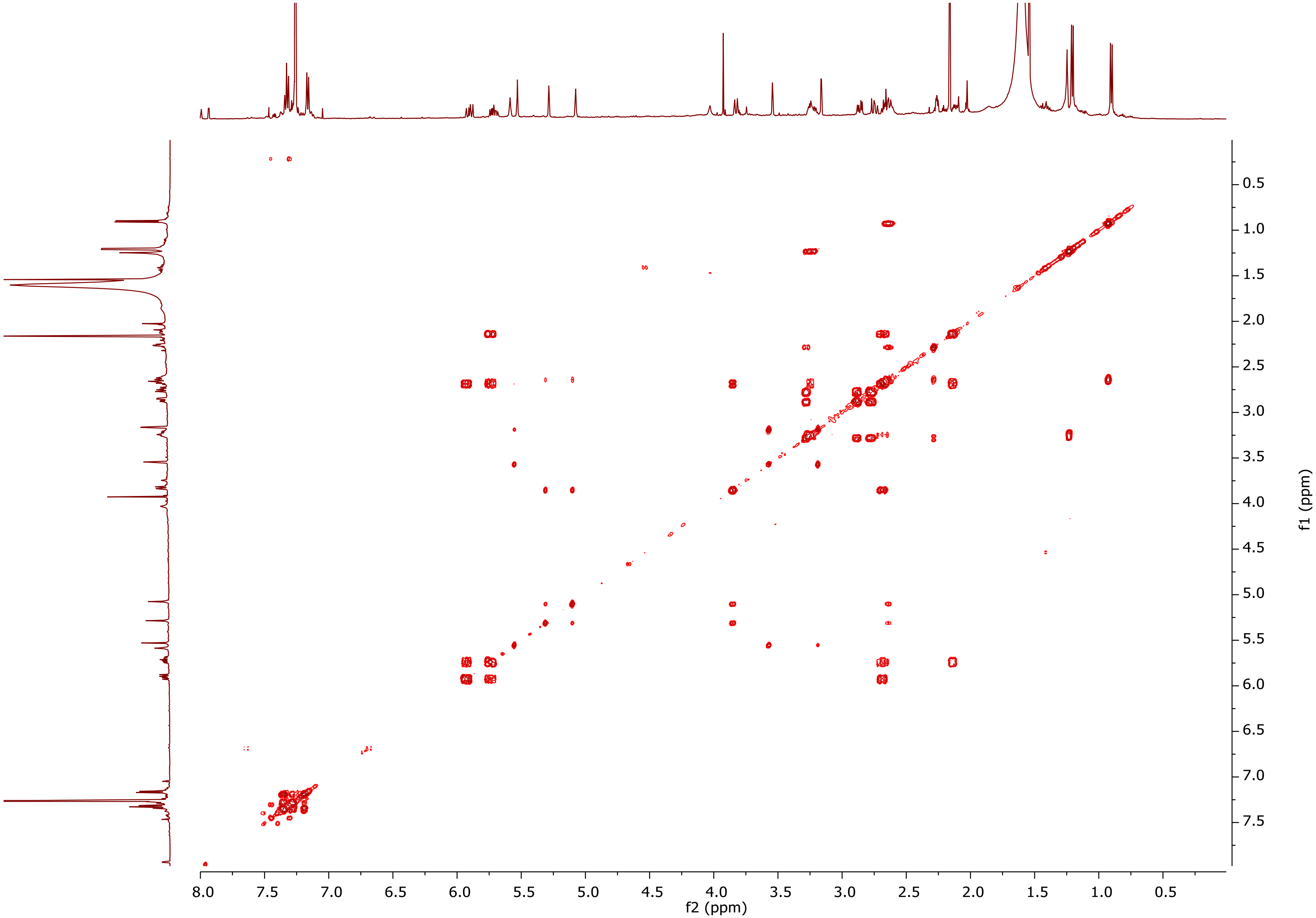

Figure SD67. ^{1}H-^{1}H COSY NMR spectrum of 19,20-epoxycytochalasin D (**9**) (500/500 MHz, $CDCl_3$)

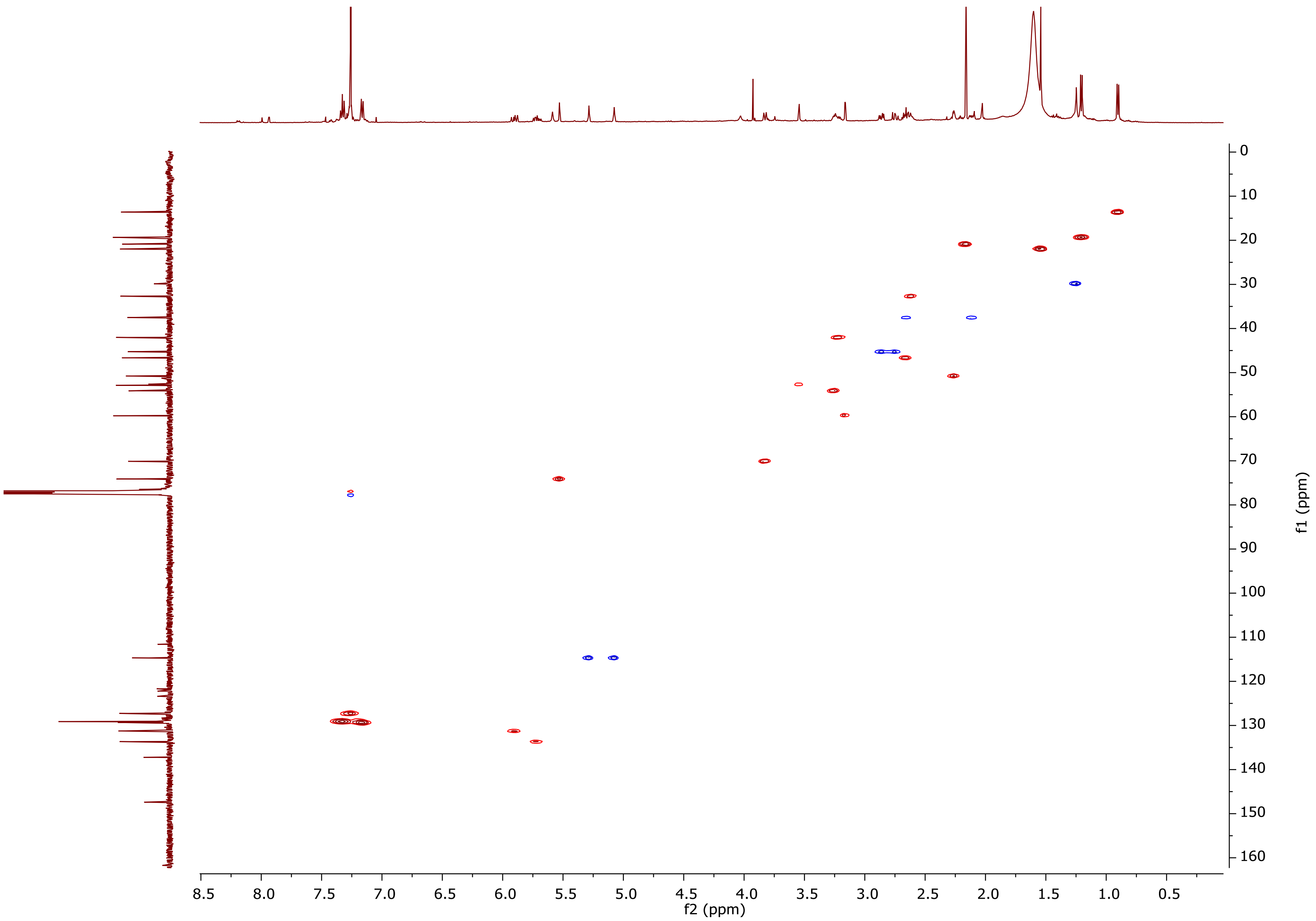

Figure SD68. ^{1}H-^{13}C HSQC NMR spectrum of 19,20-epoxycytochalasin D (**9**) (500/125 MHz, $CDCl_3$)

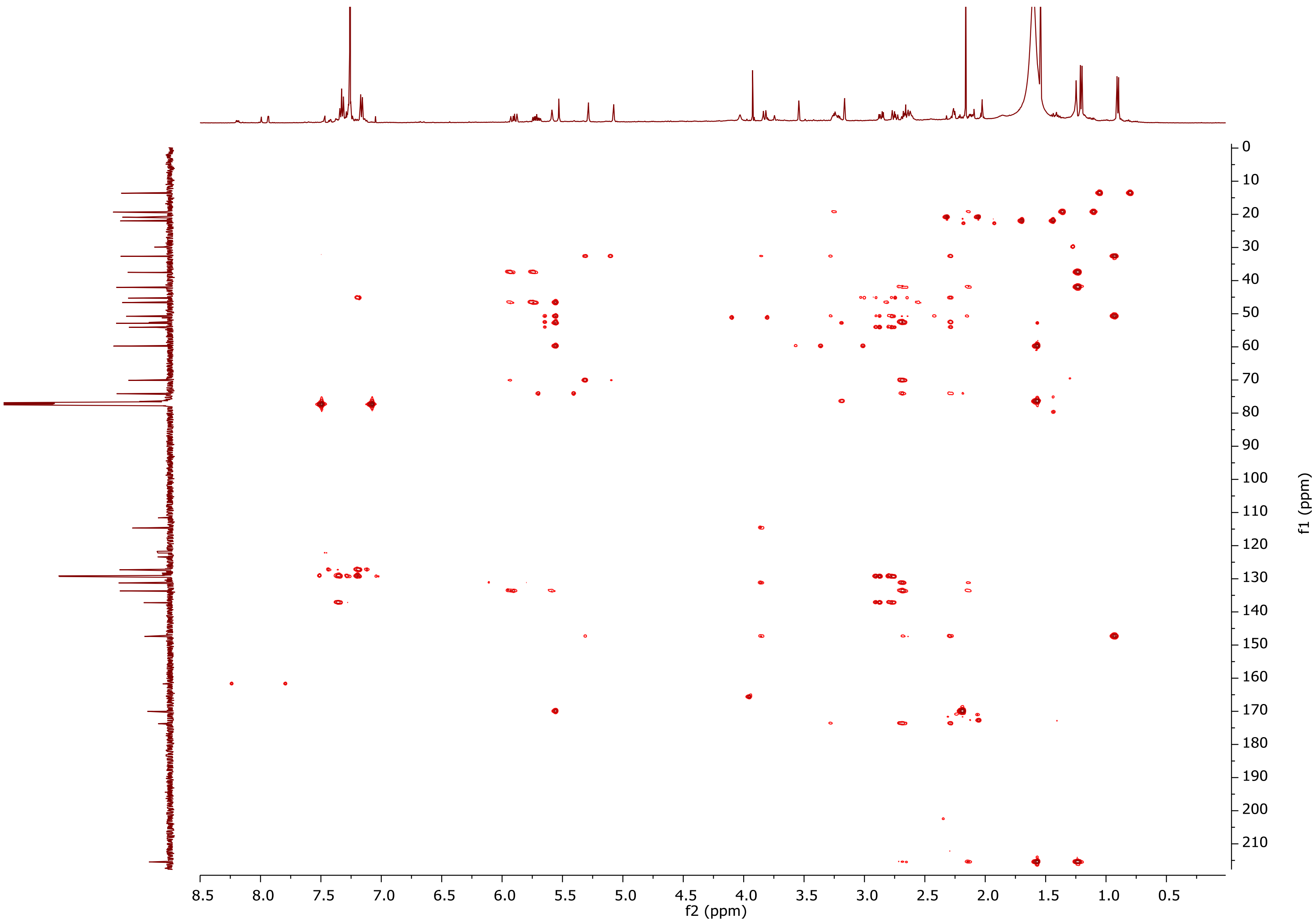

Figure SD69. ^{1}H-^{13}C HMBC NMR spectrum of 19,20-epoxycytochalasin D (**9**) (500/125 MHz, $CDCl_3$)

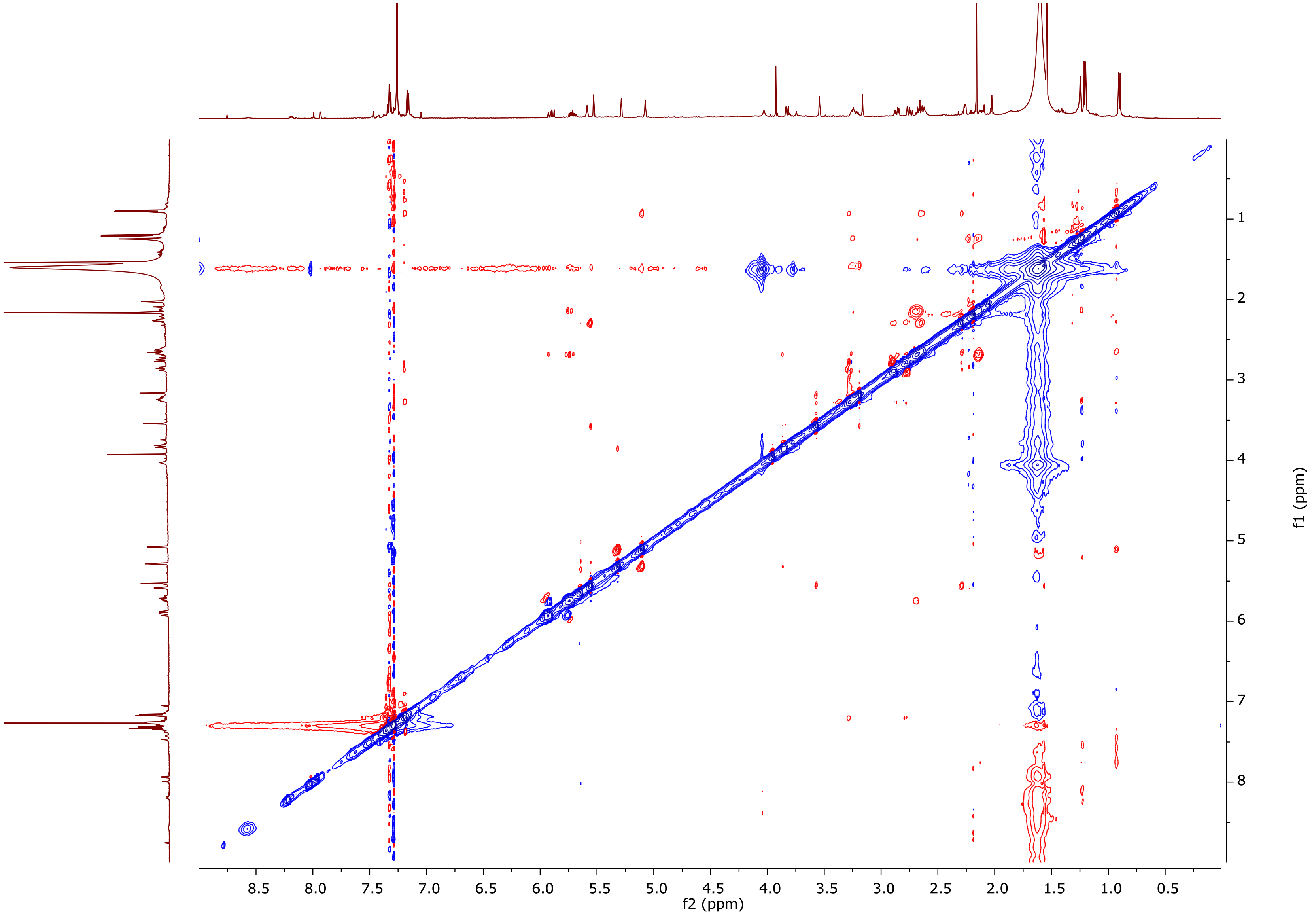

Figure SD70. ^{1}H-^{1}H NOESY NMR spectrum of 19,20-epoxycytochalasin D (**9**) (500/500 MHz, $CDCl_3$)

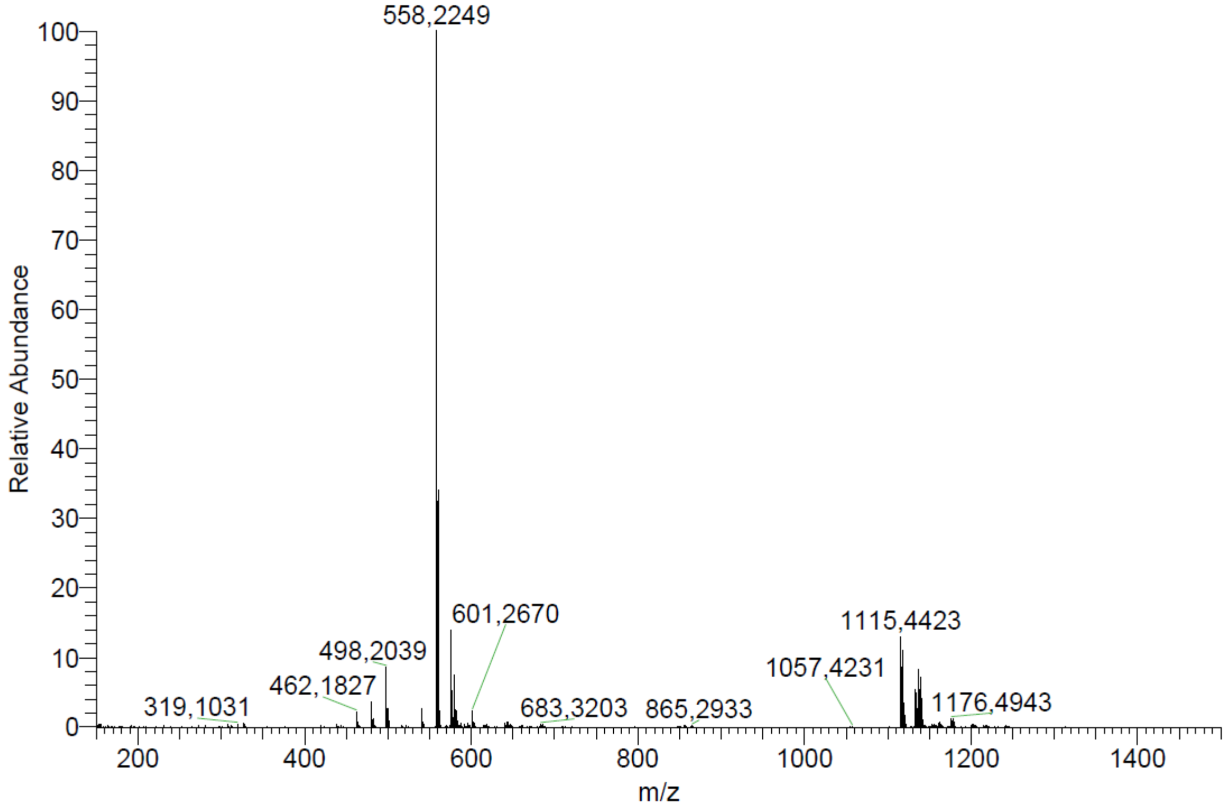

Figure SD71. ESI-HRMS spectrum of *m*-chloro-19,20-epoxycytochalasin C (**11**)

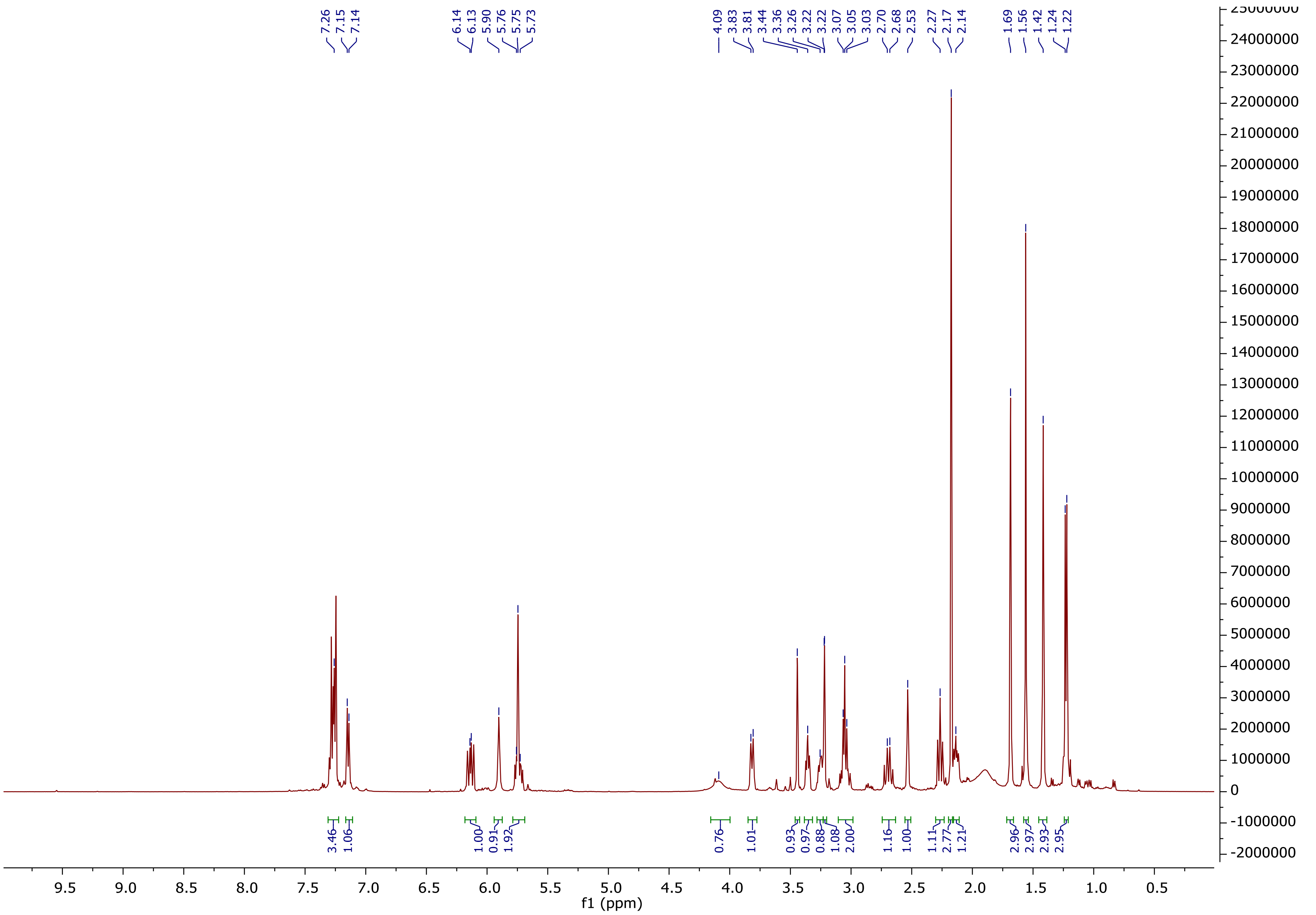

Figure SD72. ^{1}H NMR spectrum of *m*-chloro-19,20-epoxycytochalasin C (**11**) (500 MHz, $CDCl_3$)

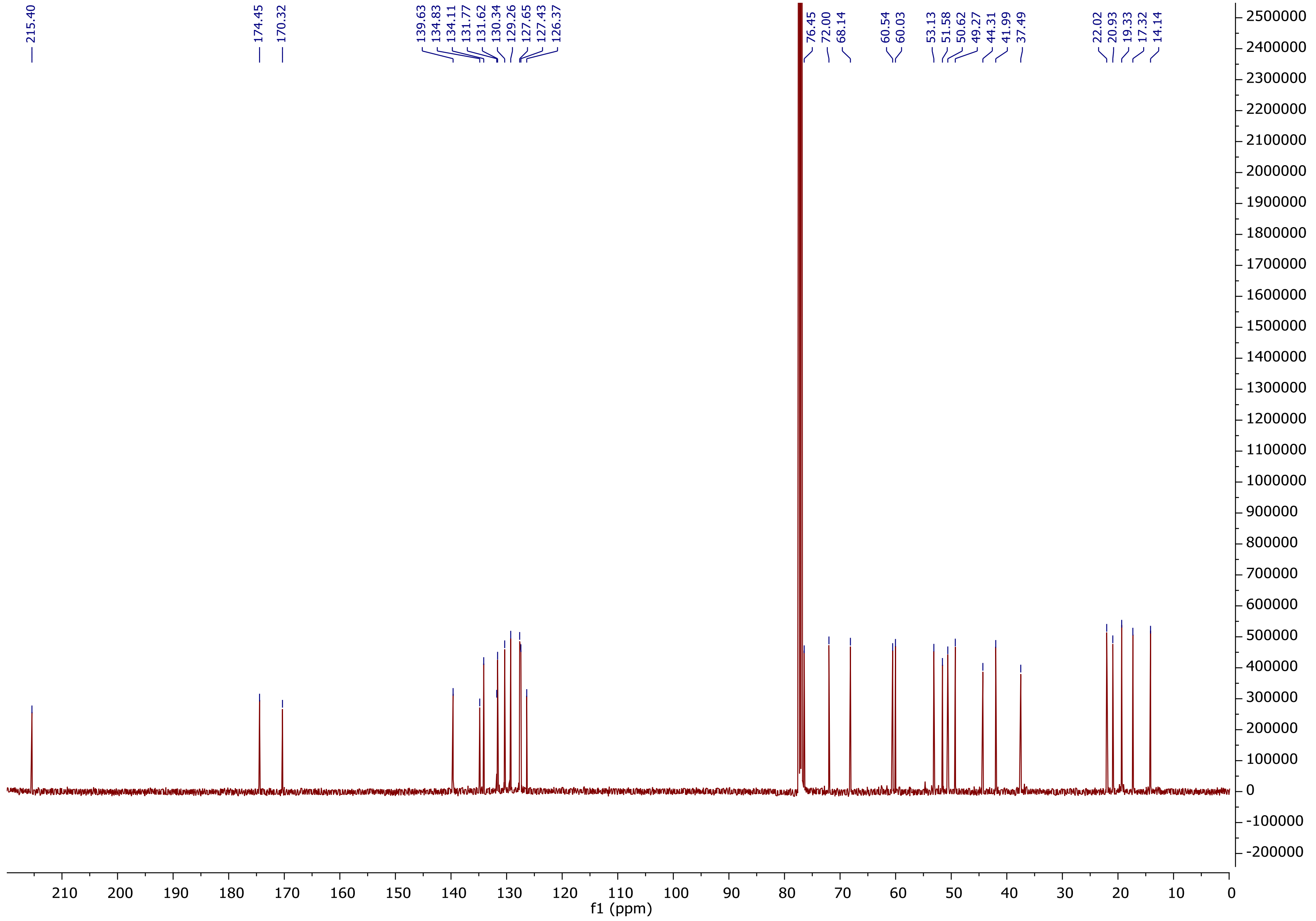

Figure SD73. ^{13}C NMR spectrum of *m*-chloro-19,20-epoxycytochalasin C (**11**) (125 MHz, $CDCl_3$)

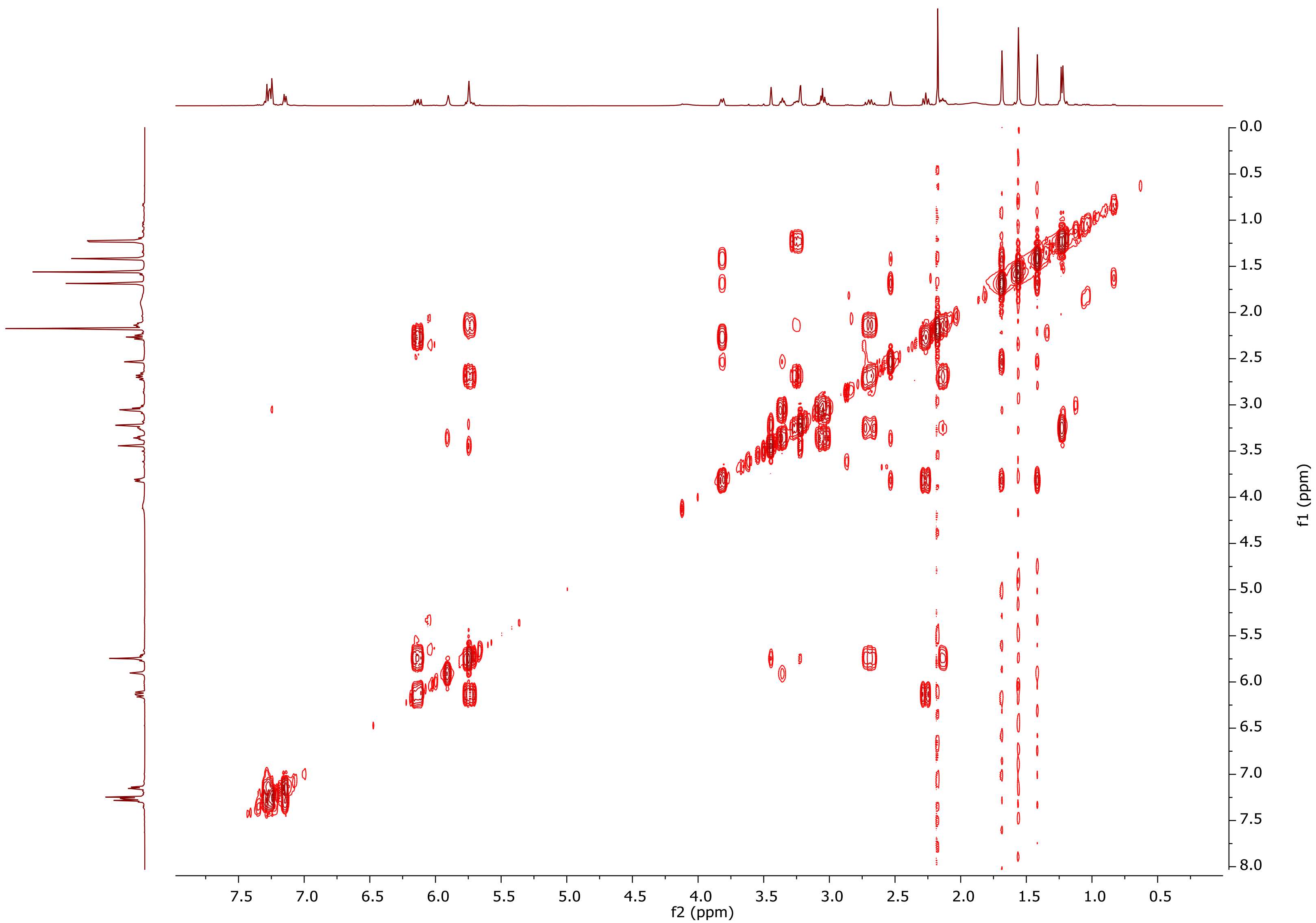

Figure SD74. ¹H-¹H COSY NMR spectrum of *m*-chloro-19,20-epoxycytochalasin C (**11**) (500/500 MHz, $CDCl_3$)

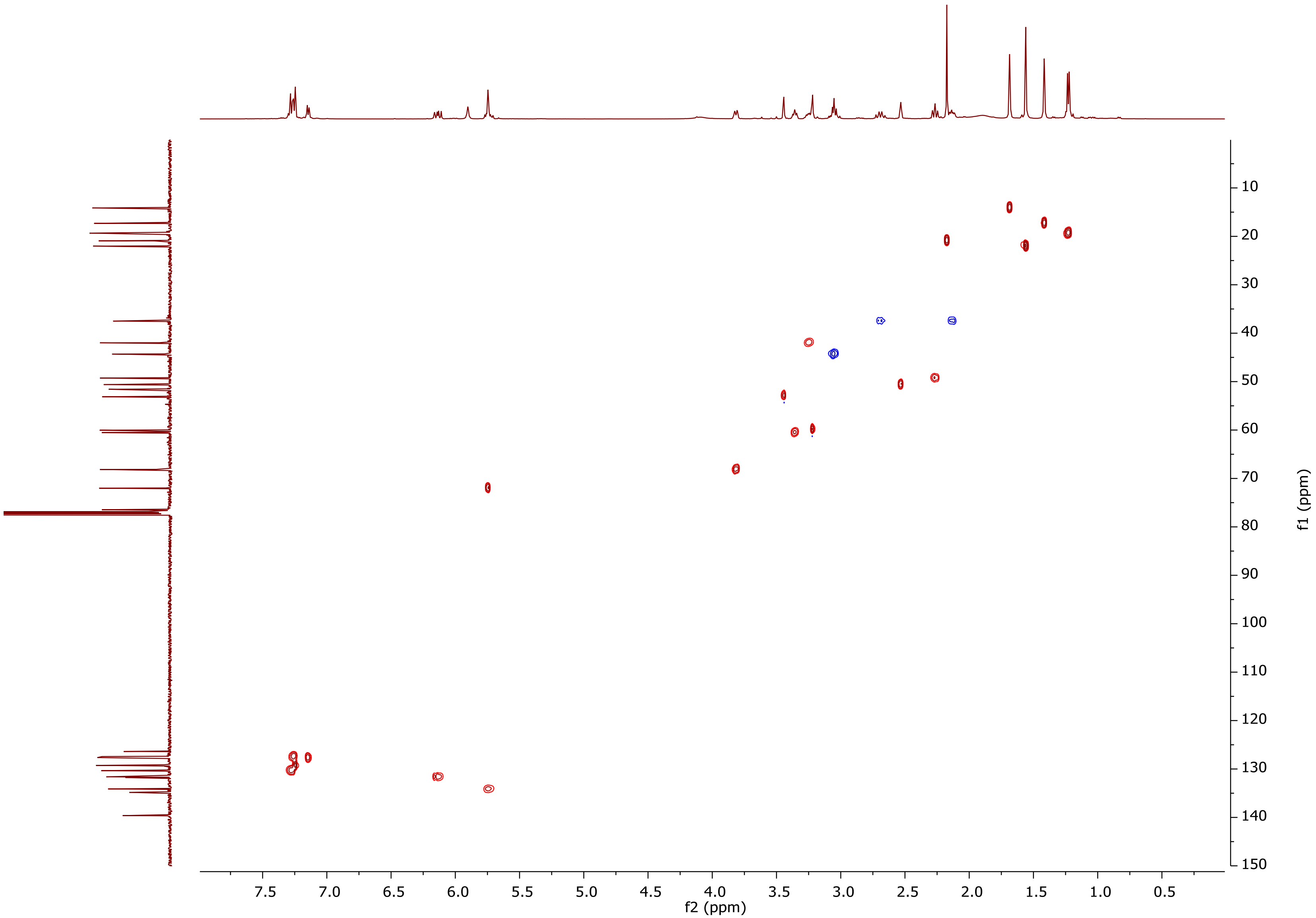

Figure SD75. ^{1}H-^{13}C HSQC NMR spectrum of *m*-chloro-19,20-epoxycytochalasin C (**11**) (500/125 MHz, $CDCl_3$)

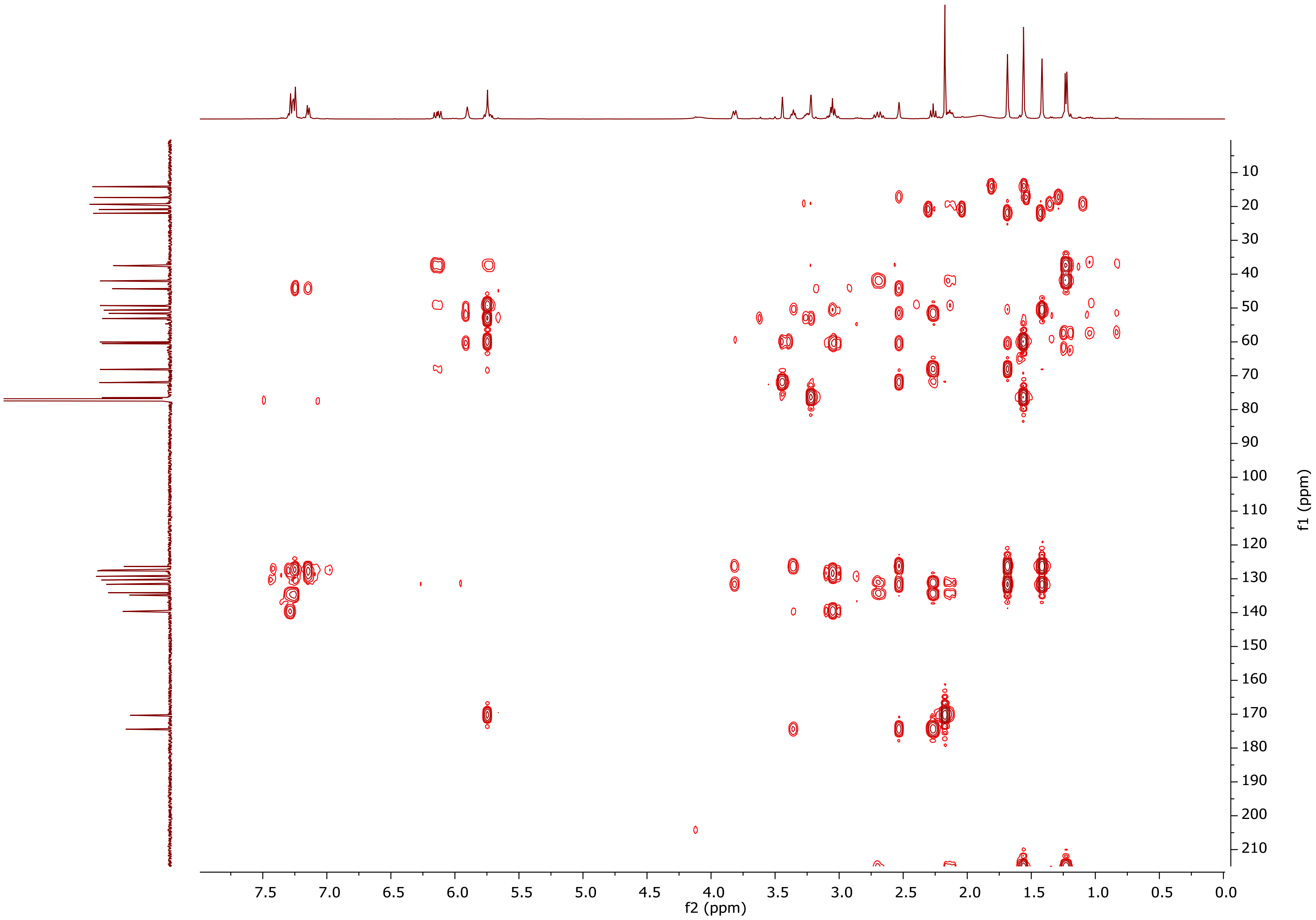

Figure SD76. ^{1}H-^{13}C HMBC NMR spectrum of *m*-chloro-19,20-epoxycytochalasin C (**11**) (500/125 MHz, CDCl$_3$)

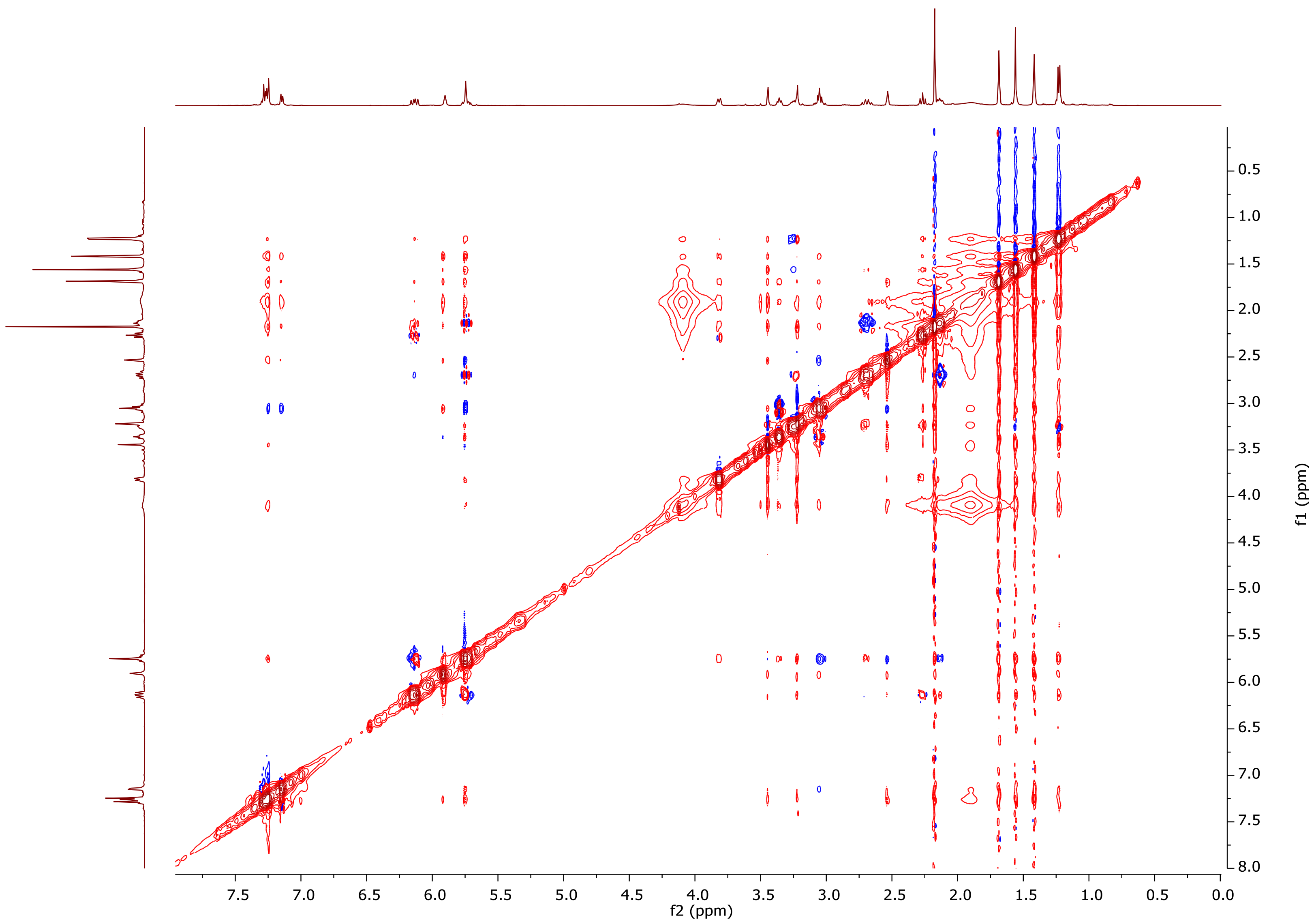

Figure SD77. ^{1}H-^{1}H NOESY NMR spectrum of *m*-chloro-19,20-epoxycytochalasin C (**11**) (500/500 MHz, $CDCl_3$)

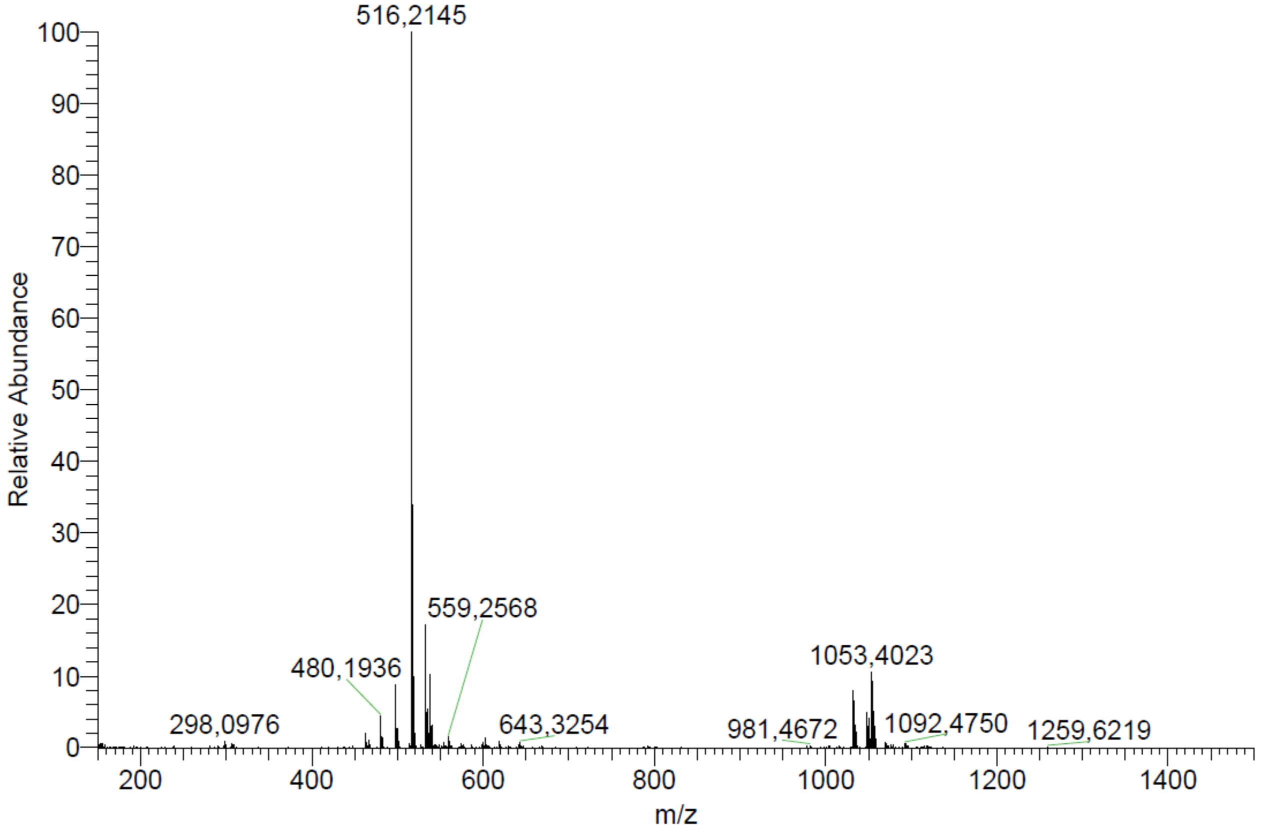

Figure SD78. ESI-HRMS spectrum of *m*-chloro-deacetyl-19,20-epoxycytochalasin C (**12**)

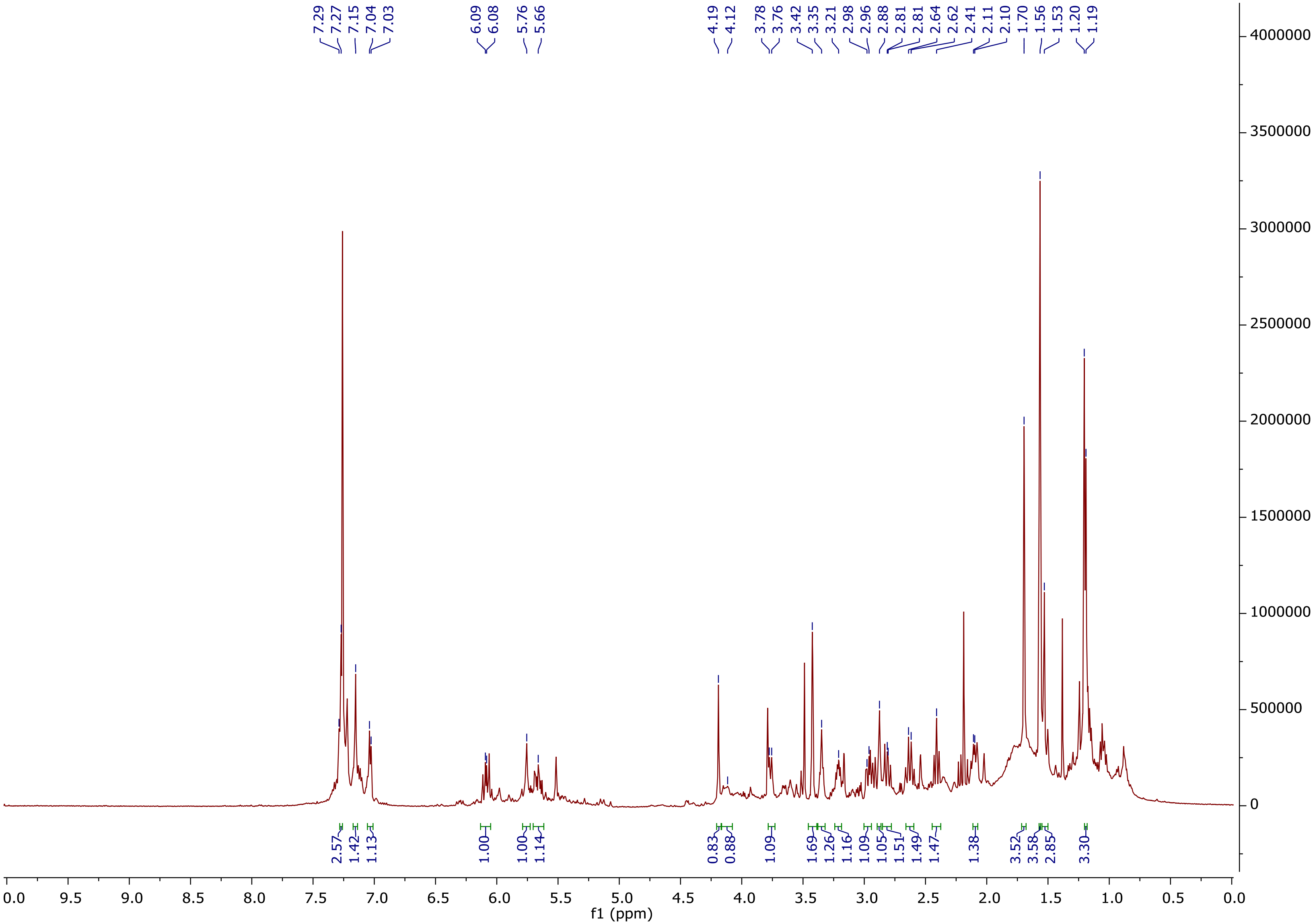

Figure SD79. ^{1}H NMR spectrum of *m*-chloro-deacetyl-19,20-epoxycytochalasin C (**12**) (500 MHz, $CDCl_3$)

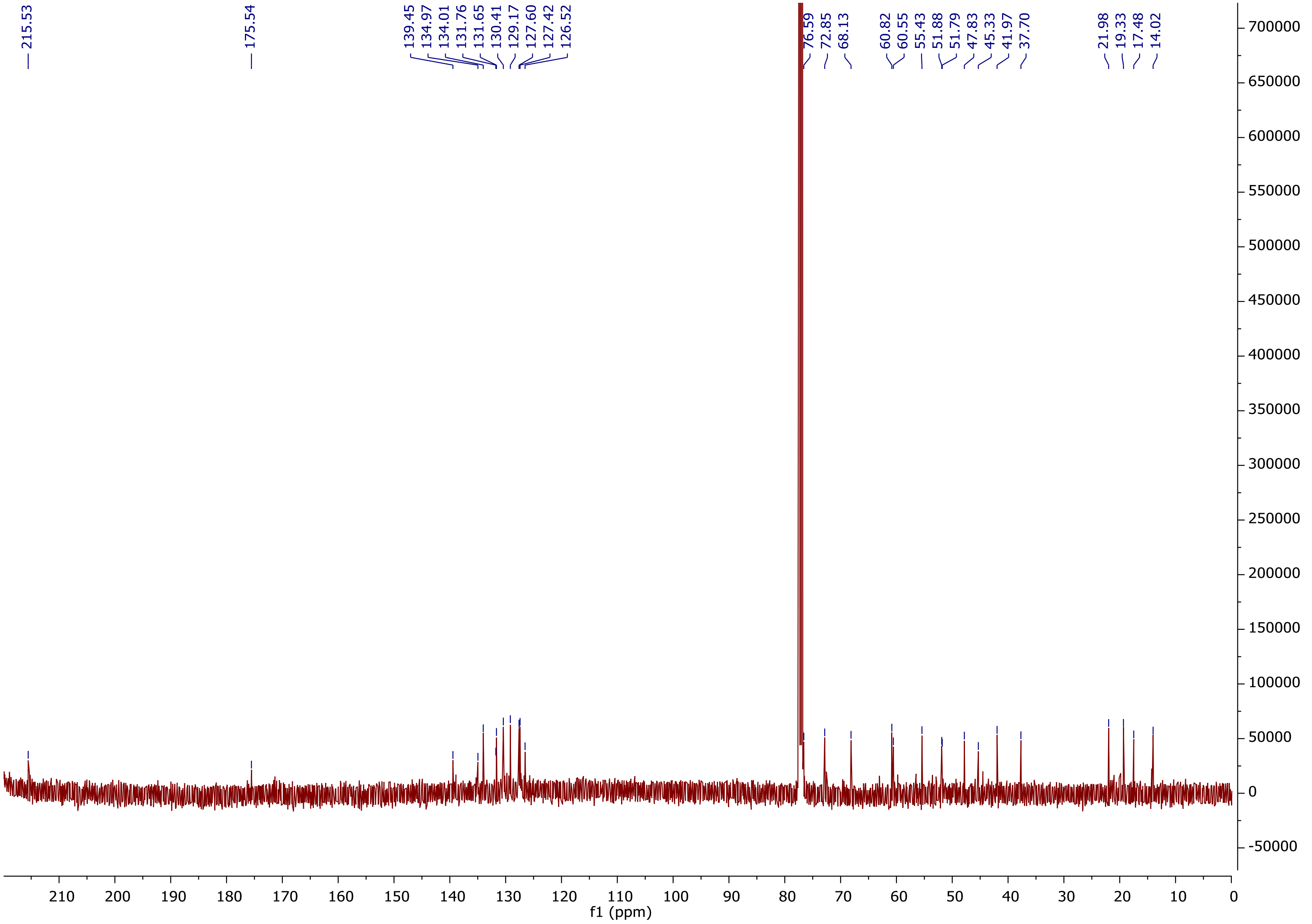

Figure SD80. ^{13}C NMR spectrum of *m*-chloro-deacetyl-19,20-epoxycytochalasin C (**12**) (125 MHz, $CDCl_3$)

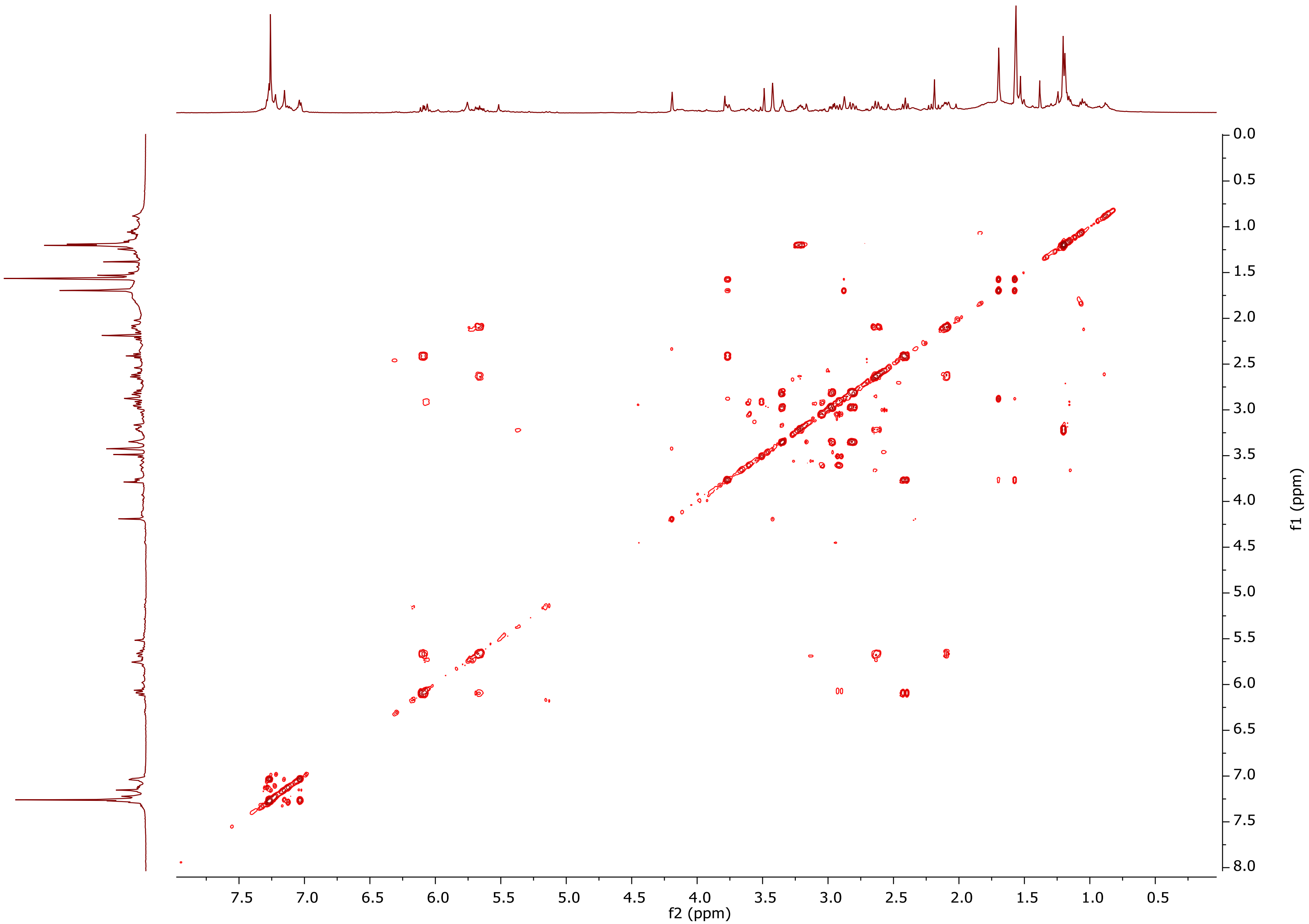

Figure SD81. ^1H-^1H COSY NMR spectrum of *m*-chloro-deacetyl-19,20-epoxycytochalasin C (**12**) (500/500 MHz, $CDCl_3$)

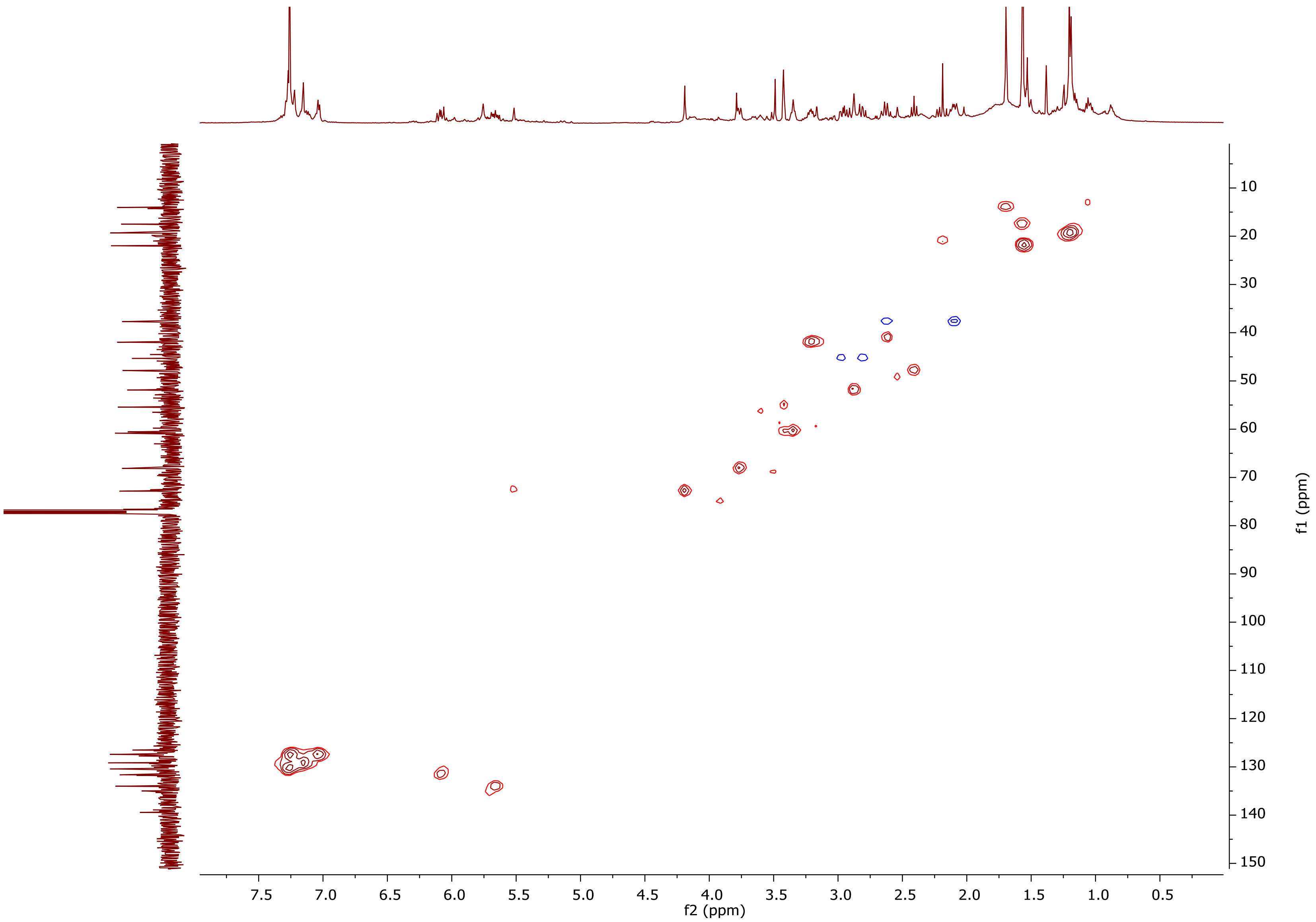

Figure SD82. ^{1}H-^{13}C HSQC NMR spectrum of *m*-chloro-deacetyl-19,20-epoxycytochalasin C (**12**) (500/125 MHz, $CDCl_3$)

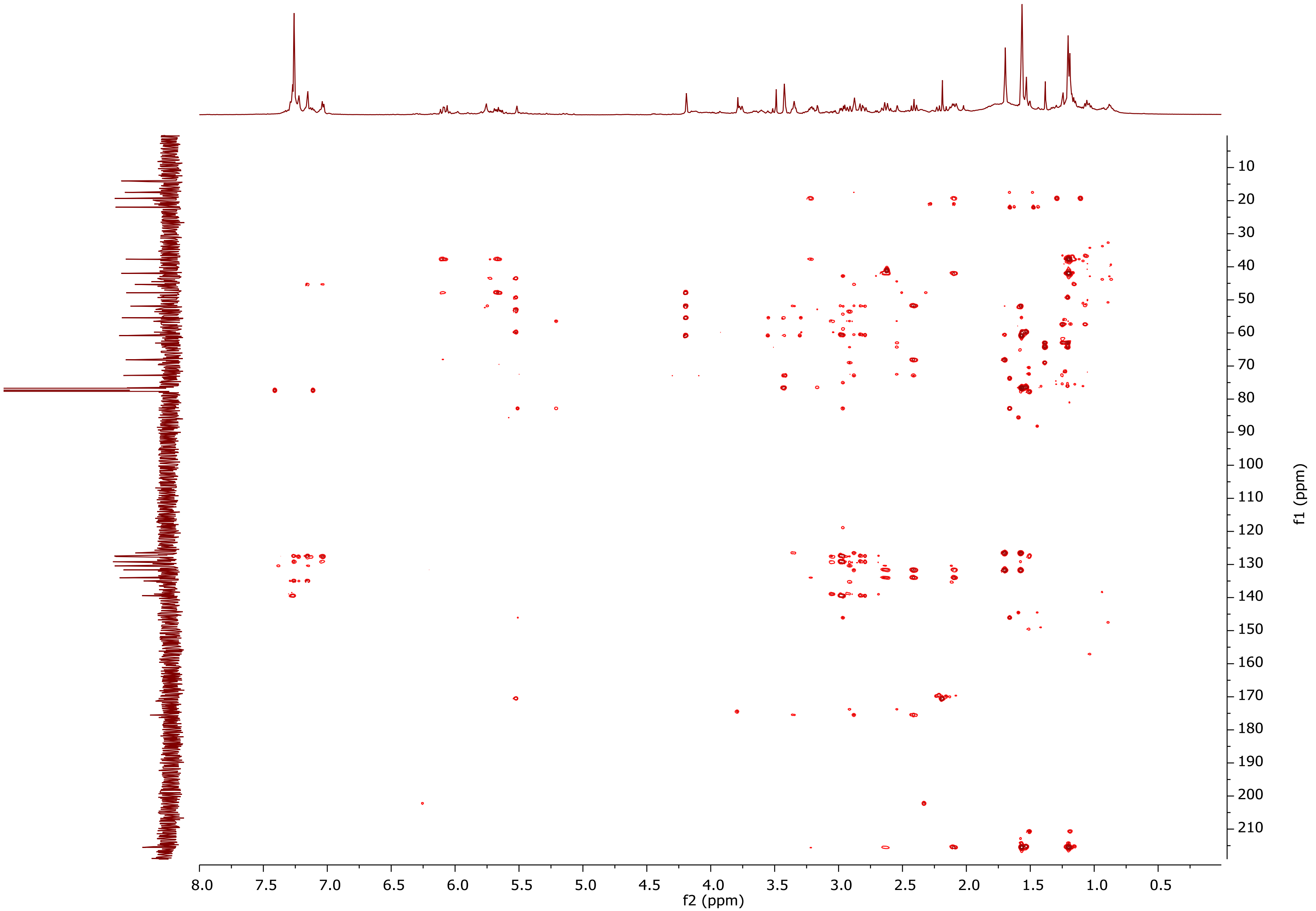

Figure SD83. ^{1}H-^{13}C HMBC NMR spectrum of *m*-chloro-deacetyl-19,20-epoxycytochalasin C (**12**) (500/125 MHz, $CDCl_3$)

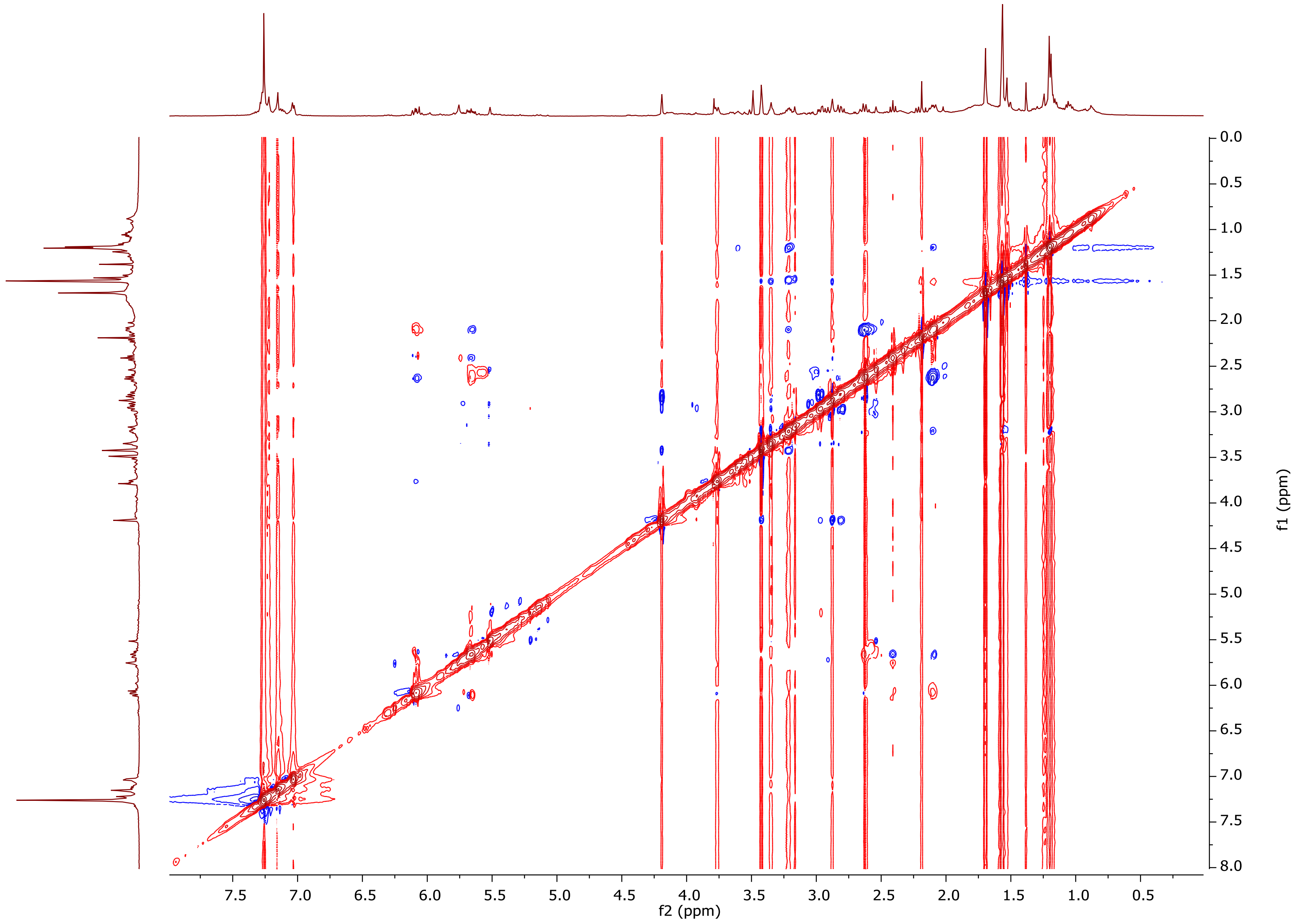

Figure SD84. ^{1}H-^{1}H NOESY NMR spectrum of *m*-chloro-deacetyl-19,20-epoxycytochalasin C (**12**) (500/500 MHz, $CDCl_3$)

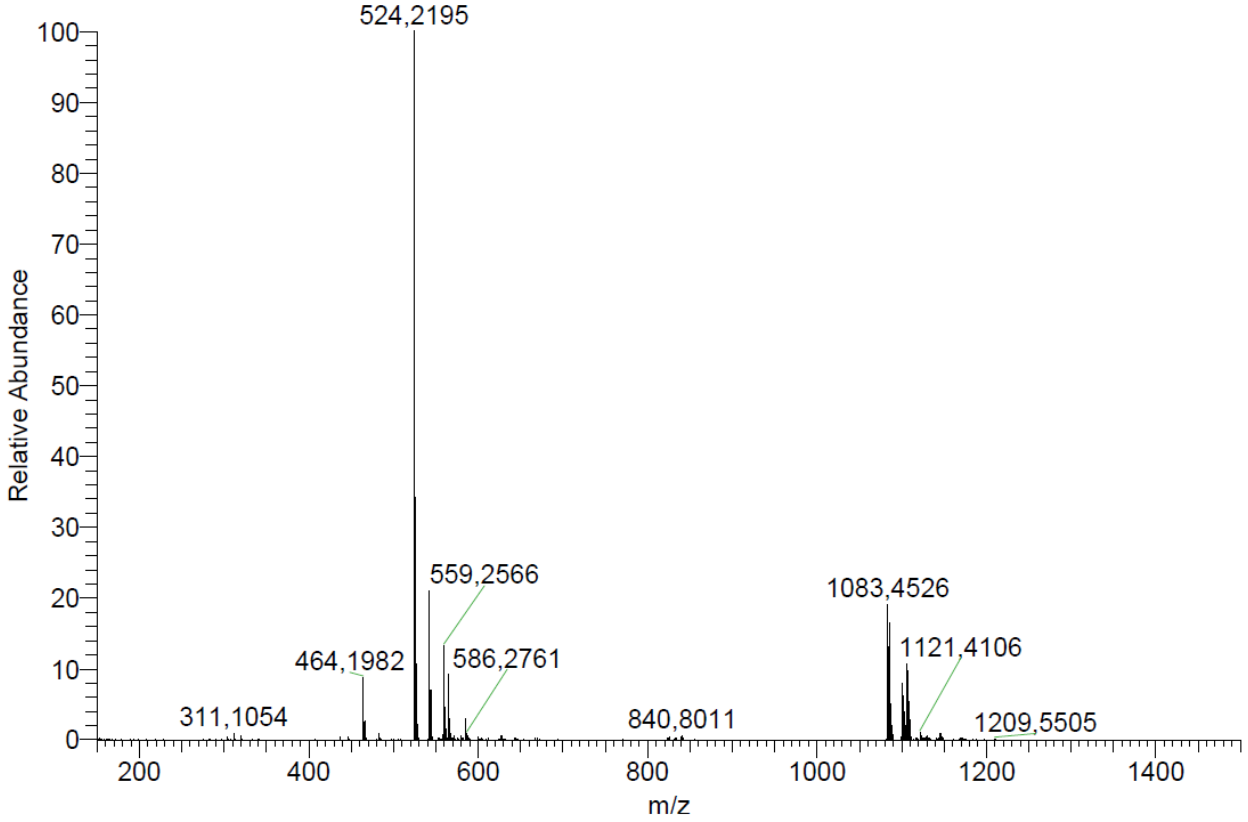

Figure SD85. ESI-HRMS spectrum of *m*-chloro-cytochalasin Q (**13**)

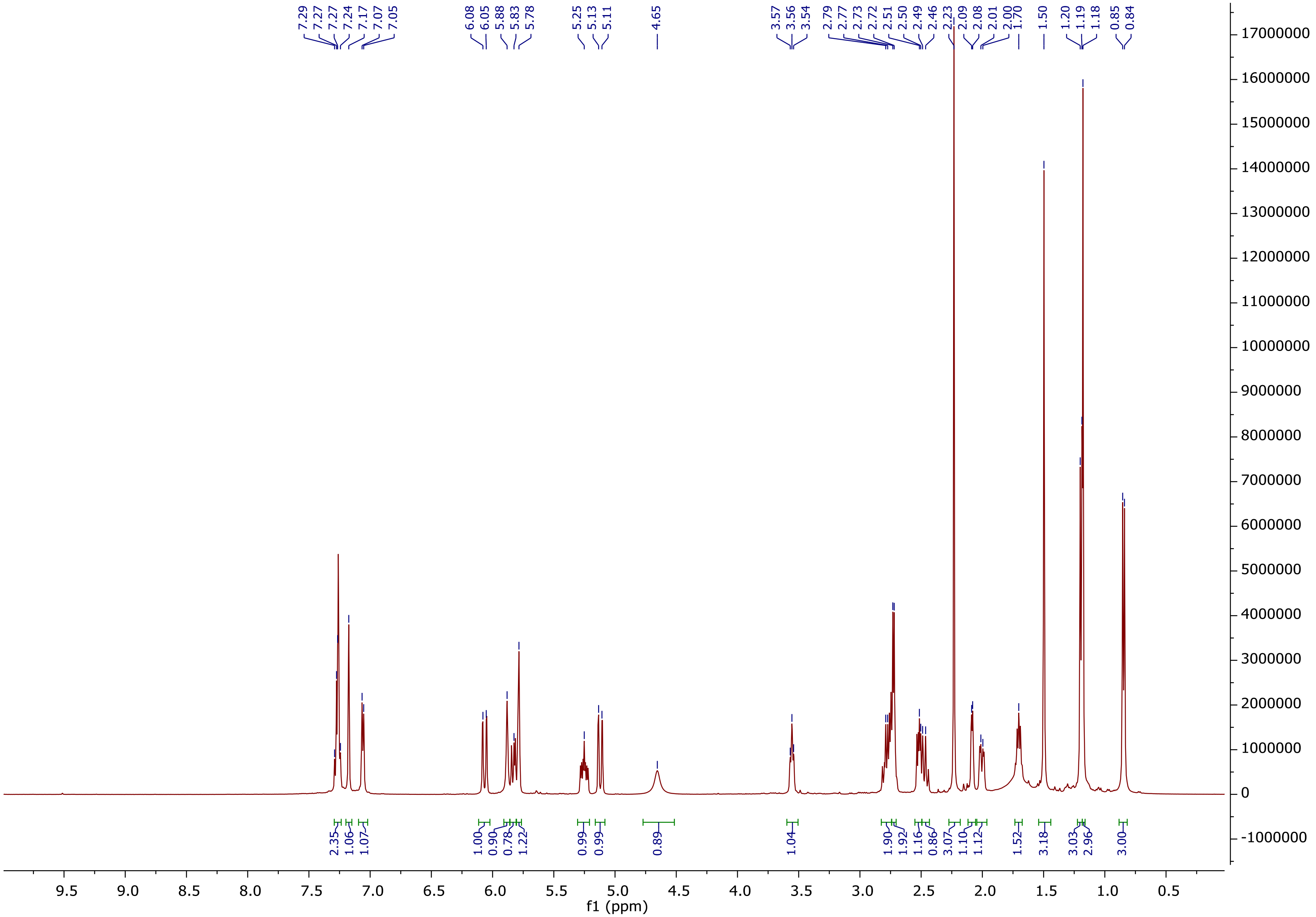

Figure SD86. ^{1}H NMR spectrum of *m*-chloro-cytochalasin Q (**13**) (500 MHz, $CDCl_3$)

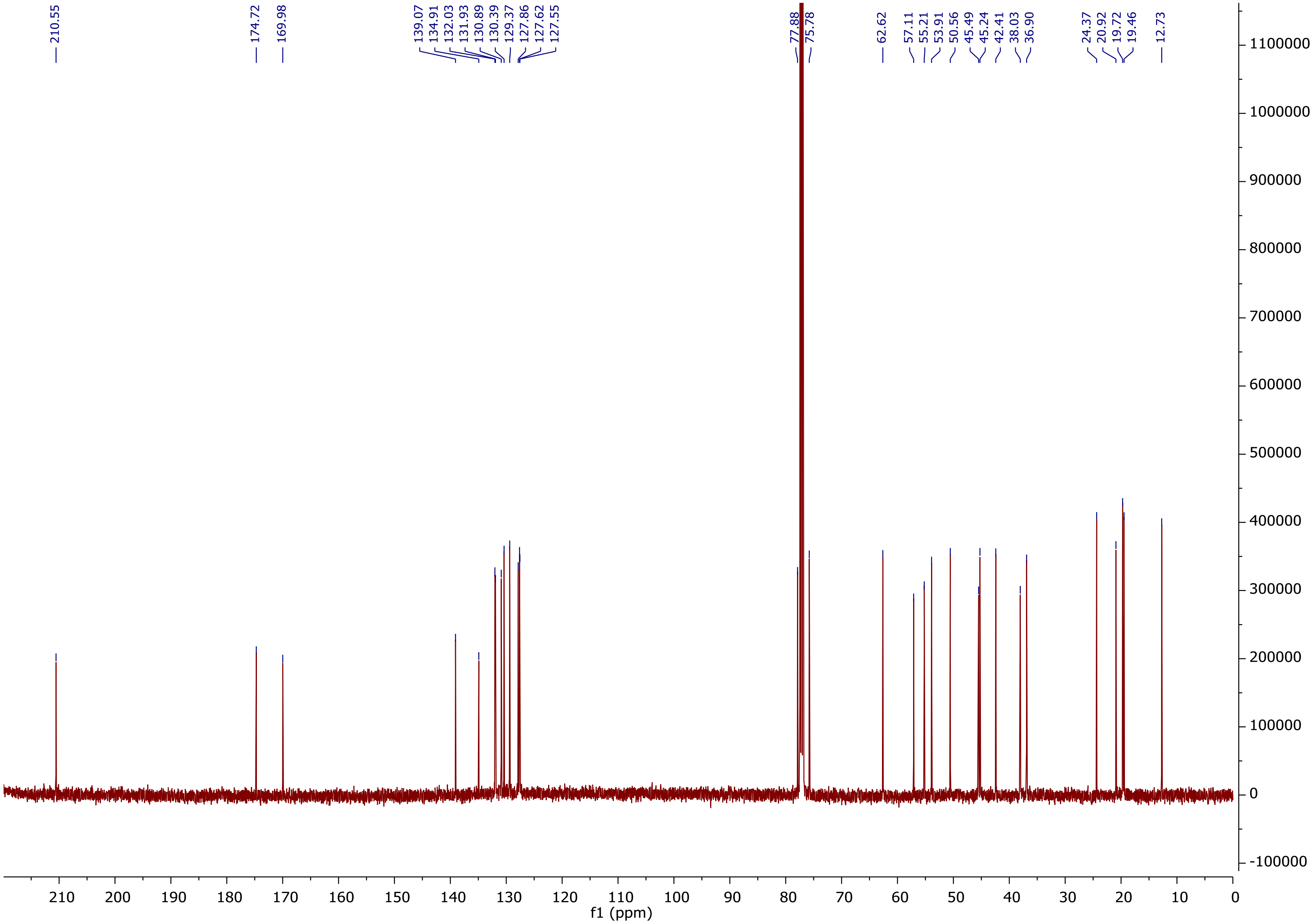

Figure SD87. ^{13}C NMR spectrum of *m*-chloro-cytochalasin Q (**13**) (125 MHz, $CDCl_3$)

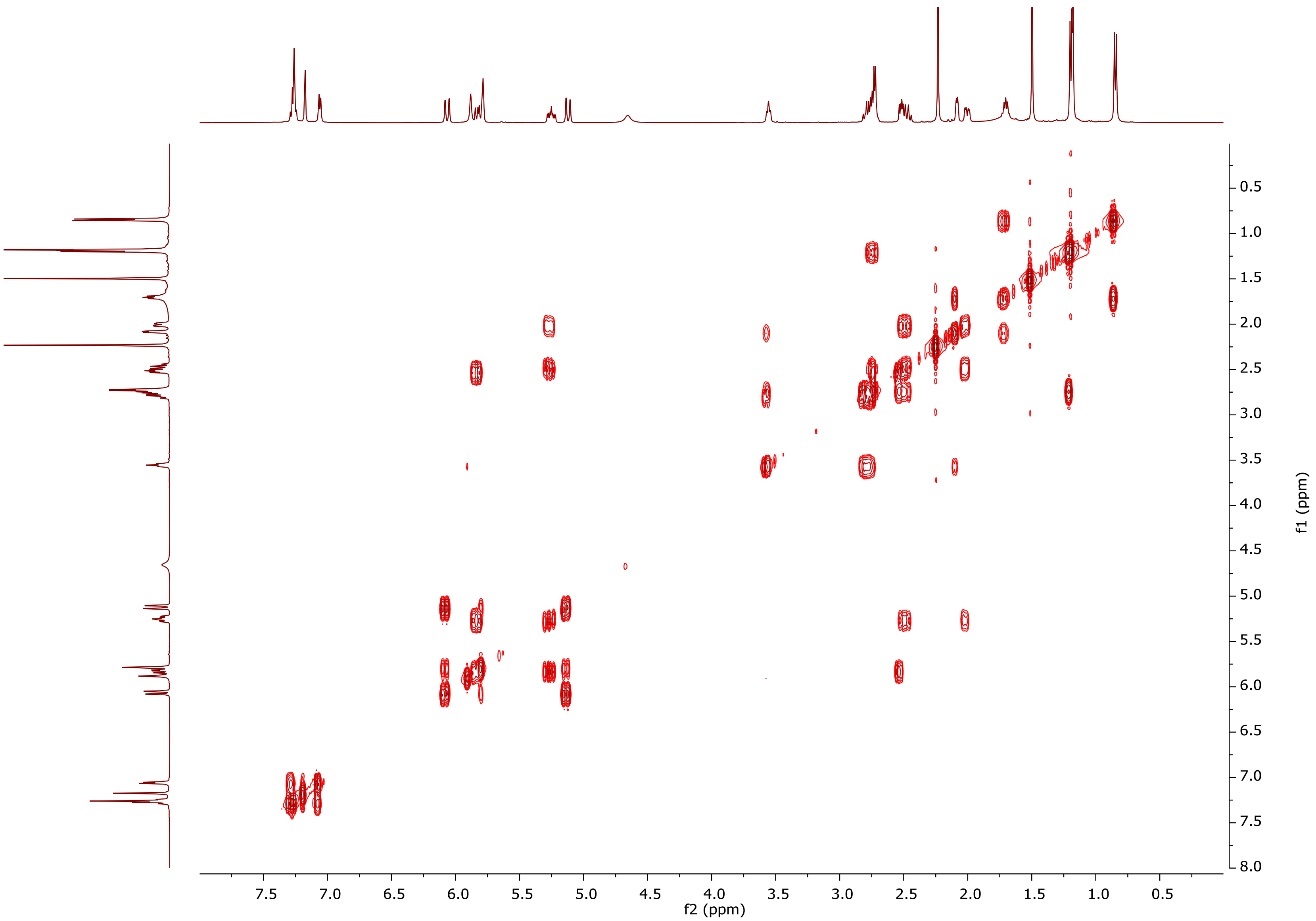

Figure SD88. ^1H-^1H COSY NMR spectrum of *m*-chloro-cytochalasin Q (**13**) (500/500 MHz, $CDCl_3$)

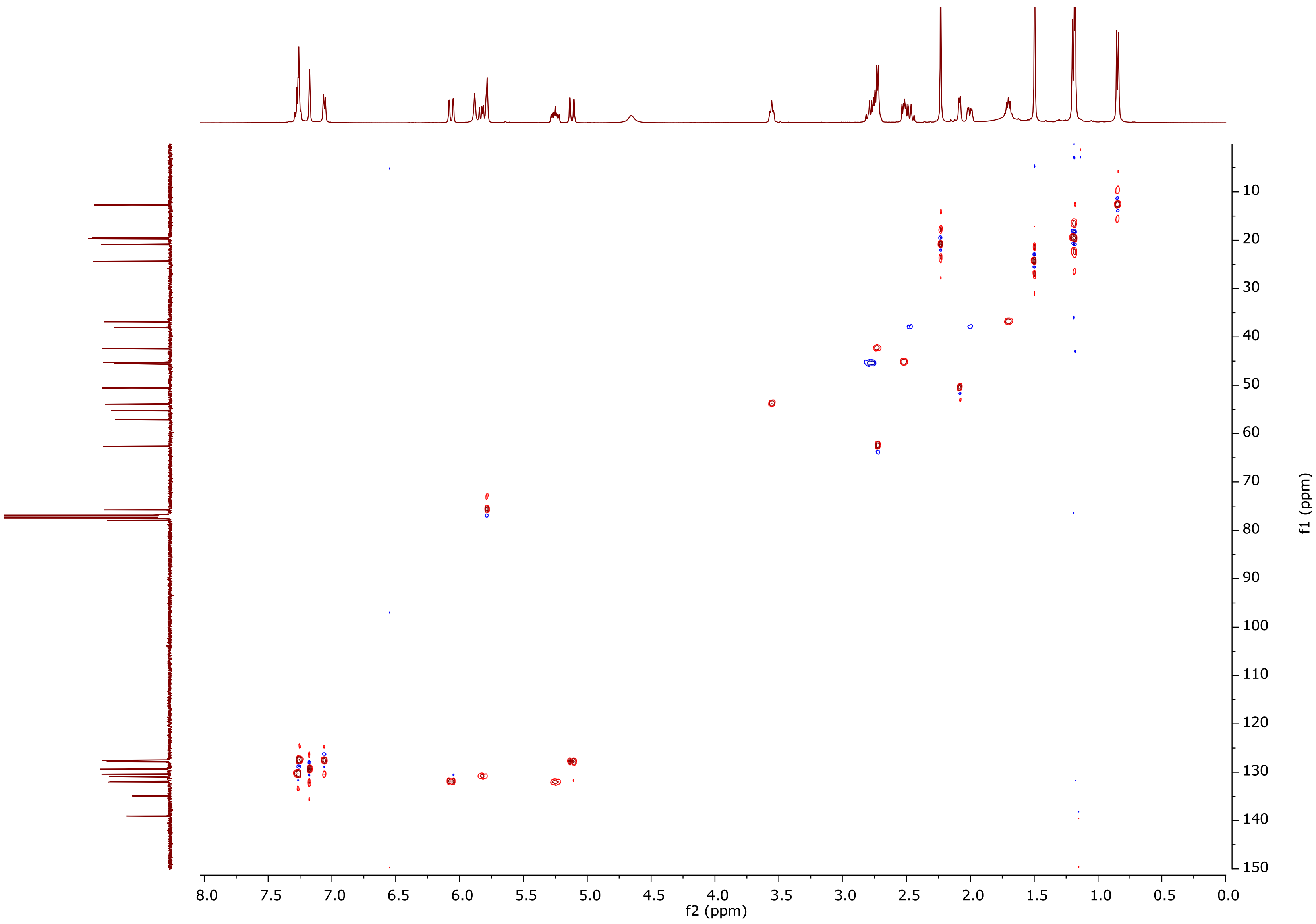

Figure SD89. ^{1}H-^{13}C HSQC NMR spectrum of *m*-chloro-cytochalasin Q (**13**) (500/125 MHz, $CDCl_3$)

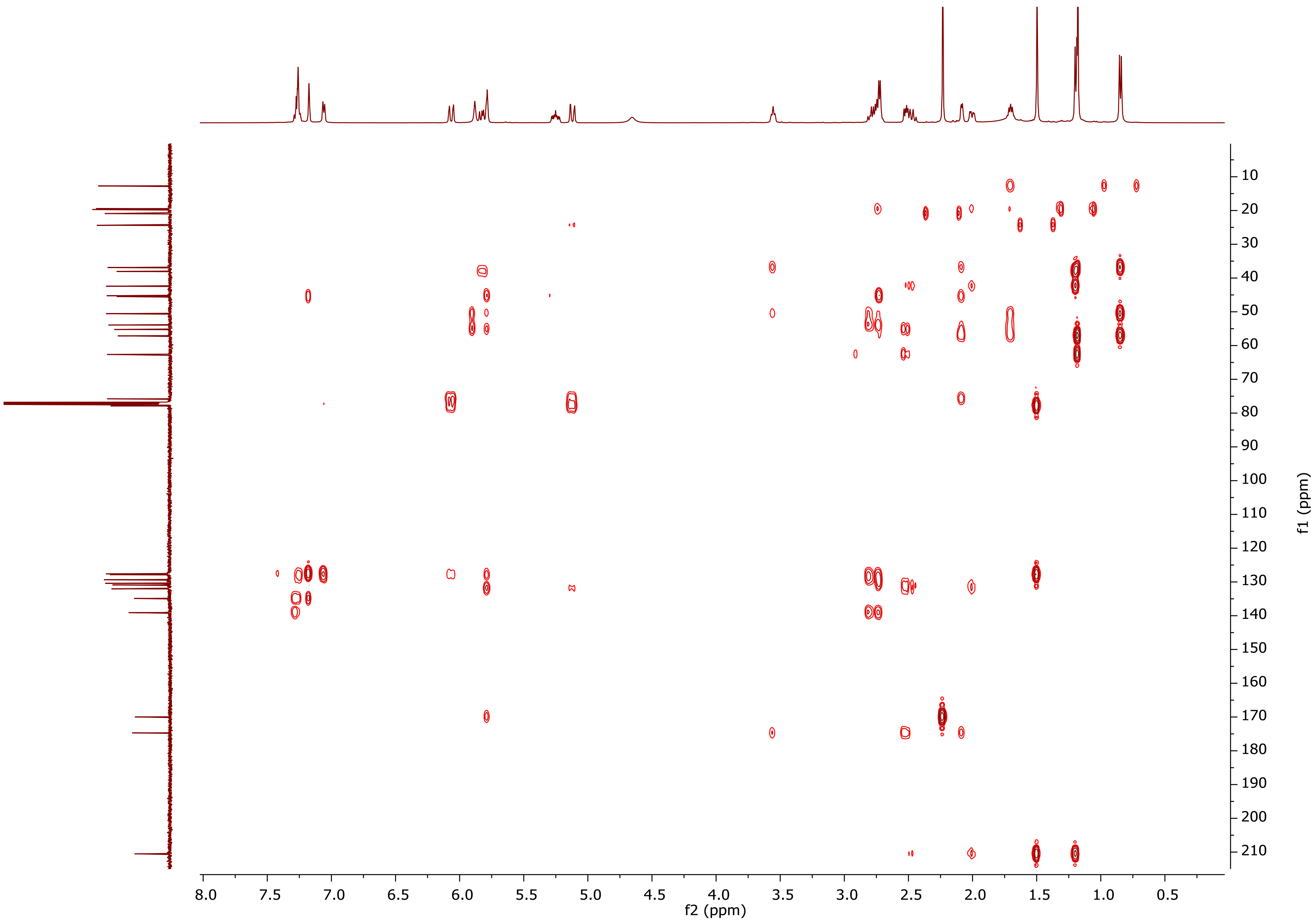

Figure SD90. ^{1}H-^{13}C HMBC NMR spectrum of *m*-chloro-cytochalasin Q (**13**) (500/125 MHz, $CDCl_3$)

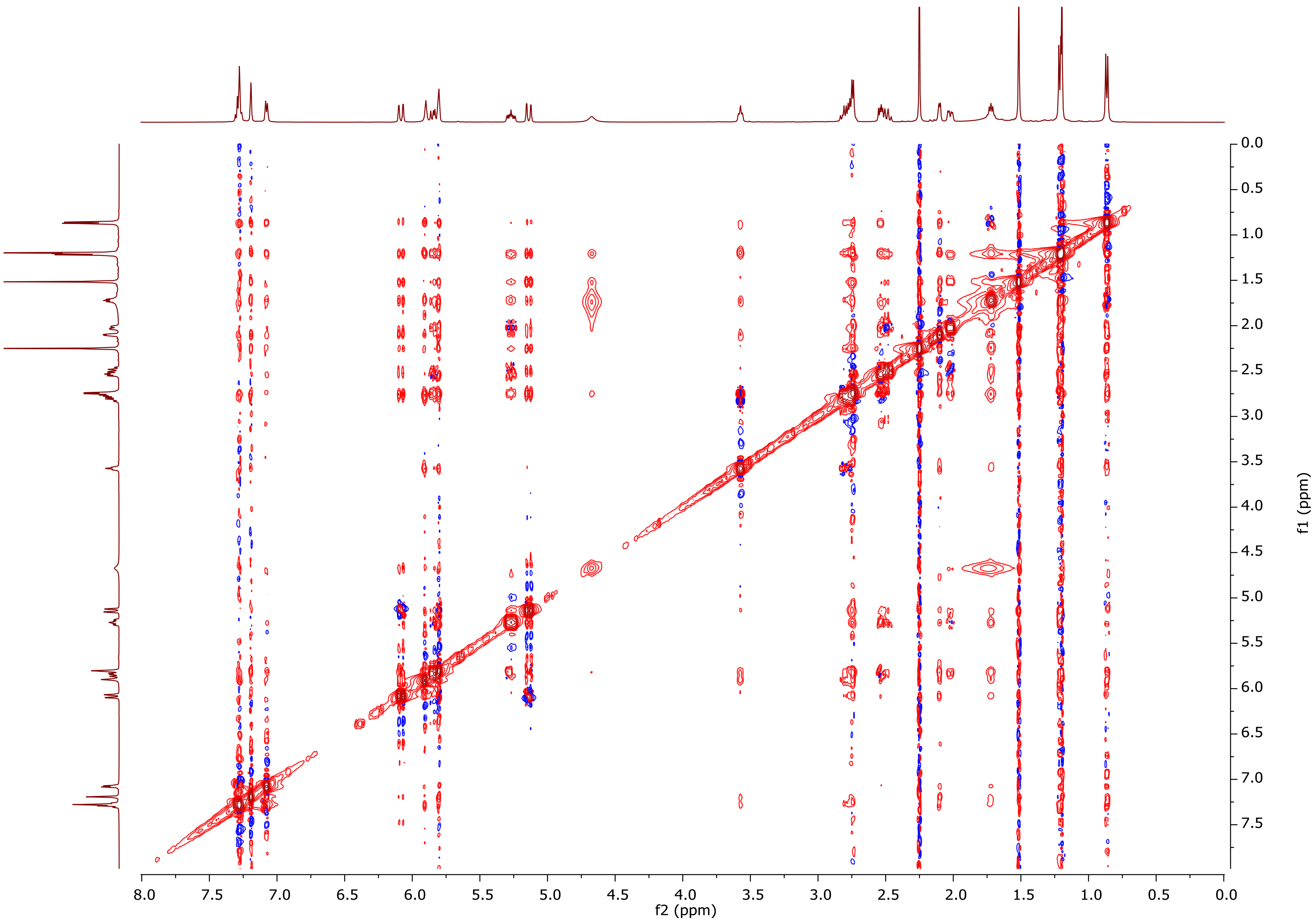

Figure SD91. ^{1}H-^{1}H NOESY NMR spectrum of *m*-chloro-cytochalasin Q (**13**) (500/500 MHz, $CDCl_3$)

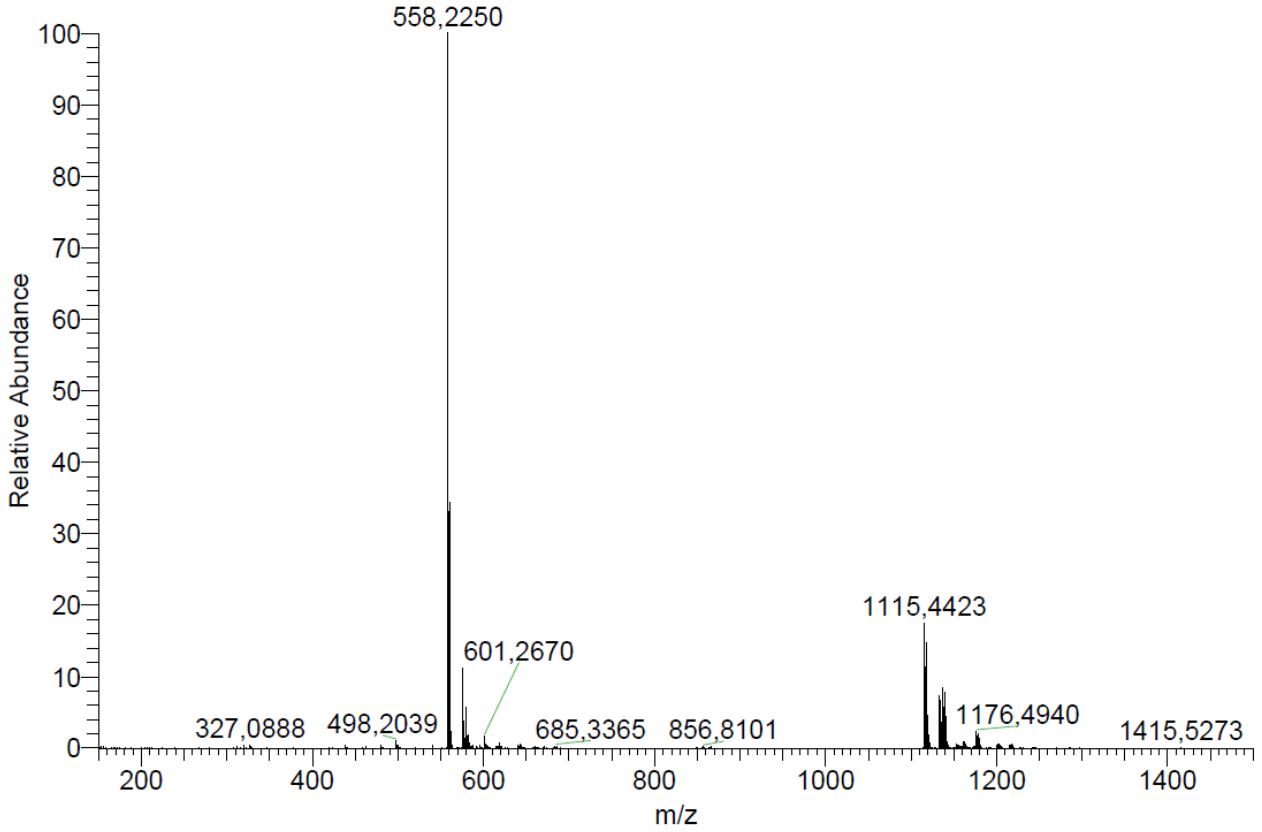

Figure SD92. ESI-HRMS spectrum of *m*-chloro-19,20-epocycytochalasin Q (**14**)

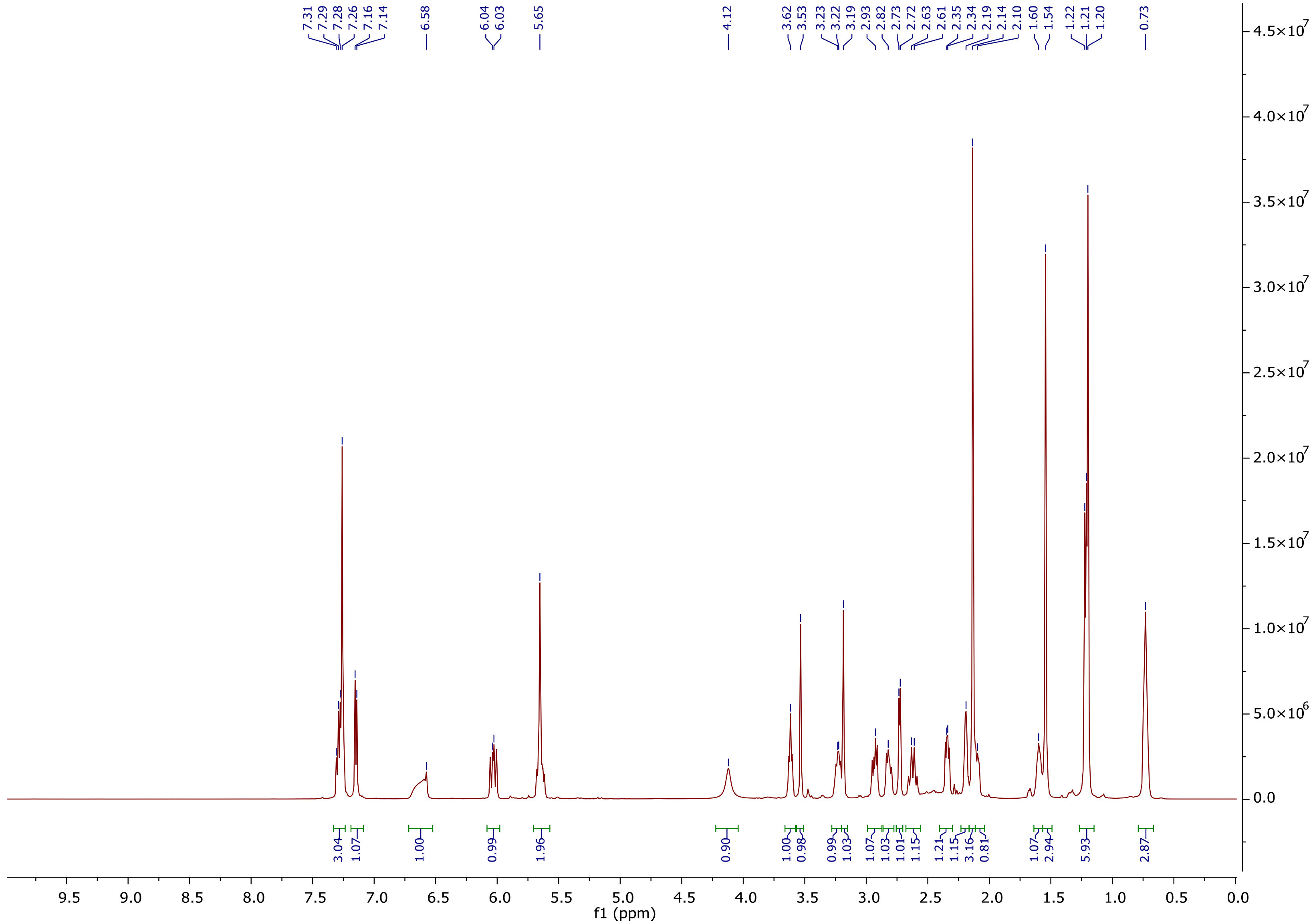

Figure SD93. ^{1}H NMR spectrum of *m*-chloro-19,20-epocycytochalasin Q (**14**) (500 MHz, $CDCl_3$)

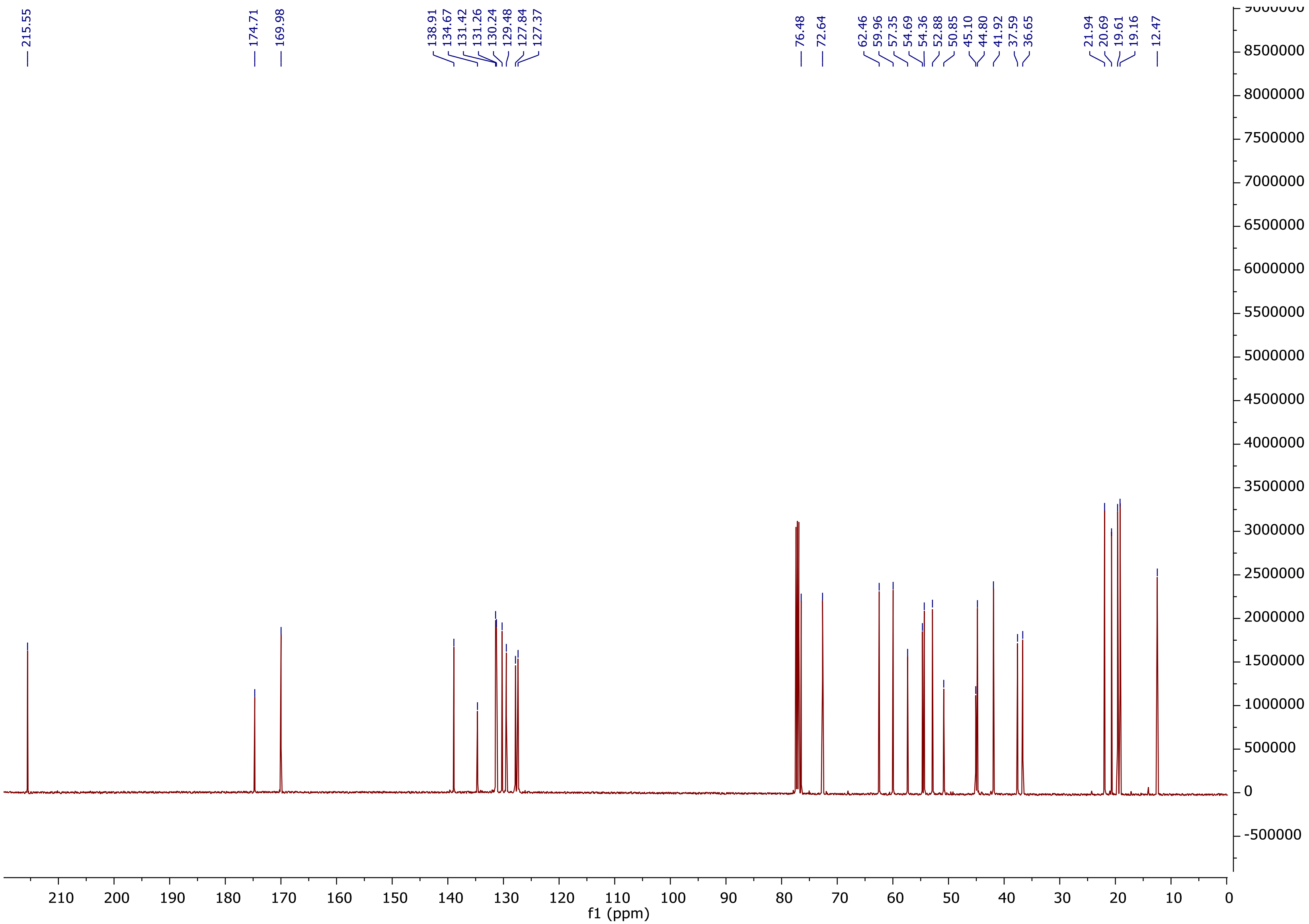

Figure SD94. ^{13}C NMR spectrum of *m*-chloro-19,20-epocycytochalasin Q (**14**) (125 MHz, $CDCl_3$)

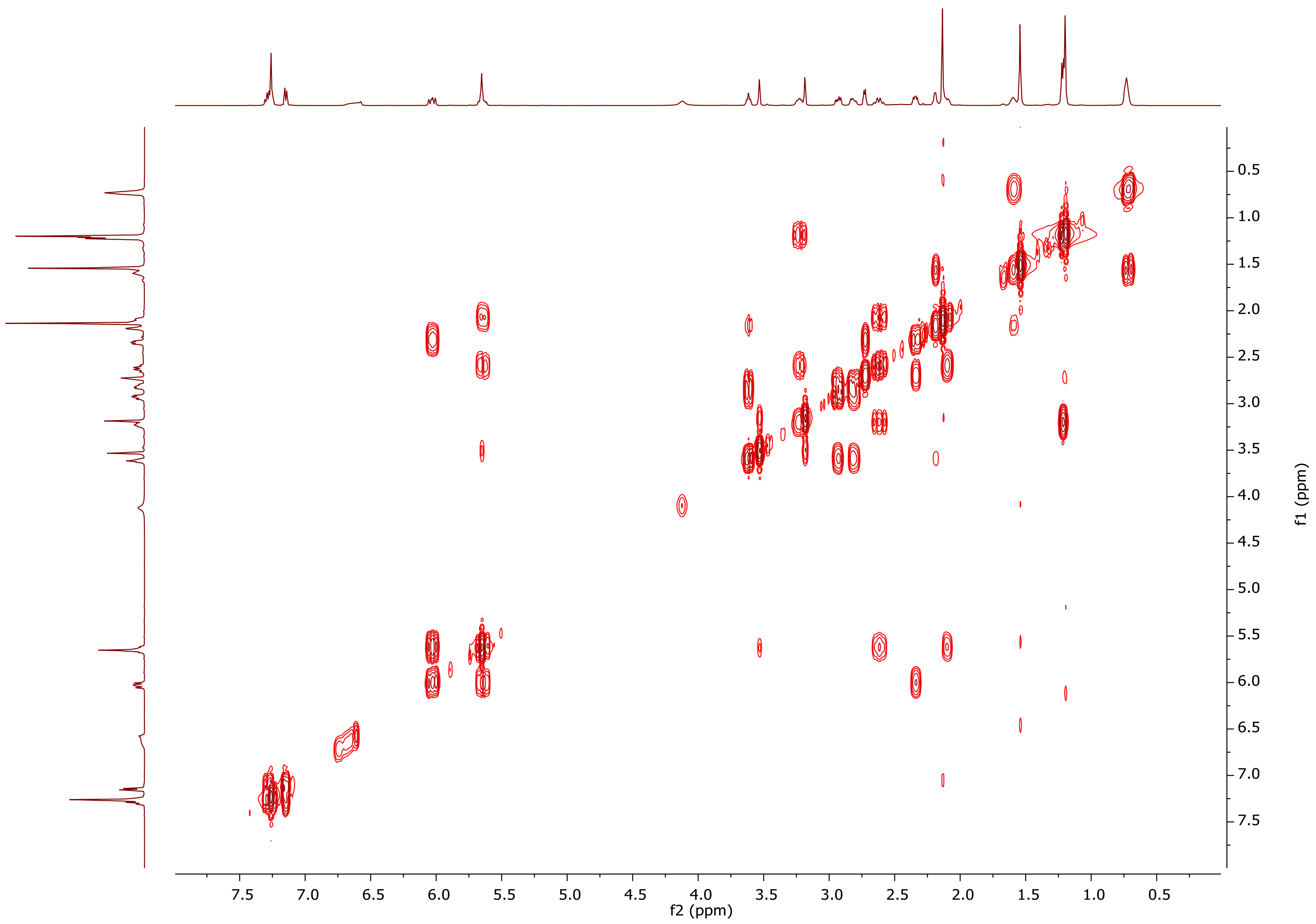

Figure SD95. ^{1}H-^{1}H COSY NMR spectrum of *m*-chloro-19,20-epocycytochalasin Q (**14**) (500/500 MHz, $CDCl_3$)

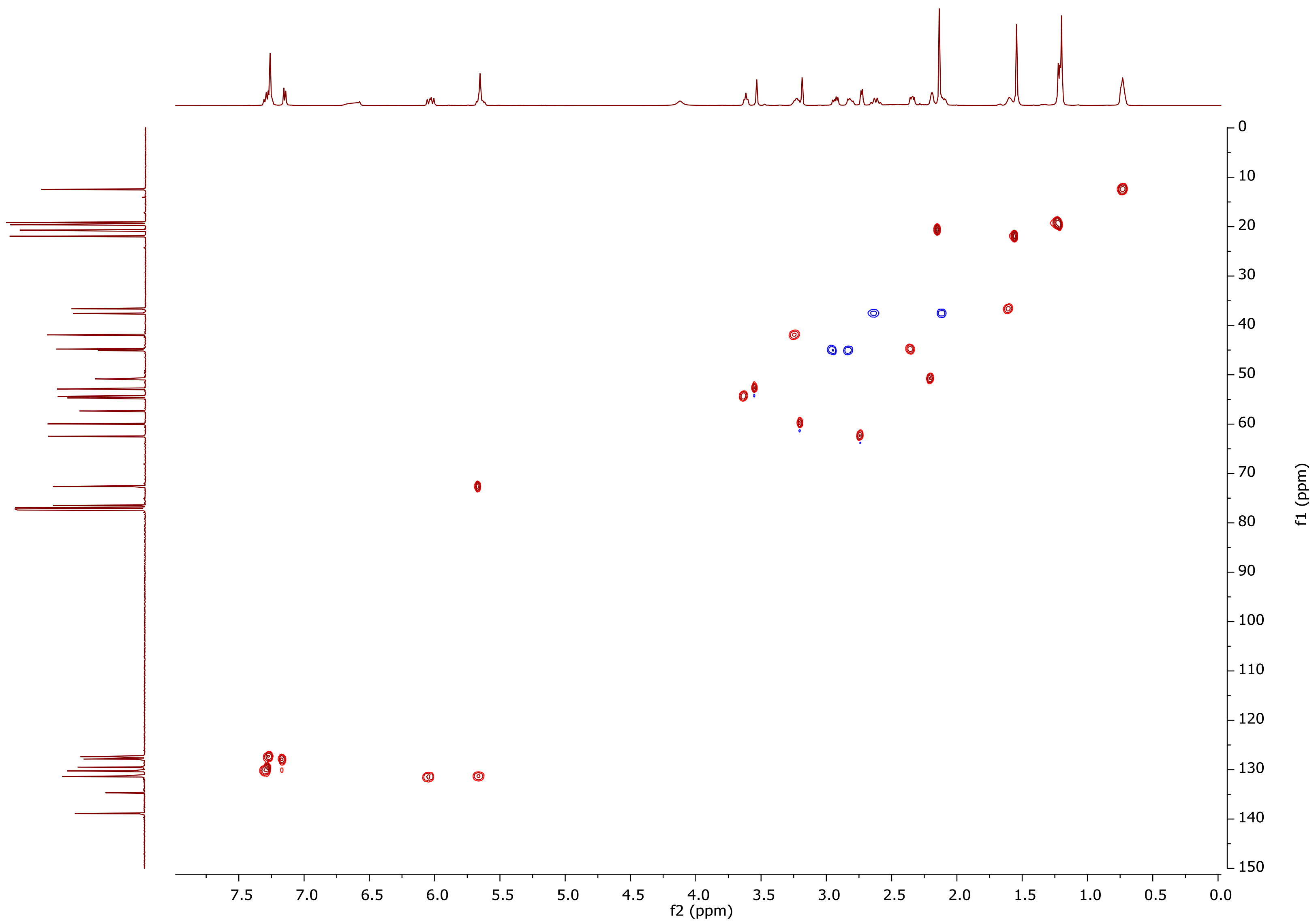

Figure SD96. ^{1}H-^{13}C HSQC NMR spectrum of *m*-chloro-19,20-epocycytochalasin Q (**14**) (500/125 MHz, $CDCl_3$)

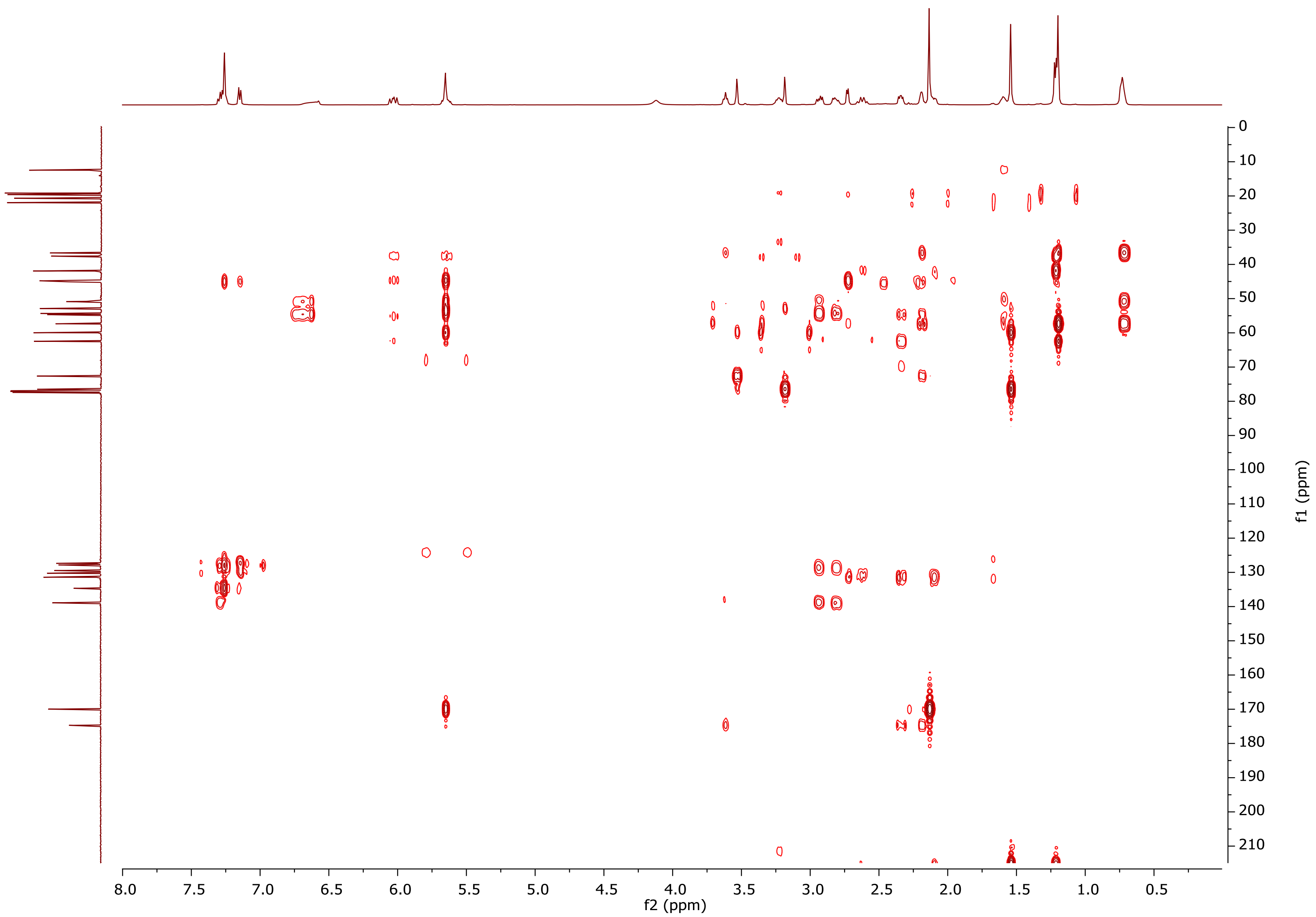

Figure SD97. ^{1}H-^{13}C HMBC NMR spectrum of *m*-chloro-19,20-epocycytochalasin Q (**14**) (500/125 MHz, $CDCl_3$)

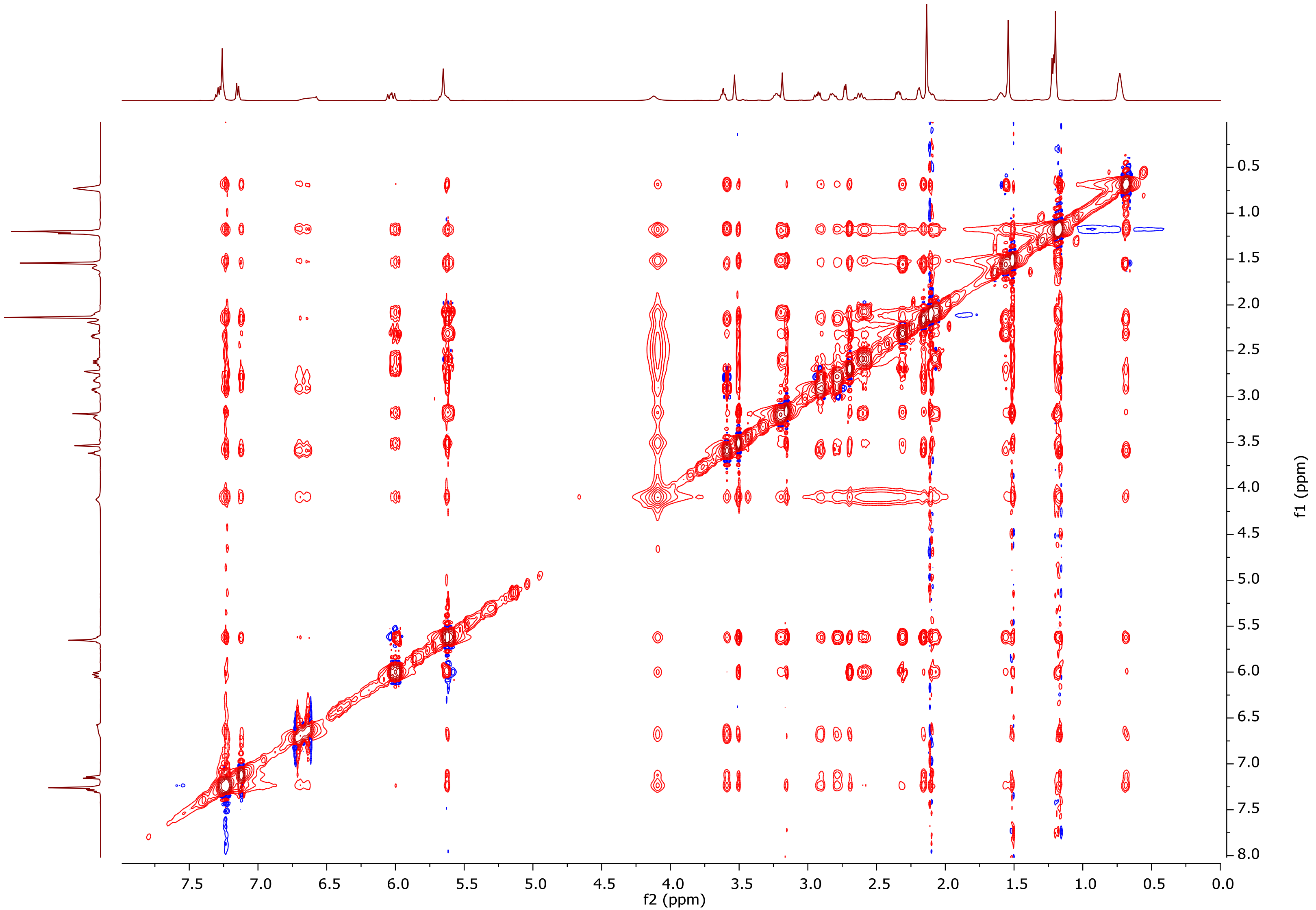

Figure SD98. ^{1}H-^{1}H NOESY NMR spectrum of *m*-chloro-19,20-epocycytochalasin Q (**14**) (500/500 MHz, $CDCl_3$)

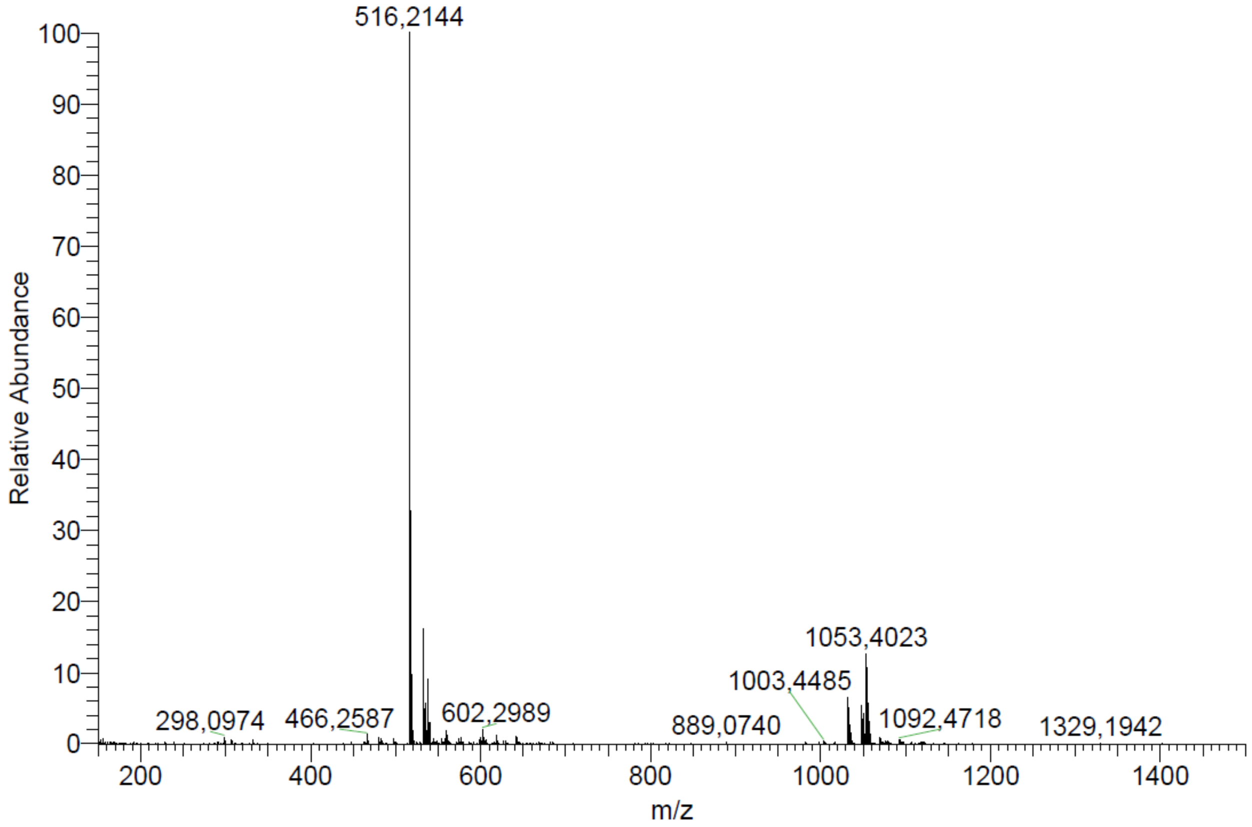

Figure SD99. ESI-HRMS spectrum of *m*-chloro-deacetyl-19,20-epocycytochalasin Q (**15**)

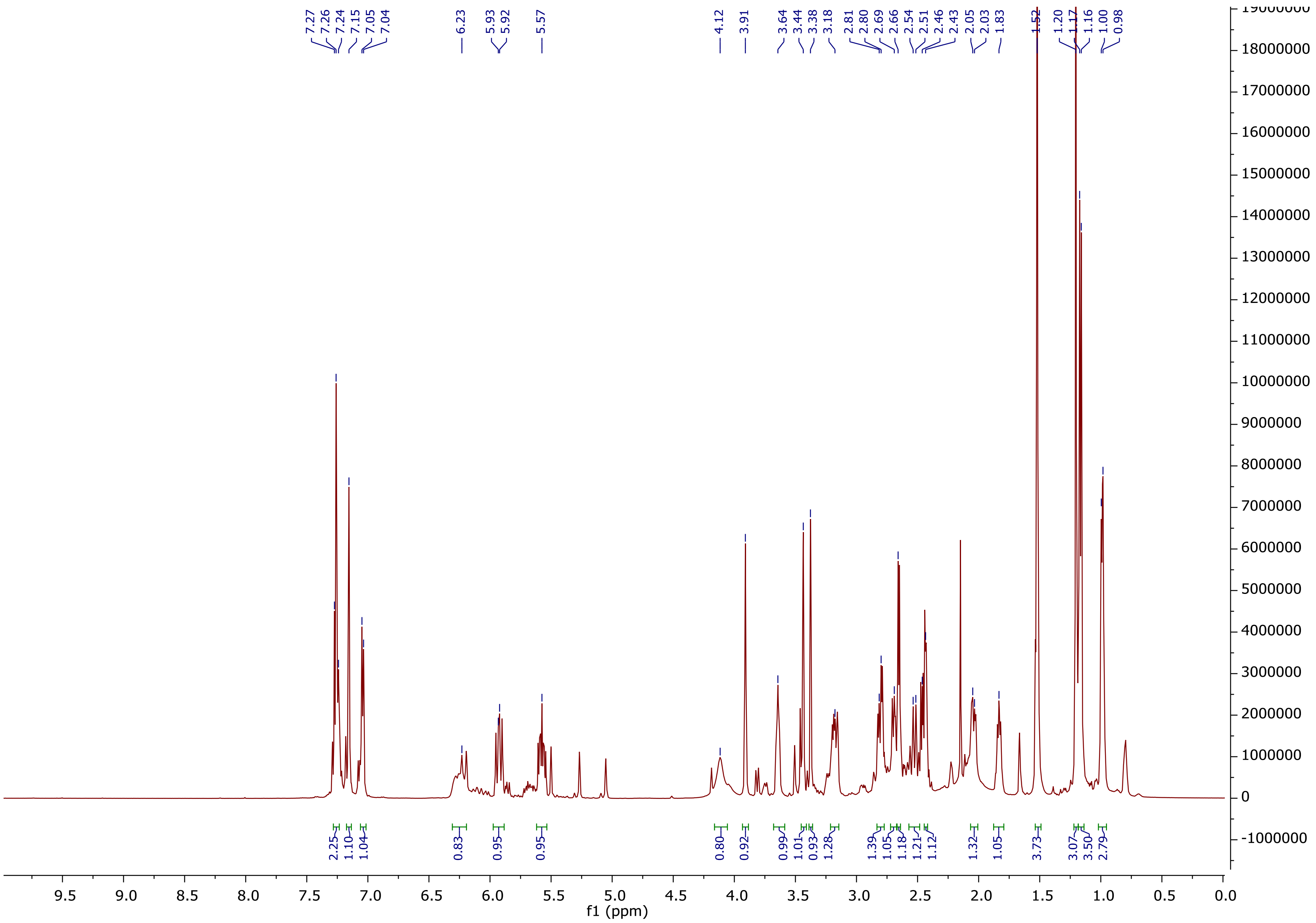

Figure SD100. ^{1}H NMR spectrum of *m*-chloro-deacetyl-19,20-epocycytochalasin Q (**15**) (500 MHz, $CDCl_3$)

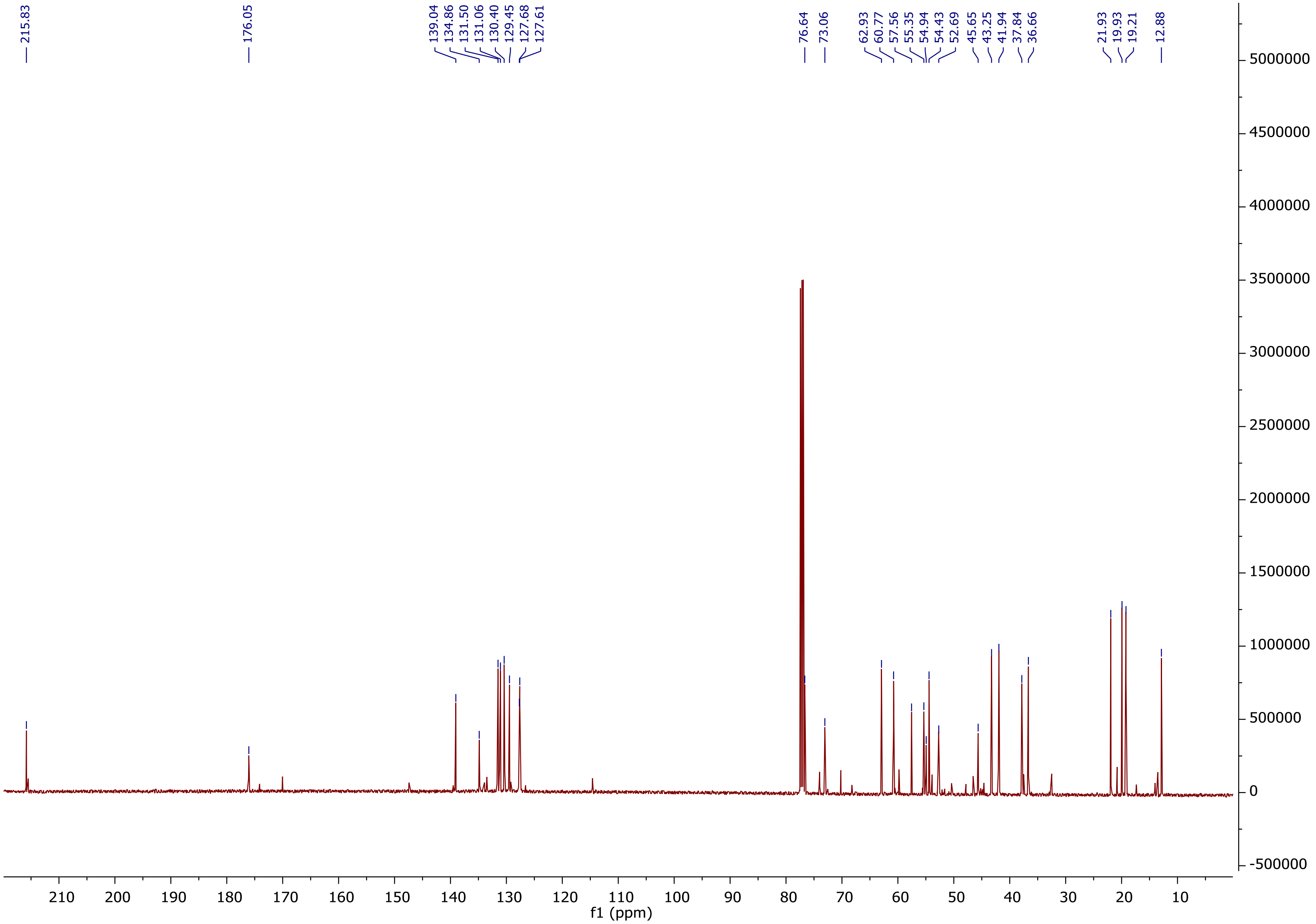

Figure SD101. ^{13}C NMR spectrum of *m*-chloro-deacetyl-19,20-epocycytochalasin Q (**15**) (125 MHz, $CDCl_3$)

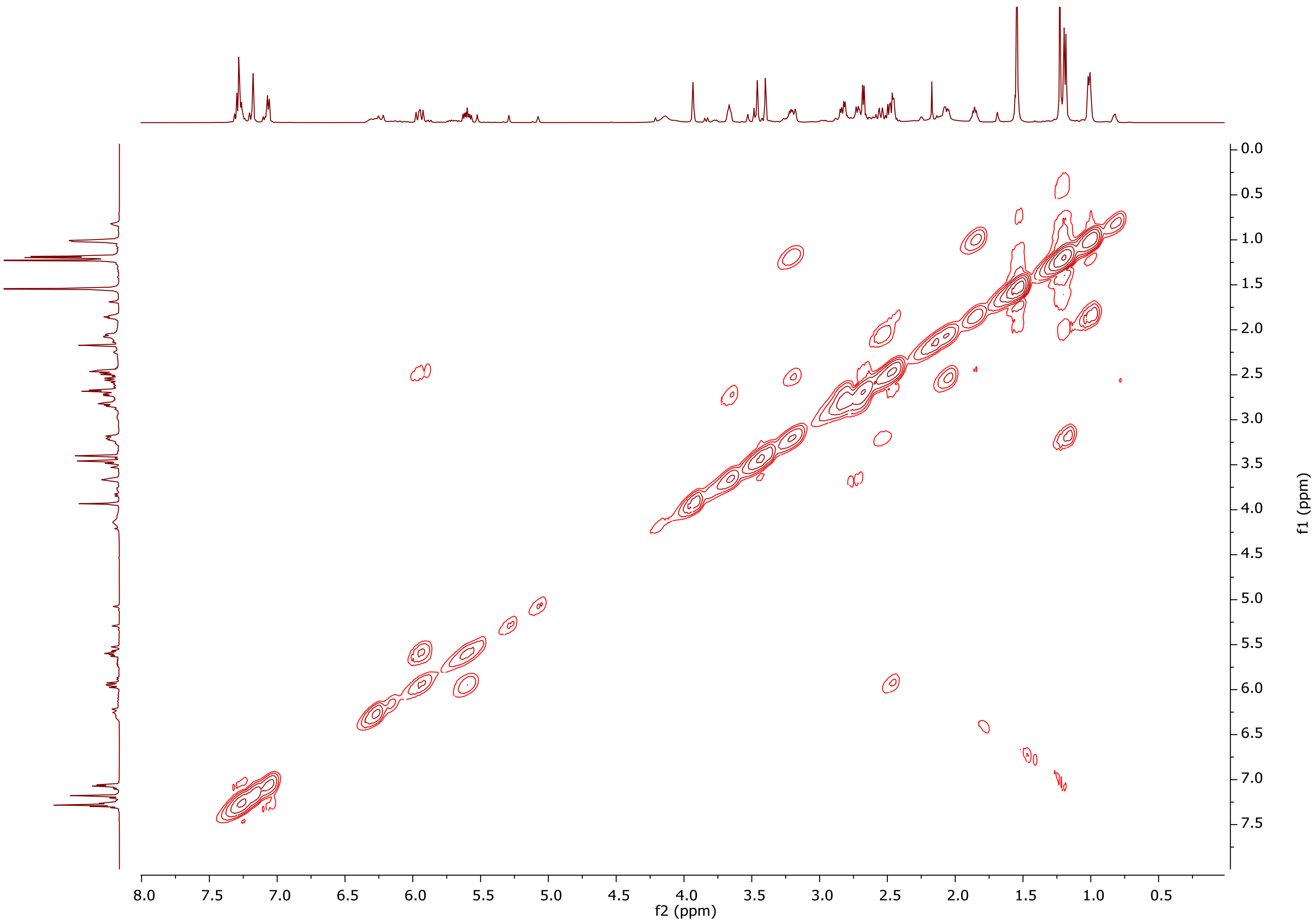

Figure SD102. ^{1}H-^{1}H COSY NMR spectrum of *m*-chloro-deacetyl-19,20-epocycytochalasin Q (**15**) (500/500 MHz, $CDCl_3$)

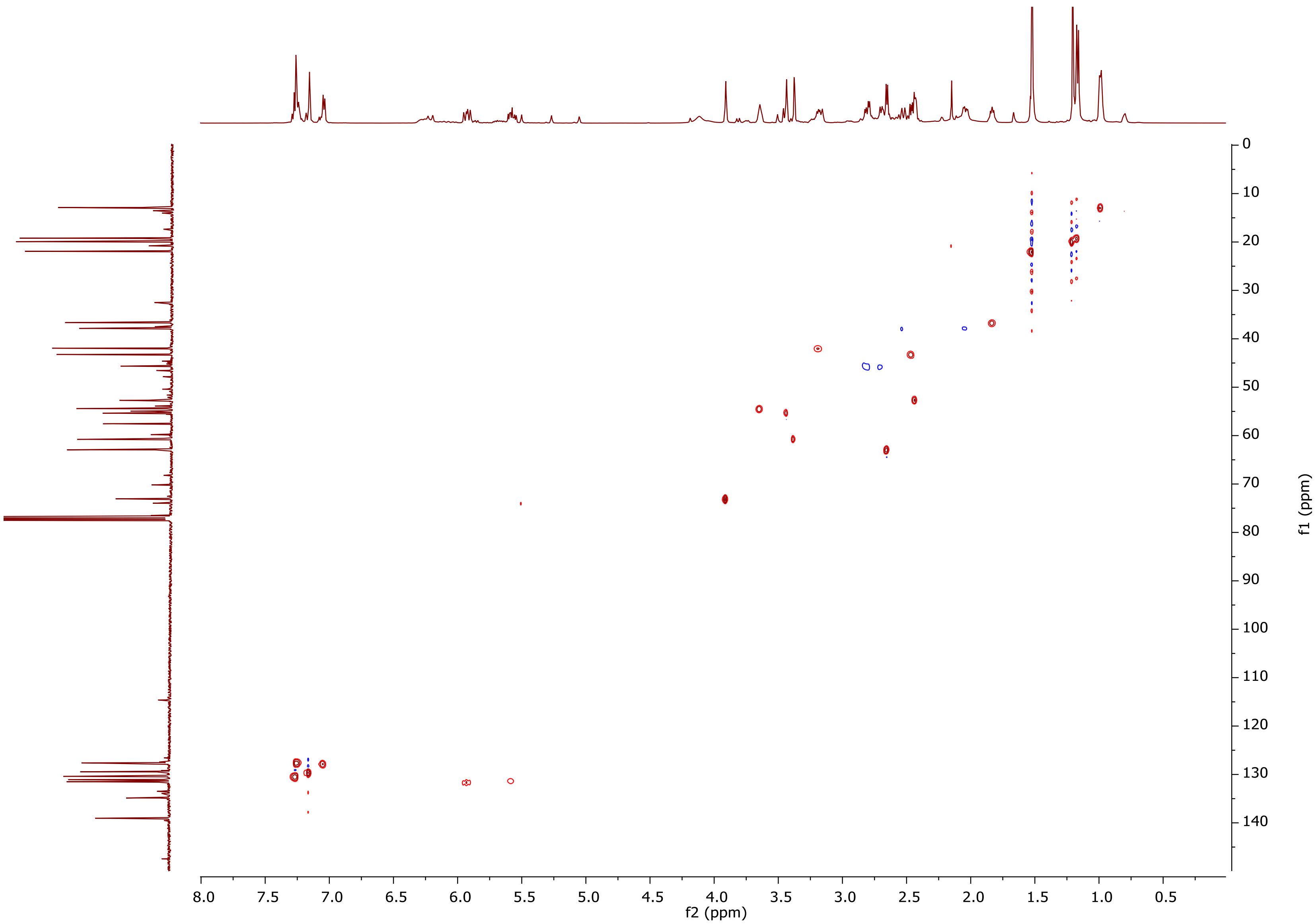

Figure SD103. ^{1}H-^{13}C HSQC NMR spectrum of *m*-chloro-deacetyl-19,20-epocycytochalasin Q (**15**) (500/125 MHz, $CDCl_3$)

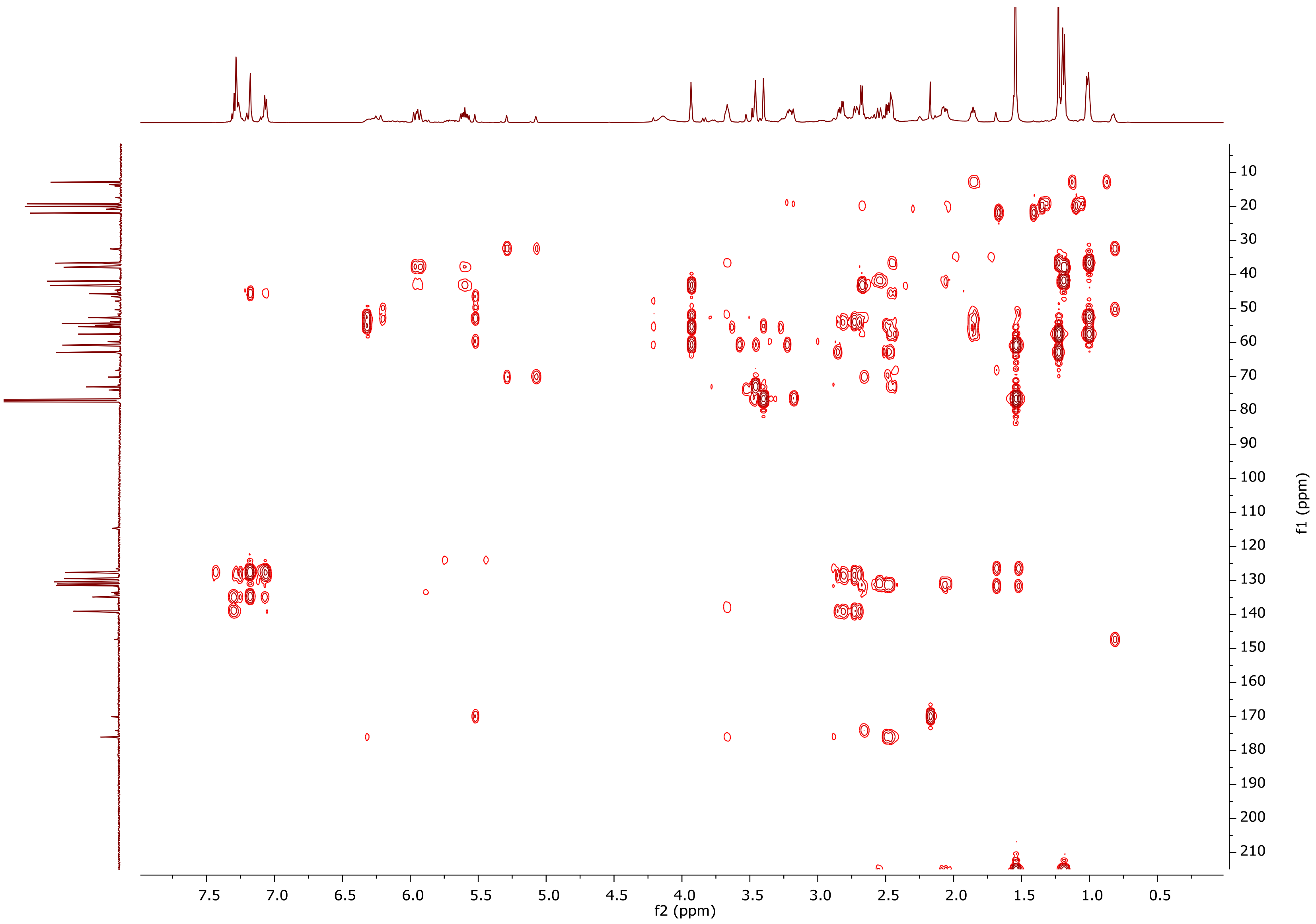

Figure SD104. ^{1}H-^{13}C HMBC NMR spectrum of *m*-chloro-deacetyl-19,20-epocycytochalasin Q (**15**) (500/125 MHz, $CDCl_3$)

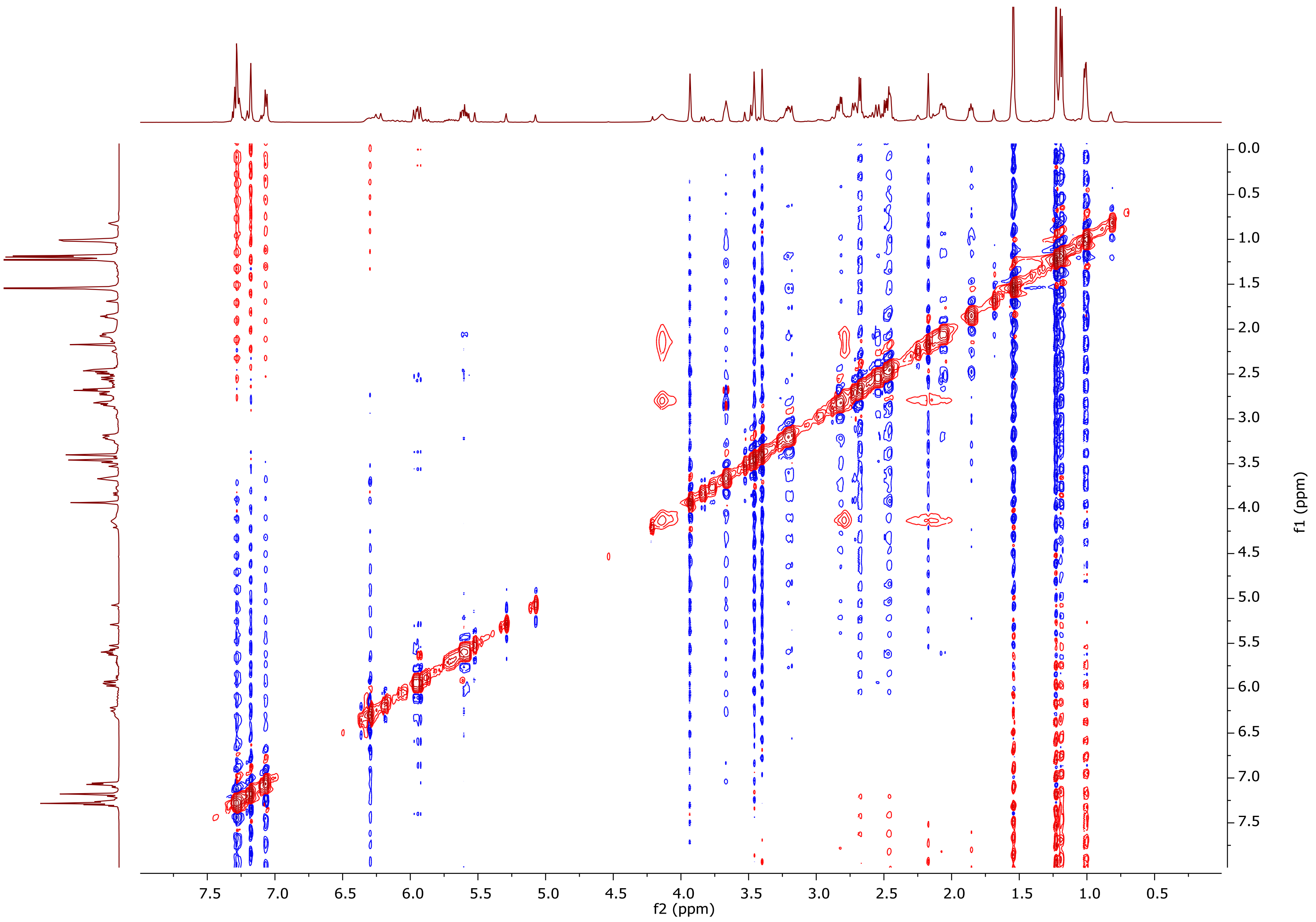

Figure SD105. ^1H-^1H NOESY NMR spectrum of *m*-chloro-deacetyl-19,20-epocycytochalasin Q (**15**) (500/500 MHz, $CDCl_3$)

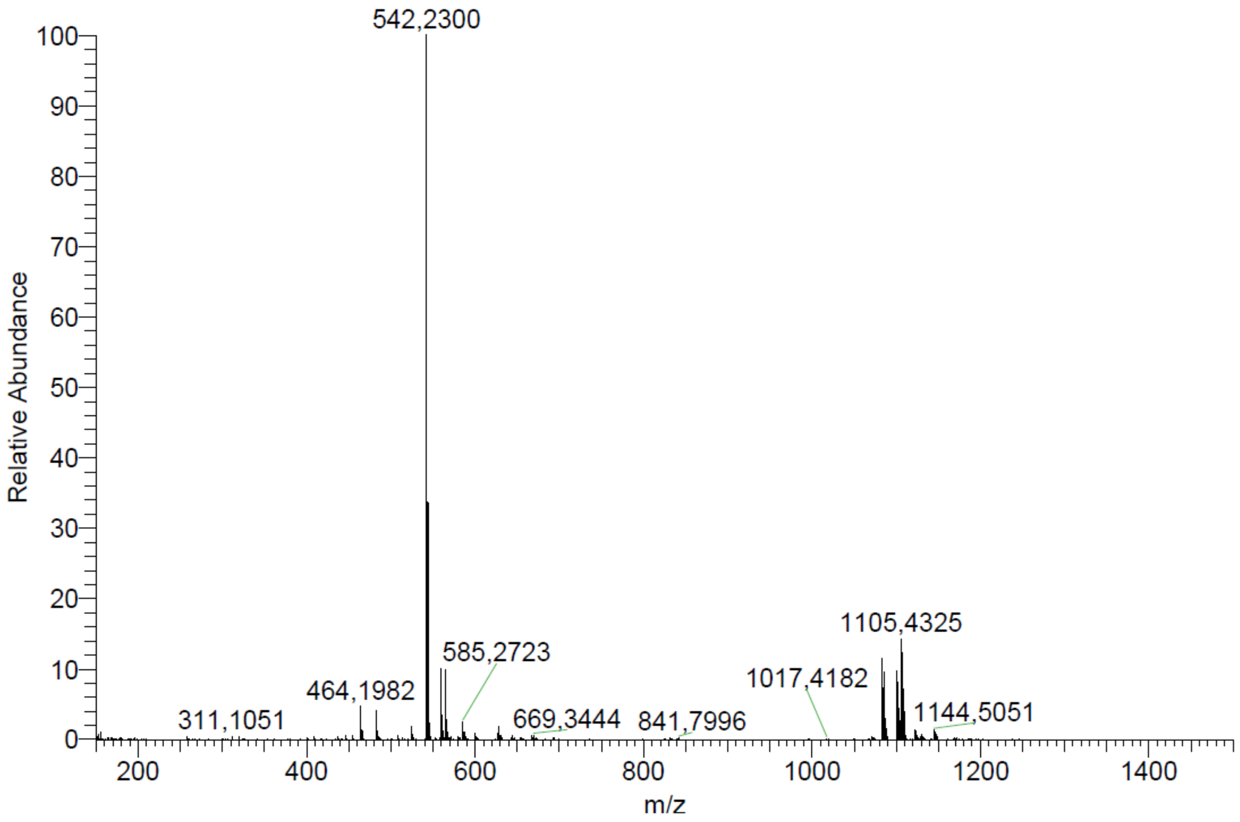

Figure SD106. ESI-HRMS spectrum of *m*-chloro-18-desoxy-19,20-epocycytochalasin Q (**16**)

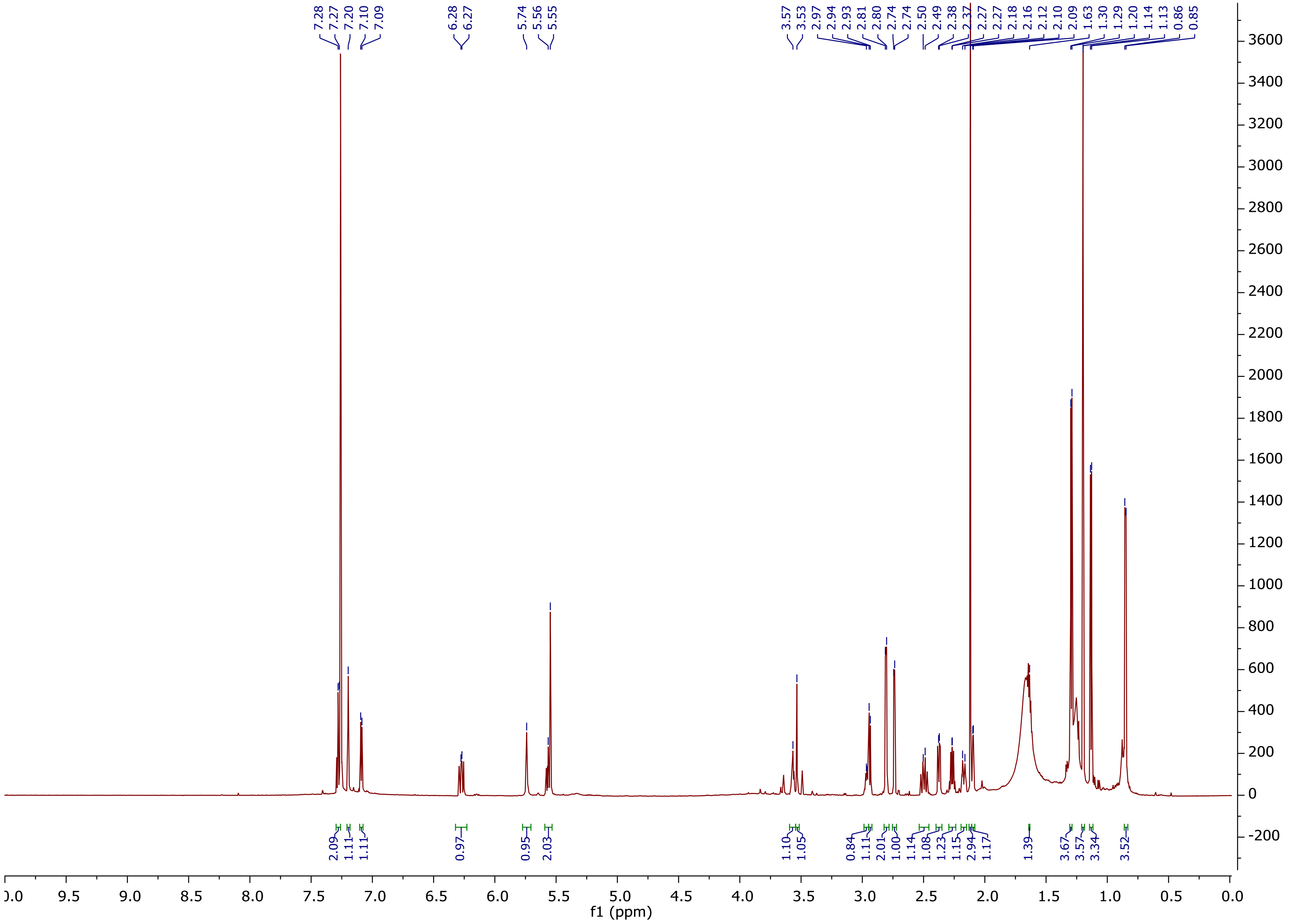

Figure SD107. ^{1}H NMR spectrum of *m*-chloro-18-desoxy-19,20-epocycytochalasin Q (**16**) (500 MHz, $CDCl_3$)

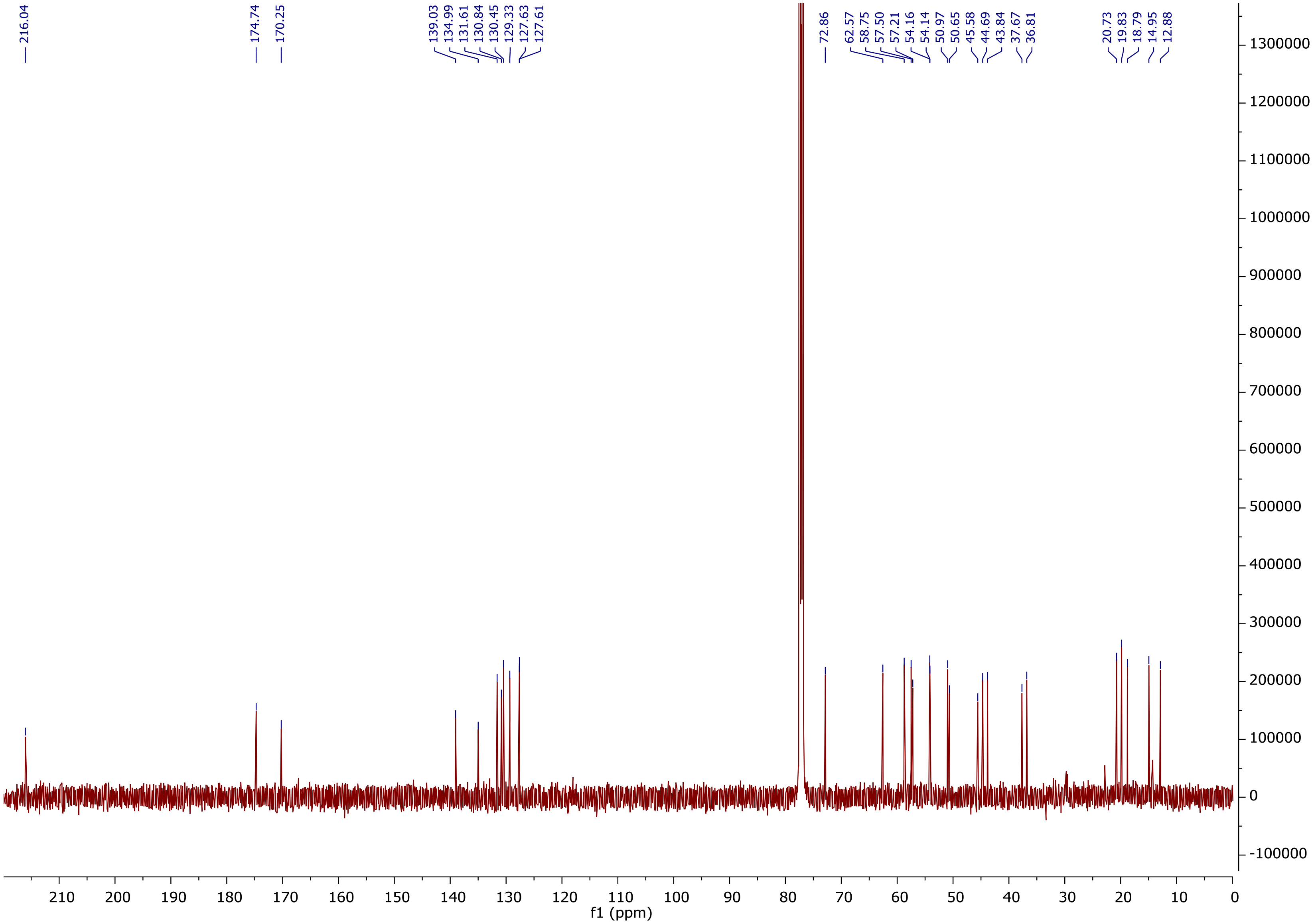

Figure SD108. ^{13}C NMR spectrum of *m*-chloro-18-desoxy-19,20-epocycytochalasin Q (**16**) (125 MHz, $CDCl_3$)

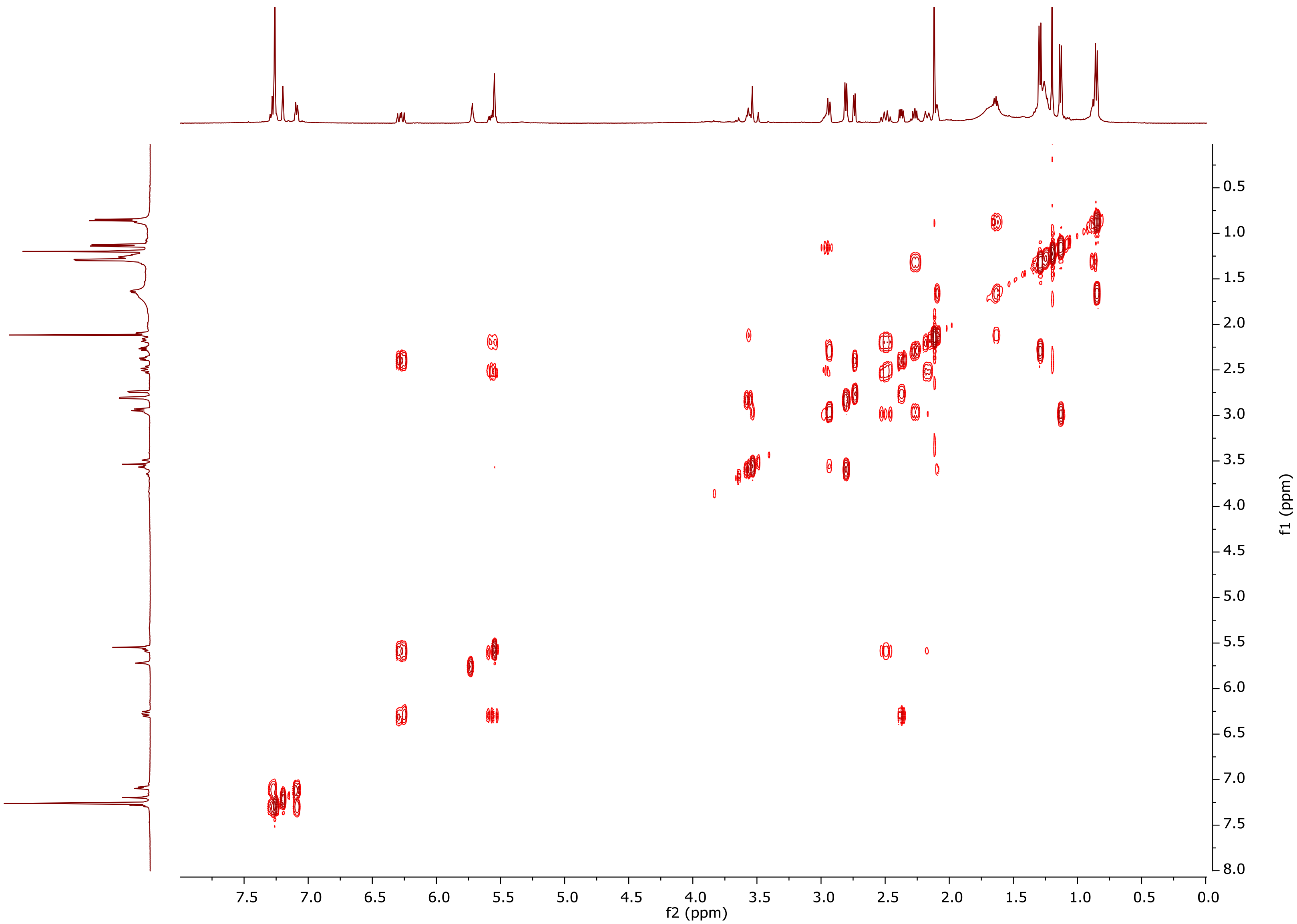

Figure SD109. ^{1}H-^{1}H COSY NMR spectrum of *m*-chloro-18-desoxy-19,20-epocycytochalasin Q (**16**) (500/500 MHz, $CDCl_3$)

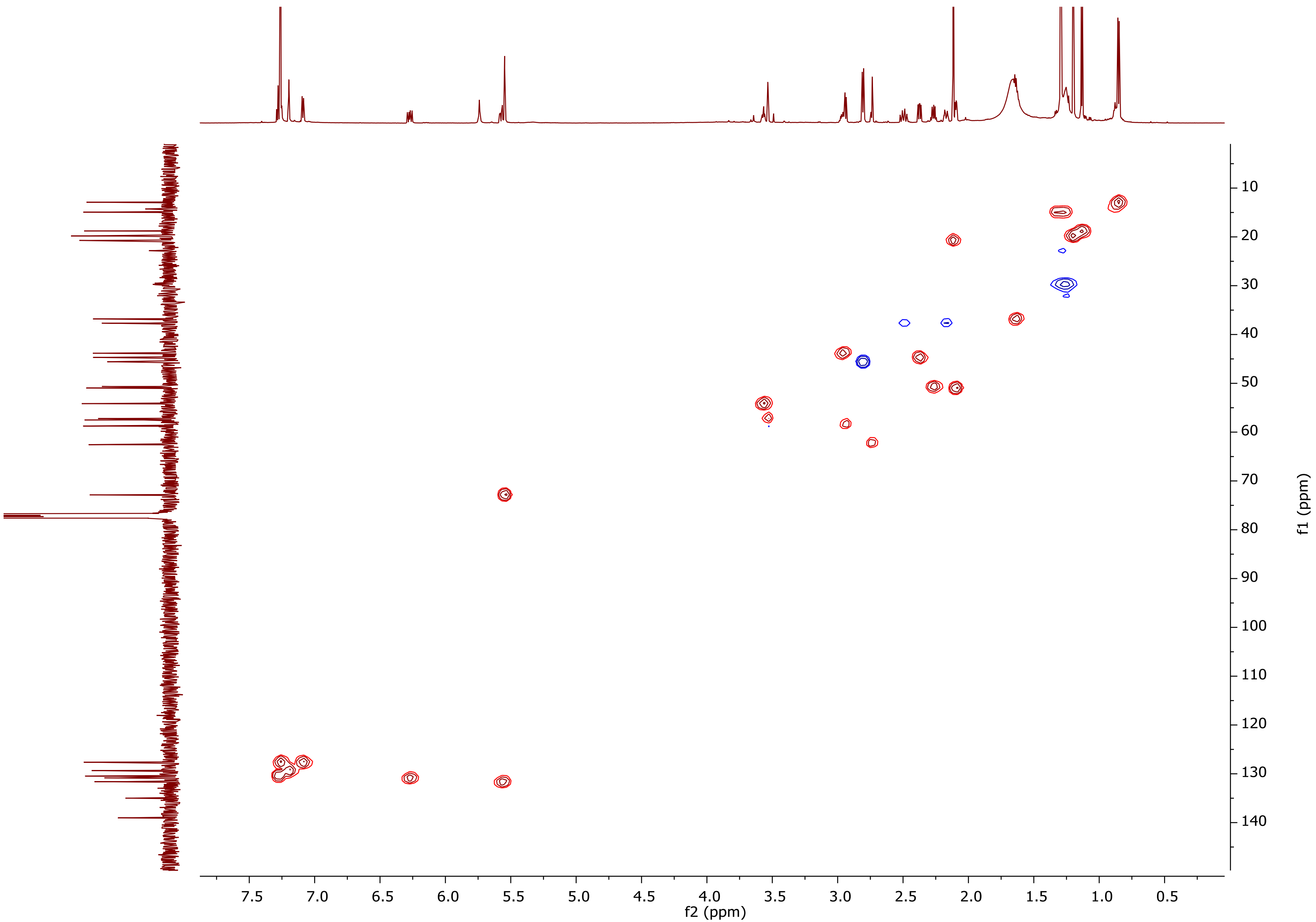

Figure SD110. ^{1}H-^{13}C HSQC NMR spectrum of *m*-chloro-18-desoxy-19,20-epocycytochalasin Q (**16**) (500/125 MHz, $CDCl_3$)

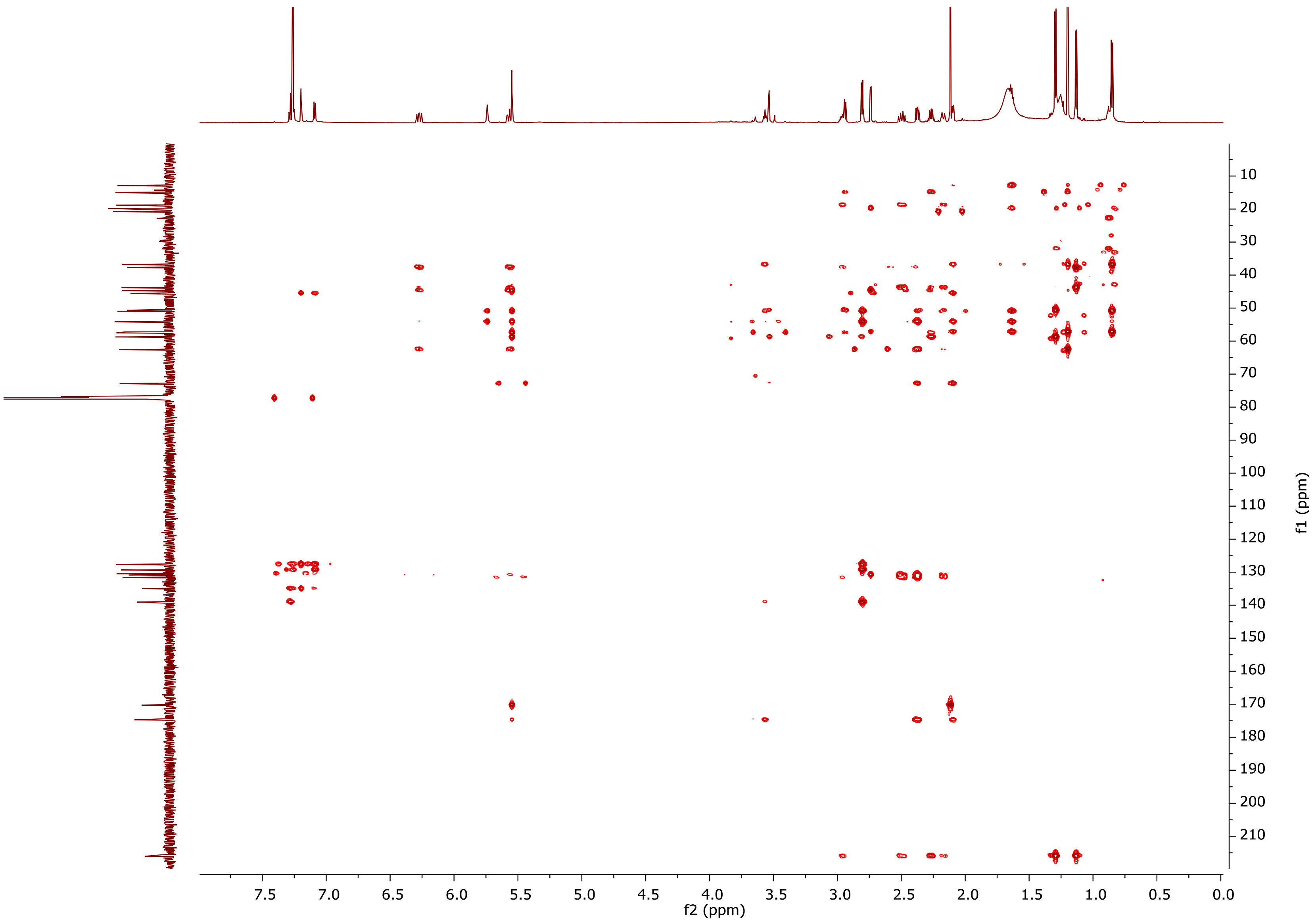

Figure SD111. ^{1}H-^{13}C HMBC NMR spectrum of *m*-chloro-18-desoxy-19,20-epocycytochalasin Q (**16**) (500/125 MHz, $CDCl_3$)

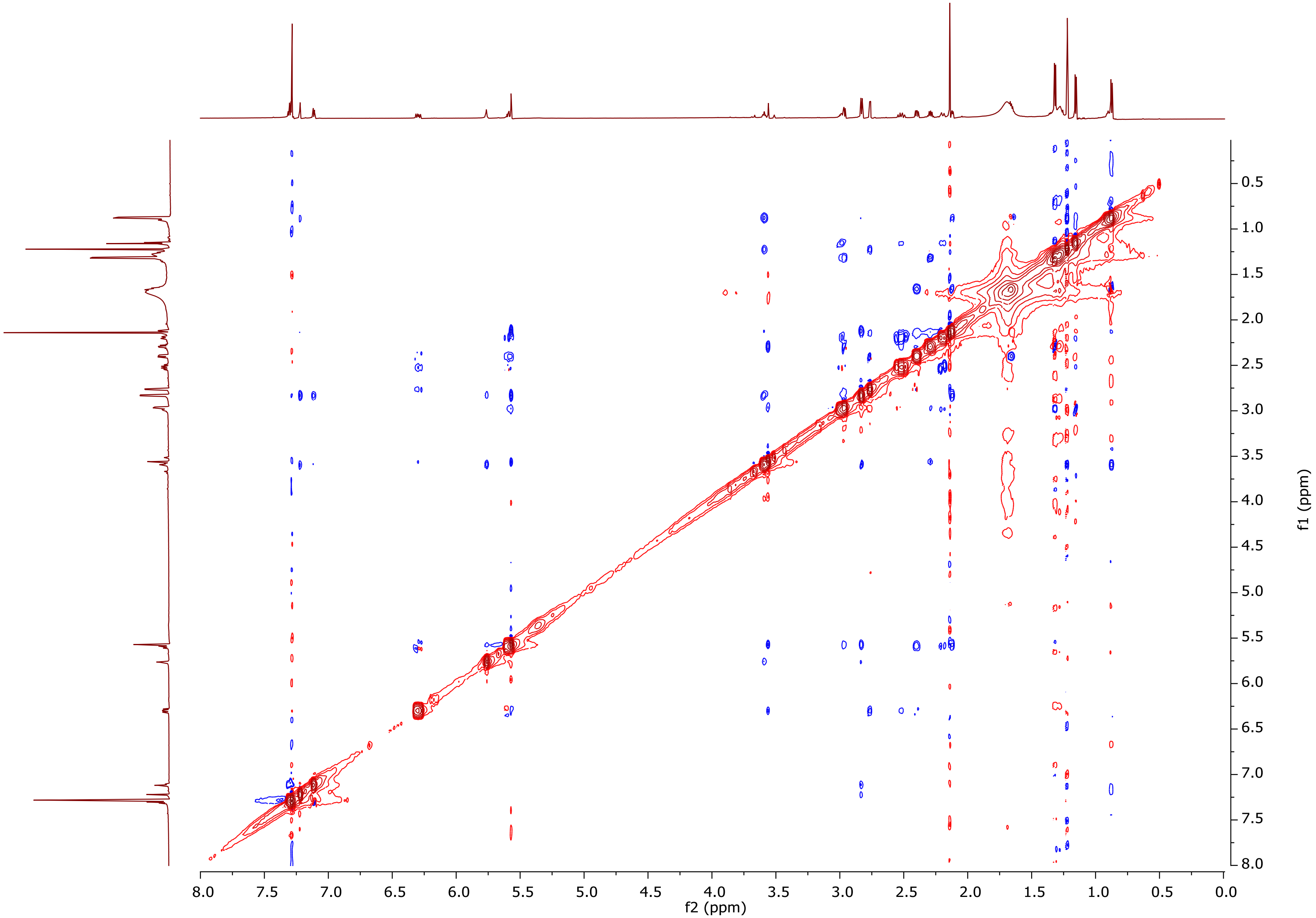

Figure SD112. ^{1}H-^{1}H NOESY NMR spectrum of *m*-chloro-18-desoxy-19,20-epocycytochalasin Q (**16**) (500/500 MHz, $CDCl_3$)

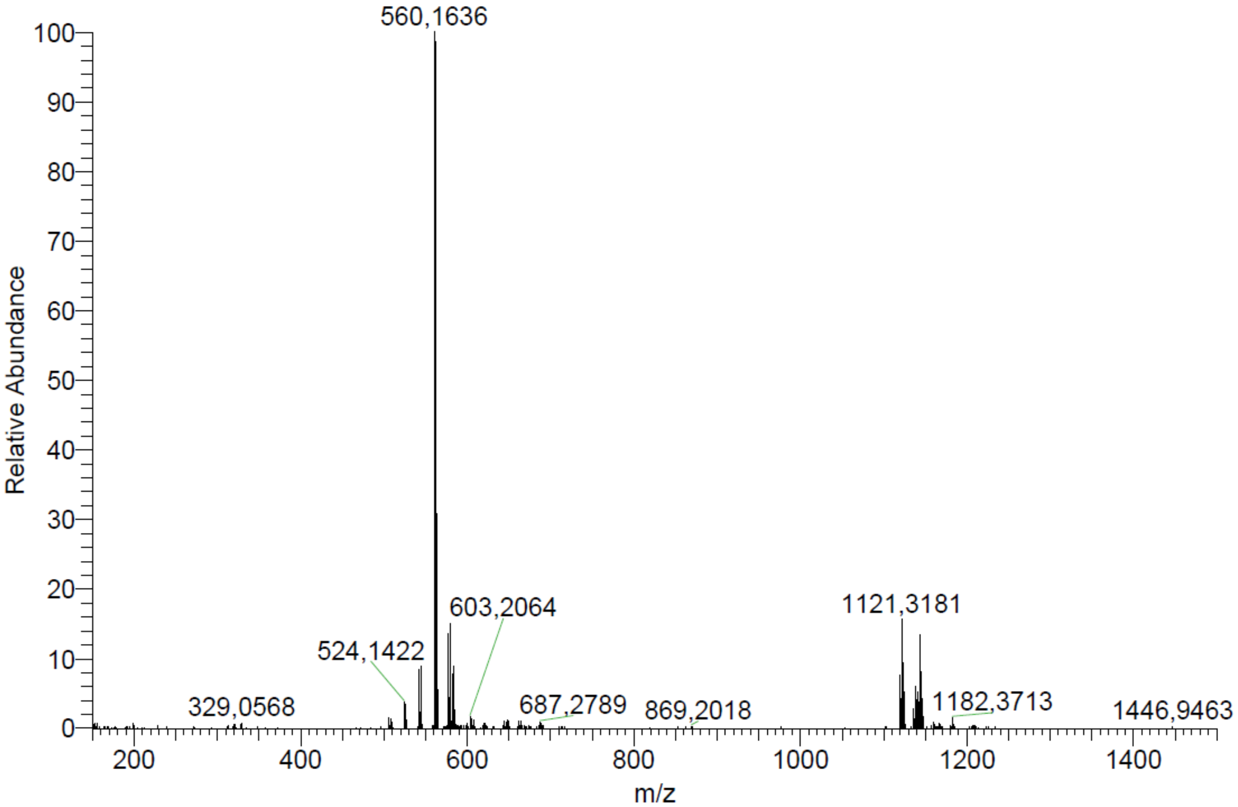

Figure SD113. ESI-HRMS spectrum of *m*-bromo-deacetyl-19,20-epocycytochalasin C (**17**)

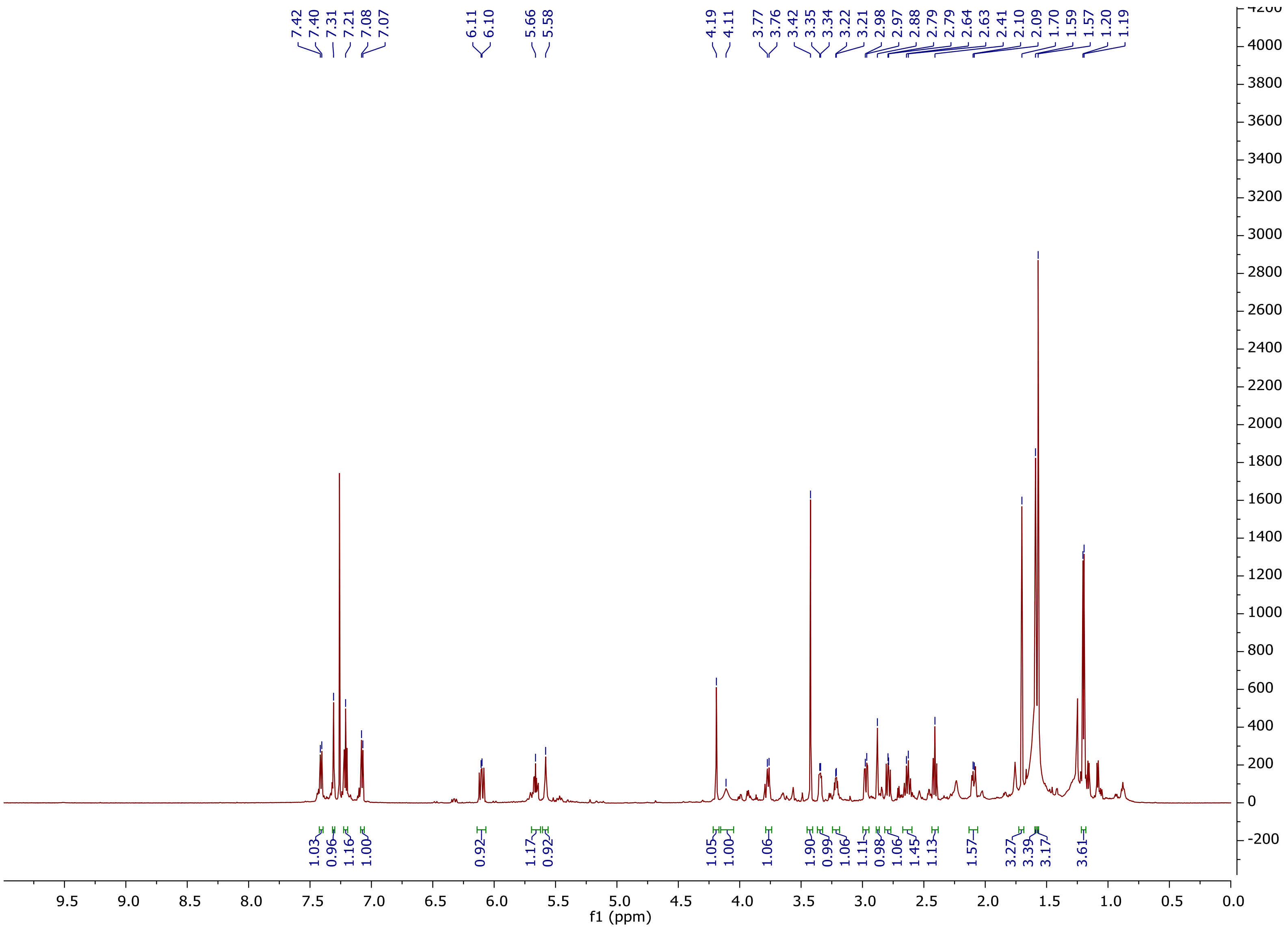

Figure SD114. ^{1}H NMR spectrum of *m*-bromo-deacetyl-19,20-epocycytochalasin C (**17**) (700 MHz, $CDCl_3$)

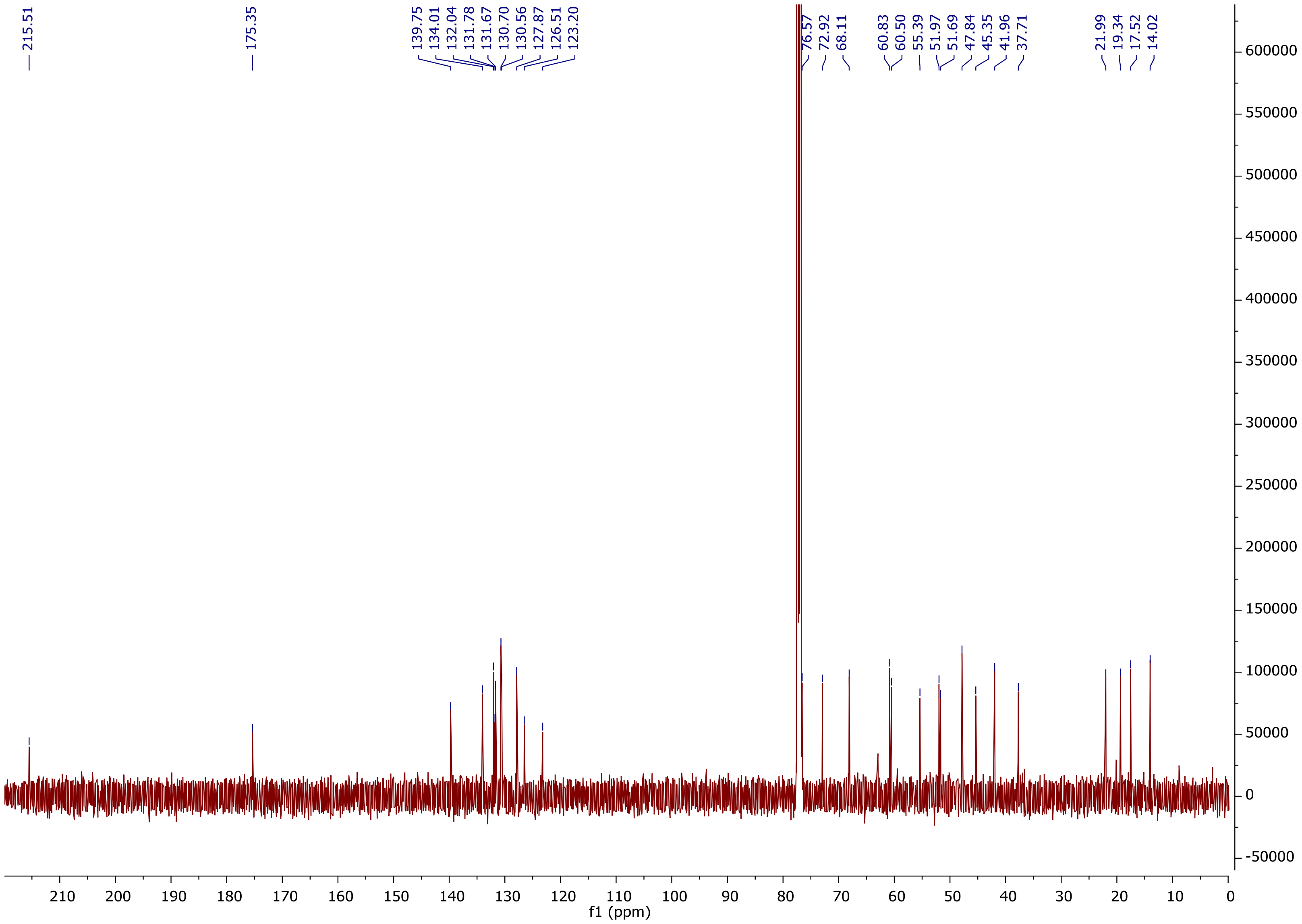

Figure SD115. ^{13}C NMR spectrum of *m*-bromo-deacetyl-19,20-epocycytochalasin C (**17**) (175 MHz, $CDCl_3$)

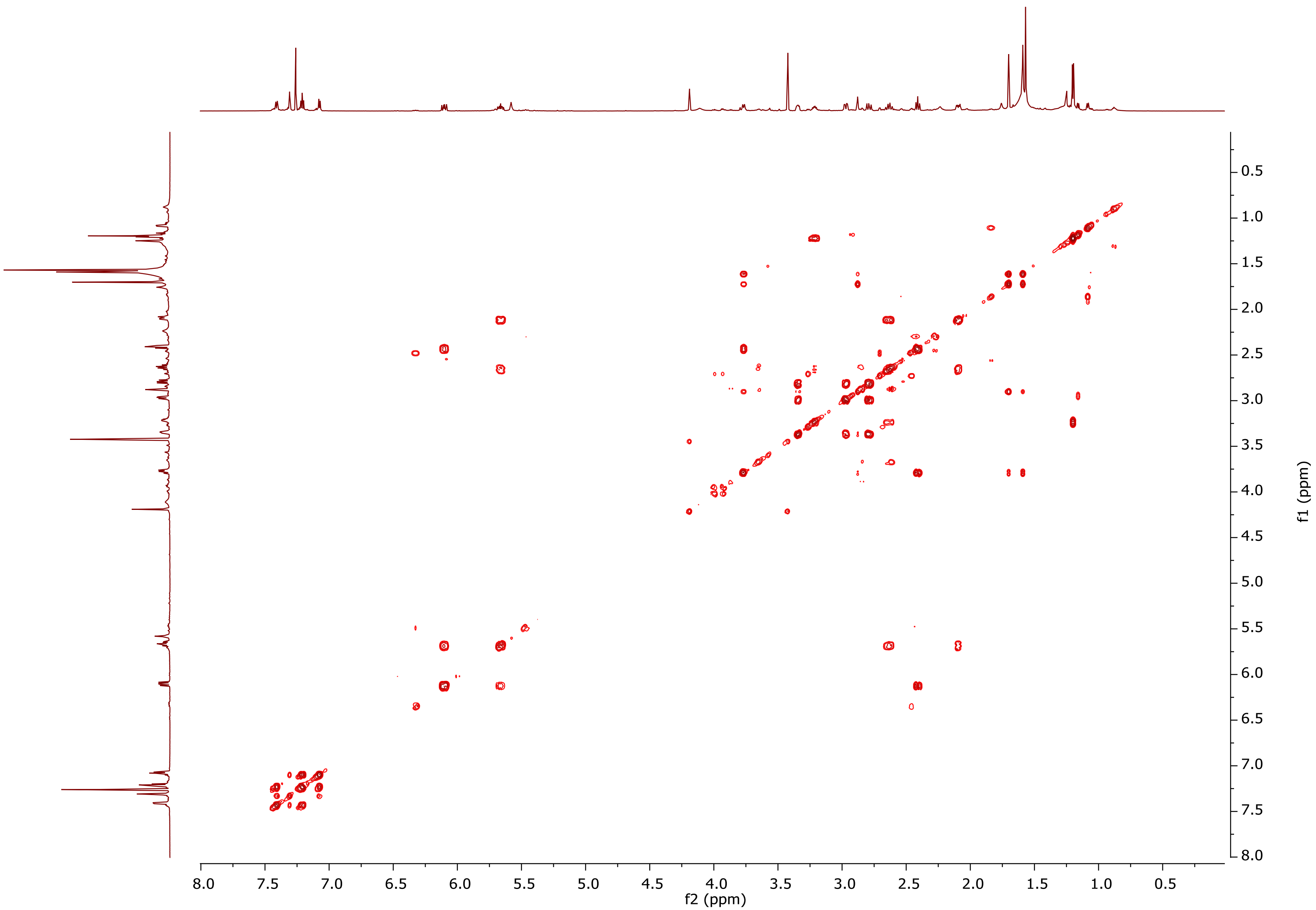

Figure SD116. ^{1}H-^{1}H COSY NMR spectrum of *m*-bromo-deacetyl-19,20-epocycytochalasin C (**17**) (700/700 MHz, $CDCl_3$)

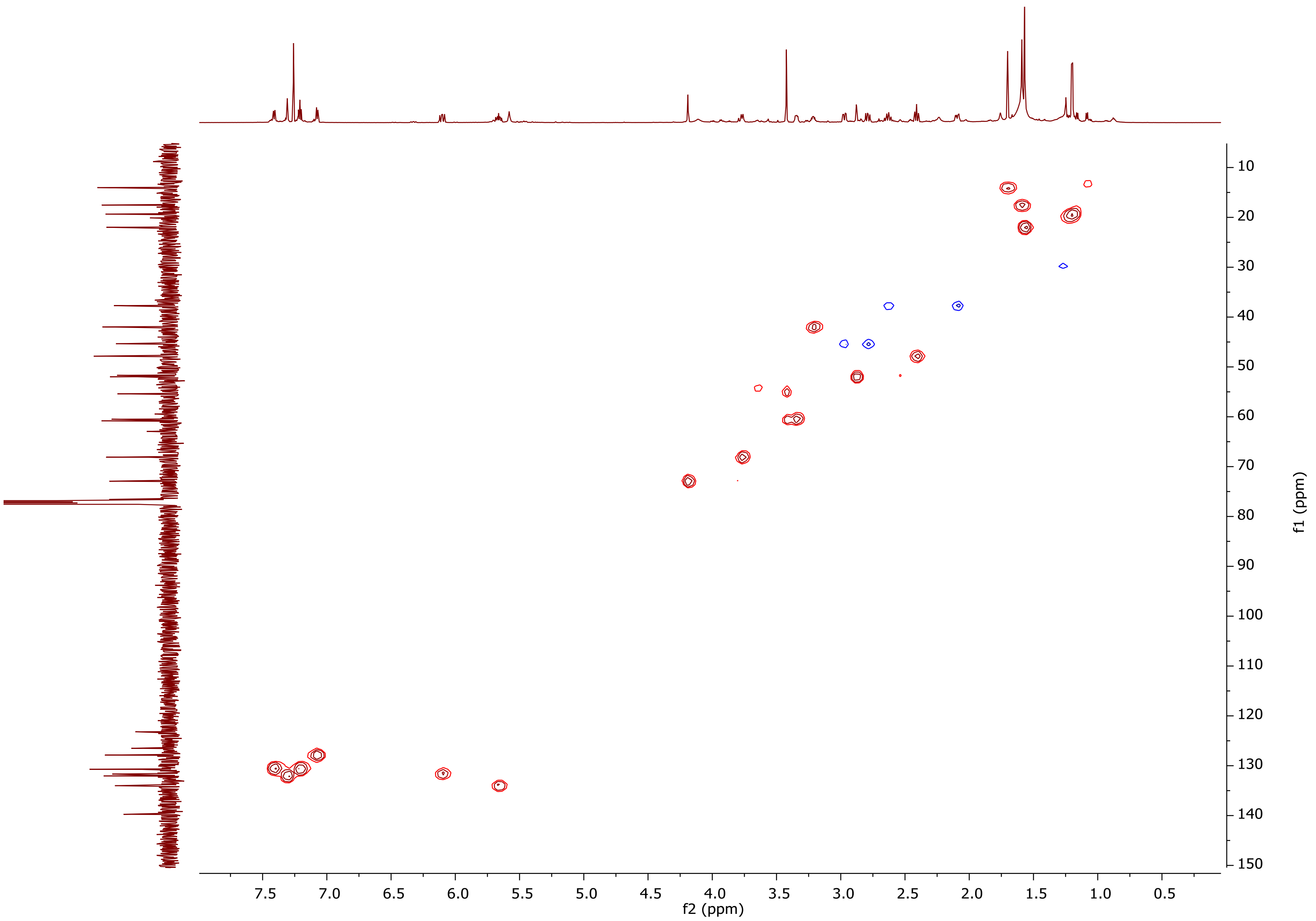

Figure SD117. ^{1}H-^{13}C HSQC NMR spectrum of *m*-bromo-deacetyl-19,20-epocycytochalasin C (**17**) (700/175 MHz, $CDCl_3$)

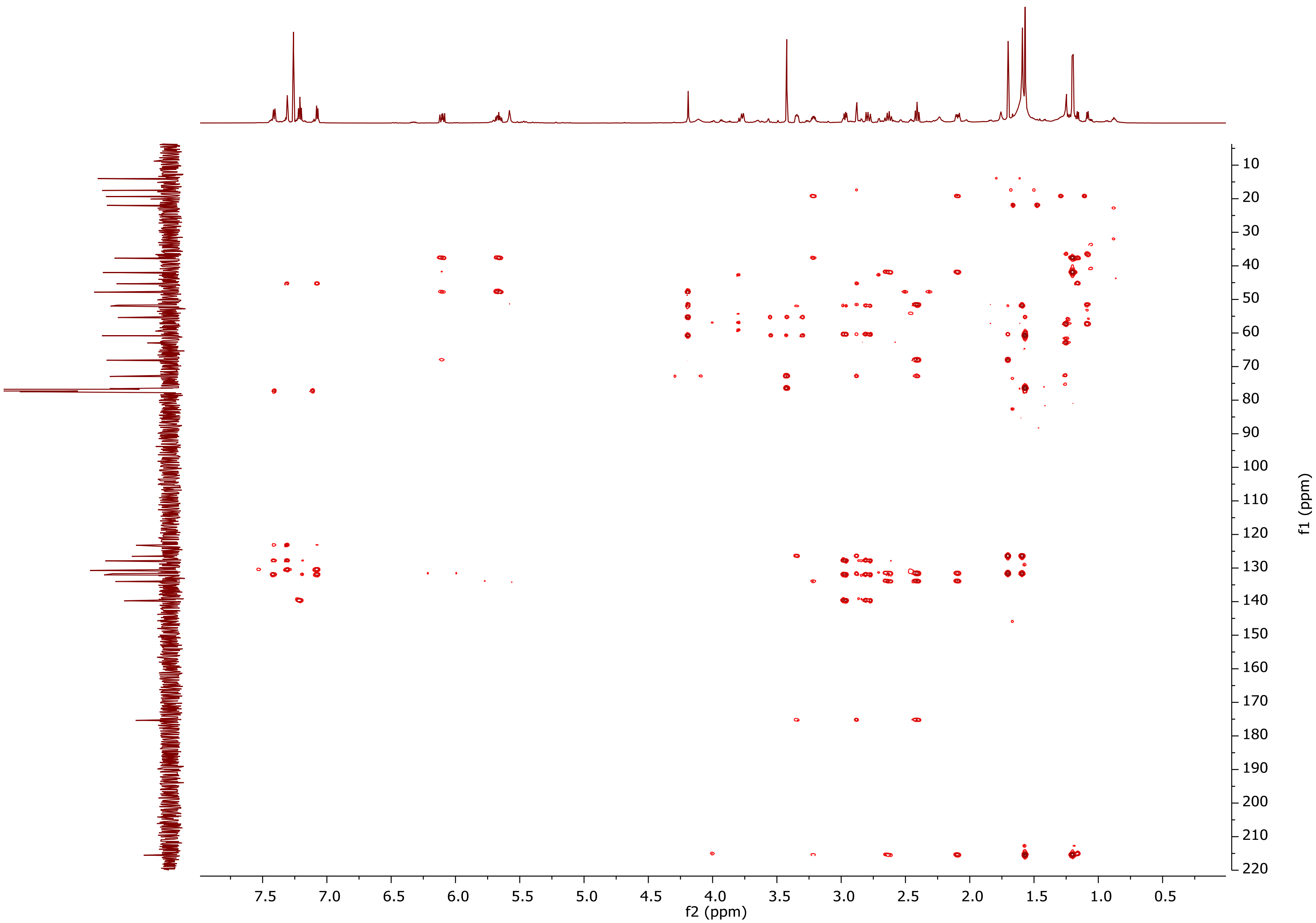

Figure SD118. ^{1}H-^{13}C HMBC NMR spectrum of *m*-bromo-deacetyl-19,20-epocycytochalasin C (**17**) (700/175 MHz, $CDCl_3$)

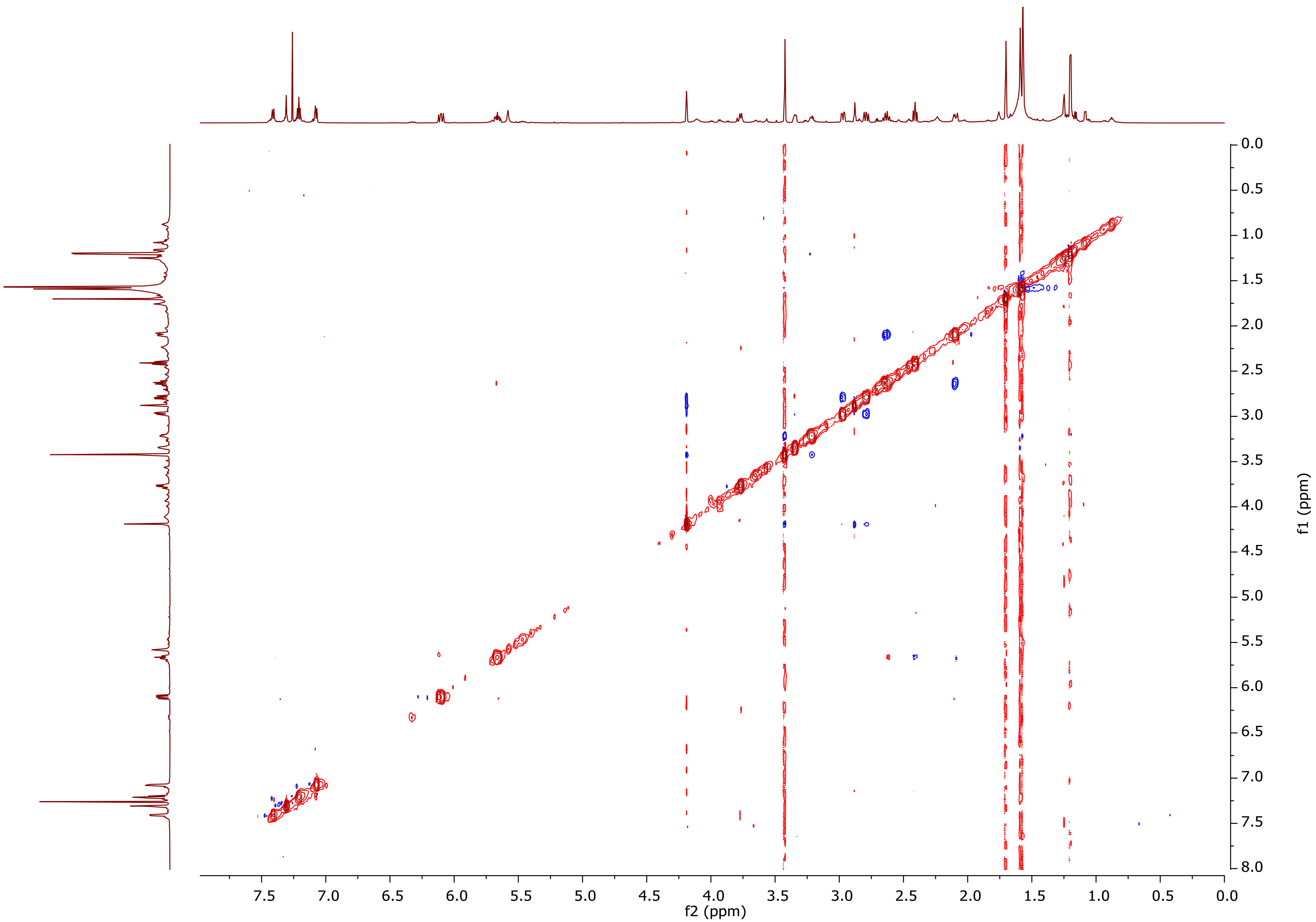

Figure SD119. ^1H-^1H NOESY NMR spectrum of *m*-bromo-deacetyl-19,20-epocycytochalasin C (**17**) (700/700 MHz, $CDCl_3$)

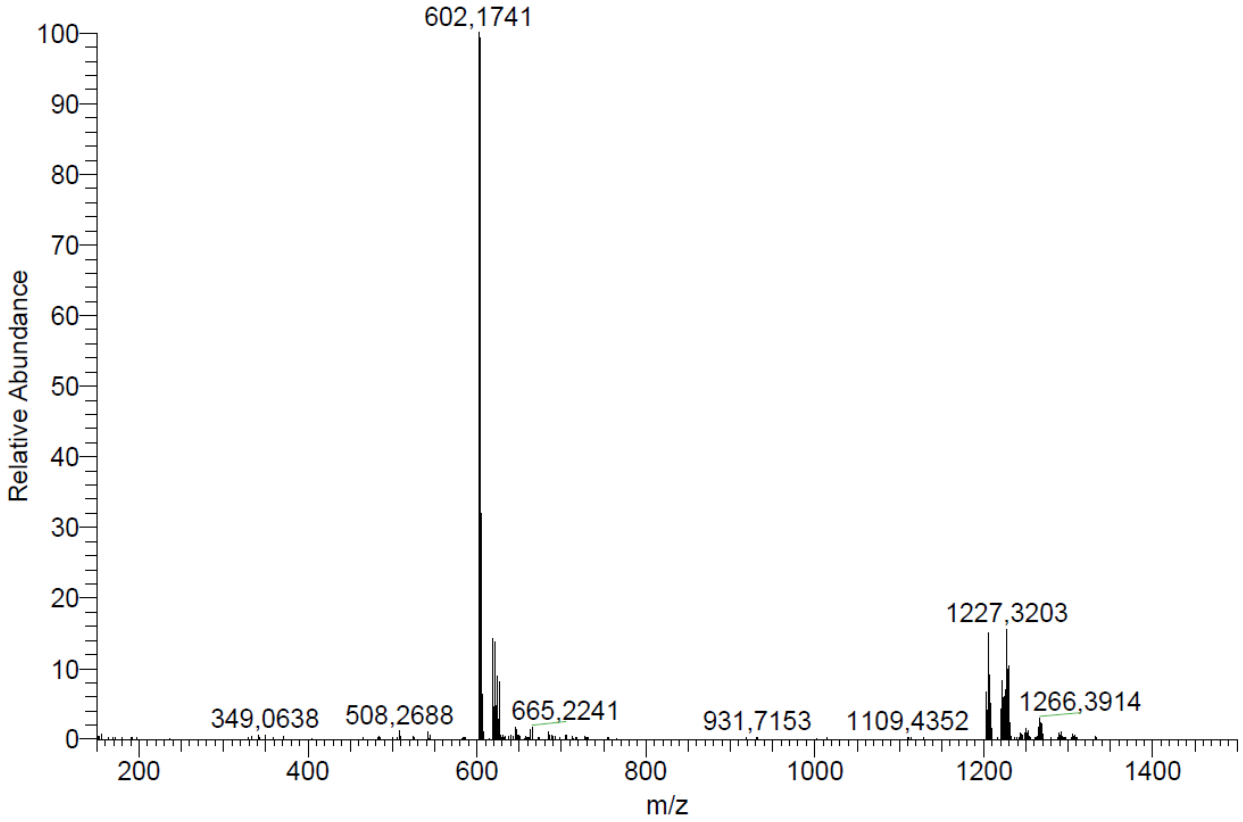

Figure SD120. ESI-HRMS spectrum of *m*-bromo-19,20-epocycytochalasin Q (**18**)

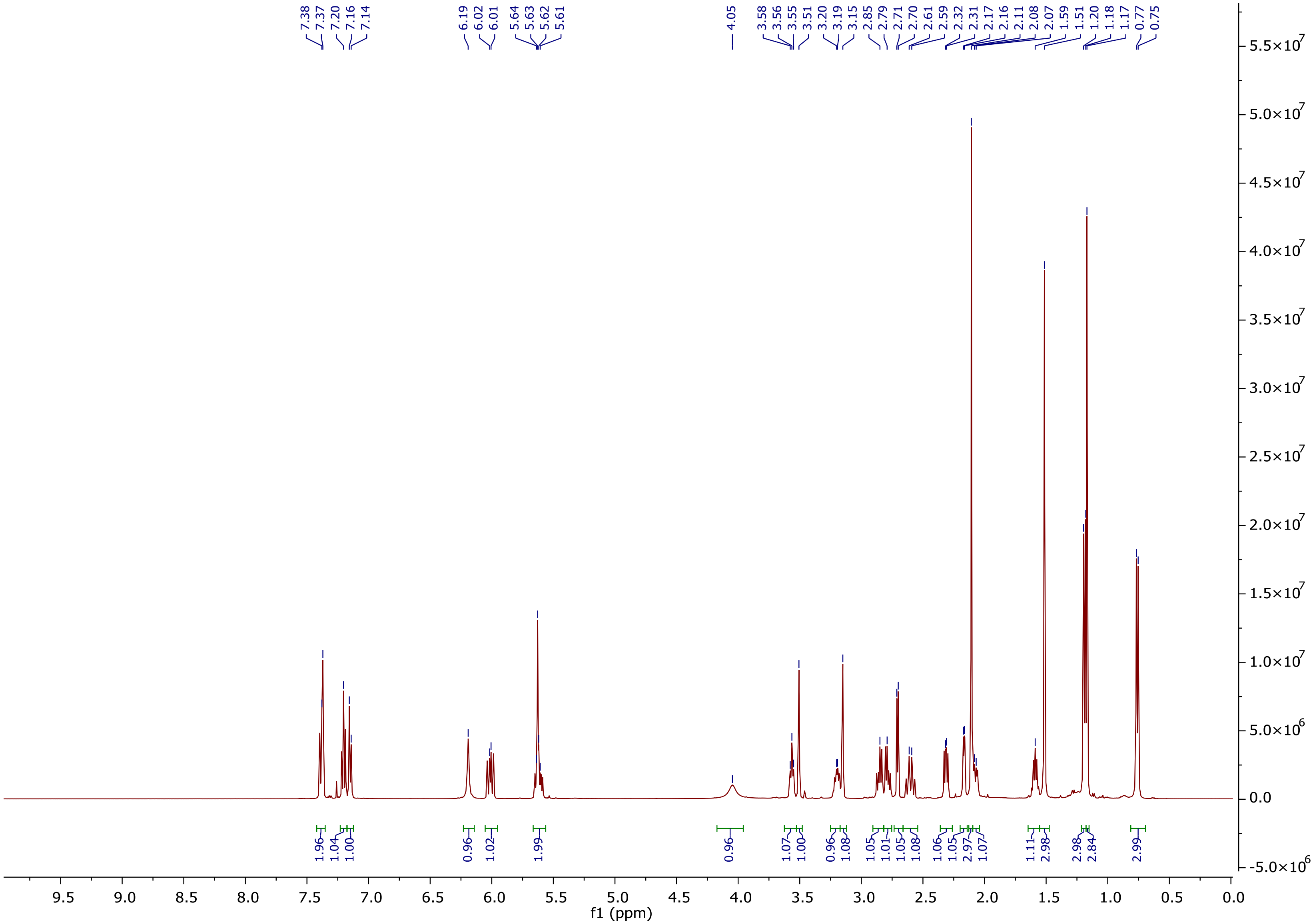

Figure SD121. ^{1}H NMR spectrum of *m*-bromo-19,20-epocycytochalasin Q (**18**) (500 MHz, $CDCl_3$)

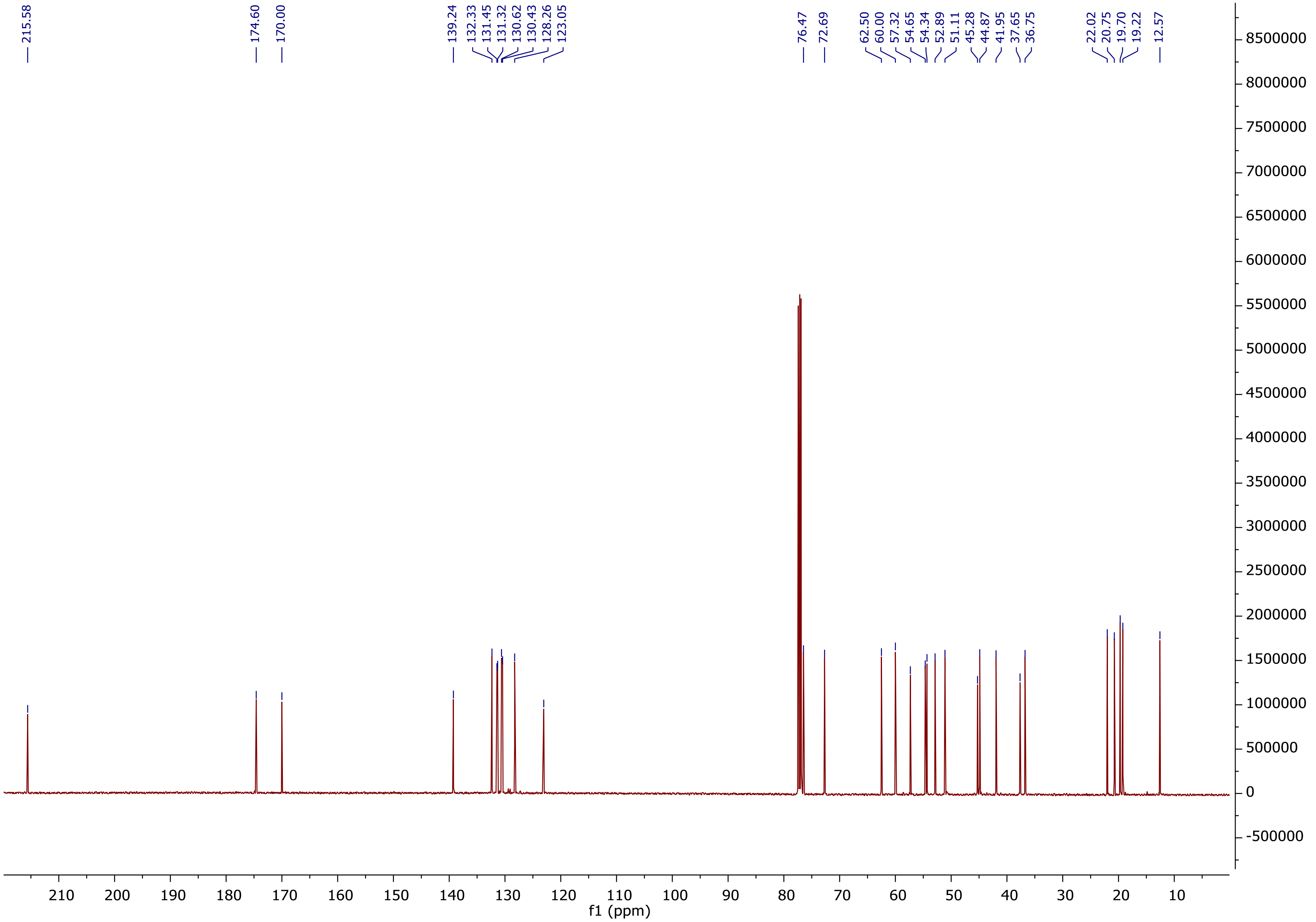

Figure SD122. ^{13}C NMR spectrum of *m*-bromo-19,20-epocycytochalasin Q (**18**) (125 MHz, $CDCl_3$)

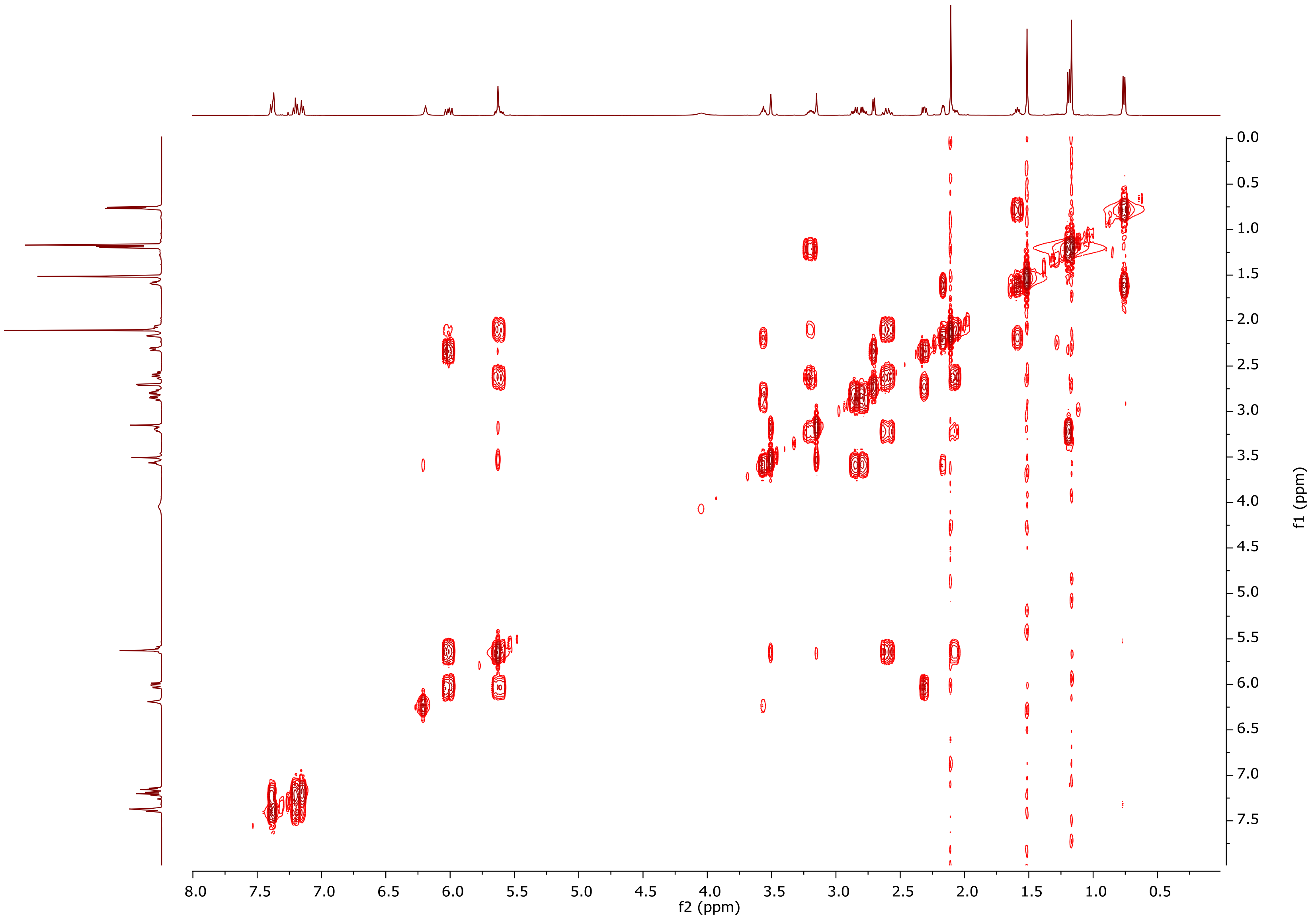

Figure SD123. ^{1}H-^{1}H COSY NMR spectrum of *m*-bromo-19,20-epocycytochalasin Q (**18**) (500/500 MHz, $CDCl_3$)

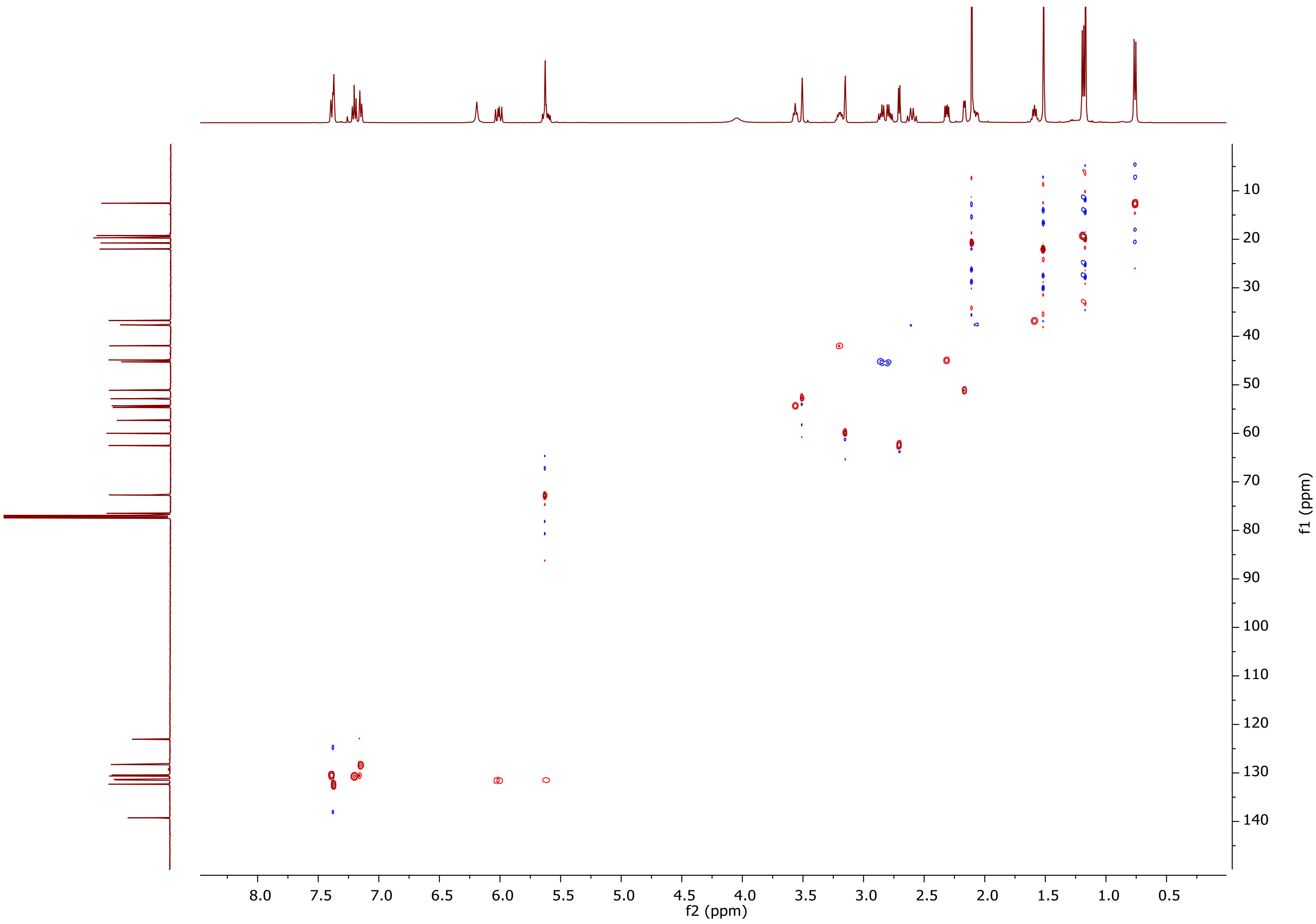

Figure SD124. ^{1}H-^{13}C HSQC NMR spectrum of *m*-bromo-19,20-epocycytochalasin Q (**18**) (500/125 MHz, $CDCl_3$)

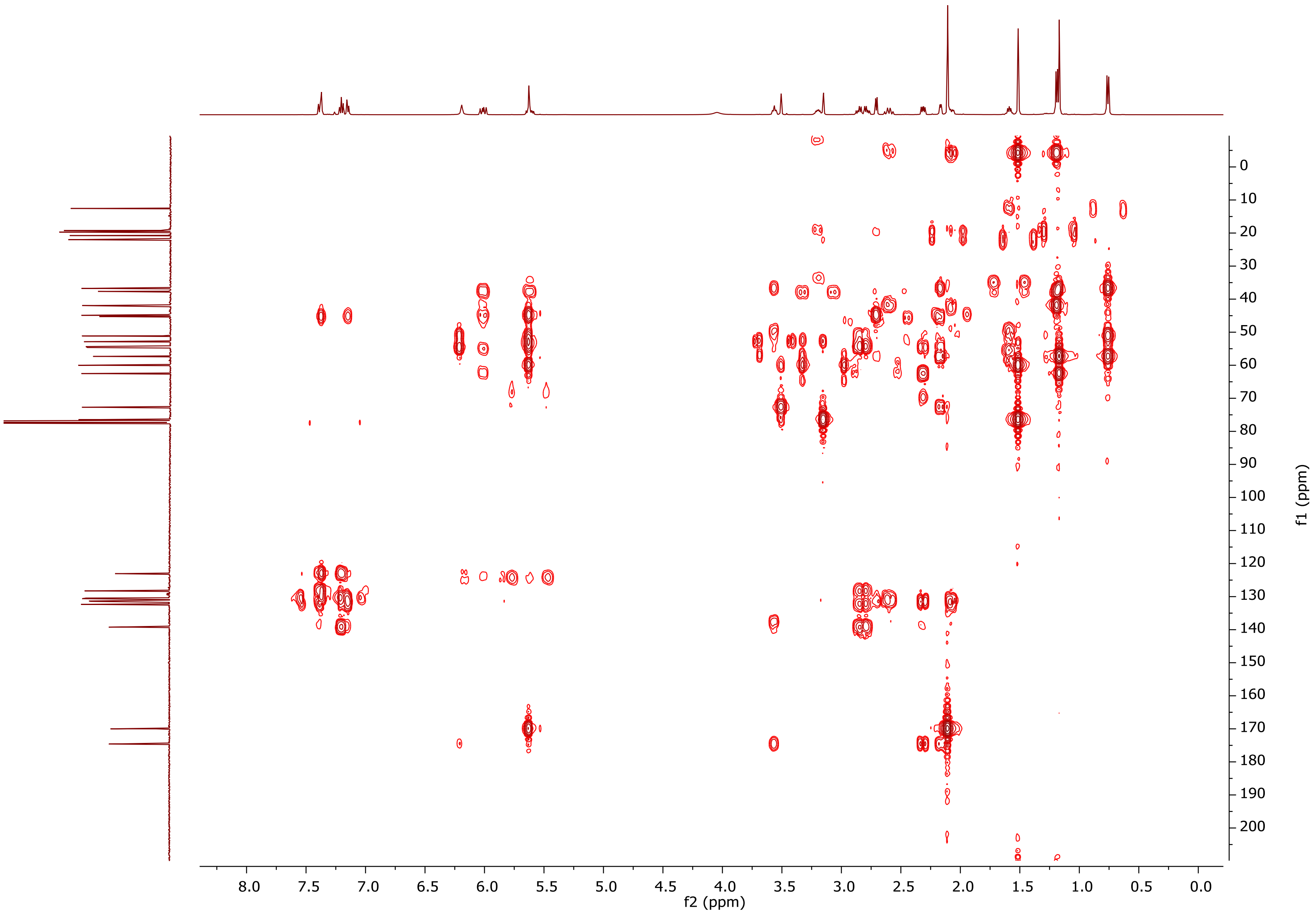

Figure SD125. ^{1}H-^{13}C HMBC NMR spectrum of *m*-bromo-19,20-epocycytochalasin Q (**18**) (500/125 MHz, $CDCl_3$)

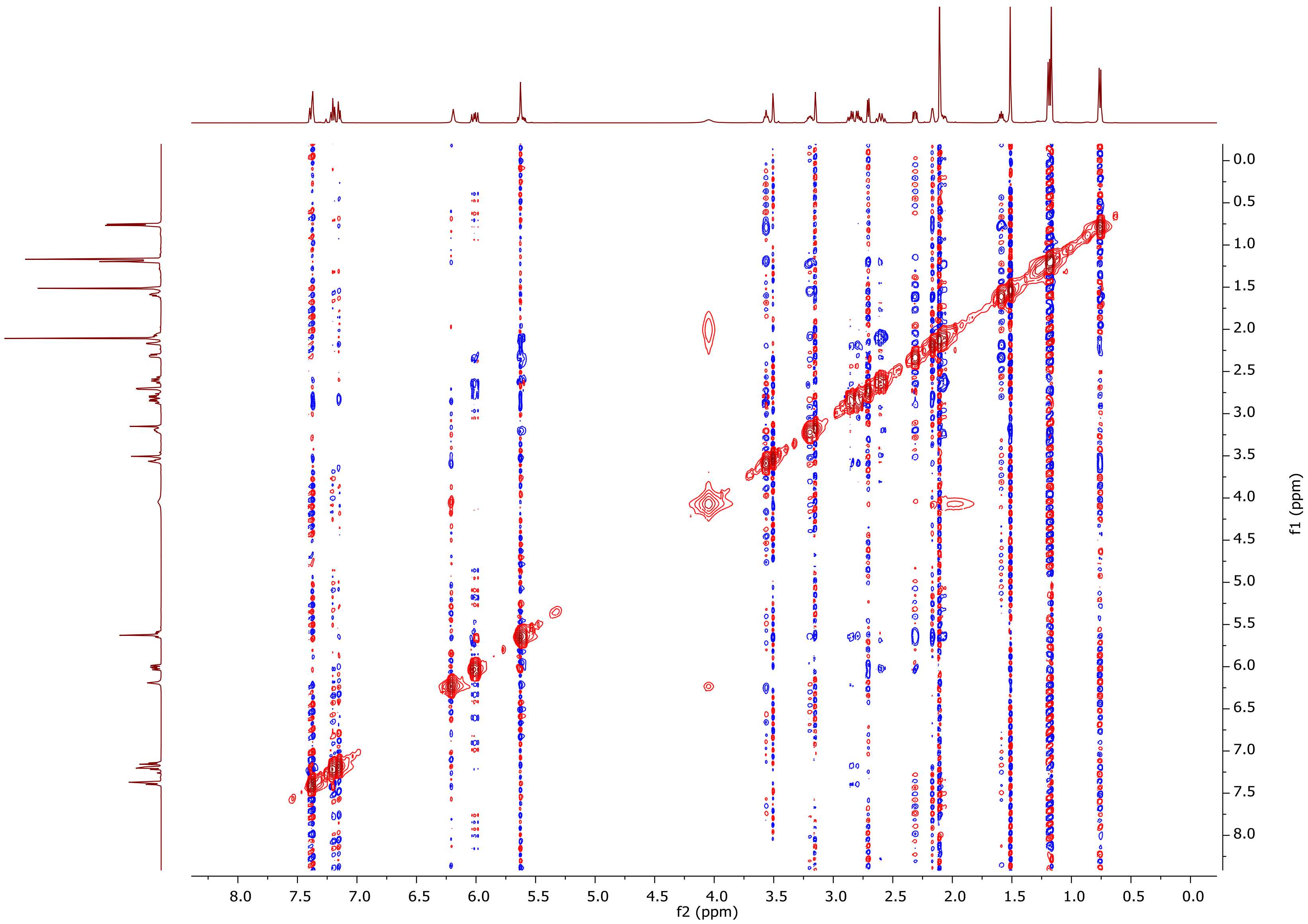

Figure SD126. ^{1}H-^{1}H NOESY NMR spectrum of *m*-bromo-19,20-epocycytochalasin Q (**18**) (500/500 MHz, $CDCl_3$)

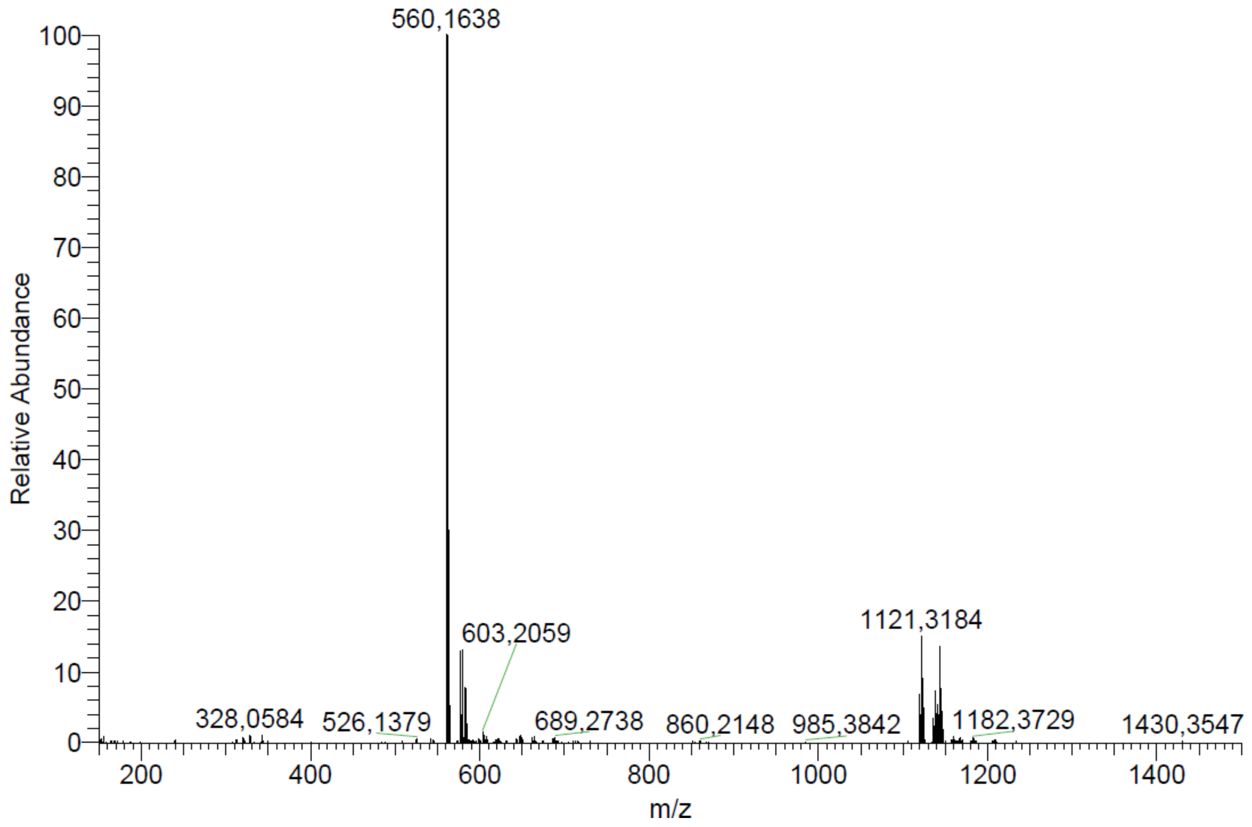

Figure SD127. ESI-HRMS spectrum of *m*-bromo-deacetyl-19,20-epocycytochalasin Q (**19**)

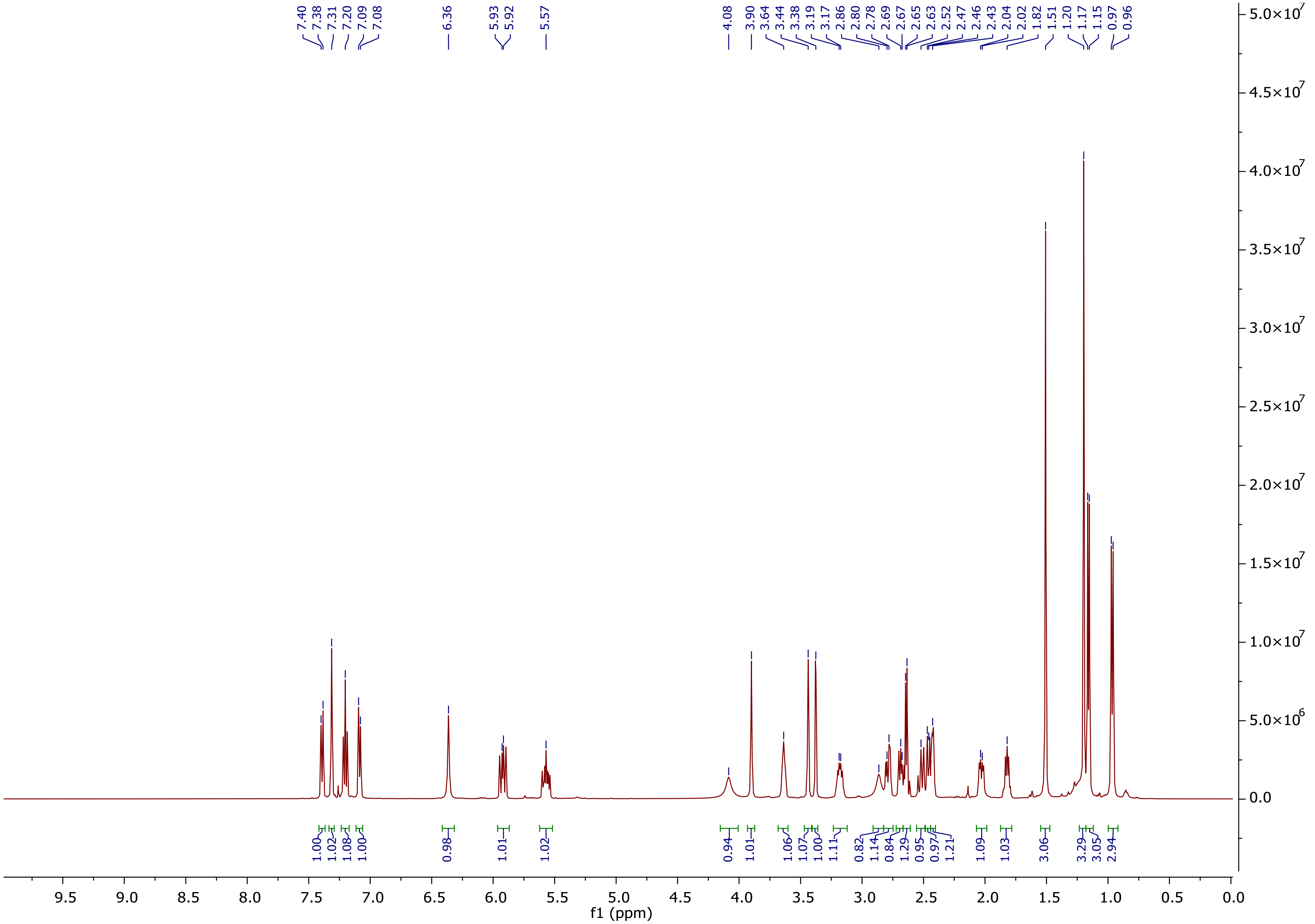

Figure SD128. ¹H NMR spectrum of *m*-bromo-deacetyl-19,20-epocycytochalasin Q (**19**) (500 MHz, $CDCl_3$)

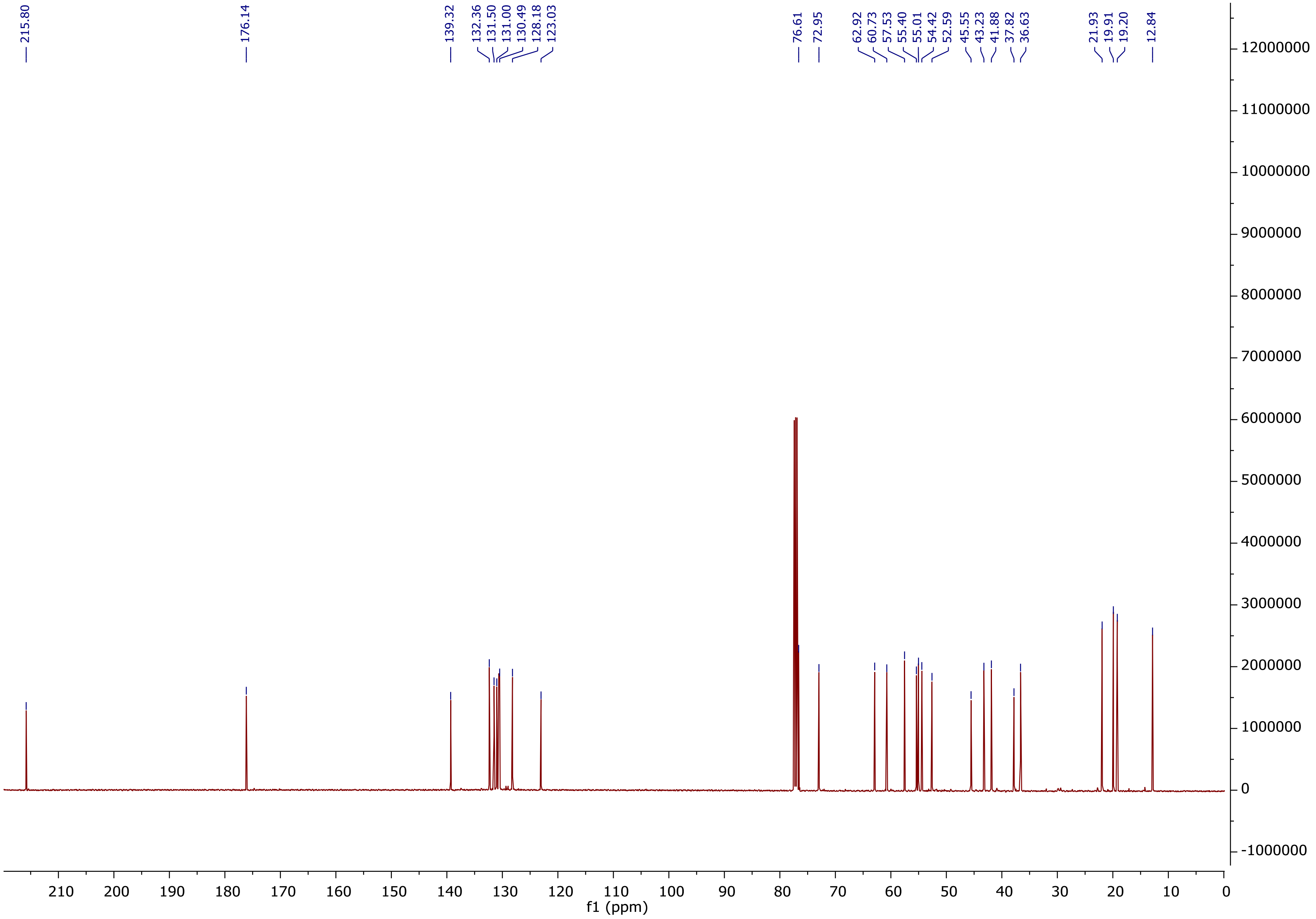

Figure SD129. ^{13}C NMR spectrum of *m*-bromo-deacetyl-19,20-epocycytochalasin Q (**19**) (125 MHz, $CDCl_3$)

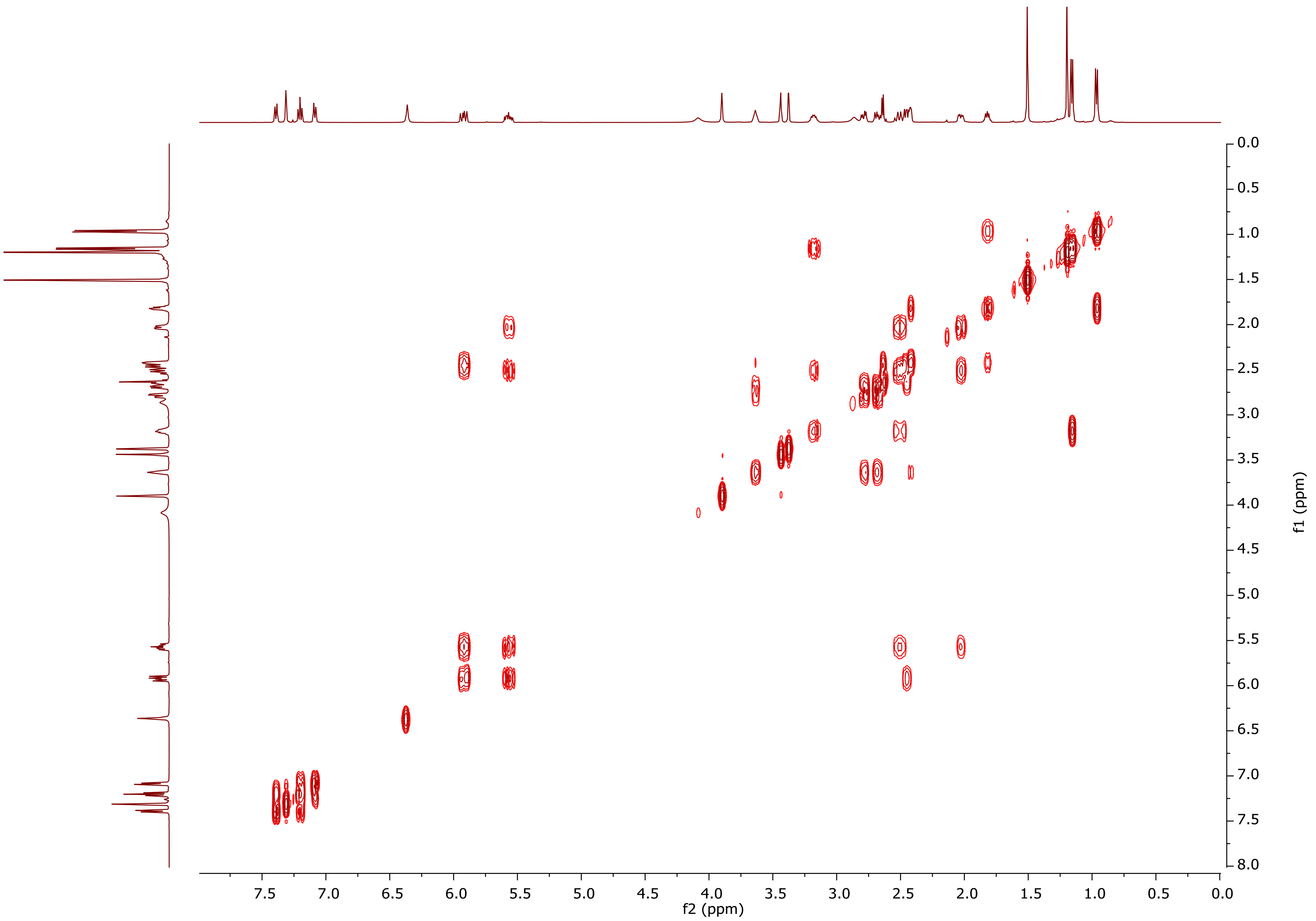

Figure SD130. ^{1}H-^{1}H COSY NMR spectrum of *m*-bromo-deacetyl-19,20-epocycytochalasin Q (**19**) (500/500 MHz, $CDCl_3$)

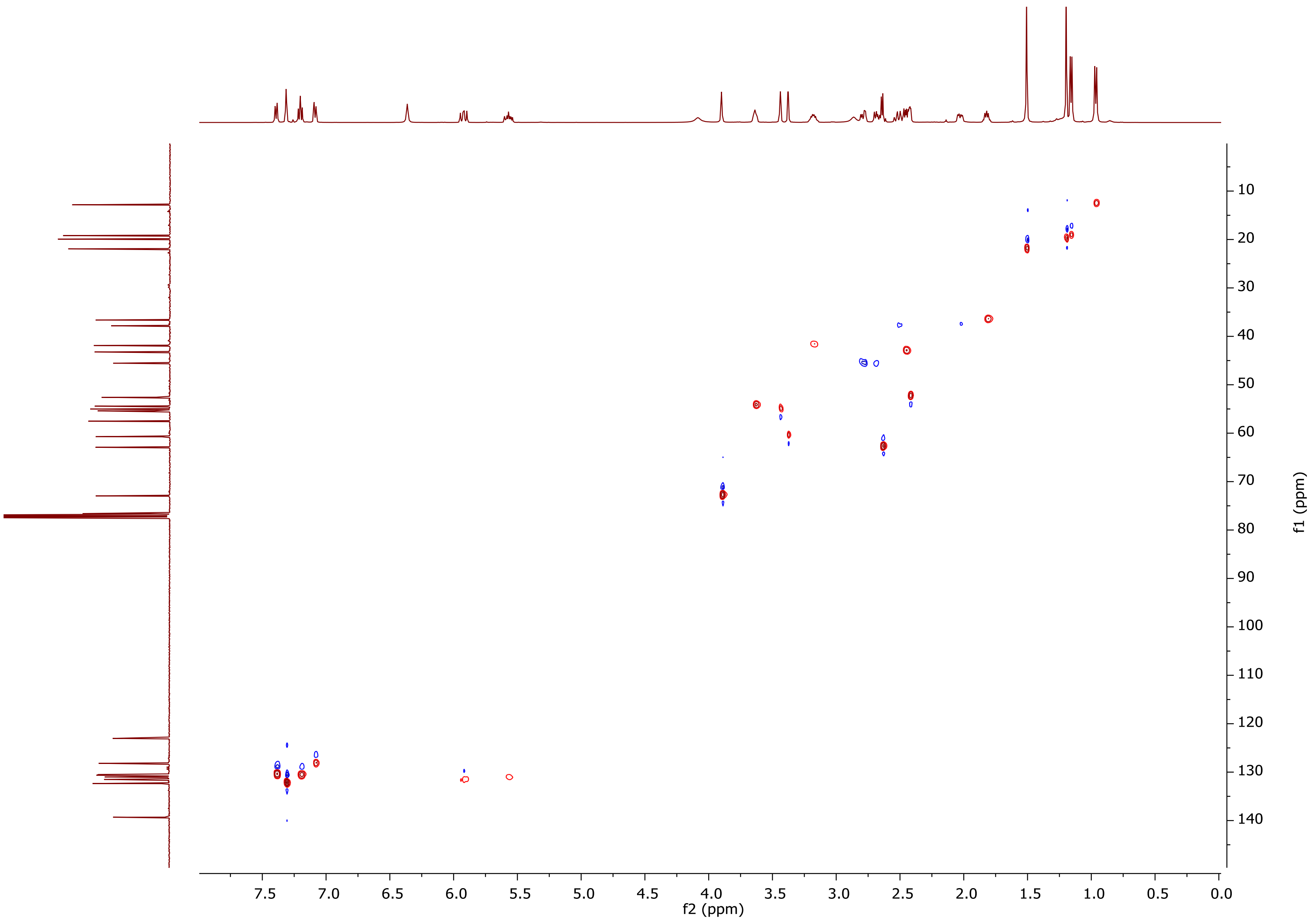

Figure SD131. ^1H-^{13}C HSQC NMR spectrum of *m*-bromo-deacetyl-19,20-epocycytochalasin Q (**19**) (500/125 MHz, $CDCl_3$)

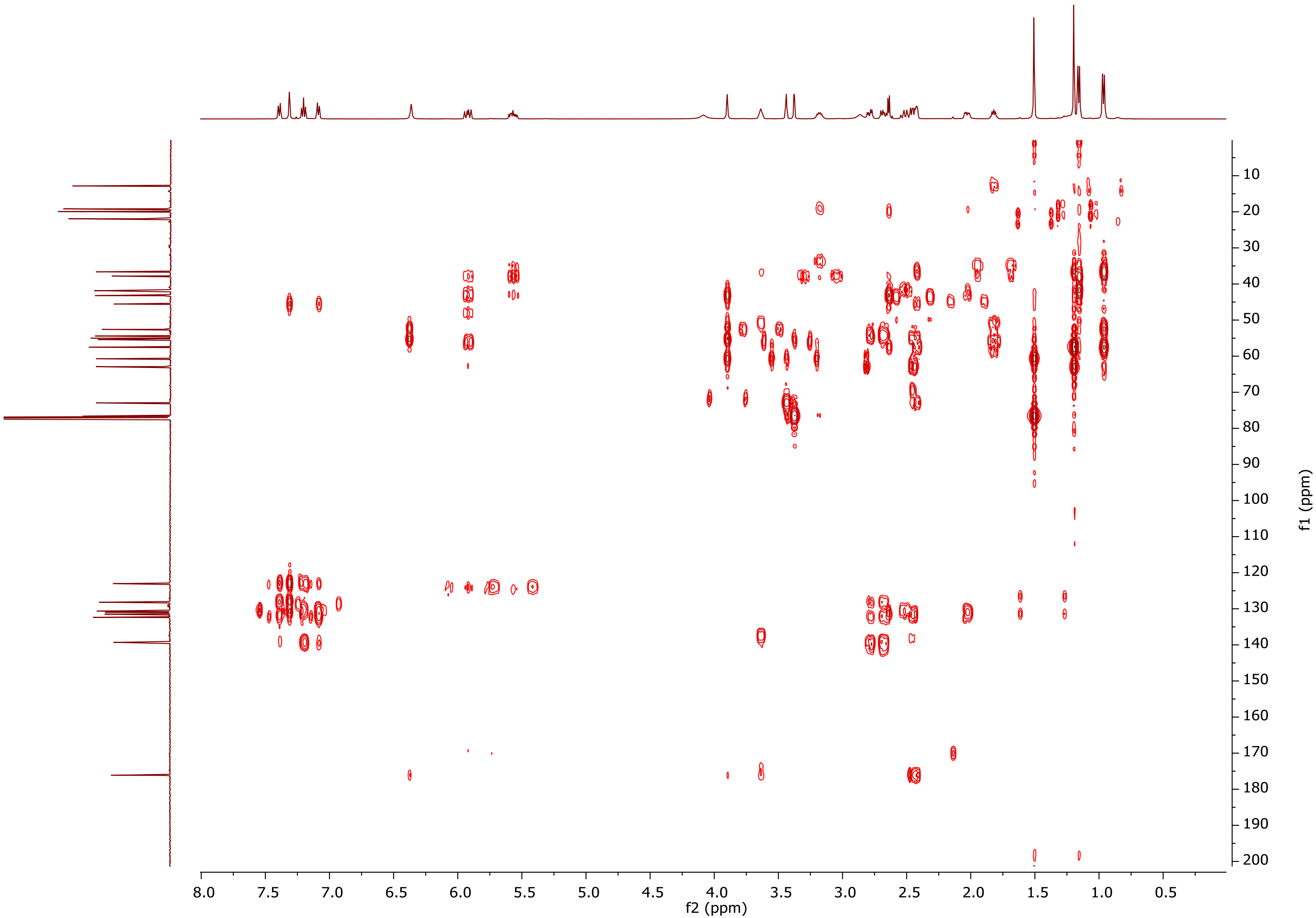

Figure SD132. ^{1}H-^{13}C HMBC NMR spectrum of *m*-bromo-deacetyl-19,20-epocycytochalasin Q (**19**) (500/125 MHz, $CDCl_3$)

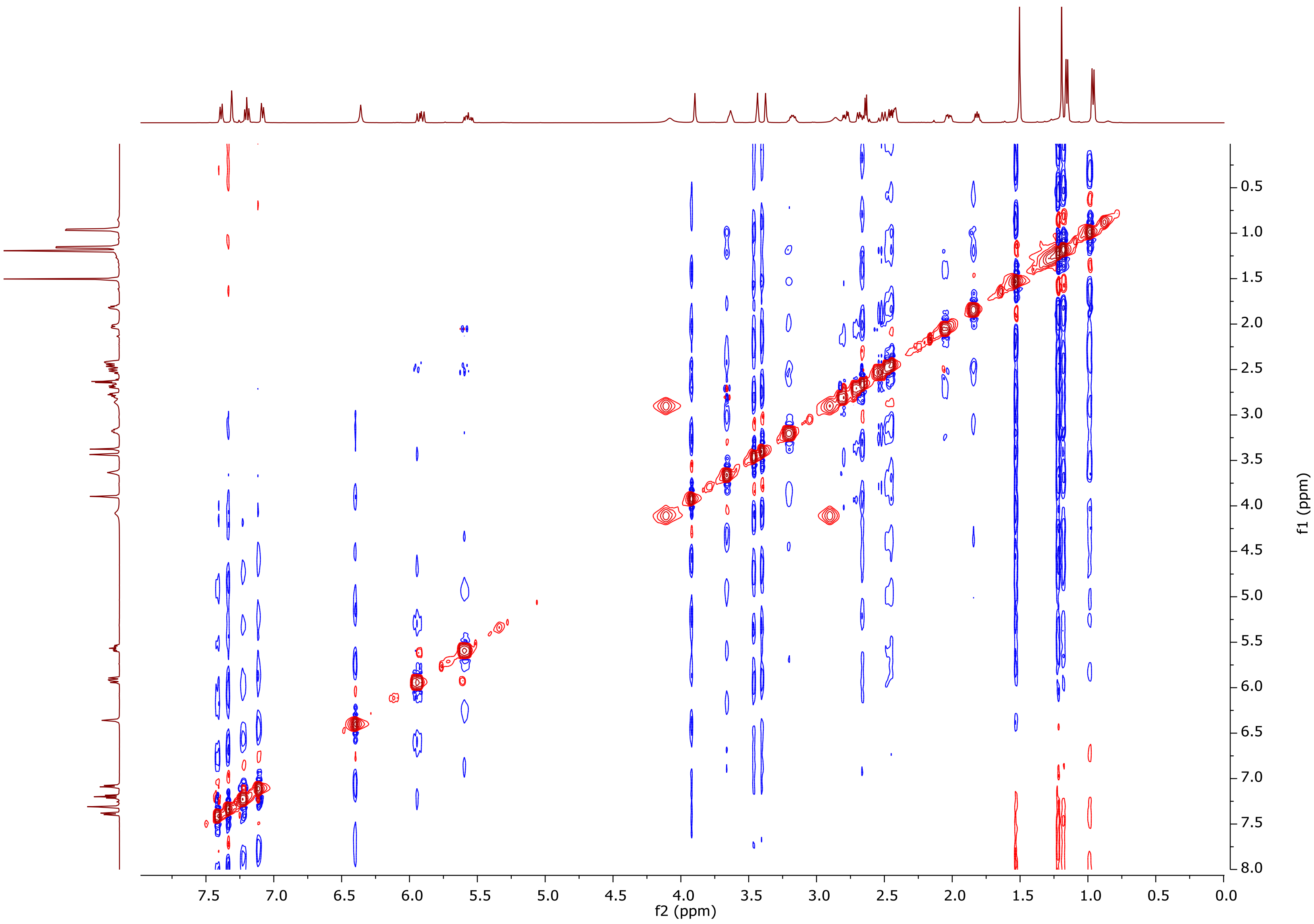

Figure SD133. ^{1}H-^{1}H NOESY NMR spectrum of *m*-bromo-deacetyl-19,20-epocycytochalasin Q (**19**) (500/500 MHz, $CDCl_3$)

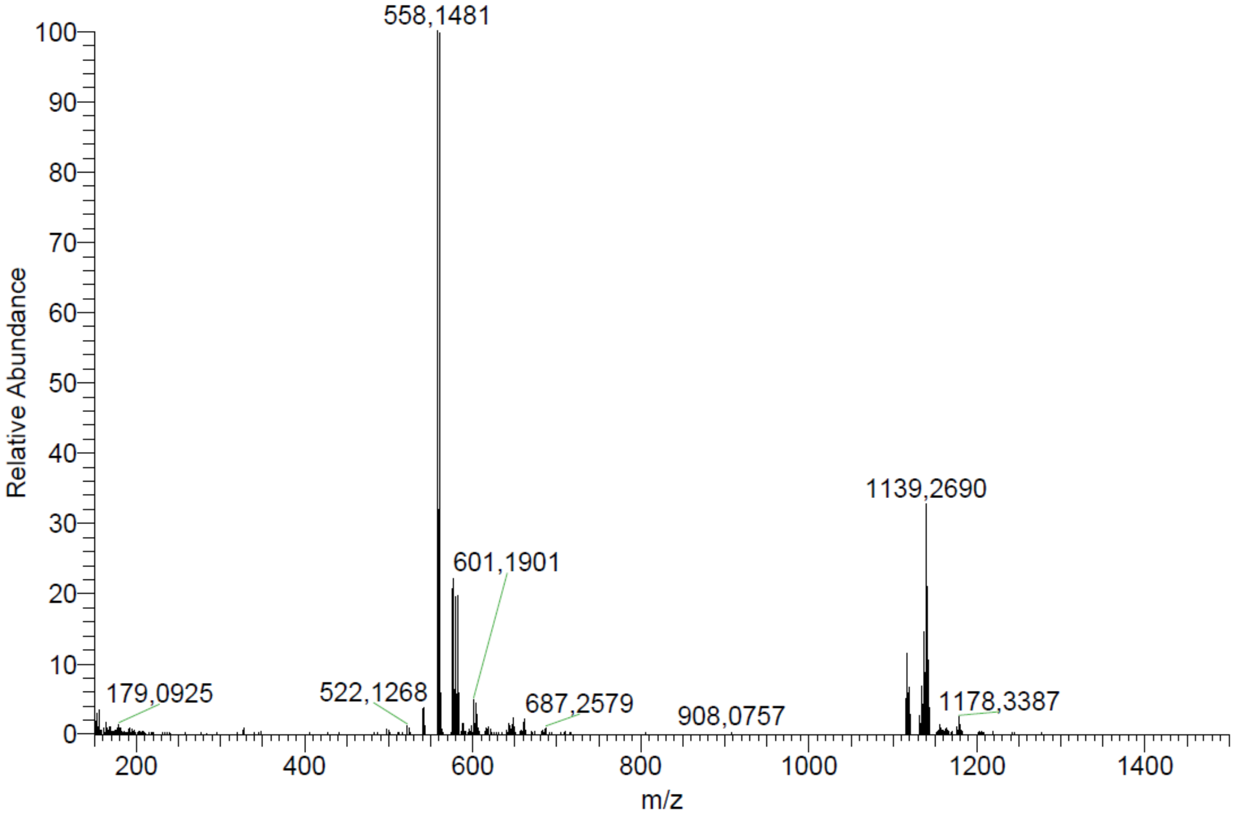

Figure SD134. ESI-HRMS spectrum of *o*-bromo-19,20-epoxy-21-oxocytochalasin Q (**20**)

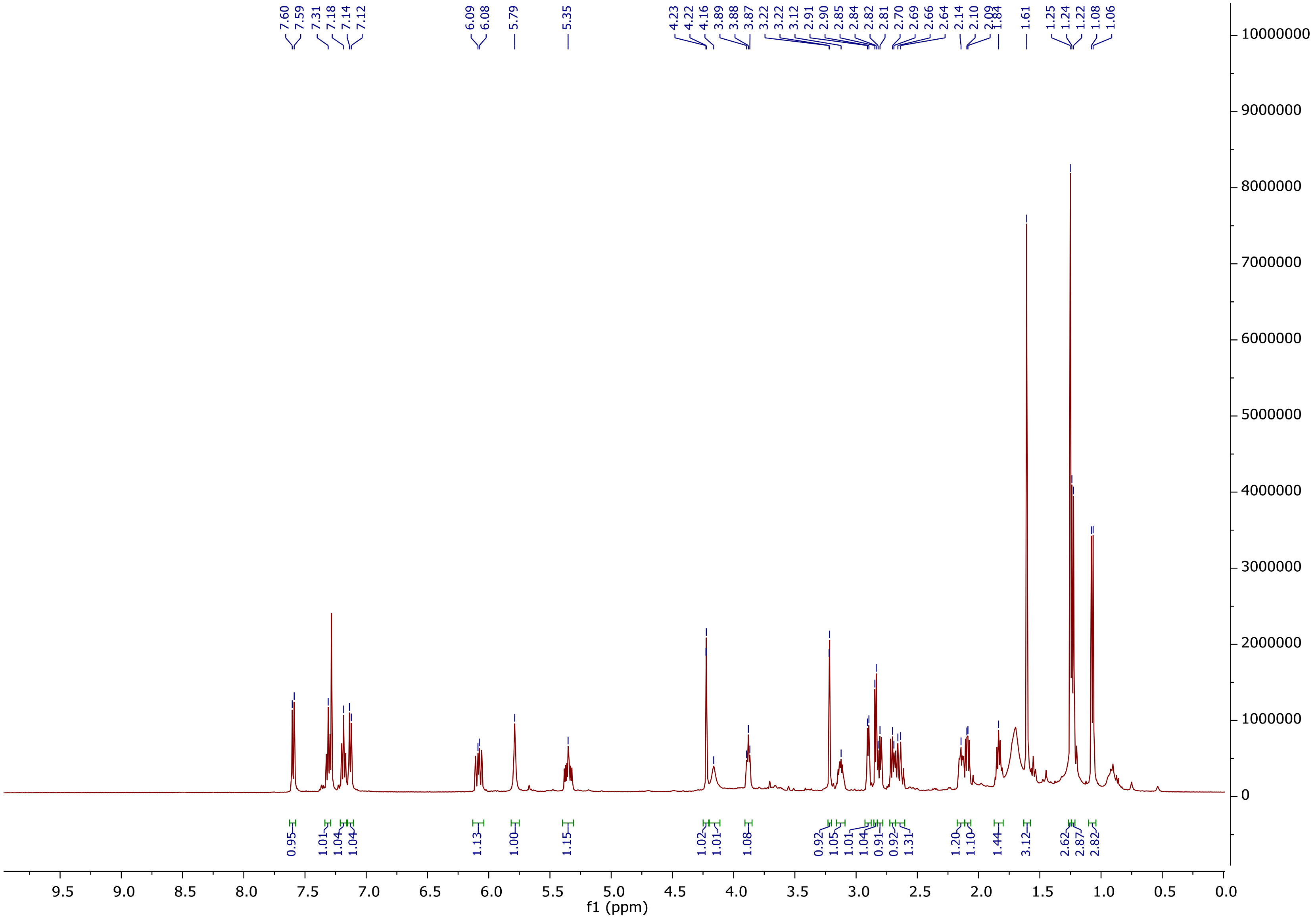

Figure SD135. ^{1}H NMR spectrum of *o*-bromo-19,20-epoxy-21-oxocytochalasin Q (**20**) (500 MHz, $CDCl_3$)

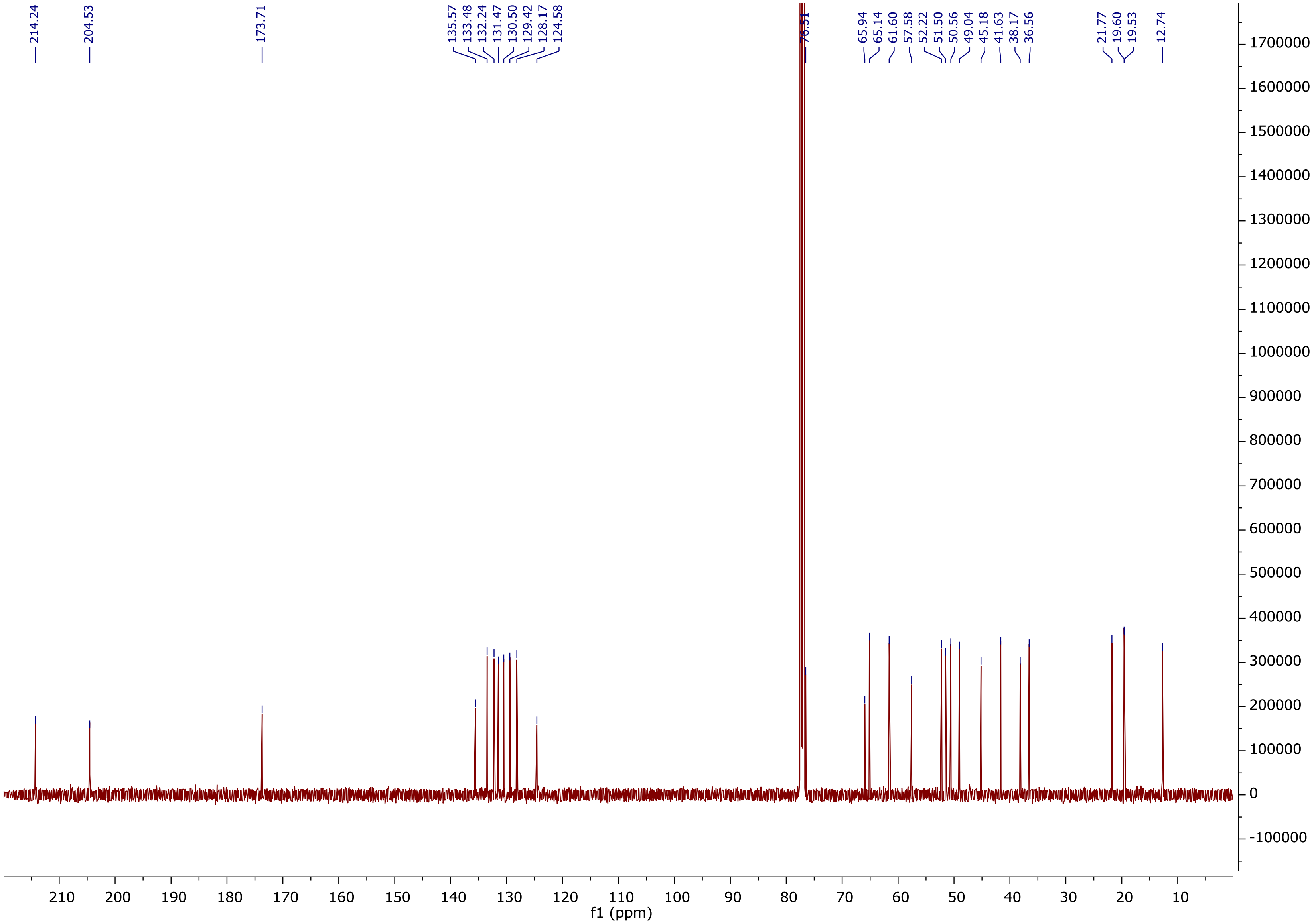

Figure SD136. ^{13}C NMR spectrum of *o*-bromo-19,20-epoxy-21-oxocytochalasin Q (**20**) (125 MHz, $CDCl_3$)

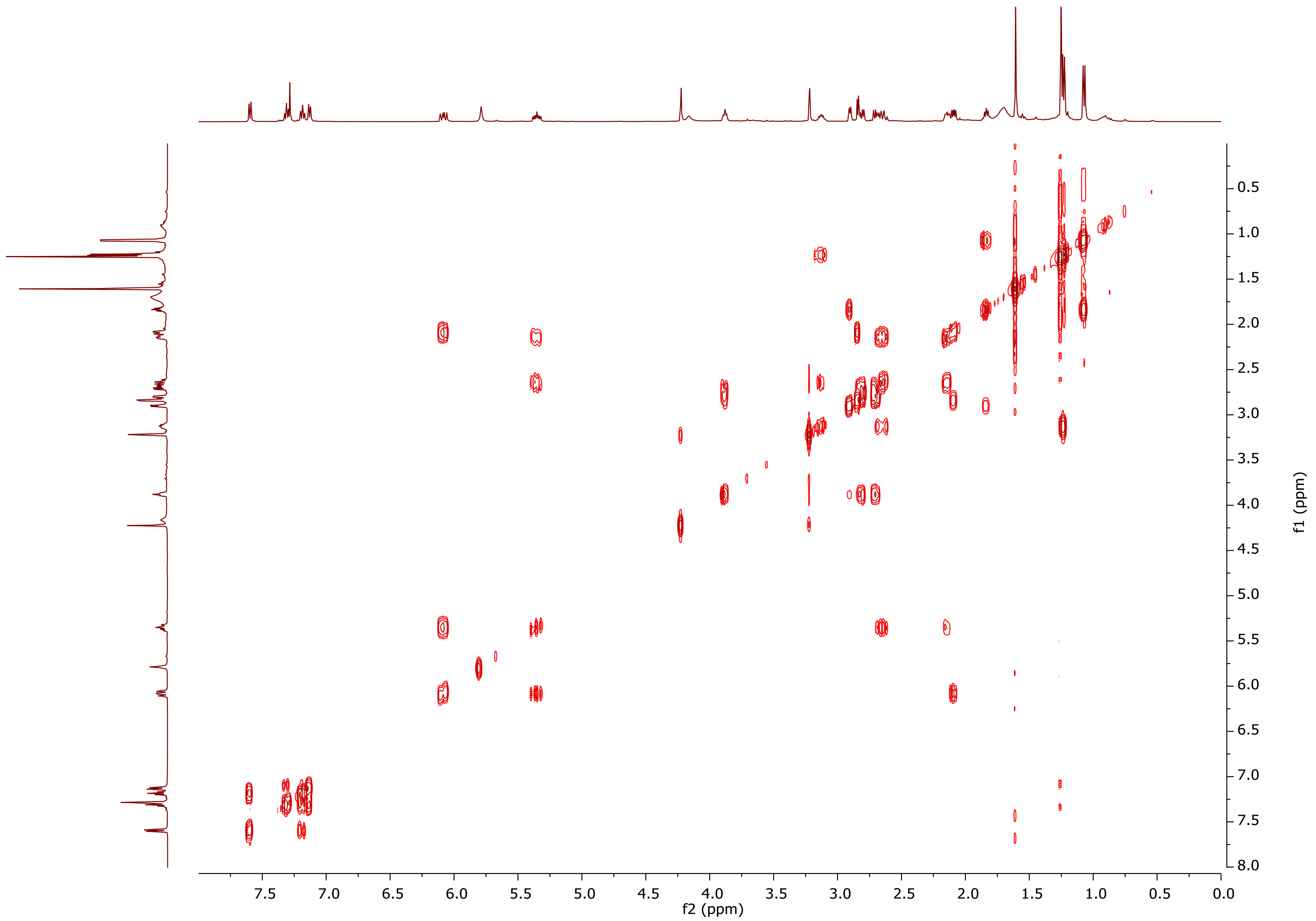

Figure SD137. ^{1}H-^{1}H COSY NMR spectrum of *o*-bromo-19,20-epoxy-21-oxocytochalasin Q (**20**) (500/500 MHz, $CDCl_3$)

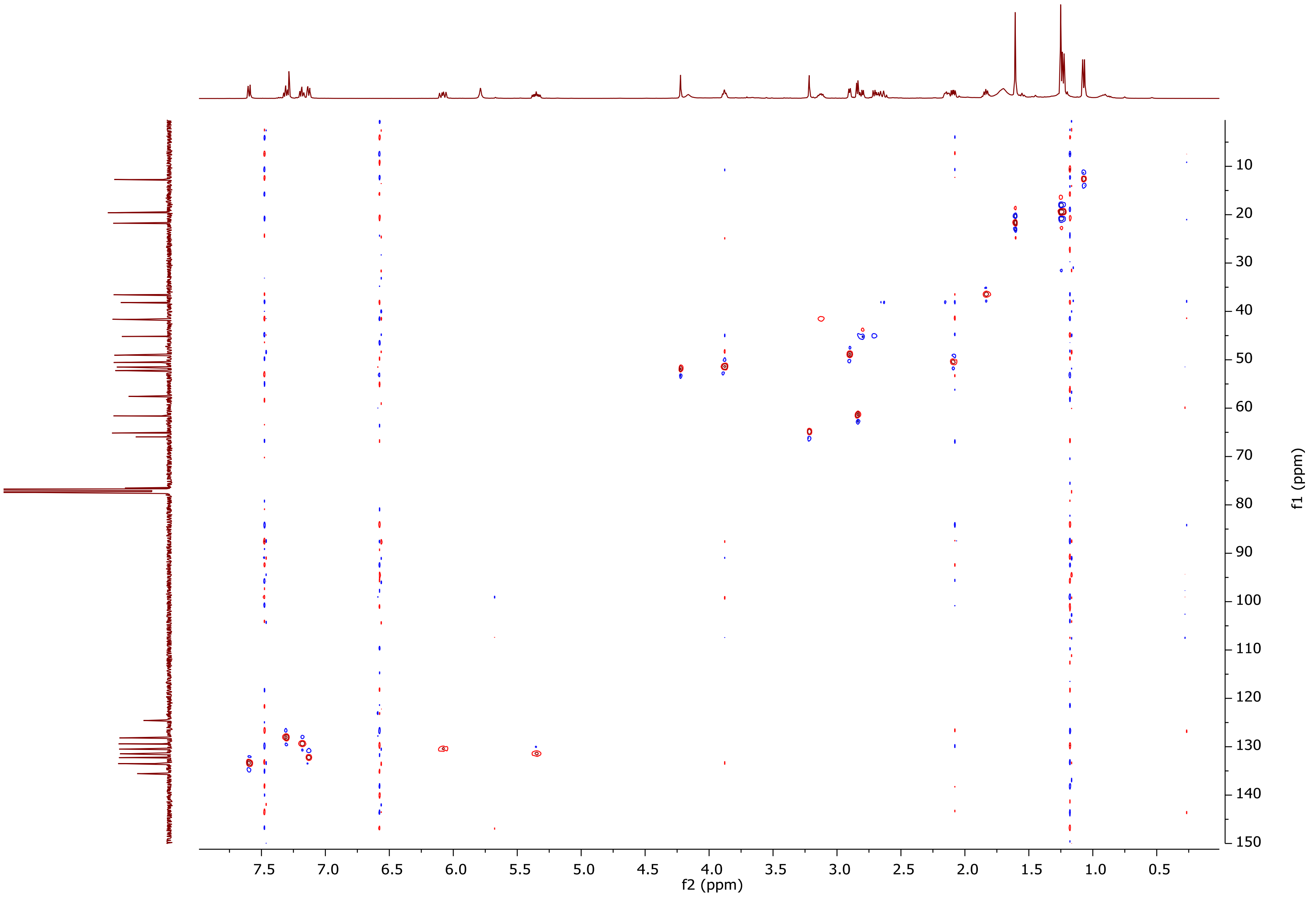

Figure SD138. ^{1}H-^{13}C HSQC NMR spectrum of *o*-bromo-19,20-epoxy-21-oxocytochalasin Q (**20**) (500/125 MHz, $CDCl_3$)

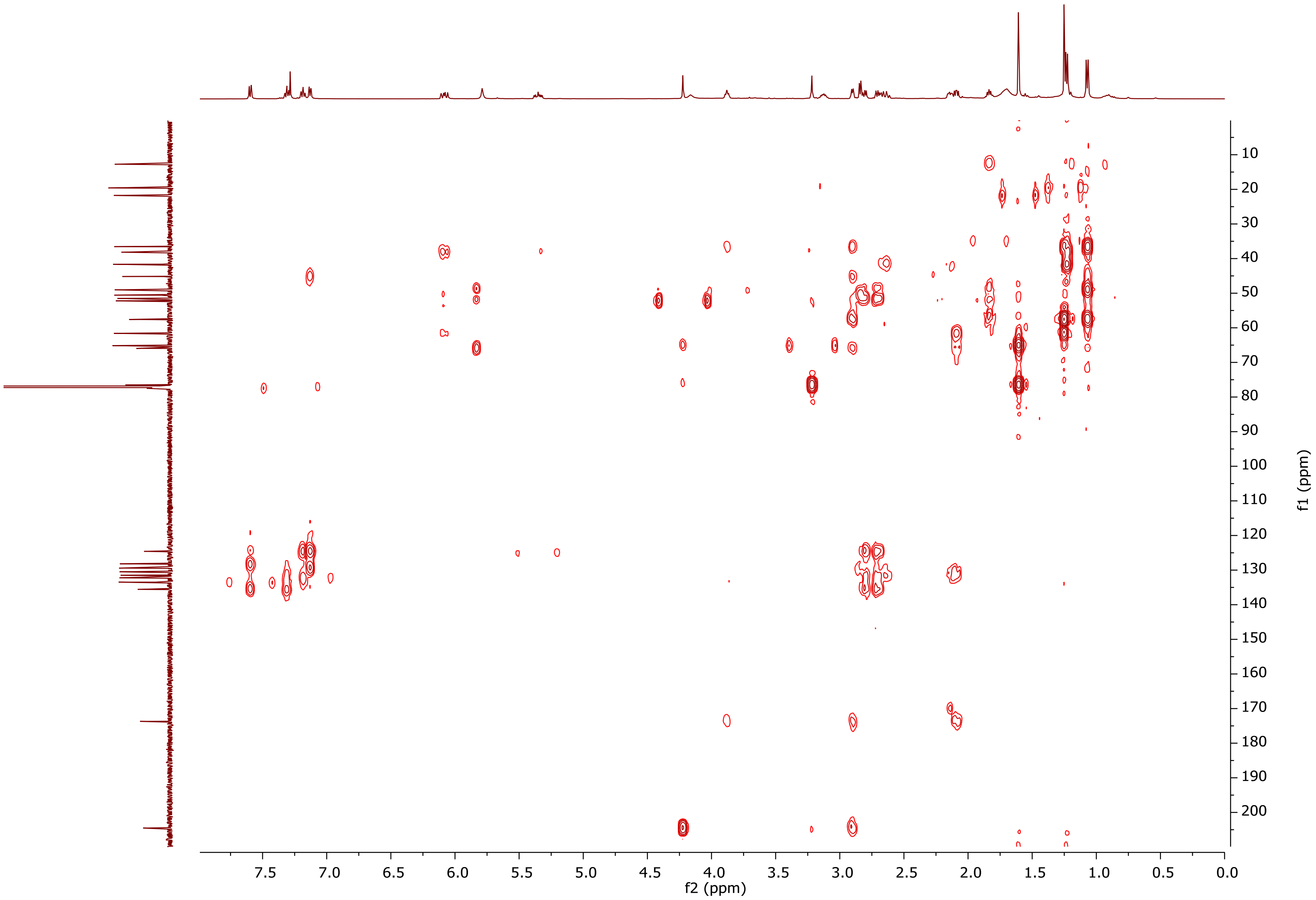

Figure SD139. ^1H-^{13}C HMBC NMR spectrum of *o*-bromo-19,20-epoxy-21-oxocytochalasin Q (**20**) (500/125 MHz, $CDCl_3$)

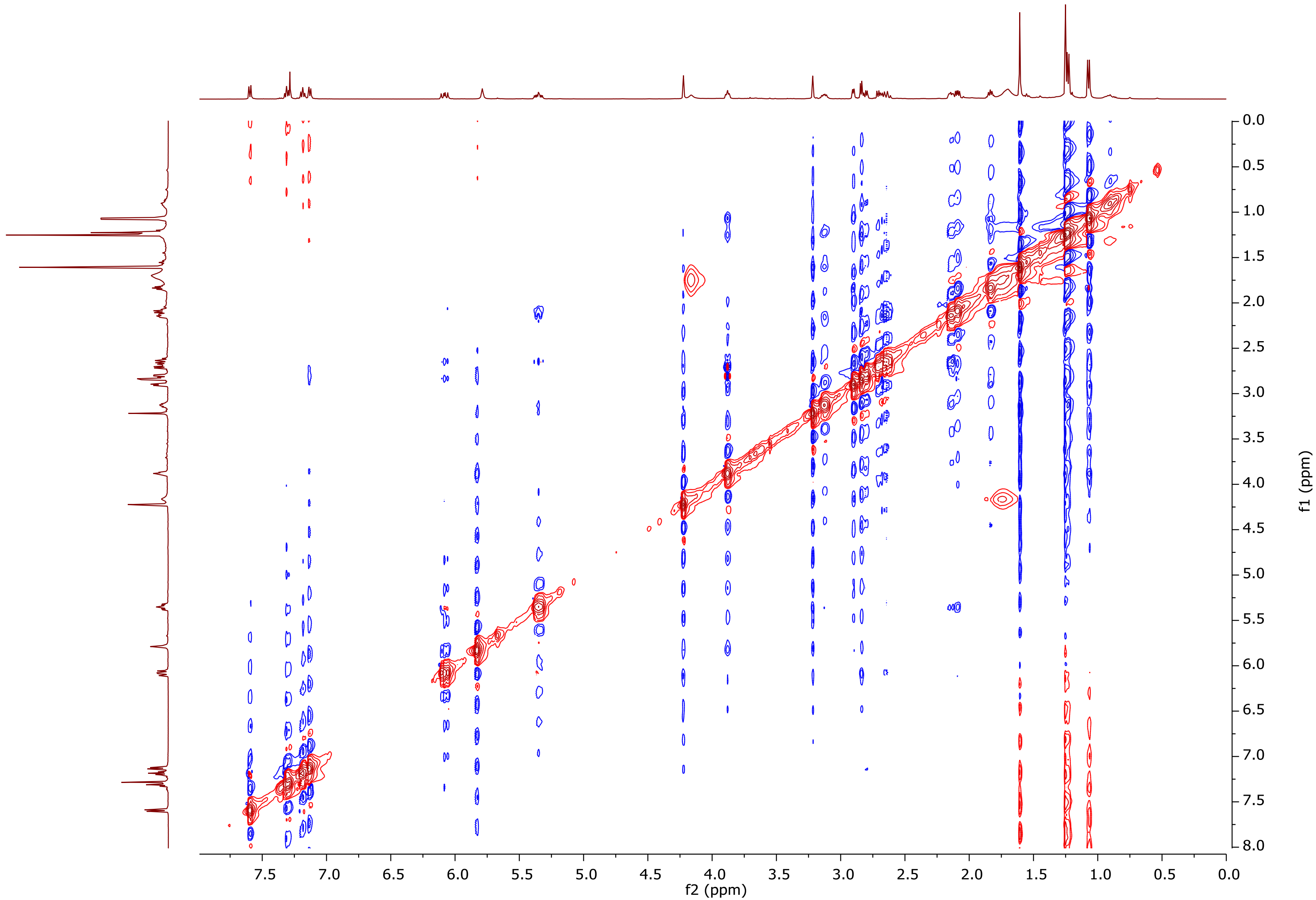

Figure SD140. ^{1}H-^{1}H NOESY NMR spectrum of *o*-bromo-19,20-epoxy-21-oxocytochalasin Q (**20**) (500/500 MHz, $CDCl_3$)

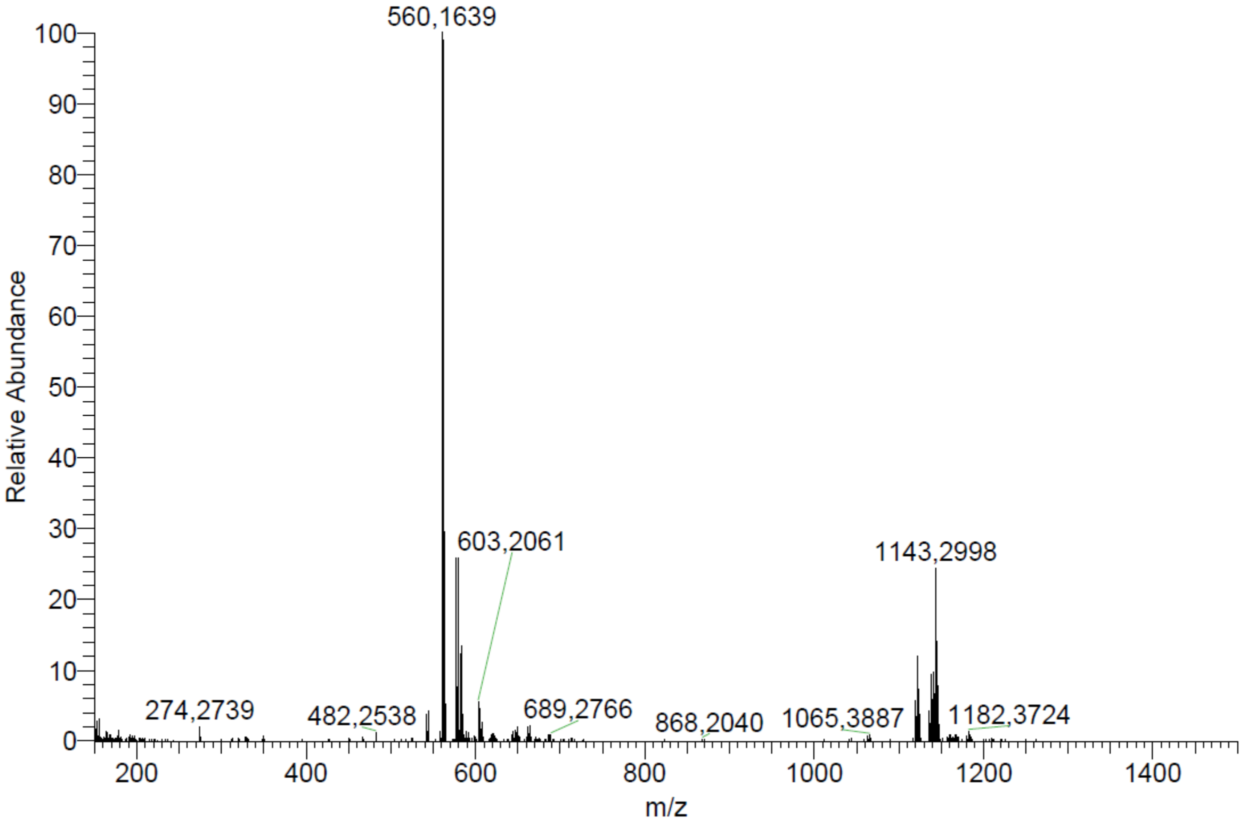

Figure SD141. ESI-HRMS spectrum of *o*-bromo-19-hydroxy-21-oxocytochalasin Q (**21**)

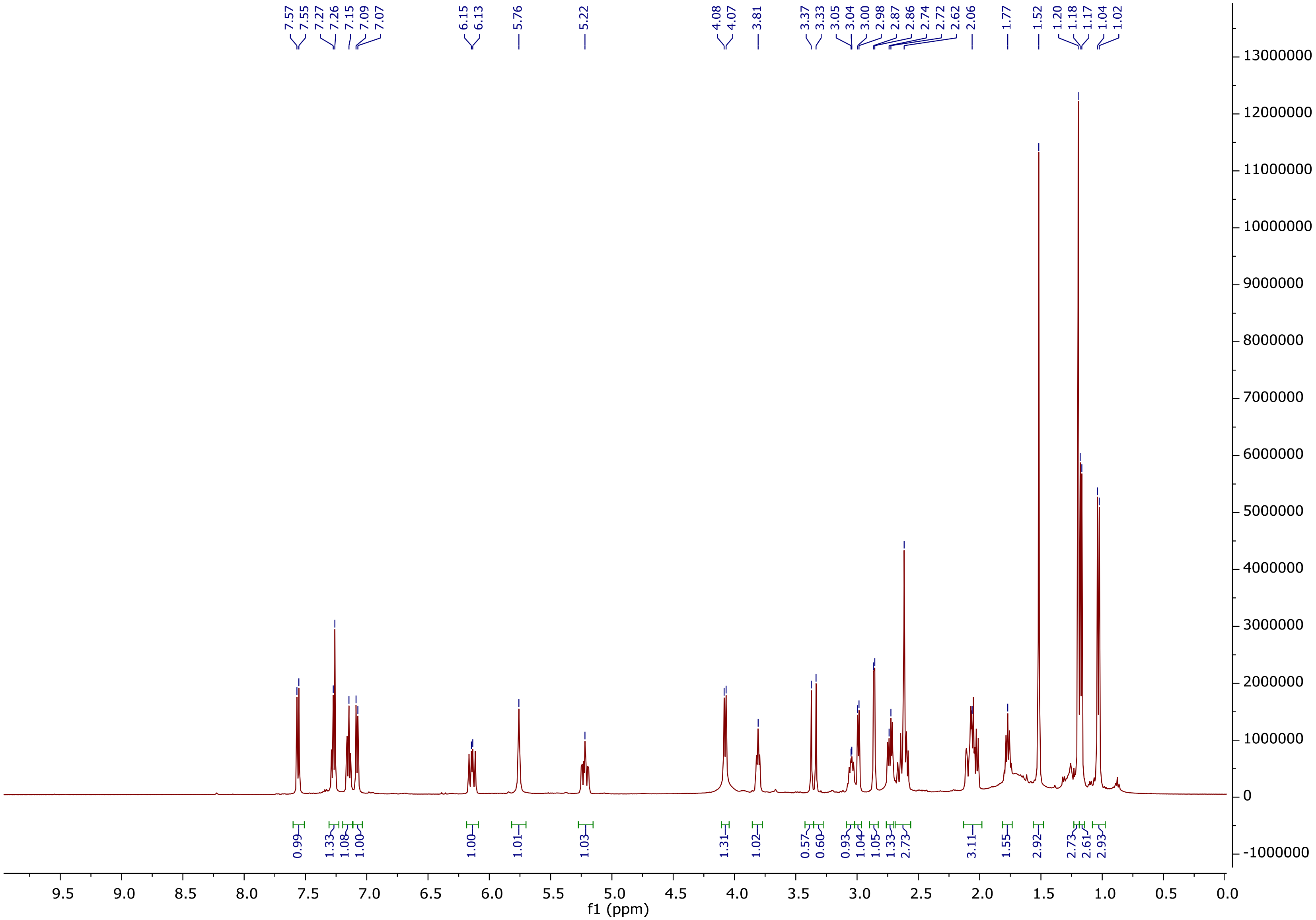

Figure SD142. ^{1}H NMR spectrum of *o*-bromo-19-hydroxy-21-oxocytochalasin Q (**21**) (500 MHz, $CDCl_3$)

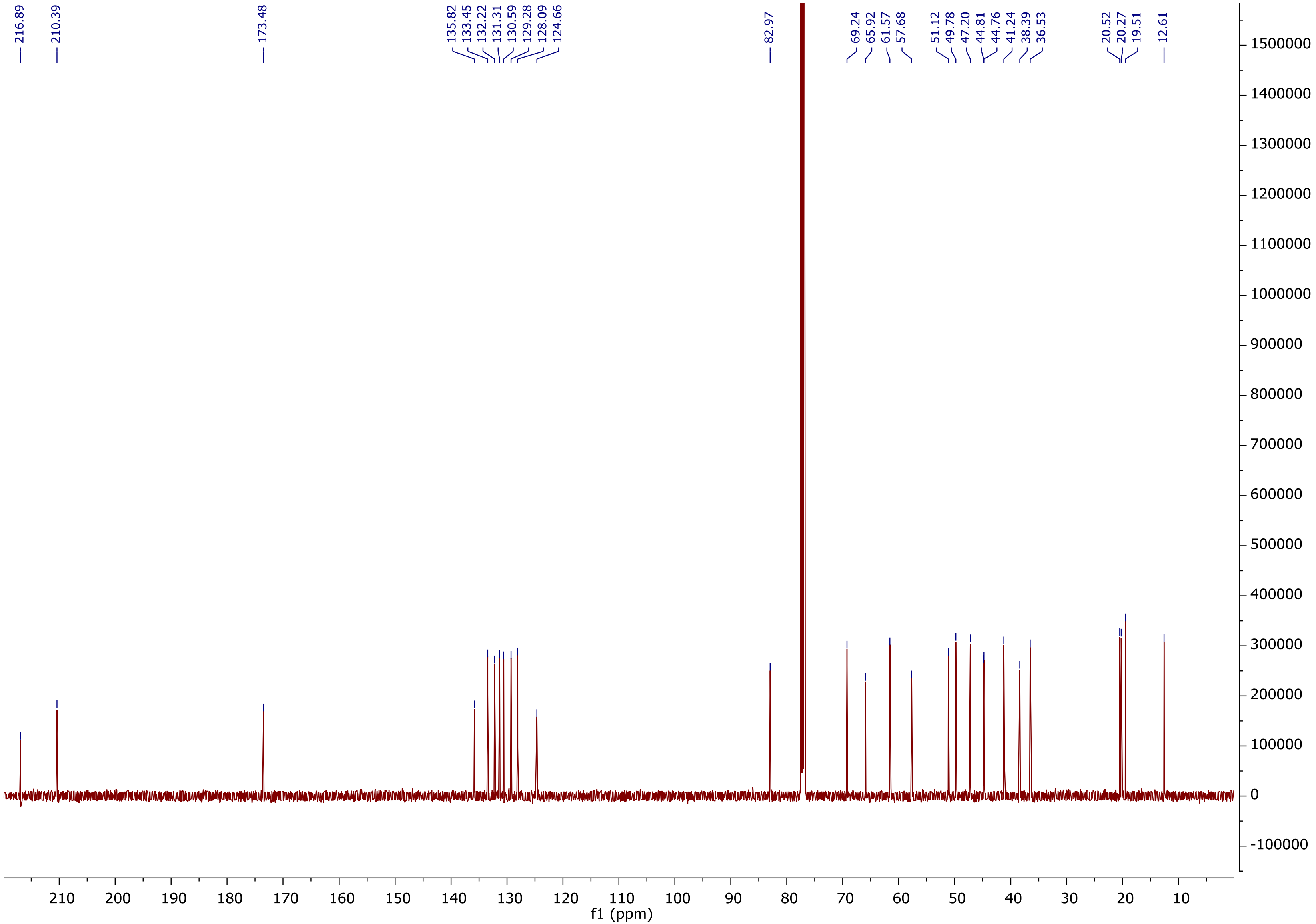

Figure SD143. ^{13}C NMR spectrum of *o*-bromo-19-hydroxy-21-oxocytochalasin Q (**21**) (125 MHz, $CDCl_3$)

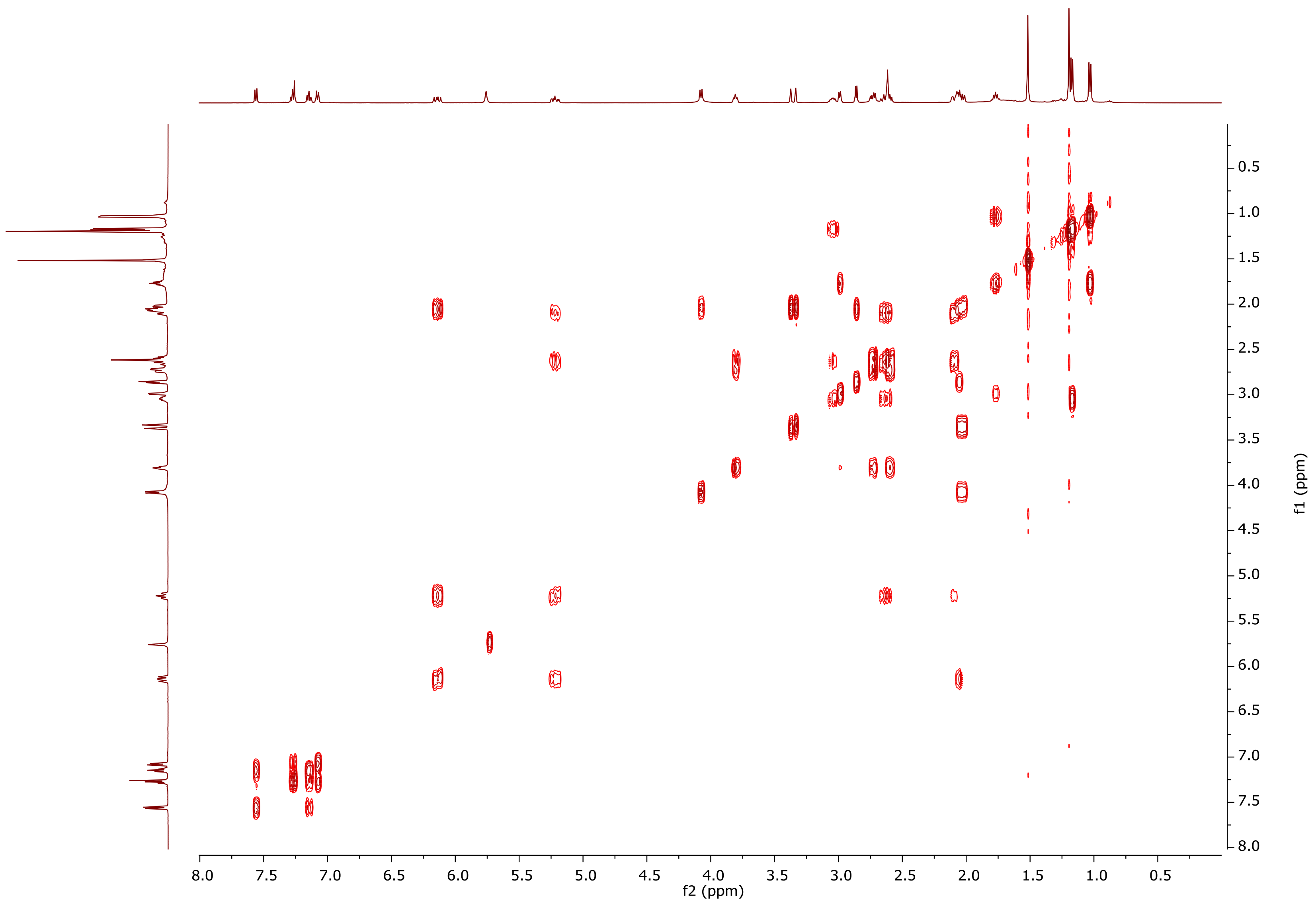

Figure SD144. ^{1}H-^{1}H COSY NMR spectrum of *o*-bromo-19-hydroxy-21-oxocytochalasin Q (**21**) (500/500 MHz, $CDCl_3$)

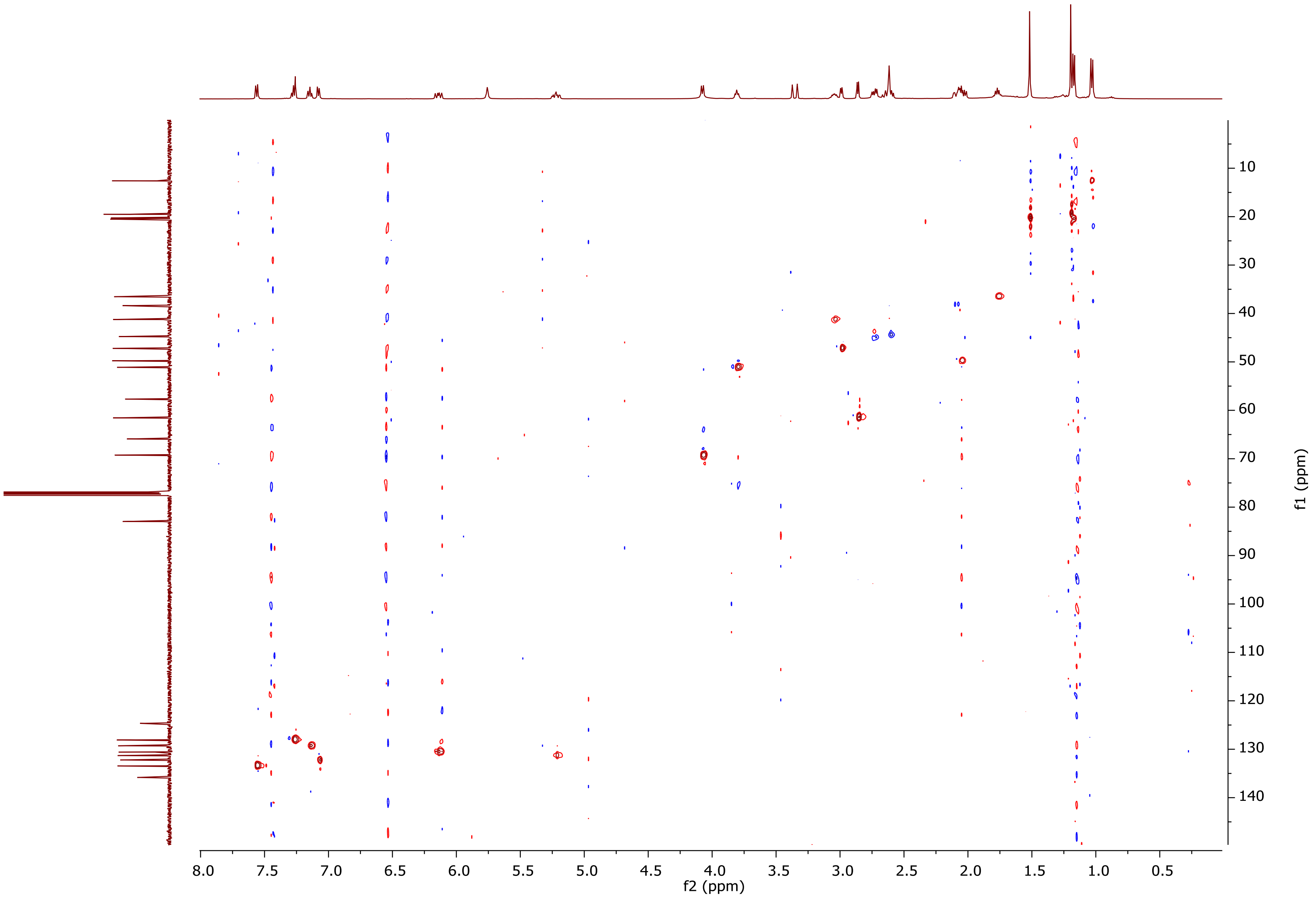

Figure SD145. ^{1}H-^{13}C HSQC NMR spectrum of *o*-bromo-19-hydroxy-21-oxocytochalasin Q (**21**) (500/125 MHz, $CDCl_3$)

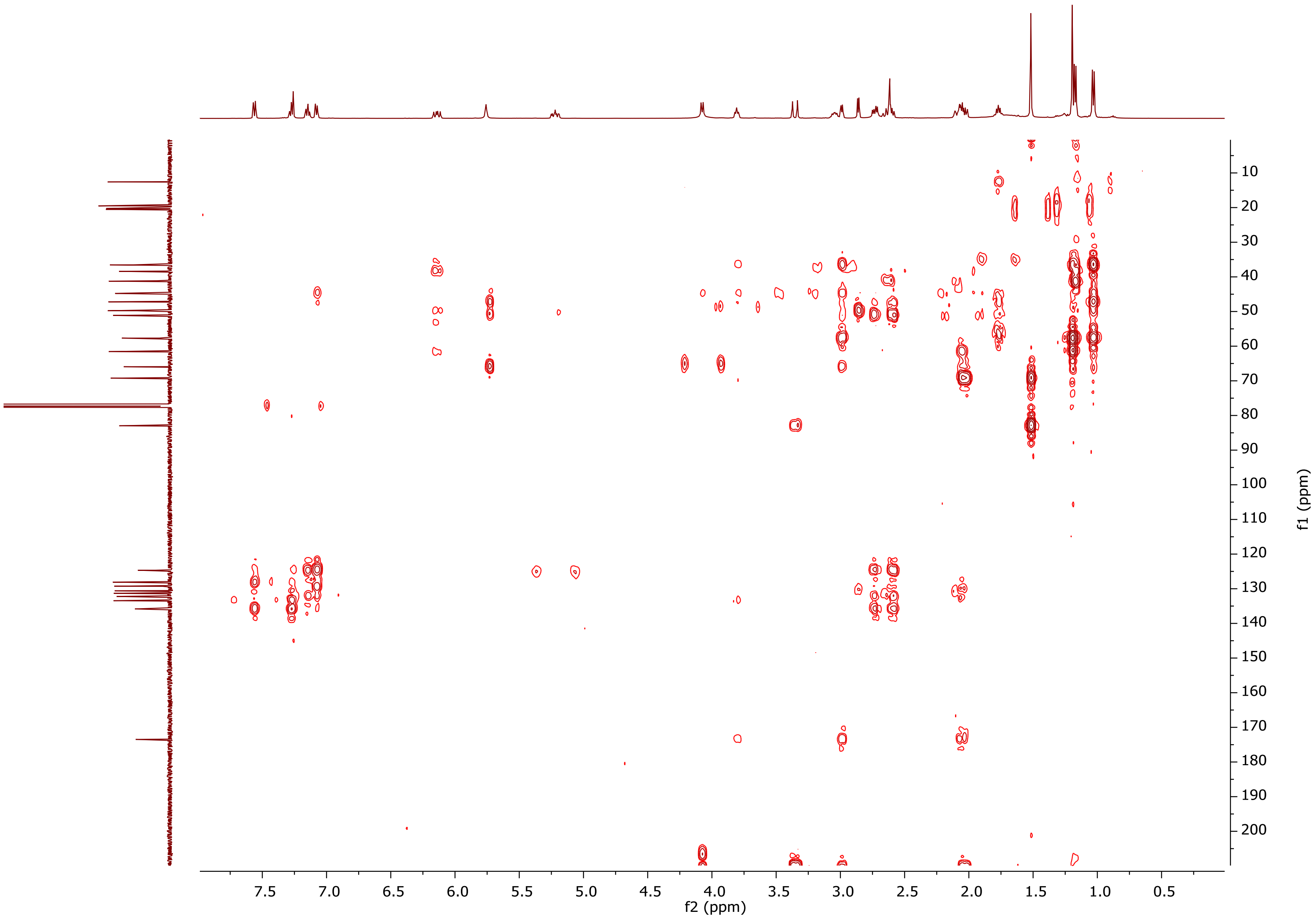

Figure SD146. ^{1}H-^{13}C HMBC NMR spectrum of *o*-bromo-19-hydroxy-21-oxocytochalasin Q (**21**) (500/125 MHz, $CDCl_3$)

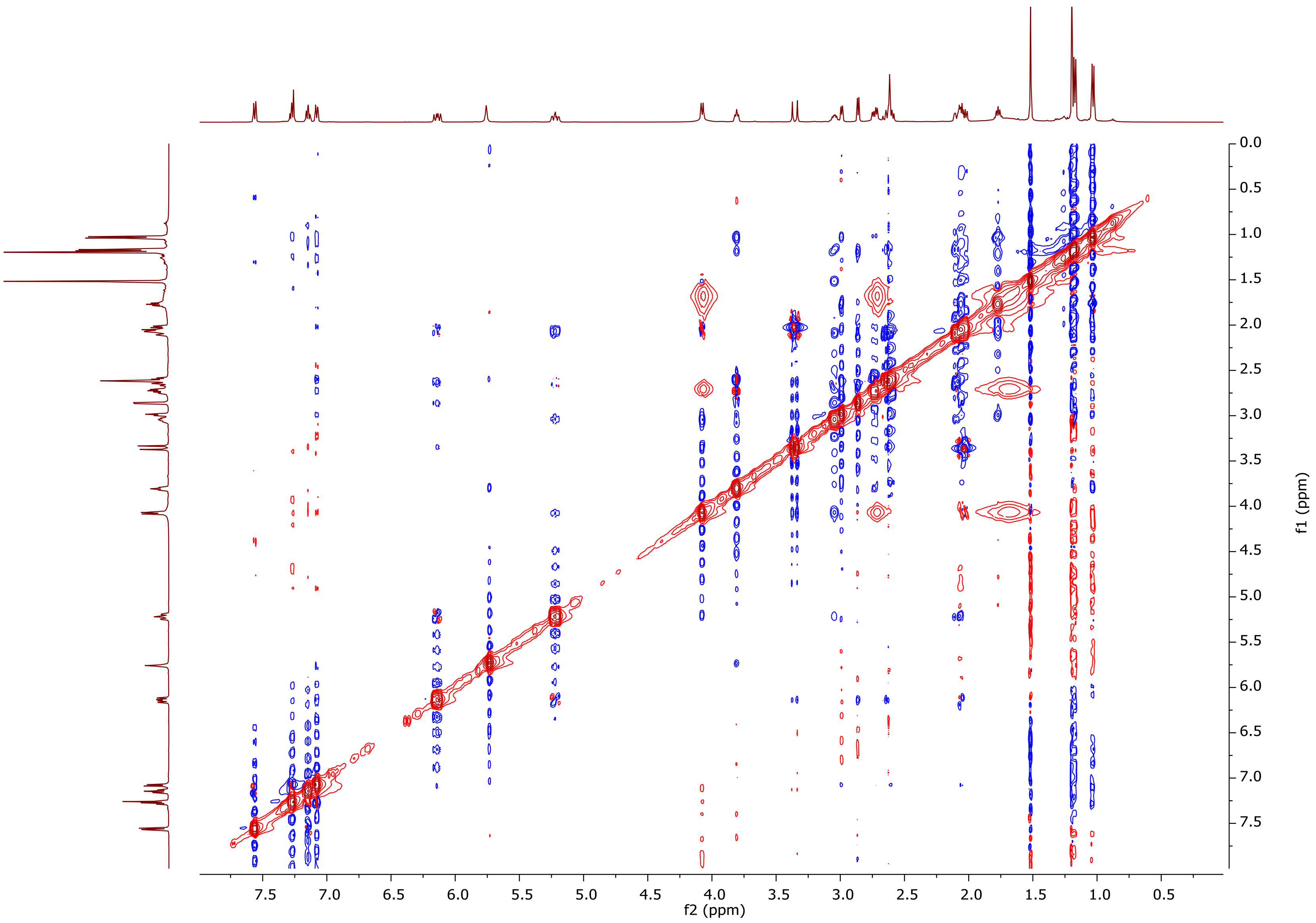

Figure SD147. ^{1}H-^{1}H NOESY NMR spectrum of *o*-bromo-19-hydroxy-21-oxocytochalasin Q (**21**) (500/500 MHz, $CDCl_3$)

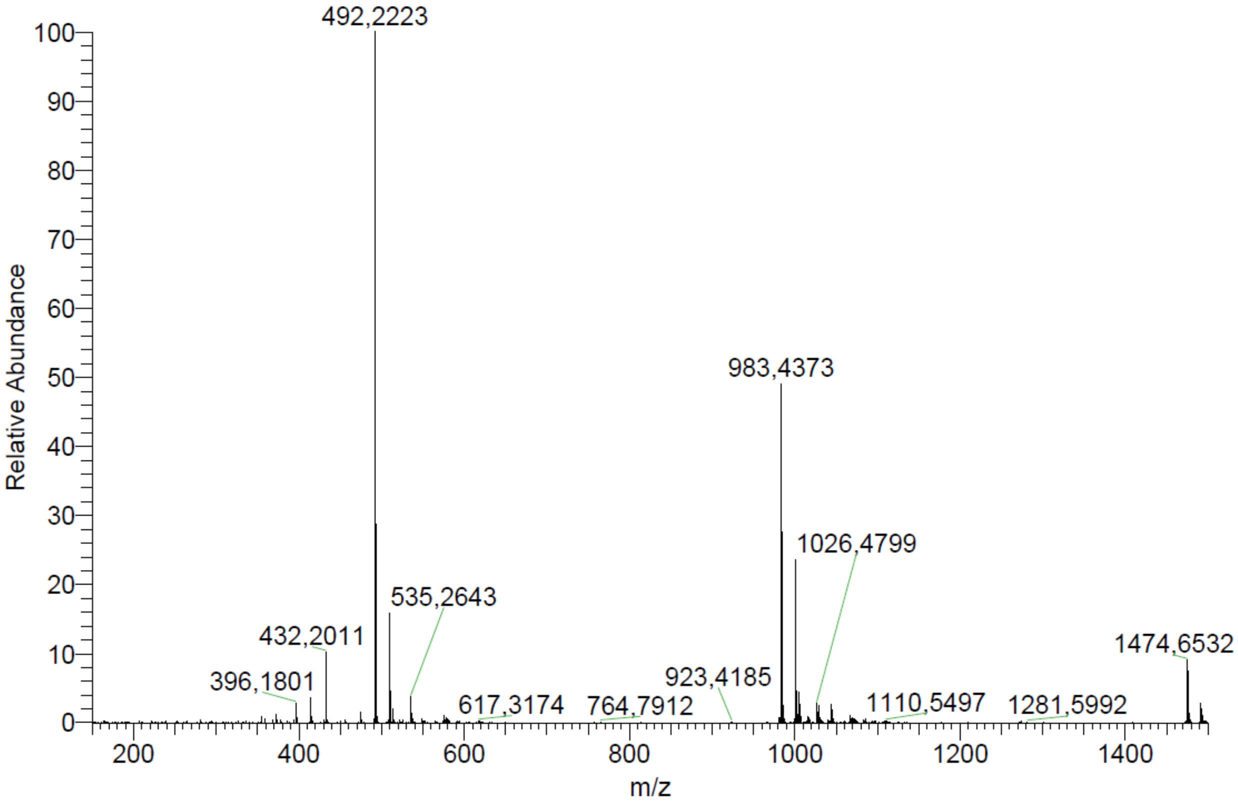

Figure SD148. ESI-HRMS spectrum of xylachalasin A (**22**)

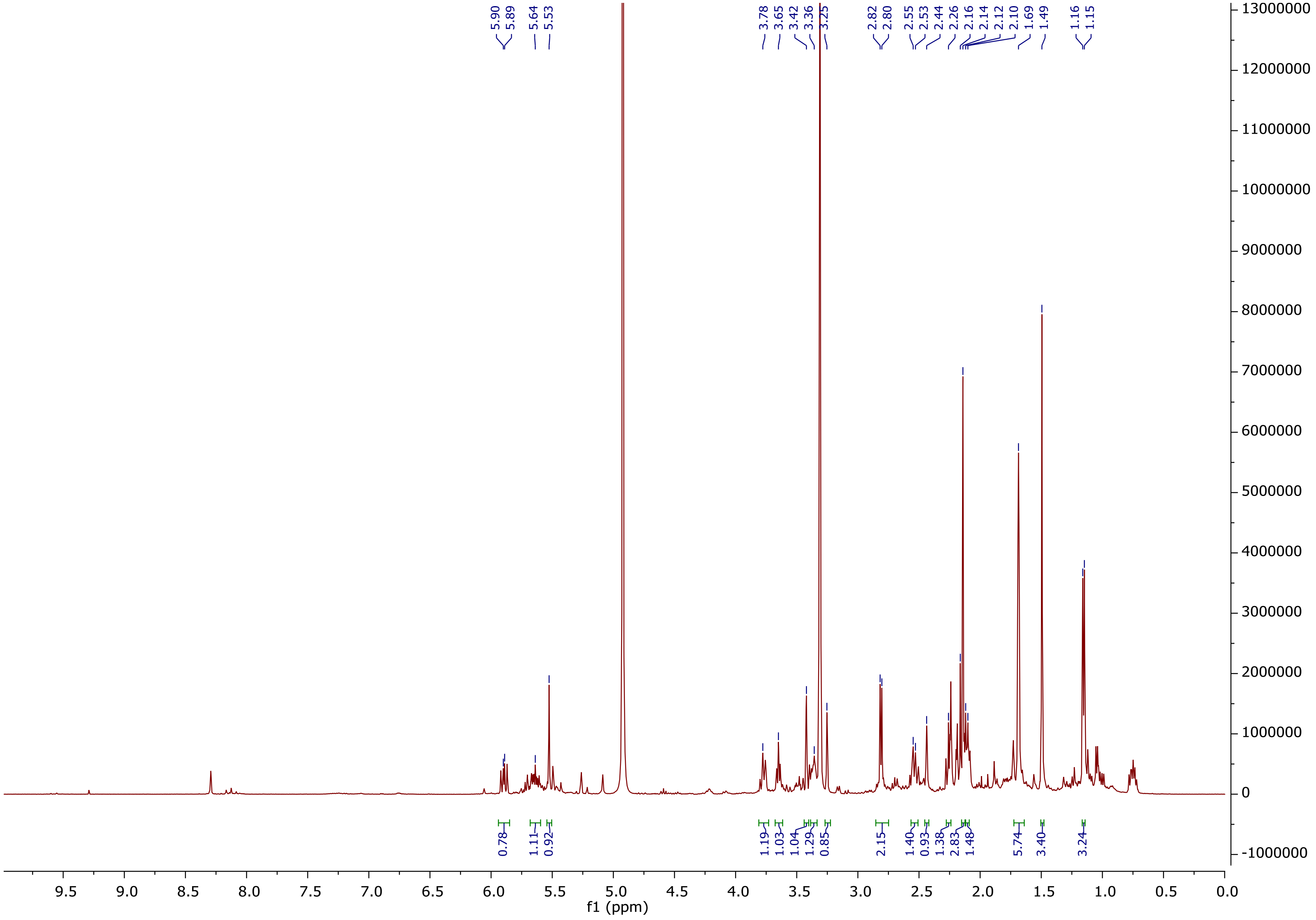

Figure SD149. ^{1}H NMR spectrum of xylachalasin A (**22**) (500 MHz, MeOH-d_4)

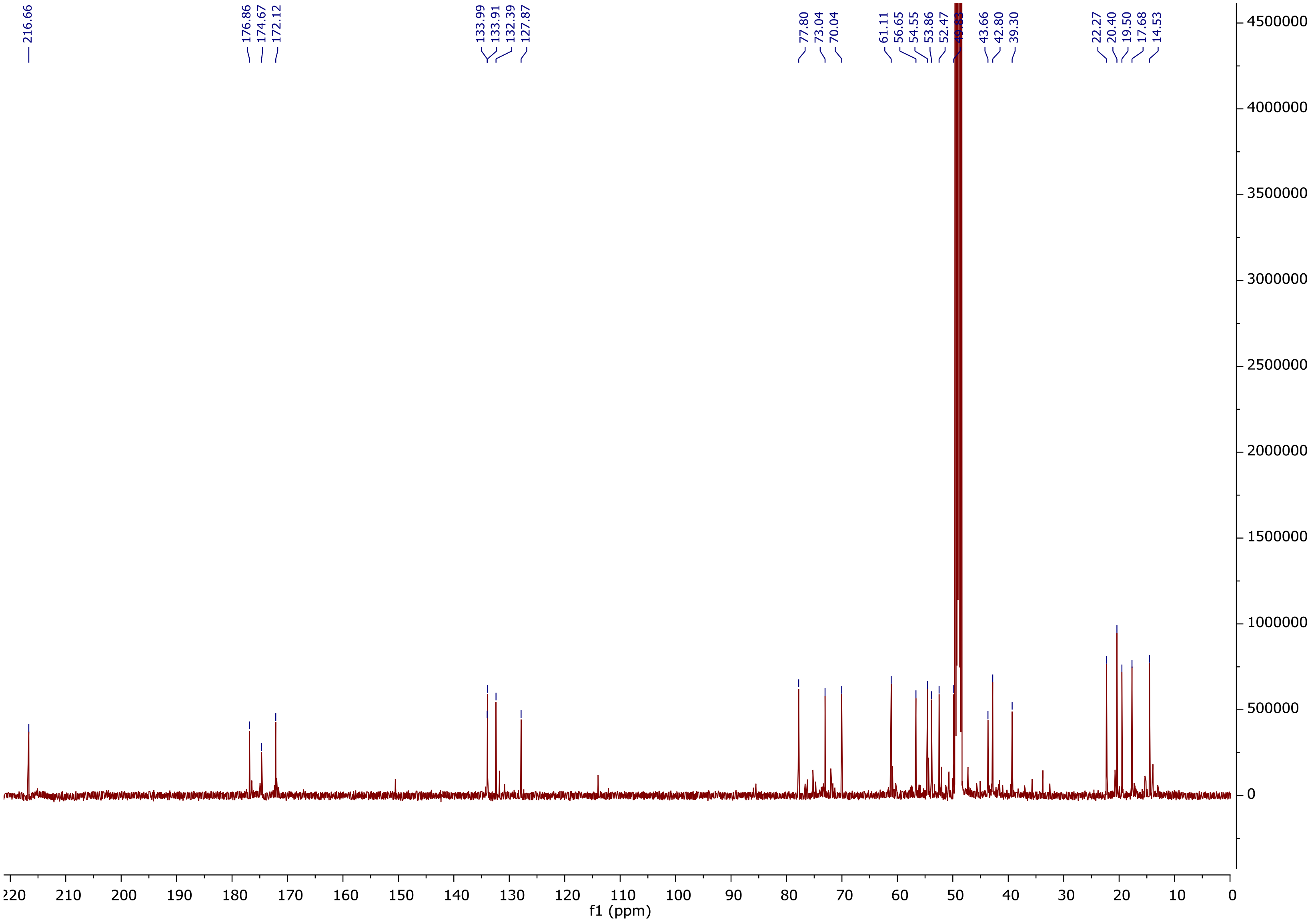

Figure SD150. ^{13}C NMR spectrum of xylachalasin A (**22**) (125 MHz, MeOH-d_4)

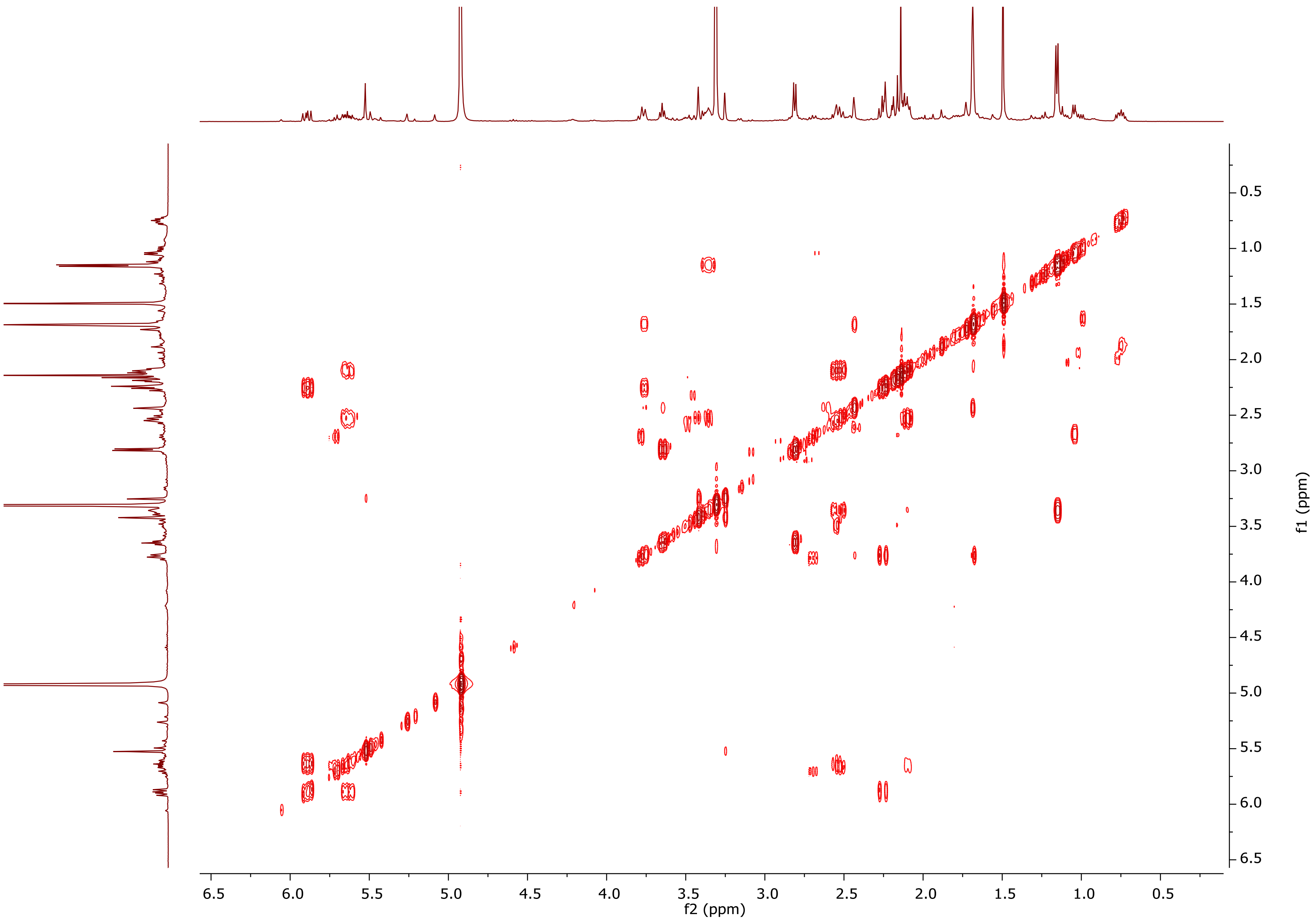

Figure SD151. ^1H-^1H COSY NMR spectrum of xylachalasin A (**22**) (500/500 MHz, MeOH-d_4)

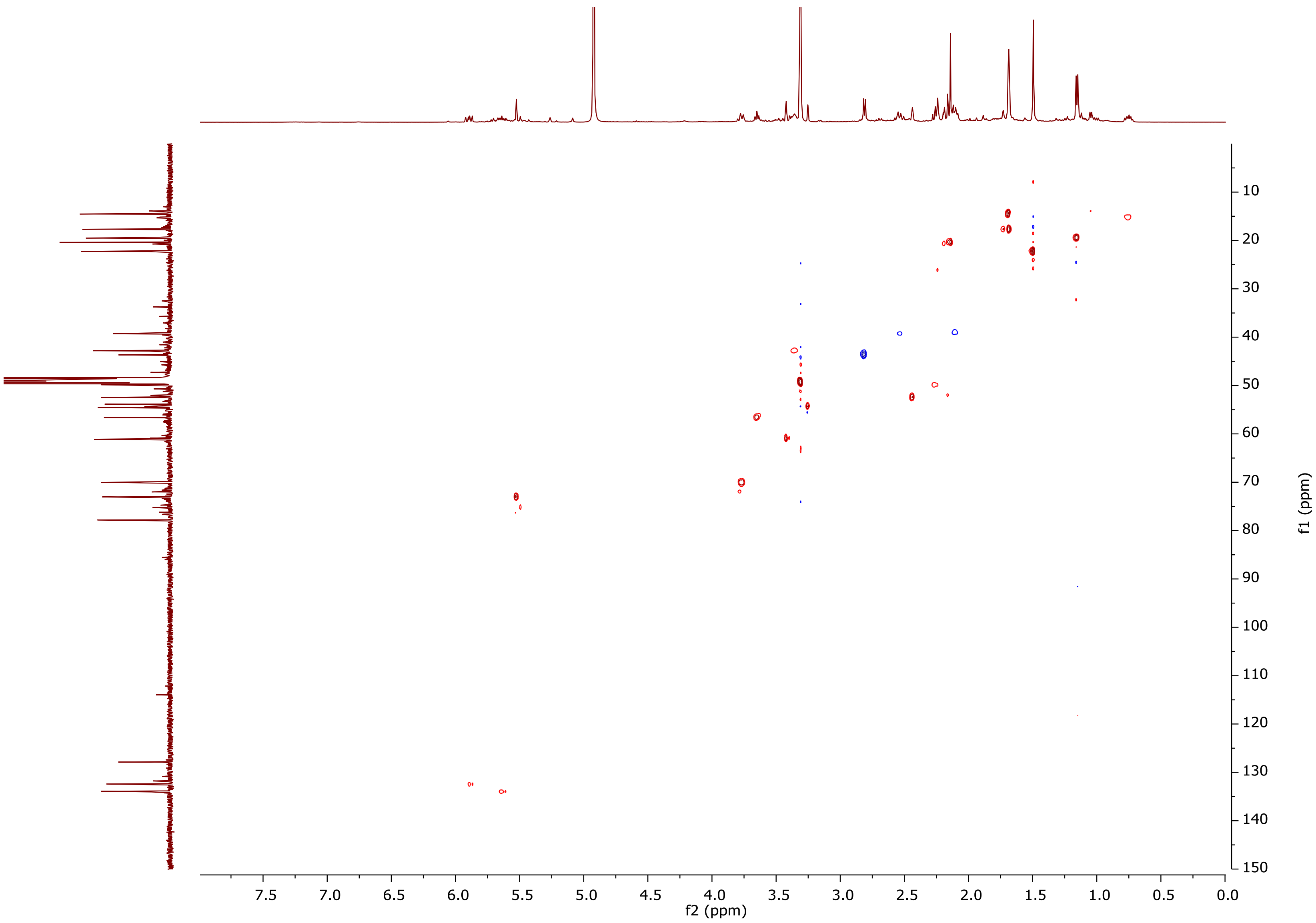

Figure SD152. ^{1}H-^{13}C HSQC NMR spectrum of xylachalasin A (**22**) (500/125 MHz, MeOH-d_4)

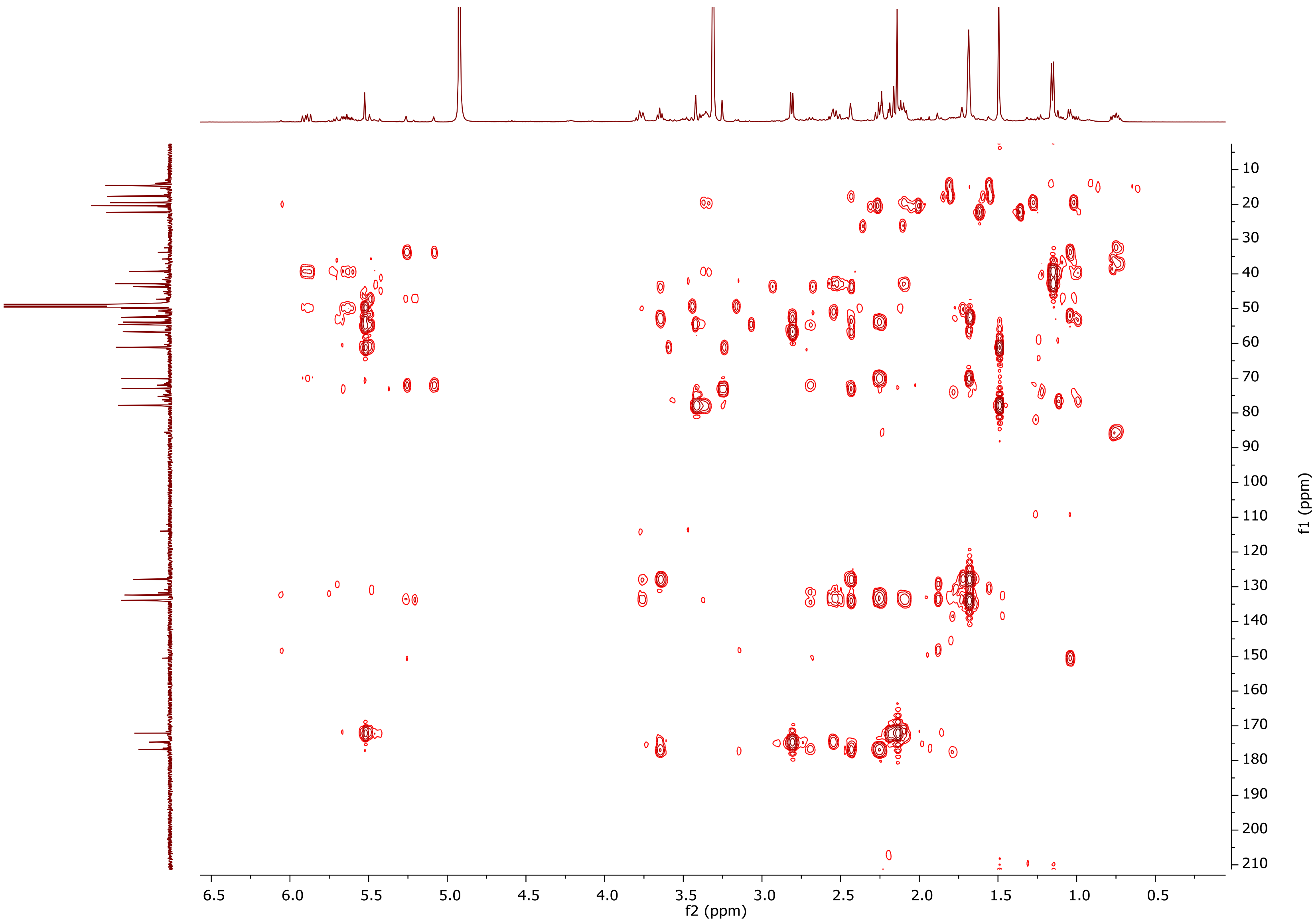

Figure SD153. ^{1}H-^{13}C HMBC NMR spectrum of xylachalasin A (**22**) (500/125 MHz, MeOH-d_4)

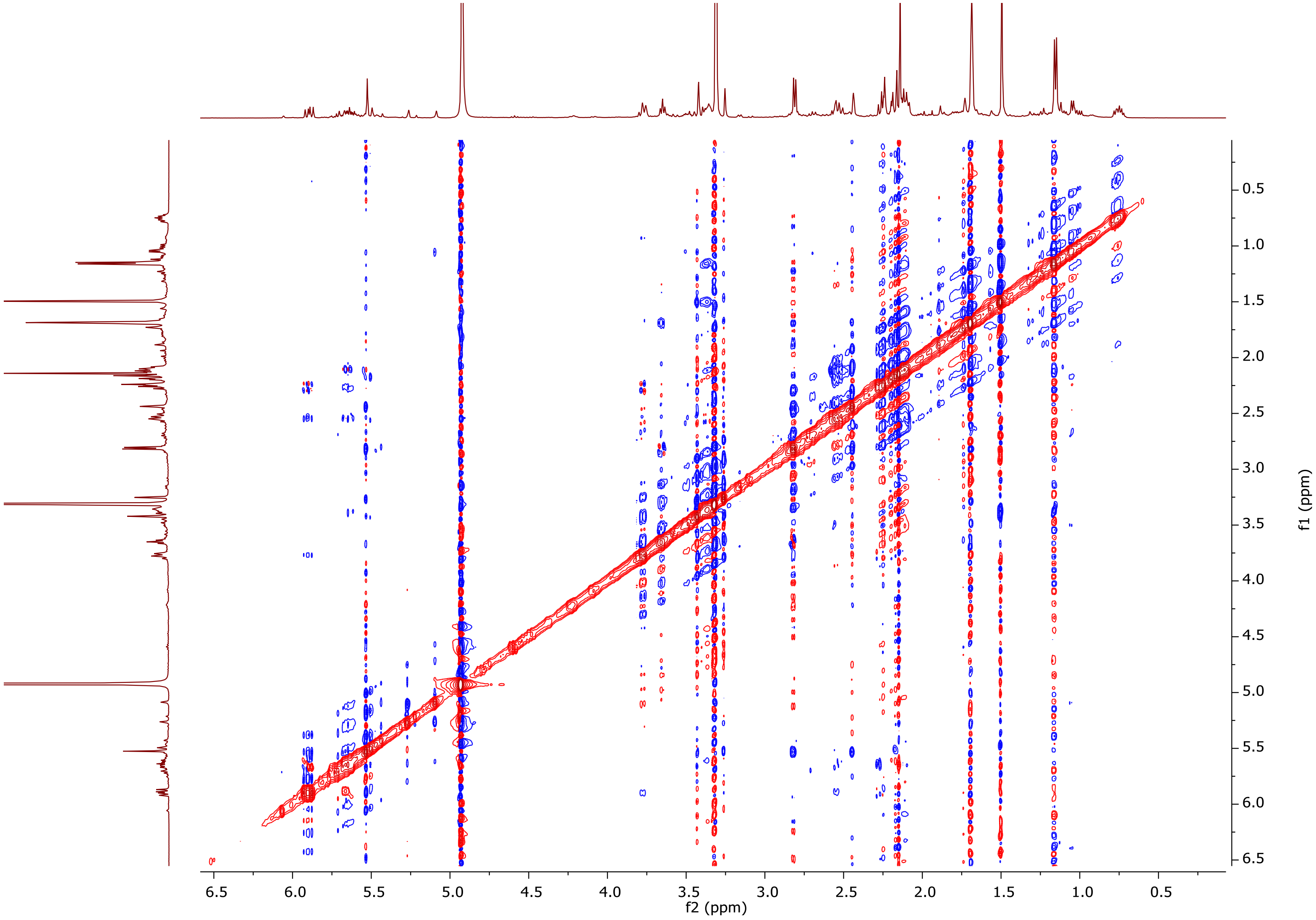

Figure SD154. ^{1}H-^{1}H NOESY NMR spectrum of xylachalasin A (**22**) (500/500 MHz, MeOH-d_4)

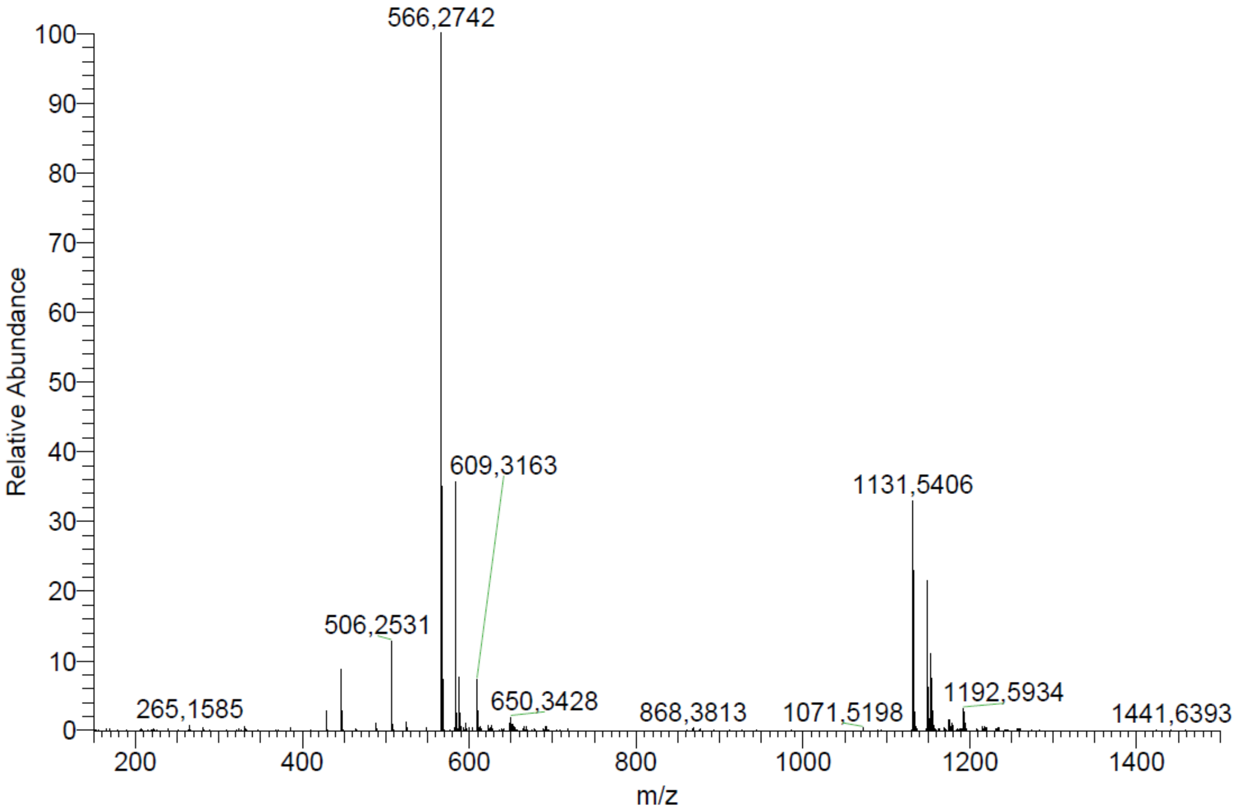

Figure SD155. ESI-HRMS spectrum of 7-acetyl-19,20-epoxycytochalasin C (**23**)

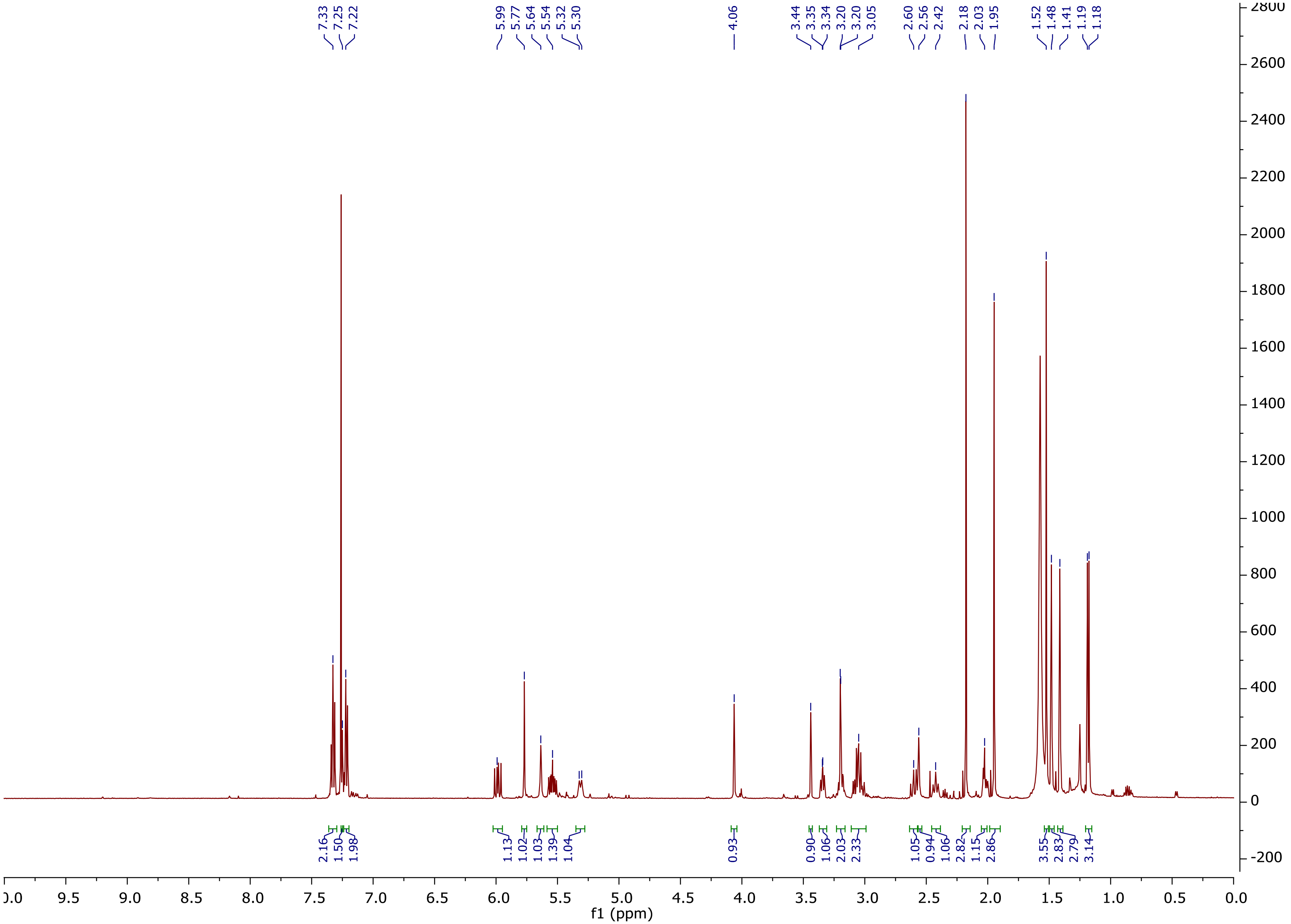

Figure SD156. ^{1}H NMR spectrum of 7-acetyl-19,20-epoxycytochalasin C (**23**) (500 MHz, $CDCl_3$)

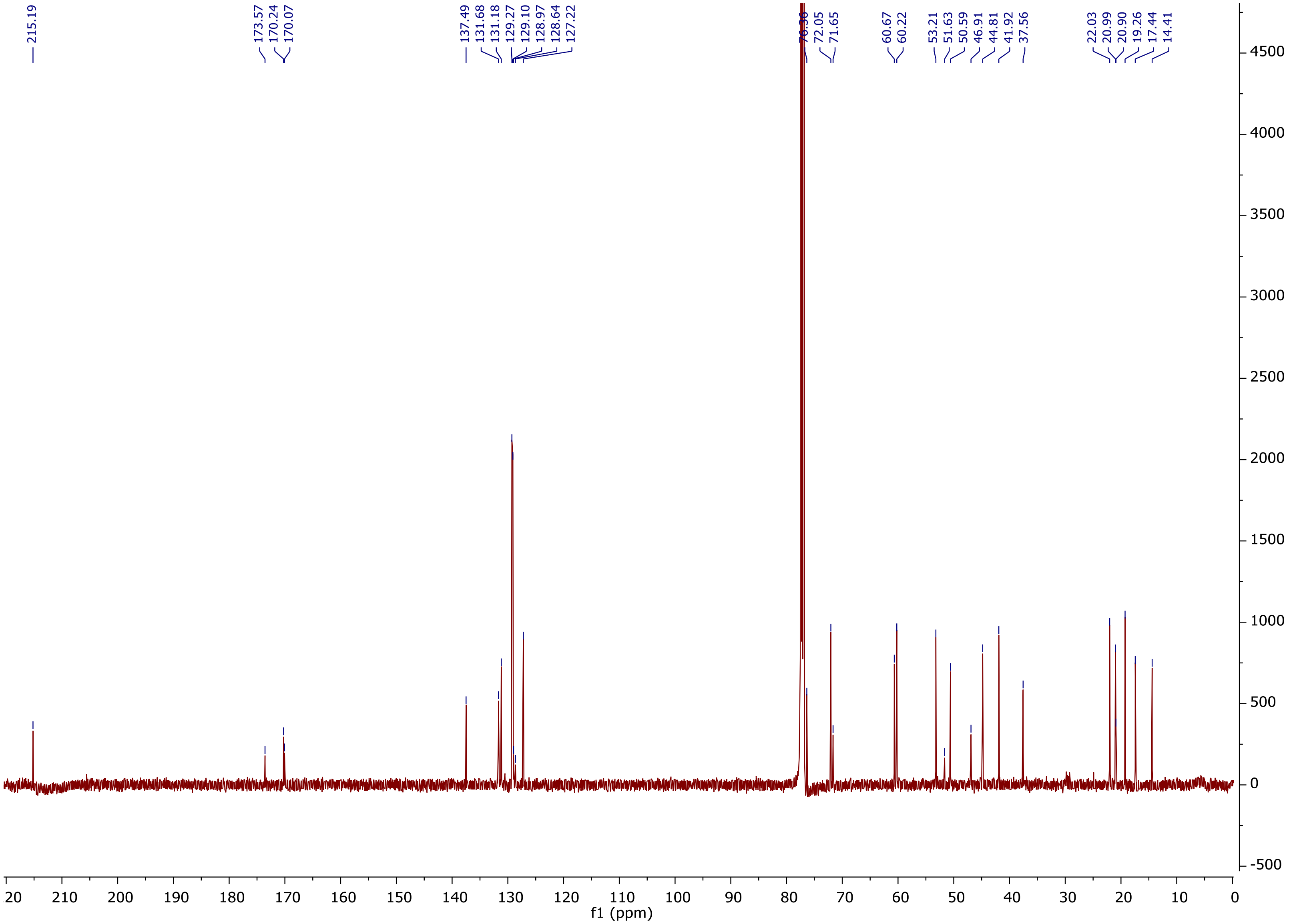

Figure SD157. ^{13}C NMR spectrum of 7-acetyl-19,20-epoxycytochalasin C (**23**) (125 MHz, $CDCl_3$)

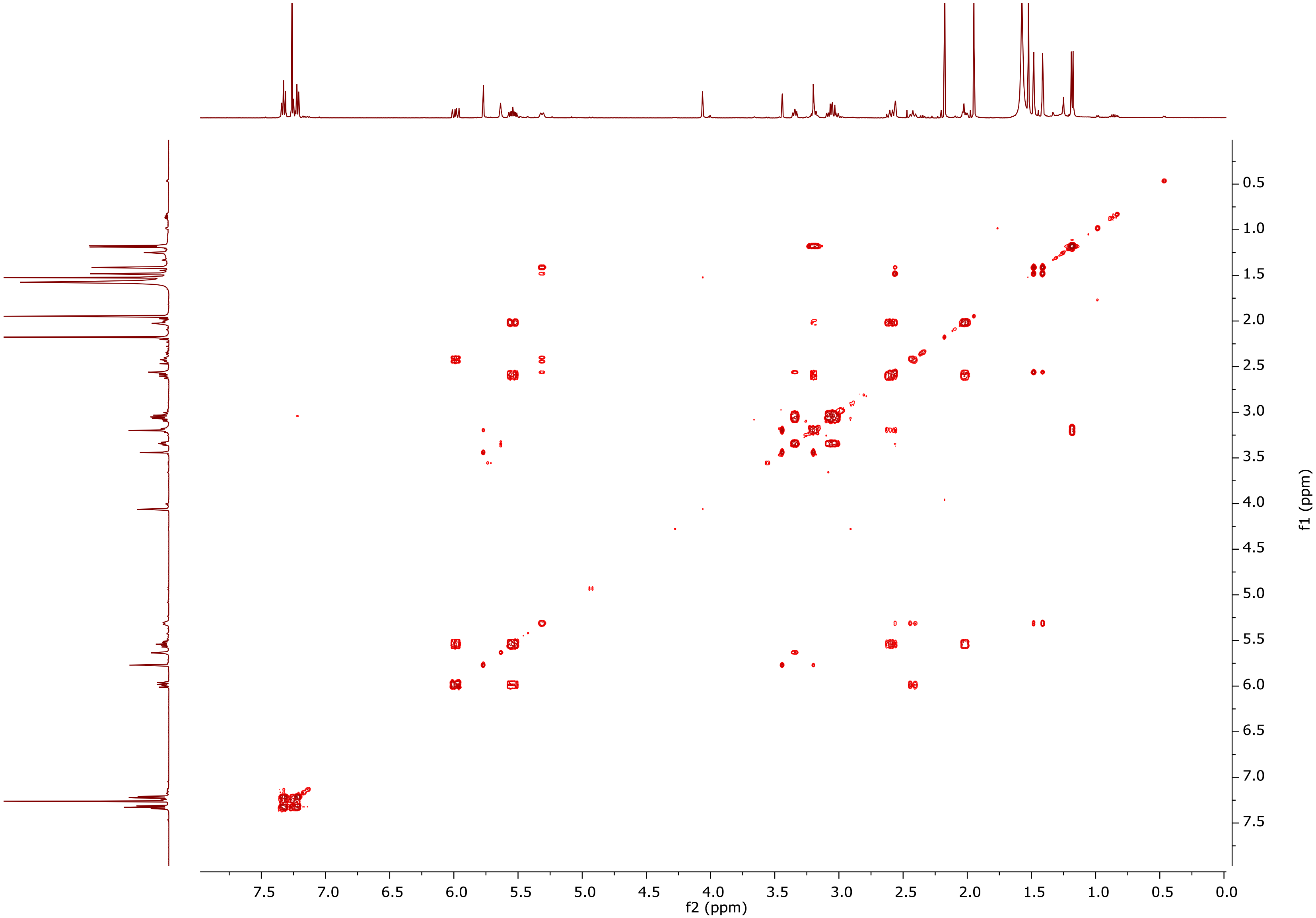

Figure SD158. ^{1}H-^{1}H COSY NMR spectrum of 7-acetyl-19,20-epoxycytochalasin C (**23**) (500/500 MHz, $CDCl_3$)

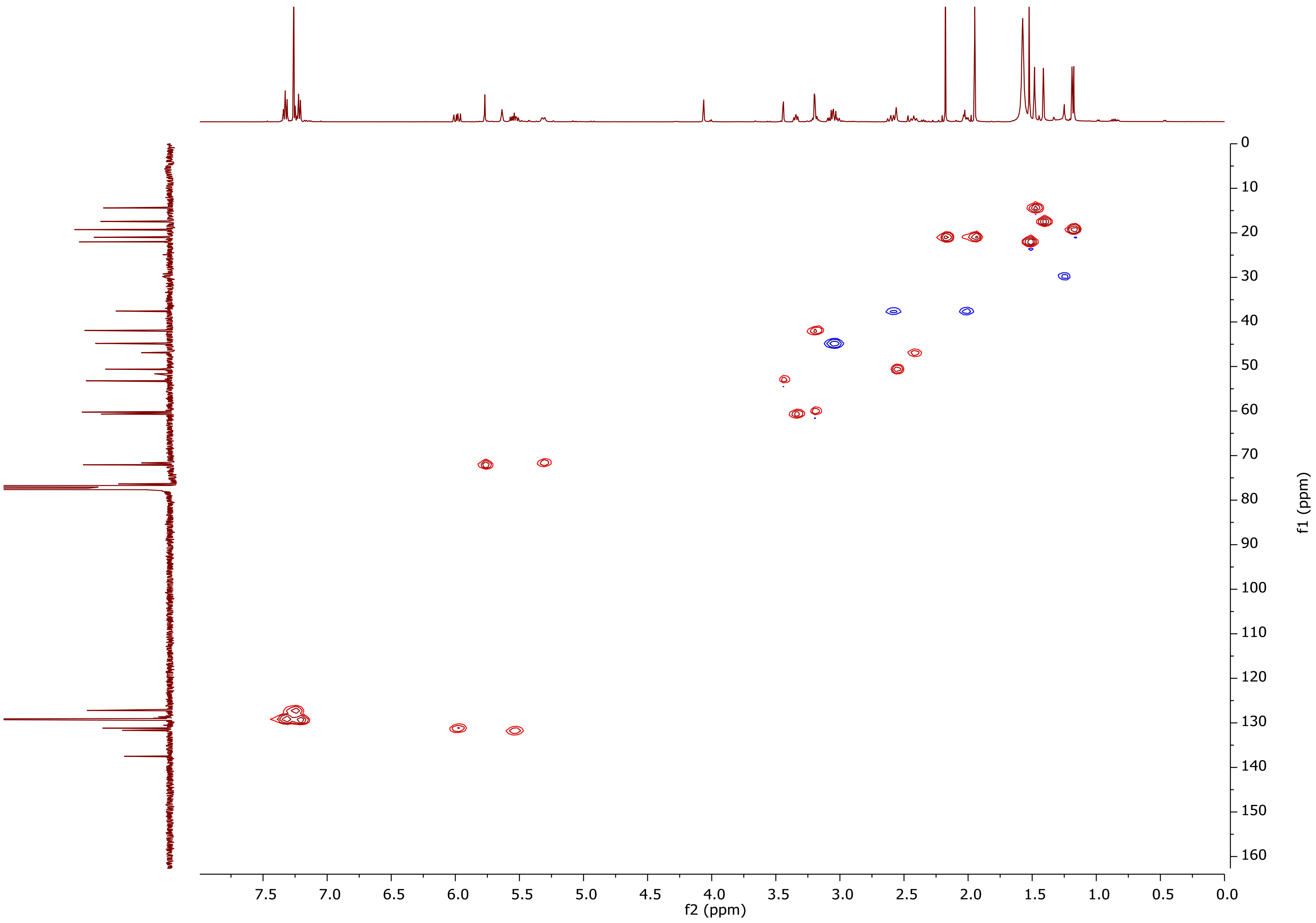

Figure SD159. ^{1}H-^{13}C HSQC NMR spectrum of 7-acetyl-19,20-epoxycytochalasin C (**23**) (500/125 MHz, $CDCl_3$)

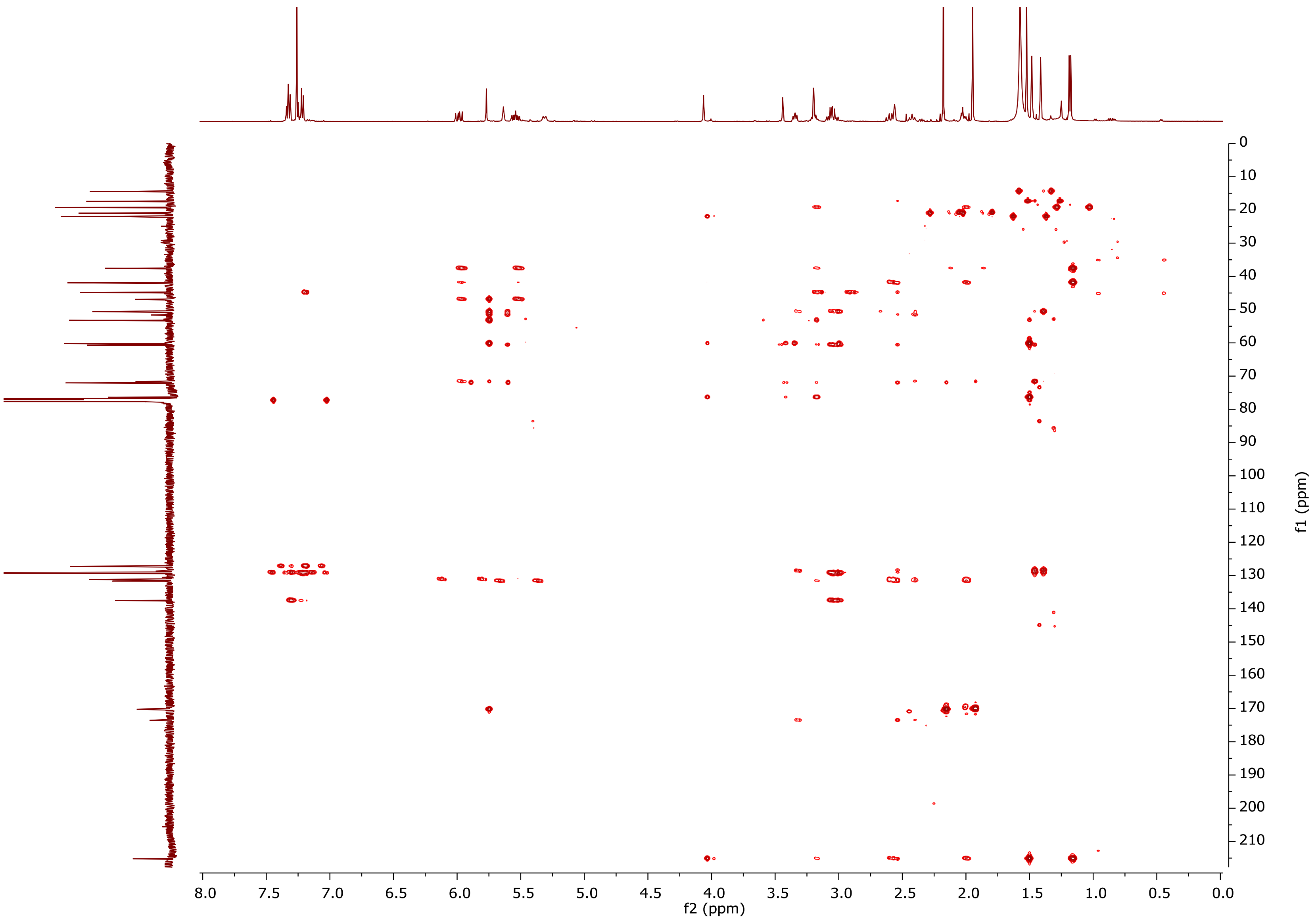

Figure SD160. ^{1}H-^{13}C HMBC NMR spectrum of 7-acetyl-19,20-epoxycytochalasin C (**23**) (500/125 MHz, $CDCl_3$)

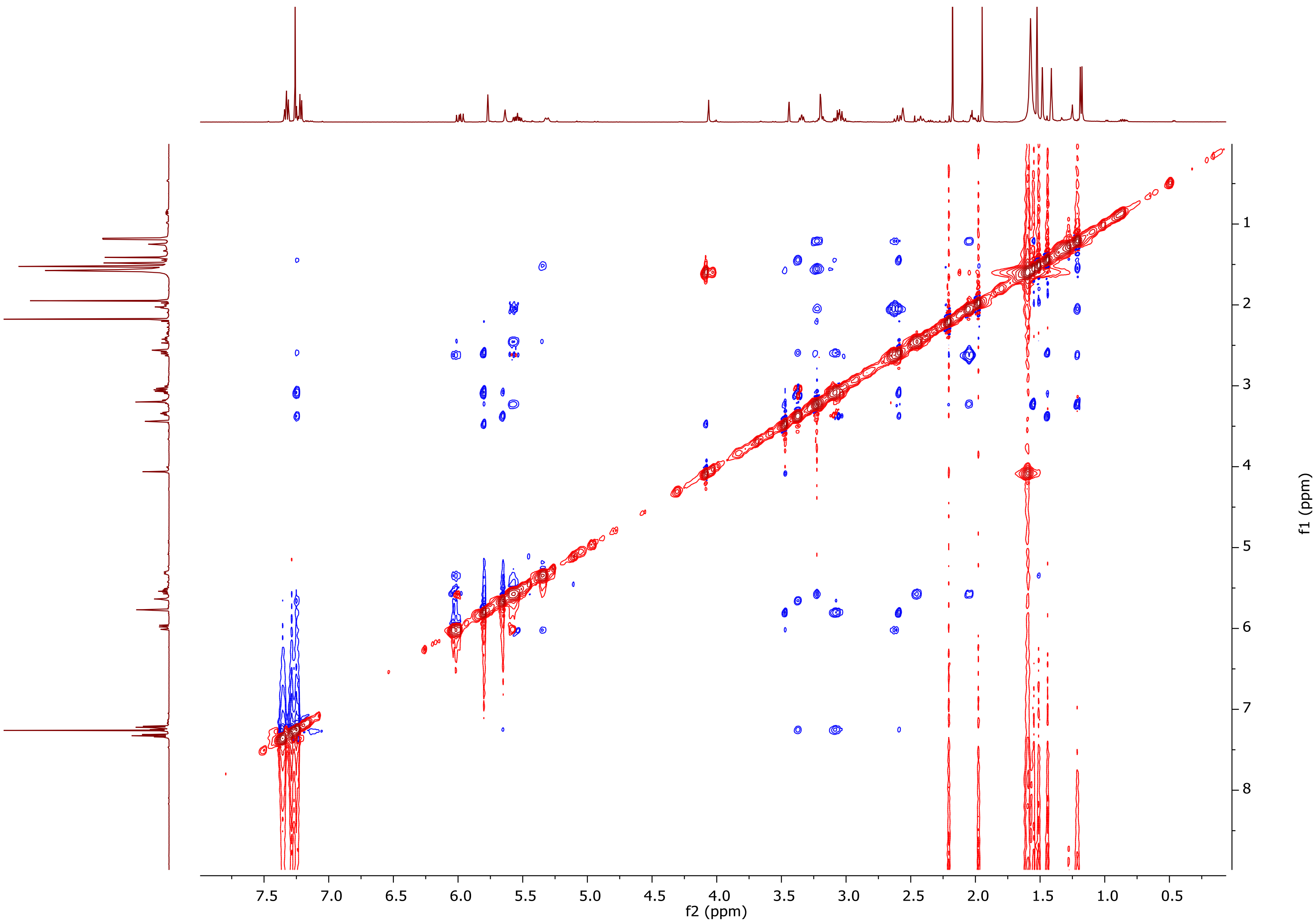

Figure SD161. ^{1}H-^{1}H NOESY NMR spectrum of 7-acetyl-19,20-epoxycytochalasin C (**23**) (500/500 MHz, $CDCl_3$)

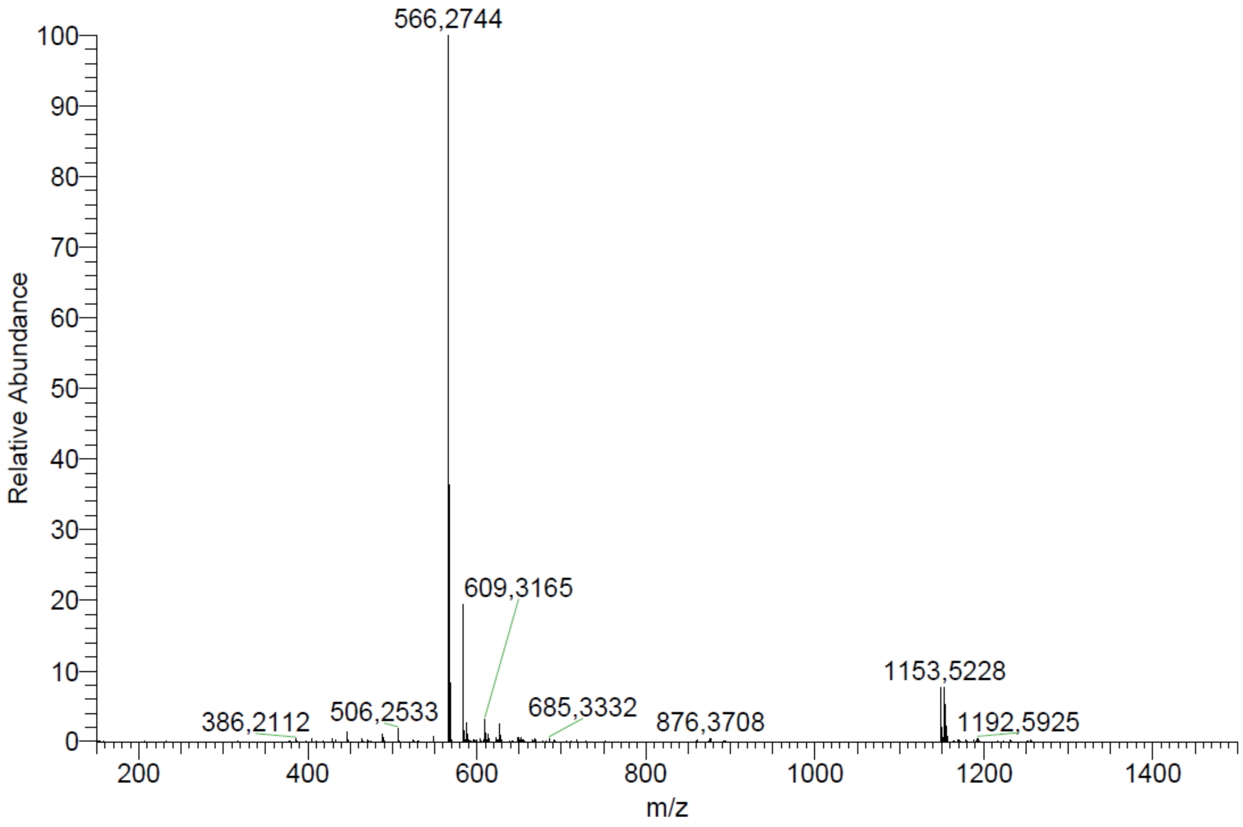

Figure SD162. ESI-HRMS spectrum of *N*-acetyl-19,20-epoxycytochalasin Q (**23**) in $CDCl_3$

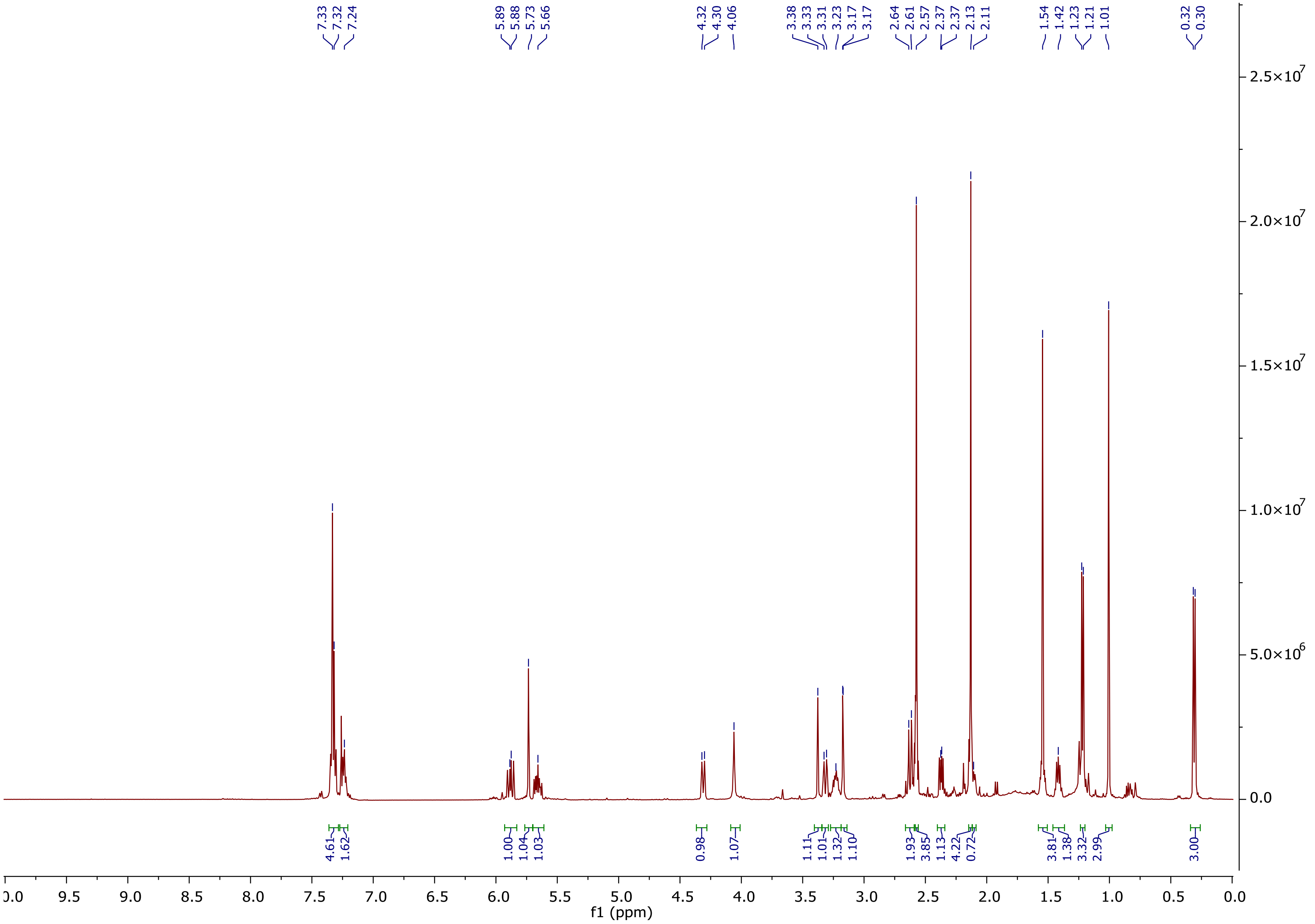

Figure SD163. ^{1}H NMR spectrum of *N*-acetyl-19,20-epoxycytochalasin Q (**23**) (500 MHz, $CDCl_3$)

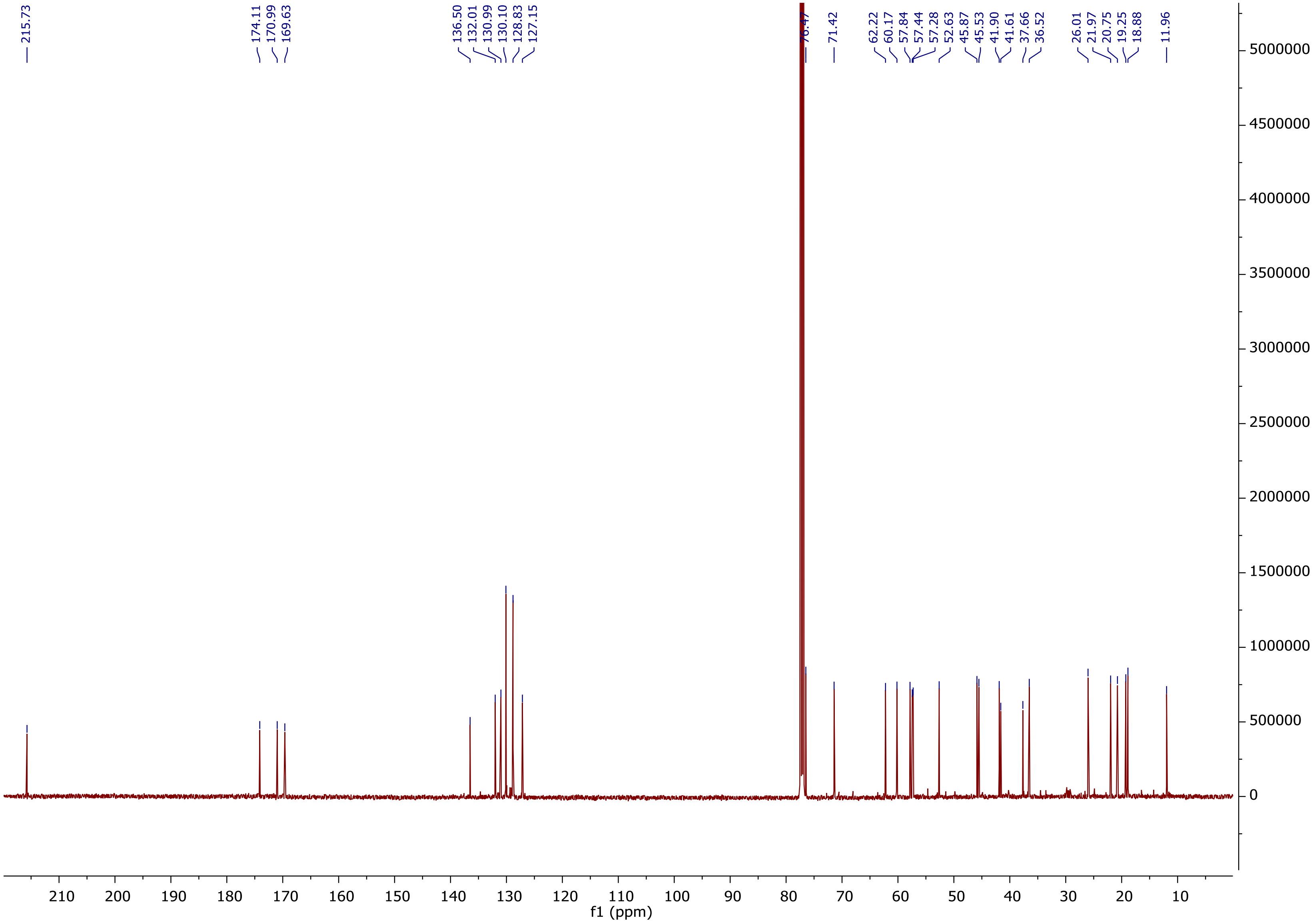

Figure SD164. ^{13}C NMR spectrum of *N*-acetyl-19,20-epoxycytochalasin Q (**23**) (125 MHz, $CDCl_3$)

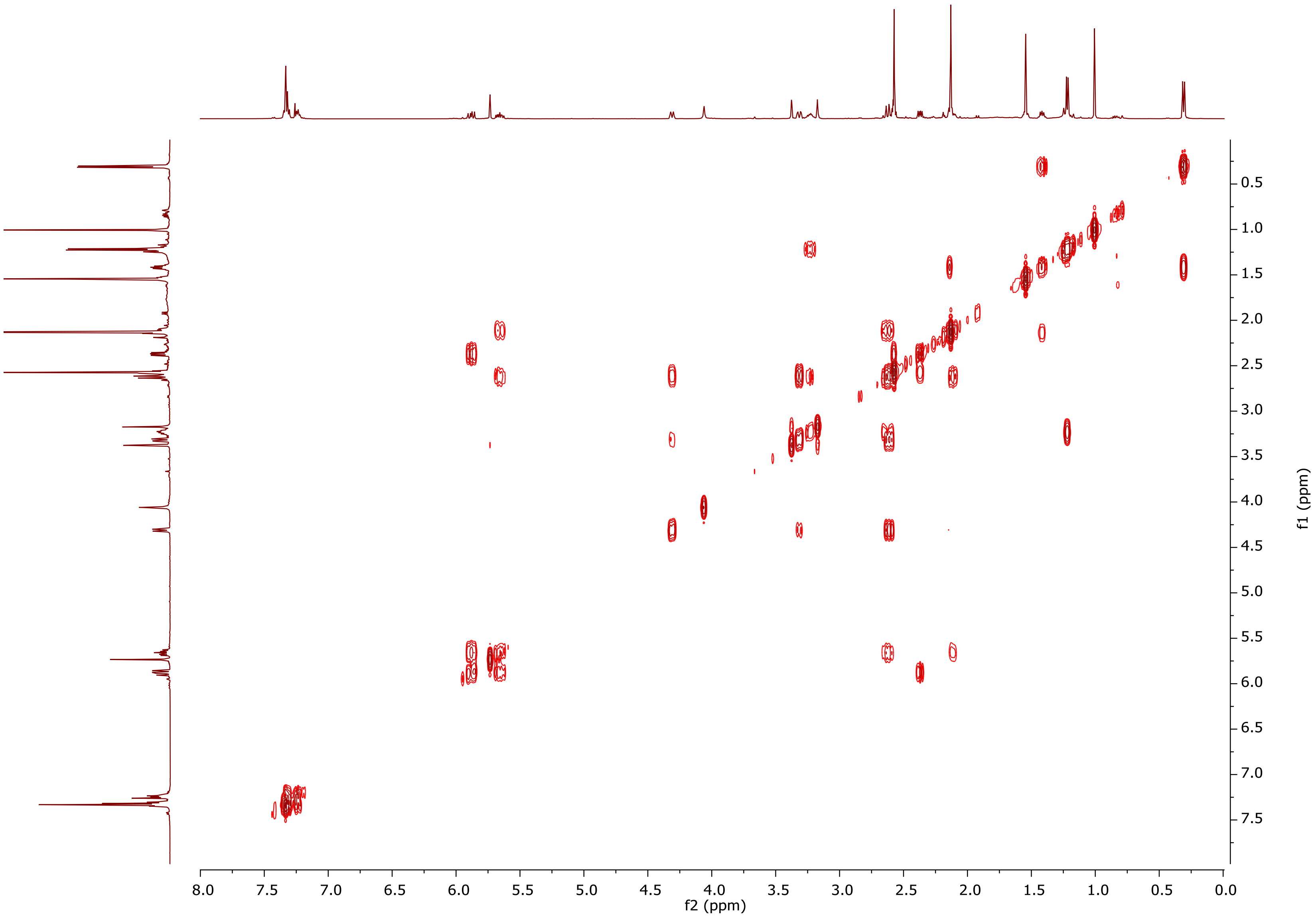

Figure SD165. ^{1}H-^{1}H COSY NMR spectrum of *N*-acetyl-19,20-epoxycytochalasin Q (**23**) (500/500 MHz, $CDCl_3$)

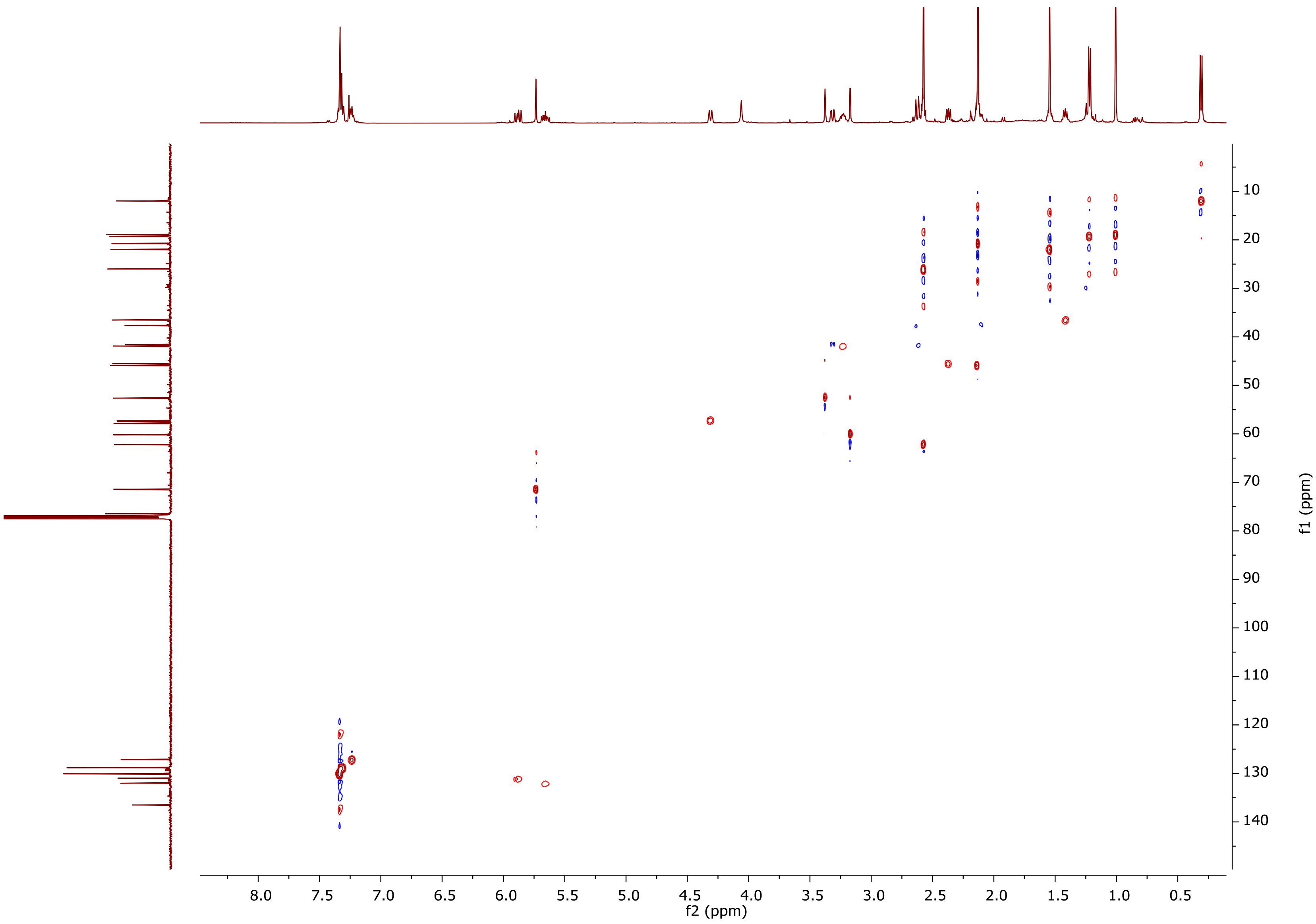

Figure SD166. ^{1}H-^{13}C HSQC NMR spectrum of *N*-acetyl-19,20-epoxycytochalasin Q (**23**) (500/125 MHz, $CDCl_3$)

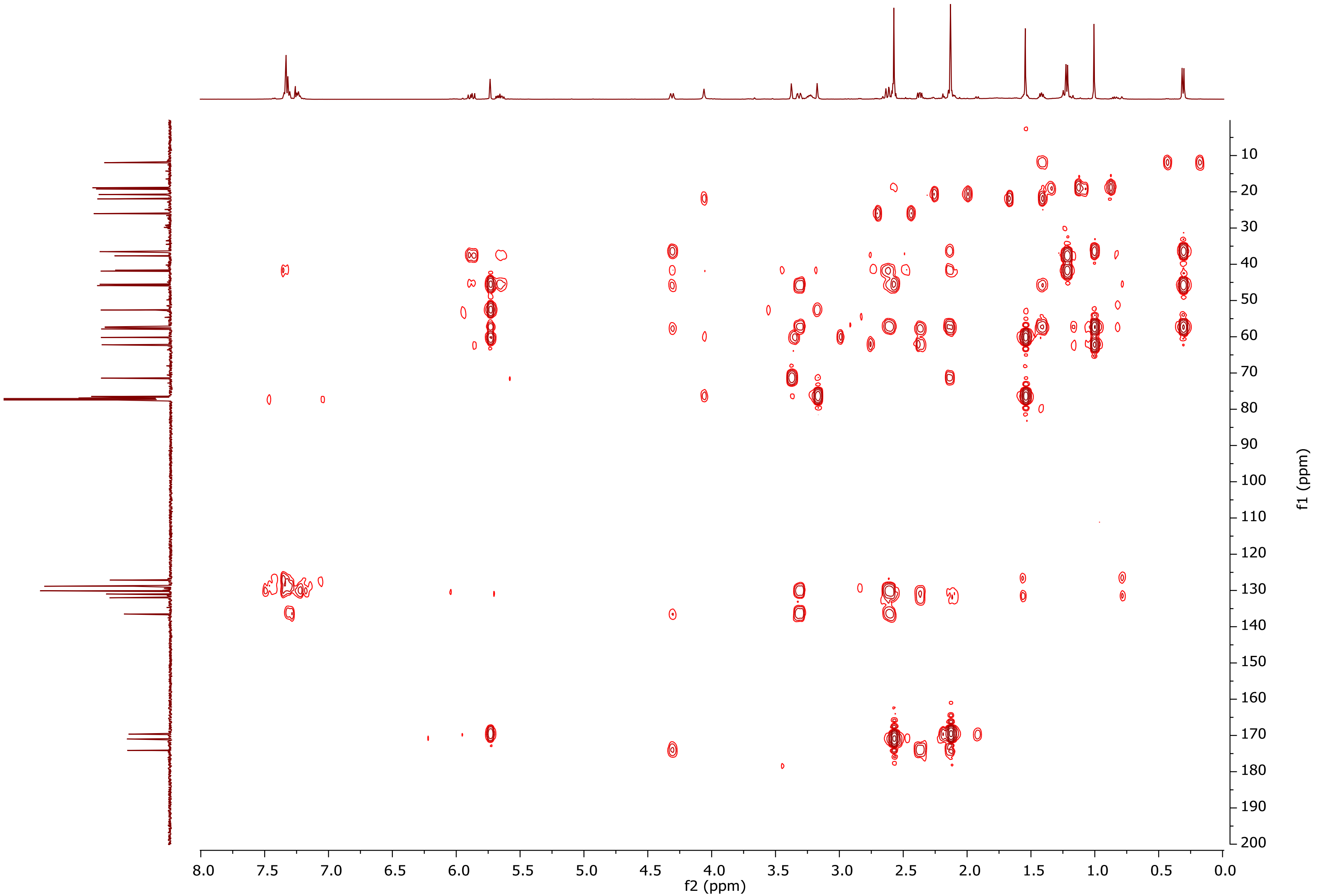

Figure SD167. ^{1}H-^{13}C HMBC NMR spectrum of *N*-acetyl-19,20-epoxycytochalasin Q (**23**) (500/125 MHz, $CDCl_3$)

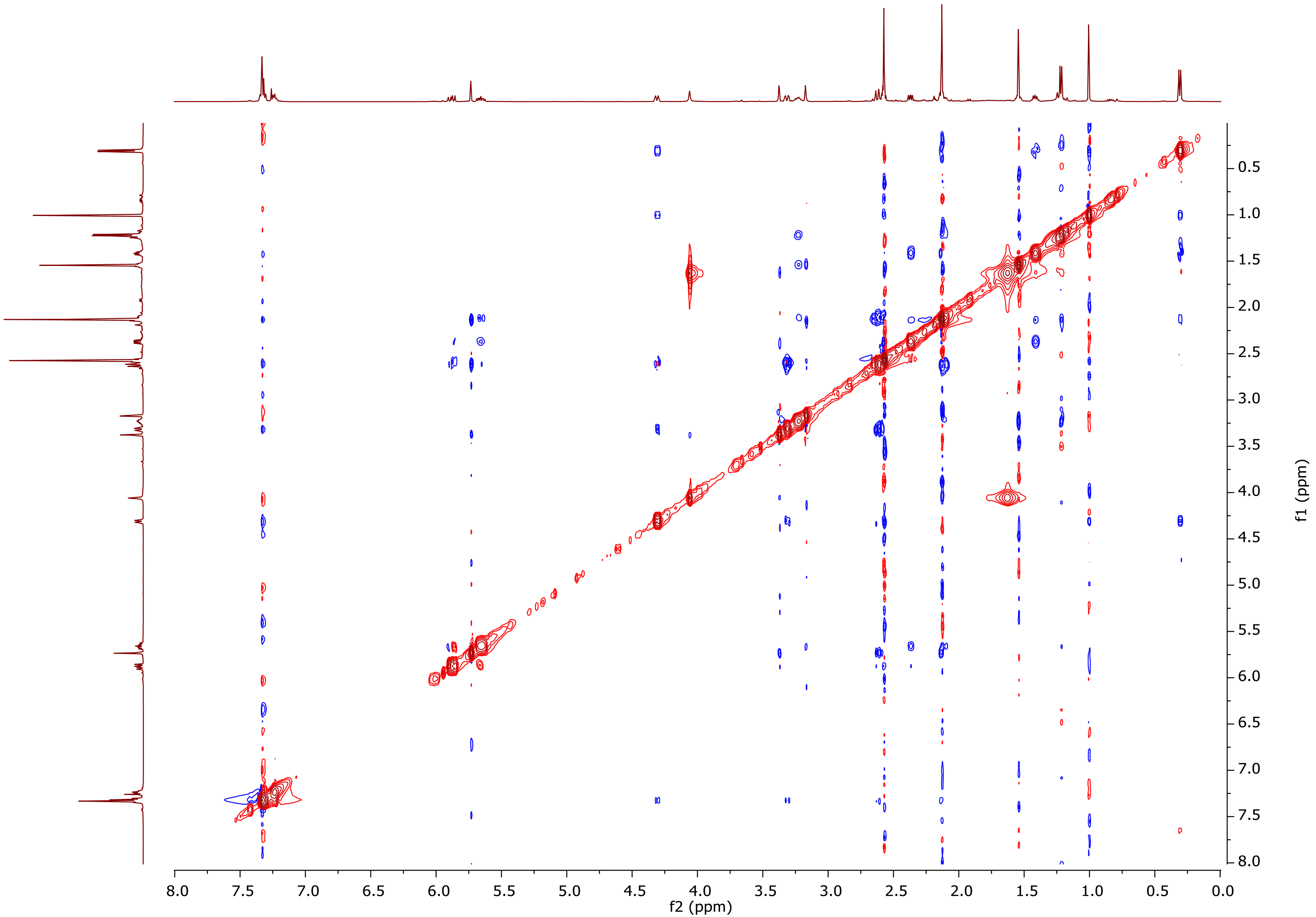

Figure SD168. ^1H-^1H NOESY NMR spectrum of *N*-acetyl-19,20-epoxycytochalasin Q (**23**) (500/500 MHz, $CDCl_3$)

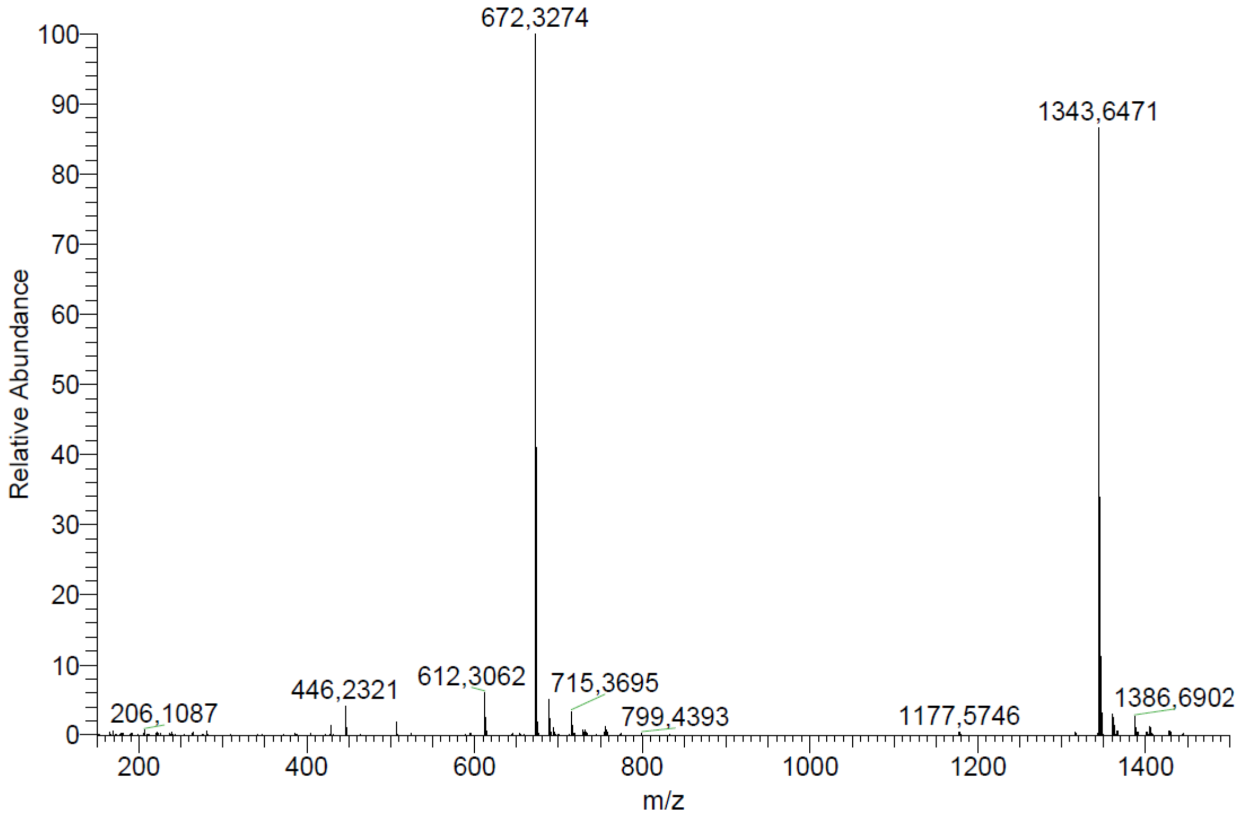

Figure SD169. ESI-HRMS spectrum of 7-diazirin-19,20-epoxycytochalasin C (**25**)

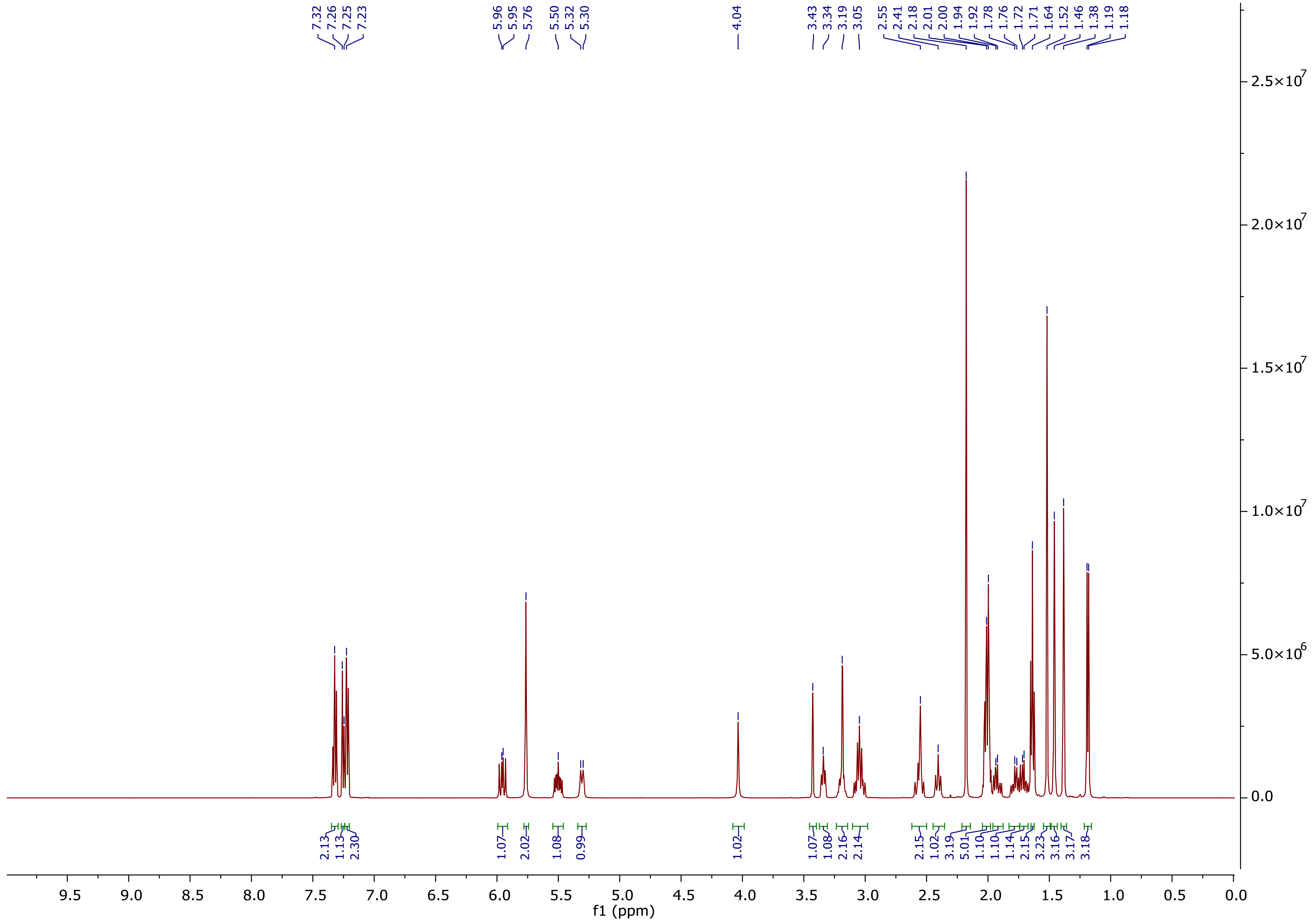

Figure SD170. ^{1}H NMR spectrum of 7-diazirin-19,20-epoxycytochalasin C (**25**) (500 MHz, $CDCl_3$)

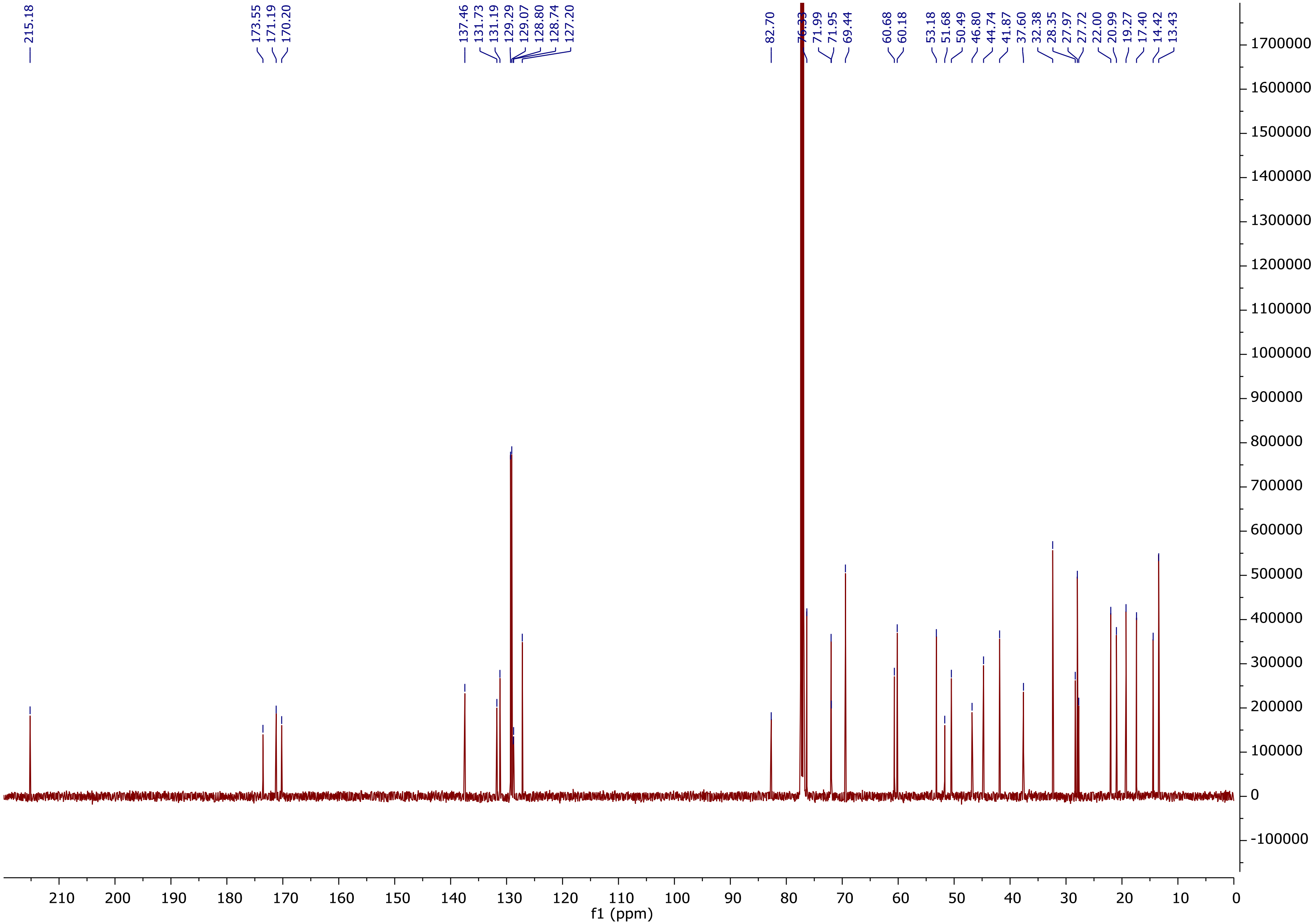

Figure SD171. ^{13}C NMR spectrum of 7-diazirin-19,20-epoxycytochalasin C (**25**) (125 MHz, $CDCl_3$)

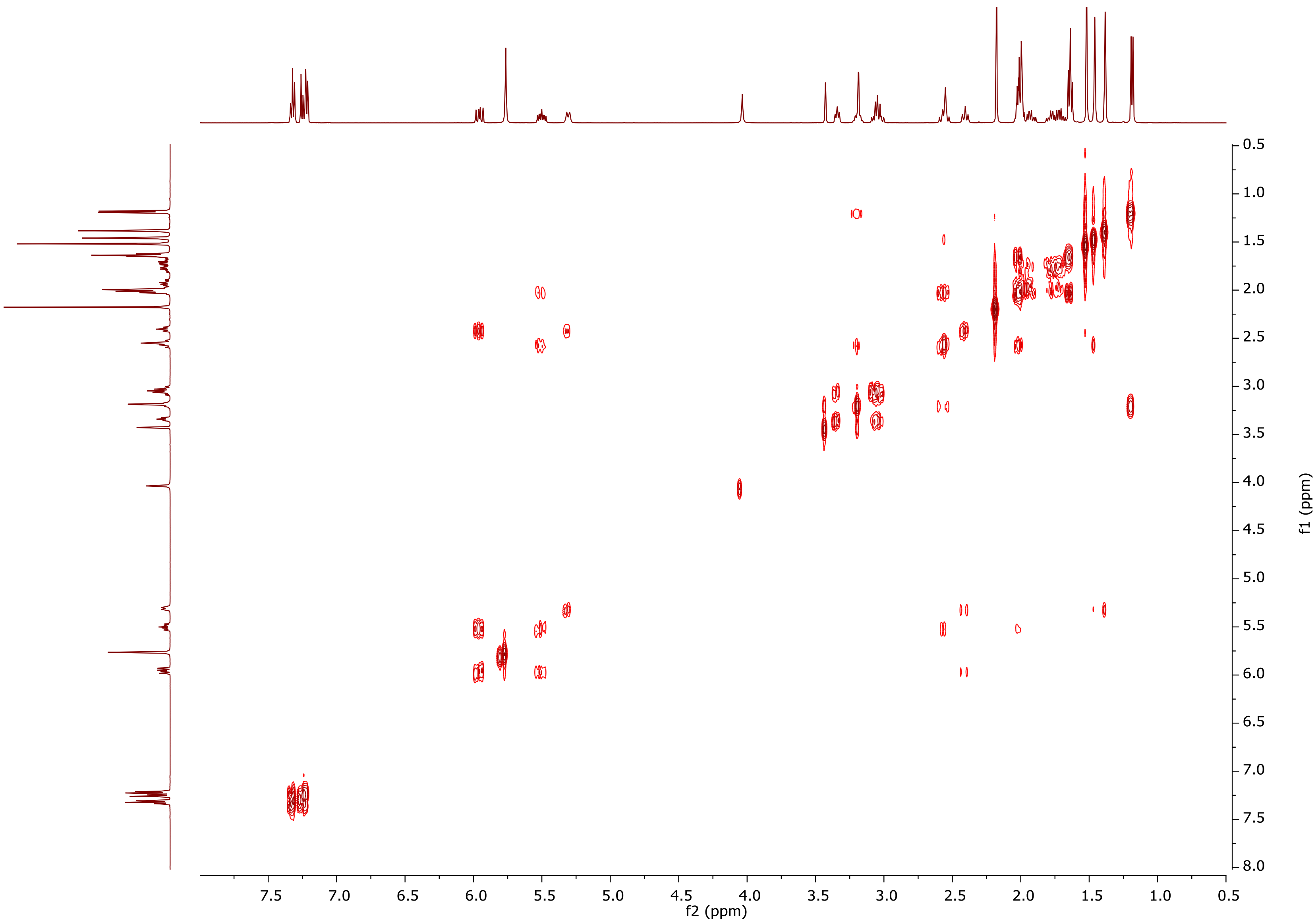

Figure SD172. ^{1}H-^{1}H COSY NMR spectrum of 7-diazirin-19,20-epoxycytochalasin C (**25**) (500/500 MHz, $CDCl_3$)

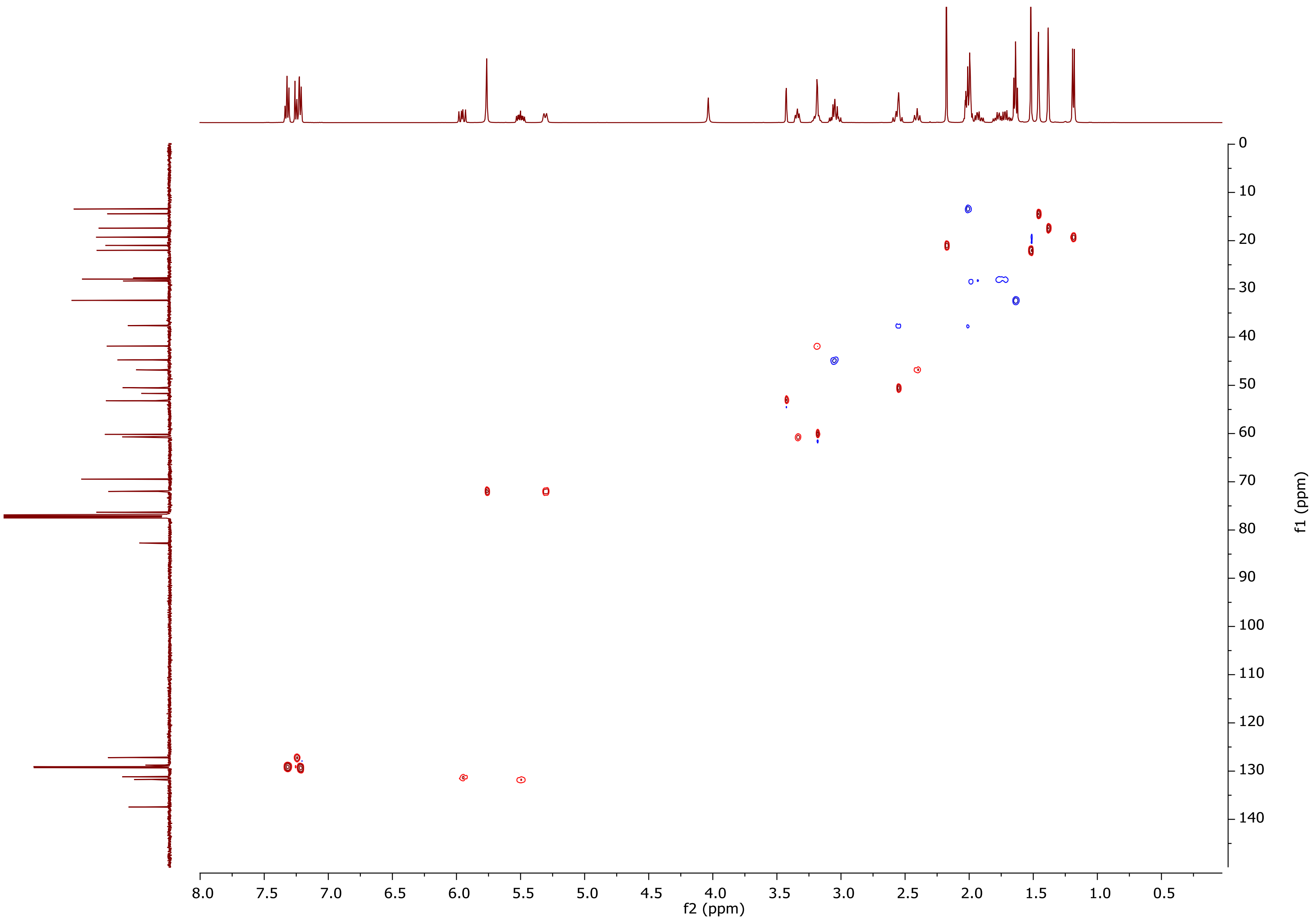

Figure SD173. ^{1}H-^{13}C HSQC NMR spectrum of 7-diazirin-19,20-epoxycytochalasin C (**25**) (500/125 MHz, $CDCl_3$)

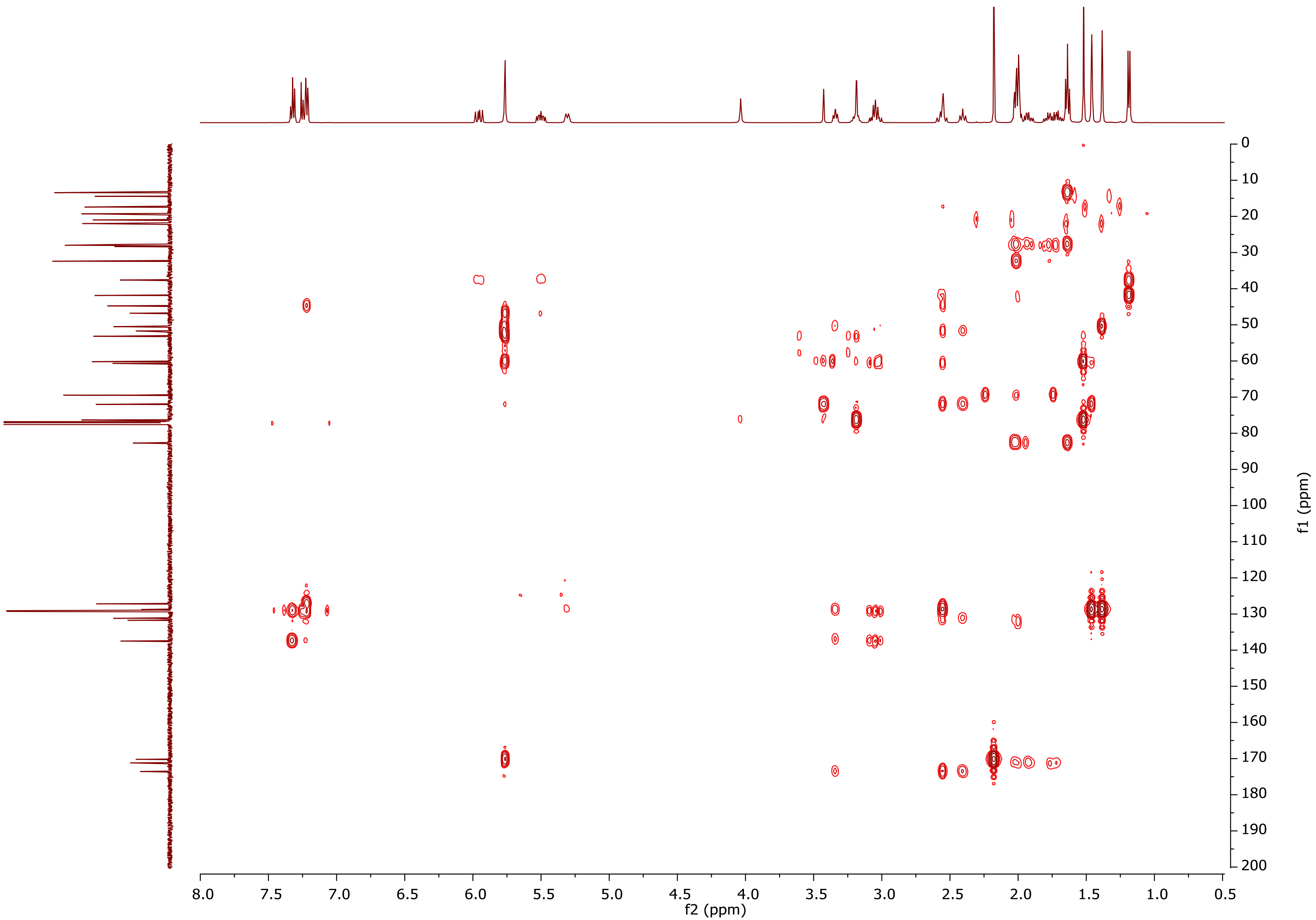

Figure SD174. ^{1}H-^{13}C HMBC NMR spectrum of 7-diazirin-19,20-epoxycytochalasin C (**25**) (500/125 MHz, $CDCl_3$)

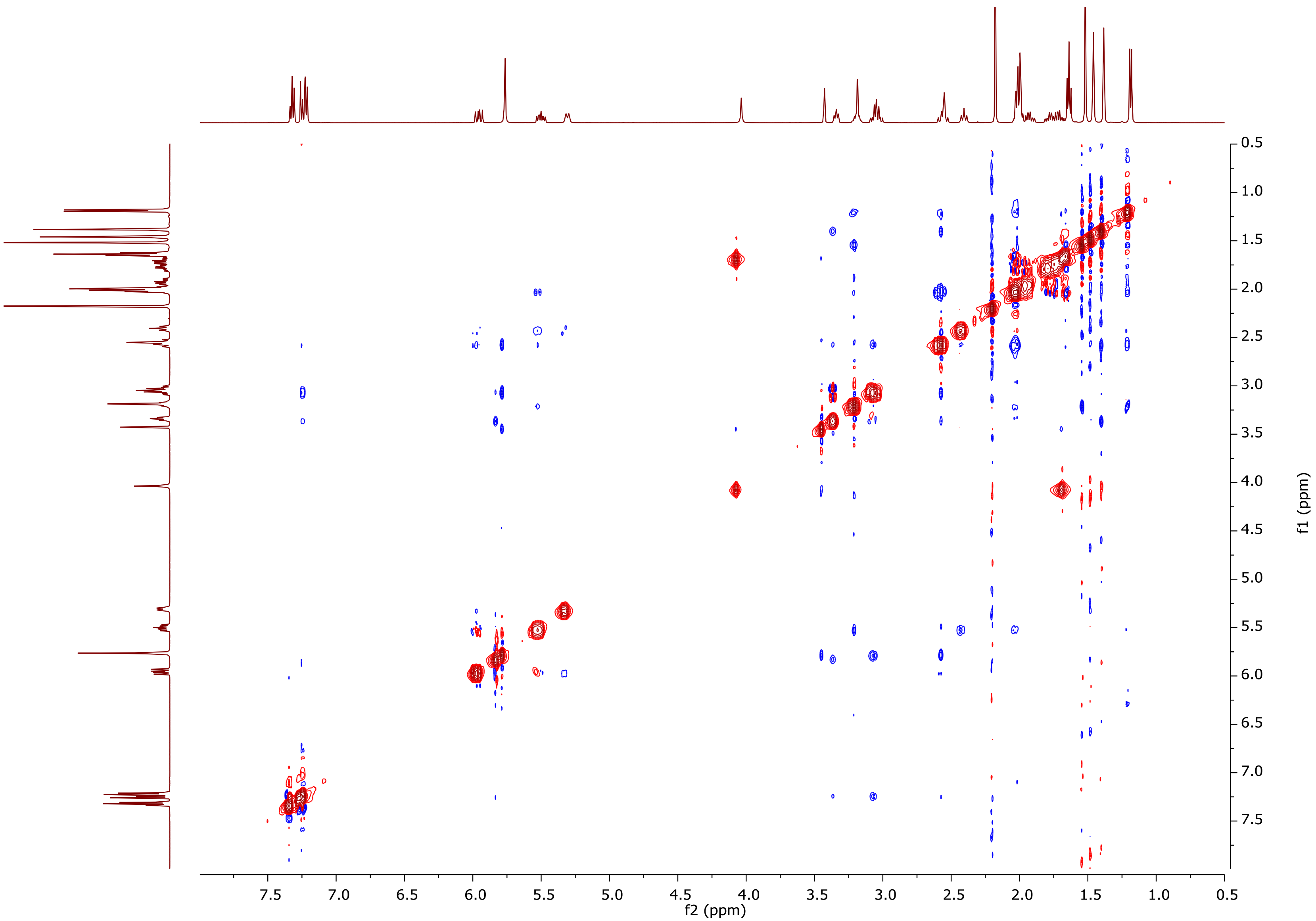

Figure SD175. ^{1}H-^{1}H NOESY NMR spectrum of 7-diazirin-19,20-epoxycytochalasin C (**25**) (500/500 MHz, $CDCl_3$)

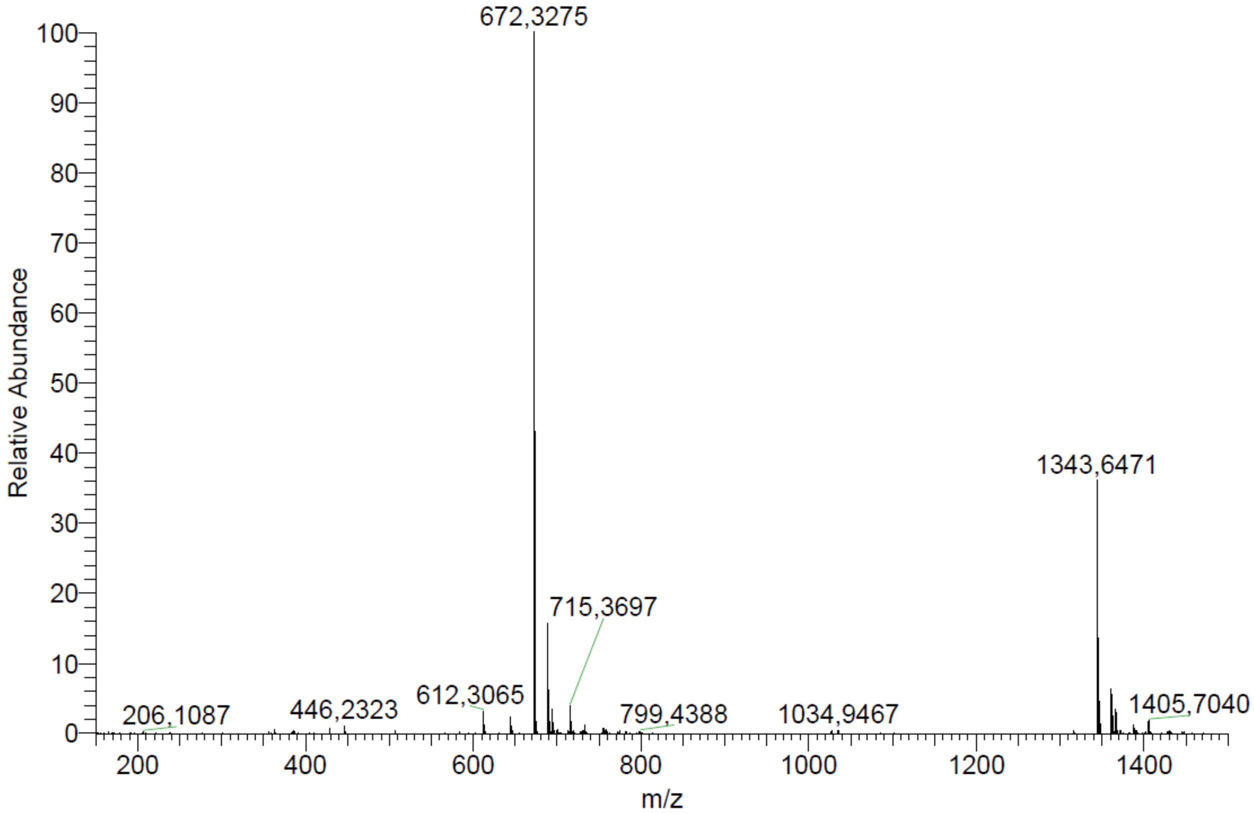

Figure SD176. ESI-HRMS spectrum of 7-diazirin-19,20-epoxycytochalasin D (**26**)

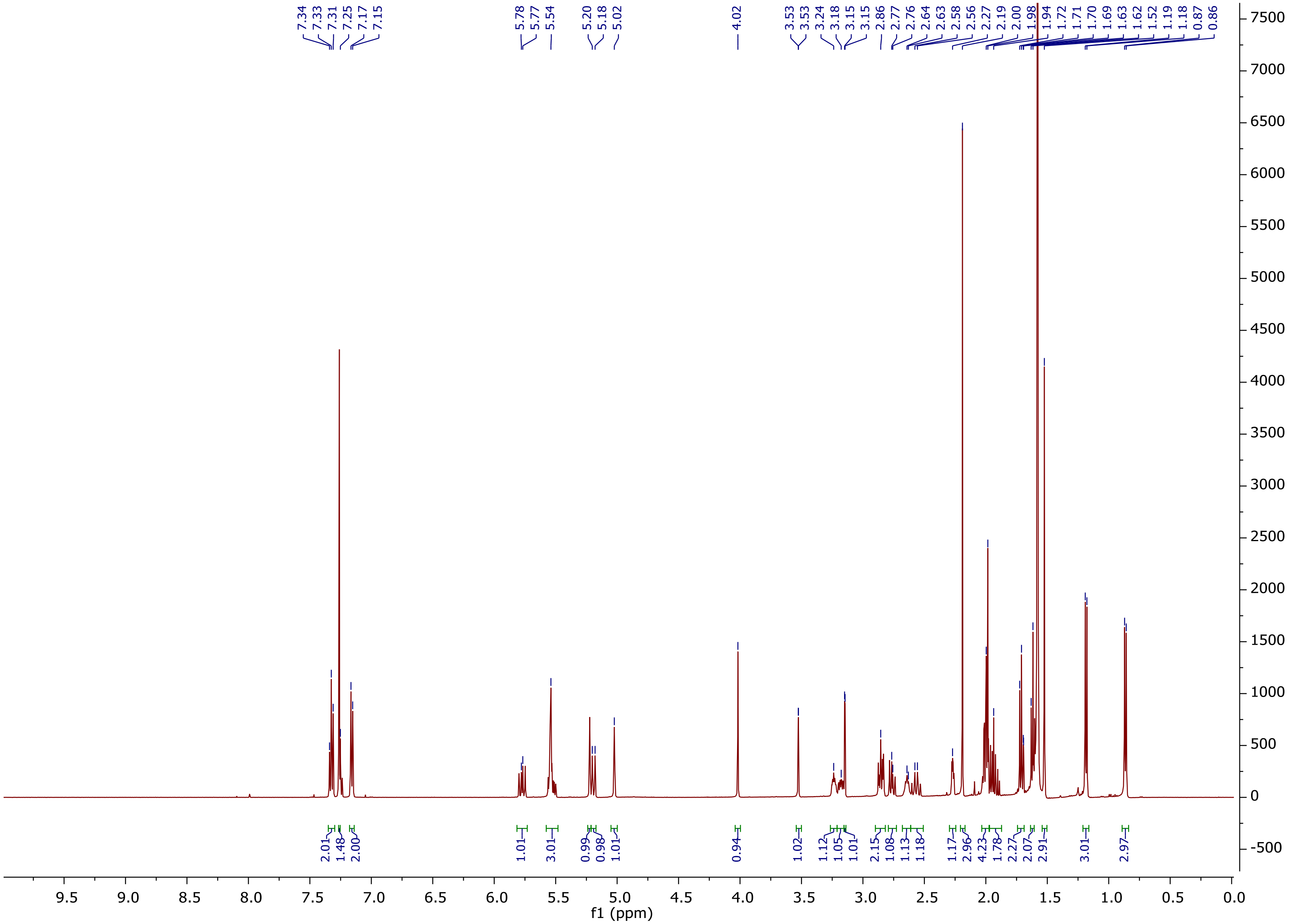

Figure SD177. ^{1}H NMR spectrum of 7-diazirin-19,20-epoxycytochalasin D (**26**) (500 MHz, $CDCl_3$)

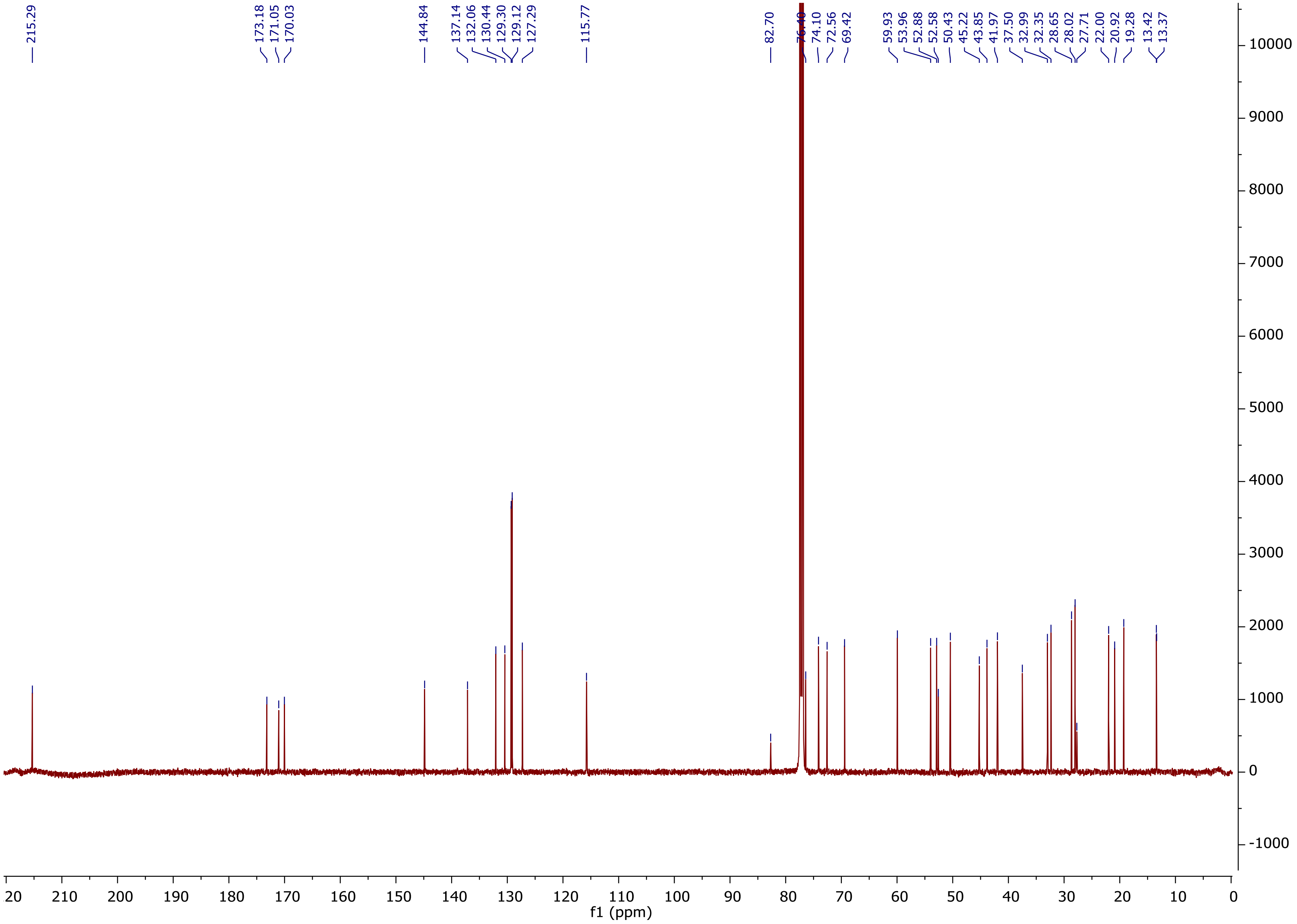

Figure SD178. ^{13}C NMR spectrum of 7-diazirin-19,20-epoxycytochalasin D (**26**) (125 MHz, $CDCl_3$)

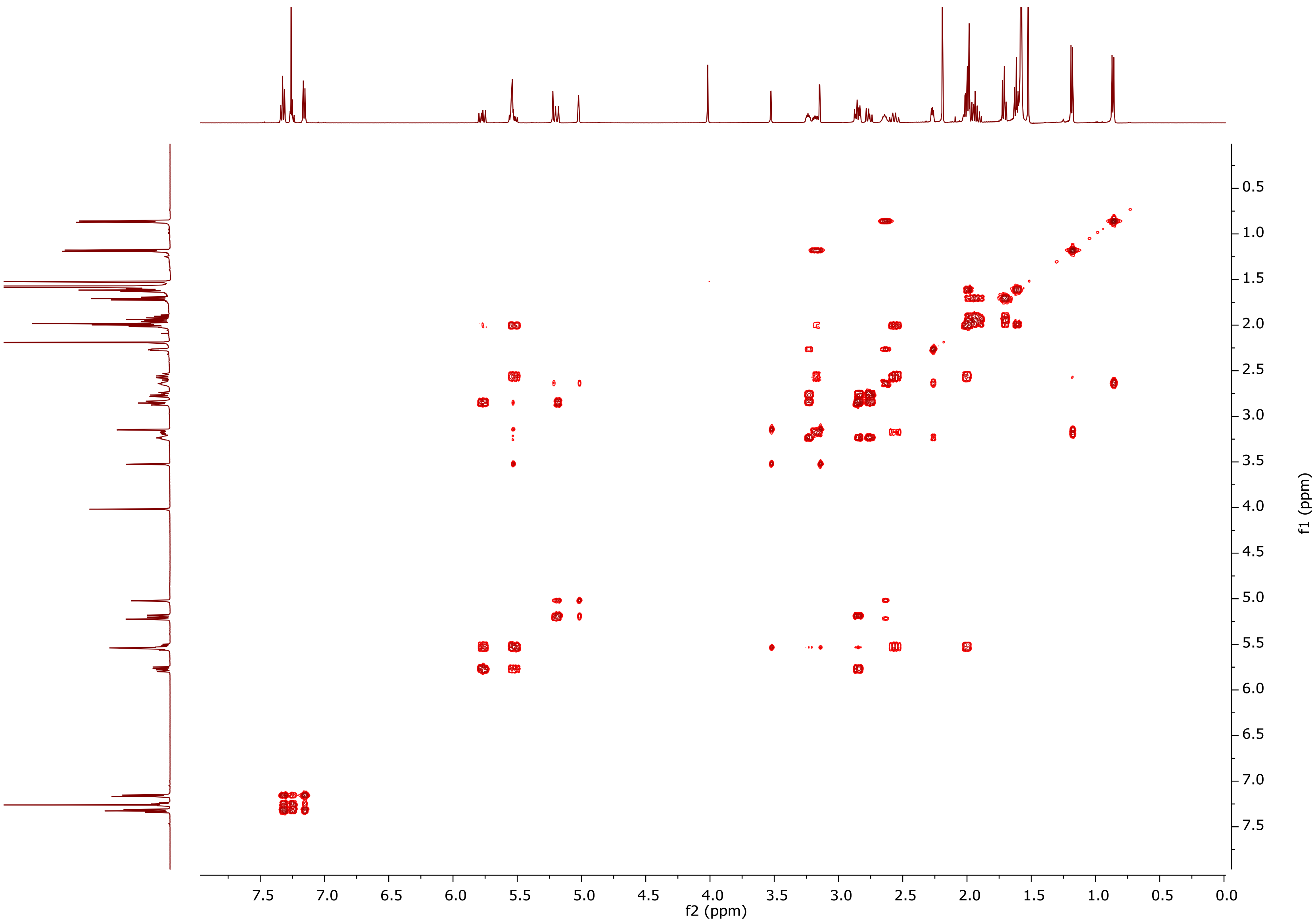

Figure SD179. ^{1}H-^{1}H COSY NMR spectrum of 7-diazirin-19,20-epoxycytochalasin D (**26**) (500/500 MHz, $CDCl_3$)

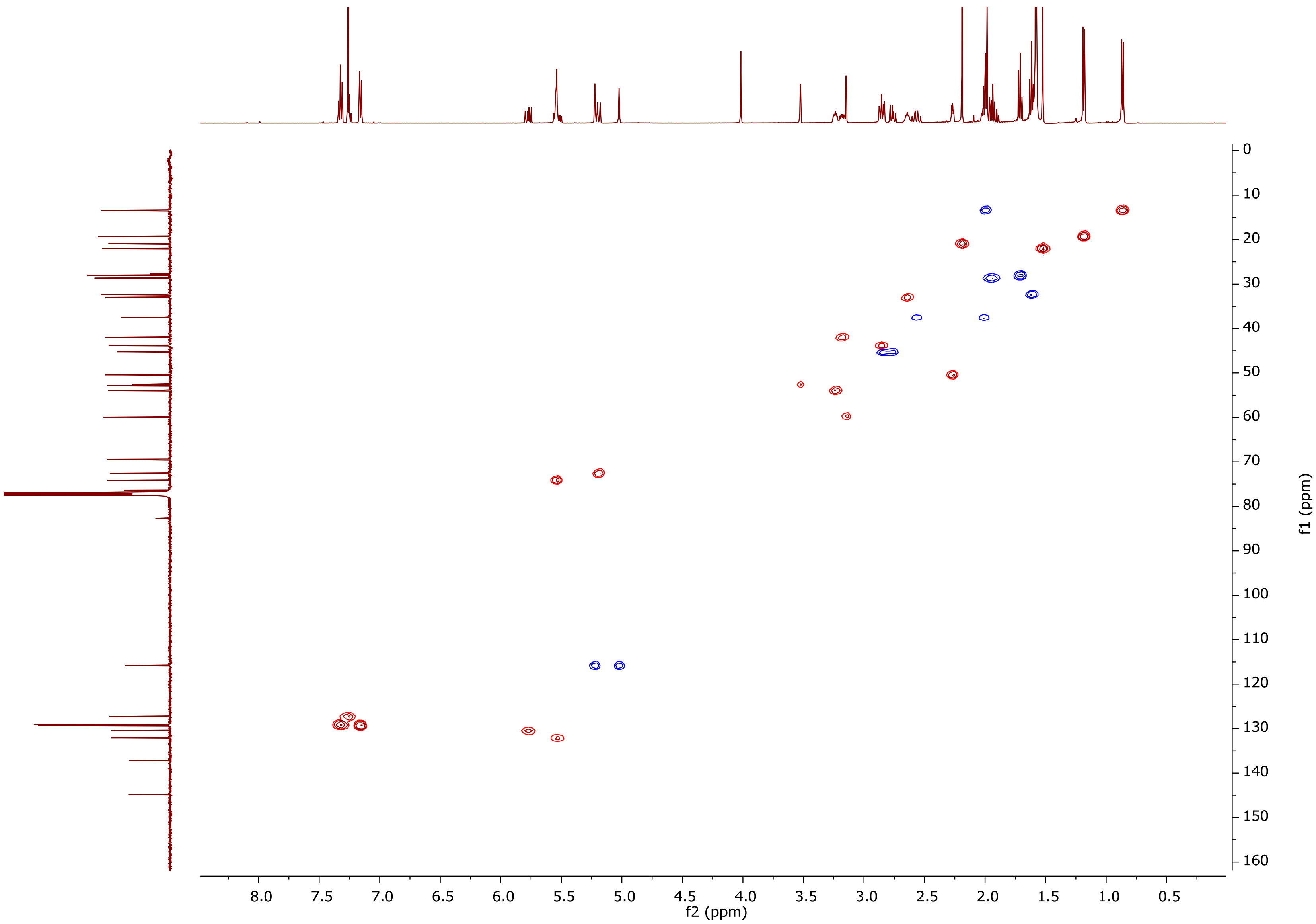

Figure SD180. ^{1}H-^{13}C HSQC NMR spectrum of 7-diazirin-19,20-epoxycytochalasin D (**26**) (500/125 MHz, $CDCl_3$)

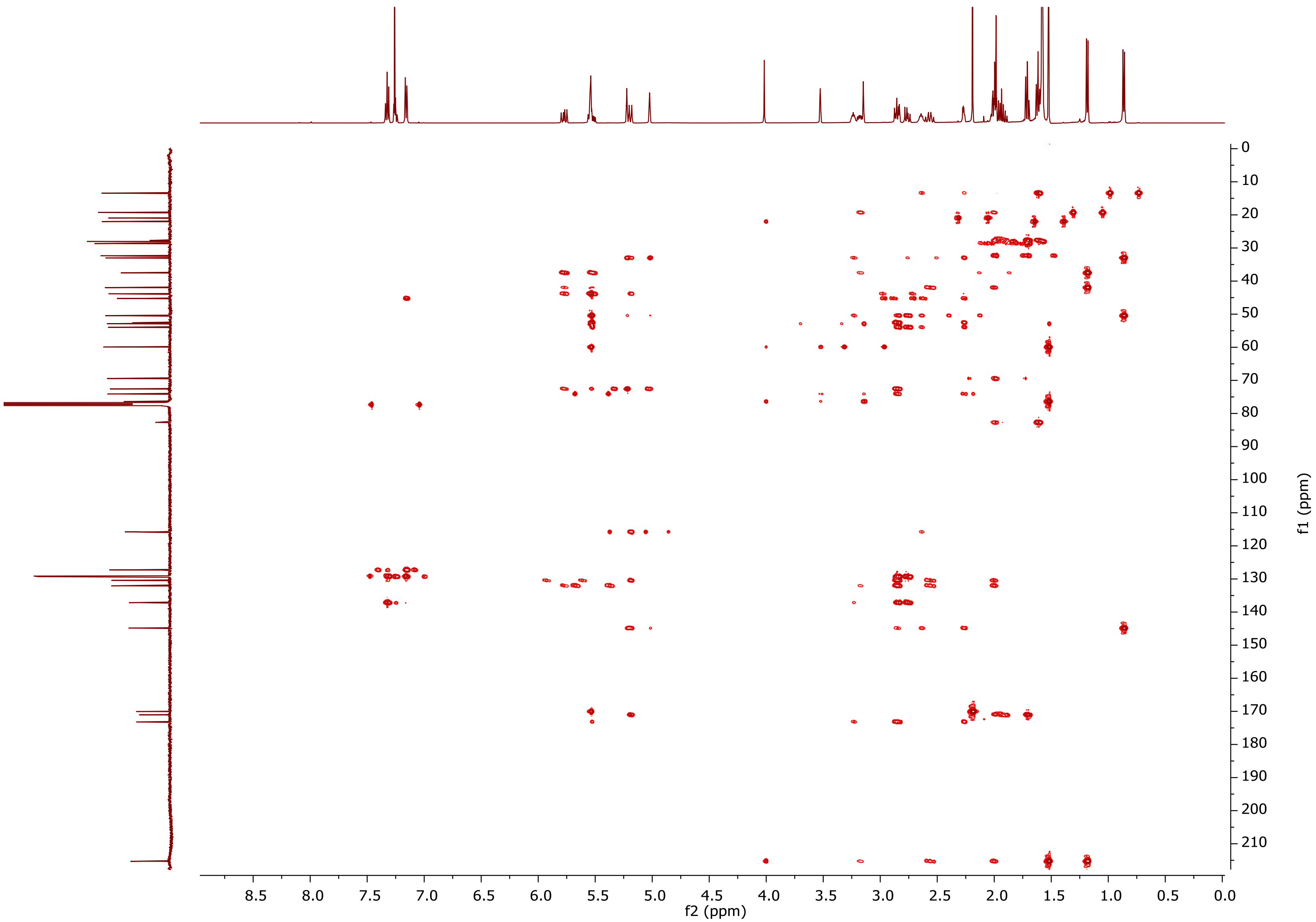

Figure SD181. ^{1}H-^{13}C HMBC NMR spectrum of 7-diazirin-19,20-epoxycytochalasin D (**26**) (500/125 MHz, $CDCl_3$)

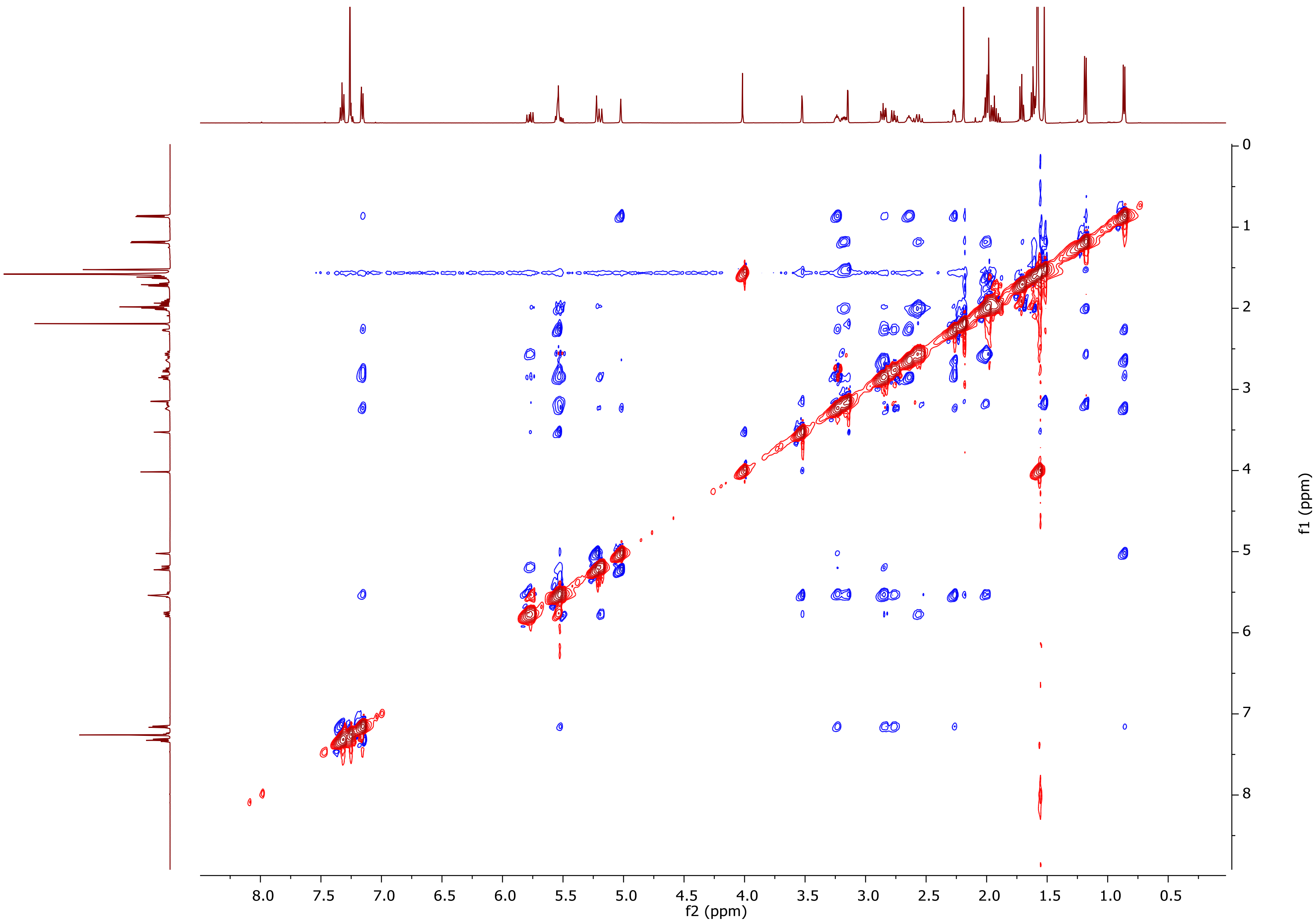

Figure SD182. ^1H-^1H NOESY NMR spectrum of 7-diazirin-19,20-epoxycytochalasin D (**26**) (500/500 MHz, $CDCl_3$)

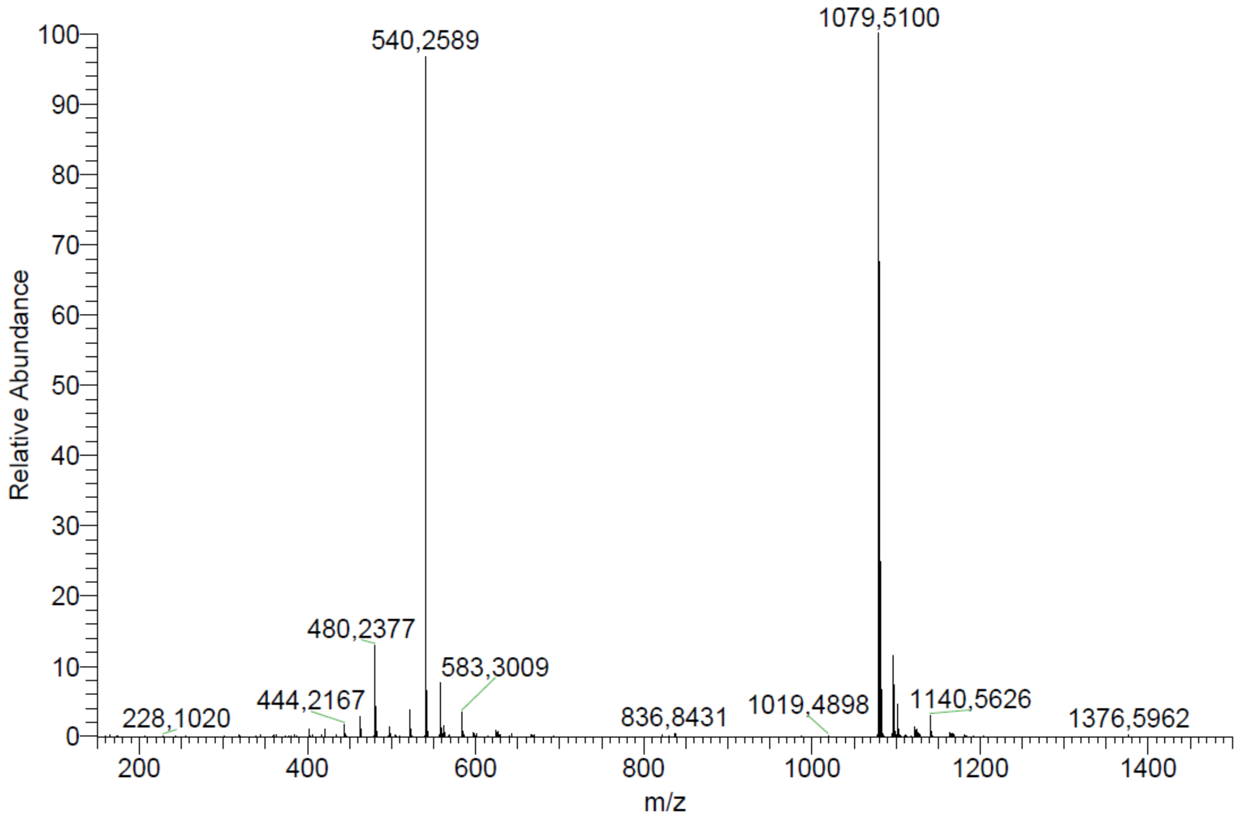

Figure SD183. ESI-HRMS spectrum of 19,20-epoxycytochalasin N (**27**)

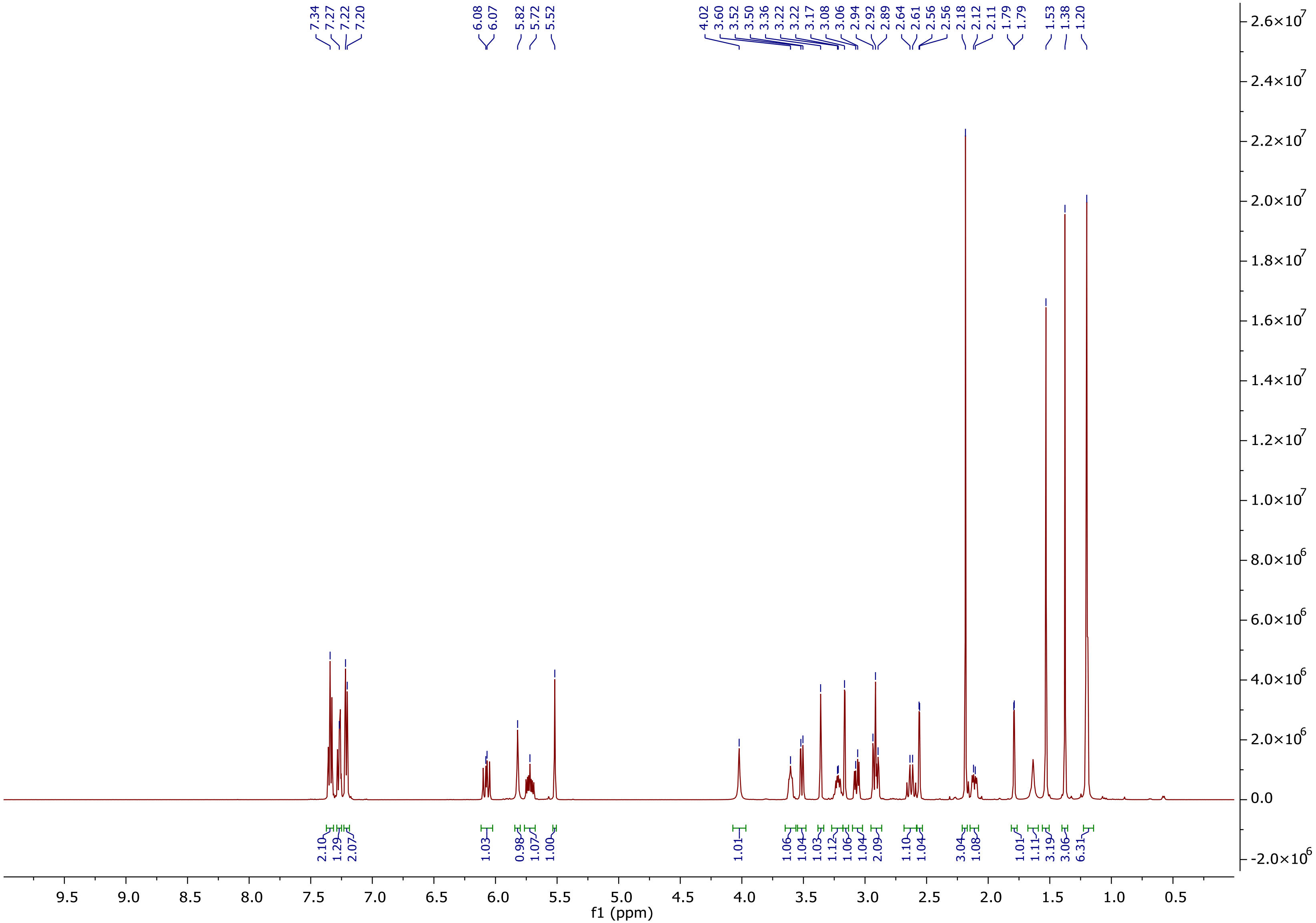

Figure SD184. ^{1}H NMR spectrum of 19,20-epoxycytochalasin N (**27**) (500 MHz, $CDCl_3$)

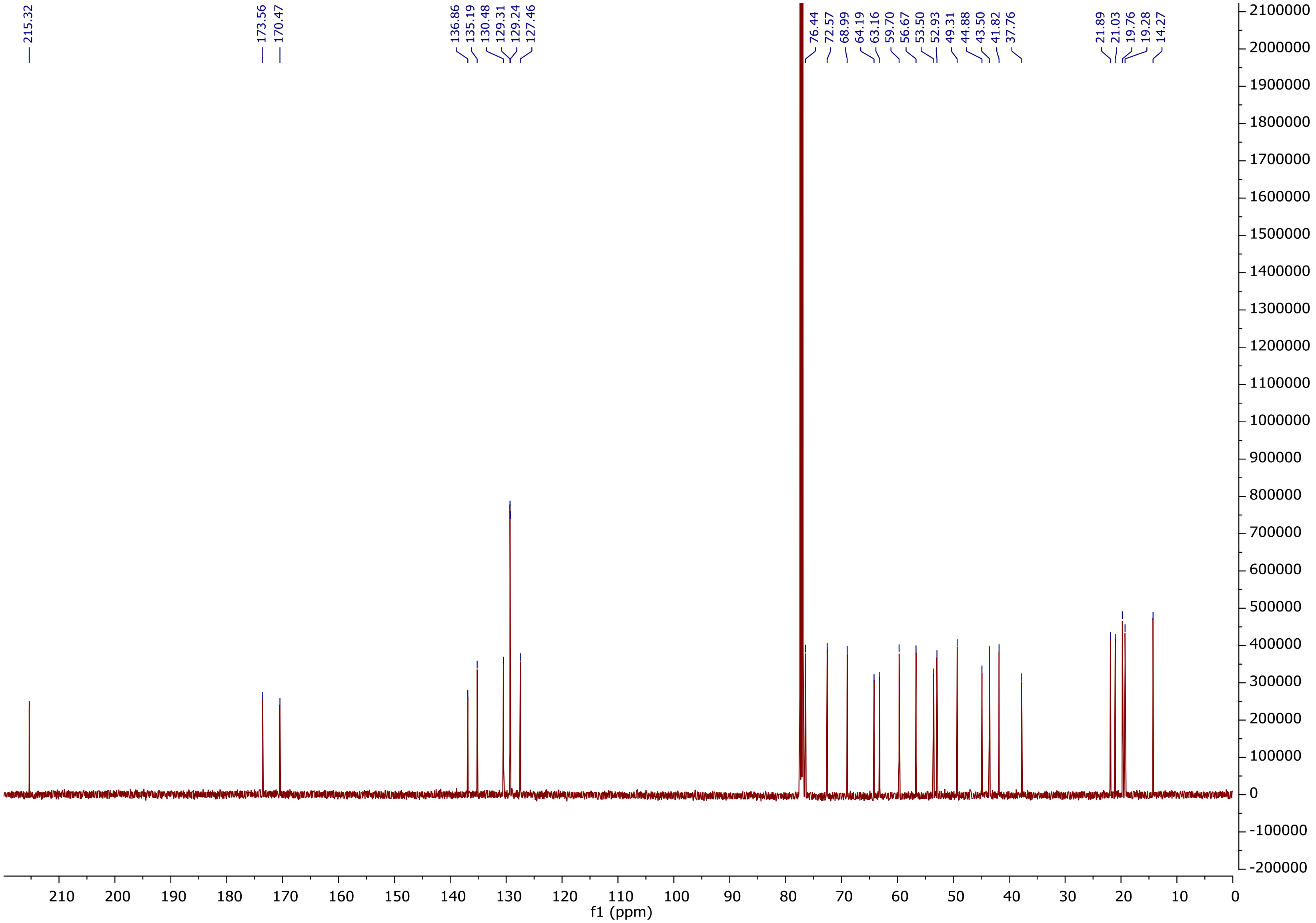

Figure SD185. ^{13}C NMR spectrum of 19,20-epoxycytochalasin N (**27**) (125 MHz, $CDCl_3$)

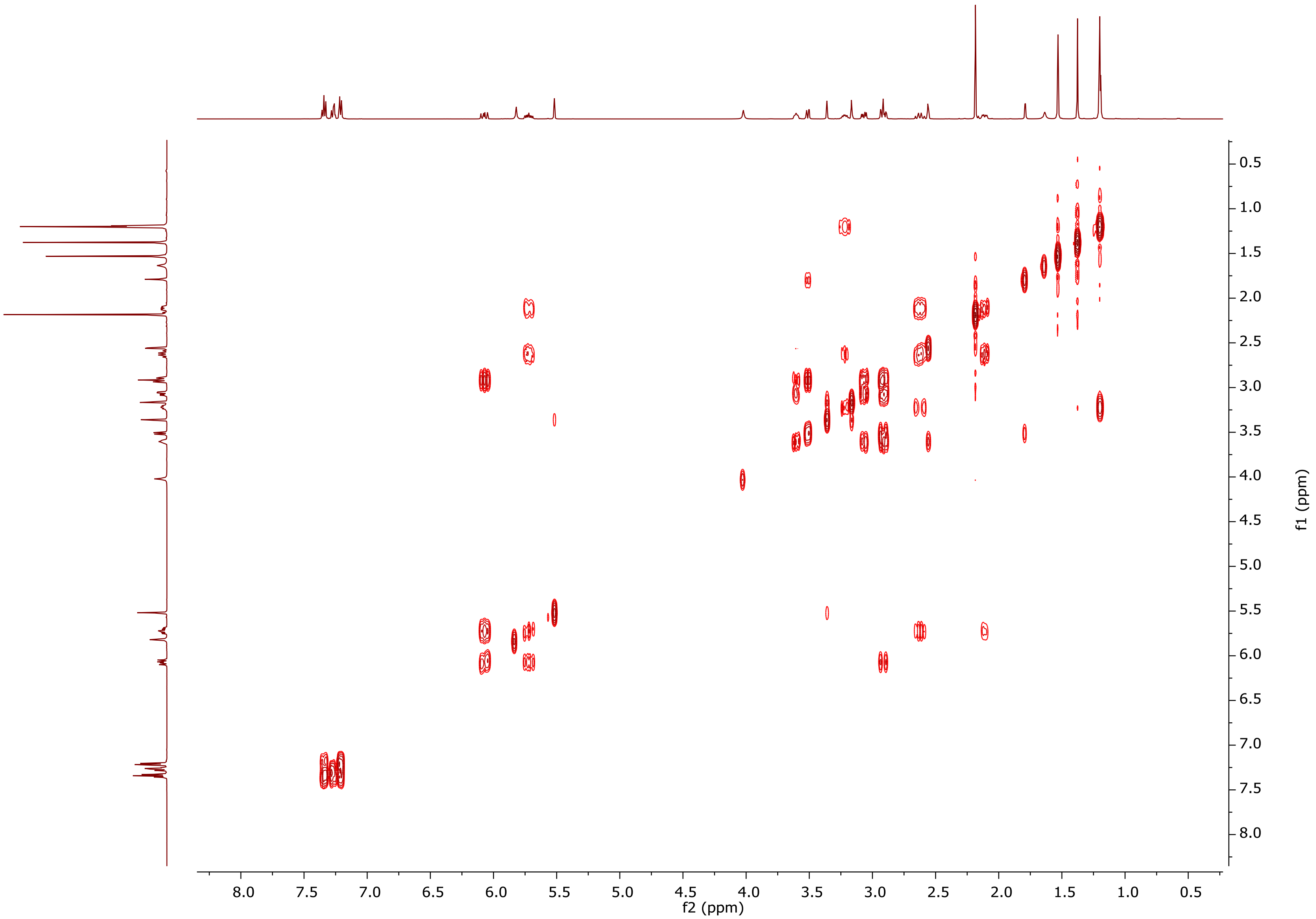

Figure SD186. ^{1}H-^{1}H COSY NMR spectrum of 19,20-epoxycytochalasin N (**27**) (500/500 MHz, $CDCl_3$)

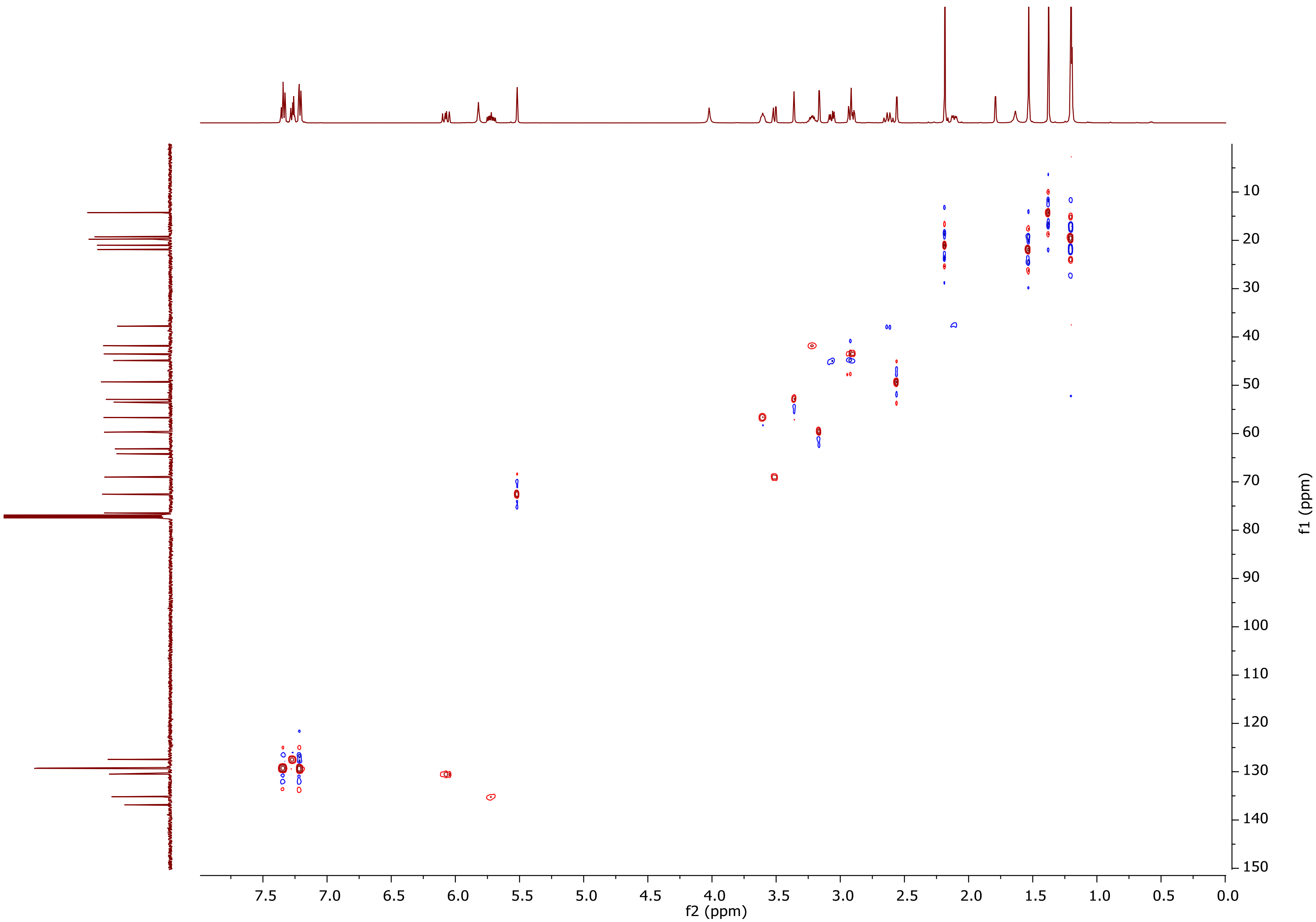

Figure SD187. ^{1}H-^{13}C HSQC NMR spectrum of 19,20-epoxycytochalasin N (**27**) (500/125 MHz, $CDCl_3$)

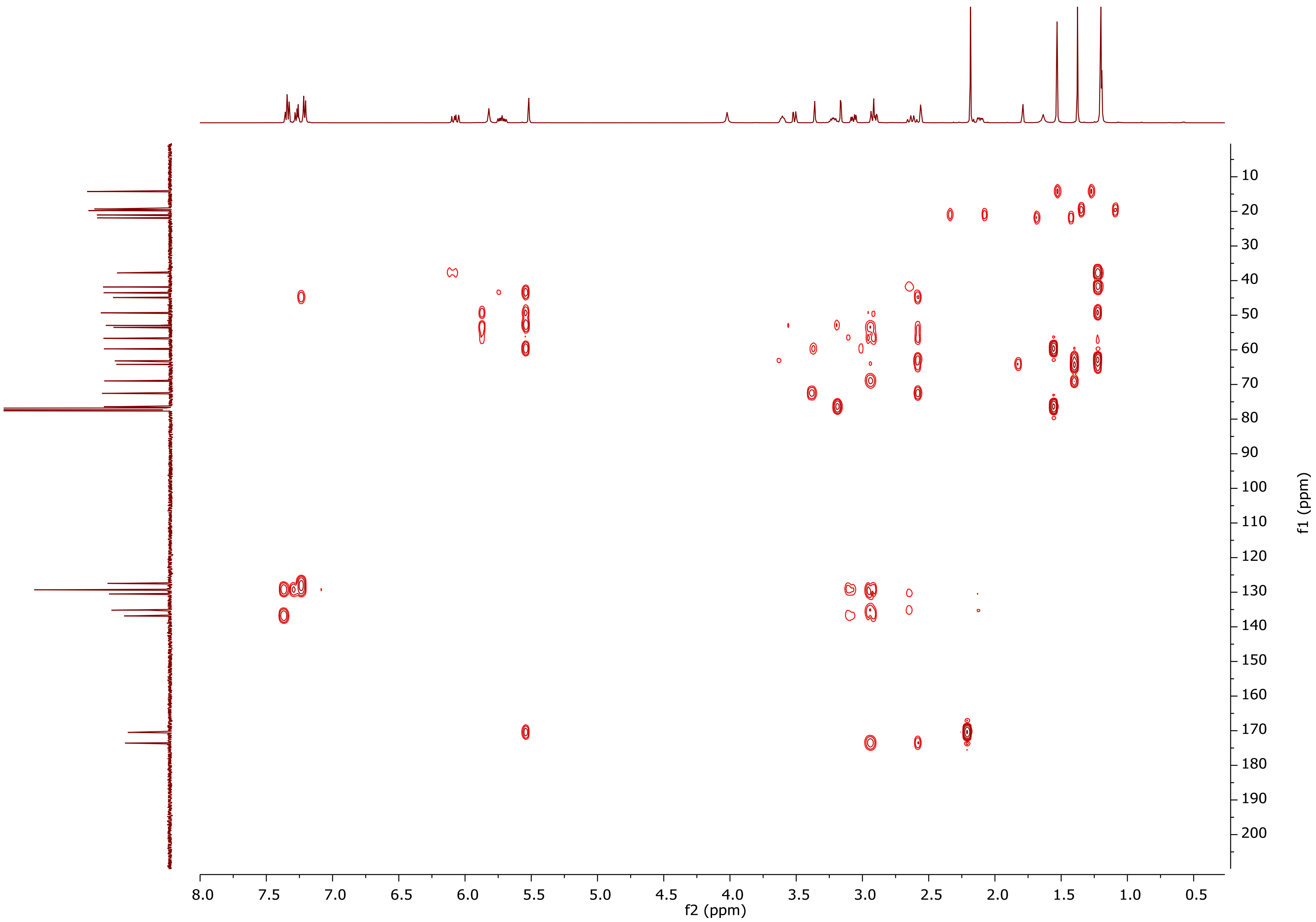

Figure SD188. ^{1}H-^{13}C HMBC NMR spectrum of 19,20-epoxycytochalasin N (**27**) (500/125 MHz, $CDCl_3$)

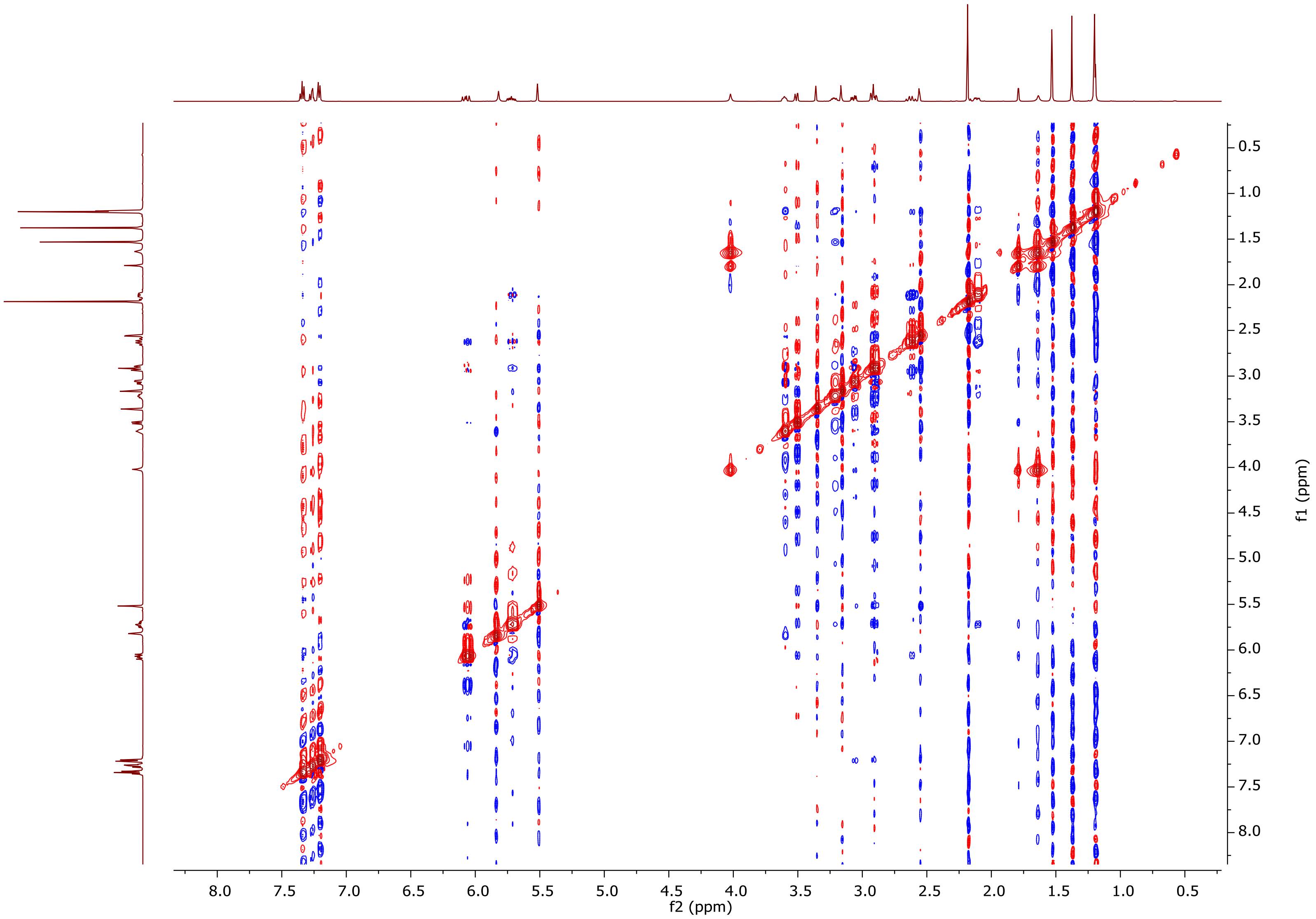

Figure SD189. ^{1}H-^{1}H NOESY NMR spectrum of 19,20-epoxycytochalasin N (**27**) (500/500 MHz, $CDCl_3$)

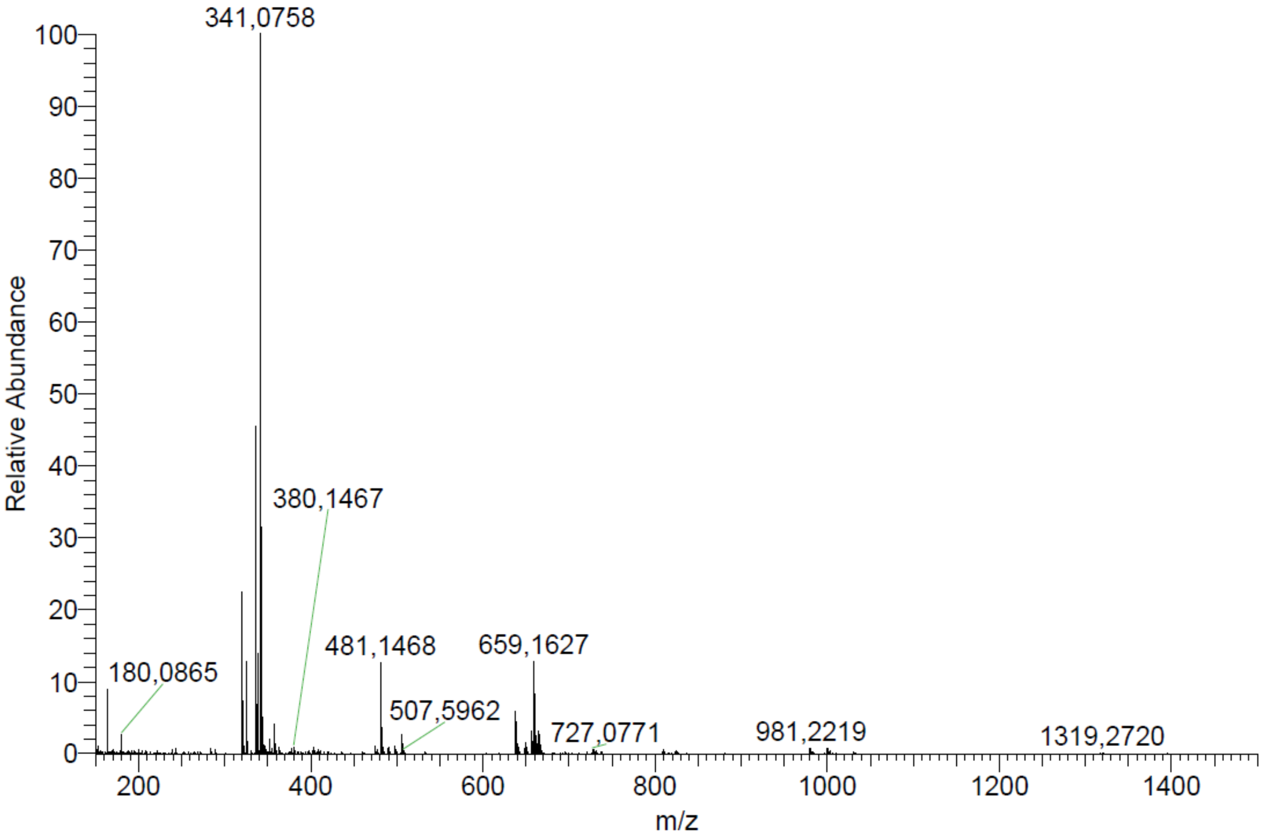

Figure SD190. ESI-HRMS spectrum of *m*-chloro-phenylethyl-*α*-D-glucopyranoside (**28**)

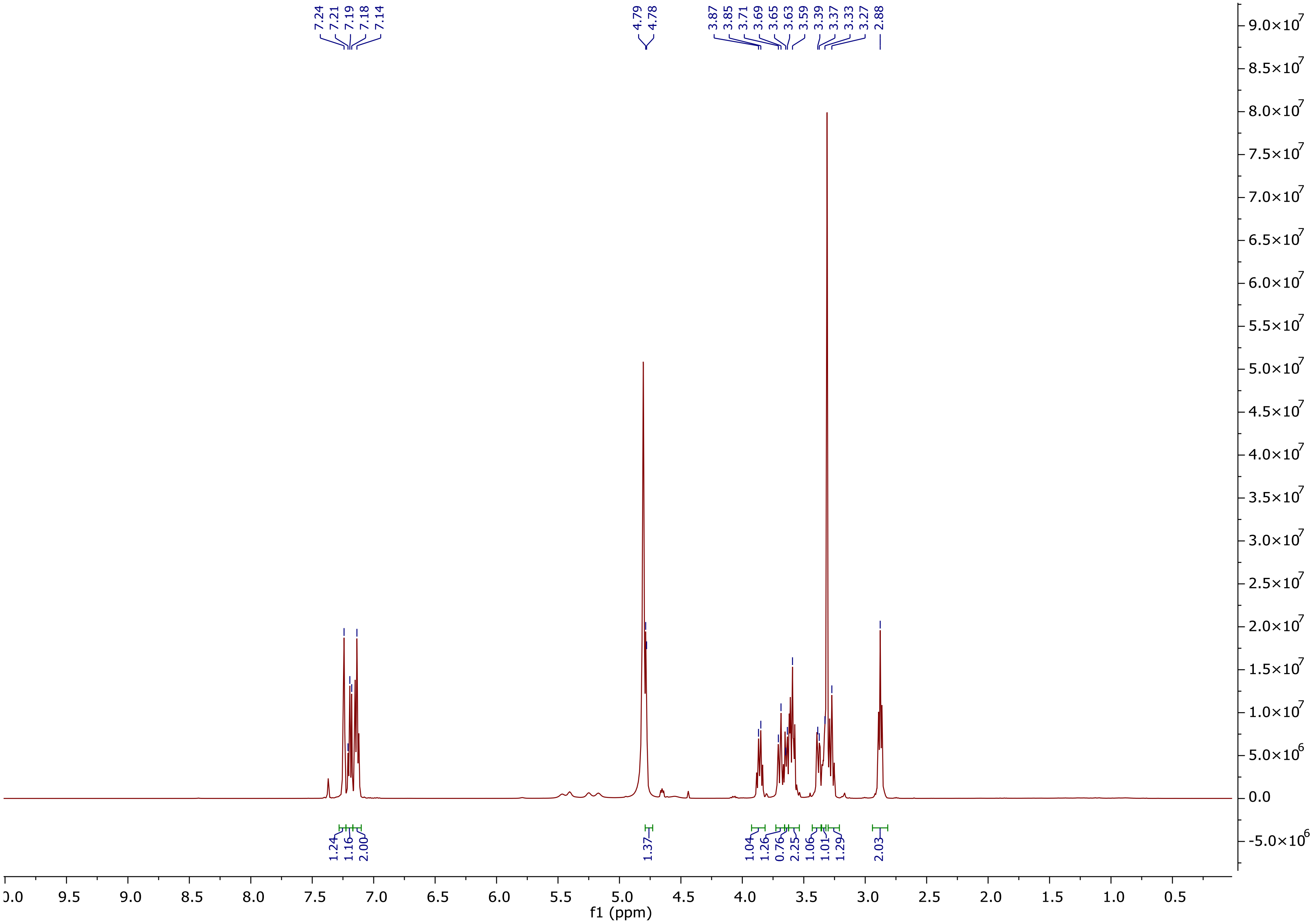

Figure SD191. ^{1}H NMR spectrum of *m*-chloro-phenylethyl-*α*-D-glucopyranoside (**28**) (500 MHz, MeOH-*d*$_4$)

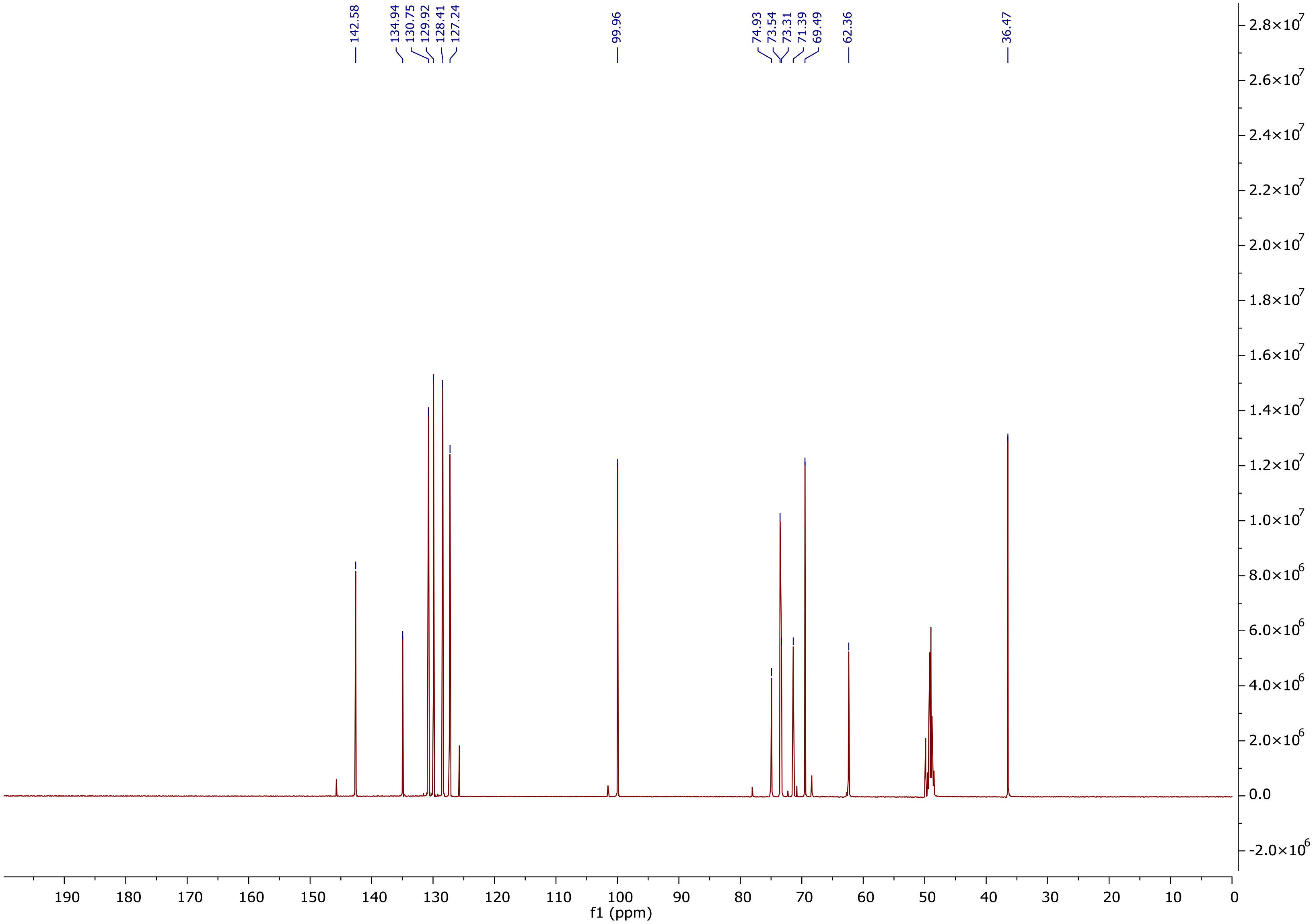

Figure SD192. ^{13}C NMR spectrum of *m*-chloro-phenylethyl-*α*-D-glucopyranoside (**28**) (125 MHz, MeOH-d_4)

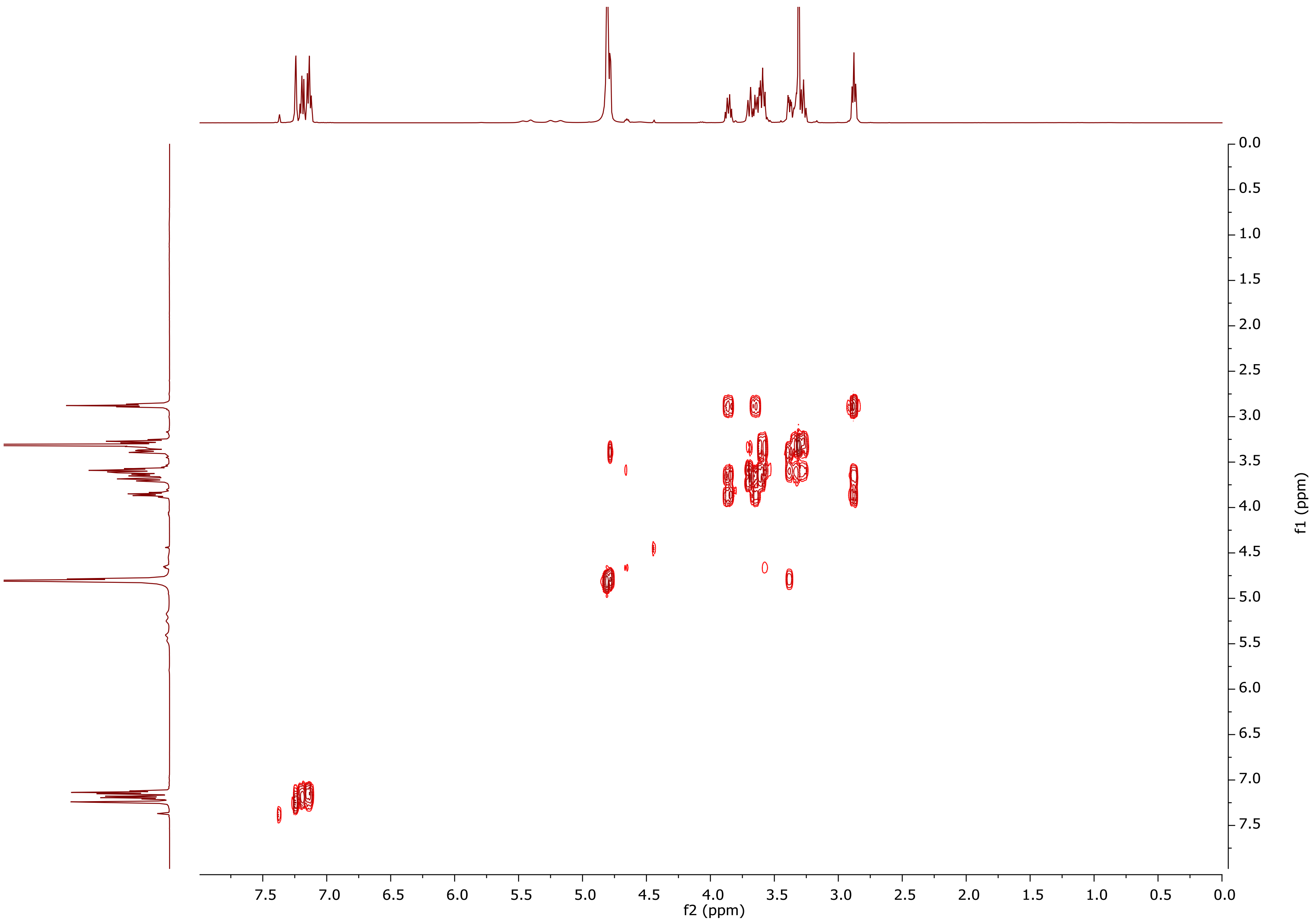

Figure SD193. ^{1}H-^{1}H COSY NMR spectrum of *m*-chloro-phenylethyl-*α*-D-glucopyranoside (**28**) (500/500 MHz, MeOH-d_4)

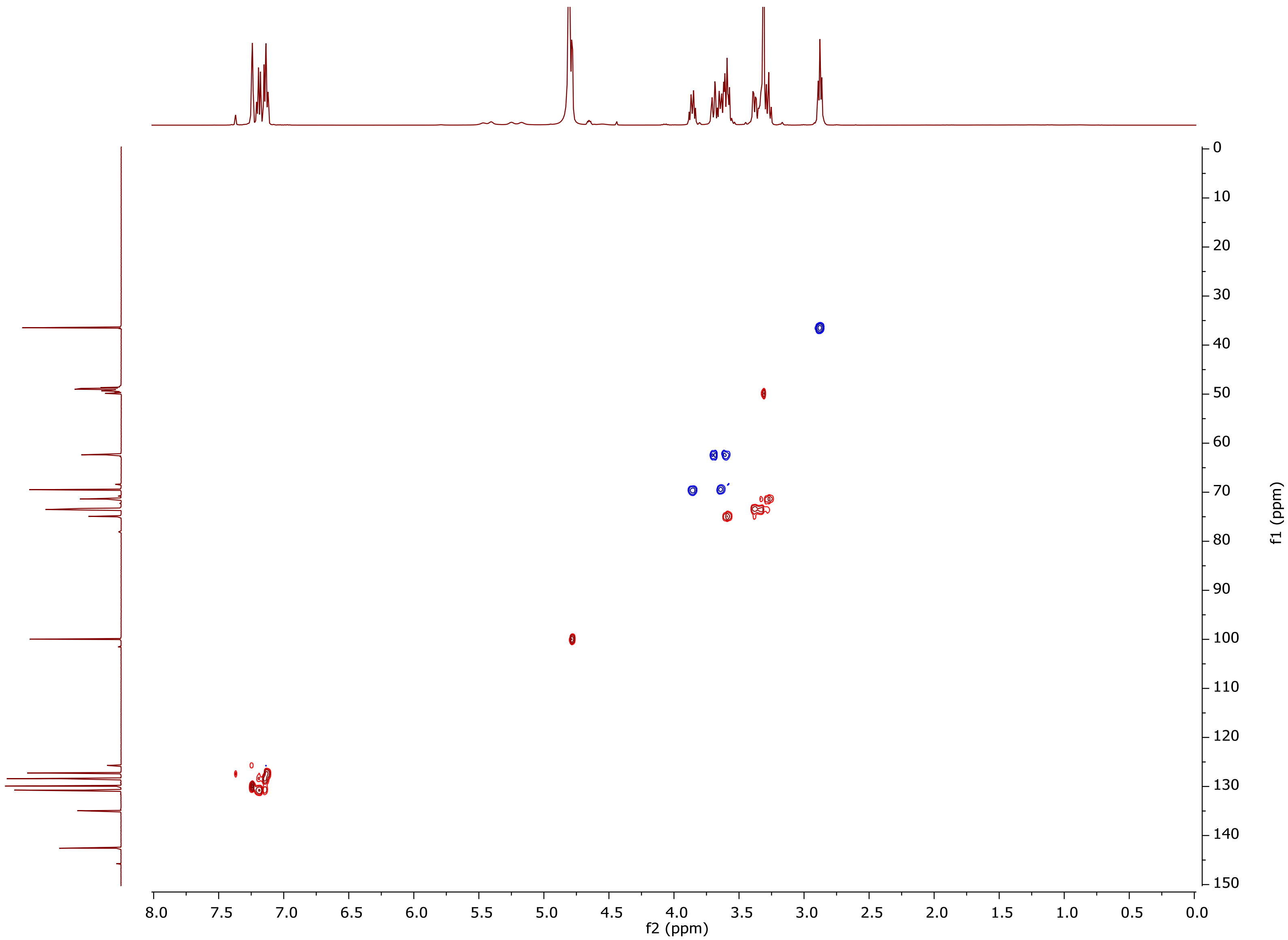

Figure SD194. ^1H-^{13}C HSQC NMR spectrum of *m*-chloro-phenylethyl-*α*-D-glucopyranoside (**28**) (500/125 MHz, MeOH-d_4)

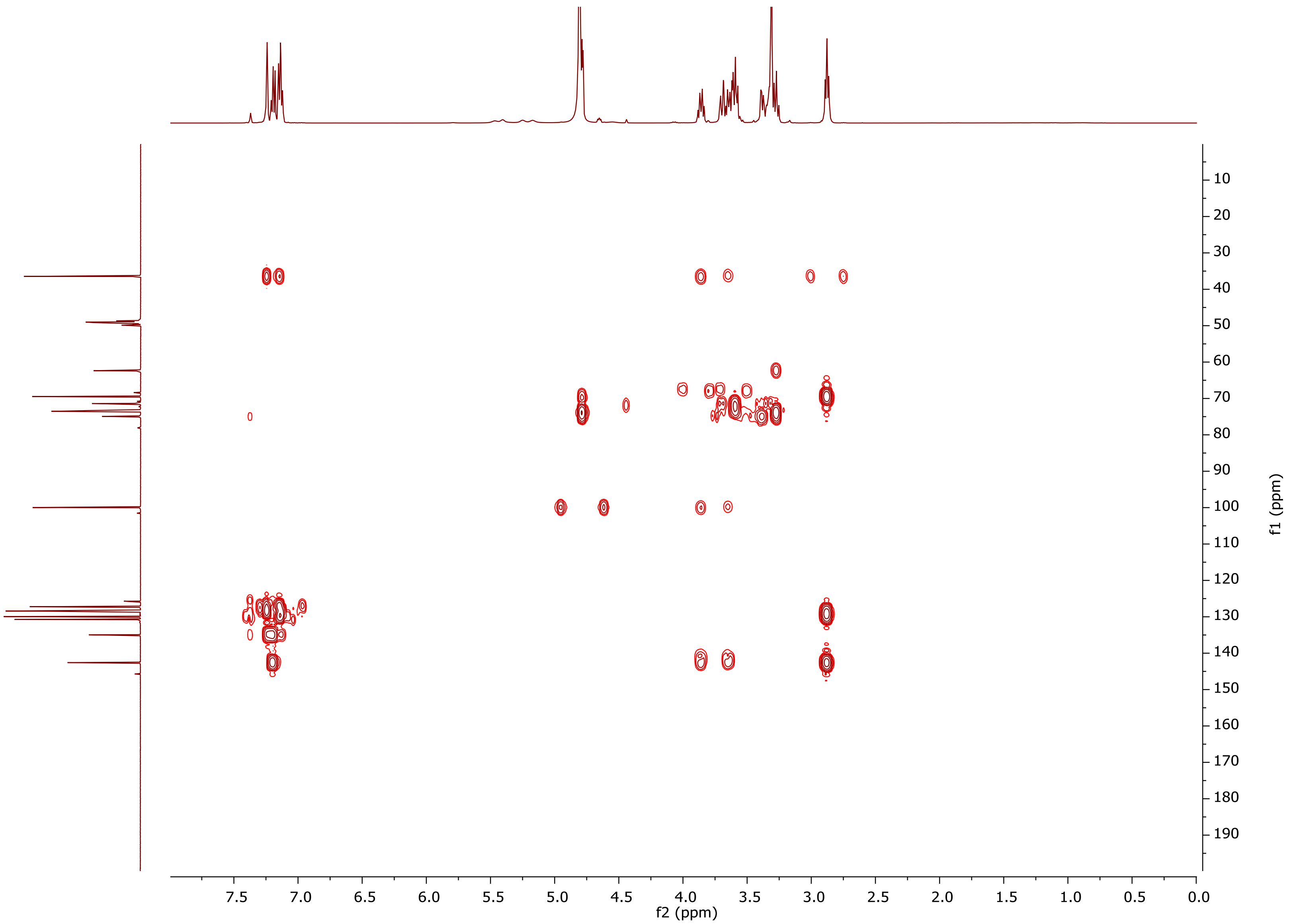

Figure SD195. ^{1}H-^{13}C HMBC NMR spectrum of *m*-chloro-phenylethyl-*α*-D-glucopyranoside (**28**) (500/125 MHz, MeOH-d_4)

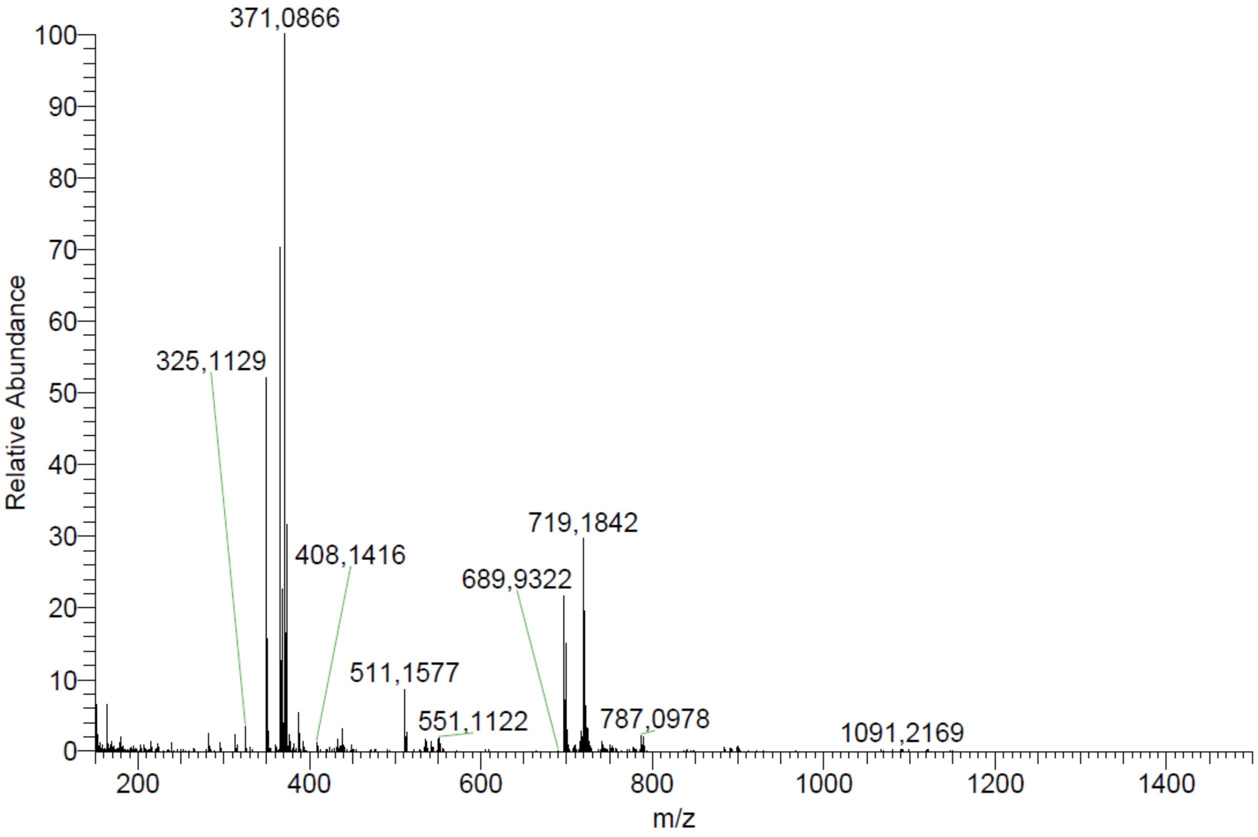

Figure SD196. ESI-HRMS spectrum of *m*-chloro-2-hydroxy-3-phenylpropyl-*α*-D-glucopyranoside (**29**)

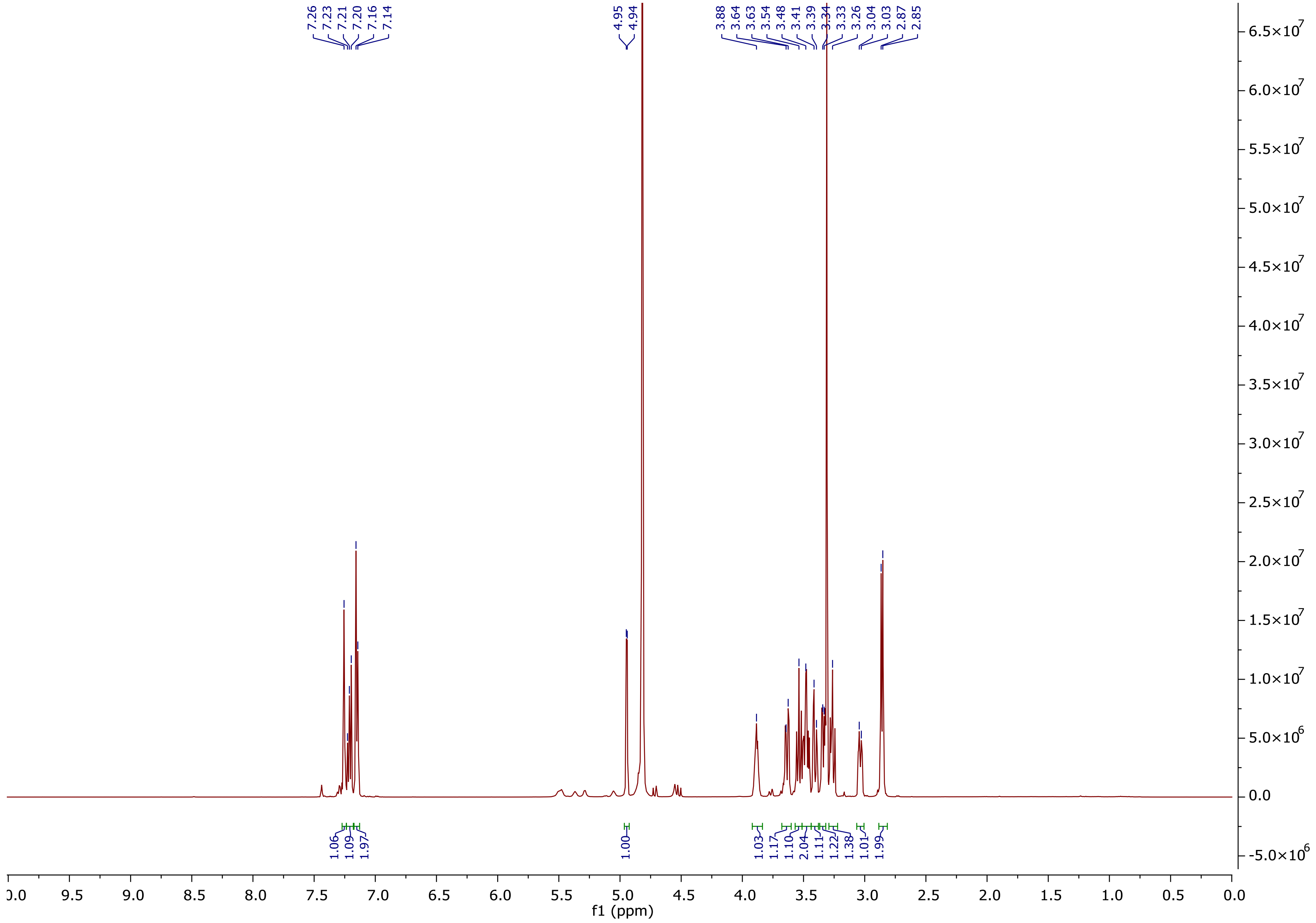

Figure SD197. ^{1}H NMR spectrum of *m*-chloro-2-hydroxy-3-phenylpropyl-*α*-D-glucopyranoside (**29**) (500 MHz, MeOH-d_4)

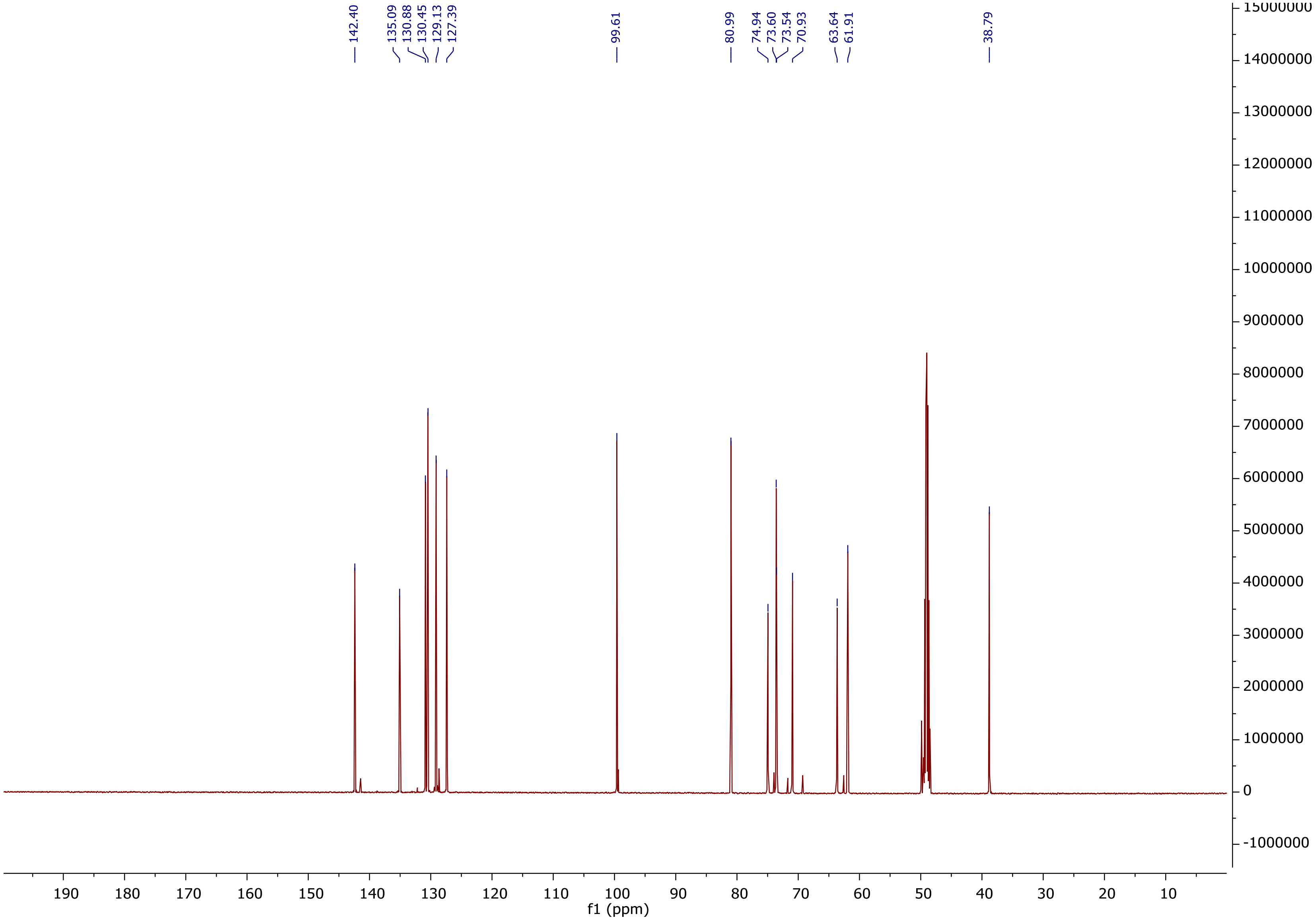

Figure SD198. ^{13}C NMR spectrum of *m*-chloro-2-hydroxy-3-phenylpropyl-*α*-D-glucopyranoside (**29**) (125 MHz, MeOH-d_4)

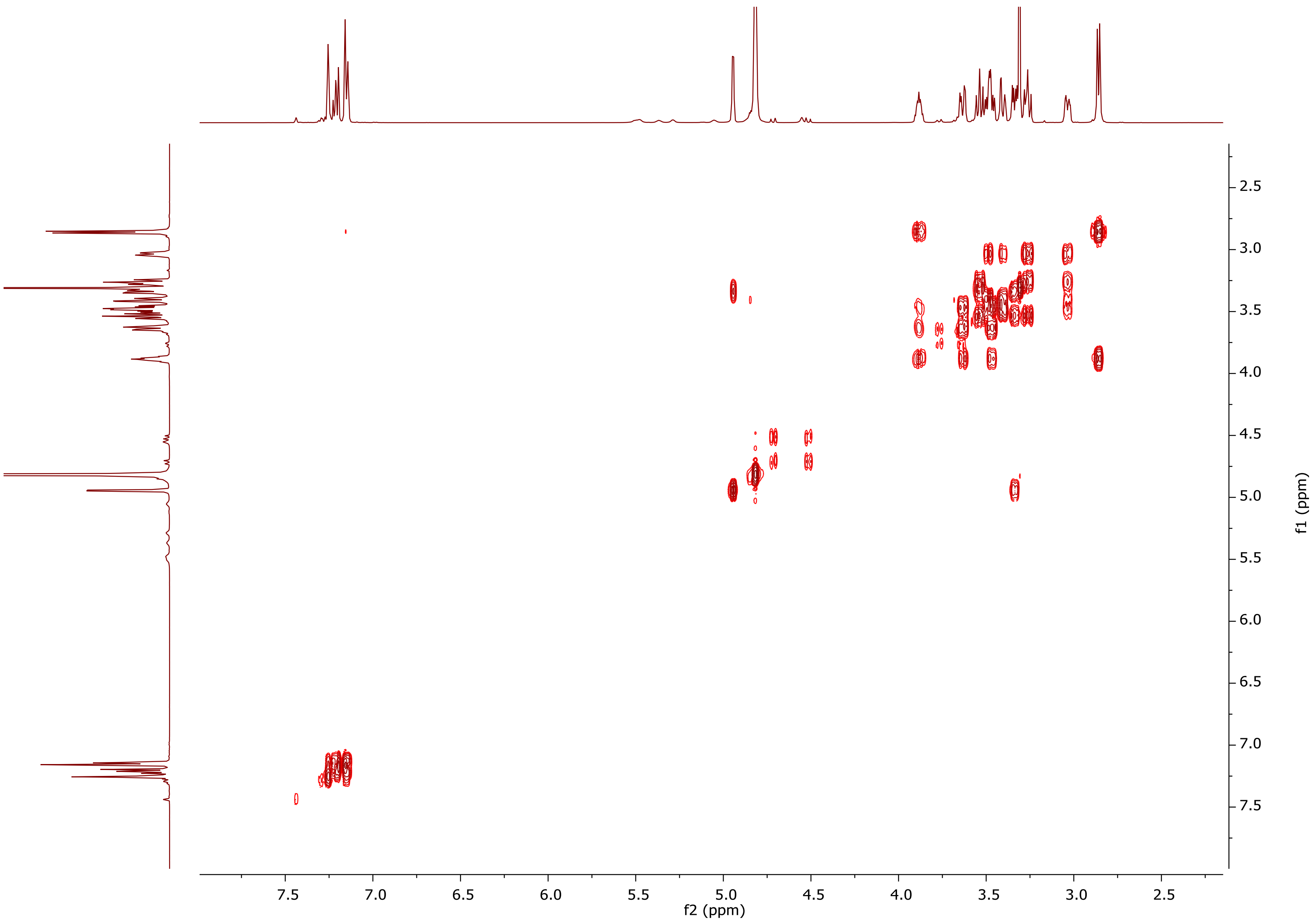

Figure SD199. ^1H-^1H COSY NMR spectrum of *m*-chloro-2-hydroxy-3-phenylpropyl-*α*-D-glucopyranoside (**29**) (500/500 MHz, MeOH-*d₄*)

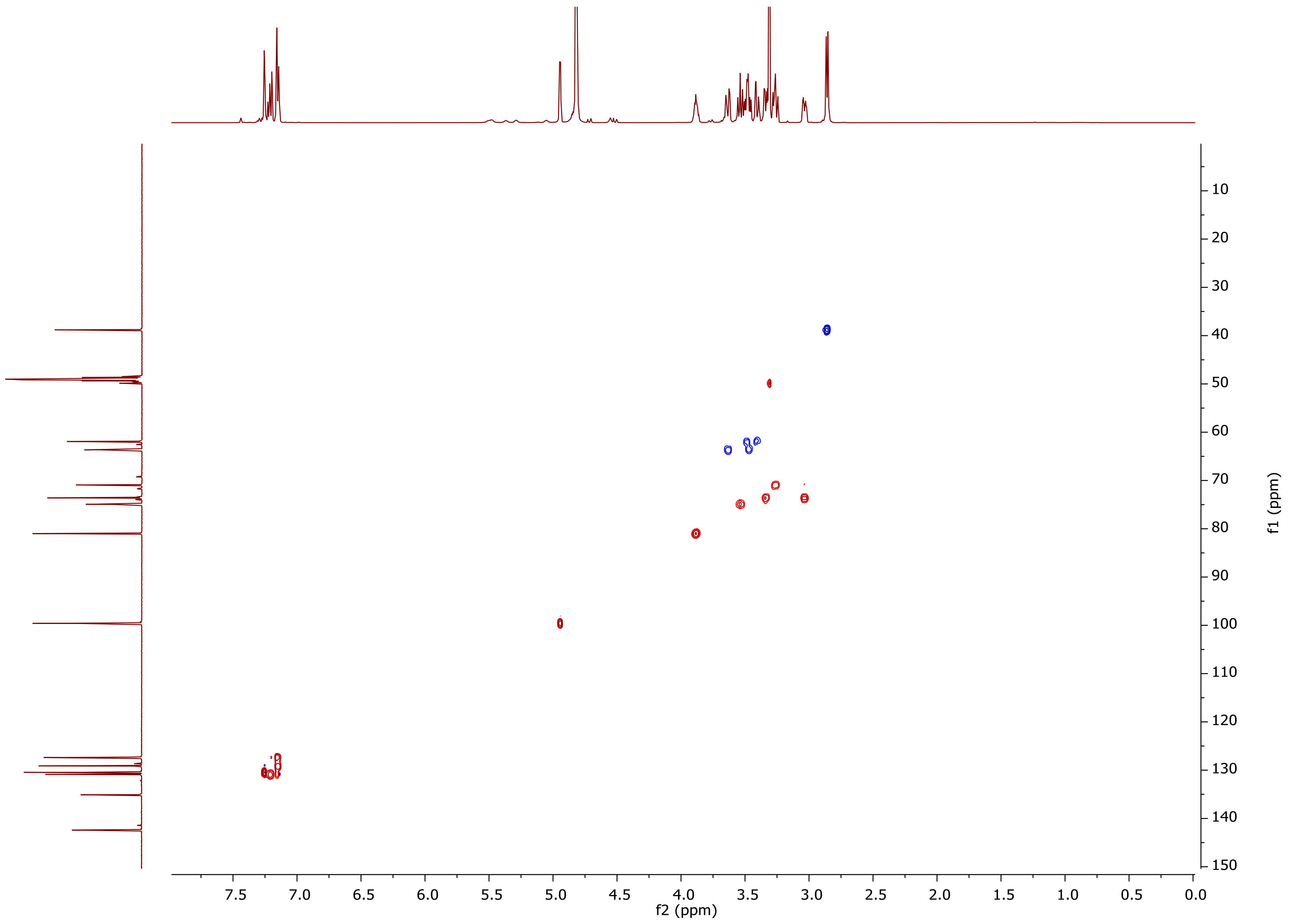

Figure SD200. ^{1}H-^{13}C HSQC NMR spectrum of *m*-chloro-2-hydroxy-3-phenylpropyl-*α*-D-glucopyranoside (**29**) (500/125 MHz, MeOH-*d*$_4$)

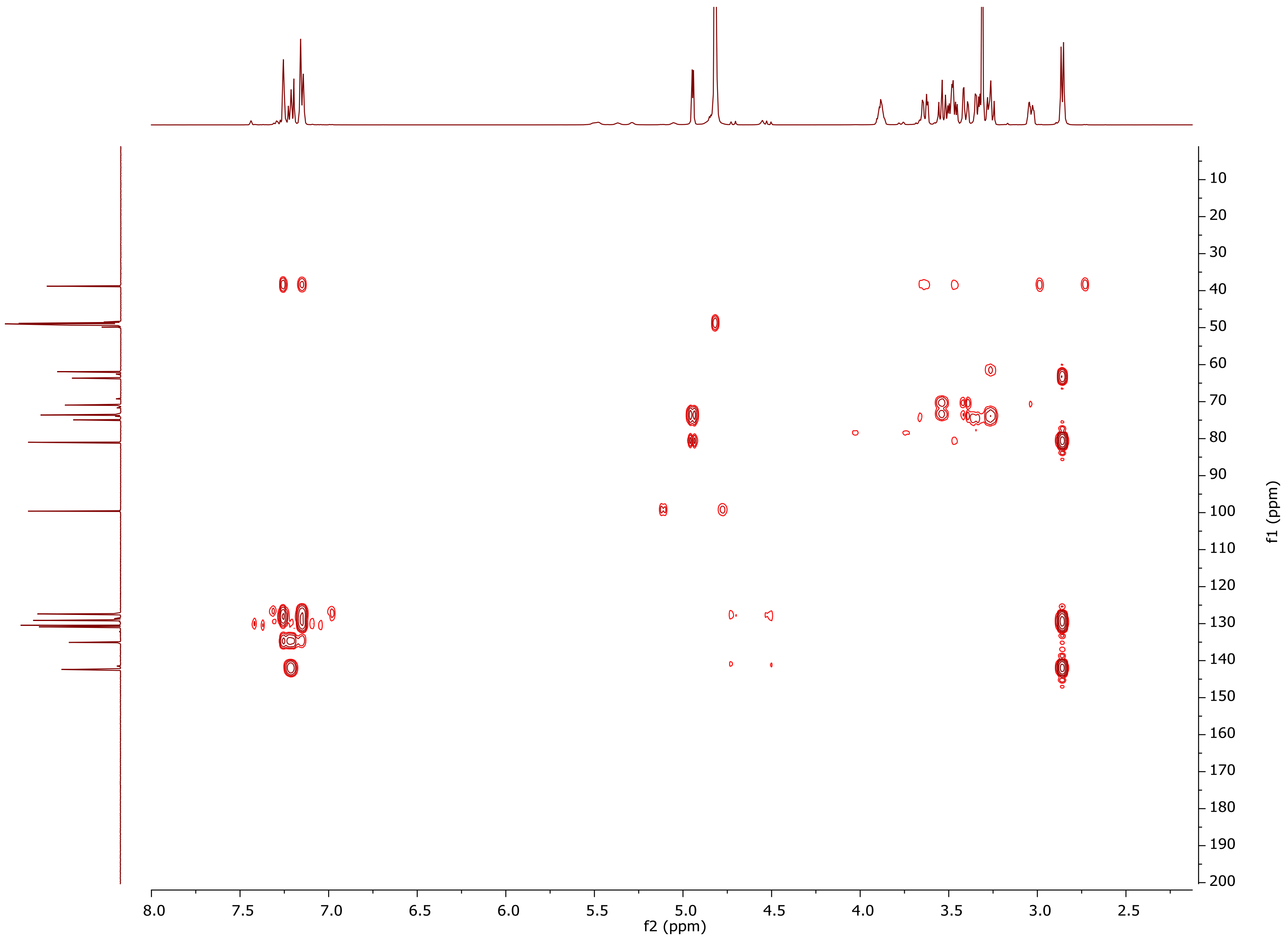

Figure SD201. ^{1}H-^{13}C HMBC NMR spectrum of *m*-chloro-2-hydroxy-3-phenylpropyl-*α*-D-glucopyranoside (**29**) (500/125 MHz, MeOH-d_4)

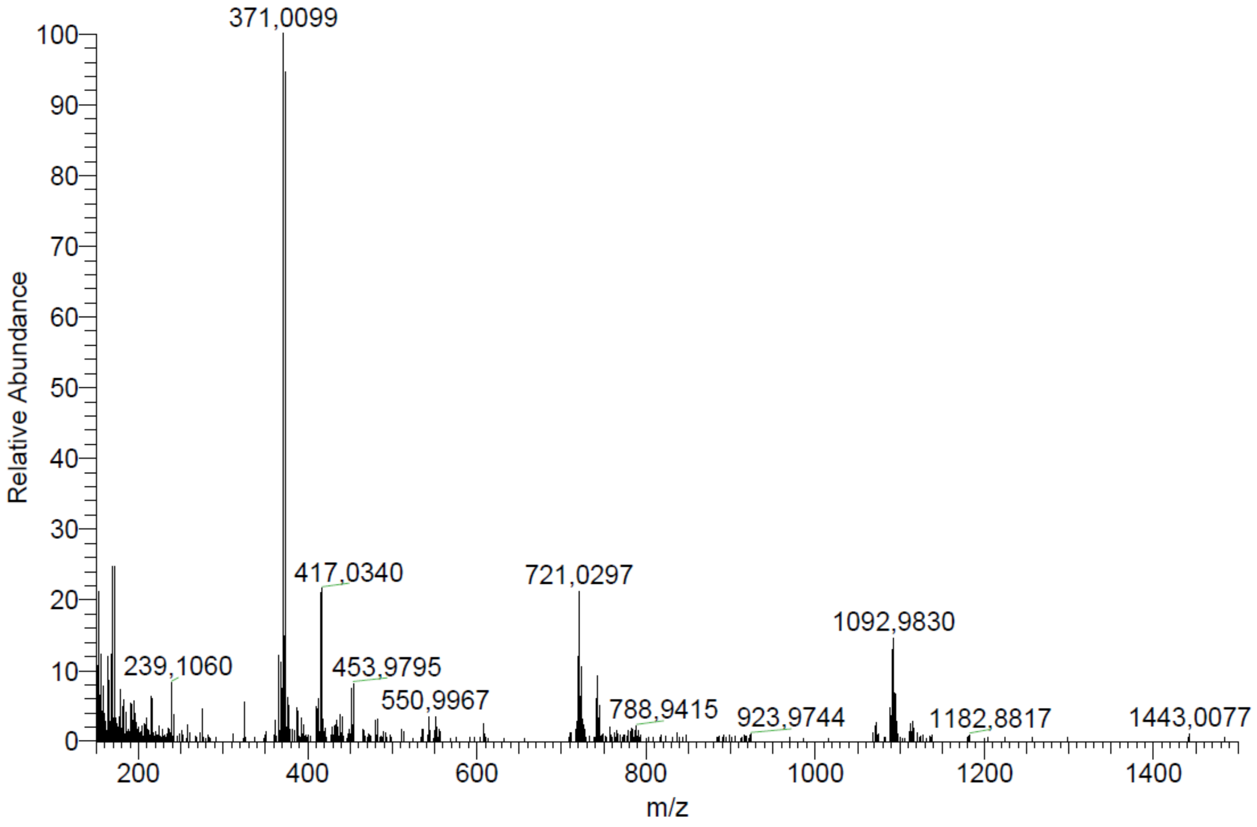

Figure SD202. ESI-HRMS spectrum of *m*-bromo-benzyl-*α*-D-glucopyranoside (**30**)

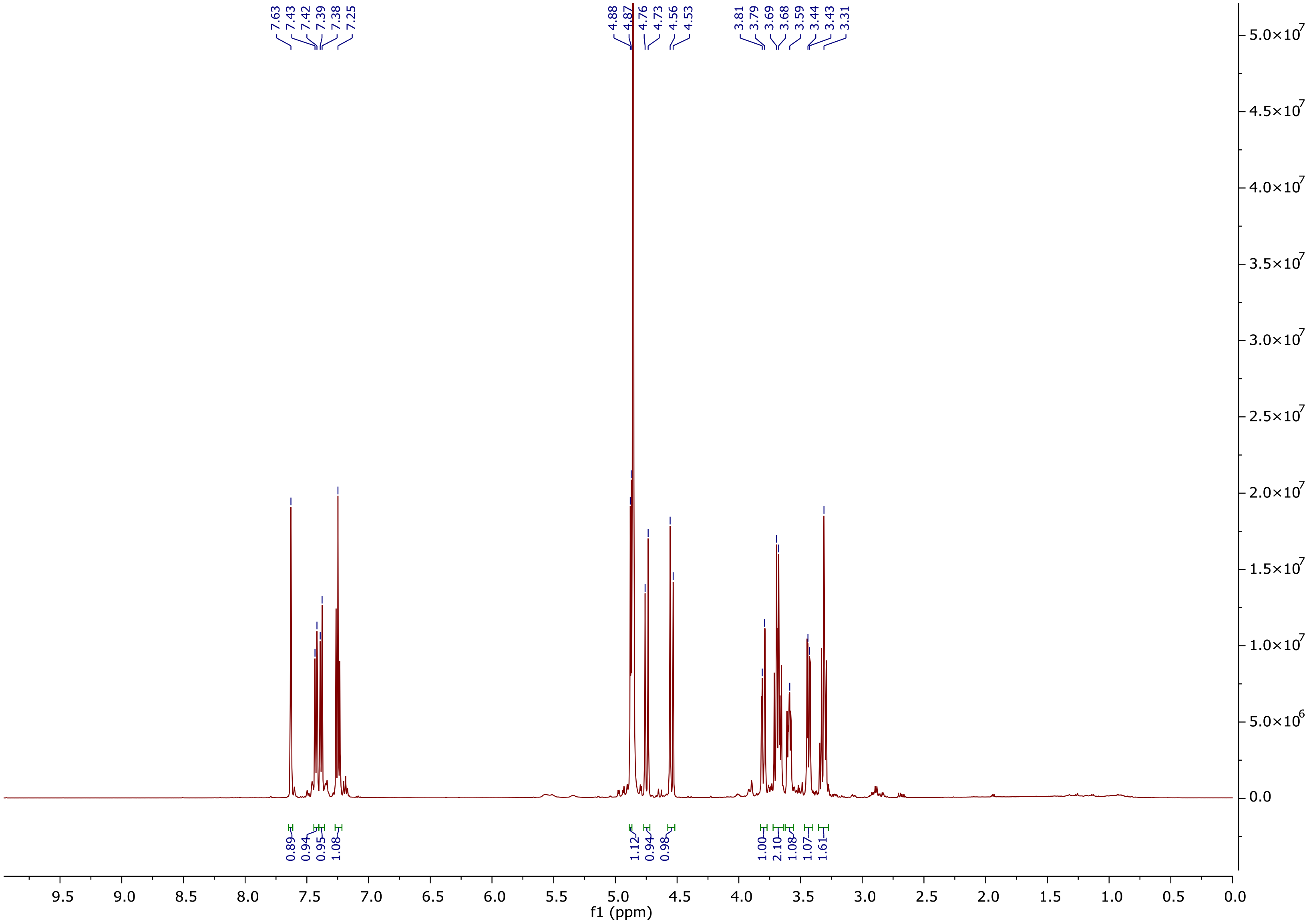

Figure SD203. ^{1}H NMR spectrum of *m*-bromo-benzyl-*α*-D-glucopyranoside (**30**) (500 MHz, MeOH-*d*$_4$)

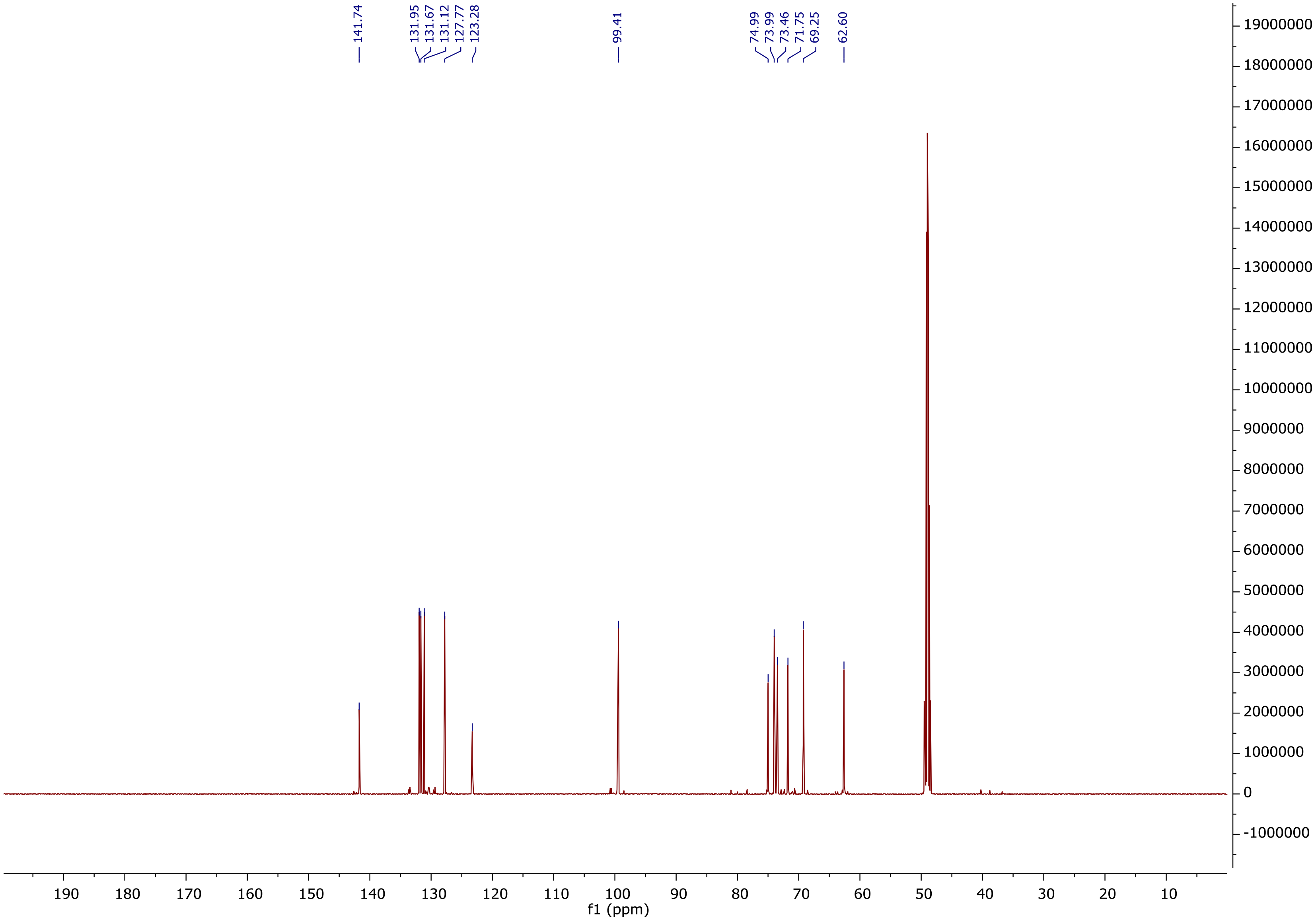

Figure SD204. ^{13}C NMR spectrum of *m*-bromo-benzyl-α-D-glucopyranoside (**30**) (125 MHz, MeOH-d_4)

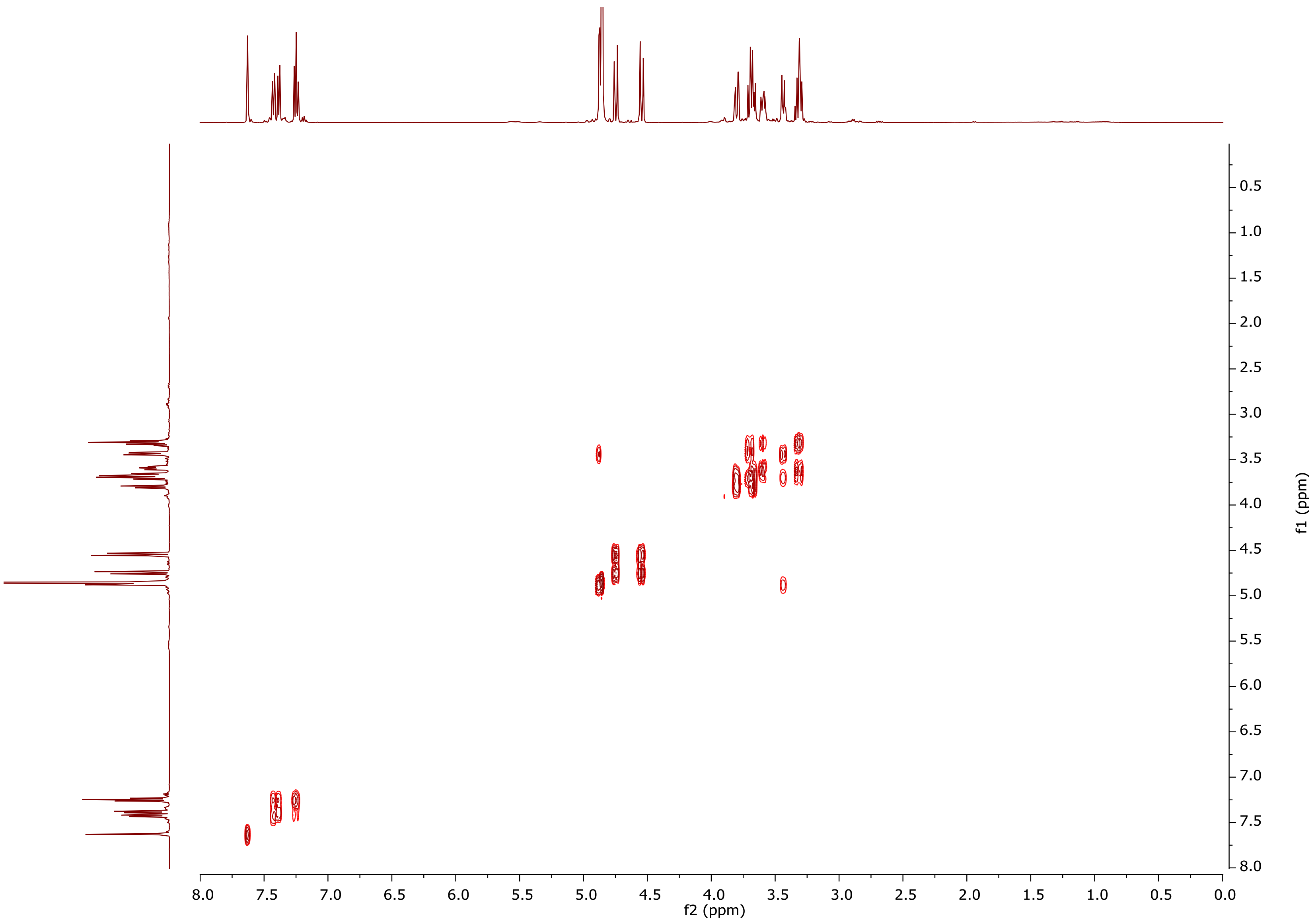

Figure SD205. ^{1}H-^{1}H COSY NMR spectrum of *m*-bromo-benzyl-*α*-D-glucopyranoside (**30**) (500/500 MHz, MeOH-d_4)

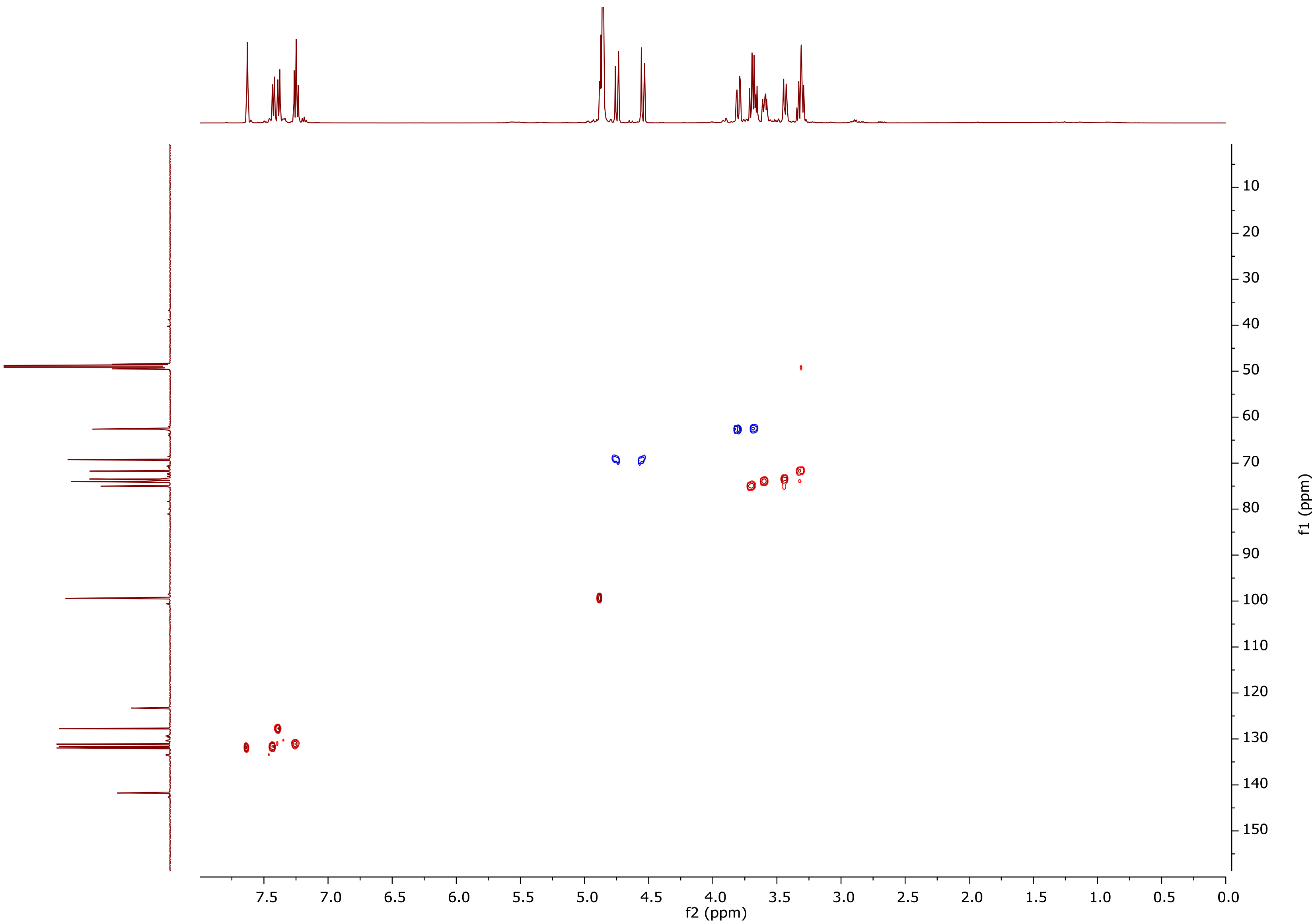

Figure SD206. ^1H-^{13}C HSQC NMR spectrum of *m*-bromo-benzyl-*α*-D-glucopyranoside (**30**) (500/125 MHz, MeOH-d_4)

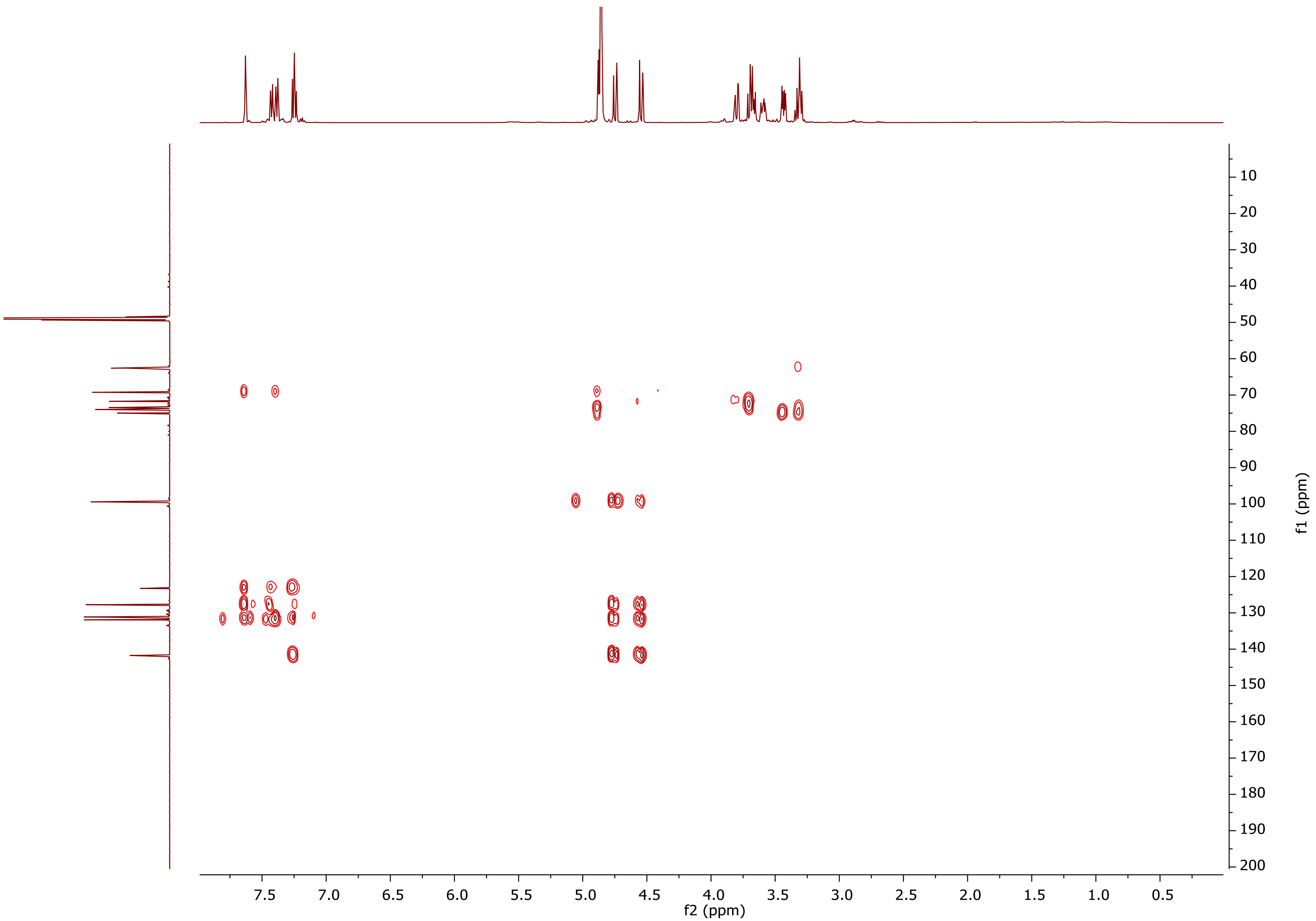

Figure SD207. ^{1}H-^{13}C HMBC NMR spectrum of *m*-bromo-benzyl-*α*-D-glucopyranoside (**30**) (500/125 MHz, MeOH-d_4)

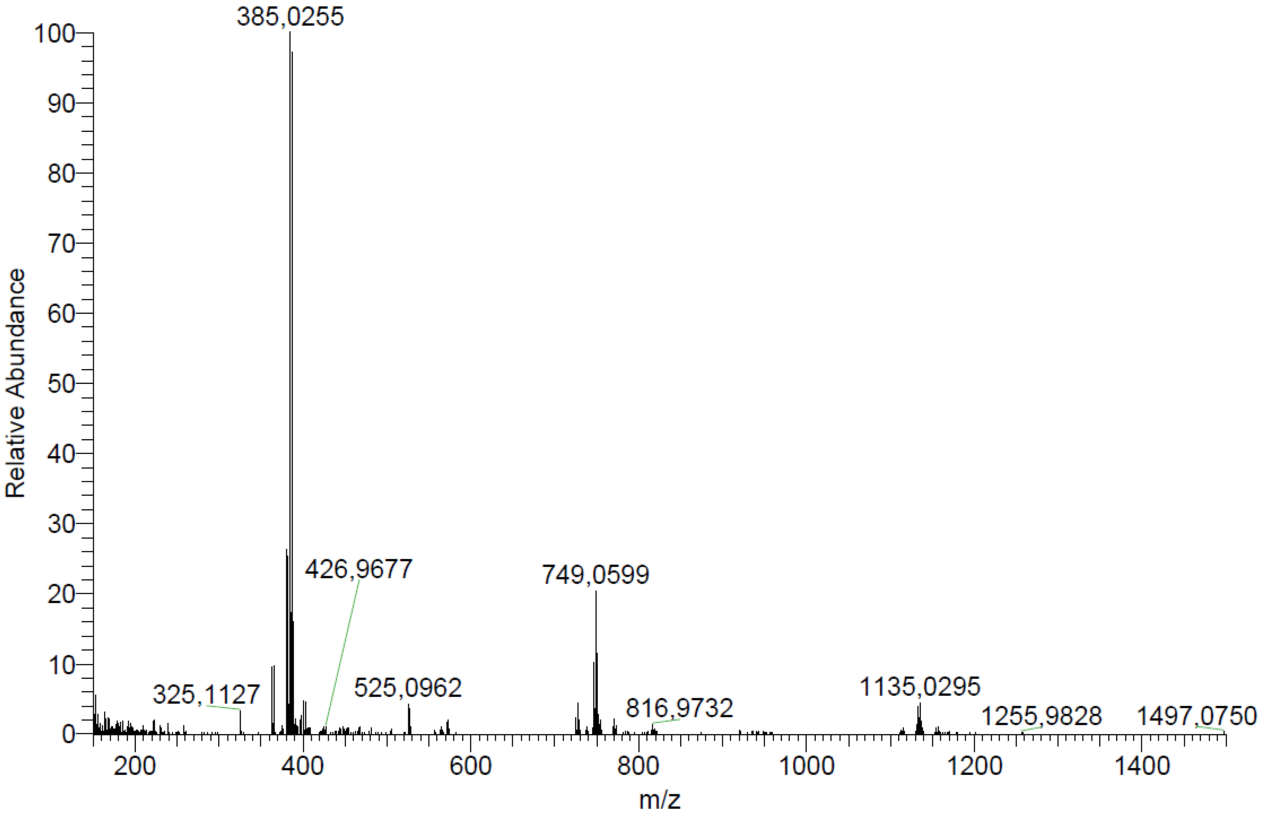

Figure SD208. ESI-HRMS spectrum of *m*-bromo-phenylethyl-α-D-glucopyranoside (**31**)

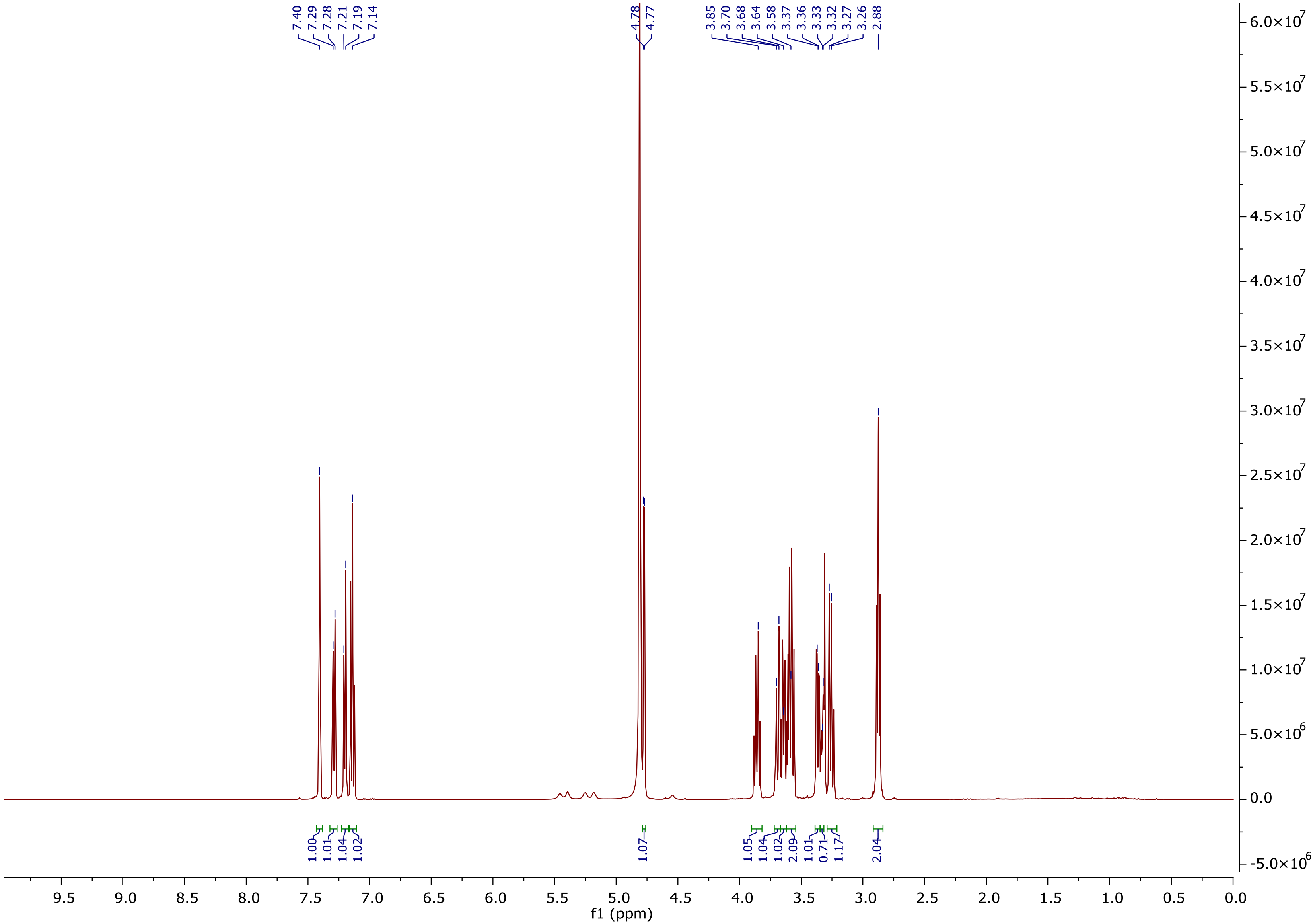

Figure SD209. ^{1}H NMR spectrum of *m*-bromo-phenylethyl-*α*-D-glucopyranoside (**31**) (500 MHz, MeOH-d_4)

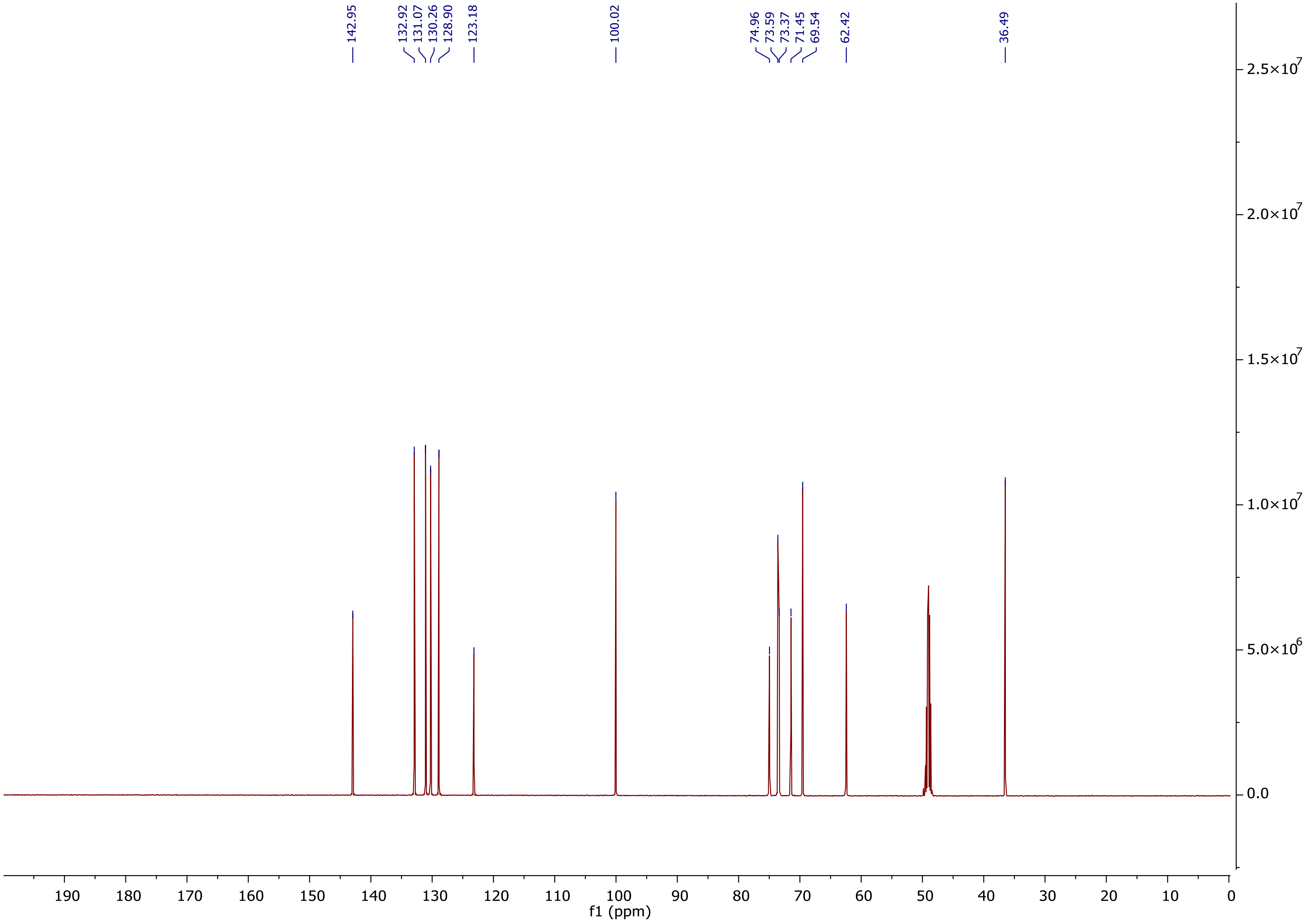

Figure SD210. ^{13}C NMR spectrum of *m*-bromo-phenylethyl-*α*-D-glucopyranoside (**31**) (125 MHz, MeOH-*d*$_4$)

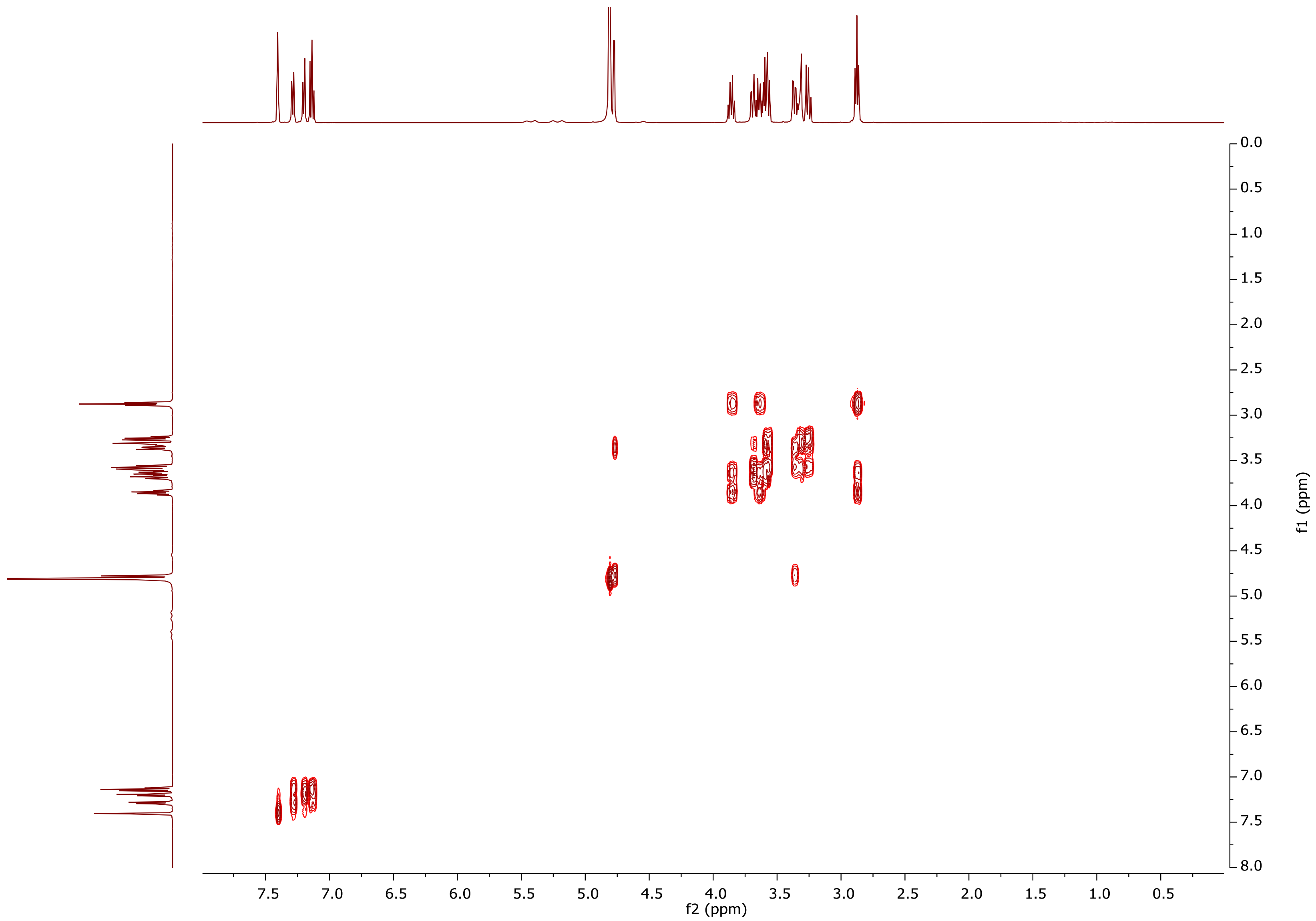

Figure SD211. ^{1}H-^{1}H COSY NMR spectrum of *m*-bromo-phenylethyl-*α*-D-glucopyranoside (**31**) (500/500 MHz, MeOH-d_4)

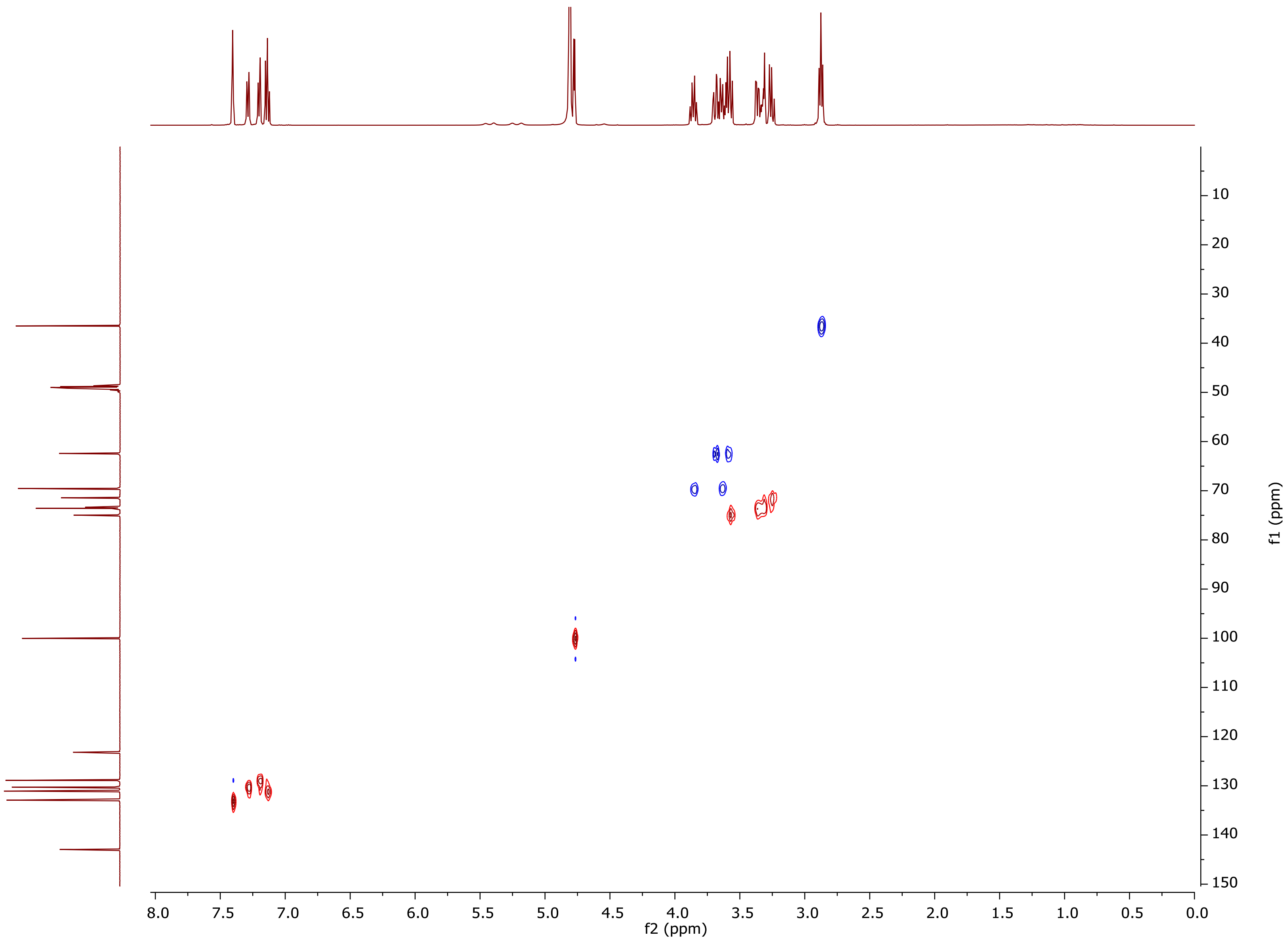

Figure SD212. ^{1}H-^{13}C HSQC NMR spectrum of *m*-bromo-phenylethyl-*α*-D-glucopyranoside (**31**) (500/125 MHz, MeOH-d_4)

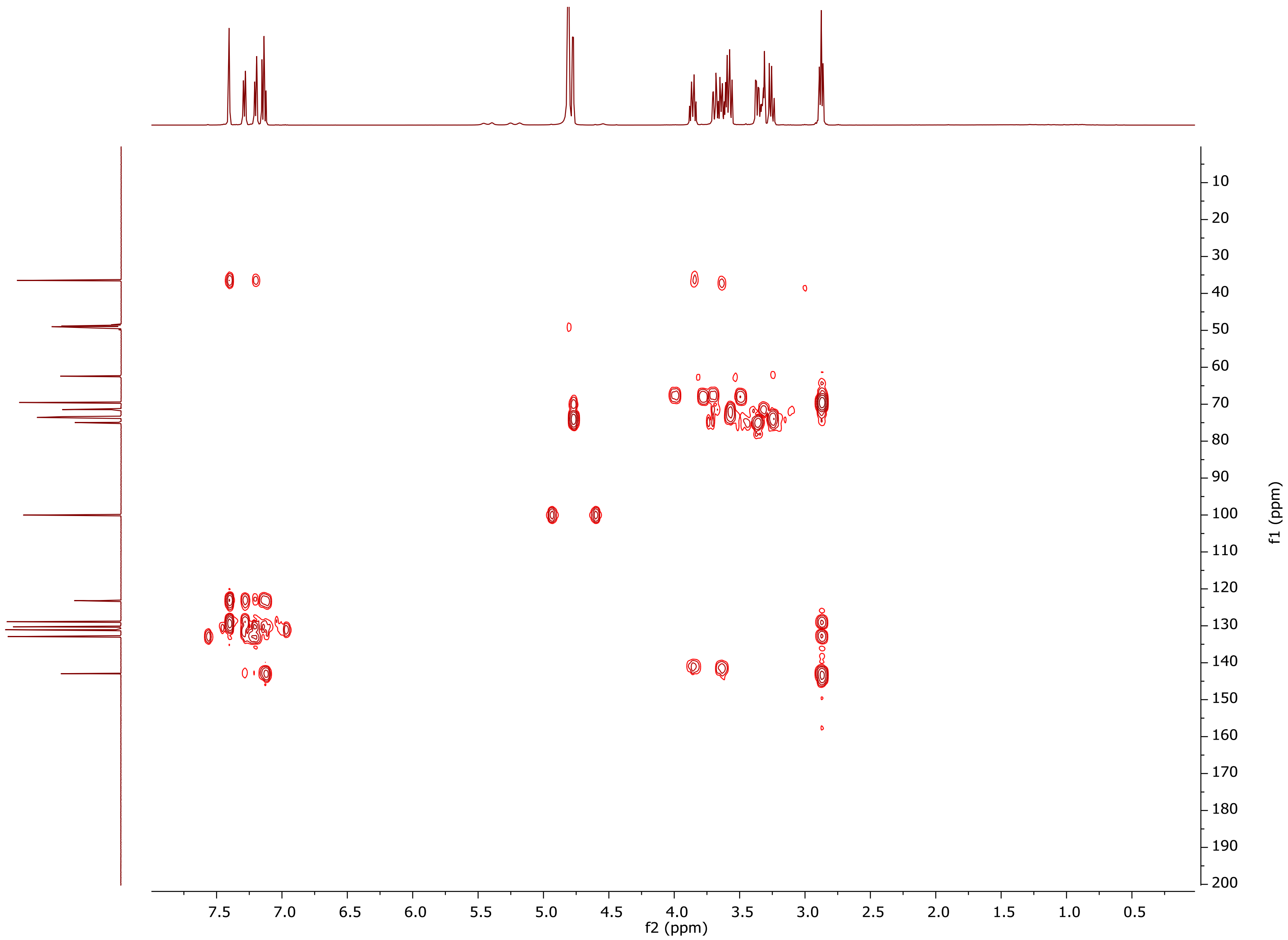

Figure SD213. ^{1}H-^{13}C HMBC NMR spectrum of *m*-bromo-phenylethyl-*α*-D-glucopyranoside (**31**) (500/125 MHz, MeOH-d_4)

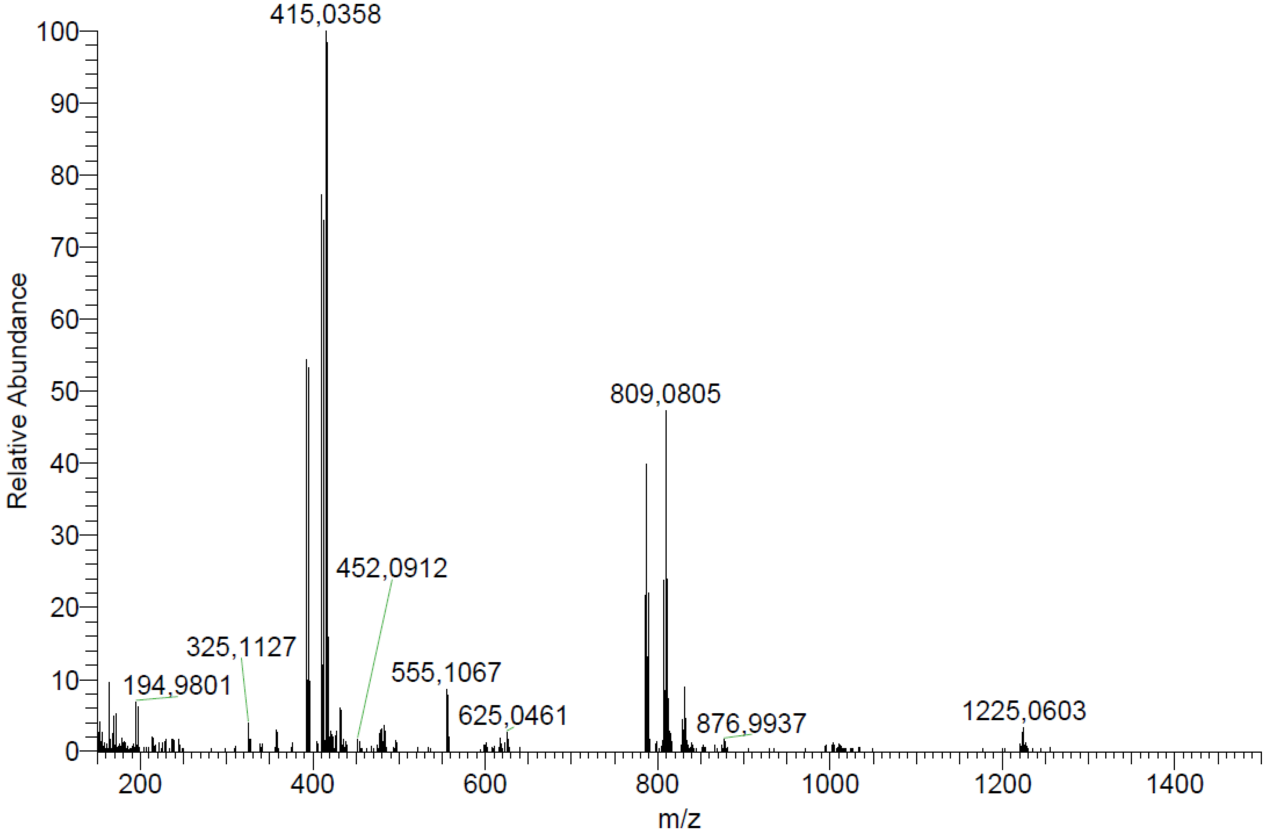

Figure SD214. ESI-HRMS spectrum of *m*-bromo-2-hydroxy-3-phenylpropyl-*α*-D-glucopyranoside (**32**)

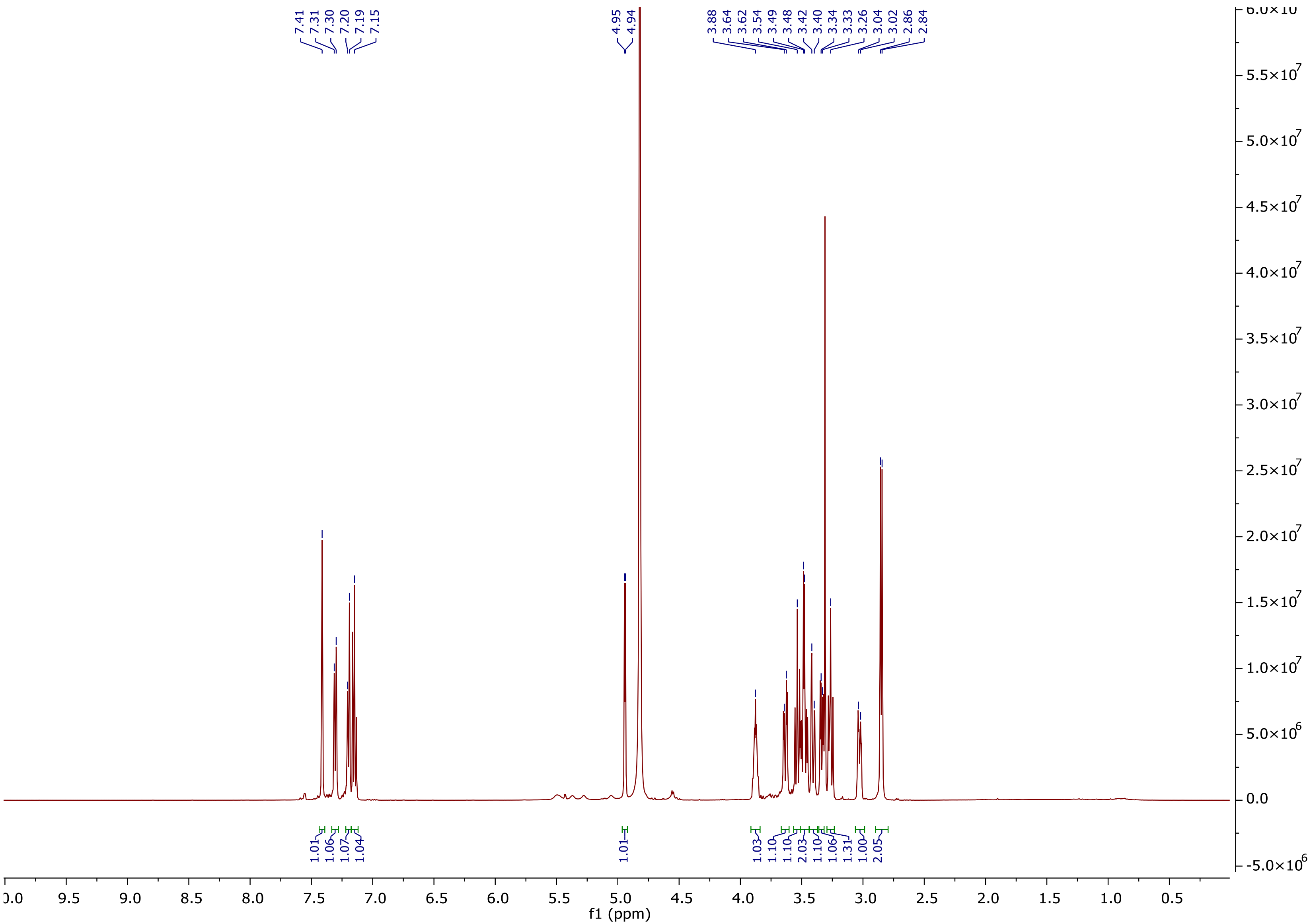

Figure SD215. ^{1}H NMR spectrum of *m*-bromo-2-hydroxy-3-phenylpropyl-*α*-D-glucopyranoside (**32**) (500 MHz, MeOH-d_4)

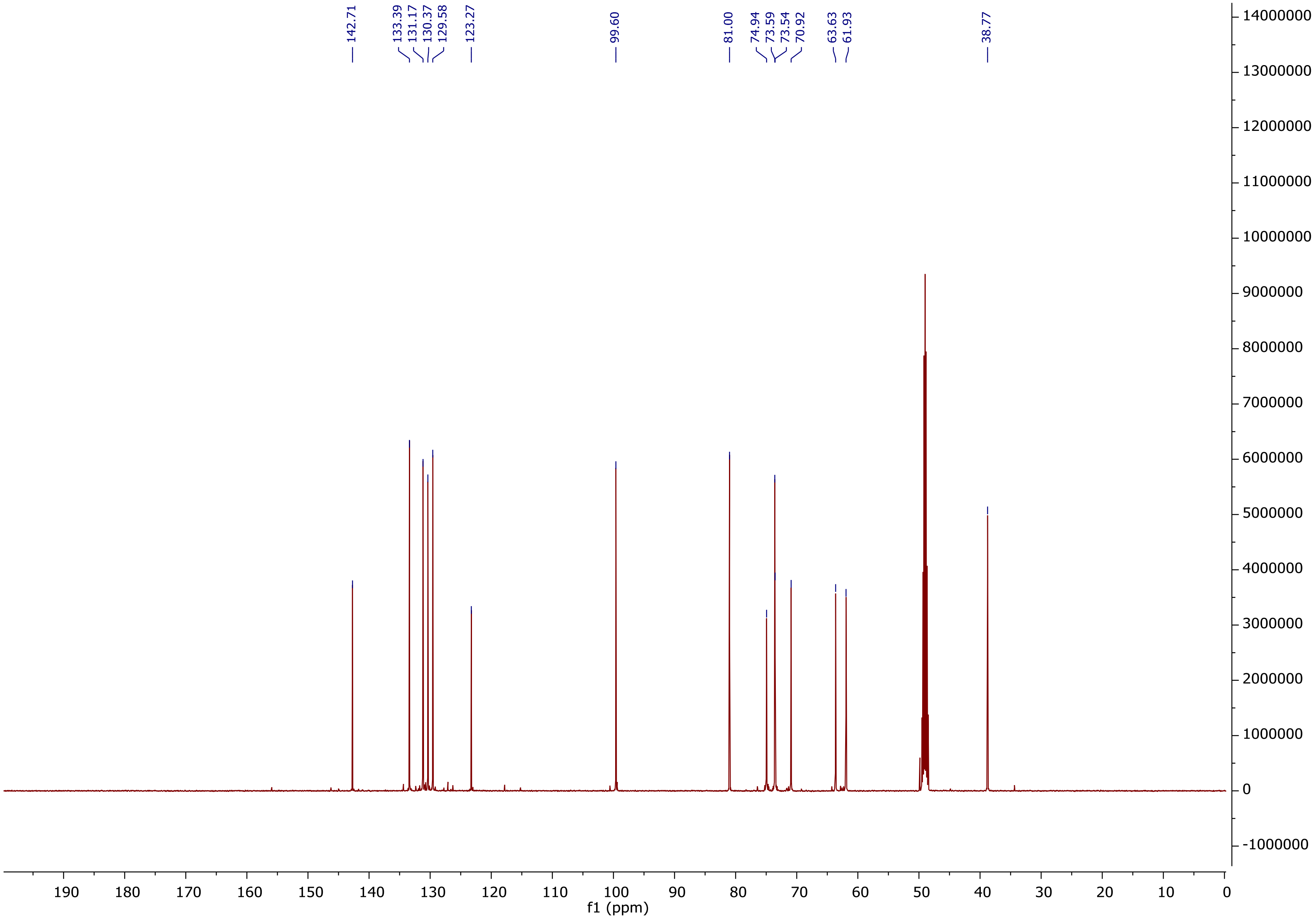

Figure SD216. ^{13}C NMR spectrum of *m*-bromo-2-hydroxy-3-phenylpropyl-*α*-D-glucopyranoside (**32**) (125 MHz, MeOH-*d*$_4$)

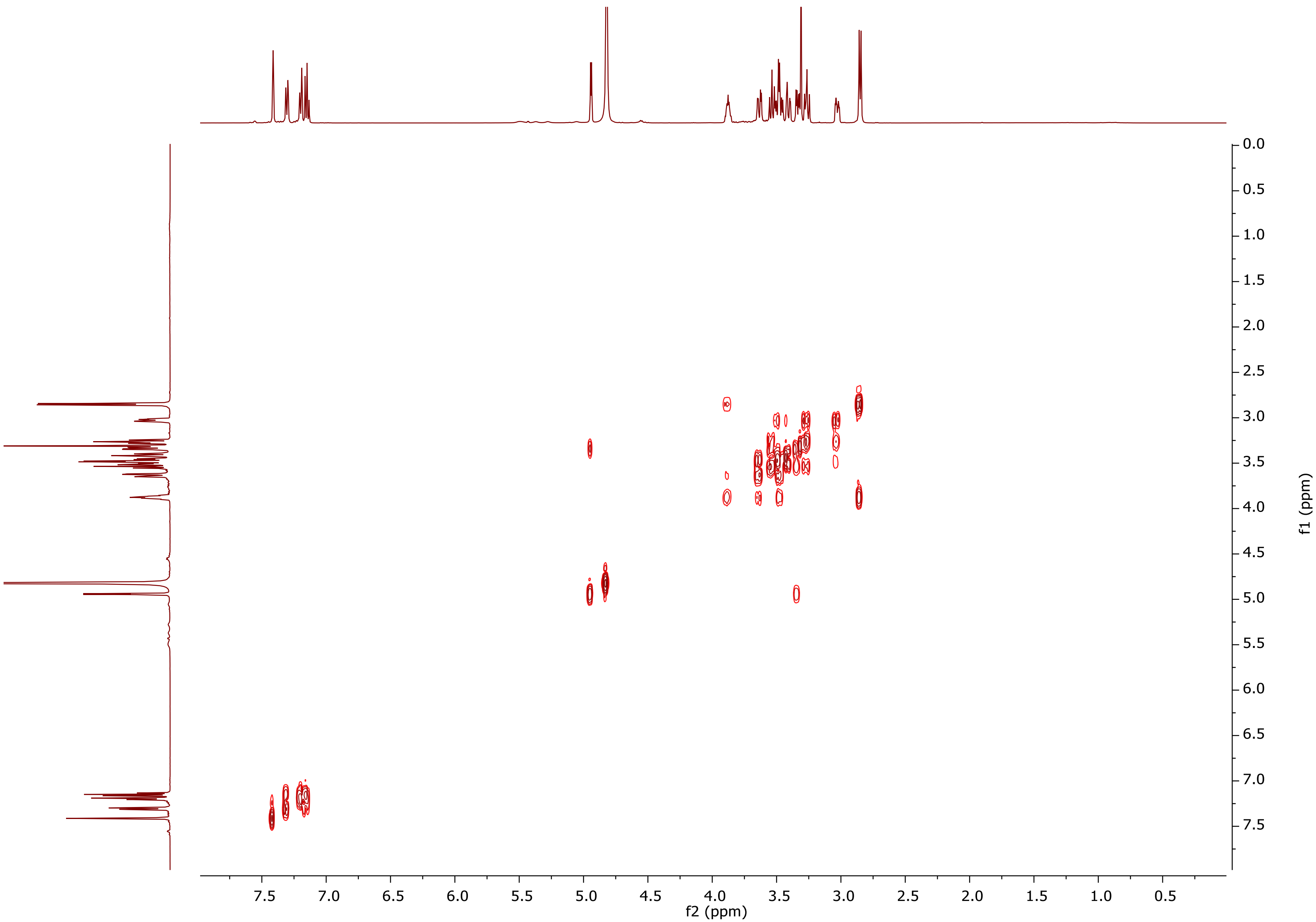

Figure SD217. ^{1}H-^{1}H COSY NMR spectrum of *m*-bromo-2-hydroxy-3-phenylpropyl-*α*-D-glucopyranoside (**32**) (500/500 MHz, MeOH-d_4)

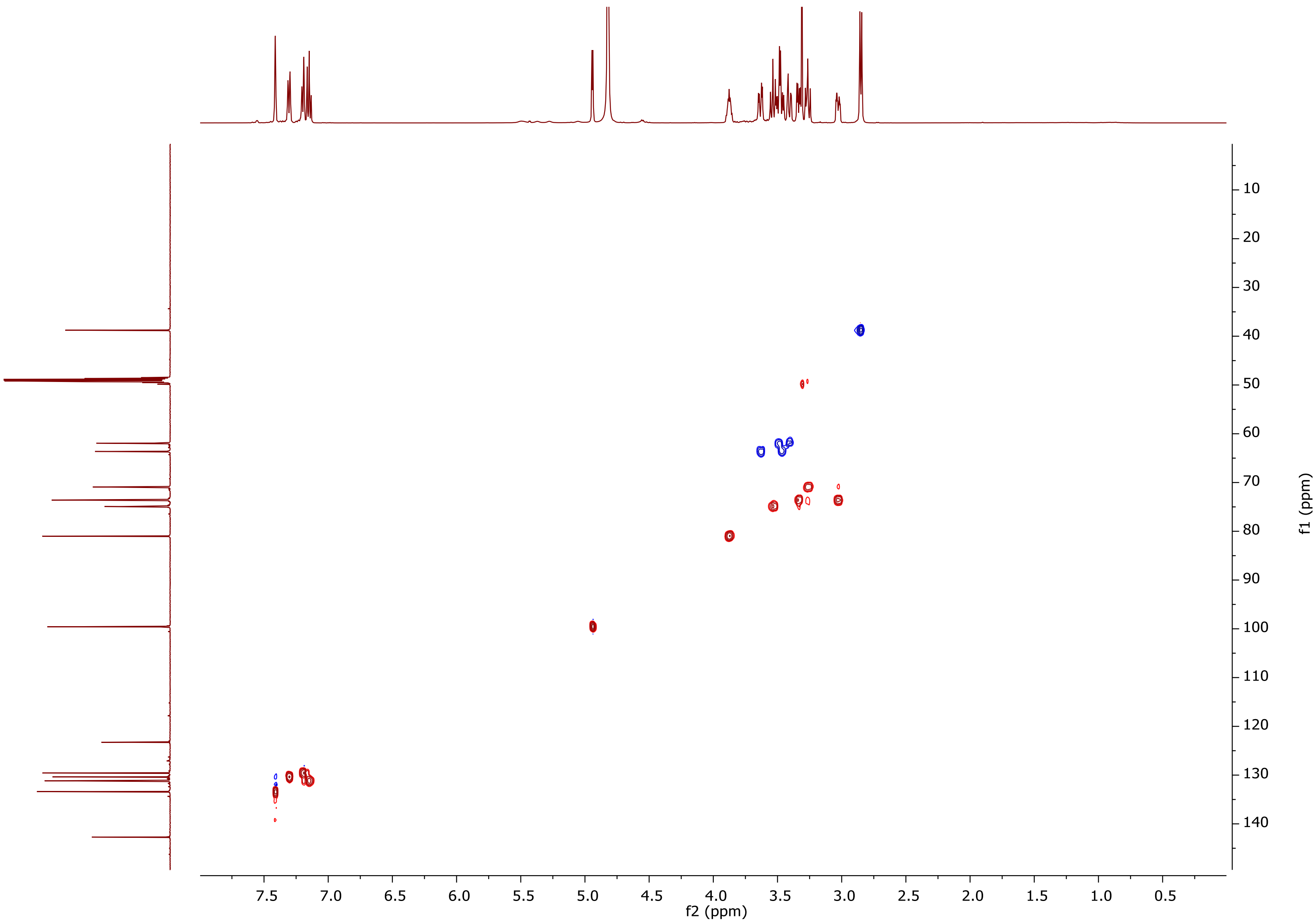

Figure SD218. ^{1}H-^{13}C HSQC NMR spectrum of *m*-bromo-2-hydroxy-3-phenylpropyl-*α*-D-glucopyranoside (**32**) (500/125 MHz, MeOH-*d*$_4$)

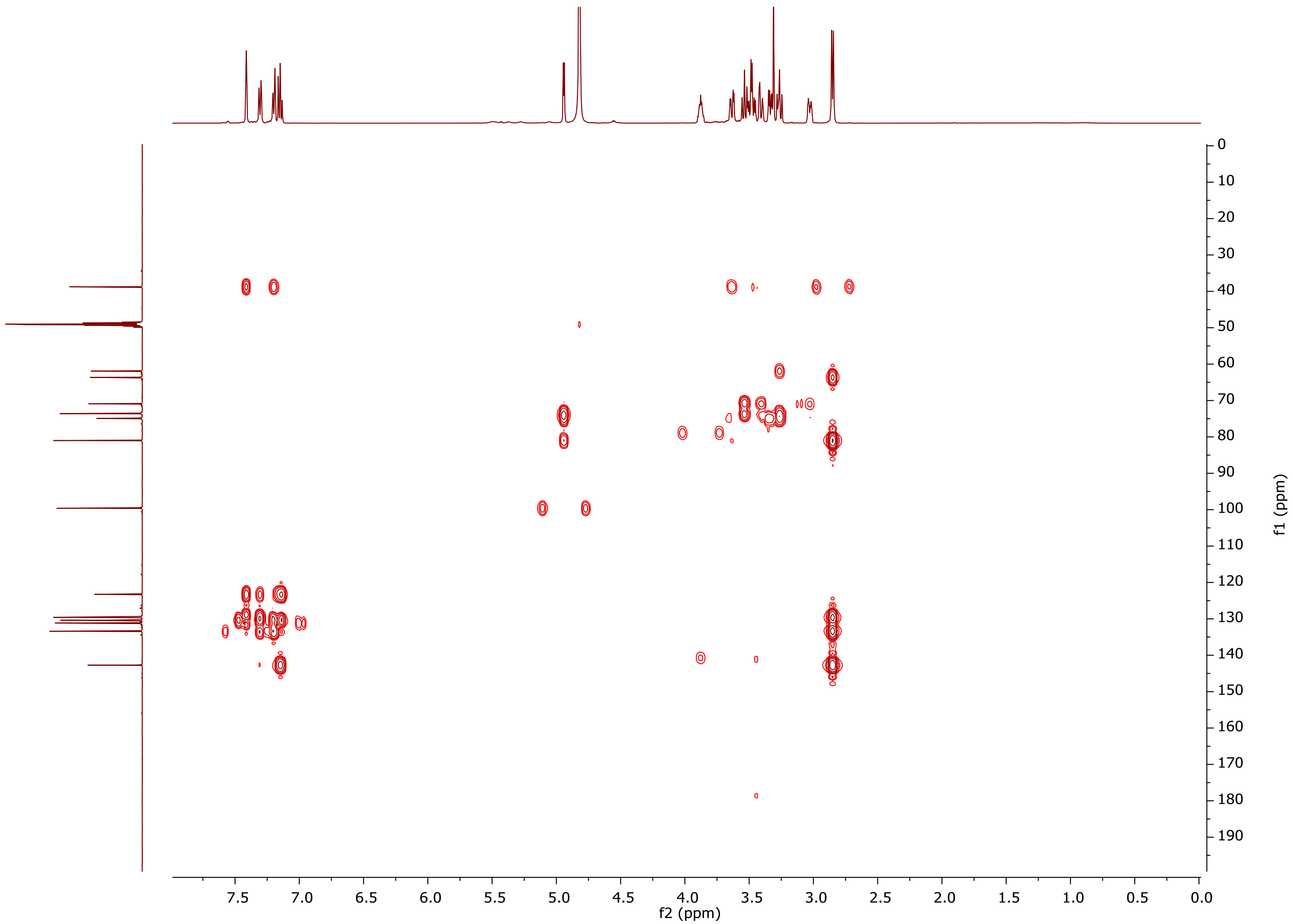

Figure SD219. ^1H-^{13}C HMBC NMR spectrum of *m*-bromo-2-hydroxy-3-phenylpropyl-*α*-D-glucopyranoside (**32**) (500/125 MHz, MeOH-d_4)
