# Supplementary material for: Microbial Cross-Talk: Unlocking the Cytochalasin Diversity from a Termite-Associated Xylaria
Source: JACS Au. 2025 Dec 22;6(1):179–92. doi: 10.1021/jacsau.5c01093 (PMC12848680; doi:10.1021/jacsau.5c01093)
Supplement: Supplementary file 2 [file au5c01093_si_002.pdf]

Supplementary Information

Microbial Cross-Talk: Unlocking the Cytochalasin Diversity from a Termite-Associated *Xylaria*

Marie Dayras,[1] Yaming Liu,[1] Rebecca Kochems,[1] Martinus de Kruijff,[1] Sven Balluff,[1] Sari Rasheed,[1] Andreas M. Kany,[1,2] Jennifer Herrmann,[1] Sebastian Götze,[1] Bernd Morgenstern,[3] N'Golo A. Koné,[4] Michael Poulsen,[5] Rolf Müller,[1,2,6] Christine Beemelmanns[1,6*]

Affiliation

1) Helmholtz Institute for Pharmaceutical Research Saarland (HIPS), Helmholtz Centre for Infection Research (HZI), Campus E8.1, 66123 Saarbrücken, Germany

2) Deutsches Zentrum für Infektionsforschung (DZIF) e.V., Braunschweig 38124, Germany

3) Inorganic Solid-State Chemistry, Saarland University, Campus Building C4 1, 66123 Saarbrücken, Germany

4) Unité de Formation et de Recherche en Sciences de la Nature (UFR-SN), Université Nangui Abrogoua, Station de Recherche en Ecologie du Parc National de la Comoé, 27 BP 847 Abidjan 27, Côte d'Ivoire

5) Section for Ecology and Evolution, Department of Biology, University of Copenhagen, 2100 Copenhagen East, Denmark

6) Pharma Science Hub (PSH), Saarland University, 66123 Saarbrücken, Germany.

*corresponding authors:

christine.beemelmanns@helmholtz-hips.de

1. Method Section

General experimental procedures

Consumables and devices for DNA extraction to genome sequencing. MinIon Mk1B (MIN-101B), MinION™ flow cell R10.4.1 (FLO-MIN114), Ligation Sequencing Kit V14 (SQK-LSK114), NEBNext® Companion Module for Oxford Nanopore Technologies® Ligation Sequencing (NEB, E7180S or E7180L), BlueRay Biotech EZDrop 1000, Agilent 4150 TapeStation System, and Agilent Genomic DNA ScreenTape devices were used, as well as Bovine Serum Albumin (BSA) (50 mg/ml) (e.g Invitrogen™ UltraPure™ BSA 50 mg/ml, AM2616) and Agilent Genomic DNA reagents, including ladder and sample buffer.

Chemicals. All media, solvents, and chemicals were purchased as follows: methanol, dichloromethane, ethyl acetate, acetonitrile (Th. Geyer, Renningen); water for analytical and preparative HPLC (Millipore, Germany), formic acid (Carl Roth, Germany), MeOH-*d*$_4$, $CDCl_3$ (Carl Roth, Germany), media ingredients (Carl Roth, Germany), reagents and solvents for synthesis (Sigma Aldrich, Germany).

Instruments. Semi-preparative HPLC was performed on a Vanquish HPLC system using a Phenomenex Luna C18(2) 250 x 10 mm column (particle size 5 µm, pore diameter 100 Å). Flash chromatography was performed on a Büchi Pure C-835 Prep using a Phenomenex Luna C18(2) 250 x 30 mm column (particle size 5 µm, pore diameter 100 Å).

UPLC-ESI-HRMS/MS. High resolution ultra-high performance liquid chromatography electrospray ionization mass spectrometry (HR-UPLC-ESI-MS) measurements were carried out on a Vanquish Flex UHPLC system (Thermo Scientific) combined with a Orbitrap Exploris 120 mass spectrometer (Thermo Scientific) equipped with an elecrospray ion (ESI) source and a Kinetex C18 column (50 x 2.1 mm, particle size 1.7 µm, pore diameter 100 Å). The column oven was set to 40 °C, scan range of MS was set to *m/z* 150 to 1,500 with resolution of 60,000 at *m/z* 200, AGC target 1e6 and maximum IT 100 ms. A combination of data dependent MS2 analysis and Top4 experiments was applied for MS2 measurements. MS2 measurements were performed in centroid mode with a resolving power of 30,000 at *m/z* 200, AGC target 1e5, and maximum IT 50 ms, an isolation window of 1.2 *m/z*, and a HCD collision energies of 20, 30, and 40. The spray voltage was set at 3.4 kV (positive mode) or 2.2 kV (negative mode), the capillary temperature at 320 °C and the vaporizer temperature at 300 °C. The sheath gas rate was set at 50 units N_2, and auxiliary gas rate at 13 units N_2. S-Lens RF level was set to 70. Each sample was analyzed through a gradient using H_2O + 0.1 % formic acid as eluent A and CH_3CN + 0.1 % formic acid as eluent B (0-1 min, 5% B, 1-10 min, 5-97% B, 10-12 min, 97% B, 12-13 min; 97-5% B, 13-15 min, 5% B) with an injection volume of 5 µl and a flow rate of 0.3 mL/min.

Feature-based molecular networking. The raw MS^2 data files were converted to mzML using MSconvert. The MZmine version 2.53 was then used for pre-processing.[1] A centroid mass detection of MS^1 at a noise level of 1.0E6 and MS^2 noise level of 1.0E3, generated a mass list. The *m/z* tolerance was set to 0.01 Da. The feature list was used to build chromatograms using ADAP. The minimum group number of scans was set at 5, the group intensity threshold was set at 1.0E6 and the minimum highest intensity was set at 3.0E6. Deconvolution of the feature lists was done with the Baseline cut-off algorithm. The feature lists were deisotoped and the features were aligned. Finally, duplicates were filtered, whereas fragments and adducts were searched. The resulting aligned list of features was exported in a mgf file along with their feature quantification table in csv format. These data as well as the mzML files were uploaded onto the feature-based molecular network (FBMN) page of GNPS.[2] The precursor and fragment ion masses were both set to 0.02 Da. The molecular network was visualized on Cytoscape version 3.9.1.[3] All data set used for molecular networks have been submitted to MassIVE repository.

NMR. ^{1}H NMR (500 MHz, 700 MHz) and ^{13}C NMR (125 MHz, 175 MHz) spectral data were performed on Bruker AVANCE III 500 MHz and 700 MHz spectrometers, equipped with a Bruker Cryo platform. The chemical shifts are reported in parts per million (ppm) relative to the solvent residual peak of $CDCl_3$ (^{1}H: 7.26 ppm; ^{13}C: 77.16 ppm) or MeOH-d_4 (^{1}H: 3.31 ppm; ^{13}C: 49.0 ppm).

2. Genome Sequencing

Cultivation of *Xylaria* sp. XSP14 for high molecular weight DNA extraction. Pre-Culture of *Xylaria* sp. XSP14 was cultivated on PDA. In order to obtain as much mycelium as possible for DNA extraction, a liquid culture of 100 ml PDB was inoculated with five 0.5 cm^2 PDA cubes from an XSP14 plate. A stainless steel spring was used to prevent the mycelium from clumping. Cultivation was carried out at 25°C and 150 rpm for 7 days. The culture liquid was then centrifuged (10,000 g for 10 min at r.t.) and the supernatant discarded so that the pure mycelium was frozen at -80°C. In the first step of DNA extraction, 2 g of the frozen mycelium were pulverized with liquid nitrogen in an autoclaved mortar. After adding 10 ml of CTAB buffer (BioChemica; A4150,0500), the suspension was incubated at 60°C for 30 min with regular inversion. The homogenate was then centrifuged (14,000 g for 5 min at r.t.) and the supernatant was transferred to a new tube. Subsequently, 100 µl of RNAse A solution was added and incubated for 20 min at 37 °C. After adding an equivalent volume of phenol/chloroform/isoamyl alcohol (25:24:1, Roth; A156.2), the sample was inverted for 10 min on a Hula mixer. Centrifugation was carried out (3000 g for 5 min), followed by transfer of the supernatant to a new tube using wide bore tips. Mixing with phenol/chloroform/isoamyl and subsequent centrifugation was repeated until the upper aqueous phase was clear. By adding and mixing with 0.7 volumes of ice-cold 100% isopropanol and incubating at -20°C for 15 min, the DNA was precipitated and visible as a pellet after additional centrifugation (10,000 g for 10 min at 4°C). To wash the pellet, 500 µl of ice-cold 70% ethanol and centrifugation (10,000 g for 10 min at 4°C) were used. After repeating the washing step, the ethanol was completely removed and the pellet dried for 3-5 min. The pellet was dissolved in 100 µl of nuclease-free water by gently flipping. The DNA concentration (1328 ng/µl) was determined using EZDrop 1000 (BlueRay Biotech) and the DNA fragment size and quality (1:10 diluted DNA sample of XSP14, DIN 8.3; 53676 bp; Figure S1) was determined using 4150 TapeStation System (Agilent).

Nanopore library preparation and whole genome sequencing. The Nanopore Library Preparation was carried out according to the protocol provided by Oxford Nanopore Technologies "Ligation sequencing DNA V14 (SQK-LSK114)" (Ligation sequencing DNA V14 (SQK-LSK114)). In the first step, "DNA repair and end-prep", 6.6 µg of extracted DNA from *Xylaria* sp. XSP14 was used. The 1 µl DNA control sample was omitted and instead the DNA volume was increased by this microliter. At the end of the "DNA repair and end-prep", a concentration of 39 ng/µl was measured, which corresponds to a total DNA content of 2.4 µg in 61 µl. In the following step, "Adapter ligation and clean-up", the Short Fragment Buffer (SFB) was used and completed with a concentration of 65 ng/µl (total amount of 975 ng DNA in 15 µL). When priming and loading the MinION Flow Cell (R10.4.1), 12 µl of the processed DNA

was used, so that 780 ng of DNA was loaded. The Nanopore sequencing run was performed with the MinION Mk1B device for 72 hours without basecalling. The "active pore" number of the MinION FlowCell measured before the run was 1568.

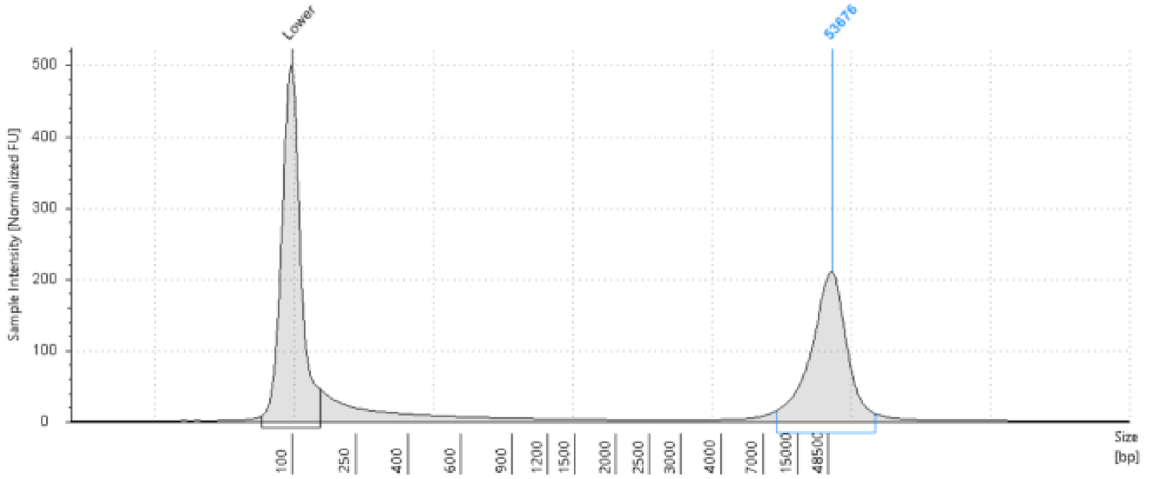

Sample Table

Well	DIN	Conc. [ng/µl]	Sample Description	Alert	Observations
B1	8.3	9.64	XSP-14 7 days old dil.	⚠	Sample concentration outside recommended range

Peak Table

Size [bp]	Calibrated Conc. [ng/µl]	Assigned Conc. [ng/µl]	% Integrated Area	From [bp]	To [bp]	Peak Comment	Observations
100	8.50	8.50	-	65	151		Lower Marker
53676	6.73	-	100.00	9625	>60000		

Figure S1. Electropherogram of a 1:10 dilution of XSP14 DNA. A peak of DNA fragment size is visible at 53676 bp.

Genome assembly and annotation. Nanopore sequencing raw data was base-called and then trimmed using dorado v0.9.0 (Oxford Nanopore Technologies). Resulted reads were then filtered by filtlong (https://github.com/rrwick/Filtlong), with the parameter '--min_length 100 --keep_percent 95'. A hybrid *de novo* genome assembly, combing BGISeq and Oxford Nanopore data, was performed using SPAdes v4.0.0.[4] The resulting draft assembly was then polished with the accurate Illumina reads using pilon v1.24,[5] and then masked by RepeatMasker v4.1.7.[6]

The final genome was annotated using the RNA sequencing reads in BRAKER v3.0.8[7] creating an Augustus species model for the *Xylaria* sp. XSP14. Annotation quality was estimated by BUSCO v5.8.1 with the *sordariomycetes_odb10* dataset.[8] Biosynthetic gene clusters were identified using the fungal version of antiSMASH version 8 beta 1.[9]

Orthology inference and species tree construction. Orthologous gene families were identified using OrthoFinder v3.0.1b1.[10] The software inferred orthogroups from predicted protein sequences using all-versus-all DIAMOND sequence similarity searches.[11] Gene trees were reconstructed for each orthogroup using FastTree,[12] and the species tree was inferred using STAG,[13] which integrates information from single-copy and multi-copy orthologs. The tree was then rooted using STRIDE,[14] which accounts for gene duplication events.

Phylogenetic analysis. For phylogenetic analysis, sequences were aligned with MAFFT v7.407_1[15] and then the alignment was trimmed with BMGE v1.12_1. The gene trees were constructed using FastTree v.2.1.11.[12] The analysis was done with the web tool NGPhylogeny.fr.[16] The trees were visualized in iTOL v7.0.[17] The default parameters were used if not described specifically.

BGC comparison and Stachelhaus code analysis. Gene pairs in BGCs are compared and visualized using clinker v0.0.29. The substrate binding residues of the PKS-NRPS adenylation domain, also known as the Stachelhaus code, are predicted and identified using the program NRPSpredictor2.[18]

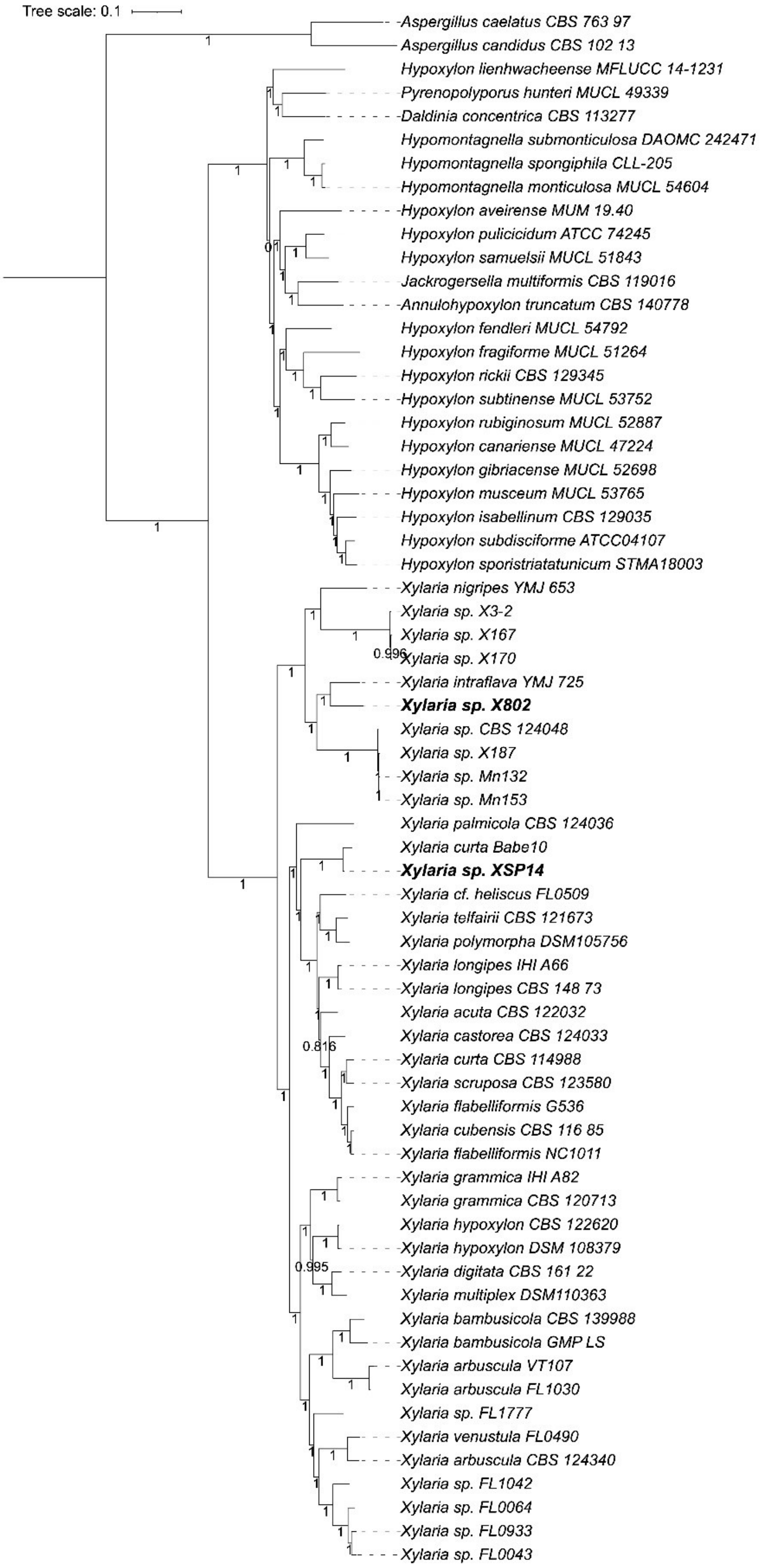

Figure S2. Phylogenomic tree of *Xylaria* species, including the species investigated in this study. Genomes of *Xylaria* spp. obtained from NCBI, along with published genomes of Hypoxylaceae were included in the analysis. Orthogroups were identified across all species, followed by the inference of gene trees for each orthogroup. A final species tree was constructed based on a selection of gene trees. The analysis was performed using OrthoFinder, with *Aspergillus caelatus* and *Aspergillus candidus* serving as outgroup species.

Table S1. Sequencing, assembly and annotation statistics of genomes from for *Pseudoxylaria* spp. and *Xylaria* spp. included in this study (I: BGIseq, D: DNBSEQ, IT: Illumina,TI: Ion Torrent S5, PB: PacBio, ONT: Oxford Nanopore Technologies)

Organism	Acc. No.	Raw Data	Assembly length (Mbp)	# Seq.	N50 (Mbp)	L50	BUSCO (C %)	Location	Host/ Lifestyle	Reference
***Xylaria* sp. XSP14**	JBPOEM000000000	D, ONT	50.2	1134	0.346	37	98.6	Ivory Coast	*Macrotermes bellicosus*	Li et al[19], this study
***Xylaria* sp. Mn132**	JAJFDL000000000	I	35.0	342	0.26	46	96.6	South Africa	*Macrotermes natalensis*/ Termite-associated stowaway (*Macrotermes natalensis*)	Fricke et al.[20]
***Xylaria* sp. Mn153**	JAJFDM000000000	I	34.5	355	0.23	49	93.3	South Africa	*Macrotermes natalensis*/ Termite-associated stowaway	Fricke et al.[20]
***Xylaria* sp. X167**	JAJFDK000000000	I	33.2	454	0.22	48	95.2	South Africa	*Odontotermes* sp./ Termite-associated stowaway	Fricke et al.[20]
***Xylaria* sp. X170**	JAJFDJ000000000	I	33.7	339	0.22	49	96.6	South Africa	*Odontotermes* sp./ Termite-associated stowaway	Fricke et al.[20]
***Xylaria* sp. X187**	JAJIZN000000000	PB,ONT	39.7	34	2.94	6	96.4	South Africa	*Macrotermes natalensis*/ Termite-associated stowaway	Fricke et al.[20]
***Xylaria* sp. X3-2**	JAJFDI000000000	I, ONT	36.9	742	0.267	41	96.2	South Africa	*Macrotermes natalensis*/ Termite-associated stowaway	Fricke et al.[20]
***Xylaria* sp. X802**	JAJFDH000000000	I, ONT	40.4	106	0.63	20	96.4	South Africa	*Macrotermes natalensis*/ Termite-associated stowaway	Fricke et al.[20]
***Xylaria flabelliformis* G536**	GCA_007182795.1	I	41.2	155	0.49	28	93.6	USA:North Carolina	Saprotroph and Endophyte; Isolated on *Asimina triloba*	Mead et al.[21]
***Xylaria grammica* IHI A82**	GCA_004014815.1	IT	47.0	1,053	0.08	172	-	Kenya: Kakamega Forest	Endophyte; deadwood	-
***Xylaria hypoxylon* CBS 122620**	GCA_902806585.1	I, ONT	54.3	88	3.89	6	-	-	Saprotroph	Wibberg et al. 2020[22]
***Xylaria hypoxylon* DSM 108379**	GCA_004768795.1	IT	42.8	635	0.12	102	96.0	Germany: Bad Lobenstein	Saprotroph; deadwood	Büttner et al. 2019b[23]
***Xylaria longipes* IHI A66**	GCA_003426265.1	IT	43.2	1,006	0.07	185	90.6	Germany: Bavarian Forrest	Saprotroph; *Acer pseudoplatanus*	Büttner et al. 2019a[23]
***Xylaria multiplex* DSM 110363**	GCA_011057905.1	IT	45.6	389	0.24	57	96.4	USA: Puerto Rico	Saprotroph;Isolated from deadwood	Büttner et al. 2020[23]
***Xylaria polymorpha* DSM 105756**	GCA_003426235.1	IT	43.5	947	0.07	173	-	Germany: Zittau, Westpark	Saprotroph;isolated from beech	-
***Xylaria nigripes* YMJ 653**	GCA_022984845.1	PB	34.6	64	1.1	12	-	Taiwan	-	-
***Xylaria intraflava* YMJ 725**	GCA_022385655.1	IT	35.9	438	0.23	42	-	Taiwan	-	-
***Xylaria palmicola* CBS 124036**	GCA_022385595.1	IT	43.9	548	0.367	35	-	-	-	Franco MEE et al.2022[24]
***Xylaria curta* Babe10**	GCA_027595895.1	TI	44.2	5737	0.013	1005	-	Viet Nam: Ba Be Nationalpark	Isolated on deadwood	-
***Xylaria cf. heliscus* FL0509**	GCA_022539335.1	PB	44.9	72	1.5	11	-	USA: Archbold Biological Station	-	Franco MEE et al. 2022[24]
***Xylaria telfairii* CBS 121673**	GCA_022495065.1	IT	46.9	1027	0.197	66	-	-	-	Franco MEE et al. 2022[24]
***Xylaria acuta* CBS 122032**	GCA_022495125.1	IT	47.2	761	0.36	41	-	-	-	Franco MEE et al. 2022[24]
***Xylaria castorea* CBS 124033**	GCA_022495115.1	IT	45.9	461	0.56	22	-	-	-	Franco MEE et al. 2022[24]
***Xylaria scruposa* CBS 123580**	GCA_022385635.1	IT	46.6	589	0.62	25	-	-	-	
***Xylaria cubensis* CBS 116.85**	GCA_022385715.1	IT	44.8	333	0.44	29	-	-	-	
***Xylaria digitata* CBS 161.22**	GCA_022495195.1	IT	58.4	888	0.18	85	-	-	-	
***Xylaria arbuscula* VT107**	GCA_027627345.1	TI	42.1	3895	0.015	877	-	Viet Nam: Ninh Binh Province, Cuc Phuong National Park	Isolated on deadwood	-
***Xylaria bambusicola* CBS 139988**	GCA_022495145.1	IT	45.7	233	0.56	23	-	Thailand: Chiang Mai Province	-	
***Xylaria venustula* FL0490**	GCA_022578775.1	IT	50.8	392	0.427	39	-	USA: Archbold Biological Station, Highlands Co., Florida	-	
***Xylaria sp.* FL0043**	GCA_022578865.1	IT	44.5	933	0.34	38	-	USA: Archbold Biological Station, Highlands Co., Florida	-	

3. Cultivation of *Xylaria* spp. in axenic and co-cultures

Time and media studies: Ten PDA plates and 10 MEA plates (15 mL medium, 92x16 mm) of 2-week-old *Xylaria* sp. X802 and XSP14 cultures were inoculated with 2 agar cubes. The plates were kept at 25 °C in the dark. The production of metabolites was monitored by HR-UPLC-ESI-MS/MS analyses. For that, 2 plates of each condition were extracted at 11 days, 15 days, 20 days, 26 days and 33 days of culture by cutting them into pieces and soaking them in MeOH overnight. The organic extract was filtered, dried under vacuum, dissolved in MeOH with a final concentration of 50 µg/mL, and analyzed by HRMS.

Data set of the molecular network has been submitted to MassIVE server: MSV000098580.

Table S2. Media composition.

Medium	Compound	Concentration	Vendor
**PDA (Potato extract Glucose Agar)**	Potato extract	4 g/L	Carl Roth
	Glucose	20 g/L	(X931)
	Agarose	15 g/L	
**MEA (Malt Extract Agar)**	Malt extract	20 g/L	Carl Roth
	Agarose	20 g/L	Carl Roth
**PDB (Potato extract Glucose Broth)**	Potato extract	6,5 g/L	Carl Roth
	Glucose	20 g/L	(CP74)

Preparative cultivation of axenic *Xylaria* sp. X802: A total of five PDA plates (15 mL PDA, 92x16 mm) of a 2-week-old *Xylaria* sp. X802 culture were used to inoculate 50 PDA plates (15 mL PDA, 92x16 mm), which were kept at 25 °C for 3 weeks in the dark. Agar plates were then cut into pieces, soaked in MeOH overnight. The organic extract was filtered and dried under vacuum. The crude extract (3 g) was submitted to SPE-C18 open column purification by step-wise elution using H_2O/MeOH mixtures. In total, 5 fractions were obtained. Fraction 5 (135 mg) was subjected to semi-preparative reverse-phase HPLC (Phenomenex Luna C18(2) 250 x 10 mm 5 µm, 100 Å column,) using the following gradient: 0–5 min, 30% B; 5–36 min, 30%–100% B; 36–44 min, 100% B (A: H_2O + 0.1% Formic acid; B: CH_3CN + 0.1% Formic acid) with a flow rate of 3 mL/min to yield deacetyl-19,20-epoxycytochalasin C (**4**, 2 mg), 19,20-epoxycytochalasin C (**3**, 11.6 mg), 19,20-epoxycytochalasin D (**9**, 0.9 mg), pseudoxylallemycin A (5.5 mg), pseudoxylallemycin B (4 mg), pseudoxylallemycin C (7 mg), pseudoxylallemycin D (0.6 mg), pseudoxylallemycin E (0.7 mg), and pseudoxylallemycin F (0.7 mg). Pseudoxylallemycin A-F: their HRMS data and ^{1}H NMR spectra matched reported data.[25]

Preparative cultivation of axenic *Xylaria* sp. XSP14: A total of five PDA plates of a 2-week-old *Xylaria* sp. XSP14 culture were used to inoculate two 5 L-Erlenmeyers filled with 1 L of PDB and four 2 L-Erlenmeyers filled with 500 mL PDB, for a total of 4 L of culture. After 1-month incubation at 25 °C at 150 rpm, the supernatant and mycelium were separated through centrifugation and filtration. Diaion HP20 resin was then added to the supernatant after conditioning and extracted with MeOH. The organic extract was dried under vacuum to afford 4.2 g of supernatant crude extract. The mycelium was freeze-dried and extracted with MeOH. The organic extract was dried under vacuum to afford 2.5 g of dried mycelium crude extract. Both crude extracts (6.0 g for the supernatant and 2.5 g for the mycelium) were then fractionated by SPE-C18 open columns by stepwise elution using H_2O/MeOH mixtures to afford 5 fractions for each crude extract. Fraction 4 from supernatant extraction (420 mg) was subjected to semi-preparative reverse-phase HPLC (Phenomenex Luna C18(2) 250 x 10 mm, 5 µm, 100 Å column) using the following gradient: 0–5 min, 35% B; 5–37 min, 35%–100% B; 37–45 min, 100% B (A: H_2O + 0.1% Formic acid; B: CH_3CN + 0.1% Formic acid) with a flow rate of 3 mL/min to yield deacetyl-19,20-epoxycytochalasin C (**4**, 21.5 mg), 19,20-epoxycytochalasin R (**8**, 1.2 mg), deacetyl-18-desoxy-19,20-epoxycytochalasin Q (**1**, 5.1 mg), 19,20-epoxycytochalasin C (**3**) and 19,20-epoxycytochalasin Q (**6**) in mixture (50 mg), cytochalasin C (**2**, 1.6 mg), and 18-desoxy-19,20-epoxycytochalasin C (**5**, 1.2 mg). Fraction 5 from supernatant extraction (109 mg) was subjected to semi-preparative reverse-phase HPLC (Phenomenex Luna C18(2) 250 x 10 mm, 5 µm, 100 Å column) using the following gradient: 0–5 min, 30% B; 5–40 min, 30%–75% B; 40–43 min, 75%-100% B; 43-50 min, 100% B (A: H_2O + 0.1% Formic acid; B: CH_3CN + 0.1% Formic acid) with a flow rate of 3 mL/min to yield deacetyl-19,20-epoxycytochalasin C (**4**, 7 mg), 19,20-epoxycytochalasin C (**3**) and 19,20-epoxycytochalasin Q (**6**) in mixture (30 mg), and 18-desoxy-19,20-epoxycytochalasin C (**5**, 1.4 mg). Fraction 4 from mycelium extraction (150 mg) was subjected to semi-preparative reverse-phase HPLC (Phenomenex Luna C18(2) 250 x 10 mm, 5 µm, 100 Å column) using the following gradient: 0–5 min, 30% B; 5–30 min, 30%–100% B; 30–37 min, 100% B (A: H_2O + 0.1% Formic acid; B: CH_3CN + 0.1% Formic acid) with a flow rate of 3 mL/min to yield deacetyl-19,20-epoxycytochalasin C (**4**, 19.5 mg), 19,20-epoxycytochalasin R (**8**, 2 mg), 19,20-epoxycytochalasin C (**3**) and 19,20-epoxycytochalasin Q (**6**) in mixture (30 mg), 19,20-epoxycytochalasin D (**9**, 1.2 mg), and 18-desoxy-19,20-epoxycytochalasin C (**5**, 2.4 mg).

Testing of acid susceptibility of 19,20-epoxycytochalasin Q: 19,20-epoxycytochalasin Q (**6,** 1 mg) was dissolved in 1 mL of CH_2Cl_2 and 1% of formic acid was added at once. The reaction was stirred at room temperature overnight. After evaporation, UHPLC-HRMS was conducted and 19,20-epoxycytochalasin C (**3**) was obtained and characterized by comparison with the isolated reference (Figure S3).

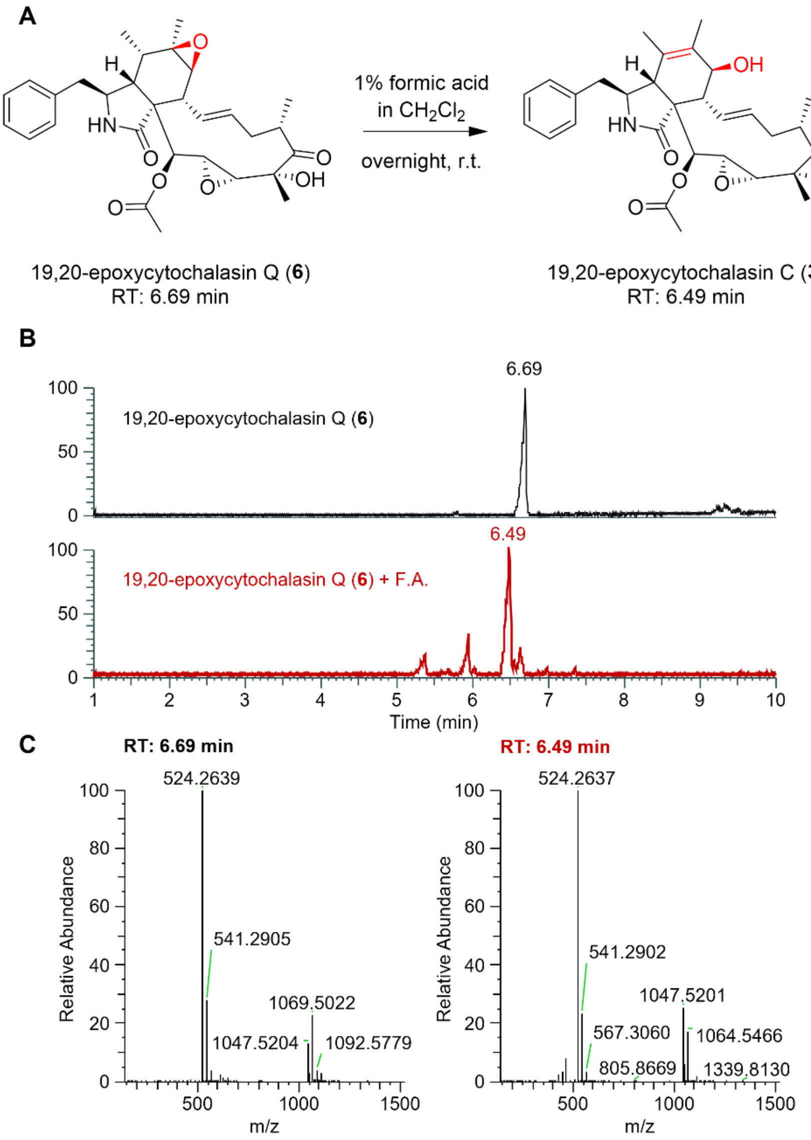

Figure S3. A) Reaction scheme of 19,20-epoxycytochalasin Q (**6**) with 1% of formic acid in dichloromethane; B) UHPLC-HRMS chromatograms of 19,20-epoxycytochalasin Q (**6**) and 19,20-epoxycytochalasin Q (**6**) treated with formic acid; C) mass spectrum of the peak at 6.69 min from UHPLC-HRMS analysis of 19,20-epoxycytochalasin Q (**6**) and mass spectrum of the peak at 6.49 min of 19,20-epoxycytochalasin Q (**6**) treated with formic acid.

Physical chemical data of isolated cytochalasins

Deacetyl-18-desoxy-19,20-epoxycytochalasin Q (1):

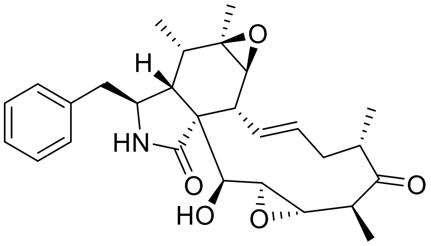

ESI-HRMS $[M+H]^+$ m/z 466.2583 (calcd for $C_{28}H_{36}NO_5$, 466.2588).

NMR spectral data, see Table S3.

Cytochalasin C (2):

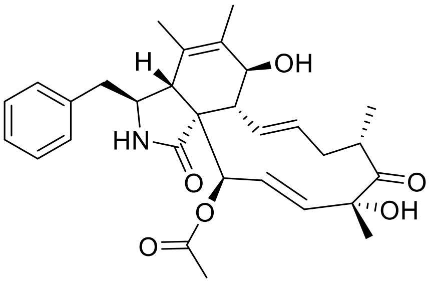

ESI-HRMS $[M+H]^+$ *m/z* 508.2687 (calcd for $C_{30}H_{38}NO_6$, 508.2694). ^1H NMR (700 MHz, $CDCl_3$) *δ* 7.31 (s, 2H), 7.24 (s, 1H), 7.17 (d, *J* = 7.7 Hz, 2H), 6.01 (dd, *J* = 15.6, 1.2 Hz, 1H), 5.90 (m, 1H), 5.56 (s, 1H), 5.34 (ddd, *J* = 15.5, 10.5, 5.3 Hz, 1H), 5.16 (dd, *J* = 15.5, 1.9 Hz, 1H), 4.65 (s, 1H), 3.78 (d, *J* = 9.7 Hz, 1H), 3.33 (s, 1H), 2.96 (m, 2H), 2.74 (m, 1H), 2.53 (q, *J* = 11.4 Hz, 1H), 2.47 (s, 1H), 2.44 (t, *J* = 10.1 Hz, 1H), 2.26 (s, 3H), 2.12 (s, 1H), 2.01 (dd, *J* = 13.0, 4.4 Hz, 1H), 1.68 (s, 3H), 1.51 (s, 3H), 1.46 (s, 3H), 1.20 (d, *J* = 6.8 Hz, 3H). ^{13}C NMR (175 MHz, $CDCl_3$) *δ* 210.1, 174.4, 170.1, 137.7, 134.3, 132.1, 131.8, 131.4, 129.2, 129.1, 128.3, 127.2, 126.5, 78.0, 75.2, 68.2, 60.3, 50.3, 49.8, 44.8, 42.5, 37.8, 24.4, 21.1, 19.5, 17.4, 14.1.

19,20-epoxycytochalasin C (3):

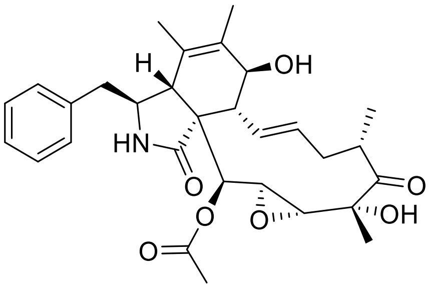

ESI-HRMS $[M+H]^+$ *m/z* 524.2634 (calcd for $C_{30}H_{38}NO_7$, 524.2643). ^1H NMR (500 MHz, $CDCl_3$) δ 7.32 (t, *J* = 7.4 Hz, 2H), 7.23 (m, 3H), 6.15 (m, 1H), 5.76 (s, 1H), 5.73 (m, 1H), 5.63 (s, 1H), 3.81 (d, *J* = 10 Hz, 1H), 3.45 (dd, *J* = 2.0, 1.0 Hz, 1H), 3.35 (m, 1H), 3.24 (m, 1H), 3.20 (d, *J* = 2.0 Hz, 1H), 3.05 (qd, *J* = 13.5, 7.6 Hz, 2H), 2.68 (m, 1H), 2.53 (s, 1H), 2.25 (t, *J* = 10.0 Hz, 1H), 2.16 (s, 3H), 2.11 (dd, *J* = 12.1, 6.1 Hz, 1H), 1.67 (s, 3H), 1.54 (s, 3H), 1.42 (s, 3H), 1.21 (d, *J* = 6.7 Hz, 3H). ^{13}C NMR (125 MHz, $CDCl_3$) δ 215.4, 174.2, 170.3, 137.6, 134.1, 131.7, 131.4, 129.2, 129.1, 127.2, 126.7, 76.4, 72.0, 68.2, 60.8, 60.0, 53.2, 51.5, 50.7, 49.3, 44.8, 42.0, 37.5, 22.0, 21,0, 19.4, 17.3, 14.1.

For NMR spectral data in MeOH-*d₄*, see Table S3.

Deacetyl-19,20-epoxycytochalasin C (4):

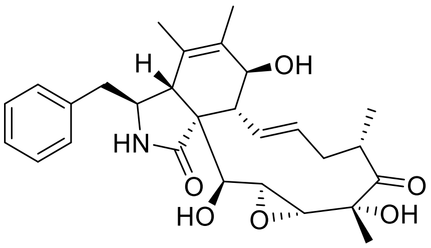

ESI-HRMS $[M+H]^+$ *m/z* 482.2531 (calcd for $C_{28}H_{36}NO_6$, 482.2537). ^1H NMR (500 MHz, $CDCl_3$) δ 7.33 (t, *J* = 7.3 Hz, 2H), 7.27 (m, 1H), 7.14 (d, *J* = 6.8 Hz, 2H), 6.11 (dd, *J* = 15.6, 10.3 Hz, 1H), 5.66 (ddd, *J* = 15.6, 10.0, 6.0 Hz, 1H), 5.56 (s, 1H), 4.17 (s, 1H), 3.77 (d, *J* = 10.1 Hz, 1H), 3.42 (s, 1H), 3.35 (m, 1H), 3.21 (m, 1H), 3.00 (dd, *J* = 13.3, 5.1 Hz, 1H), 2.89 (s, 1H), 2.81 (dd, *J* = 13.3, 9.7 Hz, 1H), 2.63 (m, 1H), 2.40 (t, *J* = 10.1 Hz, 1H), 2.17 (s, 1H), 2.09 (dd, *J* = 12.6, 5.9 Hz, 1H), 1.69 (m, 3H), 1.56 (s, 6H), 1.20 (d, *J* = 6.7 Hz, 3H). ^{13}C NMR (125 MHz, $CDCl_3$) δ 215.5, 175.3, 137.4, 133.9, 131.7, 131.4, 129.2, 129.2, 127.4, 126.8, 76.6, 72.8, 68.1, 60.8, 60.8, 55.4, 51.8, 51.7, 47.8, 45.7, 42.0, 37.7, 22.0, 19.3, 17.5, 14.0.

18-desoxy-19,20-epoxycytochalasin C (5):

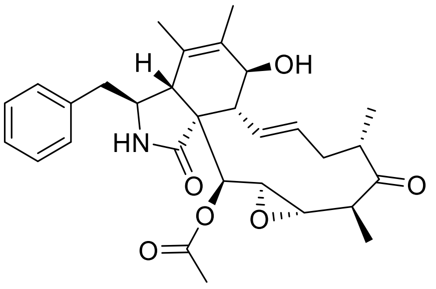

ESI-HRMS $[M+H]^+$ *m/z* 508.2688 (calcd for $C_{30}H_{38}NO_6$, 508.2694). ^1H NMR (500 MHz, $CDCl_3$) δ 7.32 (m, 2H), 7.25 (m, 1H), 7.21 (m, 2H), 6.37(m, 1H), 5.64 (m, 2H), 3.80 (m, 1H), 3.44 (m, 1H), 3.34 (m, 1H), 3.02 (m, 1H), 2.98 (m, 1H), 2.96 (m, 1H), 2.55 (m, 1H), 2.45 (m, 1H), 2.28 (m, 1H), 2.18 (m, 1H), 2.15 (m, 3H), 1.68 (s, 3H), 1.40 (s, 3H), 1.32 (d, *J* = 6.7 Hz, 3H), 1.14 (d, *J* = 6.7 Hz, 3H). ^{13}C NMR (125 MHz, $CDCl_3$) δ 215.9, 174.7, 170.4, 137.6, 134.4, 131.5, 131.1, 129.2, 129.1, 127.2, 126.6, 72.3, 67.9, 60.8, 58.7, 57.8, 51.4, 51.1, 50.3, 49.3, 44.7, 43.5, 37.6, 29.8, 20.9, 18.9, 17.4, 14.9, 14.1.

19,20-epoxycytochalasin Q (**6**):

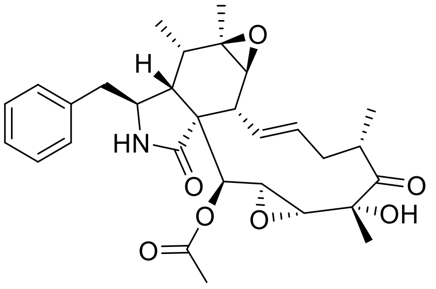

ESI-HRMS $[M+H]^+$ *m/z* 524.2640 (calcd for $C_{30}H_{38}NO_7$, 524.2643). ^1H NMR (500 MHz, $CDCl_3$) δ 7.34 (t, *J* = 7.4 Hz, 2H), 7.26 (t, *J* = 7.4 Hz, 1H), 7.20 (d, *J* = 7.1 Hz, 2H), 6.02 (dd, *J* = 15.5, 10.0 Hz, 1H), 5.83 (s, 1H), 5.64 (m, 2H), 4.07 (s, 1H), 3.59 (t, *J* = 6.9 Hz, 1H), 3.52 (s, 1H), 3.21 (dtt, *J* = 13.4, 6.7, 3.3 Hz, 1H), 3.16 (d, *J* = 1.9 Hz, 1H), 2.84 (d, *J* = 7.2 Hz, 2H), 2.72 (d, *J* = 5.7 Hz, 1H), 2.61 (q, *J* = 12.3 Hz, 1H), 2.33 (dd, *J* = 10.0, 5.7 Hz, 1H), 2.20 (dd, *J* = 5.6, 2.2 Hz, 1H), 2.12 (s, 3H), 2.08 (m, 1H), 1.61 (dt, *J* = 13.9, 7.2 Hz, 2H), 1.53 (s, 3H), 1.20 (d, *J* = 6.7 Hz, 3H), 1.17 (s, 3H), 0.83 (d, *J* = 7.3 Hz, 3H). ^{13}C NMR (125 MHz, $CDCl_3$) δ 215.6, 174.4, 170.0, 136.9, 131.5, 131.3, 129.4, 129.2, 127.3, 76.5, 72.8, 62.5, 60.0, 57.4, 54.6, 53.0, 51.5, 45.9, 44.9, 41.9, 37.7, 36.8, 22.0, 20.8, 19.8, 19.3, 12.6.

For assignments, see Tables S3 and S4.

Deacetyl-19,20-epoxycytochalasin Q (7):

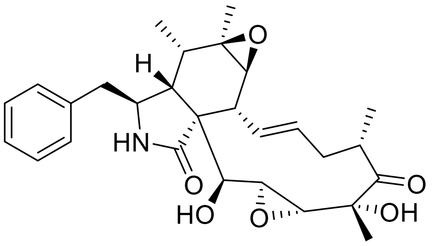

ESI-HRMS $[M+H]^+$ *m/z* 482.2534 (calcd for $C_{28}H_{36}NO_6$, 482.2537). ^1H NMR (500 MHz, $CDCl_3$) δ 7.35 (t, *J* = 7.3 Hz, 2H), 7.29 (t, *J* = 7.3 Hz, 1H), 7.14 (d, *J* = 7.0 Hz, 2H), 5.96 (dd, *J* = 15.5, 10.1 Hz, 1H), 5.66 (s, 1H), 5.57 (ddd, *J* = 15.5, 10.1, 5.8 Hz, 1H), 4.12 (s, 1H), 3.81 (s, 1H), 3.66 (dt, *J* = 7.6, 3.3 Hz, 1H), 3.43 (s, 1H), 3.35 (d, *J* = 2.1 Hz, 1H), 3.18 (dtt, *J* = 13.0, 6.3, 3.0 Hz, 1H), 2.86 (dd, *J* = 13.4, 4.6 Hz, 1H), 2.68 (d, *J* = 5.8 Hz, 1H), 2.65 (dd, *J* = 13.5, 8.7 Hz, 1H), 2.56 (q, *J* = 12.2 Hz, 1H), 2.45 (m, 2H), 2.06 (dd, *J* = 12.6, 5.5 Hz, 1H), 1.84 (dt, *J* = 12.6, 6.4 Hz, 1H), 1.55 (s, 3H), 1.22 (s, 3H), 1.18 (d, *J* = 6.7 Hz, 3H), 1.06 (d, *J* = 7.3 Hz, 3H). ^{13}C NMR (125 MHz, $CDCl_3$) δ 215.8, 175.5, 136.9, 131.6, 131.0, 129.4, 129.2, 127.5, 76.6, 73.1, 62.9, 60.8, 57.6, 55.2, 54.6, 54.5, 52.7, 46.1, 43.2, 42.0, 37.9, 36.7, 21.9, 20.0, 19.3, 13.0.

For assignments, see Table S3.

19,20-epoxycytochalasin R (**8**):

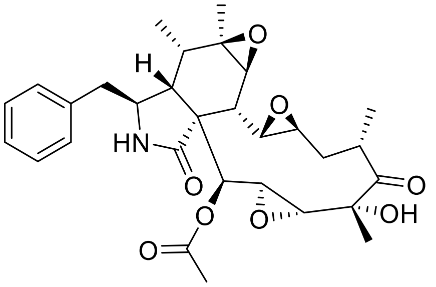

ESI-HRMS $[M+H]^+$ *m/z* 540.2587 (calcd for $C_{30}H_{38}NO_8$, 540.2592). ^1H NMR (700 MHz, $CDCl_3$) δ 7.36 (t, *J* = 7.5 Hz, 2H), 7.28 (t, *J* = 7.5 Hz, 1H), 7.20 (d, *J* = 7.5 Hz, 2H), 5.82 (s, 1H), 5.61 (s, 1H), 3.67 (m, 1H), 3.65 (m, 1H), 3.62 (d, *J* = 8.4 Hz, 1H), 3.35 (m, 1H), 3.24 (dq, *J* = 13.5, 6.8 Hz, 1H), 2.99 (d, *J* = 5.5 Hz, 1H), 2.86 (m, 3H), 2.25 (m, 2H), 2.08 (s, 3H), 1.77 (m, 1H), 1.60 (s, 3H), 1.52 (m, 1H), 1.40 (dd, *J* = 8.3, 5.7, 1H), 1.25 (d, *J* = 6.8 Hz, 3H), 1.22 (s, 3H), 0.87 (d, *J* = 7.3 Hz, 3H). ^{13}C NMR (175 MHz, $CDCl_3$) δ 214.4, 174.3, 169.6, 136.7, 129.4, 129.3, 127.5, 76.5, 73.1, 61.5, 59.8, 58.0, 57.6, 56.1, 55.1, 53.6, 52.8, 46,0, 43.9, 38.8, 37.7, 36.9, 22.0, 20.8, 20.0, 19.7, 12.7.

19,20-epoxycytochalasin D (9):

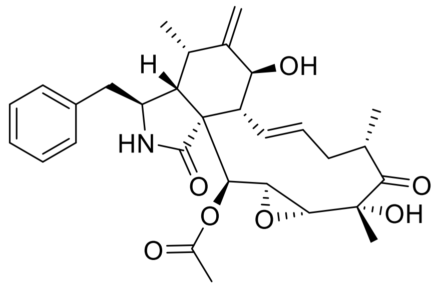

ESI-HRMS [M+H]$^+$ *m/z* 524.2637 (calcd for $C_{30}H_{38}NO_7$, 524.2643). ^{1}H NMR (500 MHz, $CDCl_3$) δ 7.33 (t, *J* = 7.4 Hz, 2H), 7.26 8m, 1H), 7.16 (d, *J* = 7.0 Hz, 2H), 5.90 (dd, *J* = 15.6, 9.8 Hz, 1H), 5.71 (ddd, *J* = 15.6, 9.9, 5.9 Hz, 1H), 5.59 (s, 1H), 5.53 (s, 1H), 5.29 (s, 1H), 5.08 (s, 1H), 3.83 (d, *J* = 10.2 Hz, 1H), 3.54 (m, 1H), 3.24 (m, 2H), 3.16 (d, J = 2.0 Hz, 1H), 2.86 (dd, *J* = 13.5, 5.0 Hz, 1H), 2.75 (dd, *J* = 13.4, 9.1 Hz, 2H), 2.67 (m, 1H), 2.64 (m, 2H), 2.26 (m, 1H), 2.16 (s, 3H), 2.11 (s, 1H), 2.03 (s, 1H), 1.54 (s, 3H), 1.21 (d, J = 6.7 Hz, 3H), 0.90 (d, J = 6.7 Hz, 3H). ^{13}C NMR (125 MHz, $CDCl_3$) δ 215.5, 173.7, 170.0, 147.4, 137.2, 133.7, 131.3, 129.3, 129.1, 127.3, 114.7, 76.5, 74.1, 70.1, 59.8, 54.1, 52.9, 52.1, 50.8, 46.6, 45.3, 42.0, 37.5, 32.7, 22.0, 20.9, 19.3, 13.6.

Table S3. ^{1}H and ^{13}C NMR (500/125 MHz) data of deacetyl-18-desoxy-19,20-epoxycytochalasin Q (**1**), deacetyl-19,20-epoxycytochalasin Q (**7**), and 19,20-epoxycytochalasin Q (**6**) in $CDCl_3$.

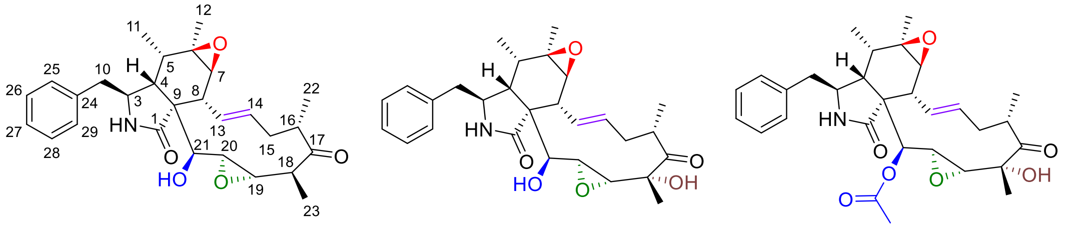

N°	Deacetyl-18-desoxy-19,20-epoxycytochalasin Q (**1**)		Deacetyl-19,20-epoxycytochalasin Q (**7**)		19,20-epoxycytochalasin Q (**6**)	
	δ_C	δ_H	δ_C	δ_H	δ_C	δ_H
1	175.9		175.5		174.4	
2		5.84, s		5.66, s		5.83, s
3	54.4	3.68, m	54.5	3.66, dt (7.6, 3.3)	54.6	3.59, t (6.9)
4	52.0	2.49, m	52.7	2.45, m	51.5	2.20, dd (5.6, 2.2)
5	36.7	1.84, m	36.7	1.84, dt (12.6, 6.4)	36.8	1.61, dt (13.9, 7.2)
6	57.6		57.6		57.4	
7	63.1	2.69 (d, 5.7)	62.9	2.68, d (5.8)	62.5	2.72, d (5.7)
8	43.1	2.45, m	43.2	2.45, m	44.9	2.33, dd (10.0, 5.7)
9	54.5		54.6		54.6	
10	46.0	2.88, dd (13.4, 4.6)<br>2.65, m	46.1	2.86, dd (13.4, 4.6)<br>2.65, dd (13.5, 8.7)	45.9	2.84, d (7.2)
11	13.1	1.06, d (7.3)	13.0	1.06, d (7.3)	12.6	0.83, d (7.3)
12	20.1	1.23, s	20.0	1.22, s	19.8	1.17, s
13	131.2	6.13, dd (15.4, 10.1)	131.6	5.96, dd (15.5, 10.1)	131.5	6.02, dd (15.5, 10.0)
14	131.2	5.52, ddd (15.4, 10.1, 5.1)	131.1	5.57, ddd (15.5, 10.1, 5.8)	131.3	5.64, m
15	37.9	2.45, m<br>2.07, m	37.9	2.56, d (12.2)<br>2.06, dd (12.6, 5.5)	36.8	2.61, q (12.3)<br>2.08, m
16	42.8	2.98, ddt (9.6, 6.8, 2.8)	42.0	3.18, dtt (13.0, 6.3, 3.0)	41.9	3.21, dtt (13.4, 6.7, 3.3)
17	216.0		215.8		215.6	
18	52.4	2.20, m	76.6		76.5	
19	59.3	3.13, dd (9.0, 1.7)	60.8	3.35, d (2.1)	60.0	3.16, d (1.9)
20	59.4	3.38, s	55.2	3.43, s	53.0	3.52, s
21	73.1	3.66, s	73.1	3.81, s	72.8	5.64, m
22	19.0	1.10, d (6.8)	19.3	1.18, d (6.7)	19.3	1.20, d (6.7)
23	14.6	1.32, d (6.8)	21.9	1.55, s	22.0	1.53, s
24	137.0		136.9		136.9	
25	129.5	7.16, d (7.2)	129.4	7.14, d (7.0)	129.4	7.20, t (7.1)
26	129.1	7.34, t (7.2)	129.2	7.35, t (7.3)	129.1	7.34, t (7.4)
27	127.5	7.29, m	127.5	7.29, t (7.3)	127.3	7.26, t (7.4)
28	129.1	7.34, t (7.2)	129.2	7.35, t (7.3)	129.1	7.34, t (7.4)
29	129.5	7.16, d (7.2)	129.4	7.14, d (7.0)	129.4	7.20, t (7.1)
30					170.0	
31					20.8	2.12, s
18-OH				4.12, s		4.07, s

4. Precursor-supplemented cultivation of *Xylaria* sp. XSP14

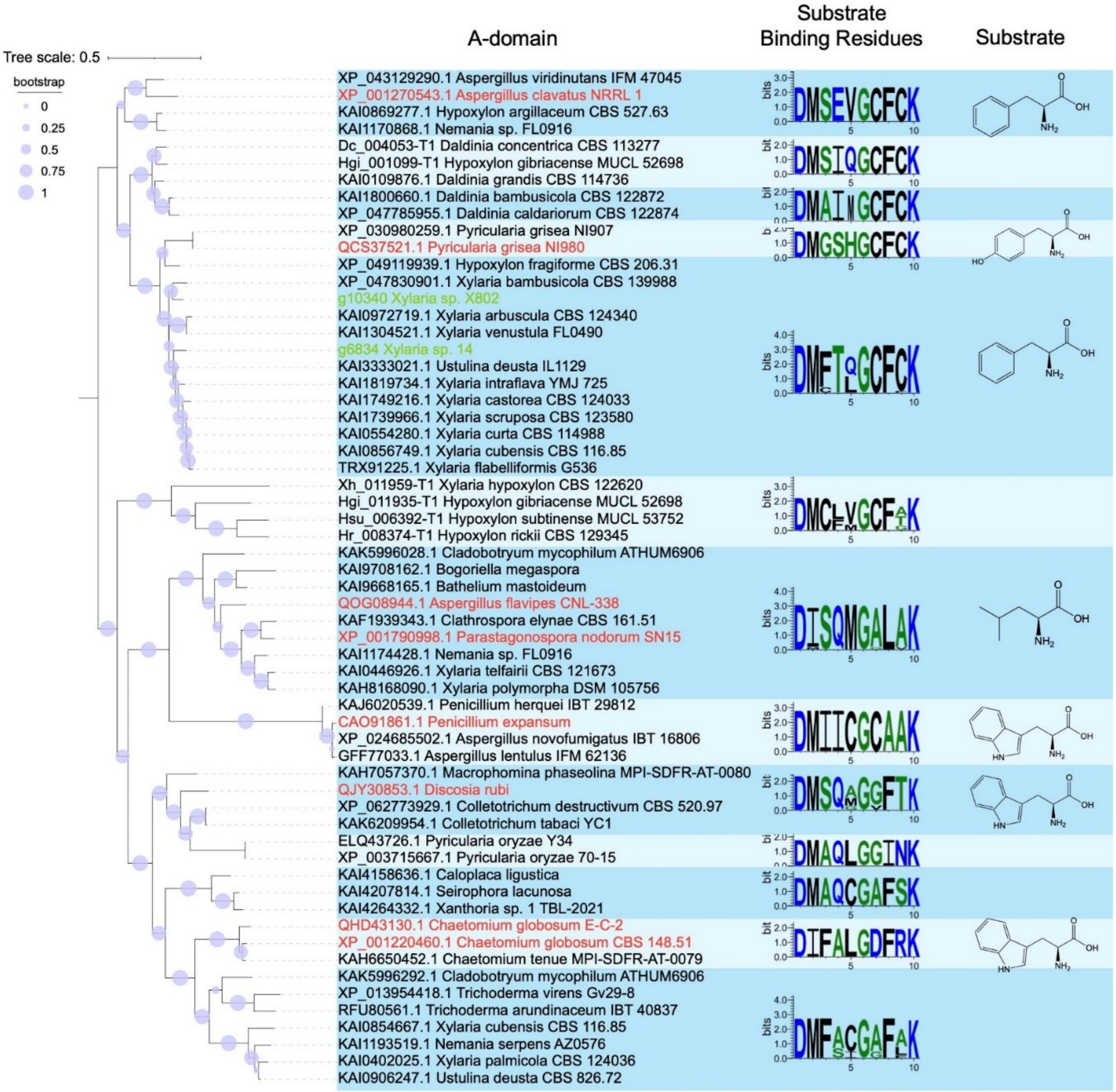

Figure S4. Phylogenetic analysis of adenylation domains from cytochalasan PKS-NRPS enzymes. The phylogram on the left shows a maximum likelihood tree constructed from the protein sequences of adenylation domains. Sequences from experimentally characterized enzymes are highlighted in red, with their known amino acid substrates indicated on the right. Sequences identified in this study are shown in green. Predicted substrate-binding residues are listed at the end of each sequence label. Adenylation domains are grouped based on phylogenetic relationships and similarity of their binding residues. For each group, a binding motif analysis was performed, and the frequency of amino acid usage at each binding position is shown in the middle column.

Cultivation of *Xylaria* sp. XSP14 and *Chaetomium globosum* CBS 148.51 on media supplemented with amino acids. A 50 mM stock solution of amino acids was prepared in ddH$_2$O or DMSO/H$_2$O (1:10, v:v) (Table S4), and sterilized by filtration through a 0.2 μm membrane. The final concentration of 2 mM, 10 mM and 20 mM were prepared from the stock solution in autoclaved PDA (10mL) in 6-well plates. *Xylaria* sp. XSP14 and *C. globosum* CBS 148.51 were first pre-cultivated on PDA for 10 days and then subcultured by inoculating modified PDA medium (supplemented with amino acids, 6-well plates) with an agar cube. The plates were kept at 25 °C in the dark for 3 weeks. The plates were then cut into pieces and soaked in MeOH overnight. The organic extract was filtered, dried under vacuum, dissolved in MeOH with a final concentration of 50 μg/mL, and analyzed by HRMS (Table S5 and Table S6).

Table S4. Preparation of amino acids stock solution for 6-well assay.

Amino acid	Amino acid abbreviation	Solvent	Concentration stock solution
L-phenylalanine	4-F-Phe	ddH$_2$O	50 mM
4-fluoro-L-phenylalanine	4-F-Phe	ddH$_2$O	50 mM
4-bromo-L-phenylalanine	4-Br-Phe	DMSO/H$_2$O (1:10, v:v)	50 mM
3-bromo-L-phenylalanine	3-Br-Phe	ddH$_2$O	50 mM
2-bromo-L-phenylalanine	2-Br-Phe	DMSO/H$_2$O (1:10, v:v)	50 mM
4-chloro-L-phenylalanine	4-Cl-Phe	DMSO/H$_2$O (1:10, v:v)	50 mM
3-chloro-L-phenylalanine	3-Cl-Phe	ddH$_2$O	50 mM
2-chloro-L-phenylalanine	2-Cl-Phe	ddH$_2$O	50 mM
4-iodo-L-phenylalanine	4-I-Phe	DMSO/H$_2$O (1:10, v:v)	50 mM
3-iodo-L-phenylalanine	3-I-Phe	DMSO/H$_2$O (1:10, v:v)	50 mM
2-iodo-L-phenylalanine	2-I-Phe	DMSO/H$_2$O (1:10, v:v)	50 mM
4-methoxy-L-phenylalanine	4-OMe-Phe	ddH$_2$O	50 mM
3-methoxy-L-phenylalanine	3-OMe-Phe	ddH$_2$O	50 mM
2-methoxy-L-phenylalanine	2-OMe-Phe	ddH$_2$O	50 mM
4-methyl-L-phenylalanine	4-Me-Phe	ddH$_2$O	50 mM
3-methyl-L-phenylalanine	3-Me-Phe	ddH$_2$O	50 mM
2-methyl-L-phenylalanine	2-Me-Phe	ddH$_2$O	50 mM
4-ethyl-L-phenylalanine	4-Et-Phe	DMSO/H$_2$O (1:10, v:v)	50 mM
4-phenyl-L-phenylalanine	4-Phe-Phe	DMSO/H$_2$O (1:10, v:v)	50 mM
4-acetyl-L-phenylalanine	4-Ac-Phe	DMSO/H$_2$O (1:10, v:v)	50 mM
4-amino-L-phenylalanine	4-NH$_2$-Phe	ddH$_2$O	50 mM
4-nitro-L-phenylalanine	4-NO$_2$-Phe	DMSO/H$_2$O (1:10, v:v)	50 mM
4-azido-L-phenylalanine	4-azido-Phe	DMSO/H$_2$O (1:10, v:v)	50 mM
3-hydroxy-L-phenylalanine	3-OH-Phe	DMSO/H$_2$O (1:10, v:v)	50 mM
3-cyano-L-phenylalanine	3-CN-Phe	DMSO/H$_2$O (1:10, v:v)	50 mM
L-tyrosine	Tyr	ddH$_2$O	50 mM
3-fluoro-L-tyrosine	3-F-Tyr	ddH$_2$O	50 mM
3-bromo-L-tyrosine	3-Br-Tyr	ddH$_2$O	50 mM
O-propargyl-L-tyrosine	*O*-propargyl-Tyr	ddH$_2$O	50 mM
L-leucine	Leu	ddH$_2$O	50 mM
L-histidine	His	ddH$_2$O	50 mM
L-tryptophan	Tryp	ddH$_2$O	50 mM
5-bromo-L-tryptophan	5-Br-Tryp	ddH$_2$O	50 mM
5-methyl-L-tryptophan	5-Me-Tryp	ddH$_2$O	50 mM

Table S5. Percentage of derivative produced relative to major chaetoglobosin produced by *Chaetomium globosum* CBS 148.51 (n.d. = below detection limit).

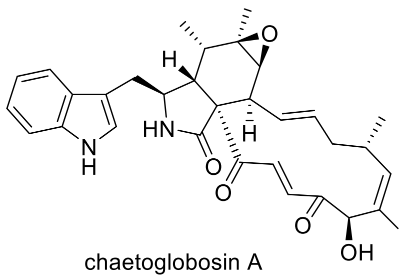

chaetoglobosin A
major chaetoglobosin

Amino acid	2 mM	10 mM	20 mM
L-phenylalanine	n.d.	n.d.	n.d.
3-bromo-L-phenylalanine	n.d.	n.d.	n.d.
2-bromo-L-phenylalanine	n.d.	n.d.	n.d.
4-chloro-L-phenylalanine	n.d.	n.d.	n.d.
3-chloro-L-phenylalanine	n.d.	n.d.	n.d.
2-chloro-L-phenylalanine	n.d.	n.d.	n.d.
4-phenyl-L-phenylalanine	n.d.	n.d.	n.d.
4-amino-L-phenylalanine	n.d.	n.d.	n.d.
4-azido-L-phenylalanine	n.d.	n.d.	n.d.
3-fluoro-L-tyrosine	n.d.	n.d.	n.d.
3-bromo-L-tyrosine	n.d.	n.d.	n.d.
O-propargyl-L-tyrosine	n.d.	n.d.	n.d.
L-histidine	n.d.	n.d.	n.d.
5-bromo-L-tryptophan	<1%	3%	n.d.
5-methyl-L-tryptophan	<1%	2%	n.d.

Table S6. Percentage of derivative produced relative to major cytochalasin **1** / major cytochalasin **2** produced by *Xylaria* sp. XSP14 (n.d. = below detection limit).

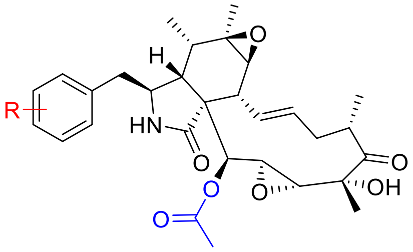

19,20-epoxycytochalasin Q
major cytochalasin 1

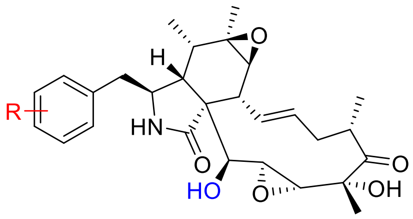

deacetyl-19,20-epoxycytochalasin Q
major cytochalasin 2

**Amino acid**		**2 mM**	**10 mM**	**20 mM**
L-phenylalanine	4-fluoro	4% / n.d.	4% / n.d.	4% / n.d.
	4-bromo	n.d. / n.d.	n.d. / n.d.	n.d. / n.d.
	3-bromo	8% / 24%	26% / 100%	34% / 200%
	2-bromo	n.d. / n.d.	n.d. / n.d.	n.d. / n.d.
	4-chloro	<1% / 5%	<1% / 5%	<1% / 5%
	3-chloro	50% / 66%	160% / 280%	340% / 760%
	2-chloro	n.d. / <1%	n.d. / 15%	n.d. / 20%
	4-iodo	n.d. / n.d.	n.d. / n.d.	n.d. / n.d.
	3-iodo	<1% / <1%	<1% / <1%	<1% / <1%
	2-iodo	n.d. / n.d.	n.d. / n.d.	n.d. / n.d.
	4-methoxy	n.d. / n.d.	n.d. / n.d.	n.d. / n.d.
	3-methoxy	n.d. / 31%	co-elution / 57%	co-elution / 85%
	2-methoxy	n.d. / n.d.	n.d. / n.d.	n.d. / n.d.
	4-methyl	<1% / <1%	<1% / 10%	<1% / 11%
	3-methyl	110% / 570%	410% / 3 800%	390% / 4 600%
	2-methyl	n.d. / <1%	n.d. / 1%	n.d. / <1%
	4-ethyl	n.d. / n.d.	n.d. / n.d.	n.d. / n.d.
	4-phenyl	n.d. / n.d.	n.d. / n.d.	n.d. / n.d.
	4-acetyl	n.d. / n.d.	n.d. / n.d.	n.d. / n.d.
	4-amino	n.d. / n.d.	n.d. / n.d.	n.d. / n.d.
	4-nitro	n.d. / n.d.	n.d. / n.d.	n.d. / n.d.
	4-azido	n.d. / n.d.	n.d. / n.d.	n.d. / n.d.
	3-hydroxy	n.d. / n.d.	n.d. / n.d.	n.d. / n.d.
	3-cyano	8% / n.d.	12% / 170%	13% / 300%
L-tyrosine		n.d. / n.d.	n.d. / n.d.	n.d. / n.d.
3-fluoro-L-tyrosine		2% / 20%	3% / 30%	3% / 40%
3-bromo-L-tyrosine		n.d. / n.d.	n.d. / n.d.	n.d. / n.d.
O-propargyl-L-tyrosine		n.d. / n.d.	n.d. / n.d.	n.d. / n.d.
L-leucine		n.d. / n.d.	n.d. / n.d.	n.d. / n.d.
L-tryptophan		n.d. / n.d.	n.d. / n.d.	n.d. / n.d.

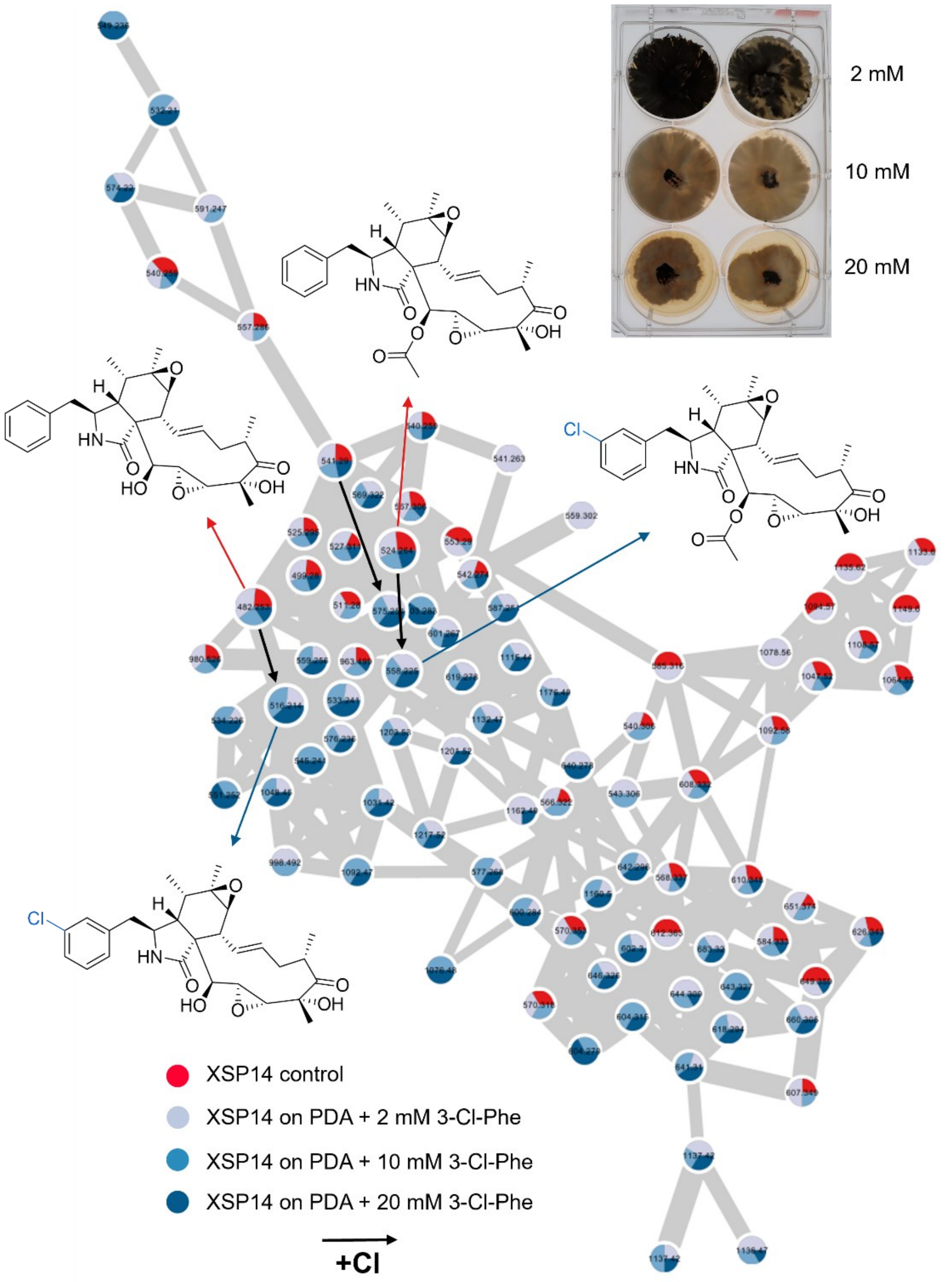

Figure S5. Precursor-directed diversification using 3-chloro-L-phenylalanine. Picture of 6-well plate assay with *Xylaria* sp. XSP14 on PDA supplemented with 2, 10 and 20 mM 3-chloro-L-phenylalanine. Expanded GNPS molecular network cluster of cytochalasans detected in methanolic extracts of from *Xylaria* sp. XSP14 cultivated on PDA and PDA supplemented with 2, 10 pr 20 mM.3-chloro-L-phenylalanine. Data set of the molecular network has been submitted to MassIVE server: MSV000098575.

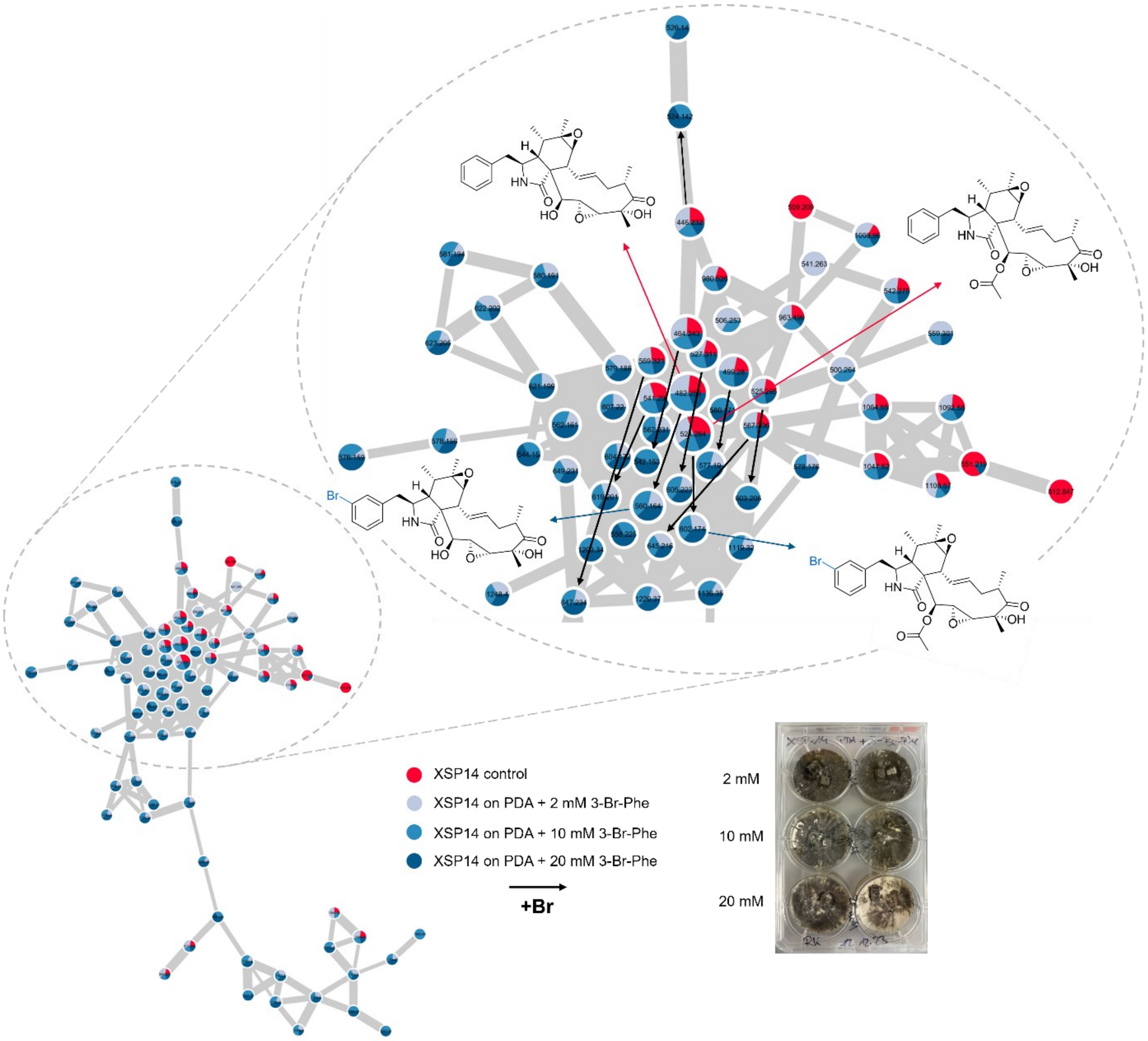

Figure S6. Precursor-directed diversification using 3-bromo-L-phenylalanine. Picture of 6-well plates of *Xylaria* sp. XSP14 on PDA supplemented with 3-bromo-L-phenylalanine at 2, 10 and 20 mM and expanded GNPS molecular network cluster of cytochalasans from *Xylaria* sp. XSP14 extracts cultivated on PDA and PDA supplemented with 2, 10 or 20 mM 3-bromo-L-phenylalanine. Data set of the molecular network has been submitted to MassIVE server: MSV000098576.

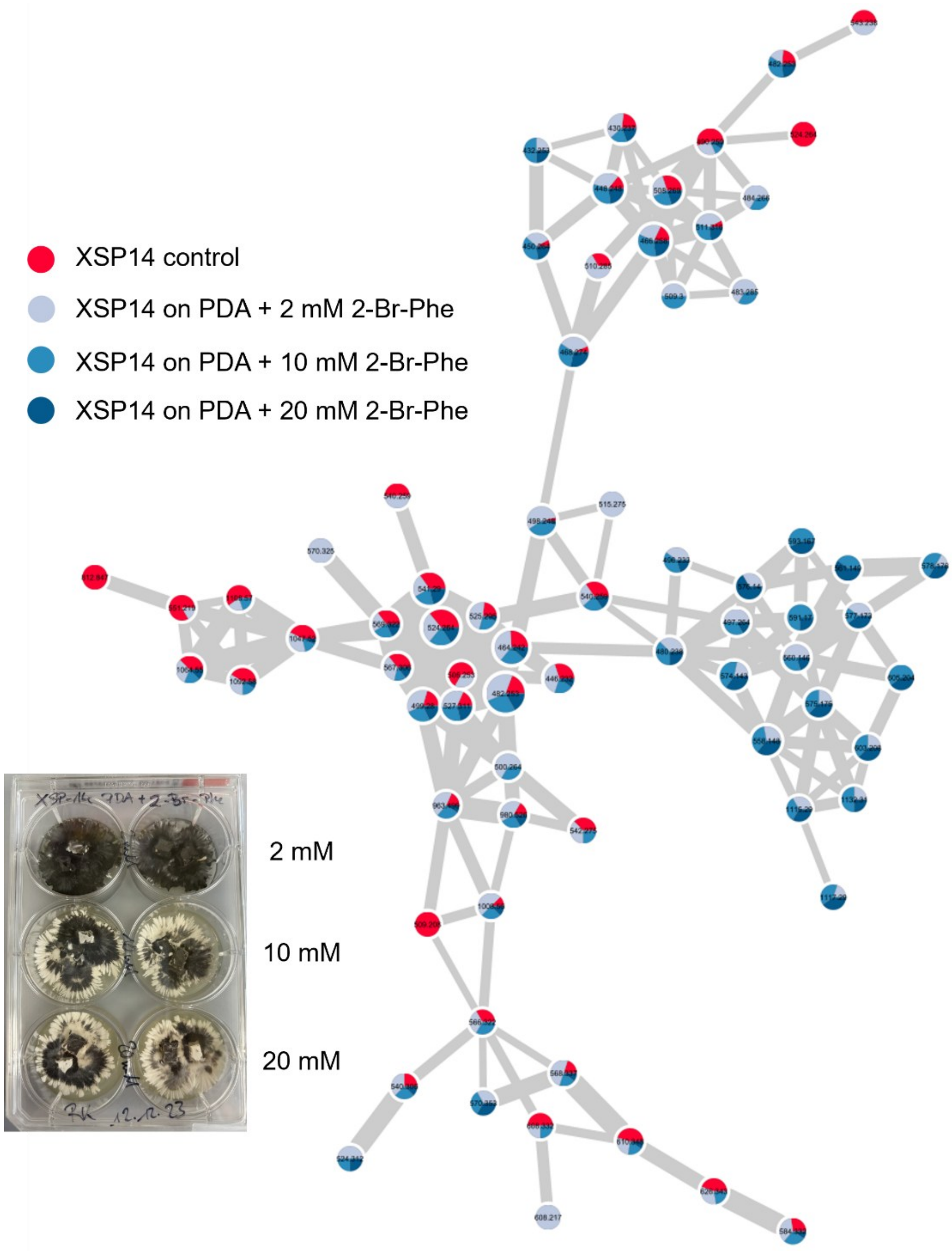

Figure S7. Precursor-directed diversification using 2-bromo-L-phenylalanine. 6-well plates of *Xylaria* sp. XSP14 on PDA supplemented with 2-bromo-L-phenylalanine at 2, 10 and 20 mM and expanded GNPS molecular network cluster of cytochalasans from *Xylaria* sp. XSP14 extracts cultivated on PDA and PDA supplemented with 2, 10 or 20 mM 2-bromo-L-phenylalanine. Data set of the molecular network has been submitted to MassIVE server: MSV000098577.

Preparative cultivation of *Xylaria* sp. XSP14 on supplemented media with 3-chloro-phenylalanine: A total of five PDA plates (15 mL PDA, 92x16 mm) of a 2-week-old *Xylaria* sp. XSP14 culture were used to inoculate 100 PDA plates (15 mL PDA, 92x16 mm) media supplemented with 3-chloro-phenylalanine at a concentration of 20 mM, which were kept at 25 °C for 3 weeks in the dark. Agar plates were then cut into pieces, soaked in MeOH overnight. The organic extract was filtered and dried under vacuum. The crude extract (8.2 g) was submitted to SPE-C18 open column purification by step-wise elution using H_2O/MeOH mixtures. In total, 5 fractions were obtained. Fraction 4 (820 mg) was subjected to preparative reverse-phase Flash chromatography (Phenomenex Luna C18(2) 250 x 30 mm, 5 µm, 100 Å column) using the following gradient: 0-5 min, 25% B; 5-60 min, 25%-100% B; 60-70 min, 100% B (A: H_2O; B: CH_3CN) with a flow rate of 20 mL/min to yield deacetyl-19,20-epoxycytochalasin C (**4**, 1.8 mg), *m*-chloro-deacetyl-19,20-epoxycytochalasin C (**12**, 1.3 mg), *m*-chloro-deacetyl-19,20-epoxycytochalasin Q (**15**, 17 mg), 19,20-epoxycytochalasin C (**3**, 6.8 mg), *m*-chloro-19,20-epoxycytochalasin C (**11**, 2.2 mg), *m*-chloro-19,20-epoxycytochalasin Q (**14**, 52 mg), and *m*-chloro-18-desoxy-19,20-epoxycytochalasin Q (**16**, 1.7 mg). Fraction 5 (645 mg) was subjected to semi-preparative reverse-phase HPLC (Phenomenex Luna C18(2) 250 x 10 mm, 5 µm, 100 Å column) using the following gradient: 0-5 min, 30% B; 5-40 min, 30%-75% B; 40-43 min, 75%-100% B; 43-50 min, 100% (A: H_2O; B: CH_3CN) with a flow rate of 3 mL/min to yield *m*-chloro-deacetyl-19,20-epoxycytochalasin C (**12**, 40 mg), *m*-chloro-deacetyl-19,20-epoxycytochalasin Q (**15**, 49 mg), 19,20-epoxycytochalasin C (**3**, 14.5 mg), 19,20-epoxycytochalasin Q (**6**, 23.4 mg), *m*-chloro-19,20-epoxycytochalasin C (**11**, 35.3 mg), *m*-chloro-19,20-epoxycytochalasin Q (**14**, 105.8 mg), and *m*-chloro-cytochalasin Q (**13**, 7.8 mg).

Preparative cultivation of *Xylaria* sp. XSP14 on supplemented media with 3-bromo-phenylalanine: A total of five PDA plates (15 mL PDA, 92x16 mm) of a 2-week-old *Xylaria* sp. XSP14 culture were used to inoculate 100 PDA plates (15 mL PDA, 92x16 mm) media supplemented with 3-bromo-phenylalanine at a concentration of 20 mM, which were kept at 25 °C for 3 weeks in the dark. Agar plates were then cut into pieces, soaked in MeOH overnight. The organic extract was filtered and dried under vacuum. The crude extract (7.6 g) was submitted to SPE-C18 open column purification by step-wise elution using H_2O/MeOH mixtures. In total, 5 fractions were obtained. Fraction 4 (633 mg) was subjected to preparative reverse-phase Flash chromatography (Phenomenex Luna C18(2) 250 x 30 mm, 5 µm, 100 Å column) using the following gradient: 0-5 min, 25% B; 5-45 min, 25%-70% B; 45-55 min, 100% B (A: H_2O; B: CH_3CN) with a flow rate of 20 mL/min to yield deacetyl-19,20-epoxycytochalasin Q (**7**, 4.3 mg), *m*-bromo-deacetyl-19,20-epoxycytochalasin C (**17**, 4.5 mg), *m*-bromo-deacetyl-19,20-epoxycytochalasin Q (**19**, 148 mg), 19,20-epoxycytochalasin Q (21.7 mg), and *m*-

bromo-19,20-epoxycytochalasin Q (**18**, 13 mg). Fraction 5 (657 mg) was subjected to preparative reverse-phase Flash chromatography (Phenomenex Luna C18(2) 250 x 30 mm, 5 µm, 100 Å column) using the following gradient: 0-5 min, 30% B; 5-45 min, 30%-70% B; 45-55 min, 100% B (A: H_2O; B: CH_3CN) with a flow rate of 20 mL/min to yield deacetyl-19,20-epoxycytochalasin Q (**7**, 7.7 mg), *m*-bromo-deacetyl-19,20-epoxycytochalasin Q (**19**, 54.6 mg), 19,20-epoxycytochalasin Q (**6**, 15.8 mg), and *m*-bromo-19,20-epoxycytochalasin Q (**18**, 34 mg).

Preparative cultivation of *Xylaria* sp. XSP14 on supplemented media with 2-bromo-phenylalanine: A total of five PDA plates (15 mL PDA, 92x16 mm) of a 2-week-old *Xylaria* sp. XSP14 culture were used to inoculate 100 PDA plates (15 mL PDA, 92x16 mm) media supplemented with 2-bromo-phenylalanine at a concentration of 20 mM, which were kept at 25 °C for 3 weeks in the dark. Agar plates were then cut into pieces, soaked in MeOH overnight. The organic extract was filtered and dried under vacuum. The crude extract (6.5 g) was submitted to SPE-C18 open column purification by step-wise elution using H_2O/MeOH mixtures. In total, 5 fractions were obtained. Fraction 4 (427 mg) was subjected to preparative reverse-phase Flash chromatography (Phenomenex Luna C18(2) 250 x 30 mm, 5 µm, 100 Å column) using the following gradient: 0-5 min, 25% B; 5-45 min, 25%-70% B; 45-55 min, 100% B (A: H_2O; B: CH_3CN) with a flow rate of 20 mL/min to yield 19,20-epoxycytochalasin D (**9**, 4.4 mg), *o*-bromo-19-hydroxy-21-oxocytochalasin Q (**21**, 4.5 mg), 19,20-epoxycytochalasin C (**3**, 9.2 mg), and 19,20-epoxycytochalasin Q (**6**, 51 mg). Fraction 5 (331 mg) was subjected to preparative reverse-phase Flash chromatography (Phenomenex Luna C18(2) 250 x 30 mm, 5 µm, 100 Å column) using the following gradient: 0-5 min, 30% B; 5-45 min, 30%-70% B; 45-55 min, 100% B (A: H_2O; B: CH_3CN) with a flow rate of 20 mL/min to yield deacetyl-19,20-epoxycytochalasin Q (**7**, 1.7 mg), 19,20-epoxycytochalasin C (**3**, 1.1 mg), 19,20-epoxycytochalasin Q (**6**, 8.9 mg), and *o*-bromo-19,20-epoxy-21-oxocytochalasin Q (**20**, 3.7 mg).

_m_-Chloro-19,20-epoxycytochalasin C (**11**):

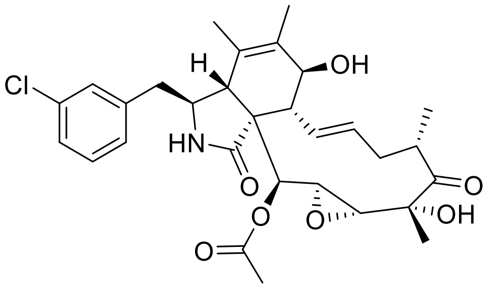

$[\alpha]^{20}_{D}$ = +36.9 (c 1.0, MeOH). ESI-HRMS $[M+H]^+$ *m/z* 558.2251 (calcd for $C_{30}H_{36}ClNO_7$, 558.2253).

NMR spectral data, see Table S7.

_m_-Chloro-deacetyl-19,20-epoxycytochalasin C (**12**):

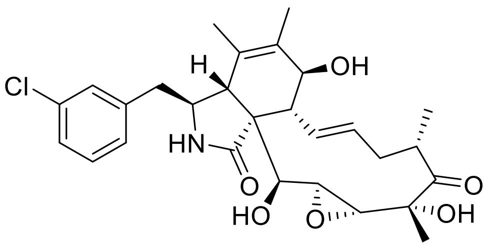

$[\alpha]^{20}_{D}$ = −1.6 (c 4.1, MeOH). ESI-HRMS $[M+H]^+$ *m/z* 516.2143 (calcd for $C_{28}H_{35}ClNO_6$, 516.2147).

NMR spectral data, see Table S7.

_m_-Chloro-cytochalasin Q (**13**):

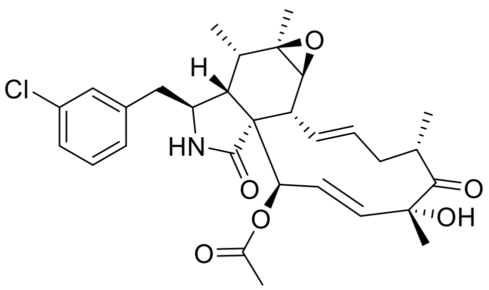

$[\alpha]^{20}_{D}$ = -3.5 (c 0.8, MeOH). ESI-HRMS $[M-H_2O+H]^+$ *m/z* 524.2196 (calcd for $C_{30}H_{37}ClNO_6$, 524.2198).

NMR spectral data, see Table S7.

***m*-Chloro-19,20-epoxycytochalasin Q** (**14**):

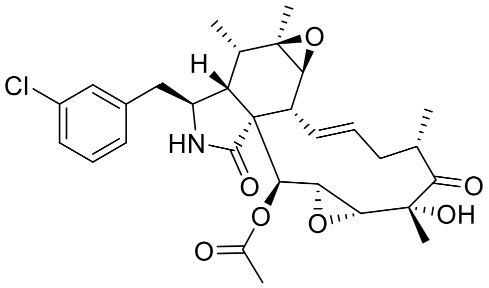

$[\alpha]^{20}_{D}$ = +29.7 (c 3.0, MeOH). ESI-HRMS $[M+H]^+$ *m/z* 558.2251 (calcd for $C_{30}H_{36}ClNO_7$, 558.2252).

NMR spectral data, see Table S8.

***m*-Chloro-deacetyl-19,20-epoxycytochalasin Q (15)**:

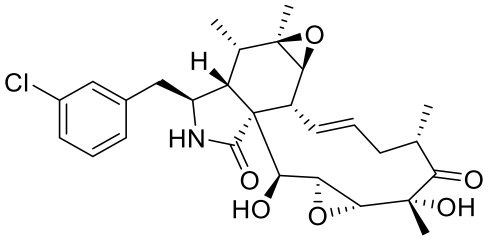

$[\alpha]^{20}_{D}$ = +1.0 (c 4.9, MeOH). ESI-HRMS $[M+H]^+$ *m/z* 516.2143 (calcd for $C_{28}H_{35}ClNO_6$, 516.2144).

NMR spectral data, see Table S8.

***m*-Chloro-18-desoxy-19,20-epoxycytochalasin Q (16):**

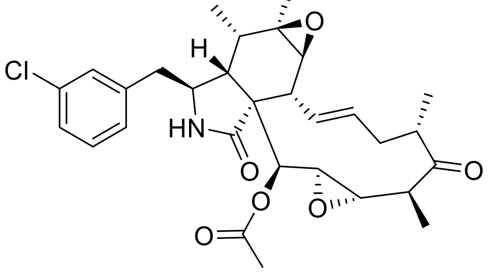

$[\alpha]^{20}_{D}$ = -2.6 (c 0.2, MeOH). ESI-HRMS $[M+H]^+$ *m/z* 542.2302 (calcd for $C_{30}H_{37}ClNO_6$, 542.2304).

NMR spectral data, see Table S8.

***m*-Bromo-deacetyl-19,20-epoxycytochalasin C (17):**

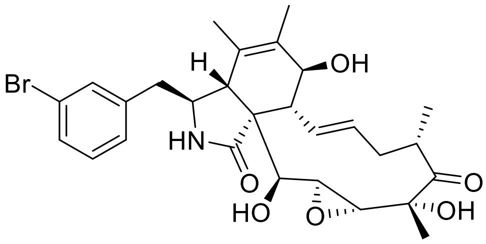

$[\alpha]^{20}_{D}$ = +12.2 (c 0.5, MeOH). ESI-HRMS $[M+H]^+$ *m/z* 560.1639 (calcd for $C_{28}H_{35}BrNO_6$, 560.1642).

NMR spectral data, see Table S9.

***m*-Bromo-19,20-epoxycytochalasin Q (18):**

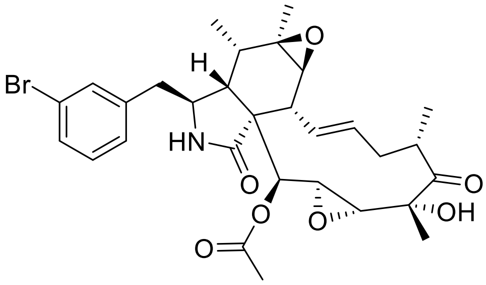

$[\alpha]^{20}_{D}$ = +27.0 (c 3.4, MeOH). ESI-HRMS $[M+H]^+$ *m/z* 602.1741 (calcd for $C_{30}H_{37}BrNO_7$, 602.1748).

NMR spectral data, see Table S9.

***m*-Bromo-deacetyl-19,20-epoxycytochalasin Q (19):**

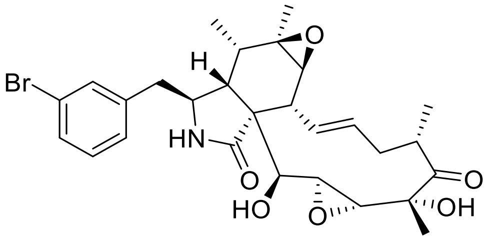

$[\alpha]^{20}_{D}$ = +4.5 (c 5.5, MeOH). ESI-HRMS $[M+H]^+$ *m/z* 560.1637 (calcd for $C_{28}H_{35}BrNO_6$, 560.1642).

NMR spectral data, see Table S9.

_o_-Bromo-19,20-epoxy-21-oxocytochalasin Q (20):

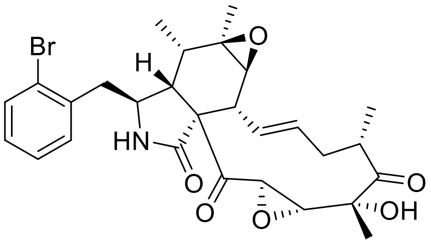

$[\alpha]^{20}_{D}$ = -12.7 (c 0.4, MeOH). ESI-HRMS $[M+H]^+$ *m/z* 558.1481 (calcd for $C_{28}H_{33}BrNO_6$, 558.1486).

NMR spectral data, see Table S10.

_o_-Bromo-19-hydroxy-21-oxocytochalasin Q (21):

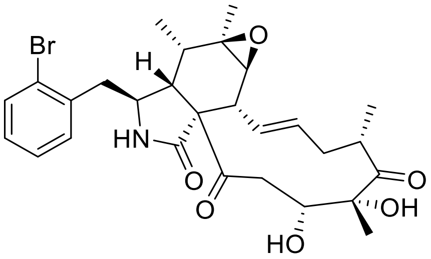

$[\alpha]^{20}_{D}$ = +2.9 (c 0.7, MeOH). ESI-HRMS $[M+H]^+$ *m/z* 560.1638 (calcd for $C_{28}H_{35}BrNO_6$, 560.1642).

NMR spectral data, see Table S10.

Table S7. ^{1}H and ^{13}C NMR (500/125 MHz) data of *m*-chloro-19,20-epoxycytochalasin C (**11**), *m*-chloro-deacetyl-19,20-epoxycytochalasin C (**12**), and *m*-chloro-cytochalasin Q (**13**) in $CDCl_3$.

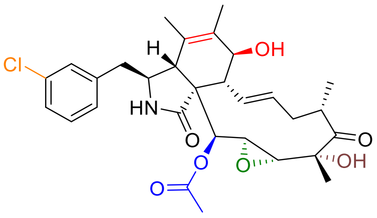

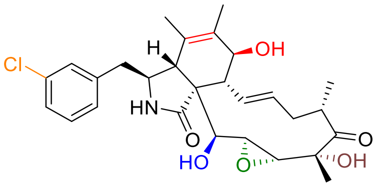

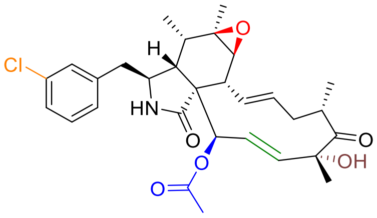

N°	*m*-chloro-19,20-epoxycytochalasin C (**11**)		*m*-chloro-deacetyl-19,20-epoxycytochalasin C (**12**)		*m*-chloro-cytochalasin Q (**13**)	
	δ_C	δ_H	δ_C	δ_H	δ_C	δ_H
1	174.5		175.5		174.7	
2		5.90, s		5.76, s		5.88, s
3	60.5	3.36, t (7.4)	60.8	3.35, t (7.3)	53.9	3.56, t (6.9)
4	50.6	2.53, s	51.8	2.88, s	50.6	2.09, d (4.0)
5	126.4		126.5		36.9	1.70, dt (13.4, 6.9)
6	131.8		131.8		57.1	
7	68.1	3.82, d (9.9)	68.1	3.77, d (10.0)	62.6	2.73, d (5.7)
8	49.3	2.27, t (9.9)	47.8	2.41, t (10.0)	45.2	2.52, dd (9.9, 5.5)
9	51.6		51.9		55.2	
10	44.3	3.04, m	45.3	2.97, dd (13.3, 5.1)<br>2.81, dd (13.3, 9.8)	45.5	2.78, m
11	14.1	1.69, s	14.0	1.70, s	12.7	0.85, d (7.2)
12	17.3	1.42, s	17.5	1.56, s	19.7	1.18, s
13	131.6	6.14, dd (15.6, 10.3)	131.7	6.09, dd (15.5, 10.0)	130.9	5.83, m
14	134.1	5.75, m	134.0	5.66, ddd (15.5, 10.0, 5.9)	132.0	5.26, td (10.6, 5.4)
15	37.5	2.69, q (12.2)<br>2.13, dd (11.5, 7.1)	37.7	2.63, q (12.1)<br>2.10, d (5.3)	38.0	2.46, m<br>2.00, dd (12.8, 5.0)
16	42.0	3.26, m	42.0	3.21, dd (11.7, 6.4)	42.4	2.73, d (5.7)
17	215.4		215.5		210.6	
18	76.5		76.6		77.9	
19	60.0	3.22, d (1.5)	60.6	3.42, s	131.9	6.07, dd (15.7, 2.0)
20	53.1	3.44, s	55.4	3.42, s	127.9	5.12, dd (15.7, 2.0)
21	72.0	5.75, s	72.9	4.19, s	75.8	5.78, s
22	19.3	1.23, d (6.7)	19.3	1.20, d (7.2)	19.5	1.19, d (6.9)
23	22.0	1.56, s	22.0	1.53, s	24.4	1.50, s
24	139.6		139.5		139.1	
25	127.4	7.26, m	127.6	7.27, m	127.6	7.27, s
26	134.8		135.0		134.9	
27	130.3	7.26, m	130.4	7.27, m	130.4	7.27, m
28	129.3	7.26, m	129.2	7.15, m	129.4	7.17, s
29	127.7	7.15, d (7.1)	127.4	7.03, d (6.6)	127.6	7.06, d (6.7)
30	170.3				170.0	
31	20.9	2.17, s			20.9	2.23, s
18-OH		4.09		4.12, s		4.65, s

Table S8. ^{1}H and ^{13}C NMR (500/125 MHz) data of *m*-chloro-19,20-epoxycytochalasin Q (**14**), *m*-chloro-deacetyl-19,20-epoxycytochalasin Q (**15**), and *m*-chloro-18-desoxy-19,20-epoxycytochalasin Q (**16**) in $CDCl_3$.

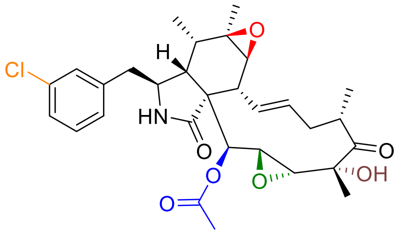
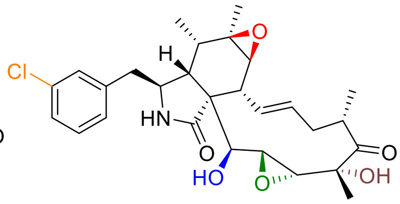
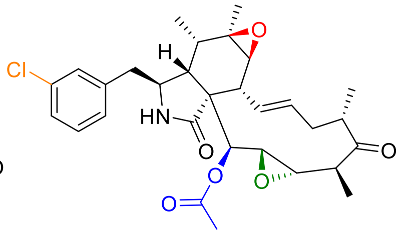

N°	*m*-chloro-19,20-epoxycytochalasin Q (**14**)		*m*-chloro-deacetyl-19,20-epoxycytochalasin Q (**15**)		*m*-chloro-18-desoxy-19,20-epoxycytochalasin Q (**16**)	
	δ_C	δ_H	δ_C	δ_H	δ_C	δ_H
1	174.7		176.05		174.7	
2		6.58, m		6.23, s		5.74, s
3	54.4	3.62, t (6.8)	54.4	3.64, m	54.2	3.57, dt (7.1, 3.5)
4	50.9	2.19, m	52.7	2.43, m	51.0	2.10, dd (5.5, 2.6)
5	36.7	1.60, m	36.7	1.83, p (6.8)	36.8	1.63, m
6	57.4		57.6		57.5	
7	62.5	2.73, d (5.5)	62.9	2.65, d (5.7)	62.6	2.74, d (5.6)
8	44.8	2.34, dd (9.7, 5.7)	43.3	2.46, m	44.7	2.37, dd (9.9, 5.6)
9	54.7		54.9		54.1	
10	45.1	2.93, dd (13.2, 6.8)<br>2.82, m	45.7	2.81, dd (13.1, 5.4)<br>2.70, d (8.2)	45.6	2.81, d (7.2)
11	12.5	0.73, m	12.9	0.99, d (6.9)	12.9	0.85, d (7.3)
12	19.6	1.20, s	19.9	1.20, s	19.8	1.20, s
13	131.4	6.03, dd (15.3, 10.1)	131.5	5.93, dd (15.4, 10.1)	130.8	6.27, m
14	131.3	5.65, m	131.1	5.57, ddd (15.4, 10.1, 5.8)	131.6	5.56, m
15	37.6	2.62, q (11.7)<br>2.10, m	37.8	2.53, q (12.0)<br>2.04, dd (12.3, 5.1)	37.7	2.50, m<br>2.17, m
16	41.9	3.23, dd (10.8, 6.8)	41.9	3.18, m	43.8	2.97, m
17	215.6		215.8		216.0	
18	76.5		76.6		50.7	2.27, m
19	60.0	3.19, s	60.8	3.38, s	58.8	2.94, dd (8.8, 2.2)
20	52.9	3.53, s	55.4	3.44, s	57.2	3.54, d (1.4)
21	72.6	5.65, m	73.1	3.91, s	72.9	5.55, m
22	19.2	1.22, d (6.6)	19.2	1.17, d (6.6)	18.9	1.13, d (6.8)
23	21.9	1.54, s	21.9	1.52, s	15.0	1.29, d (6.9)
24	138.9		139.0		139.0	
25	127.4	7.28, m	127.6	7.26, m	127.6	7.27, m
26	134.7		134.9		135.0	
27	130.2	7.28, m	130.4	7.26, m	130.5	7.27, m
28	129.5	7.26, s	129.5	7.15, s	129.3	7.20, t (1.5)
29	127.8	7.15, d (7.2)	127.7	7.04, d (6.7)	127.6	7.09, dt (7.2, 1.5)
30	170.0				170.3	
31	20.7	2.14, s			20.7	2.12, s
18-OH		4.12, s		4.12, s		

Table S9. ^{1}H and ^{13}C NMR (700/175 MHz) data of *m*-bromo-deacetyl-19,20-epoxycytochalasin C (**17**) and ^{1}H and ^{13}C NMR (500/125 MHz) data of *m*-bromo-19,20-epoxycytochalasin Q (**18**), and *m*-bromo-deacetyl-19,20-epoxycytochalasin Q (**19**) in $CDCl_3$.

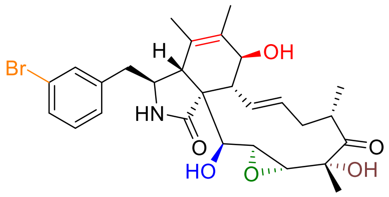

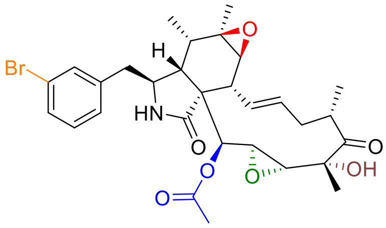

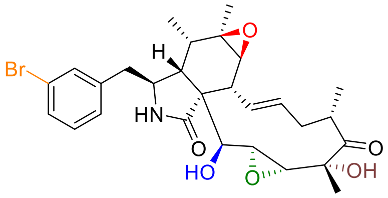

N°	*m*-bromo-deacetyl-19,20-epoxycytochalasin C (**17**)		*m*-bromo-19,20-epoxycytochalasin Q (**18**)		*m*-bromo-deacetyl-19,20-epoxycytochalasin Q (**19**)	
	δ_C	δ_H	δ_C	δ_H	δ_C	δ_H
1	175.4		174.6		176.1	
2		5.58, s		6.19, s		6.36, s
3	60.8	3.34, dd (9.8, 5.0)	54.3	3.56, t (6.9)	54.4	3.64, m
4	52.0	2.88, s	51.1	2.17, dd (5.5, 1.7)	52.6	2.43, dt (5.1, 2.9)
5	126.5		36.8	1.59, p (7.1)	36.6	1.82, p (7.0)
6	131.8		57.3		57.5	
7	68.1	3.77, d (10.0)	62.5	2.71, d (5.6)	62.9	2.64, d (5.7)
8	47.8	2.41, t (10.0)	44.9	2.31, dd (10.0, 5.7)	43.2	2.46, m
9	51.7		54.7		55.0	
10	45.4	2.97, dd (13.3, 5.0)<br>2.79, dd (13.3, 9.9)	45.3	2.85, dd (13.3, 7.3)<br>2.78, dd (13.3, 7.3)	45.6	2.79, dd (13.4, 5.4)<br>2.68, dd (13.4, 7.9)
11	14.0	1.70, s	12.6	0.76, d (7.2)	12.8	0.97, d (7.2)
12	17.5	1.59, s	19.7	1.17, s	19.9	1.20, s
13	131.7	6.10, dd (15.6, 10.0)	131.5	6.01, dd (15.4, 10.0)	131.5	5.92, dd (15.4, 10.1)
14	134.0	5.66, ddd (15.6, 10.0, 5.9)	131.3	5.62, m	131.0	5.57, ddd (15.4, 10.1, 5.8)
15	37.7	2.63, q (12.3)<br>2.09, dd (13.1, 6.1)	37.7	2.60, q (12.2)<br>2.08, dd (13.3, 7.5)	37.8	2.52, m<br>2.03, dd (12.3, 5.4)
16	42.0	3.21, dtt (13.7, 6.8, 3.3)	42.0	3.20, dd (11.1, 7.4)	41.9	3.17, dt (11.7, 5.9)
17	215.5		215.6		215.8	
18	76.6		76.5		76.6	
19	60.5	3.42, s	60.0	3.15, d (1.6)	60.7	3.38, s
20	55.4	3.42, s	52.9	3.51, s	55.4	3.44, s
21	72.9	4.19, s	72.7	5.63, m	73.0	3.90, s
22	19.3	1.20, d (6.7)	19.2	1.19, d (6.7)	19.2	1.16, d (6.7)
23	22.0	1.57, s	22.0	1.51, s	21.9	1.51, s
24	139.8		139.2		139.3	
25	132.0	7.31, s	132.3	7.37, s	132.4	7.31, s
26	123.2		123.1		123.0	
27	130.7	7.41, d (7.9)	130.4	7.39, d (7.6)	130.5	7.39, d (7.6)
28	130.6	7.21, t (7.9)	130.6	7.20, t (7.6)	131.0	7.20, t (7.6)
29	127.9	7.08, d (7.9)	128.3	7.15, d (7.6)	128.1	7.09, d (7.6)
30			170.0			
31			20.8	2.11, s		
18-OH		4.11, s		4.05, s		4.08, s

Table S10. ^{1}H and ^{13}C NMR (500/125 MHz) data of *o*-bromo-19,20-epoxy-21-oxocytochalasin Q (**20**) and *o*-bromo-19-hydroxy-21-oxocytochalasin Q (**21**) in $CDCl_3$.

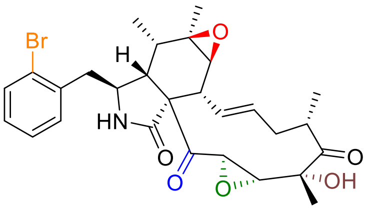

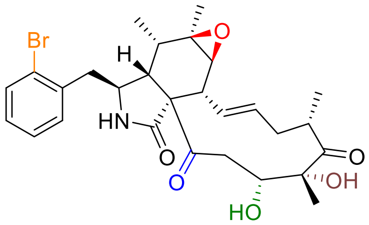

N°	*o*-bromo-19-hydroxy-21-oxocytochalasin Q (**20**)		*o*-bromo-19,20-epoxy-21-oxocytochalasin Q (**21**)	
	δ_C	δ_H	δ_C	δ_H
1	173.7		173.5	
2		5.79, s		5.76, s
3	51.5	3.88, t (6.7)	51.1	3.81, t (6.8)
4	49.0	2.90, d (5.9)	47.2	2.99, d (5.6)
5	36.6	1.84, dt (13.6, 7.1)	36.5	1.77, dt (13.9, 7.1)
6	57.6		57.7	
7	61.6	2.84, d (5.6)	61.6	2.86, d (5.7)
8	50.6	2.09, dd (10.0, 5.6)	49.8	2.06, m
9	65.9		65.9	
10	45.2	2.81, m<br>2.70, dd (11.4, 5.8)	44.8	2.73, dd (13.3, 5.9)<br>2.62, m
11	12.7	1.07, d (7.3)	12.6	1.03, d (7.2)
12	19.5	1.25, s	19.5	1.20, s
13	130.5	6.08, dd (15.4, 10.0)	130.6	6.14, dd (15.4, 10.0)
14	131.5	5.35, ddd (15.5, 10.5, 5.2)	131.3	5.22, ddd (15.4, 10.0, 5.0)
15	38.2	2.65, m<br>2.15, m	44.8	2.62, m<br>2.15, m
16	41.6	3.13, m	41.2	3.04, m
17	214.2		216.9	
18	76.5		83.0	
19	65.1	3.22, d (1.6)	69.2	4.08, d (8.0)
20	52.2	4.22, d (1.6)	38.4	2.06, m
21	204.5		210.4	
22	19.6	1.23, d (6.7)	20.5	1.17, d (6.7)
23	21.8	1.61, s	20.3	1.52, s
24	135.6		135.8	
25	124.6		124.7	
26	132.2	7.13, d (7.5)	132.2	7.08, d (7.5)
27	128.2	7.31, t (7.5)	128.1	7.27, m
28	129.4	7.18, t (7.5)	129.3	7.15, t (7.5)
29	133.5	7.60, d (7.5)	133.4	7.56, d (7.5)
30				
31				
32		4.16, s		3.35, s
18-OH				3.35, s

Table S11. Abundance of phenylglucopyranoside derivatives produced by *Xylaria* sp. XSP14 (n.d.: not detected; +: detected; ++: detected with high abundancy. +++: detected with abundancy comparable to major cytochalasans)

Amino acid		2 mM	10 mM	20 mM
L-phenylalanine	4-fluoro	+	+	+
	4-bromo	+	+	+
	3-bromo	+	++	+++
	2-bromo	+	+	++
	4-chloro	+	+	++
	3-chloro	+	++	+++
	2-chloro	+	+++	+++
	4-iodo	n.d.	n.d.	n.d.
	3-iodo	n.d.	n.d.	n.d.
	2-iodo	n.d.	n.d.	n.d.
	4-methoxy	n.d.	n.d.	n.d.
	3-methoxy	n.d.	n.d.	n.d.
	2-methoxy	n.d.	n.d.	n.d.
	4-methyl	n.d.	n.d.	n.d.
	3-methyl	n.d.	n.d.	n.d.
	2-methyl	n.d.	n.d.	n.d.
	4-ethyl	n.d.	n.d.	n.d.
	4-phenyl	n.d.	n.d.	n.d.
	4-acetyl	n.d.	n.d.	n.d.
	4-amino	n.d.	n.d.	n.d.
	4-nitro	n.d.	n.d.	n.d.
	4-azido	+	++	++
	3-hydroxy	n.d.	n.d.	n.d.
	3-cyano	n.d.	n.d.	n.d.
L-tyrosine		n.d.	n.d.	n.d.
3-fluoro-L-tyrosine		n.d.	n.d.	n.d.
3-bromo-L-tyrosine		n.d.	n.d.	n.d.
O-propargyl-L-tyrosine		+	++	++
L-leucine		n.d.	n.d.	n.d.
L-tryptophan		n.d.	n.d.	n.d.

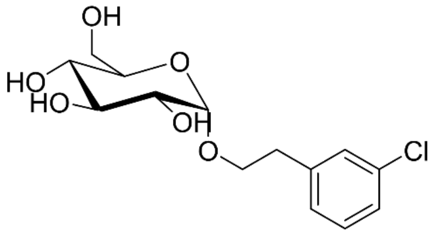

m-chloro-phenylethyl-*α*-D-glucopyranoside (**28**)

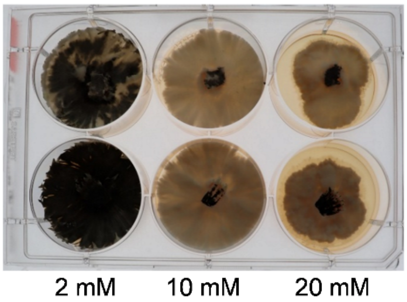

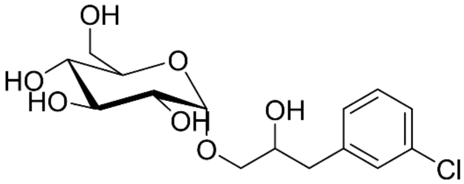

m-chloro-2-hydroxy-3-phenylpropyl-
α-D-glucopyranoside (**29**)

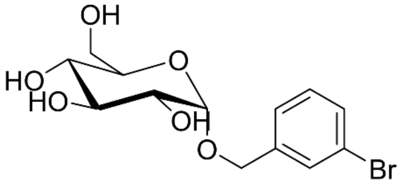

m-chloro-phenylethyl-*α*-D-glucopyranoside **(30)**

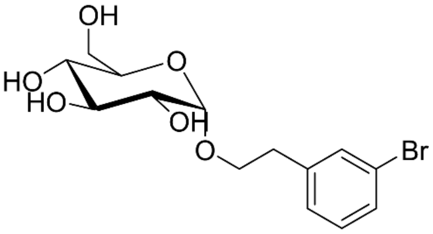

m-bromo-phenylethyl-*α*-D-glucopyranoside (**31**)

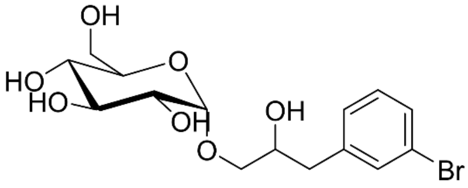

m-bromo-2-hydroxy-3-phenylpropyl-
α-D-glucopyranoside (**32**)

Figure S8. Picture of 6-well plates of *Xylaria* sp. XSP14 on PDA supplemented with 3-chloro-L-phenylalanine at 2, 10 and 20 mM concentration and structures of isolated phenylglucopyranoside derivatives from *Xylaria* sp. XSP14 cultures supplemented with 3-chloro and 3-bromo -L-phenylalanine.

***m*-chloro-phenylethyl-*α*-D-glucopyranoside (28):**

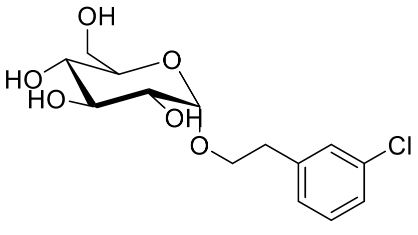

ESI-HRMS [M+Na]$^+$ *m/z* 341.0758 (calcd for $C_{14}H_{19}ClNaO_6$, 341.0762).

NMR spectral data, see Table S12.

***m*-chloro-2-hydroxy-3-phenylpropyl-*α*-D-glucopyranoside (29):**

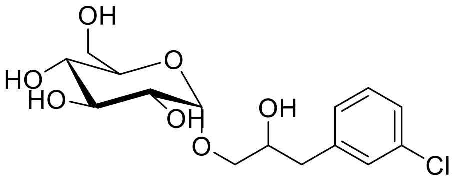

ESI-HRMS [M+Na]$^+$ *m/z* 371.0866 (calcd for $C_{15}H_{21}ClNaO_7$, 371.0868).

NMR spectral data, see Table S12.

Table S12. ^{1}H and ^{13}C NMR (500/125 MHz) data of *m*-chloro-phenylethyl-*α*-D-glucopyranoside (**28**) and *m*-chloro-2-hydroxy-3-phenylpropyl-*α*-D-glucopyranoside (**29**) in MeOH-*d₄*.

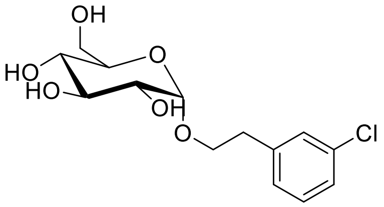

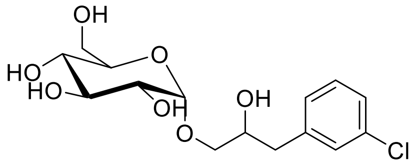

N°	*m*-chloro-phenylethyl-*α*-D-glucopyranoside (**27**)		*m*-chloro-2-hydroxy-3-phenylpropyl-*α*-D-glucopyranoside (**28**)	
	δ_C	δ_H	δ_C	δ_H
1	69.5	3.86, q (7.4)<br>3.64, m	63.6	3.63, dd (11.7, 3.4)<br>3.48, dq (11.7, 5.2, 4.7)
2	36.5	2.88, t (6.9)	81.0	3.88, tt (7.1, 4.7)
3	142.6		38.8	2.86, d (6.9)
4	129.9	7.24, s	142.4	
5	134.9		130.5	7.26, s
6	127.2	7.14, t (7.6)	135.1	
7	130.8	7.19, t (7.6)	127.4	7.15, d (7.5)
8	128.4	7.14, t (7.6)	130.9	7.21, m
9			129.1	7.15, d (7.5)
2‘	100.0	4.78, d (3.6)	99.6	4.94, d (3.8)
3‘	73.3	3.38, dd (9.7, 3.6)	73.6	3.34, dd (9.6, 3.8)
4‘	74.9	3.59, m	74.9	3.54, t (9.6)
5’	73.5	3.33, m	70.9	3.26, t (9.6)
6’	71.4	3.27, t (9.3)	73.5	3.05, ddd (9.6, 3.8, 2.0)
8‘	62.4	3.70, m<br>3.59, m	61.9	3.48, dq (11.7, 5.2, 4.7)<br>3.40, dd (11.7, 2.0)

_m_-bromo-benzyl-_α_-D-glucopyranoside (30):

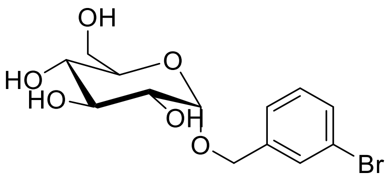

ESI-HRMS $[M+Na]^+$ *m/z* 371.0099 (calcd for $C_{13}H_{17}BrNaO_6$, 371.0101).

NMR spectral data, see

Table S13.

***m*-bromo-phenylethyl-*α*-D-glucopyranoside (31):**

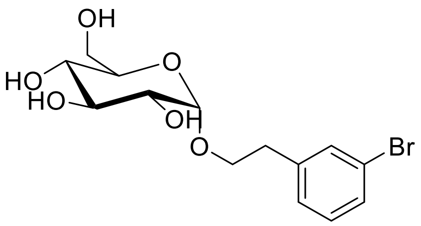

ESI-HRMS $[M+Na]^+$ *m/z* 385.0255 (calcd for $C_{14}H_{19}BrNaO_6$, 385.0257).

NMR spectral data, see

Table S13.

***m*-bromo-2-hydroxy-3-phenylpropyl-*α*-D-glucopyranoside (32):**

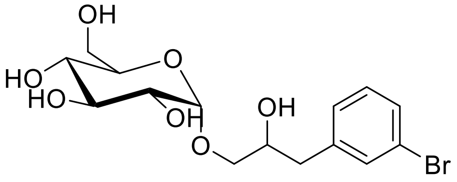

ESI-HRMS [M+Na]$^+$ *m/z* 415.0358 (calcd for $C_{15}H_{21}BrNaO_7$, 415.0363).

NMR spectral data, see

Table S13.

Table S13. ^{1}H and ^{13}C NMR (500/125 MHz) data of *m*-bromo-benzyl-*α*-D-glucopyranoside (**30**), *m*-bromo-phenylethyl-*α*-D-glucopyranoside (**31**), and *m*-bromo-2-hydroxy-3-phenylpropyl-*α*-D-glucopyranoside (**32**) in MeOH-d_4.

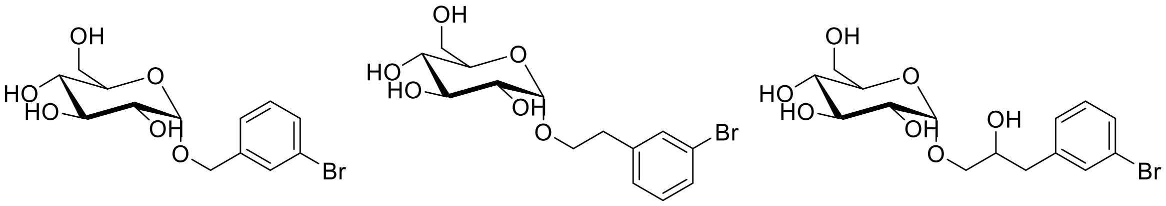

N°	*m*-bromo-benzyl-*α*-D-glucopyranoside (**30**)		*m*-bromo-phenylethyl-*α*-D-glucopyranoside (**31**)		*m*-bromo-2-hydroxy-3-phenylpropyl-*α*-D-glucopyranoside (**32**)	
	*δ*C	*δ*H	*δ*C	*δ*H	*δ*C	*δ*H
1	69.3	4.75, d (12.3)<br>4.54, d (12.3)	69.5	3.86, dt (9.6, 7.3)<br>3.64, m	63.6	3.63, dd (12.0, 3.4)<br>3.48, td (12.0,4.4)
2	141.7		36.5	2.88, d (7.0)	81.0	3.88, tt (7.0, 4.4)
3	132.0	7.63, s	143.0		38.8	2.85, d (6.9)
4	123.3		132.9	7.40, s	142.7	
5	131.7	7.43, d (7.8)	123.2		133.4	7.41, s
6	131.1	7.25, t (7.8)	130.3	7.29, d (7.8)	123.3	
7	127.8	7.38, d (7.8)	131.1	7.14, t (7.8)	130.4	7.31, d (7.7)
8			128.9	7.20, d (7.8)	131.2	7.15, t (7.7)
9					129.6	7.20, d (7.7)
2‘	99.4	4.88, d (3.7)	100.0	4.77, d (3.7)	99.6	4.94, d (3.8)
3‘	73.5	3.43, dd (9.7, 3.7)	73.6	3.37, dd (9.7, 3.7)	73.6	3.34, dd (9.6, 3.8)
4‘	75.0	3.68, m	75.0	3.58, m	74.9	3.54, t (9.6)
5’	71.8	3.31, m	73.4	3.33, m	70.9	3.26, t (9.6)
6’	74.0	3.60, m	71.5	3.25, m	73.5	3.03, ddd (9.6, 3.9, 2.3)
8‘	62.6	3.80, dd (11.8, 2.1)<br>3.68, m	62.4	3.69, dd (11.9, 2.1)<br>3.58, m	61.9	3.48, td (12.0,4.4)<br>3.41, dd (12.0, 2.3)

5. Co-culture of *Xylaria* sp. XSP14 and *Streptomyces* sp. M56

Time-dependent co-culture of *Xylaria* sp. XSP14 and *Streptomyces* sp. M56: Two PDA plates of 2-week-old *Xylaria* sp. XSP14 culture were inoculated with 2 agar cubes. Afterwards, the pre-culture was used to inoculate 6 co-culture plates by putting 2 agar cubes of *Xylaria* sp. XSP14 on one side. The plates were then incubated at 25 °C in the dark for 1 week. Liquid cultures of *Streptomyces* sp. M56 were prepared by excising PDA slices covered with bacterial growth from a plate culture less than 1 week old and adding them to 50 mL PDB liquid medium. The inoculated broth was then incubated at 30 ºC while shaken at 150 rpm. Turbidity was reached within 4 days. After the one-week incubation of the co-culture plates, *Streptomyces* sp. M56 was inoculated with 50 µl cell suspension on the other side. The plates were kept at 25 °C in the dark for 2 more weeks. The production of metabolites was monitored by HR-UPLC-ESI-MS/MS analyses. For that, 3 plates were extracted after 1 and 2 weeks of incubation by cutting the agar into pieces and soaking in MeOH overnight. The organic extract was filtered, dried under vacuum, dissolved in MeOH with a final concentration of 50 µg/mL, and analyzed by HRMS. Data set of the molecular network has been submitted to MassIVE server: MSV000098573.

Preparative co-culture *Xylaria* sp. XSP14 and *Streptomyces* sp. M56: 5 PDA plates of 2-week-old *Xylaria* sp. XSP14 culture were used to inoculate 100 co-culture plates by putting 2 agar cubes of *Xylaria* sp. XSP14 on one side. The plates were then incubated at 25 °C in the dark for 1 week. Liquid cultures of *Streptomyces* sp. M56 were prepared by excising PDA slices covered with bacterial growth from a plate culture less than 1 week old and adding them to 50 mL PDB liquid medium. The inoculated broth was then incubated at 30 ºC while shaken at 150 rpm. After the one-week incubation of the co-culture plates, *Streptomyces* sp. M56 was inoculated with 50 µl cell suspension on the other side. The plates were kept at 25 °C in the dark for 2 more weeks. Agar plates were then cut into pieces, each side (bacteria and fungus) of the plates were separated into two different beakers, soaked in MeOH overnight. The two organic extracts were filtered and dried under vacuum. Both crude extracts (8.0 g for XSP14 side and 2.6 g for M56 side) were then fractionated by SPE-C18 open columns by step-wise elution using H_2O/MeOH mixtures to afford 5 fractions for each crude extract. Fraction 3 of XSP14-side extract (120 mg) was subjected to semi-preparative reverse-phase HPLC (Phenomenex Luna C18(2) 250 x 10 mm, 5 µm, 100 Å column) using the following gradient: 0–5 min, 30% B; 5–40 min, 30%–75% B; 40–43 min, 75%-100% B; 43-50 min, 100% (A: H_2O; B: CH_3CN) with a flow rate of 3 mL/min to yield deacetyl-19,20-epoxycytochalasin Q (**7**, 1.4

mg) and 19,20-epoxycytochalasin Q (**6**, 5 mg). Fraction 4 of XSP14-side extract (340 mg) was subjected to semi-preparative reverse-phase HPLC (Phenomenex Luna C18(2) 250 x 10 mm, 5 µm, 100 Å column) using the following gradient: 0–5 min, 30% B; 5–40 min, 30%–75% B; 40–43 min, 75%-100% B; 43-50 min, 100% (A: H_2O + 0.1% Formic acid; B: CH_3CN + 0.1% Formic acid) with a flow rate of 3 mL/min to yield xylachalasin A (**10**, 5.6 mg), deacetyl-19,20-epoxycytochalasin C (**4**, 3.5 mg), 19,20-epoxycytochalasin C (**3**, 40 mg), and 18-desoxy-19,20-epoxycytochalasin C (**5**, 1 mg). Fraction 5 of XSP14-side extract (220 mg) was subjected to semi-preparative reverse-phase HPLC (Phenomenex Luna C18(2) 250 x 10 mm, 5 µm, 100 Å column) using the following gradient: 0–5 min, 30% B; 5-40 min, 30%-75% B; 40-43 min, 75%-100% B; 43-50 min, 100% (A: H_2O + 0.1% Formic acid; B: CH_3CN + 0.1% Formic acid) with a flow rate of 3 mL/min to yield xylachalasin A (**10**, 3.8 mg), deacetyl-19,20-epoxycytochalasin C (**4**, 2.7 mg), 19,20-epoxycytochalasin D (**9**, 0.7 mg), and 19,20-epoxycytochalasin C (**3**, 30 mg).

Co-culture of *Xylaria* sp. XSP14 and *Streptomyces* sp. M56 on a labelled phenylalanine-enriched medium. The same protocol has previously described in the section “Co-culture XSP14/M56 – Set up and time studies” has been conducted to prepare 3 plates with the addition of a labelled phenylalanine (1-^{13}C) at a concentration of 10 mM in PDA medium. The plates were extracted by cutting them into pieces and soaking them in MeOH overnight. The organic extract was filtered, dried under vacuum, dissolved in MeOH with a final concentration of 50 µg/mL, and analyzed by HRMS.

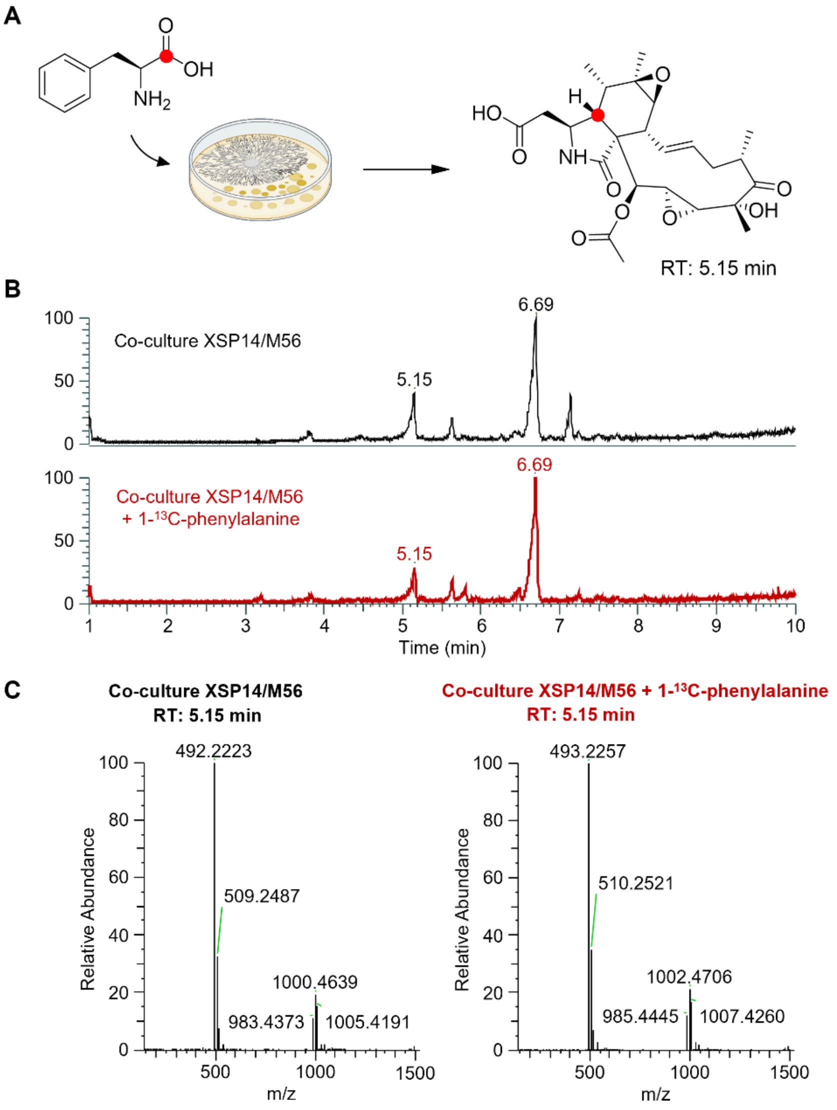

Figure S9. A) Comparative UHPLC-HRMS chromatograms of co-cultures of *Xylaria* sp. XSP14 and *Streptomyces* sp. M56 extracts with and without addition of 1-^{13}C-phenylalanine in PDA medium; B) Mass spectra of peaks at 5.15 min of co-cultures of *Xylaria* sp. XSP14 and *Streptomyces* sp. M56 extracts with and without addition of 1-^{13}C-phenylalanine in PDA medium.

Streptomyces sp. M56 cultivation on a cytochalasin-enriched medium: Liquid cultures of *Streptomyces* sp. M56 were prepared by excising PDA slices covered with bacterial growth from a plate culture less than 1 week old and adding them to 50 mL PDB liquid medium. The inoculated broth was then incubated at 30 ºC while shaken at 150 rpm. Turbidity was reached within 4 days. A cytochalasans-enriched fraction from previous fractionation of XSP14 extract was used to prepare a cytochalasans-enriched PDA medium at a concentration of 0.5 mg/mL. Then, *Streptomyces* sp. M56 was inoculated with 100 µl cell suspension on 3 cytochalasans-enriched PDA plates. The plates were kept at 25 °C in the dark for 2 weeks. Then, the plates were extracted by cutting them into pieces and soaking them in MeOH overnight. The organic extract was filtered, dried under vacuum, dissolved in MeOH with a final concentration of 50 µg/mL, and analyzed by HRMS. Data set of the molecular network has been submitted to MassIVE server: MSV000098574.

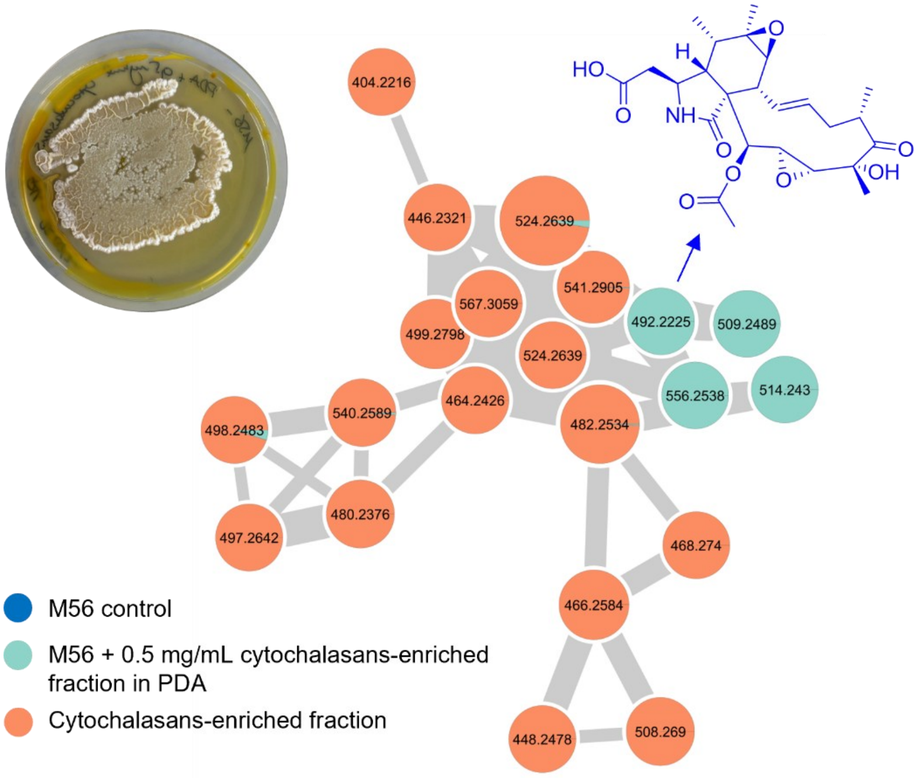

Figure S10. Plate of *Streptomyces* sp. M56 axenic culture with 0.5 mg/mL cytochalasans-enriched fraction in PDA medium and expanded representative GNPS-feature-based molecular network cluster of cytochalasans from *Streptomyces* sp. M56 axenic culture extract, extract of *Streptomyces* sp. M56 axenic culture with 0.5 mg/mL cytochalasans-enriched fraction in PDA medium, and cytochalasans-enriched fraction.

***Streptomyces* sp. M56 cultivation on medium supplemented with 19,20-epoxycytochalasin Q (6):** Liquid cultures of *Streptomyces* sp. M56 were prepared by excising PDA slices covered with bacterial growth from a plate culture less than 1 week old and adding them to 50 mL PDB liquid medium. The inoculated broth was then incubated at 30 ºC while shaken at 150 rpm. Turbidity was reached within 4 days. 19,20-epoxycytochalasin Q (**6**) was used to prepare a PDA medium supplemented with this compound at a concentration of 1 mM. Then, *Streptomyces* sp. M56 was inoculated with 100 µl cell suspension on 3 19,20-epoxycytochalasin Q-enriched PDA plates. The plates were kept at 25 °C in the dark for 2 weeks. Then, the plates were extracted by cutting them into pieces and soaking them in MeOH overnight. The organic extract was filtered, dried under vacuum, dissolved in MeOH with a final concentration of 50 µg/mL, and analyzed by HRMS.

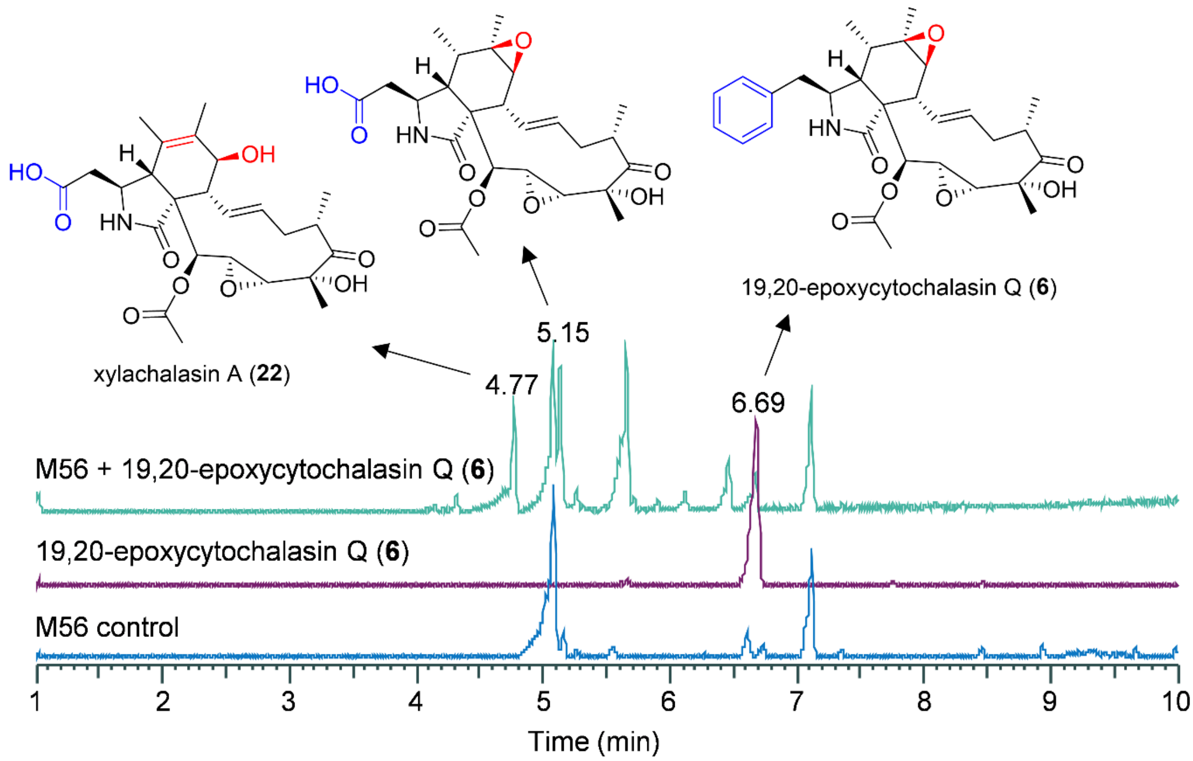

Figure S11. Comparative UHPLC-HRMS chromatograms of *Streptomyces* sp. M56 axenic culture supplemented with a) 1 mM of 19,20-epoxycytochalasin Q (**6**) in PDA medium, b) 19,20-epoxycytochalasin Q (**6**), and c) *Streptomyces* sp. M56 axenic culture extract.

Instability of xylachalasin derivatives: The same protocol has previously described in the section "Co-culture XSP14/M56 – Set up and time studies" was conducted to prepare 3 plates in PDA medium. The plates were extracted by cutting them into pieces and soaking them in MeOH overnight. The organic extract was filtered, dried under vacuum, dissolved in MeOH with a final concentration of 50 µg/mL, and analyzed by HRMS.

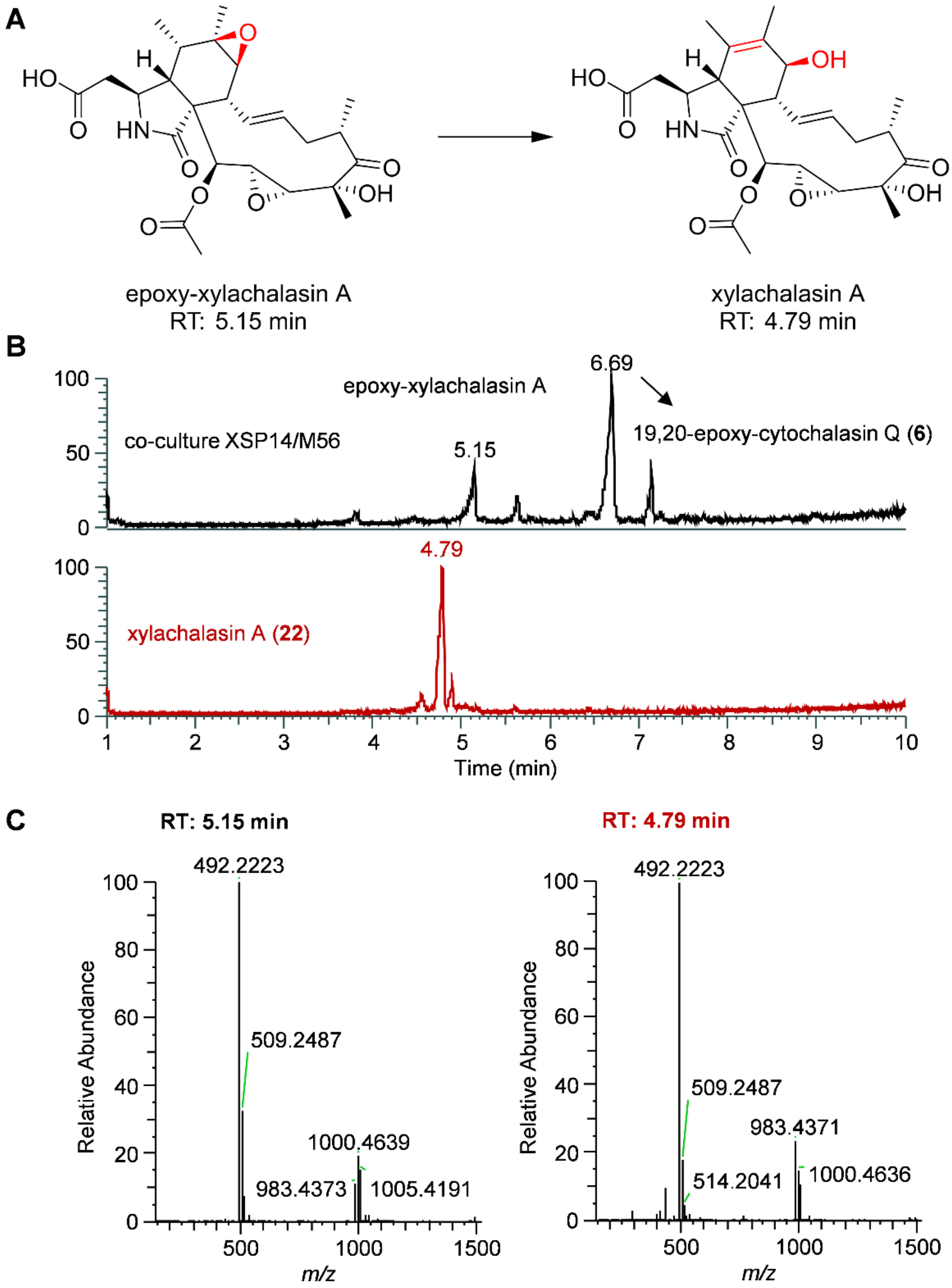

Figure S12. A) UHPLC-HRMS chromatograms of co-culture of *Xylaria* sp. XSP14 and *Streptomyces* sp. M56 extracts and xylachalasin A (**22**); B) mass spectrum from UHPLC-HRMS analysis of co-culture experiment with peak at 5.15 min (putative epoxy-xylachalasin A) and mass spectrum of the peak at 4.79 min of purified product **22**.

Xylachalasin A (**22**) was fully characterized by HRMS and 1D and 2D NMR analyses. The molecular formula, $C_{25}H_{33}NO_9$, was deduced from ESI(+)HRMS data (*m/z* 492.2223 [M+H]+, calcd. for $C_{25}H_{34}NO_9$, 492.2228). The ^{1}H NMR and ^{13}C NMR spectra were close to the reported and isolated compound 19,20-epoxycytochalasin C (**3**), differing by a lack of the aromatic moiety.

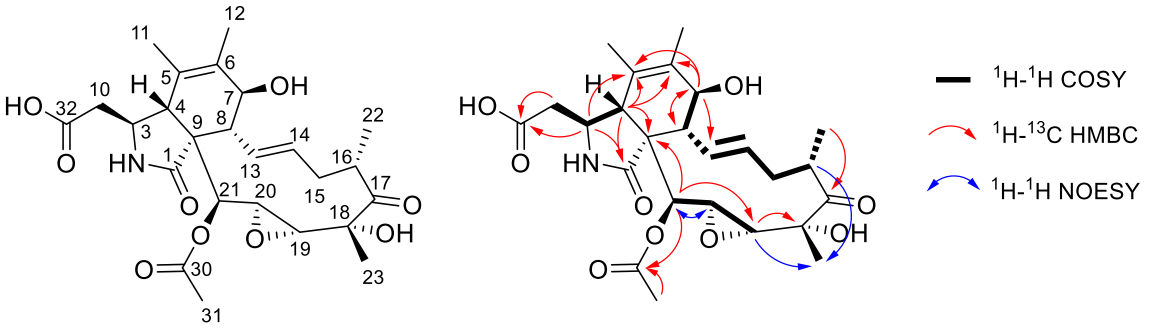

^{1}H and ^{13}C NMR spectra revealed signals for four carbonyls (δ_C 216.7, 174.7, 176.9, and 172.1), two methylenes (δ_H 2.81 (d, *J* = 7.1 Hz, 2H, H-10), δ_C 43.7; δ_H 2.54 (dd, *J* = 9.4 Hz, 1H, H-15), 2.11 (m, 1H, H-15), δ_C 39.3), eight methines (δ_H 5.53 (s, 1H, H-21), δ_C 73.0; δ_H 3.78 (d, *J* = 10.8 Hz, 1H, H-7), δ_C 70.0; δ_H 3.65 (s, 1H, H-3), δ_C 56.7); δ_H 3.42 (s, 1H, H-19), δ_C 61.1; δ_H 3.36 (s, 1H, H-16), δ_C 42.8; δ_H 3.25 (s, 1H, H-20), δ_C 53.9; δ_H 2.26 (s, 1H, H-8), δ_C 49.8; δ_H 2.44 (s, 1H, H-4), δ_C 42.5), and five methyls at δ_H 2.14 (s, 3H, H-31), 1.69 (s, 6H, H-11, H-12), 1.49 (s, 3H, H-23), and 1.15 (d, *J* = 6.6 Hz, 3H, H-22) corresponding to the signals of their attached carbons at δ_C 20.4, 14.5, 17.7, 22.3, and 19.5, respectively. Signals at δ_H 5.89 (1H, dd, *J* = 15.5, 10.2 Hz) and 5.64 (1H, ddd, *J* = 15.6, 10.1, 5.8 Hz) in the ^{1}H NMR spectrum and signals at δ_C 127.9, 132.4, 133.9, and 134.0 in the ^{13}C NMR spectrum suggested the presence of two olefinic bonds. Three fragments, –CH_2CHCH, –CHCHCHCHCH$_2$–, and –CHCHCH–, were observed from the ^{1}H-^{1}H COSY spectrum. Thus, the substituted isoindole scaffold fused to the macrocyclic ring of **10** was shown to be the exact same as 19,20-epoxycytochalasin C (**3**). Comparison of the ^{1}H et ^{13}C chemical shifts of both compounds and HMBC key correlations confirmed this assumption. The presence of a carboxylic acid linked to C-10 instead of an aromatic ring in 19,20-epoxycytochalasin C (**3**) was confirmed by the key HMBC correlations from H-3 (δ_H 3.65, s, 1H) to C-32 (δ_C 174.7) and H-10 (δ_H 2.81, d, *J* = 7.1 Hz, 2H) to C-32 (δ_C 174.7). The relative configuration 3*S**, 4*R**, 7*S**, 8*R**, 9*R**, 16*S**, 18*R**, 19*R**, 20*S**, 21*S** of **10** was determined on the analysis of the NOESY experiment and highly supported by the X-ray structure of 19,20-epoxycytochalasin C (**3**).

$[\alpha]^{20}_{D}$ = +5.2 (c 0.7, MeOH).

Table S14. ^{1}H and ^{13}C NMR (500/125 MHz) data of xylachalasin A (**22**) and 19,20-epoxycytochalasin C (**3**) in MeOH-*d*$_4$.

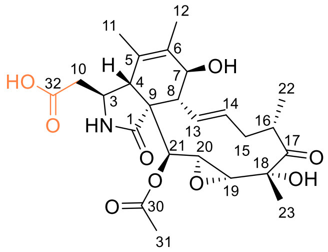

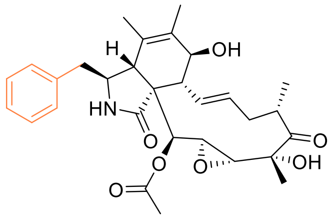

N°	Xylachalasin A (**22**)		19,20-epoxycytochalasin C (**3**)	
	δ_C	δ_H	δ_C	δ_H
1	176.9		176.7	
2				5.36, s
3	56.7	3.65, t (7.2)	62.8	3.33, m
4	52.5	2.44, s	50.8	2.50, s
5	127.9		127.9	
6	133.9		133.8	
7	70.0	3.78, t (10.8)	70.1	3.74, m
8	49.8	2.26, m	50.0	2.26, t (10.1)
9	53.9		54.0	
10	43.7	2.81, d (7.1)	44.9	3.03, m
11	14.5	1.69, s	14.4	1.59, s
12	17.7	1.69, s	17.0	0.97, s
13	132.4	5.89, dd (15.5, 10.2)	132.6	5.92, dd (15.7, 10.2)
14	134.0	5.64, ddd (15.6, 10.1, 5.8)	133.8	5.64, ddd (15.7, 10.2, 5.8)
15	39.3	2.54, dd (9.4)<br>2.11, m	39.4	2.55, m<br>2.10, m
16	42.8	3.36, m	42.7	3.38, m
17	216.7		216.7	
18	77.8		77.8	
19	61.1	3.42, s	61.2	3.45, d (2.0)
20	53.9	3.25, s	54.7	3.33, m
21	73.0	5.53, s	73.7	5.73, s
22	19.5	1.15, d (6.6)	19.5	1.16, d (6.7)
23	22.3	1.49, s	22.2	1.51, s
24			139.0	
25			130.8	7.33, m
26			129.7	7.33, m
27			127.9	7.23, m
28			129.7	7.33, m
29			130.8	7.33, m
30	172.1		172.1	
31	20.4	2.14, s	20.5	2.19, s
32	174.7			
18-OH				

6. Semi-synthetic derivatization

7-Acetyl-19,20-epoxycytochalasin C and *N*-acetyl-19,20-epoxycytochalasin Q. A mixture of 20 mg of 19,20-epoxycytochalasin C (**3**) and 19,20-epoxycytochalasin Q (**6**) (1:1, 1.0 eq) was dissolved in 2 mL of CH_2Cl_2 and cooled to 0 °C. Et_3N (1.0 eq.) was added at once and the solution was stirred for 25 min at 0 °C. Then, DMAP solution (1.0 eq., 1 M in CH_2Cl_2) was added dropwise and the reaction was stirred for 30 min. Afterwards, a solution of anhydride acetic (3.0 eq.) was added dropwise. The temperature was allowed to rise to room temperature and the reaction mixture was stirred overnight. After that, the reaction was quenched with sat. aq. $NaHCO_3$. The aqueous phase was extracted with CH_2Cl_2 (3 x). The combined organic layers were washed with brine and dried over Na_2SO_4. Evaporation of the solvent and flash chromatography with a Phenomenex Luna C18(2) 250 x 30 mm column (particle size 5 µm, pore diameter 100 Å) and the following gradient: 0–5 min, 15% B; 5–25 min, 15%–100% B; 25–35 min, 100% B (A: H_2O; B: CH_3CN) with a flow rate of 20 mL/min yielded 7-acetyl-19,20-epoxycytochalasin C (**23**, 0.8 mg, 7%) and *N*-acetyl-19,20-epoxycytochalasin Q (**24**, 6.4 mg, 59%) as white amorphous solids.

7-Acetyl-19,20-epoxycytochalasin C (23):

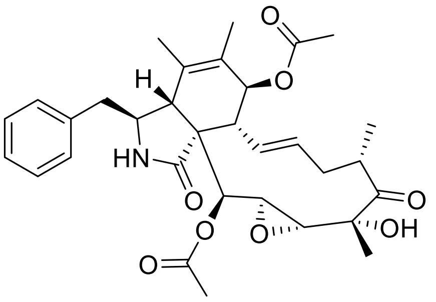

ESI-HRMS $[M+H]^+$ *m/z* 566.2742 (calcd for $C_{32}H_{40}NO_8$, 566.2748). NMR spectral data, see Table S15.

***N*-acetyl-19,20-epoxycytochalasin Q (24):**

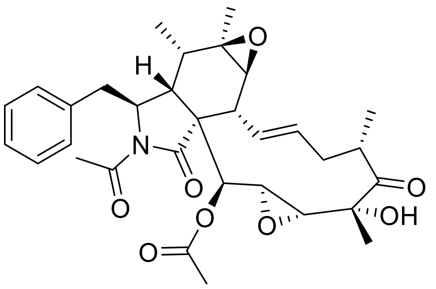

ESI-HRMS $[M+H]^+$ *m/z* 566.2744 (calcd for $C_{32}H_{40}NO_8$, 566.2748). NMR spectral data, see Table S15.

7-Azirin-19,20-epoxycytochalasin C and 7-diazirin-19,20-epoxycytochalasin D. A mixture of 20 mg of 19,20-epoxycytochalasin C and 19,20-epoxycytochalasin Q (1:1, 1.0 eq) was dissolved in 2 mL of CH_2Cl_2 and cooled to 0 °C. EDC hydrochloride (2.0 eq.) was added at once and the solution was stirred for 25 min at 0 °C. Then, DMAP solution (0.2 eq., 1 M in CH_2Cl_2) was added dropwise and the reaction was stirred for 30 min. Afterwards, a solution of 3-(3-(but-3-yn-1-yl)-3H-diazirin-3-yl)propanoic acid (2.0 eq., 0.06 M in CH_2Cl_2) was added dropwise. The temperature was allowed to rise to room temperature and the reaction mixture was stirred overnight. After that, the reaction was quenched with sat. aq. $NaHCO_3$. The aqueous phase was extracted with CH_2Cl_2 (3 x). The combined organic layers were washed with brine and dried over Na_2SO_4. Evaporation of the solvent and flash chromatography with a Phenomenex Luna C18(2) 250 x 30 mm column (particle size 5 µm, pore diameter 100 Å) and the following gradient: 0–5 min, 45% B; 5–20 min, 45%–100% B; 20–35 min, 100% B (A: H_2O; B: CH_3CN) with a flow rate of 20 mL/min yielded 7-diazirin-19,20-epoxycytochalasin C (**25**, 6.7 mg, 52%) and 7-diazirin-19,20-epoxycytochalasin D (**26**, 1 mg, 8%) as white amorphous solids.

7-Diazirin-19,20-epoxycytochalasin C (25):

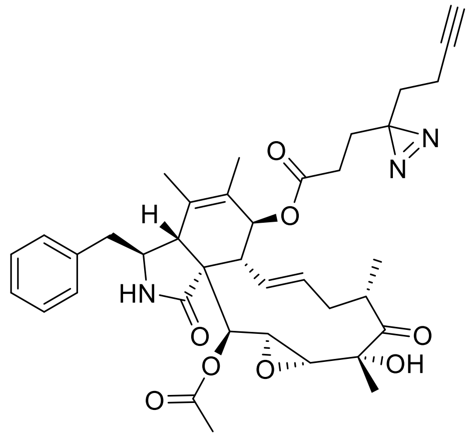

ESI-HRMS $[M+H]^+$ *m/z* 672.3274 (calcd for $C_{38}H_{46}N_3O_8$, 672.3279). NMR spectral data, see Table S16.

7-Diazirin-19,20-epoxycytochalasin D (26):

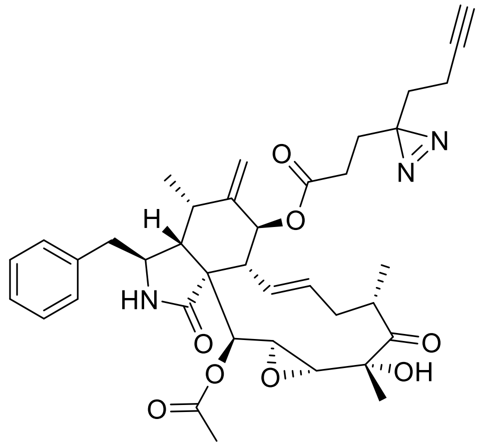

ESI-HRMS $[M+H]^+$ *m/z* 672.3275 (calcd for $C_{38}H_{46}N_3O_8$, 672.3279). NMR spectral data, see Table S16.

19,20-epoxycytochalasin N (27):

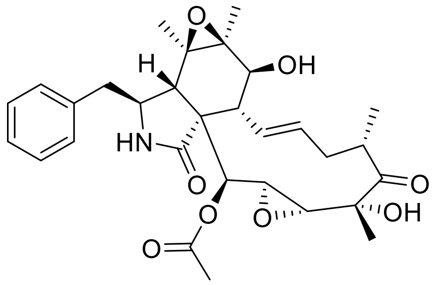

A mixture of 20 mg 19,20-epoxycytochalasin C and 19,20-epoxycytochalasin Q (1:1, 1.0 eq) was dissolved in 2 mL of CH_2Cl_2 and cooled to 0 °C, and then *m*-CPBA (1.2 eq.) was added at once. The temperature was allowed to rise to room temperature and the reaction mixture was stirred overnight. After that, the reaction was quenched with sat. aq. $NaHCO_3$. The aqueous phase was extracted with CH_2Cl_2 (3 x). The combined organic layers were washed with brine and dried over Na_2SO_4. Evaporation of the solvent and flash chromatography with a Phenomenex Luna C18(2) 250 x 30 mm column (particle size 5 µm, pore diameter 100 Å) and the following gradient: 0–5 min, 30% B; 5–25 min, 30%–100% B; 25–35 min, 100% B (A: H_2O; B: CH_3CN) with a flow rate of 20 mL/min yielded 19,20-epoxycytochalasin N (**27**, 6.5 mg, 63%) as clear needle crystals.

ESI-HRMS $[M+H]^+$ *m/z* 540.2589 (calcd for $C_{30}H_{38}NO_8$, 540.2592). ^{1}H NMR (500 MHz, $CDCl_3$) δ 7.34 (t, *J* = 7.4 Hz, 2H), 7.27 (t, *J* = 7.4 Hz, 1H), 7.21 (d, *J* = 7.0 Hz, 2H), 6.07 (dd, *J* = 15.5, 10.6 Hz, 1H), 5.82 (s, 1H), 5.72 (ddd, *J* = 15.7, 10.2, 5.7 Hz, 1H), 5.52 (s, 1H), 4.02 (s, 1H), 3.60 (t, *J* = 6.8 Hz, 1H), 3.51 (dd, *J* = 10.5, 2.0 Hz, 1H), 3.36 (m, 1H), 3.22 (dtt, *J* = 13.2, 6.4, 3.2 Hz, 1H), 3.16 (d, *J* = 1.9 Hz, 1H), 3.07 (dd, *J* = 13.5, 5.4 Hz, 1H), 2.91 (m, 2H), 2.62 (q, *J* = 12.1 Hz, 1H), 2.56 (d, *J* = 2.3 Hz, 1H), 2.18 (s, 3H), 2.11 (dd, *J* = 12.5, 5.4 Hz, 1H), 1.79 (d, *J* = 2.1 Hz, 1H), 1.64 (s, 1H), 1.53 (s, 3H), 1.38 (s, 3H), 1.20 (t, *J* = 3.3 Hz, 6H). ^{13}C NMR (125 MHz, $CDCl_3$) δ 215.3, 173.6, 170.5, 136.9, 135.2, 130.5, 129.3, 129.2, 127.5, 76.4, 72.6, 69.0, 64.2, 63.2, 59.7, 56.7, 53.5, 52.9, 49.3, 44.9, 43.5, 41.8, 37.8, 21.9, 21.0, 19.8, 19.3, 14.3.

Table S15. ^{1}H and ^{13}C NMR (500/125 MHz) data of 7-acetyl-19,20-epoxycytochalasin C (**23**) and *N*-acetyl-19,20-epoxycytochalasin Q (**24**) in $CDCl_3$.

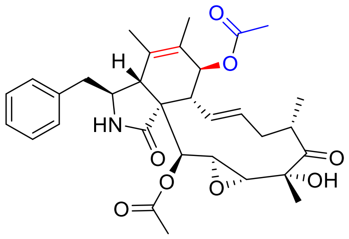

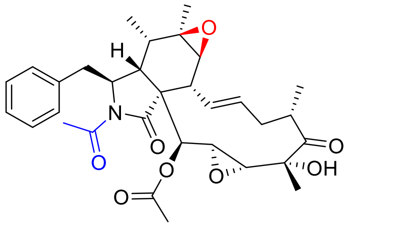

N°	7-acetyl-19,20-epoxycytochalasin C (**23**)		*N*-acetyl-19,20-epoxycytochalasin Q (**24**)	
	δ_C	δ_H	δ_C	δ_H
1	173.6		174.1	
2		5.64, s		
3	60.7	3.35, m	57.3	4.31, d (10.5)
4	50.6	2.56, s	45.9	2.13, m
5	128.6		36.5	1.42, p (7.0)
6	129.0		57.4	
7	71.7	5.31, d (10.2)	62.2	2.57, m
8	46.9	2.42, t (10.2)	45.5	2.37, dd (9.9, 5.9)
9	51.6		57.9	
10	44.8	3.05, qd (13.6, 7.6)	41.6	3.32, dd (12.5, 2.3)<br>2.62, m
11	17.3	1.41, s	12.0	0.31, d (7.2)
12	14.1	1.48, s	18.9	1.01, s
13	131.2	5.99, dd (15.6, 10.2)	131.0	5.88, dd (15.5, 9.9)
14	131.7	5.54, ddd (15.6, 10.2, 6.0)	132.0	5.66, ddd (15.5, 9.9, 5.9)
15	37.6	2.60, m<br>2.03, m	37.7	2.62, m<br>2.11, m
16	41.9		41.9	3.23, dtd (13.0, 6.6, 4.6)
17	215.2		215.7	
18	76.4	3.20, d (2.0)	76.5	
19	60.2	3.44, dd (2.0, 1.0)	60.2	3.17, d (1.8)
20	53.2	5.77, s	52.6	3.38, s
21	72.1	1.18, d (6.7)	71.4	5.73, m
22	19.3	1.52, s	19.3	1.22, d (6.7)
23	22.0		22.0	1.54, s
24	137.5	7.22, m	136.5	
25	129.3	7.33, t (7.3)	130.1	7.23, t (6.7)
26	129.1	7.25, m	128.8	7.33, m
27	127.2	7.33, t (7.3)	127.2	7.33, m
28	129.1	7.22, m	128.8	7.33, m
29	129.3		130.1	7.23, t (6.7)
30	170.2	2.18, s	169.6	
31	21.0		20.8	2.13, s
32	170.1	1.95, s	171.0	
33	20.9	4.06, s	26.0	2.57, s
18-OH				4.06, s

Table S16. ^{1}H and ^{13}C NMR (500/125 MHz) data of 7-diazirin-19,20-epoxycytochalasin C (**25**) and 7-diazirin-19,20-epoxycytochalasin D (**26**) in $CDCl_3$.

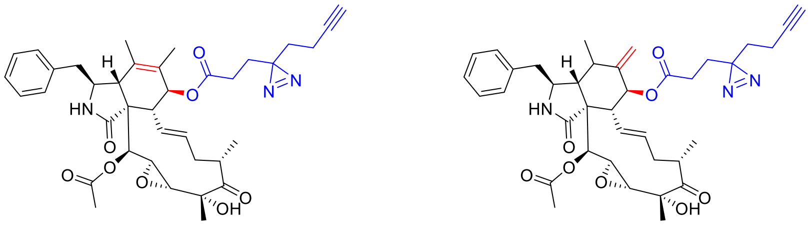

N°	7-diazirin-19,20-epoxycytochalasin C (**25**)		7-diazirin-19,20-epoxycytochalasin D (**26**)	
	δ_C	δ_H	δ_C	δ_H
1	173.6		173.2	
2		5.76, s		5.54, s
3	60.7	3.34, m	54.0	3.24, m
4	50.5	2.55, s	50.4	2.27, dd (5.2, 3.1)
5	128.7		33.0	2.64, p (6.4)
6	128.8		144.8	
7	72.0	5.31, d (10.1)	72.6	5.19, d (11.5)
8	46.8	2.41, t (10.2)	43.9	2.86, m
9	51.7		52.6	
10	44.7	3.05, m	45.2	2.86, m<br>2.76, dd (13.4, 9.1)
11	17.4	1.38, s	13.4	0.86, d (6.7)
12	14.4	1.46, s	115.8	5.20, s<br>5.02, s
13	131.2	5.96, dd (15.6, 10.2)	131.0	5.77, dd (15.5, 9.8)
14	131.7	5.50, ddd (15.6, 10.2, 6.0)	132.0	5.54, m
15	37.6	2.56, m<br>2.00, m	37.5	2.57, m<br>2.00, m
16	41.9	3.19, m	42.0	3.18, ddd (12.0, 6.8, 2.1)
17	215.2		215.3	
18	76.3		76.4	
19	60.2	3.19, d (2.0)	59.9	3.15, d (2.0)
20	53.2	3.43, m	52.9	3.53, s
21	72.0	5.76, s	74.1	5.54, m
22	19.3	1.19, d (6.7)	19.3	1.18, d (6.7)
23	22.0	1.52, s	22.0	1.52, s
24	137.5		137.1	
25	129.3	7.23, t (7.4)	129.3	7.16, t (6.9)
26	129.1	7.32, t (7.4)	129.1	7.33, t (7.4)
27	127.2	7.26, m	127.3	7.25, m
28	129.1	7.32, t (7.4)	129.1	7.33, t (7.4)
29	129.3	7.23, t (7.4)	129.3	7.16, t (6.9)
30	170.2		170.0	
31	21.0	2.18, s	20.9	2.19, s
32	171.2		171.1	
33	28.4	2.00, m<br>1.92, m	28.7	1.94, m
34	28.0	1.77, m<br>1.71, m	28.0	1.71, m
35	69.4		69.4	
36	32.4	1.64, t (7.3)	32.4	1.62, t (7.2)
37	13.4	2.00, m	13.4	2.00, m
38	82.7		82.7	
39	69.4	2.00, m	69.4	2.00, m
18-OH		4.04, s		4.02, s

7. Crystallography data

X-ray analysis. The data set was collected using a Bruker D8 Venture diffractometer (compounds **3**, **4** and **5**) with a microfocus sealed tube and a Photon II detector and a Rigaku XtaLAB Synergy-S diffractometer (compounds **11** and **27**) with a microfocus sealed tube and a HyPix-6000HE Hybrid Photon Counting (HPC) detector, respectively. Monochromated CuKa radiation (λ = 1.54178 Å; compound **3**, **4** and **5**) or MoKα radiation (λ = 0.71073; compound **11** and **27**) was used. Data was collected at 143(2) K (**3** and **4**), 133(2) K (**5**) or 130(2) K (**11** and **27**) and corrected for absorption effects using the multi-scan method. The structures were solved by direct methods using SHELXT[26] and were refined by full matrix least squares calculations on F^2 SHELXL2018[27] in the graphical user interface SHELXLe.[28]

Refinement. All non H-atoms were located in the electron density maps and refined anisotropically. C-bound H atoms were placed in positions of optimized geometry and treated as riding atoms. Their isotropic displacement parameters were coupled to the corresponding carrier atoms by a factor of 1.2 (CH, CH2) or 1.5 (CH3). The O and N bonded H-atoms were located in the electron density maps. Their positional parameters were refined using isotropic displacement parameters which were set at 1.2 (N) and 1.5 (O) times the Ueq value of the parent atoms. Restraints of 0.88(0.01) Å and 0.84(0.01) Å were used for the N-H and O-H bond lengths, respectively. *Disorder:* In compound **4** one benzyl-residue is split over two positions (0.58/0.42). The solvent water was freely refined (sof: 0.31) without including the two hydrogen atoms. In **5** the acetate-group attached on C15 is split over two positions (fvar 2: 0.67/0.33) and the side occupancy factor of the crystal water was freely refined to 0.42.

Crystallographic data has been deposited at the Cambridge Crystallographic Data Centre (CCDC 2473293, CCDC 2473295, CCDC 2473297, CCDC 2473538 and CCDC 2473298). This data can be obtained free of charge via www.ccdc.cam.ac.uk/conts/retrieving.html (or from the Cambridge Crystallographic Data Centre, 12, Union Road, Cambridge CB2 1EZ, UK; fax: (+44) 1223-336-033; or deposit@ccdc.cam.ac.uk).

Table S17. Crystal data and structure refinement for compound **3** (CCDC 2473293).

Identification code	sh5338cu_a	
Empirical formula	C30 H39 N O8	
Formula weight	541.62	
Temperature	143(2) K	
Wavelength	1.54178 Å	
Crystal system	Monoclinic	
Space group	$P2_1$	
Unit cell dimensions	a = 13.1251(3) Å	α= 90°
	b = 7.09160(10) Å	β= 94.0770(10)°
	c = 15.8576(3) Å	γ = 90°
Volume	1472.26(5) $Å^3$	
Z	2	
Density (calculated)	1.222 Mg/m^3	
Absorption coefficient	0.724 mm^{-1}	
F(000)	580	
Crystal size	0.260 x 0.100 x 0.020 mm^3	
Theta range for data collection	2.794 to 74.511°	
Index ranges	-16<=h<=14, -8<=k<=8, -19<=l<=15	
Reflections collected	18396	
Independent reflections	5539 [$R_{(int)}$ = 0.0358]	
Completeness to theta = 67.679°	99.8 %	
Absorption correction	Semi-empirical from equivalents	
Max. and min. transmission	0.7538 and 0.6037	
Refinement method	Full-matrix least-squares on F^2	
Data / restraints / parameters	5539 / 6 / 371	
Goodness-of-fit on F^2	1.031	
Final R indices [I>2sigma(I)]	R_1 = 0.0288, wR_2 = 0.0759	
R indices (all data)	R_1 = 0.0304, wR_2 = 0.0768	
Absolute structure parameter	0.05(4)	
Extinction coefficient	n/a	
Largest diff. peak and hole	0.196 and -0.161 e.$Å^{-3}$	

Table S18. Crystal data and structure refinement for compound **4** (CCDC 2473295).

Identification code	sh5316_a	
Empirical formula	C28.39 H35.58 N1.19 O6	
Formula weight	489.49	
Temperature	143(2) K	
Wavelength	1.54178 Å	
Crystal system	Monoclinic	
Space group	C2	
Unit cell dimensions	a = 26.8258(6) Å	α= 90°
	b = 7.3430(2) Å	β= 119.1190(10)°
	c = 15.1404(4) Å	γ = 90°
Volume	2605.44(12) $Å^3$	
Z	4	
Density (calculated)	1.248 Mg/m^3	
Absorption coefficient	0.707 mm^{-1}	
F(000)	1049	
Crystal size	0.300 x 0.060 x 0.010 mm^3	
Theta range for data collection	3.623 to 70.159°	
Index ranges	-32<=h<=28, -8<=k<=8, -18<=l<=18	
Reflections collected	14253	
Independent reflections	4593 [$R_{(int)}$ = 0.0391]	
Completeness to theta = 67.679°	99.8 %	
Absorption correction	Semi-empirical from equivalents	
Max. and min. transmission	0.7533 and 0.5725	
Refinement method	Full-matrix least-squares on F^2	
Data / restraints / parameters	4593 / 397 / 422	
Goodness-of-fit on F^2	1.049	
Final R indices [I>2sigma(I)]	R_1 = 0.0334, wR_2 = 0.0857	
R indices (all data)	R_1 = 0.0349, wR_2 = 0.0867	
Absolute structure parameter	-0.09(8)	
Extinction coefficient	n/a	
Largest diff. peak and hole	0.214 and -0.174 e.$Å^{-3}$	

Table S19. Crystal data and structure refinement for compound **5** (CCDC 2473297).

Identification code	sh5635_a	
Empirical formula	C30 H37.84 N O6.42	
Formula weight	515.19	
Temperature	133(2) K	
Wavelength	1.54178 Å	
Crystal system	Monoclinic	
Space group	$P2_1$	
Unit cell dimensions	a = 13.0882(4) Å	α= 90°
	b = 7.1036(2) Å	β= 99.570(3)°
	c = 15.6644(6) Å	γ = 90°
Volume	1436.10(8) $Å^3$	
Z	2	
Density (calculated)	1.191 Mg/m^3	
Absorption coefficient	0.675 mm^{-3}	
F(000)	552	
Crystal size	0.220 x 0.040 x 0.020 mm^3	
Theta range for data collection	2.861 to 66.573°	
Index ranges	-14<=h<=15, -8<=k<=8, -18<=l<=18	
Reflections collected	20109	
Independent reflections	5026 [$R_{(int)}$ = 0.0930]	
Completeness to theta = 66.573°	99.6 %	
Absorption correction	Semi-empirical from equivalents	
Max. and min. transmission	0.7533 and 0.4892	
Refinement method	Full-matrix least-squares on F^2	
Data / restraints / parameters	5026 / 119 / 400	
Goodness-of-fit on F^2	1.050	
Final R indices [I>2sigma(I)]	R_1 = 0.0627, wR_2 = 0.1534	
R indices (all data)	R_1 = 0.1055, wR_2 = 0.1685	
Absolute structure parameter	-0.4(4)	
Extinction coefficient	n/a	
Largest diff. peak and hole	0.191 and -0.239 $e.Å^{-3}$	

Table S20. Crystal data and structure refinement for compound **11** (CCDC 2473538).

Identification code	sh5769_a	
Empirical formula	C31 H37 Cl4 N O7	
Formula weight	677.41	
Temperature	130(2) K	
Wavelength	0.71073 Å	
Crystal system	Monoclinic	
Space group	$P2_1$	
Unit cell dimensions	a = 13.0819(3) Å	α = 90°
	b = 7.6592(2) Å	β = 104.398(3)°
	c = 16.4463(5) Å	γ = 90°
Volume	1596.11(8) $Å^3$	
Z	2	
Density (calculated)	1.410 Mg/m^3	
Absorption coefficient	0.418 mm^{-3}	
F(000)	708	
Crystal size	0.300 x 0.040 x 0.020 mm^3	
Theta range for data collection	2.289 to 27.145°	
Index ranges	-16<=h<=16, -9<=k<=9, -21<=l<=20	
Reflections collected	23697	
Independent reflections	7040 [$R_{(int)}$ = 0.0473]	
Completeness to theta = 25.242°	99.9 %	
Absorption correction	Semi-empirical from equivalents	
Max. and min. transmission	1.0000 and 0.5465	
Refinement method	Full-matrix least-squares on F^2	
Data / restraints / parameters	7040 / 4 / 402	
Goodness-of-fit on F^2	1.054	
Final R indices [I>2sigma(I)]	R_1 = 0.0403, wR_2 = 0.1017	
R indices (all data)	R_1 = 0.0496, wR_2 = 0.1062	
Absolute structure parameter	0.00(3)	
Extinction coefficient	n/a	
Largest diff. peak and hole	0.430 and -0.367 e.$Å^{-3}$	

Table S21. Crystal data and structure refinement for compound **27** (CCDC 2473298).

Identification code	sh5689_a	
Empirical formula	C31 H38 Cl3 N O8	
Formula weight	658.97	
Temperature	130(2) K	
Wavelength	0.71073 Å	
Crystal system	Monoclinic	
Space group	$P2_1$	
Unit cell dimensions	a = 12.3045(5) Å	α= 90°
	b = 7.9979(3) Å	β= 104.627(4)°
	c = 16.9300(6) Å	γ = 90°
Volume	1612.09(11) $Å^3$	
Z	2	
Density (calculated)	1.358 Mg/m^3	
Absorption coefficient	0.334 mm^{-1}	
F(000)	692	
Crystal size	0.240 x 0.030 x 0.010 mm^3	
Theta range for data collection	2.355 to 27.120°	
Index ranges	-15<=h<=15, -9<=k<=10, -21<=l<=21	
Reflections collected	16865	
Independent reflections	6690 [$R_{(int)}$ = 0.0376]	
Completeness to theta = 25.242°	99.9 %	
Absorption correction	Semi-empirical from equivalents	
Max. and min. transmission	1.0000 and 0.5266	
Refinement method	Full-matrix least-squares on F^2	
Data / restraints / parameters	6690 / 4 / 402	
Goodness-of-fit on F^2	1.046	
Final R indices [I>2sigma(I)]	R_1 = 0.0446, wR_2 = 0.1129	
R indices (all data)	R_1 = 0.0527, wR_2 = 0.1171	
Absolute structure parameter	-0.03(3)	
Extinction coefficient	n/a	
Largest diff. peak and hole	0.788 and -0.718 e.$Å^{-3}$	

8. Bioassays

Assays in liver microsomes: For metabolic stability, the compound (1 µM) was incubated with 0.5 mg/mL pooled mouse (C57BL/6) microsomes (Xenotech, Kansas City, USA), 2 mM NADPH, 10 mM $MgCl_2$ at 37 °C for 120 min on a microplate shaker. The metabolic stability of testosterone, verapamil and ketoconazole were determined in parallel to confirm the enzymatic activity of mouse/rat liver microsomes. The incubation was stopped after defined time points by precipitation of aliquots of the incubation mixture with 2 volumes of cold internal standard solution (15 nM diphenhydramine in 10% methanol/acetonitrile). Samples were stored on ice until the end of the incubation and precipitated protein was removed by centrifugation (15 min, 4 °C, and 4,000 g). Concentration of the remaining test compound at the different time points was analyzed by HPLC-MS/MS (Vanquish Flex coupled to a TSQ Altis Plus, Thermo Fisher, Dreieich, Germany) and used to determine half-life (t1/2).

Plasma stability assays: To determine stability in plasma, the compound (1 µM) was incubated with pooled CD-1 mouse plasma (Neo Biotech, Nanterre, France). Samples were taken by mixing aliquots with 4 volumes of cold internal standard solution (12.5 nM diphenhydramine in 10% methanol/acetonitrile). The plasma stability of procain, propantheline and diltiazem were determined in parallel to confirm the enzymatic activity. Samples were stored on ice until the end of the incubation and precipitated protein was removed by centrifugation (15 min, 4 °C, 4,000 g, two centrifugation steps). Concentration of the remaining test compound at the different time points was analyzed by HPLC-MS/MS (Vanquish Flex coupled to a TSQ Altis Plus, Thermo Fisher, Dreieich, Germany). The plasma stability of procain, propantheline and diltiazem were determined in parallel to confirm the enzymatic activity.

Cytotoxicity assay: A549 (human lung carcinoma cell line; ACC 107) and CHO-K1 (chinese hamster ovary cells; ACC 110) were obtained from the German Collection of Microorganisms and Cell Cultures (DSMZ) and cultured under the conditions recommended by the depositor. Cells were propagated in Dulbeccos's Modified Eagle's Medium (DMEM) and Ham's F12 medium, respectively, supplemented with 10% fetal bovine serum (FBS), and seeded at $6×10^3$ cells per well of 96-well plates in 120 µL of complete medium. After 2 h of equilibration (37 °C, 5% CO_2), the cells were treated with a serial dilution of cytochalasins in 60 µL growth medium. Cytochalasins, doxorubicin as reference, as well as the solvent control were tested as duplicates in two independent experiments. After 5 d of incubation (37 °C, 5% CO2), a total of 20 µL of 5 mg/ml MTT (thiazolyl blue tetrazolium bromide) in phosphate-buffered saline (PBS) were added to each well

and the cells were further incubated for 2 h at 37 °C before the supernatant was discarded. Subsequently, the cells were washed with 100 µL of PBS and treated with 100 µl of 2-propanol/10 N HCl (250:1) to dissolve formazan granules. Cell viability was measured as percentage relative to the respective solvent control by measuring the absorbance at 570 nm using a microplate reader (Tecan Infinite M200Pro). GraphPad Prism (version 10.0.3, GraphPad, Boston, MA, USA) was used for sigmoidal curve fitting to determine the IC50 values.

9. References

[1] Schmid R, Heuckeroth S, Korf A, Smirnov A, Myers O, Dyrlund TS, Bushuiev R, Murray KJ, Hoffmann N, Lu M, Sarvepalli A, Zhang Z, Fleischauer M, Dührkop K, Wesner M, Hoogstra SJ, Rudt E, Mokshyna O, Brungs C, Ponomarov K, Mutabdžija L, Damiani T, Pudney CJ, Earll M, Helmer PO, Fallon TR, Schulze T, Rivas-Ubach A, Bilbao A, Richter H, Nothias LF, Wang M, Orešič M, Weng JK, Böcker S, Jeibmann A, Hayen H, Karst U, Dorrestein PC, Petras D, Du X, Pluskal T. Integrative analysis of multimodal mass spectrometry data in MZmine 3. Nat Biotechnol. 2023;41(4):447-449.

[2] Wang M, Carver JJ, Phelan VV, Sanchez LM, Garg N, Peng Y, Nguyen DD, Watrous J, Kapono CA, Luzzatto-Knaan T, Porto C, Bouslimani A, Melnik AV, Meehan MJ, Liu WT, Crüsemann M, Boudreau PD, Esquenazi E, Sandoval-Calderón M, Kersten RD, Pace LA, Quinn RA, Duncan KR, Hsu CC, Floros DJ, Gavilan RG, Kleigrewe K, Northen T, Dutton RJ, Parrot D, Carlson EE, Aigle B, Michelsen CF, Jelsbak L, Sohlenkamp C, Pevzner P, Edlund A, McLean J, Piel J, Murphy BT, Gerwick L, Liaw CC, Yang YL, Humpf HU, Maansson M, Keyzers RA, Sims AC, Johnson AR, Sidebottom AM, Sedio BE, Klitgaard A, Larson CB, P CAB, Torres-Mendoza D, Gonzalez DJ, Silva DB, Marques LM, Demarque DP, Pociute E, O'Neill EC, Briand E, Helfrich EJN, Granatosky EA, Glukhov E, Ryffel F, Houson H, Mohimani H, Kharbush JJ, Zeng Y, Vorholt JA, Kurita KL, Charusanti P, McPhail KL, Nielsen KF, Vuong L, Elfeki M, Traxler MF, Engene N, Koyama N, Vining OB, Baric R, Silva RR, Mascuch SJ, Tomasi S, Jenkins S, Macherla V, Hoffman T, Agarwal V, Williams PG, Dai J, Neupane R, Gurr J, Rodríguez AMC, Lamsa A, Zhang C, Dorrestein K, Duggan BM, Almaliti J, Allard PM, Phapale P, Nothias LF, Alexandrov T, Litaudon M, Wolfender JL, Kyle JE, Metz TO, Peryea T, Nguyen DT, VanLeer D, Shinn P, Jadhav A, Müller R, Waters KM, Shi W, Liu X, Zhang L, Knight R, Jensen PR, Palsson BO, Pogliano K, Linington RG, Gutiérrez M, Lopes NP, Gerwick WH, Moore BS, Dorrestein PC, Bandeira N. Sharing and community curation of mass spectrometry data with Global Natural Products Social Molecular Networking. Nat Biotechnol. 2016; 34:828-837.

[3] https://cytoscape.org/

[4] Bankevich A, Nurk S, Antipov D, Gurevich AA, Dvorkin M, Kulikov AS, Lesin VM, Nikolenko SI, Pham S, Prjibelski AD, et al. 2012. SPAdes: A New Genome Assembly Algorithm and Its Applications to Single-Cell Sequencing. Journal of Computational Biology 19(5): 455-477.

[5] Walker BJ, Abeel T, Shea T, Priest M, Abouelliel A, Sakthikumar S, Cuomo CA, Zeng Q, Wortman J, Young SK, et al. 2014. Pilon: an integrated tool for comprehensive microbial variant detection and genome assembly improvement. *PLOS ONE* 9(11): e112963.

[6] Smit A, Hubley R, Green P 2013-2015. RepeatMasker Open-4.0. http://www.repeatmasker.org.

[7] Gabriel L, Brůna T, Hoff KJ, Ebel M, Lomsadze A, Borodovsky M, Stanke M. 2024. BRAKER3: Fully automated genome annotation using RNA-seq and protein evidence with GeneMark-ETP, AUGUSTUS, and TSEBRA. *Genome Research* 34(5): 769-777.

[8] Simão FA, Waterhouse RM, Ioannidis P, Kriventseva EV, Zdobnov EM. 2015. BUSCO: assessing genome assembly and annotation completeness with single-copy orthologs. *Bioinformatics* 31(19): 3210-3212.

[9] Blin K, Shaw S, Augustijn HE, Reitz ZL, Biermann F, Alanjary M, Fetter A, Terlouw BR, Metcalf WW, Helfrich EJN, et al. 2023. antiSMASH 7.0: new and improved predictions for detection, regulation, chemical structures and visualisation. *Nucleic Acids Research* 51(W1): W46-W50.

[10] Emms DM, Kelly S. 2019. OrthoFinder: phylogenetic orthology inference for comparative genomics. *Genome Biology* 20(1): 238.

[11] Buchfink B, Reuter K, Drost H-G. 2021. Sensitive protein alignments at tree-of-life scale using DIAMOND. *Nature Methods* 18(4): 366-368.

[12] Price MN, Dehal PS, Arkin AP. 2010. FastTree 2 – Approximately Maximum-Likelihood Trees for Large Alignments. *PLOS ONE* 5(3): e9490.

[13] Emms DM, Kelly S. 2018. STAG: Species Tree Inference from All Genes. *bioRxiv*: 267914.

[14] Emms DM, Kelly S. 2017. STRIDE: Species Tree Root Inference from Gene Duplication Events. *Molecular Biology and Evolution* 34(12): 3267-3278.

[15] Katoh K, Standley DM. 2013. MAFFT Multiple Sequence Alignment Software Version 7: Improvements in Performance and Usability. *Molecular Biology and Evolution* 30(4): 772-780.

[16] Lemoine F, Correia D, Lefort V, Doppelt-Azeroual O, Mareuil F, Cohen-Boulakia S, Gascuel O. 2019. NGPhylogeny.fr: new generation phylogenetic services for non-specialists. *Nucleic Acids Research* 47(W1): W260-W265.

[17] Letunic I, Bork P. 2007. Interactive Tree Of Life (iTOL): an online tool for phylogenetic tree display and annotation. *Bioinformatics* 23(1): 127-128.

[18] Röttig M, Medema MH, Blin K, Weber T, Rausch C, Kohlbacher O. NRPSpredictor2--a web server for predicting NRPS adenylation domain specificity. Nucleic Acids Res. 2011;39:W362-7. doi: 10.1093/nar/gkr323.

[19] Li G, Schmidt S, Silué SK, Koné NA, Poulsen M. Phylogenetic analysis of termite-associated Xylaria from Africa reveals hidden diversity. Fungal Biol. 2025;129(1):101523. doi: 10.1016/j.funbio.2024.12.001.

[20] Fricke J, Schalk F, Kreuzenbeck NB, Seibel E, Hoffmann J, Dittmann G, Conlon BH, Guo H, Wilhelm de Beer Z, Vassão DG, Gleixner G, Poulsen M, Beemelmanns C. Adaptations of Pseudoxylaria towards a comb-associated lifestyle in fungus-farming termite colonies. ISME J. 2023;17(5):733-747.

[21] Mead ME, Raja HA, Steenwyk JL, et al (2019) Draft Genome Sequence of the Griseofulvin-Producing Fungus *Xylaria flabelliformis* Strain G536. Microbiol Resour Announc 8:14–16. https://doi.org/10.1128/MRA.00890-19

[22] Wibberg D, Stadler M, Lambert C, et al (2020) High quality genome sequences of thirteen Hypoxylaceae (Ascomycota) strengthen the phylogenetic family backbone and enable the discovery of new taxa. Fungal Divers. https://doi.org/10.1007/s13225-020-00447-5

[23] Büttner E, Liers C, Hofrichter M, et al (2019b) Draft Genome Sequence of Xylaria hypoxylon DSM 108379, a Ubiquitous Fungus on Hardwood. Microbiol Resour Announc 8:9–11. https://doi.org/10.1128/mra.00845-19

[24] Franco MEE, Wisecaver JH, Arnold AE, Ju YM, Slot JC, Ahrendt S, Moore LP, Eastman KE, Scott K, Konkel Z, Mondo SJ, Kuo A, Hayes RD, Haridas S, Andreopoulos B, Riley R, LaButti K, Pangilinan J, Lipzen A, Amirebrahimi M, Yan J, Adam C, Keymanesh K, Ng V, Louie K, Northen T, Drula E, Henrissat B, Hsieh HM, Youens-Clark K, Lutzoni F, Miadlikowska J, Eastwood DC, Hamelin RC, Grigoriev IV, U'Ren JM. Ecological generalism drives hyperdiversity of secondary metabolite gene clusters in xylarialean endophytes. New Phytol. 2022;233(3):1317-1330.

[25] Guo H, Schmidt A, Stephan P, Raguž L, Braga D, Kaiser M, Dahse HM, Weigel C, Lackner G, Beemelmanns C. Precursor-Directed Diversification of Cyclic Tetrapeptidic Pseudoxylallemycins. Chembiochem. 2018;19(21):2307-2311.

[26] Sheldrick, G. M. SHELXT – Integrated space-group and crystal-structure determination. *Acta Cryst. A* 2015;71:3–8.

[27] Sheldrick, G. M. Crystal structure refinement with SHELXL. *Acta Cryst. C* 2015;71:3–8.

[28] Hübschle, C. B., Sheldrick, G. M. & Dittrich, B. ShelXle: a Qt graphical user interface for SHELXL. *J. Appl. Cryst.* 2011;44:1281–1284.
